# Research on OpenCL optimization for FPGA deep learning application

**DOI:** 10.1371/journal.pone.0222984

**Published:** 2019-10-10

**Authors:** Shuo Zhang, Yanxia Wu, Chaoguang Men, Hongtao He, Kai Liang

**Affiliations:** College of Computer Science and Technology, Harbin Engineering University, Harbin 150001, China; National University of Sciences and Technology, PAKISTAN

## Abstract

In recent years, with the development of computer science, deep learning is held as competent enough to solve the problem of inference and learning in high dimensional space. Therefore, it has received unprecedented attention from both the academia and the business community. Compared with CPU/GPU, FPGA has attracted much attention for its high-energy efficiency, short development cycle and reconfigurability in the aspect of deep learning algorithm. However, because of the limited research on OpenCL optimization on FPGA of deep learning algorithms, OpenCL tools and models applied to CPU/GPU cannot be directly used on FPGA. This makes it difficult for software programmers to use FPGA when implementing deep learning algorithms for a rewarding performance. To solve this problem, this paper proposed an OpenCL computational model based on FPGA template architecture to optimize the time-consuming convolution layer in deep learning. The comparison between the program applying the computational model and the corresponding optimization program provided by Xilinx indicates that the former is 8-40 times higher than the latter in terms of performance.

## Introduction

Recently, artificial intelligence technology has attracted worldwide attention. As a new field of artificial intelligence, deep learning has an excellent strength to solve complex learning problems [1] [2]. However, with the progressive innovation of technology, the number of neural network models also increases rapidly. During the period from 2012 to 2018, as the number of neural network models increased, both the amount of model parameters and calculation increased rapidly. The size of the AlexNet model designed in 2012 and VGG-16 model designed in 2014 exceeded 200MB and 500MB respectively [3]. Meanwhile, the model parameters have increased from 60 million to 138 million. Hundreds of millions operations are required for each run. In order to improve performance, scholars have turned to designing more efficient deep neural networks [4].

In accelerating the application of deep learning, FPGA has attracted a lot of attention due to its advantages over GPU and ASIC. Compared with GPU, the acceleration design of FPGA is hardware design. Its power consumption is lower than GPU. The acceleration of FPGA can achieve higher performance under per power consumption. For example, in the reasoning stage of convolutional neural network, Microsoft team uses FPGA (Stratix V D5) to achieve the acceleration performance of 134 pictures processing per second and the power consumption is only 25 watts. If the superior FPGA (Arria 10 GX1150) is used, this acceleration performance is expected to 233 pictures processing per second, while the power consumption is basically unchanged. For high-performance GPU implementation (Caffe + cuDNN), the acceleration performance is 500-824 pictures processing per second, and the power consumption is 235 watts [5]. It means that FPGA has better energy efficiency compared with GPU [6] [7] [8] [9]. Unlike GPU and ASIC with fixed hardware architectures, FPGA is reconfigurable hardware, which means developers can connect the logical blocks within the FPGA through programmable connections to achieve their desired function [10]. This programmability enables developers to adjust their hardware design at any time according to the deep learning algorithm. However, hardware acceleration design based on FPGA requires software developers have a certain amount of hardware expertise, which is a high threshold for them. In recent years, FPGA programming environment has been greatly improved. Until now, the developers without corresponding hardware expertise have been allowed to develop FPGA with advanced programming languages such as C, C++ and OpenCL. It to some extent reduces the difficulty of FPGA development, shortens the FPGA development cycle and provides convenience for researchers and developers [11]. In order to reduce the difficulty of FPGA development, the key technologies in the automated high level synthesis tool chain are studied. These researches can be easily classified from different perspectives. From the perspective of the input language used by the user, it can be divided into C language and C−like language. The research uses C/C++ as its input language [12] [13] [14], this kind of research is divided into two categories when implementing automated generation of FPGA hardware architecture. One category of research is a complete automated generation tool chain. The process of generating hardware architecture is completely controllable, but the disadvantage is the insufficient universality of tools [14]. The other is to use the current mainstream hardware generation high level synthesis tool chain [12] [13], but it need to study the automation code generator in depth. The C/C++ language is translated by users to generate the input language supported by the commercial tool chain. The main research of this category is how to map one high-level language to another high-level language (such as OpenCL). Another kind of research work that directly uses C−like language (such as OpenCL) as an input language, focuses on different architecture of the CNN Accelerator [15] [16] [17]. However, because the same function of the program is implemented in different OpenCL code, the hardware architecture generated by the automation tool optimization is different. To implement efficient hardware circuits, developers need to constantly try to optimize various configuration combinations. Even though the push-button automated tool also requires iterative optimization. The key innovation point of this paper is proposed a computational model to help software engineers rationally design parameters without additional third-party tools, how to quickly reduce the iteratively written OpenCL code, and generate efficient hardware based on deeply loop pipelined architecture.

## Convolutional neural network

As is often used in image processing, convolutional neural network is one of the most classical models in deep learning. Given an image and using filtering to extract features, the machine will obtain an image called feature map [17]. The most commonly used Valid convolution assumes that the input feature is one dimension and the filter is one dimension, there are Eq (1):

$$\begin{array}{c} \;\{\begin{array}{c} y=con(x,w{,}^{\prime }valid^{\prime }=\left(y\left(1\right),\cdots ,y\left(t\right),\cdots ,y\left(n-m+1\right)\right)\in R^{n-m+1}\\ y\left(t\right)=\sum_{i=1}^{m}x\left(t+i-1\right)w\left(i\right) \end{array} \end{array}$$ (1)

In this equation, t = 1,2,…,n-m+1, and *n* > *m*.

In addition to CNN, most of the neurons in the neural network layer are fully connected. Although full-connected neurons can recognize more complex images, there are also some problems such as lack of flexibility and computational complexity. Convolution operation can reduce unnecessary weight connections to make the transformed images more robust.

The pooling layer is also called the subsampling layer. It usually connects with the convolution layer. Through partial correlation principle, on the one hand, it can improve the robustness of the system, and on the other hand, it can reduce the calculation of the characteristic pattern.

The propagation process in the maximum pooling layer is as Eq (2):

$$\begin{array}{c} y_{ij}^{\left(k\right)}=\text{max}\left(a_{\left(l_{1}i+s\right)\left(l_{2}j+t\right)}^{\left(k\right)}\right) \end{array}$$ (2)

In the formula, *L*_1_ and *L*_2_ represent the core pool size. For pooling layer and convolution layer, we tend to pool after convolution and put an activation function after convolution. The activation function is a simple non-linear operation, which improves the ability of non-linear characterization. With the process of convolution-activation function-pooling, CNN can obtain more robust features.

Deep learning convolutional neural networks bring is that the convolution layer needs to consume a lot of memory [18], especially in the training process, because back-propagation needs all the intermediate values of forward transmission. If the size of the input image is *H* × *W* and the filter size is *m* × *n*, the convolution can be expressed in the Eq (3):

$$\begin{array}{c} z_{ij}^{\left(k\right)}=\sum_{s=0}^{m-1}\sum_{t=0}^{n-1}w_{st}^{\left(k\right)}x\left(i+s\right)\left(j+t\right) \end{array}$$ (3)

In the equation, w is the weight of the kernel. However, the equation above is not enough with multiple convolution layers considered. Thus, a parameter is added to the kernel. The modified equation is as Eq (4):

$$\begin{array}{c} z_{ij}^{\left(k\right)}=\sum_{c}\sum_{s=0}^{m-1}\sum_{t=1}^{n-1}w_{st}^{\left(k,c\right)}x_{\left(i+s)(j+t\right)}^{\left(c\right)} \end{array}$$ (4)

In the equation, *c* represents the image channel. If the number of kernels is *k* and the channel is *c*, the convolution image size is (*M* − *m* + 1) × (*N* − *n* + 1) through the above equation. Assuming that the size of the convolution kernel is 5×5, 200 feature map with the size of 150×100 are needed to output. If the input is three channels, the whole process requires 225 million floating point multiplication. This process involves a large number of multiplication addition calculations, which requires a reasonable calculation computational model to improve the performance of the system. However, in the actual optimization, we should not only consider the optimization of computation, but also whether the storage resources on the FPGA chip can transmit the data needed for multiplication addition calculation at one time [19]. Assuming that ThroughputRate is the throughput of the system, it is affected by two aspects of computation and memory access. The relationship between system throughput and computation and memory access is shown in Eq (5):

$$\begin{array}{c} ThroughputRate=\text{min}(CalculatedPeak,MemoryPeak) \end{array}$$ (5)

*CalculatedPeak* is the peak of computing power of calculation resources, and MemoryPeak is the maximum floating point performance of the memory support. According to the above equation, the overall throughput of the system is less than the minimum value [20] of two items of calculation and memory access. The execution of the computation requires data support. The original data convolution layer computation requires are usually copied from Host memory to FPGA off-chip global memory by the Host program. When the FPGA use that data, it usually needs to be read from the off-chip global memory. Besides, the data generated after the execution of the FPGA also need to be written back to the Host memory through the off-chip global memory. However, FPGA off-chip global memory is not on the FPGA chip, and FPGA usually takes much time to make data interaction with it. If no optimization is carried out, it usually has a great impact on the performance of the program [21]. At the same time, convolution calculation involves a relatively large amount of data, and a lot of data also need to be reused in calculation, which results in low computational efficiency. The paper [22] proposed a parallel acceleration strategy of CNN based on FPGA with OpenCL by the use of Xilinx SDAccel. But there is no optimization details and method to configure the parameters, it is difficult for researchers to reproduce. To deal with the above problems, this paper proposes an OpenCL optimization strategy with an effective solution to the low memory efficiency and the extra overhead generated by repeatedly read / written data from FPGA out of memory.

## Loop pipelined architecture

The use of OpenCL tools to implement deep learning algorithms on FPGA greatly reduces the work of designers. For the process of the internal hardware architecture mapping of the FPGA is not considered, the development threshold is reduced. However, the process of converting OpenCL into FPGA bitstream through development tools is transparent to the designers, for whom it is hard to add into the project better hardware modules in other languages. In addition, most designers do not know the way to configure the bit width reasonably and the way improve the parallelism to make full use of the advantages of FPGA and the effects of data transfer on performance [23]. As a result, the deep learning algorithm designed by those designers has no obvious advantage in performance. In this section, an loop pipelined-architecture template is proposed for the optimization of convolutional neural network performance. By using the optimized architecture template given in this section and the configuration of specific technical parameters, it is verified by experiments that the performance of the algorithm can be improved effectively in the accelerated design of FPGA deep learning algorithm. The following section gives a detailed description of the optimized template architecture and configuration of technical parameters.

### Loop pipelined architecture abstraction

**Fig 1:**
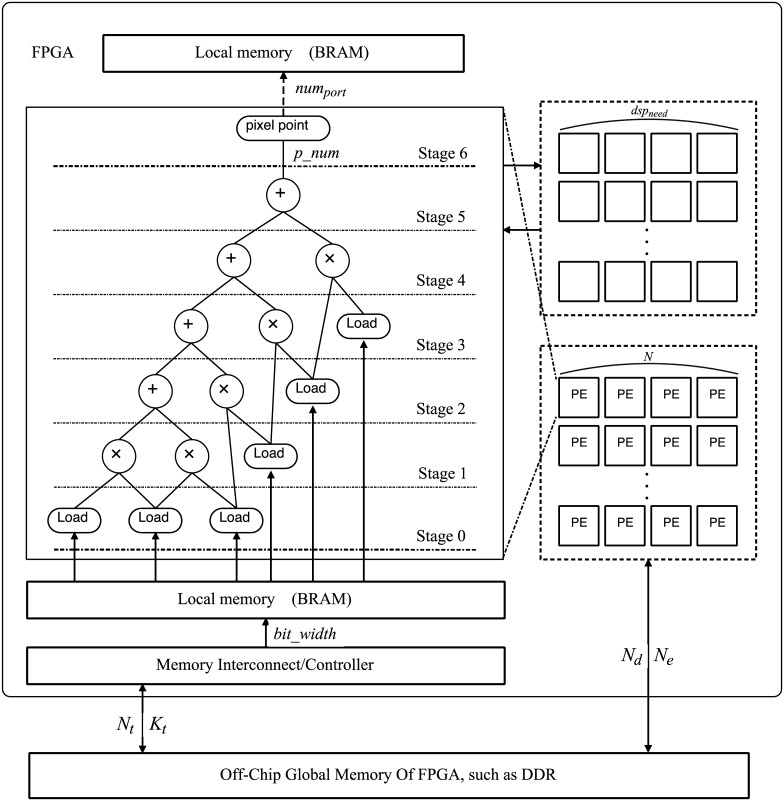
Hardware architecture template diagram.

Convolutional neural network algorithm involves a large number of computations. By making full use of the parallel characteristics of FPGA, convolution algorithm based on OpenCL on FPGA can be utilized to the advantages of FPGA. An optimized architecture template is proposed of OpenCL based on FPGA, which is shown in Fig 1.

### Template parameters

According to the parameterized optimization architecture diagram, the following parameters are needed for calculation and determination:

- Determining the parameter data *N*_*d*_, the number of elements in the vector data type is *N*_*e*_.
  $$\begin{array}{c} N_{d}=K_{n}\cdot N_{e} \end{array}$$ (6)
- Determining the width of the data port *bit_width*.
  $$\begin{array}{c} bit\_width=\{\begin{array}{c} num\_bit,num\_bit\in \{32,64,128,256,512\}\\ default,num\_bit\notin \{32,64,128,256,512\} \end{array} \end{array}$$ (7)
  In the equation, *nun_bit* is the number of data digits corresponding to the data type.
- The theoretical value of the total number of data transfer times is *N*_*t*_, and the average data amount of each data transfer is *K*_*t*_.
  $$\begin{array}{c} N_{t}=\sum_{i=1}^{n}\lceil \frac{N_{i}}{Bu_{i}}\rceil \end{array}$$ (8)
  $$\begin{array}{c} K_{t}=\frac{\sum_{i=1}^{n}N_{i}B_{i}}{N_{t}\cdot 8\cdot 1000} \end{array}$$ (9)
  In the equation, *N*_*i*_ is the total number of per variable data transfer and Bi is the data bits.
- The number of DSP needed for the calculation is *dsp_need*.
  $$\begin{array}{c} dsp_{need}=N_{unroll}\cdot K \end{array}$$ (10)
  In the equation, *N*_*unroll*_ is the unrolling loop degree in the unrolling loop scheme; *K* is the number of using DSP for each loop iteration. This value can be obtained from the resource use report. The number of DSP required for each multiplication operation can be obtained through the related development board documents, and then *K* also can be calculated.
- Calculate the number of sub parts of the array data memory, *p_num*.
  $$\begin{array}{c} p_{num=}\{\begin{array}{c} \text{min}(num,num_{\text{max}}),loop\;partition\\ \text{min}(\frac{num\_d}{addr\_i},num_{\text{max}}),block\;partition \end{array} \end{array}$$ (11)
  In the equation, *num_d* is the total number of data in the array. num is the number of consecutive data addresses for each calculation. *num_max* is the upper limit of the array partition supported by the compiler. *addr_i* is the address interval of each of the adjacent data.
- The cyclic boundary *L*_*b*_.
  $$\begin{array}{c} L_{b}=\{\begin{array}{c} x,cyclic\;boundary\;is\;variable\\ c,cyclic\;boundary\;is\;constant \end{array} \end{array}$$ (12)
- Determine the number of the configuration computing unit, *N*.
  $$\begin{array}{c} N<\text{min}\;(\frac{1}{B},\frac{1}{D},N_{k}) \end{array}$$ (13)
  In the equation, *B* and *D* are the percentages of BRAM resources consumed by a single cell and the percentage of DSP resources consumed by a single cell respectively, and *N*_*k*_ is the number of cells restricted by the compiler.
- The number of memory ports for storing data is numport, and the theoretical parallelism of the data calculation is *v_cal*. The maximum parallelism of data reading/writing is *v_data*, and these three parameters can be obtained from the program execution information.

## Computational model

1. The relevant parameters are obtained by calculation out of hardware architecture template drawing and parameter calculation equation. The specific OpenCL optimization technology and related parameters are selected by the following algorithm steps.
  (1) Judge whether the address stored in the off-chip global memory of the corresponding parameter is continuous. If so, entered (2); if not, the data vectorization is not optimized.
  (2) Judge whether the data quantity contained in parameter is suitable for data vectorization. *N*_*e*_ represents the number of elements in vector data type supported by OpenCL compiler. *N*_*e*_ ∈ {2, 3, 4, 8, 16}. Traverse the value of *N*_*e*_. If ∃*K*_*n*_ ∈ *N*^+^ can make *N*_*d*_ and *N*_*e*_ satisfy the formula (1), the value of *K*_*n*_ is recorded. After the traversal is completed, the whole value of *K*_*n*_ recorded is combined into a set called *L*. If the set *L* is not empty, enter (3). If the set is empty, the data vectorization optimization is not carried out.
  (3) The minimum value in the set is recorded as *L*_*min*_. The data are grouped in ascending order of size, and the number of groups is *L*_*min*_. The number of data in each group is *N*_*d*_/*L*_*min*_. After completing the grouping, each group of data are used as a whole to replace the original data in kernel. If they can be substituted equivalently and do not affect the correct execution of the program, they enter (4). Otherwise, remove the *L*_*min*_ from the set *L* and repeat (3).
  (4) Carry out data vectorization optimization. *N*_*d*_/*L*_*min*_ represents the number of elements contained in vector type data.
2. Configure the number of data ports and the bit width. The bit width of a data port is usually related to the data type of data transmitted through the port. At present, the OpenCL compiler supports a bit width of 32, 64, 128, 256 and 512 bits. If the data type corresponding to the data bit *num*_*bit* ∈ {32, 64, 128, 256, 512}, the port bit width is set as *num_bit*. Otherwise, keep the default setting. By default, the OpenCL compiler automatically configures the bit width of the data port according to the actual situation.
3. Assuming that there are n global variables involved in the data transfer(read), the total number of data transfers (read) per variable is *N*_1_, *N*_2_, ⋯, *N*_*n*_. The data digits are *B*_1_, *B*_2_, ⋯, *B*_*n*_, and the burst length of data read and write is *Bu*_1_, *Bu*_2_, ⋯, *Bu*_*n*_. The burst length of burst read-write model is usually 16, and the length of non-burst read-write model is 1. According to the procedure execution report, it is judged whether the new optimization is carried out. The steps are as follows:
  (1) Record the total number of data transfers (reading/writing) in the program execution report and the average amount of data (reading/writing) per data transfer. The values are *N*_*r*_, *N*_*w*_, *K*_*r*_, *K*_*w*_ respectively.
  (2) According to the Eqs (3) and (4), calculate the total number of data transfers (reading/writing) and the average amount of data (reading/writing) per data transfer after memory optimization. The values obtained are *N*_*tr*_, *N*_*tw*_, *K*_*tr*_, and *K*_*tw*_ respectively.
  (3) If *N*_*tr*_ is less than *N*_*r*_ (or *K*_*tr*_ is greater than *K*_*r*_) or *N*_*tw*_ is less than *N*_*w*_ (or *K*_*tw*_ is greater than *K*_*w*_), and the difference is larger, it is necessary to adjust the optimization; Otherwise, there is no need to be re-optimized.
4. The set *A* is all iterations in the nested loop to be analyzed. Unrolling loop and optimizing array partition are carried out according to the following process:
  (1) The analysis is started from the most inner loop in the set *A*. If the layer is already the outermost loop or the cycle order of the layer cannot be exchanged with the innermost loop, record the innermost loop and all loops that can exchange order with the inner loop as the set *B*. Remove the elements in set *B* from set *A* and enter (2); Otherwise, analyze the outer loop.
  (2) According to the Eq (10), the number of DSP dspneed required for the scheme is calculated. And then, compare dspneed with the total number of DSP on-chip dsptotal. If *dsp*_*need*_ < *dsp*_*total*_, and the array division that conforms to the computational parallelism of the scheme can be realized, enter (3); Otherwise, analyze the next scheme.
  (3) In this scheme, if all loops in set *B* are fully expanded and set *A* is not empty, then enter (1) and calculate the degree of parallelism; otherwise, enter (4).
  (4) Optimization is carried out according to the unrolling loop scheme and the corresponding array partition scheme. Analyze whether an array partition that satisfies the computation parallelism in (2) can be achieved. Next, analyze the data after the unrolling loop and group the data, and then the array stored on the same FPGA on-chip memory is divided into a set. Analyze each group of data sequentially, and select the corresponding analysis method according to its storage method on FPGA in the forms of one-dimensional array and multi-dimensional array. If all arrays can be partitioned to satisfy computational parallelism, it is shown that an efficient array partition can be made for the unrolling loop scheme. Otherwise, the effective array partition cannot be carried out.
  (5) It is analyzed from two aspects: one-dimensional array and multi-dimensional array.
    The steps of one-dimensional array analysis are given as follows:
    (a) Analyze the address characteristics of each calculation involving data after the loop unrolling. If the addresses are continuous, carry out cyclic division to the array and enter (c). If the address is not continuous but the interval is uniform, carry out block division to the array and enter (c). If the data address characteristics do not meet the both of the above conditions, enter (b). The calculation method of dividing the number of sub-parts storing array data memory is like Eq (11).
    (b) If *num*_*d* < *num*_*reg*, the array is to be divided entirely and enter (c), otherwise it cannot be effectively divided.*num_reg* is the total number of FPGA on-chip registers available.
    (c) Verify whether the parallelism of data reading/writing after array partition satisfies the parallelism of computation in the unrolling loop scheme. If it is satisfied, the array partition is effective and the array partition scheme is recorded; otherwise, the array partition is invalid.
    The steps of multi-dimensional array analysis are as follows:
    (a) According to the array dimension, the array is completely divided on this dimension. Verify whether the parallelism of data reading/writing after array partition satisfies the parallelism of computation in the unrolling loop scheme.
    (b) If there exists a dimension that can be realized, the multi-dimensional array is regarded as one dimension array of this dimension, and the analysis method is carried out according to the one dimension array; otherwise, it enters (c). It should be noted that in the analysis of complete partitioning, what needs to be considered is not the restriction of registers on the FPGA chip, but the number of subparts of the array partitioning *p*_*num* < *num*_*max*.
    (c) If *num*_*d* < *num*_*reg*, the multi-dimensional array is to be divided entirely, otherwise it cannot be effectively divided.
  (6) Optimization based on the cyclic pipelining is as follows:
    (a) In loop unrolling, if there is a circular boundary *L*_*b*_ which is *x*, no optimization is made; Otherwise, enter (2).
    (b) If there is a loop which enables all loops to unfold optimization, the involved cycles are to be its sub-cycles, with the most inner loop selected and enter (3); otherwise, the optimization of cyclic flow is not carried out.
    (c) If the *L*_*b*_ of all the sub loops in the inner loop is *c*, the optimization of the cyclic flow is performed; otherwise, it will not be optimized.
  (7) According to the program execution report, the percentage of BRAM(B) resources and the percentage of DSP(D) resources consumed by a single computing element are obtained. Calculate the values of 1/B and 1/D, and then compare them with *N*_*k*_ to find out the minimum, with the limit conditions for configuring the number of computing element *N* obtained according to the Eq (13).
  (8) According to the program execution report, the number of storing data memory ports is *num*_*port*_, and the theoretical parallelism of data calculation is *v*_*cal*. If the data is not stored in registers and *num*_*port*_ = 1, it can be judged that the computing does not make full use of data reading/writing parallelism, because the OpenCL compiler can allocate up to two data ports for each memory at most. To deal with this situation, it is generally necessary to re-optimize the calculation. If *num*_*port*_ = 2, it illustrates that the parallelism of data reading/writing is fully utilized. At this time, compare the values of A and B. If *v*_*call* > *v*_*data*, repartition the array.

## Experimental section

The compiler tool used in this experiment is the Xilinx SDx tool, and the FPGA development board produced by the Alpha Data company is the ADM-PCIE-7V3 board. Linux is the execution environment of the Host terminal. The specific environment of this experiment is shown in Table 1 and the specific configuration of the ADM-PCIE-7V3 board is given in Table 2.

**Table 1:** Experimental software and hardware environment.

software environment	operating system	Centos release 6.7 final
	compiler tools	SDx 2016.3
hardware environment	Host terminal processer	Intel (R) Xeon (R) CPU E5-2620 @ 2.00GHz
	FPGA development board	ADM-PCIE-7V3board

**Table 2:** ADM-PCIE-7V3 the specific configuration of the board.

FPGA	Logic unit	Host interface	DDR3	Network interface	Power dissipation (typical value)
Virtex-7 VX690t-2(28nm)	693K	PCIe Gen3 x8	16GB ECC DDR3 1333MT/sec	Dual SFP+(10GbE)	23-26W

### Optimization example

In this section, this paper introduces the OpenCL example of convolution layer on FPGA firstly. Based on this example, the computational model is applied to the convolution layer. Later the application of the computational model is explained in detail. Finally, the results of the optimized program execution are given.

#### Convolution layer OpenCL example on FPGA

In the convolutional neural network model, the operation of each convolution layer is consistent and it is convolution operation. The difference lies in data processing and the scale of data, so the optimization methods and ideas are basically the same in the optimization of different convolution layer. Accordingly, this section focuses on the example of a single convolution layer in convolutional neural network. The example program given in this section is an ordinary convolution layer program without any optimization, whose parameters are shown in Table 3.

**Table 3:** Parameters of a single convolution layer.

Input		Convolution Kernel			Output	
Feature Map Size	Channels	Kernel Size	Stride	Padding	Feature Map Size	Channels
27×27	48	5×5	1	2	27×27	256

The number of convolution kernel channels is 48, and the number of convolution kernels is 256. The convolution layer is mainly implemented in the OpenCL kernel program, and the Host is mainly responsible for configuring the environment required by the kernel program, calling the kernel program, carrying out data transfer with kernel, etc. The specific implementation of pseudo code is shown in Algorithm 1.

**Algorithm 1**: Realization of pseudo code in convolution layer

**Input**: *image, input feature map data   *weights, input weight data

**Output**: *out output feature map data

1 **async_work_group_copy**(local_image,image,i_channel*ISize*ISize, 0);

2 **async_work_group_copy**(local_weight,weights,o_channel*i_channel*WSize*WSize, 0);

3 index←0;

4 outputLoop: **for** *o_num*←*0 to o_channel* **do**

5  outYAxis: **for** *o_y*←*0 to OSize* **do**

6   outXAxis: **for** *o_x*←*0 to OSize* **do**

7    sum←0;

8    convInchan: **for** *conv_num*←*0 to i_channel* **do**

9     convILoop:**for** *conv_y*←*0 to WSize* **do**

10      convJLoop: **for** *conv_x*←*0 to WSize* **do**

11       x_padding←o_x*Stride+con_x-Padding;

12       y_padding←o_y*Stride+con_y-Padding;

13       **if** *0*≤*y_padding*<*Isize and 0*≤*x_padding*<*Isize* **then**

14        sum += local_image[con_num * ISize * ISize+y_padding * ISize + x_padding]*local_weight[((o_num*i_channel + conv_num)*WSize+conv_y)*WSize+conv_x];

15       **end**

16      **end**

17     **end**

18    **end**

19    local_out[index++]=sum;

20   **end**

21  **end**

22 **end**

23 **async_work_group_copy**(out, local_out, o_channel*OSize*OSize, 0);

The original convolution layer program is compiled, deployed and run to get information about data transfer as shown in Table 4. where it can be seen that there are multiple data transfers between the kernel of convolution layer and the off-chip global memory, and the average data amount for each data transfer is only 4 bytes. Therefore, the data transfer efficiency is low.

**Table 4:** Related information of data transfer in the convolution layer basic program.

	Transfer Type	Number of Transfers	Transfer Rate(MB/s)	AvgBandwidth Utilization(%)	Avg Size(KB)	Avg Time(ns)
Data Transfer: Kernels and Global Memory	Read	408969216	63.049	0.547	0.004	27.240
	Write	186624	0.029	2.4975E-4	0.004	15.000

#### Example optimization scheme

The basic program of convolution layer is optimized according to the hardware architecture diagram and computational model proposed in the third and fourth section. The main work of this section is to apply the computational model to the example program, and give the parameters needed for each step and the detailed optimization scheme.

According to the first step of the computational model, the amount of data *N*_*d*_ contained in the parameter is 307200. Traverse *N*_*e*_, and figure out *K*_*n*_ ∈ {19200, 38400, 76800, 102400, 153600} according to the parameter calculation Eq (6), and *K*_*n*_ is not empty. Take out the minimum value 19200. The data are grouped in ascending order according to the address. The number of groups is 19200, and the number of data in each group is 16. Carry out the vector optimization to the data which can equivalently replace the original data used in the kernel.

According to the second step of the computational model, the global parameter is set to be _global int 16* pre, and the bit width of data transfer between the computing unit and the memory interconnection / memory controller is set to be 16×32, namely, 512 bits. According to the parameter Eq (8), the bit width is determined to be 512 bits, which makes it accessible to support a single data transfer of 512 bits. Thus, the number of data transfer each time varies-from 1 to 16, which can effectively reduce the number of memory transfer data.

According to the third step of the computational model, *N*_*t*_ and *K*_*t*_ are calculated by the parameter calculation Eqs (8) and (9). For *N*_*tr*_ is larger than *N*_*r*_ in the program execution report and *N*_*tw*_ is larger than *N*_*w*_ in the execution report in the example, there is no need to re-optimize this time.

The data transfer between convolution layer kernel and off-chip global memory can be obtained after the first three steps of optimization according to the computational model, as shown in Table 5, which indicates that the number of data transfers between the convolution layer kernel program and the global memory off-chip is greatly reduced, and the average amount of data transfer is increased to 64 bytes.

**Table 5:** Information related to data transfer after memory optimization.

	Transfer Type	Number of Transfers	Transfer Rate(MB/s)	AvgBandwidth Utilization(%)	Avg Size(KB)
Data Transfer: Kernels and Global Memory	Read	21387	1.198	0.010	0.064
	Write	11664	0.654	0.006	0.064

**Fig 2:**
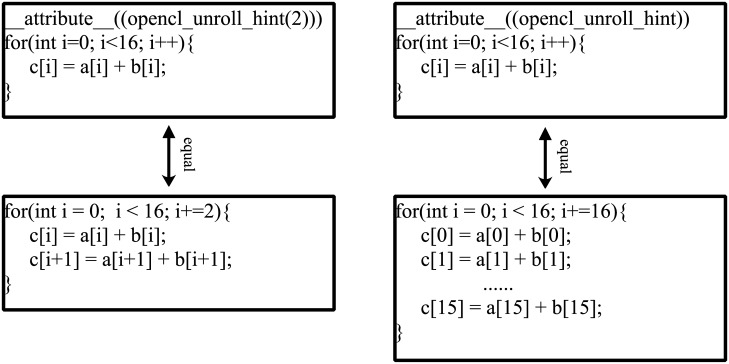
Example of using unrolling loop instruction.

According to the fourth step of the computational model, the *dsp*_*need*_ is figured out to be 251 by the parameter calculation Eq (10). The total number of DSP on-chip is 3600. The number of DSP needed is less than the total number and set *B* is fully unrolled loop. Fig 2 shows the use of optimized instruction and the equivalent code after unrolling. Graph (a) shows the equivalent code when the unrolling factor is 2, and graph (b) shows the equivalent code when the unrolling factor is by default.

For the size of convolution kernel is 5×5, the theoretical parallelism of computation is 25. However, the input characteristic graph data involved in the calculation and the convolution kernel data are stored locally in the form of one-dimensional arrays. Without the array partition optimization, the OpenCL compiler only assigns two ports to it at most. That is, the degree of parallelism of reading is 2, which is much less than the degree of parallelism of calculation, so the arrays need to implement array partitioning.

**Fig 3:**
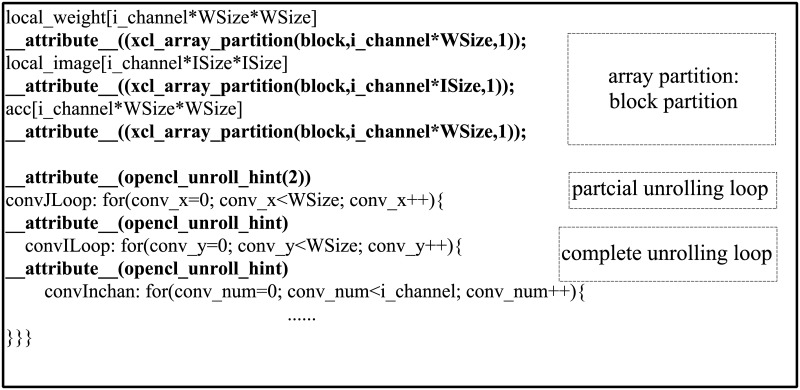
The specific code optimization of convolution multiplication calculation.

According to the fifth step of the computational model, the *p_num* of cyclic partitioning and block partitioning are calculated by Eq (11). Since the total number of *num_d* in the array is less than that of *num_reg* of available registers on-chip, the array is completely partitioned. In the process of loop unwrapping and array partitioning optimization, the last three layers of the convolution layer implementation code (calculation of single pixel in output characteristic graph) are optimized and the corresponding array partitioning is carried out. Meanwhile, for the convenience of optimization, this section divides the last three layers into double three-layer according to convolution multiplication and addition. For the two three-layer loops are consistent in architecture, the corresponding optimization strategies are nearly identical. The specific code of the optimization of the convolution multiplication calculation is shown as in Fig 3. In this optimization, the inner and outer two-layer cycles are completely unrolled, and the theoretical calculation parallel degree is 48×5, namely 240. The theoretical value of data reading/writing parallelism involved in the calculation is 48×5×2, namely 480. Since the parallelism of data reading and writing is greater than that of computing, the outer loop is partially unrolled in order to match the parallelism of the both and the unrolling factor is 2 in this optimization.

**Fig 4:**
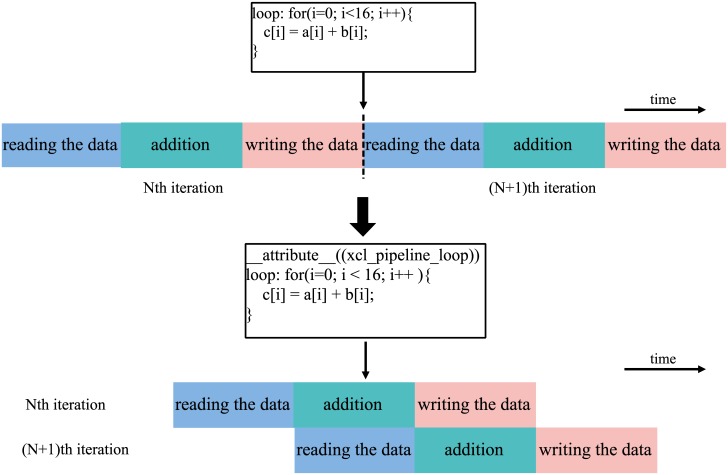
Using examples of loop instructions.

According to the sixth step of the computational model, the cyclic pipelining instruction __attribute__ ((xcl_pipeline_loop)) can be used when writing an OpenCL program. The main function of this instruction is to ensure the FPGA performs each iteration of the loop in a pipelining manner by adding the instruction outside of the for-loop. The entire loop boundaries are constants and the *L*_*b*_ is obtained from Eq (12) to optimize cyclic pipelining for *c*. Fig 4 shows the use of loop instructions and the execution situation of loops before and after the use of instructions. Figure (a) gives the performance of the cycle execution without using loop pipelining optimization, and figure (b) demonstrates the use of loop pipelining.

**Fig 5:**
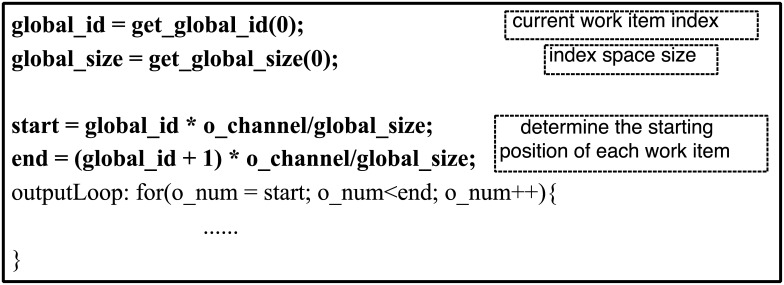
Kernel optimization code for multiple computing units.

According to the seventh step of the computational model, the 1/B and 1/D in the Eq (13) are 22.1 and 14.3 respectively according to the execution report. The compiler limits the number of computing units to 10. According to the parameter calculation Eq (13), the number of *N* is less than 10. In this range, it is the best to use six computing units for this example program, so six computational are configured elements this time. The kernel program is split into 6 working groups, with each containing only one work items and the specific OpenCL kernel optimization code shown in Fig 5. The output characteristic graph data are stored in order according to the channel. To make the address space of the output data in the off-chip global memory continuous when each computing unit transmits data with the off-chip global memory, this division of the kernel program is based on the number of channels in the output characteristic graph.

According to the eighth step of the computational model, unrolling loop and array partition optimization of the last three layer loops of convolution operation are carried out. The degree of parallelism of the optimized convolution multiplication is 480, which is two fifths of that of the ideally optimized convolution multiplication. Multiple computing units optimization of the outermost loop of the first three layers is carried out, and the parallelism of the output feature image pixel calculation after optimization is 6, which is much less than that of the pixel calculation of the output feature image after optimization. The optimization of cyclic flow is carried out for the inner loop. Finally, by comparing the values of additions, it is found that there is no need to repartition the array.

#### Optimization performance analysis of the program

According to the computational model proposed, the example code is directed toward optimization, whose result compared to the latest Xilinx optimization program [17] is shown in Table 6. From the runtime of each cell and the whole kernel, it is found that these four cells are basically executed in parallel.

**Table 6:** Optimized kernel program execution time.

Kernel Execution		Total Times(ms)				
		9.760				
	Computational Unit	Global Work Size	Local Work Size	Number of Calls	Xilinx(ms)	Optimized Program
Computational Unit Utilization	1	6:1:1	1:1:1	1	290.751	8.730
	2	6:1:1	1:1:1	1	290.859	8.930
	3	6:1:1	1:1:1	1	291.721	9.026
	4	6:1:1	1:1:1	1	291.684	8.887

The final optimization result of this example program is shown in Table 7. The final execution time of the example program is 9.76 milliseconds after optimization. Moreover, this paper also tests the performance of the convolution layer optimization program provided by Xilinx, as summarized in Table 5 where it can be seen that the final performance of the program is 29 times higher than that of the optimization program provided by Xilinx company.

**Table 7:** Performance comparison of the different optimization programs in the convolution layer OpenCL.

	Original Convolution Layer Program	The Optimization Program Provided by Xilinx Company	The Example Optimization Program	Speedup Ratio
Execution Time(ms)	1142.26	291.977	9.76	29x

The final optimization results of this experiment are compared with the CPU implementation [20], as indicated in Table 8. The 1-thread in the table is set as single thread execution, and the 16-thread is set as the 16-thread execution. -O3 represents the optimization level of a compiler is -O3.

**Table 8:** Comparison of optimization examples and CPU implementation.

	Intel Xeon 2.20GHz		FPGA
	1-thread –O3	16-thread –O3	
Execution Time (ms)	94.66	27.0	9.76
Speedup Ratio	1x	3.5x	9.7x
Power(Watt)	95.00	95.00	25.00
Energy(J)	8.99	2.57	0.24

From Table 8, it can be seen that the performance of the optimized convolution on FPGA is 9.76 times higher than that of single-thread CPU, 2.8 times higher than that of 16-thread CPU. Also, it is indicated that the energy consumption of the convolution program optimized by the computational model proposed and implemented on FPGA is significantly lower than that of CPU.

### Comparison and analysis of different scale convolution programs

In order to analyze the performance of different scale convolution programs, eight kinds of convolution layer programs are set up according to the ascending order as shown in Table 9. Layer 1 is one of scales, and the number of input and output channels is 64 and 128 respectively. Input a picture sample of a size of 111164 with the convolution kernel of 3364 size, the number of convolution kernels are 128 and the step size is 1. The result of the final output after the convolution operation is 1111128. The other 7 scales of convolution program analysis methods are the same with Layer1.

**Table 9:** CNN configurations.

Layer	1	2	3	4	5	6	7	8
Input_Ch	64	128	64	48	64	48	64	96
Output_Ch	128	192	96	96	128	128	96	128
Row	11	13	17	19	23	27	33	36
Col	11	13	17	19	23	27	33	36
Kernel	3×3	3×3	3×3	3×3	3×3	3×3	3×3	3×3
Stride	1	1	1	1	1	1	1	1

**Fig 6:**
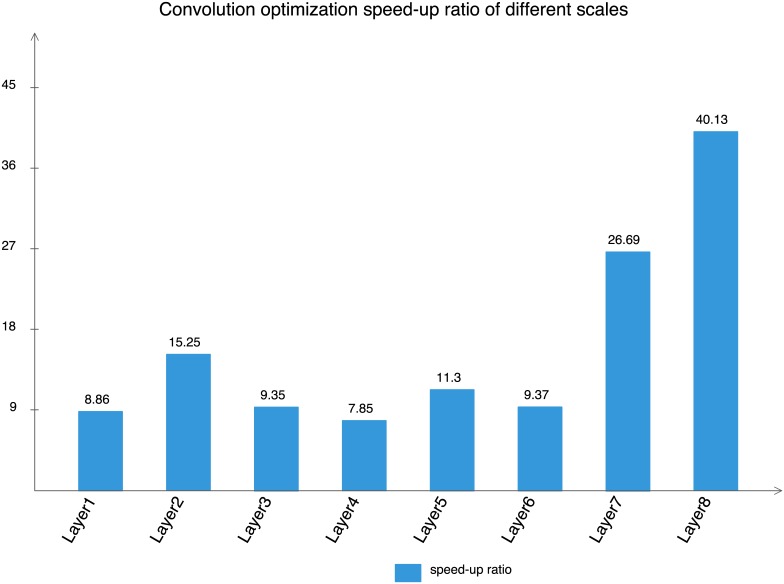
Optimized speed-up ratio of different scale convolution.

The computational model proposed is applied to the convolution layer of different scale, and its performance is compared with the corresponding optimization program provided by Xilinx company, as shown in Table 10. Convolution of different scales is optimized based on the computational model proposed, and its optimized time consumption is significantly reduced while compared with that of the optimization program by Xilinx. Fig 6 shows the speed-up ratio of Layer1L̃ayer8 where the higher the speed-up ratio, the better the optimization effect is. Accordingly, the optimized program has higher performance than optimized program of Xilinx.

**Table 10:** Performance comparison of different convolution scale optimization program with Xilinx company.

Convolution scale	Execution time (ms)	
	The optimization program provided by Xilinx company	The program optimized by the computational model
Layer1	12.4	1.4
Layer2	48.81	3.2
Layer3	21.5	2.3
Layer4	20.4	2.6
Layer5	52	4.6
Layer6	54.5	5.6
Layer7	216.2	8.1
Layer8	508.4	12.67

This paper put the code link into the paper openly accessible for other researchers to study and explore new accelerator method for deep neural networks. It can be found at the following link: https://github.com/PoetryAndWine/FPGA_CNN_Acceleration.

## Conclusion

This paper proposes an computational model based on OpenCL, which enables the transformation of the OpenCL model on GPU/CPU to FPGA. This computational model is used to help software programmers without fundamental hardware knowledge for a quick implementation in deep learning algorithm with high performance using FPGA. In terms of performance, the computational model not only reduces the cost of data interaction, but also improves the efficiency of data calculation. In terms of adaptability, the computational model is flexible and suitable for convolution layers of different sizes. The results of the proposed computational model applied to convolution layers of different scales show that the performance of the proposed computational model is 8-40 times higher than that of the corresponding optimization program provided by Xilinx Company.

## Supporting information

S1 File Convolution layer optimization code and the performance data. (Xilinx and this paper).
(ZIP)
