# Supplementary material for: Research on OpenCL optimization for FPGA deep learning application
Source: PLoS One. 2019 Oct 10;14(10):e0222984. doi: 10.1371/journal.pone.0222984 (PMC6786543; doi:10.1371/journal.pone.0222984)
Supplement: S1 File — (Xilinx and this paper). (ZIP) [file pone.0222984.s001.zip › SupportingFiles/Xlinx original data.zip!/Xlinx original data/sdaccel_timeline_trace.html]

# SDAccel Timeline Trace

  

### Generated on: 2019-02-02 00:51:07

### Profiled application: Xilinx\_fpgacnn\_best.exe

### Target platform: Xilinx

  

| Time (msec) | Name | Event | Address/Port | Size (Bytes or Num) | Latency (cycles) | Start (cycles) | End (cycles) | Latency (usec) | Start (msec) | End (msec) |
| --- | --- | --- | --- | --- | --- | --- | --- | --- | --- | --- |
| 0.386848 | clGetPlatformIDs\|General | START |  |  |  |  |  |  |  |  |
| 0.430738 | clGetPlatformIDs\|General | END |  |  |  |  |  |  |  |  |
| 0.441963 | clGetPlatformIDs\|General | START |  |  |  |  |  |  |  |  |
| 0.45079 | clGetPlatformIDs\|General | END |  |  |  |  |  |  |  |  |
| 0.460271 | clGetPlatformInfo\|General | START |  |  |  |  |  |  |  |  |
| 0.471372 | clGetPlatformInfo\|General | END |  |  |  |  |  |  |  |  |
| 0.480348 | clGetPlatformInfo\|General | START |  |  |  |  |  |  |  |  |
| 0.488841 | clGetPlatformInfo\|General | END |  |  |  |  |  |  |  |  |
| 0.504989 | clGetDeviceIDs\|General | START |  |  |  |  |  |  |  |  |
| 0.515324 | clGetDeviceIDs\|General | END |  |  |  |  |  |  |  |  |
| 0.527223 | clGetDeviceInfo\|General | START |  |  |  |  |  |  |  |  |
| 0.539148 | clGetDeviceInfo\|General | END |  |  |  |  |  |  |  |  |
| 0.54774 | clGetDeviceInfo\|General | START |  |  |  |  |  |  |  |  |
| 0.556229 | clGetDeviceInfo\|General | END |  |  |  |  |  |  |  |  |
| 0.567612 | clCreateContext\|General | START |  |  |  |  |  |  |  |  |
| 1702.10527 | clGetPlatformIDs\|General | START |  |  |  |  |  |  |  |  |
| 1702.171877 | clGetPlatformIDs\|General | END |  |  |  |  |  |  |  |  |
| 1702.195091 | clCreateContext\|General | END |  |  |  |  |  |  |  |  |
| 1702.209546 | clCreateCommandQueue\|General | START |  |  |  |  |  |  |  |  |
| 1702.224282 | clCreateCommandQueue\|General | END |  |  |  |  |  |  |  |  |
| 1710.753039 | clCreateProgramWithBinary\|General | START |  |  |  |  |  |  |  |  |
| 3999.223405 | clCreateProgramWithBinary\|General | END |  |  |  |  |  |  |  |  |
| 3999.292192 | clBuildProgram\|General | START |  |  |  |  |  |  |  |  |
| 3999.310292 | clBuildProgram\|General | END |  |  |  |  |  |  |  |  |
| 3999.684217 | clCreateKernel\|General | START |  |  |  |  |  |  |  |  |
| 3999.713687 | clCreateKernel\|General | END |  |  |  |  |  |  |  |  |
| 3999.752233 | clCreateBuffer\|General | START |  |  |  |  |  |  |  |  |
| 3999.791001 | clCreateBuffer\|General | END |  |  |  |  |  |  |  |  |
| 3999.802982 | clCreateBuffer\|General | START |  |  |  |  |  |  |  |  |
| 3999.817625 | clCreateBuffer\|General | END |  |  |  |  |  |  |  |  |
| 3999.829015 | clCreateBuffer\|General | START |  |  |  |  |  |  |  |  |
| 3999.845766 | clCreateBuffer\|General | END |  |  |  |  |  |  |  |  |
| 3999.859017 | clEnqueueWriteBuffer\|29507352 | START |  |  |  |  |  |  |  |  |
| 3999.893974 | WRITE\_BUFFER | QUEUE | 0X1C43790 | 139968 |  |  |  |  |  |  |
| 3999.951687 | WRITE\_BUFFER | SUBMIT | 0X1C43790 | 139968 |  |  |  |  |  |  |
| 4000.063631 | WRITE\_BUFFER | START | 0X1C43790 | 139968 |  |  |  |  |  |  |
| 4000.203617 | WRITE\_BUFFER | END | 0X1C43790 | 139968 |  |  |  |  |  |  |
| 4000.22085 | clEnqueueWriteBuffer\|29507352 | END |  |  |  |  |  |  |  |  |
| 4000.282717 | clEnqueueWriteBuffer\|29507352 | START |  |  |  |  |  |  |  |  |
| 4000.313543 | WRITE\_BUFFER | QUEUE | 0X1C42AA0 | 1228800 |  |  |  |  |  |  |
| 4000.388215 | WRITE\_BUFFER | SUBMIT | 0X1C42AA0 | 1228800 |  |  |  |  |  |  |
| 4000.474537 | WRITE\_BUFFER | START | 0X1C42AA0 | 1228800 |  |  |  |  |  |  |
| 4001.329177 | WRITE\_BUFFER | END | 0X1C42AA0 | 1228800 |  |  |  |  |  |  |
| 4001.366363 | clEnqueueWriteBuffer\|29507352 | END |  |  |  |  |  |  |  |  |
| 4001.401627 | clSetKernelArg\|General | START |  |  |  |  |  |  |  |  |
| 4001.429218 | clSetKernelArg\|General | END |  |  |  |  |  |  |  |  |
| 4001.439443 | clSetKernelArg\|General | START |  |  |  |  |  |  |  |  |
| 4001.449471 | clSetKernelArg\|General | END |  |  |  |  |  |  |  |  |
| 4001.458946 | clSetKernelArg\|General | START |  |  |  |  |  |  |  |  |
| 4001.472196 | clSetKernelArg\|General | END |  |  |  |  |  |  |  |  |
| 4001.481873 | clSetKernelArg\|General | START |  |  |  |  |  |  |  |  |
| 4001.491679 | clSetKernelArg\|General | END |  |  |  |  |  |  |  |  |
| 4001.50098 | clSetKernelArg\|General | START |  |  |  |  |  |  |  |  |
| 4001.510846 | clSetKernelArg\|General | END |  |  |  |  |  |  |  |  |
| 4001.519994 | clSetKernelArg\|General | START |  |  |  |  |  |  |  |  |
| 4001.529663 | clSetKernelArg\|General | END |  |  |  |  |  |  |  |  |
| 4001.594505 | clEnqueueNDRangeKernel\|29507352 | START |  |  |  |  |  |  |  |  |
| 4001.635643 | clEnqueueNDRangeKernel\|29507352 | END |  |  |  |  |  |  |  |  |
| 4001.649403 | clFinish\|General | START |  |  |  |  |  |  |  |  |
| 4002.137779 | KERNEL\|xilinx:adm-pcie-7v3:1ddr:3.0-0\|cnn\_GOOD\|good\_cnn\|1:1:1\|good\_cnn\_1 | START | 0X1C408A8 | 1 |  |  |  |  |  |  |
| 4002.262033 | KERNEL\|xilinx:adm-pcie-7v3:1ddr:3.0-0\|cnn\_GOOD\|good\_cnn\|1:1:1\|good\_cnn\_2 | START | 0X1C408A8 | 1 |  |  |  |  |  |  |
| 4002.344083 | KERNEL\|xilinx:adm-pcie-7v3:1ddr:3.0-0\|cnn\_GOOD\|good\_cnn\|1:1:1\|good\_cnn\_3 | START | 0X1C408A8 | 1 |  |  |  |  |  |  |
| 4002.400078 | KERNEL\|xilinx:adm-pcie-7v3:1ddr:3.0-0\|cnn\_GOOD\|good\_cnn\|1:1:1\|good\_cnn\_4 | START | 0X1C408A8 | 1 |  |  |  |  |  |  |
| 4292.888757 | KERNEL\|xilinx:adm-pcie-7v3:1ddr:3.0-0\|cnn\_GOOD\|good\_cnn\|1:1:1\|good\_cnn\_1 | END | 0X1C408A8 | 1 |  |  |  |  |  |  |
| 4293.12103 | KERNEL\|xilinx:adm-pcie-7v3:1ddr:3.0-0\|cnn\_GOOD\|good\_cnn\|1:1:1\|good\_cnn\_2 | END | 0X1C408A8 | 1 |  |  |  |  |  |  |
| 4294.065564 | KERNEL\|xilinx:adm-pcie-7v3:1ddr:3.0-0\|cnn\_GOOD\|good\_cnn\|1:1:1\|good\_cnn\_3 | END | 0X1C408A8 | 1 |  |  |  |  |  |  |
| 4294.084307 | KERNEL\|xilinx:adm-pcie-7v3:1ddr:3.0-0\|cnn\_GOOD\|good\_cnn\|1:1:1\|good\_cnn\_4 | END | 0X1C408A8 | 1 |  |  |  |  |  |  |
| 4294.140353 | clFinish\|General | END |  |  |  |  |  |  |  |  |
| 4294.183972 | clGetEventProfilingInfo\|General | START |  |  |  |  |  |  |  |  |
| 4294.196733 | clGetEventProfilingInfo\|General | END |  |  |  |  |  |  |  |  |
| 4294.206372 | clGetEventProfilingInfo\|General | START |  |  |  |  |  |  |  |  |
| 4294.215533 | clGetEventProfilingInfo\|General | END |  |  |  |  |  |  |  |  |
| 4294.226936 | clEnqueueReadBuffer\|29507352 | START |  |  |  |  |  |  |  |  |
| 4294.250127 | READ\_BUFFER | QUEUE | 0X1C422D0 | 746496 |  |  |  |  |  |  |
| 4294.288524 | READ\_BUFFER | SUBMIT | 0X1C422D0 | 746496 |  |  |  |  |  |  |
| 4294.383512 | READ\_BUFFER | START | 0X1C422D0 | 746496 |  |  |  |  |  |  |
| 4295.031023 | READ\_BUFFER | END | 0X1C422D0 | 746496 |  |  |  |  |  |  |
| 4295.058271 | clEnqueueReadBuffer\|29507352 | END |  |  |  |  |  |  |  |  |
| 4295.111869 | clReleaseMemObject\|General | START |  |  |  |  |  |  |  |  |
| 4295.132449 | clReleaseMemObject\|General | END |  |  |  |  |  |  |  |  |
| 4295.142772 | clReleaseMemObject\|General | START |  |  |  |  |  |  |  |  |
| 4295.152542 | clReleaseMemObject\|General | END |  |  |  |  |  |  |  |  |
| 4295.16217 | clReleaseMemObject\|General | START |  |  |  |  |  |  |  |  |
| 4295.172004 | clReleaseMemObject\|General | END |  |  |  |  |  |  |  |  |
| 4295.183634 | clReleaseKernel\|General | START |  |  |  |  |  |  |  |  |
| 4295.194637 | clReleaseKernel\|General | END |  |  |  |  |  |  |  |  |
| 4295.205737 | clReleaseProgram\|General | START |  |  |  |  |  |  |  |  |
| 4295.216821 | clReleaseProgram\|General | END |  |  |  |  |  |  |  |  |
| 4295.228045 | clReleaseCommandQueue\|General | START |  |  |  |  |  |  |  |  |
| 4295.23896 | clReleaseCommandQueue\|General | END |  |  |  |  |  |  |  |  |
| 4295.249978 | clReleaseContext\|General | START |  |  |  |  |  |  |  |  |
| 4295.260665 | clReleaseContext\|General | END |  |  |  |  |  |  |  |  |
| 4002.138052 | Kernel\_Read\|xilinx:adm-pcie-7v3:1ddr:3.0-0\|cnn\_GOOD | Read | OCL Region | 1 | 1 | 103813 | 103814 | 0.00333333 | 4002.138052 | 4002.138056 |
| 4002.138059 | Kernel\_Read\|xilinx:adm-pcie-7v3:1ddr:3.0-0\|cnn\_GOOD | Read | OCL Region | 1 | 1 | 103815 | 103816 | 0.00333333 | 4002.138059 | 4002.138062 |
| 4002.138069 | Kernel\_Read\|xilinx:adm-pcie-7v3:1ddr:3.0-0\|cnn\_GOOD | Read | OCL Region | 1 | 1 | 103818 | 103819 | 0.00333333 | 4002.138069 | 4002.138072 |
| 4002.138086 | Kernel\_Read\|xilinx:adm-pcie-7v3:1ddr:3.0-0\|cnn\_GOOD | Read | OCL Region | 1 | 1 | 103823 | 103824 | 0.00333333 | 4002.138086 | 4002.138089 |
| 4002.138109 | Kernel\_Read\|xilinx:adm-pcie-7v3:1ddr:3.0-0\|cnn\_GOOD | Read | OCL Region | 1 | 1 | 103830 | 103831 | 0.00333333 | 4002.138109 | 4002.138112 |
| 4002.138119 | Kernel\_Read\|xilinx:adm-pcie-7v3:1ddr:3.0-0\|cnn\_GOOD | Read | OCL Region | 1 | 1 | 103833 | 103834 | 0.00333333 | 4002.138119 | 4002.138122 |
| 4002.138136 | Kernel\_Read\|xilinx:adm-pcie-7v3:1ddr:3.0-0\|cnn\_GOOD | Read | OCL Region | 1 | 1 | 103838 | 103839 | 0.00333333 | 4002.138136 | 4002.138139 |
| 4002.138159 | Kernel\_Read\|xilinx:adm-pcie-7v3:1ddr:3.0-0\|cnn\_GOOD | Read | OCL Region | 1 | 1 | 103845 | 103846 | 0.00333333 | 4002.138159 | 4002.138162 |
| 4002.138169 | Kernel\_Read\|xilinx:adm-pcie-7v3:1ddr:3.0-0\|cnn\_GOOD | Read | OCL Region | 1 | 1 | 103848 | 103849 | 0.00333333 | 4002.138169 | 4002.138172 |
| 4002.138186 | Kernel\_Read\|xilinx:adm-pcie-7v3:1ddr:3.0-0\|cnn\_GOOD | Read | OCL Region | 1 | 1 | 103853 | 103854 | 0.00333333 | 4002.138186 | 4002.138189 |
| 4002.138209 | Kernel\_Read\|xilinx:adm-pcie-7v3:1ddr:3.0-0\|cnn\_GOOD | Read | OCL Region | 1 | 1 | 103860 | 103861 | 0.00333333 | 4002.138209 | 4002.138212 |
| 4002.138219 | Kernel\_Read\|xilinx:adm-pcie-7v3:1ddr:3.0-0\|cnn\_GOOD | Read | OCL Region | 1 | 1 | 103863 | 103864 | 0.00333333 | 4002.138219 | 4002.138222 |
| 4002.138236 | Kernel\_Read\|xilinx:adm-pcie-7v3:1ddr:3.0-0\|cnn\_GOOD | Read | OCL Region | 1 | 1 | 103868 | 103869 | 0.00333333 | 4002.138236 | 4002.138239 |
| 4002.138259 | Kernel\_Read\|xilinx:adm-pcie-7v3:1ddr:3.0-0\|cnn\_GOOD | Read | OCL Region | 1 | 1 | 103875 | 103876 | 0.00333333 | 4002.138259 | 4002.138262 |
| 4002.138269 | Kernel\_Read\|xilinx:adm-pcie-7v3:1ddr:3.0-0\|cnn\_GOOD | Read | OCL Region | 1 | 1 | 103878 | 103879 | 0.00333333 | 4002.138269 | 4002.138272 |
| 4002.138286 | Kernel\_Read\|xilinx:adm-pcie-7v3:1ddr:3.0-0\|cnn\_GOOD | Read | OCL Region | 1 | 1 | 103883 | 103884 | 0.00333333 | 4002.138286 | 4002.138289 |
| 4002.138652 | Kernel\_Read\|xilinx:adm-pcie-7v3:1ddr:3.0-0\|cnn\_GOOD | Read | OCL Region | 1 | 1 | 103993 | 103994 | 0.00333333 | 4002.138652 | 4002.138656 |
| 4002.138719 | Kernel\_Read\|xilinx:adm-pcie-7v3:1ddr:3.0-0\|cnn\_GOOD | Read | OCL Region | 1 | 1 | 104013 | 104014 | 0.00333333 | 4002.138719 | 4002.138722 |
| 4002.138786 | Kernel\_Read\|xilinx:adm-pcie-7v3:1ddr:3.0-0\|cnn\_GOOD | Read | OCL Region | 1 | 1 | 104033 | 104034 | 0.00333333 | 4002.138786 | 4002.138789 |
| 4002.138852 | Kernel\_Read\|xilinx:adm-pcie-7v3:1ddr:3.0-0\|cnn\_GOOD | Read | OCL Region | 1 | 1 | 104053 | 104054 | 0.00333333 | 4002.138852 | 4002.138856 |
| 4002.138919 | Kernel\_Read\|xilinx:adm-pcie-7v3:1ddr:3.0-0\|cnn\_GOOD | Read | OCL Region | 1 | 1 | 104073 | 104074 | 0.00333333 | 4002.138919 | 4002.138922 |
| 4002.138986 | Kernel\_Read\|xilinx:adm-pcie-7v3:1ddr:3.0-0\|cnn\_GOOD | Read | OCL Region | 1 | 1 | 104093 | 104094 | 0.00333333 | 4002.138986 | 4002.138989 |
| 4002.139052 | Kernel\_Read\|xilinx:adm-pcie-7v3:1ddr:3.0-0\|cnn\_GOOD | Read | OCL Region | 1 | 1 | 104113 | 104114 | 0.00333333 | 4002.139052 | 4002.139056 |
| 4002.139119 | Kernel\_Read\|xilinx:adm-pcie-7v3:1ddr:3.0-0\|cnn\_GOOD | Read | OCL Region | 1 | 1 | 104133 | 104134 | 0.00333333 | 4002.139119 | 4002.139122 |
| 4002.139186 | Kernel\_Read\|xilinx:adm-pcie-7v3:1ddr:3.0-0\|cnn\_GOOD | Read | OCL Region | 1 | 1 | 104153 | 104154 | 0.00333333 | 4002.139186 | 4002.139189 |
| 4002.139252 | Kernel\_Read\|xilinx:adm-pcie-7v3:1ddr:3.0-0\|cnn\_GOOD | Read | OCL Region | 1 | 1 | 104173 | 104174 | 0.00333333 | 4002.139252 | 4002.139256 |
| 4002.139319 | Kernel\_Read\|xilinx:adm-pcie-7v3:1ddr:3.0-0\|cnn\_GOOD | Read | OCL Region | 1 | 1 | 104193 | 104194 | 0.00333333 | 4002.139319 | 4002.139322 |
| 4002.139386 | Kernel\_Read\|xilinx:adm-pcie-7v3:1ddr:3.0-0\|cnn\_GOOD | Read | OCL Region | 1 | 1 | 104213 | 104214 | 0.00333333 | 4002.139386 | 4002.139389 |
| 4002.139452 | Kernel\_Read\|xilinx:adm-pcie-7v3:1ddr:3.0-0\|cnn\_GOOD | Read | OCL Region | 1 | 1 | 104233 | 104234 | 0.00333333 | 4002.139452 | 4002.139456 |
| 4002.139519 | Kernel\_Read\|xilinx:adm-pcie-7v3:1ddr:3.0-0\|cnn\_GOOD | Read | OCL Region | 1 | 1 | 104253 | 104254 | 0.00333333 | 4002.139519 | 4002.139522 |
| 4002.139586 | Kernel\_Read\|xilinx:adm-pcie-7v3:1ddr:3.0-0\|cnn\_GOOD | Read | OCL Region | 1 | 1 | 104273 | 104274 | 0.00333333 | 4002.139586 | 4002.139589 |
| 4002.139652 | Kernel\_Read\|xilinx:adm-pcie-7v3:1ddr:3.0-0\|cnn\_GOOD | Read | OCL Region | 1 | 1 | 104293 | 104294 | 0.00333333 | 4002.139652 | 4002.139656 |
| 4002.139719 | Kernel\_Read\|xilinx:adm-pcie-7v3:1ddr:3.0-0\|cnn\_GOOD | Read | OCL Region | 1 | 1 | 104313 | 104314 | 0.00333333 | 4002.139719 | 4002.139722 |
| 4002.139786 | Kernel\_Read\|xilinx:adm-pcie-7v3:1ddr:3.0-0\|cnn\_GOOD | Read | OCL Region | 1 | 1 | 104333 | 104334 | 0.00333333 | 4002.139786 | 4002.139789 |
| 4002.139852 | Kernel\_Read\|xilinx:adm-pcie-7v3:1ddr:3.0-0\|cnn\_GOOD | Read | OCL Region | 1 | 1 | 104353 | 104354 | 0.00333333 | 4002.139852 | 4002.139856 |
| 4002.139919 | Kernel\_Read\|xilinx:adm-pcie-7v3:1ddr:3.0-0\|cnn\_GOOD | Read | OCL Region | 1 | 1 | 104373 | 104374 | 0.00333333 | 4002.139919 | 4002.139922 |
| 4002.139986 | Kernel\_Read\|xilinx:adm-pcie-7v3:1ddr:3.0-0\|cnn\_GOOD | Read | OCL Region | 1 | 1 | 104393 | 104394 | 0.00333333 | 4002.139986 | 4002.139989 |
| 4002.140052 | Kernel\_Read\|xilinx:adm-pcie-7v3:1ddr:3.0-0\|cnn\_GOOD | Read | OCL Region | 1 | 1 | 104413 | 104414 | 0.00333333 | 4002.140052 | 4002.140056 |
| 4002.140119 | Kernel\_Read\|xilinx:adm-pcie-7v3:1ddr:3.0-0\|cnn\_GOOD | Read | OCL Region | 1 | 1 | 104433 | 104434 | 0.00333333 | 4002.140119 | 4002.140122 |
| 4002.140186 | Kernel\_Read\|xilinx:adm-pcie-7v3:1ddr:3.0-0\|cnn\_GOOD | Read | OCL Region | 1 | 1 | 104453 | 104454 | 0.00333333 | 4002.140186 | 4002.140189 |
| 4002.140252 | Kernel\_Read\|xilinx:adm-pcie-7v3:1ddr:3.0-0\|cnn\_GOOD | Read | OCL Region | 1 | 1 | 104473 | 104474 | 0.00333333 | 4002.140252 | 4002.140256 |
| 4002.140319 | Kernel\_Read\|xilinx:adm-pcie-7v3:1ddr:3.0-0\|cnn\_GOOD | Read | OCL Region | 1 | 1 | 104493 | 104494 | 0.00333333 | 4002.140319 | 4002.140322 |
| 4002.140386 | Kernel\_Read\|xilinx:adm-pcie-7v3:1ddr:3.0-0\|cnn\_GOOD | Read | OCL Region | 1 | 1 | 104513 | 104514 | 0.00333333 | 4002.140386 | 4002.140389 |
| 4002.140449 | Kernel\_Read\|xilinx:adm-pcie-7v3:1ddr:3.0-0\|cnn\_GOOD | Read | OCL Region | 1 | 1 | 104532 | 104533 | 0.00333333 | 4002.140449 | 4002.140452 |
| 4002.140529 | Kernel\_Read\|xilinx:adm-pcie-7v3:1ddr:3.0-0\|cnn\_GOOD | Read | OCL Region | 1 | 1 | 104556 | 104557 | 0.00333333 | 4002.140529 | 4002.140532 |
| 4002.140586 | Kernel\_Read\|xilinx:adm-pcie-7v3:1ddr:3.0-0\|cnn\_GOOD | Read | OCL Region | 1 | 1 | 104573 | 104574 | 0.00333333 | 4002.140586 | 4002.140589 |
| 4002.140652 | Kernel\_Read\|xilinx:adm-pcie-7v3:1ddr:3.0-0\|cnn\_GOOD | Read | OCL Region | 1 | 1 | 104593 | 104594 | 0.00333333 | 4002.140652 | 4002.140656 |
| 4002.140719 | Kernel\_Read\|xilinx:adm-pcie-7v3:1ddr:3.0-0\|cnn\_GOOD | Read | OCL Region | 1 | 1 | 104613 | 104614 | 0.00333333 | 4002.140719 | 4002.140722 |
| 4002.140786 | Kernel\_Read\|xilinx:adm-pcie-7v3:1ddr:3.0-0\|cnn\_GOOD | Read | OCL Region | 1 | 1 | 104633 | 104634 | 0.00333333 | 4002.140786 | 4002.140789 |
| 4002.140852 | Kernel\_Read\|xilinx:adm-pcie-7v3:1ddr:3.0-0\|cnn\_GOOD | Read | OCL Region | 1 | 1 | 104653 | 104654 | 0.00333333 | 4002.140852 | 4002.140856 |
| 4002.140919 | Kernel\_Read\|xilinx:adm-pcie-7v3:1ddr:3.0-0\|cnn\_GOOD | Read | OCL Region | 1 | 1 | 104673 | 104674 | 0.00333333 | 4002.140919 | 4002.140922 |
| 4002.140986 | Kernel\_Read\|xilinx:adm-pcie-7v3:1ddr:3.0-0\|cnn\_GOOD | Read | OCL Region | 1 | 1 | 104693 | 104694 | 0.00333333 | 4002.140986 | 4002.140989 |
| 4002.141052 | Kernel\_Read\|xilinx:adm-pcie-7v3:1ddr:3.0-0\|cnn\_GOOD | Read | OCL Region | 1 | 1 | 104713 | 104714 | 0.00333333 | 4002.141052 | 4002.141056 |
| 4002.141119 | Kernel\_Read\|xilinx:adm-pcie-7v3:1ddr:3.0-0\|cnn\_GOOD | Read | OCL Region | 1 | 1 | 104733 | 104734 | 0.00333333 | 4002.141119 | 4002.141122 |
| 4002.141186 | Kernel\_Read\|xilinx:adm-pcie-7v3:1ddr:3.0-0\|cnn\_GOOD | Read | OCL Region | 1 | 1 | 104753 | 104754 | 0.00333333 | 4002.141186 | 4002.141189 |
| 4002.141252 | Kernel\_Read\|xilinx:adm-pcie-7v3:1ddr:3.0-0\|cnn\_GOOD | Read | OCL Region | 1 | 1 | 104773 | 104774 | 0.00333333 | 4002.141252 | 4002.141256 |
| 4002.141316 | Kernel\_Read\|xilinx:adm-pcie-7v3:1ddr:3.0-0\|cnn\_GOOD | Read | OCL Region | 1 | 1 | 104792 | 104793 | 0.00333333 | 4002.141316 | 4002.141319 |
| 4002.141386 | Kernel\_Read\|xilinx:adm-pcie-7v3:1ddr:3.0-0\|cnn\_GOOD | Read | OCL Region | 1 | 1 | 104813 | 104814 | 0.00333333 | 4002.141386 | 4002.141389 |
| 4002.141452 | Kernel\_Read\|xilinx:adm-pcie-7v3:1ddr:3.0-0\|cnn\_GOOD | Read | OCL Region | 1 | 1 | 104833 | 104834 | 0.00333333 | 4002.141452 | 4002.141456 |
| 4002.141519 | Kernel\_Read\|xilinx:adm-pcie-7v3:1ddr:3.0-0\|cnn\_GOOD | Read | OCL Region | 1 | 1 | 104853 | 104854 | 0.00333333 | 4002.141519 | 4002.141522 |
| 4002.141586 | Kernel\_Read\|xilinx:adm-pcie-7v3:1ddr:3.0-0\|cnn\_GOOD | Read | OCL Region | 1 | 1 | 104873 | 104874 | 0.00333333 | 4002.141586 | 4002.141589 |
| 4002.141652 | Kernel\_Read\|xilinx:adm-pcie-7v3:1ddr:3.0-0\|cnn\_GOOD | Read | OCL Region | 1 | 1 | 104893 | 104894 | 0.00333333 | 4002.141652 | 4002.141656 |
| 4002.141719 | Kernel\_Read\|xilinx:adm-pcie-7v3:1ddr:3.0-0\|cnn\_GOOD | Read | OCL Region | 1 | 1 | 104913 | 104914 | 0.00333333 | 4002.141719 | 4002.141722 |
| 4002.141786 | Kernel\_Read\|xilinx:adm-pcie-7v3:1ddr:3.0-0\|cnn\_GOOD | Read | OCL Region | 1 | 1 | 104933 | 104934 | 0.00333333 | 4002.141786 | 4002.141789 |
| 4002.141852 | Kernel\_Read\|xilinx:adm-pcie-7v3:1ddr:3.0-0\|cnn\_GOOD | Read | OCL Region | 1 | 1 | 104953 | 104954 | 0.00333333 | 4002.141852 | 4002.141856 |
| 4002.141919 | Kernel\_Read\|xilinx:adm-pcie-7v3:1ddr:3.0-0\|cnn\_GOOD | Read | OCL Region | 1 | 1 | 104973 | 104974 | 0.00333333 | 4002.141919 | 4002.141922 |
| 4002.141986 | Kernel\_Read\|xilinx:adm-pcie-7v3:1ddr:3.0-0\|cnn\_GOOD | Read | OCL Region | 1 | 1 | 104993 | 104994 | 0.00333333 | 4002.141986 | 4002.141989 |
| 4002.142049 | Kernel\_Read\|xilinx:adm-pcie-7v3:1ddr:3.0-0\|cnn\_GOOD | Read | OCL Region | 1 | 1 | 105012 | 105013 | 0.00333333 | 4002.142049 | 4002.142052 |
| 4002.142119 | Kernel\_Read\|xilinx:adm-pcie-7v3:1ddr:3.0-0\|cnn\_GOOD | Read | OCL Region | 1 | 1 | 105033 | 105034 | 0.00333333 | 4002.142119 | 4002.142122 |
| 4002.142186 | Kernel\_Read\|xilinx:adm-pcie-7v3:1ddr:3.0-0\|cnn\_GOOD | Read | OCL Region | 1 | 1 | 105053 | 105054 | 0.00333333 | 4002.142186 | 4002.142189 |
| 4002.142252 | Kernel\_Read\|xilinx:adm-pcie-7v3:1ddr:3.0-0\|cnn\_GOOD | Read | OCL Region | 1 | 1 | 105073 | 105074 | 0.00333333 | 4002.142252 | 4002.142256 |
| 4002.142319 | Kernel\_Read\|xilinx:adm-pcie-7v3:1ddr:3.0-0\|cnn\_GOOD | Read | OCL Region | 1 | 1 | 105093 | 105094 | 0.00333333 | 4002.142319 | 4002.142322 |
| 4002.142386 | Kernel\_Read\|xilinx:adm-pcie-7v3:1ddr:3.0-0\|cnn\_GOOD | Read | OCL Region | 1 | 1 | 105113 | 105114 | 0.00333333 | 4002.142386 | 4002.142389 |
| 4002.142452 | Kernel\_Read\|xilinx:adm-pcie-7v3:1ddr:3.0-0\|cnn\_GOOD | Read | OCL Region | 1 | 1 | 105133 | 105134 | 0.00333333 | 4002.142452 | 4002.142456 |
| 4002.142519 | Kernel\_Read\|xilinx:adm-pcie-7v3:1ddr:3.0-0\|cnn\_GOOD | Read | OCL Region | 1 | 1 | 105153 | 105154 | 0.00333333 | 4002.142519 | 4002.142522 |
| 4002.142586 | Kernel\_Read\|xilinx:adm-pcie-7v3:1ddr:3.0-0\|cnn\_GOOD | Read | OCL Region | 1 | 1 | 105173 | 105174 | 0.00333333 | 4002.142586 | 4002.142589 |
| 4002.142652 | Kernel\_Read\|xilinx:adm-pcie-7v3:1ddr:3.0-0\|cnn\_GOOD | Read | OCL Region | 1 | 1 | 105193 | 105194 | 0.00333333 | 4002.142652 | 4002.142656 |
| 4002.142719 | Kernel\_Read\|xilinx:adm-pcie-7v3:1ddr:3.0-0\|cnn\_GOOD | Read | OCL Region | 1 | 1 | 105213 | 105214 | 0.00333333 | 4002.142719 | 4002.142722 |
| 4002.142786 | Kernel\_Read\|xilinx:adm-pcie-7v3:1ddr:3.0-0\|cnn\_GOOD | Read | OCL Region | 1 | 1 | 105233 | 105234 | 0.00333333 | 4002.142786 | 4002.142789 |
| 4002.142852 | Kernel\_Read\|xilinx:adm-pcie-7v3:1ddr:3.0-0\|cnn\_GOOD | Read | OCL Region | 1 | 1 | 105253 | 105254 | 0.00333333 | 4002.142852 | 4002.142856 |
| 4002.142919 | Kernel\_Read\|xilinx:adm-pcie-7v3:1ddr:3.0-0\|cnn\_GOOD | Read | OCL Region | 1 | 1 | 105273 | 105274 | 0.00333333 | 4002.142919 | 4002.142922 |
| 4002.143002 | Kernel\_Read\|xilinx:adm-pcie-7v3:1ddr:3.0-0\|cnn\_GOOD | Read | OCL Region | 1 | 1 | 105298 | 105299 | 0.00333333 | 4002.143002 | 4002.143006 |
| 4002.143059 | Kernel\_Read\|xilinx:adm-pcie-7v3:1ddr:3.0-0\|cnn\_GOOD | Read | OCL Region | 1 | 1 | 105315 | 105316 | 0.00333333 | 4002.143059 | 4002.143062 |
| 4002.143119 | Kernel\_Read\|xilinx:adm-pcie-7v3:1ddr:3.0-0\|cnn\_GOOD | Read | OCL Region | 1 | 1 | 105333 | 105334 | 0.00333333 | 4002.143119 | 4002.143122 |
| 4002.143186 | Kernel\_Read\|xilinx:adm-pcie-7v3:1ddr:3.0-0\|cnn\_GOOD | Read | OCL Region | 1 | 1 | 105353 | 105354 | 0.00333333 | 4002.143186 | 4002.143189 |
| 4002.143252 | Kernel\_Read\|xilinx:adm-pcie-7v3:1ddr:3.0-0\|cnn\_GOOD | Read | OCL Region | 1 | 1 | 105373 | 105374 | 0.00333333 | 4002.143252 | 4002.143256 |
| 4002.143319 | Kernel\_Read\|xilinx:adm-pcie-7v3:1ddr:3.0-0\|cnn\_GOOD | Read | OCL Region | 1 | 1 | 105393 | 105394 | 0.00333333 | 4002.143319 | 4002.143322 |
| 4002.143386 | Kernel\_Read\|xilinx:adm-pcie-7v3:1ddr:3.0-0\|cnn\_GOOD | Read | OCL Region | 1 | 1 | 105413 | 105414 | 0.00333333 | 4002.143386 | 4002.143389 |
| 4002.143449 | Kernel\_Read\|xilinx:adm-pcie-7v3:1ddr:3.0-0\|cnn\_GOOD | Read | OCL Region | 1 | 1 | 105432 | 105433 | 0.00333333 | 4002.143449 | 4002.143452 |
| 4002.143519 | Kernel\_Read\|xilinx:adm-pcie-7v3:1ddr:3.0-0\|cnn\_GOOD | Read | OCL Region | 1 | 1 | 105453 | 105454 | 0.00333333 | 4002.143519 | 4002.143522 |
| 4002.143586 | Kernel\_Read\|xilinx:adm-pcie-7v3:1ddr:3.0-0\|cnn\_GOOD | Read | OCL Region | 1 | 1 | 105473 | 105474 | 0.00333333 | 4002.143586 | 4002.143589 |
| 4002.143649 | Kernel\_Read\|xilinx:adm-pcie-7v3:1ddr:3.0-0\|cnn\_GOOD | Read | OCL Region | 1 | 1 | 105492 | 105493 | 0.00333333 | 4002.143649 | 4002.143652 |
| 4002.143719 | Kernel\_Read\|xilinx:adm-pcie-7v3:1ddr:3.0-0\|cnn\_GOOD | Read | OCL Region | 1 | 1 | 105513 | 105514 | 0.00333333 | 4002.143719 | 4002.143722 |
| 4002.144012 | Kernel\_Read\|xilinx:adm-pcie-7v3:1ddr:3.0-0\|cnn\_GOOD | Read | OCL Region | 1 | 1 | 105601 | 105602 | 0.00333333 | 4002.144012 | 4002.144016 |
| 4002.144016 | Kernel\_Read\|xilinx:adm-pcie-7v3:1ddr:3.0-0\|cnn\_GOOD | Read | OCL Region | 1 | 1 | 105602 | 105603 | 0.00333333 | 4002.144016 | 4002.144019 |
| 4002.144029 | Kernel\_Read\|xilinx:adm-pcie-7v3:1ddr:3.0-0\|cnn\_GOOD | Read | OCL Region | 1 | 1 | 105606 | 105607 | 0.00333333 | 4002.144029 | 4002.144032 |
| 4002.144032 | Kernel\_Read\|xilinx:adm-pcie-7v3:1ddr:3.0-0\|cnn\_GOOD | Read | OCL Region | 1 | 1 | 105607 | 105608 | 0.00333333 | 4002.144032 | 4002.144036 |
| 4002.144036 | Kernel\_Read\|xilinx:adm-pcie-7v3:1ddr:3.0-0\|cnn\_GOOD | Read | OCL Region | 1 | 1 | 105608 | 105609 | 0.00333333 | 4002.144036 | 4002.144039 |
| 4002.144119 | Kernel\_Read\|xilinx:adm-pcie-7v3:1ddr:3.0-0\|cnn\_GOOD | Read | OCL Region | 1 | 1 | 105633 | 105634 | 0.00333333 | 4002.144119 | 4002.144122 |
| 4002.144186 | Kernel\_Read\|xilinx:adm-pcie-7v3:1ddr:3.0-0\|cnn\_GOOD | Read | OCL Region | 1 | 1 | 105653 | 105654 | 0.00333333 | 4002.144186 | 4002.144189 |
| 4002.144249 | Kernel\_Read\|xilinx:adm-pcie-7v3:1ddr:3.0-0\|cnn\_GOOD | Read | OCL Region | 1 | 1 | 105672 | 105673 | 0.00333333 | 4002.144249 | 4002.144252 |
| 4002.144319 | Kernel\_Read\|xilinx:adm-pcie-7v3:1ddr:3.0-0\|cnn\_GOOD | Read | OCL Region | 1 | 1 | 105693 | 105694 | 0.00333333 | 4002.144319 | 4002.144322 |
| 4002.144386 | Kernel\_Read\|xilinx:adm-pcie-7v3:1ddr:3.0-0\|cnn\_GOOD | Read | OCL Region | 1 | 1 | 105713 | 105714 | 0.00333333 | 4002.144386 | 4002.144389 |
| 4002.144452 | Kernel\_Read\|xilinx:adm-pcie-7v3:1ddr:3.0-0\|cnn\_GOOD | Read | OCL Region | 1 | 1 | 105733 | 105734 | 0.00333333 | 4002.144452 | 4002.144456 |
| 4002.144519 | Kernel\_Read\|xilinx:adm-pcie-7v3:1ddr:3.0-0\|cnn\_GOOD | Read | OCL Region | 1 | 1 | 105753 | 105754 | 0.00333333 | 4002.144519 | 4002.144522 |
| 4002.144586 | Kernel\_Read\|xilinx:adm-pcie-7v3:1ddr:3.0-0\|cnn\_GOOD | Read | OCL Region | 1 | 1 | 105773 | 105774 | 0.00333333 | 4002.144586 | 4002.144589 |
| 4002.144652 | Kernel\_Read\|xilinx:adm-pcie-7v3:1ddr:3.0-0\|cnn\_GOOD | Read | OCL Region | 1 | 1 | 105793 | 105794 | 0.00333333 | 4002.144652 | 4002.144656 |
| 4002.144719 | Kernel\_Read\|xilinx:adm-pcie-7v3:1ddr:3.0-0\|cnn\_GOOD | Read | OCL Region | 1 | 1 | 105813 | 105814 | 0.00333333 | 4002.144719 | 4002.144722 |
| 4002.144786 | Kernel\_Read\|xilinx:adm-pcie-7v3:1ddr:3.0-0\|cnn\_GOOD | Read | OCL Region | 1 | 1 | 105833 | 105834 | 0.00333333 | 4002.144786 | 4002.144789 |
| 4002.144852 | Kernel\_Read\|xilinx:adm-pcie-7v3:1ddr:3.0-0\|cnn\_GOOD | Read | OCL Region | 1 | 1 | 105853 | 105854 | 0.00333333 | 4002.144852 | 4002.144856 |
| 4002.144919 | Kernel\_Read\|xilinx:adm-pcie-7v3:1ddr:3.0-0\|cnn\_GOOD | Read | OCL Region | 1 | 1 | 105873 | 105874 | 0.00333333 | 4002.144919 | 4002.144922 |
| 4002.144986 | Kernel\_Read\|xilinx:adm-pcie-7v3:1ddr:3.0-0\|cnn\_GOOD | Read | OCL Region | 1 | 1 | 105893 | 105894 | 0.00333333 | 4002.144986 | 4002.144989 |
| 4002.145049 | Kernel\_Read\|xilinx:adm-pcie-7v3:1ddr:3.0-0\|cnn\_GOOD | Read | OCL Region | 1 | 1 | 105912 | 105913 | 0.00333333 | 4002.145049 | 4002.145052 |
| 4002.145119 | Kernel\_Read\|xilinx:adm-pcie-7v3:1ddr:3.0-0\|cnn\_GOOD | Read | OCL Region | 1 | 1 | 105933 | 105934 | 0.00333333 | 4002.145119 | 4002.145122 |
| 4002.145186 | Kernel\_Read\|xilinx:adm-pcie-7v3:1ddr:3.0-0\|cnn\_GOOD | Read | OCL Region | 1 | 1 | 105953 | 105954 | 0.00333333 | 4002.145186 | 4002.145189 |
| 4002.145249 | Kernel\_Read\|xilinx:adm-pcie-7v3:1ddr:3.0-0\|cnn\_GOOD | Read | OCL Region | 1 | 1 | 105972 | 105973 | 0.00333333 | 4002.145249 | 4002.145252 |
| 4002.145319 | Kernel\_Read\|xilinx:adm-pcie-7v3:1ddr:3.0-0\|cnn\_GOOD | Read | OCL Region | 1 | 1 | 105993 | 105994 | 0.00333333 | 4002.145319 | 4002.145322 |
| 4002.145386 | Kernel\_Read\|xilinx:adm-pcie-7v3:1ddr:3.0-0\|cnn\_GOOD | Read | OCL Region | 1 | 1 | 106013 | 106014 | 0.00333333 | 4002.145386 | 4002.145389 |
| 4002.145449 | Kernel\_Read\|xilinx:adm-pcie-7v3:1ddr:3.0-0\|cnn\_GOOD | Read | OCL Region | 1 | 1 | 106032 | 106033 | 0.00333333 | 4002.145449 | 4002.145452 |
| 4002.145519 | Kernel\_Read\|xilinx:adm-pcie-7v3:1ddr:3.0-0\|cnn\_GOOD | Read | OCL Region | 1 | 1 | 106053 | 106054 | 0.00333333 | 4002.145519 | 4002.145522 |
| 4002.145586 | Kernel\_Read\|xilinx:adm-pcie-7v3:1ddr:3.0-0\|cnn\_GOOD | Read | OCL Region | 1 | 1 | 106073 | 106074 | 0.00333333 | 4002.145586 | 4002.145589 |
| 4002.145652 | Kernel\_Read\|xilinx:adm-pcie-7v3:1ddr:3.0-0\|cnn\_GOOD | Read | OCL Region | 1 | 1 | 106093 | 106094 | 0.00333333 | 4002.145652 | 4002.145656 |
| 4002.145719 | Kernel\_Read\|xilinx:adm-pcie-7v3:1ddr:3.0-0\|cnn\_GOOD | Read | OCL Region | 1 | 1 | 106113 | 106114 | 0.00333333 | 4002.145719 | 4002.145722 |
| 4002.145786 | Kernel\_Read\|xilinx:adm-pcie-7v3:1ddr:3.0-0\|cnn\_GOOD | Read | OCL Region | 1 | 1 | 106133 | 106134 | 0.00333333 | 4002.145786 | 4002.145789 |
| 4002.145849 | Kernel\_Read\|xilinx:adm-pcie-7v3:1ddr:3.0-0\|cnn\_GOOD | Read | OCL Region | 1 | 1 | 106152 | 106153 | 0.00333333 | 4002.145849 | 4002.145852 |
| 4002.145919 | Kernel\_Read\|xilinx:adm-pcie-7v3:1ddr:3.0-0\|cnn\_GOOD | Read | OCL Region | 1 | 1 | 106173 | 106174 | 0.00333333 | 4002.145919 | 4002.145922 |
| 4002.145986 | Kernel\_Read\|xilinx:adm-pcie-7v3:1ddr:3.0-0\|cnn\_GOOD | Read | OCL Region | 1 | 1 | 106193 | 106194 | 0.00333333 | 4002.145986 | 4002.145989 |
| 4002.146052 | Kernel\_Read\|xilinx:adm-pcie-7v3:1ddr:3.0-0\|cnn\_GOOD | Read | OCL Region | 1 | 1 | 106213 | 106214 | 0.00333333 | 4002.146052 | 4002.146056 |
| 4002.146119 | Kernel\_Read\|xilinx:adm-pcie-7v3:1ddr:3.0-0\|cnn\_GOOD | Read | OCL Region | 1 | 1 | 106233 | 106234 | 0.00333333 | 4002.146119 | 4002.146122 |
| 4002.146186 | Kernel\_Read\|xilinx:adm-pcie-7v3:1ddr:3.0-0\|cnn\_GOOD | Read | OCL Region | 1 | 1 | 106253 | 106254 | 0.00333333 | 4002.146186 | 4002.146189 |
| 4002.146249 | Kernel\_Read\|xilinx:adm-pcie-7v3:1ddr:3.0-0\|cnn\_GOOD | Read | OCL Region | 1 | 1 | 106272 | 106273 | 0.00333333 | 4002.146249 | 4002.146252 |
| 4002.146319 | Kernel\_Read\|xilinx:adm-pcie-7v3:1ddr:3.0-0\|cnn\_GOOD | Read | OCL Region | 1 | 1 | 106293 | 106294 | 0.00333333 | 4002.146319 | 4002.146322 |
| 4002.146386 | Kernel\_Read\|xilinx:adm-pcie-7v3:1ddr:3.0-0\|cnn\_GOOD | Read | OCL Region | 1 | 1 | 106313 | 106314 | 0.00333333 | 4002.146386 | 4002.146389 |
| 4002.146466 | Kernel\_Read\|xilinx:adm-pcie-7v3:1ddr:3.0-0\|cnn\_GOOD | Read | OCL Region | 1 | 1 | 106337 | 106338 | 0.00333333 | 4002.146466 | 4002.146469 |
| 4002.146519 | Kernel\_Read\|xilinx:adm-pcie-7v3:1ddr:3.0-0\|cnn\_GOOD | Read | OCL Region | 1 | 1 | 106353 | 106354 | 0.00333333 | 4002.146519 | 4002.146522 |
| 4002.146586 | Kernel\_Read\|xilinx:adm-pcie-7v3:1ddr:3.0-0\|cnn\_GOOD | Read | OCL Region | 1 | 1 | 106373 | 106374 | 0.00333333 | 4002.146586 | 4002.146589 |
| 4002.146649 | Kernel\_Read\|xilinx:adm-pcie-7v3:1ddr:3.0-0\|cnn\_GOOD | Read | OCL Region | 1 | 1 | 106392 | 106393 | 0.00333333 | 4002.146649 | 4002.146652 |
| 4002.146719 | Kernel\_Read\|xilinx:adm-pcie-7v3:1ddr:3.0-0\|cnn\_GOOD | Read | OCL Region | 1 | 1 | 106413 | 106414 | 0.00333333 | 4002.146719 | 4002.146722 |
| 4002.146786 | Kernel\_Read\|xilinx:adm-pcie-7v3:1ddr:3.0-0\|cnn\_GOOD | Read | OCL Region | 1 | 1 | 106433 | 106434 | 0.00333333 | 4002.146786 | 4002.146789 |
| 4002.146849 | Kernel\_Read\|xilinx:adm-pcie-7v3:1ddr:3.0-0\|cnn\_GOOD | Read | OCL Region | 1 | 1 | 106452 | 106453 | 0.00333333 | 4002.146849 | 4002.146852 |
| 4002.146919 | Kernel\_Read\|xilinx:adm-pcie-7v3:1ddr:3.0-0\|cnn\_GOOD | Read | OCL Region | 1 | 1 | 106473 | 106474 | 0.00333333 | 4002.146919 | 4002.146922 |
| 4002.146986 | Kernel\_Read\|xilinx:adm-pcie-7v3:1ddr:3.0-0\|cnn\_GOOD | Read | OCL Region | 1 | 1 | 106493 | 106494 | 0.00333333 | 4002.146986 | 4002.146989 |
| 4002.147049 | Kernel\_Read\|xilinx:adm-pcie-7v3:1ddr:3.0-0\|cnn\_GOOD | Read | OCL Region | 1 | 1 | 106512 | 106513 | 0.00333333 | 4002.147049 | 4002.147052 |
| 4002.147119 | Kernel\_Read\|xilinx:adm-pcie-7v3:1ddr:3.0-0\|cnn\_GOOD | Read | OCL Region | 1 | 1 | 106533 | 106534 | 0.00333333 | 4002.147119 | 4002.147122 |
| 4002.147186 | Kernel\_Read\|xilinx:adm-pcie-7v3:1ddr:3.0-0\|cnn\_GOOD | Read | OCL Region | 1 | 1 | 106553 | 106554 | 0.00333333 | 4002.147186 | 4002.147189 |
| 4002.147259 | Kernel\_Read\|xilinx:adm-pcie-7v3:1ddr:3.0-0\|cnn\_GOOD | Read | OCL Region | 1 | 1 | 106575 | 106576 | 0.00333333 | 4002.147259 | 4002.147262 |
| 4002.147319 | Kernel\_Read\|xilinx:adm-pcie-7v3:1ddr:3.0-0\|cnn\_GOOD | Read | OCL Region | 1 | 1 | 106593 | 106594 | 0.00333333 | 4002.147319 | 4002.147322 |
| 4002.147386 | Kernel\_Read\|xilinx:adm-pcie-7v3:1ddr:3.0-0\|cnn\_GOOD | Read | OCL Region | 1 | 1 | 106613 | 106614 | 0.00333333 | 4002.147386 | 4002.147389 |
| 4002.147449 | Kernel\_Read\|xilinx:adm-pcie-7v3:1ddr:3.0-0\|cnn\_GOOD | Read | OCL Region | 1 | 1 | 106632 | 106633 | 0.00333333 | 4002.147449 | 4002.147452 |
| 4002.147519 | Kernel\_Read\|xilinx:adm-pcie-7v3:1ddr:3.0-0\|cnn\_GOOD | Read | OCL Region | 1 | 1 | 106653 | 106654 | 0.00333333 | 4002.147519 | 4002.147522 |
| 4002.147586 | Kernel\_Read\|xilinx:adm-pcie-7v3:1ddr:3.0-0\|cnn\_GOOD | Read | OCL Region | 1 | 1 | 106673 | 106674 | 0.00333333 | 4002.147586 | 4002.147589 |
| 4002.147649 | Kernel\_Read\|xilinx:adm-pcie-7v3:1ddr:3.0-0\|cnn\_GOOD | Read | OCL Region | 1 | 1 | 106692 | 106693 | 0.00333333 | 4002.147649 | 4002.147652 |
| 4002.147719 | Kernel\_Read\|xilinx:adm-pcie-7v3:1ddr:3.0-0\|cnn\_GOOD | Read | OCL Region | 1 | 1 | 106713 | 106714 | 0.00333333 | 4002.147719 | 4002.147722 |
| 4002.147786 | Kernel\_Read\|xilinx:adm-pcie-7v3:1ddr:3.0-0\|cnn\_GOOD | Read | OCL Region | 1 | 1 | 106733 | 106734 | 0.00333333 | 4002.147786 | 4002.147789 |
| 4002.147849 | Kernel\_Read\|xilinx:adm-pcie-7v3:1ddr:3.0-0\|cnn\_GOOD | Read | OCL Region | 1 | 1 | 106752 | 106753 | 0.00333333 | 4002.147849 | 4002.147852 |
| 4002.147919 | Kernel\_Read\|xilinx:adm-pcie-7v3:1ddr:3.0-0\|cnn\_GOOD | Read | OCL Region | 1 | 1 | 106773 | 106774 | 0.00333333 | 4002.147919 | 4002.147922 |
| 4002.147986 | Kernel\_Read\|xilinx:adm-pcie-7v3:1ddr:3.0-0\|cnn\_GOOD | Read | OCL Region | 1 | 1 | 106793 | 106794 | 0.00333333 | 4002.147986 | 4002.147989 |
| 4002.148052 | Kernel\_Read\|xilinx:adm-pcie-7v3:1ddr:3.0-0\|cnn\_GOOD | Read | OCL Region | 1 | 1 | 106813 | 106814 | 0.00333333 | 4002.148052 | 4002.148056 |
| 4002.148119 | Kernel\_Read\|xilinx:adm-pcie-7v3:1ddr:3.0-0\|cnn\_GOOD | Read | OCL Region | 1 | 1 | 106833 | 106834 | 0.00333333 | 4002.148119 | 4002.148122 |
| 4002.148186 | Kernel\_Read\|xilinx:adm-pcie-7v3:1ddr:3.0-0\|cnn\_GOOD | Read | OCL Region | 1 | 1 | 106853 | 106854 | 0.00333333 | 4002.148186 | 4002.148189 |
| 4002.148249 | Kernel\_Read\|xilinx:adm-pcie-7v3:1ddr:3.0-0\|cnn\_GOOD | Read | OCL Region | 1 | 1 | 106872 | 106873 | 0.00333333 | 4002.148249 | 4002.148252 |
| 4002.148319 | Kernel\_Read\|xilinx:adm-pcie-7v3:1ddr:3.0-0\|cnn\_GOOD | Read | OCL Region | 1 | 1 | 106893 | 106894 | 0.00333333 | 4002.148319 | 4002.148322 |
| 4002.148386 | Kernel\_Read\|xilinx:adm-pcie-7v3:1ddr:3.0-0\|cnn\_GOOD | Read | OCL Region | 1 | 1 | 106913 | 106914 | 0.00333333 | 4002.148386 | 4002.148389 |
| 4002.148449 | Kernel\_Read\|xilinx:adm-pcie-7v3:1ddr:3.0-0\|cnn\_GOOD | Read | OCL Region | 1 | 1 | 106932 | 106933 | 0.00333333 | 4002.148449 | 4002.148452 |
| 4002.148519 | Kernel\_Read\|xilinx:adm-pcie-7v3:1ddr:3.0-0\|cnn\_GOOD | Read | OCL Region | 1 | 1 | 106953 | 106954 | 0.00333333 | 4002.148519 | 4002.148522 |
| 4002.148586 | Kernel\_Read\|xilinx:adm-pcie-7v3:1ddr:3.0-0\|cnn\_GOOD | Read | OCL Region | 1 | 1 | 106973 | 106974 | 0.00333333 | 4002.148586 | 4002.148589 |
| 4002.148649 | Kernel\_Read\|xilinx:adm-pcie-7v3:1ddr:3.0-0\|cnn\_GOOD | Read | OCL Region | 1 | 1 | 106992 | 106993 | 0.00333333 | 4002.148649 | 4002.148652 |
| 4002.148719 | Kernel\_Read\|xilinx:adm-pcie-7v3:1ddr:3.0-0\|cnn\_GOOD | Read | OCL Region | 1 | 1 | 107013 | 107014 | 0.00333333 | 4002.148719 | 4002.148722 |
| 4002.148786 | Kernel\_Read\|xilinx:adm-pcie-7v3:1ddr:3.0-0\|cnn\_GOOD | Read | OCL Region | 1 | 1 | 107033 | 107034 | 0.00333333 | 4002.148786 | 4002.148789 |
| 4002.148852 | Kernel\_Read\|xilinx:adm-pcie-7v3:1ddr:3.0-0\|cnn\_GOOD | Read | OCL Region | 1 | 1 | 107053 | 107054 | 0.00333333 | 4002.148852 | 4002.148856 |
| 4002.148916 | Kernel\_Read\|xilinx:adm-pcie-7v3:1ddr:3.0-0\|cnn\_GOOD | Read | OCL Region | 1 | 1 | 107072 | 107073 | 0.00333333 | 4002.148916 | 4002.148919 |
| 4002.148986 | Kernel\_Read\|xilinx:adm-pcie-7v3:1ddr:3.0-0\|cnn\_GOOD | Read | OCL Region | 1 | 1 | 107093 | 107094 | 0.00333333 | 4002.148986 | 4002.148989 |
| 4002.149049 | Kernel\_Read\|xilinx:adm-pcie-7v3:1ddr:3.0-0\|cnn\_GOOD | Read | OCL Region | 1 | 1 | 107112 | 107113 | 0.00333333 | 4002.149049 | 4002.149052 |
| 4002.149119 | Kernel\_Read\|xilinx:adm-pcie-7v3:1ddr:3.0-0\|cnn\_GOOD | Read | OCL Region | 1 | 1 | 107133 | 107134 | 0.00333333 | 4002.149119 | 4002.149122 |
| 4002.149186 | Kernel\_Read\|xilinx:adm-pcie-7v3:1ddr:3.0-0\|cnn\_GOOD | Read | OCL Region | 1 | 1 | 107153 | 107154 | 0.00333333 | 4002.149186 | 4002.149189 |
| 4002.149252 | Kernel\_Read\|xilinx:adm-pcie-7v3:1ddr:3.0-0\|cnn\_GOOD | Read | OCL Region | 1 | 1 | 107173 | 107174 | 0.00333333 | 4002.149252 | 4002.149256 |
| 4002.149319 | Kernel\_Read\|xilinx:adm-pcie-7v3:1ddr:3.0-0\|cnn\_GOOD | Read | OCL Region | 1 | 1 | 107193 | 107194 | 0.00333333 | 4002.149319 | 4002.149322 |
| 4002.149386 | Kernel\_Read\|xilinx:adm-pcie-7v3:1ddr:3.0-0\|cnn\_GOOD | Read | OCL Region | 1 | 1 | 107213 | 107214 | 0.00333333 | 4002.149386 | 4002.149389 |
| 4002.149449 | Kernel\_Read\|xilinx:adm-pcie-7v3:1ddr:3.0-0\|cnn\_GOOD | Read | OCL Region | 1 | 1 | 107232 | 107233 | 0.00333333 | 4002.149449 | 4002.149452 |
| 4002.149519 | Kernel\_Read\|xilinx:adm-pcie-7v3:1ddr:3.0-0\|cnn\_GOOD | Read | OCL Region | 1 | 1 | 107253 | 107254 | 0.00333333 | 4002.149519 | 4002.149522 |
| 4002.149586 | Kernel\_Read\|xilinx:adm-pcie-7v3:1ddr:3.0-0\|cnn\_GOOD | Read | OCL Region | 1 | 1 | 107273 | 107274 | 0.00333333 | 4002.149586 | 4002.149589 |
| 4002.149652 | Kernel\_Read\|xilinx:adm-pcie-7v3:1ddr:3.0-0\|cnn\_GOOD | Read | OCL Region | 1 | 1 | 107293 | 107294 | 0.00333333 | 4002.149652 | 4002.149656 |
| 4002.149732 | Kernel\_Read\|xilinx:adm-pcie-7v3:1ddr:3.0-0\|cnn\_GOOD | Read | OCL Region | 1 | 1 | 107317 | 107318 | 0.00333333 | 4002.149732 | 4002.149736 |
| 4002.149786 | Kernel\_Read\|xilinx:adm-pcie-7v3:1ddr:3.0-0\|cnn\_GOOD | Read | OCL Region | 1 | 1 | 107333 | 107334 | 0.00333333 | 4002.149786 | 4002.149789 |
| 4002.149849 | Kernel\_Read\|xilinx:adm-pcie-7v3:1ddr:3.0-0\|cnn\_GOOD | Read | OCL Region | 1 | 1 | 107352 | 107353 | 0.00333333 | 4002.149849 | 4002.149852 |
| 4002.149919 | Kernel\_Read\|xilinx:adm-pcie-7v3:1ddr:3.0-0\|cnn\_GOOD | Read | OCL Region | 1 | 1 | 107373 | 107374 | 0.00333333 | 4002.149919 | 4002.149922 |
| 4002.149986 | Kernel\_Read\|xilinx:adm-pcie-7v3:1ddr:3.0-0\|cnn\_GOOD | Read | OCL Region | 1 | 1 | 107393 | 107394 | 0.00333333 | 4002.149986 | 4002.149989 |
| 4002.150269 | Kernel\_Read\|xilinx:adm-pcie-7v3:1ddr:3.0-0\|cnn\_GOOD | Read | OCL Region | 1 | 1 | 107478 | 107479 | 0.00333333 | 4002.150269 | 4002.150272 |
| 4002.150276 | Kernel\_Read\|xilinx:adm-pcie-7v3:1ddr:3.0-0\|cnn\_GOOD | Read | OCL Region | 1 | 1 | 107480 | 107481 | 0.00333333 | 4002.150276 | 4002.150279 |
| 4002.150282 | Kernel\_Read\|xilinx:adm-pcie-7v3:1ddr:3.0-0\|cnn\_GOOD | Read | OCL Region | 1 | 1 | 107482 | 107483 | 0.00333333 | 4002.150282 | 4002.150286 |
| 4002.150286 | Kernel\_Read\|xilinx:adm-pcie-7v3:1ddr:3.0-0\|cnn\_GOOD | Read | OCL Region | 1 | 1 | 107483 | 107484 | 0.00333333 | 4002.150286 | 4002.150289 |
| 4002.150309 | Kernel\_Read\|xilinx:adm-pcie-7v3:1ddr:3.0-0\|cnn\_GOOD | Read | OCL Region | 1 | 1 | 107490 | 107491 | 0.00333333 | 4002.150309 | 4002.150312 |
| 4002.150386 | Kernel\_Read\|xilinx:adm-pcie-7v3:1ddr:3.0-0\|cnn\_GOOD | Read | OCL Region | 1 | 1 | 107513 | 107514 | 0.00333333 | 4002.150386 | 4002.150389 |
| 4002.150452 | Kernel\_Read\|xilinx:adm-pcie-7v3:1ddr:3.0-0\|cnn\_GOOD | Read | OCL Region | 1 | 1 | 107533 | 107534 | 0.00333333 | 4002.150452 | 4002.150456 |
| 4002.150519 | Kernel\_Read\|xilinx:adm-pcie-7v3:1ddr:3.0-0\|cnn\_GOOD | Read | OCL Region | 1 | 1 | 107553 | 107554 | 0.00333333 | 4002.150519 | 4002.150522 |
| 4002.150586 | Kernel\_Read\|xilinx:adm-pcie-7v3:1ddr:3.0-0\|cnn\_GOOD | Read | OCL Region | 1 | 1 | 107573 | 107574 | 0.00333333 | 4002.150586 | 4002.150589 |
| 4002.150649 | Kernel\_Read\|xilinx:adm-pcie-7v3:1ddr:3.0-0\|cnn\_GOOD | Read | OCL Region | 1 | 1 | 107592 | 107593 | 0.00333333 | 4002.150649 | 4002.150652 |
| 4002.150719 | Kernel\_Read\|xilinx:adm-pcie-7v3:1ddr:3.0-0\|cnn\_GOOD | Read | OCL Region | 1 | 1 | 107613 | 107614 | 0.00333333 | 4002.150719 | 4002.150722 |
| 4002.150786 | Kernel\_Read\|xilinx:adm-pcie-7v3:1ddr:3.0-0\|cnn\_GOOD | Read | OCL Region | 1 | 1 | 107633 | 107634 | 0.00333333 | 4002.150786 | 4002.150789 |
| 4002.150849 | Kernel\_Read\|xilinx:adm-pcie-7v3:1ddr:3.0-0\|cnn\_GOOD | Read | OCL Region | 1 | 1 | 107652 | 107653 | 0.00333333 | 4002.150849 | 4002.150852 |
| 4002.150919 | Kernel\_Read\|xilinx:adm-pcie-7v3:1ddr:3.0-0\|cnn\_GOOD | Read | OCL Region | 1 | 1 | 107673 | 107674 | 0.00333333 | 4002.150919 | 4002.150922 |
| 4002.150986 | Kernel\_Read\|xilinx:adm-pcie-7v3:1ddr:3.0-0\|cnn\_GOOD | Read | OCL Region | 1 | 1 | 107693 | 107694 | 0.00333333 | 4002.150986 | 4002.150989 |
| 4002.151049 | Kernel\_Read\|xilinx:adm-pcie-7v3:1ddr:3.0-0\|cnn\_GOOD | Read | OCL Region | 1 | 1 | 107712 | 107713 | 0.00333333 | 4002.151049 | 4002.151052 |
| 4002.151119 | Kernel\_Read\|xilinx:adm-pcie-7v3:1ddr:3.0-0\|cnn\_GOOD | Read | OCL Region | 1 | 1 | 107733 | 107734 | 0.00333333 | 4002.151119 | 4002.151122 |
| 4002.151186 | Kernel\_Read\|xilinx:adm-pcie-7v3:1ddr:3.0-0\|cnn\_GOOD | Read | OCL Region | 1 | 1 | 107753 | 107754 | 0.00333333 | 4002.151186 | 4002.151189 |
| 4002.151252 | Kernel\_Read\|xilinx:adm-pcie-7v3:1ddr:3.0-0\|cnn\_GOOD | Read | OCL Region | 1 | 1 | 107773 | 107774 | 0.00333333 | 4002.151252 | 4002.151256 |
| 4002.151319 | Kernel\_Read\|xilinx:adm-pcie-7v3:1ddr:3.0-0\|cnn\_GOOD | Read | OCL Region | 1 | 1 | 107793 | 107794 | 0.00333333 | 4002.151319 | 4002.151322 |
| 4002.151386 | Kernel\_Read\|xilinx:adm-pcie-7v3:1ddr:3.0-0\|cnn\_GOOD | Read | OCL Region | 1 | 1 | 107813 | 107814 | 0.00333333 | 4002.151386 | 4002.151389 |
| 4002.151449 | Kernel\_Read\|xilinx:adm-pcie-7v3:1ddr:3.0-0\|cnn\_GOOD | Read | OCL Region | 1 | 1 | 107832 | 107833 | 0.00333333 | 4002.151449 | 4002.151452 |
| 4002.151519 | Kernel\_Read\|xilinx:adm-pcie-7v3:1ddr:3.0-0\|cnn\_GOOD | Read | OCL Region | 1 | 1 | 107853 | 107854 | 0.00333333 | 4002.151519 | 4002.151522 |
| 4002.151586 | Kernel\_Read\|xilinx:adm-pcie-7v3:1ddr:3.0-0\|cnn\_GOOD | Read | OCL Region | 1 | 1 | 107873 | 107874 | 0.00333333 | 4002.151586 | 4002.151589 |
| 4002.151649 | Kernel\_Read\|xilinx:adm-pcie-7v3:1ddr:3.0-0\|cnn\_GOOD | Read | OCL Region | 1 | 1 | 107892 | 107893 | 0.00333333 | 4002.151649 | 4002.151652 |
| 4002.151719 | Kernel\_Read\|xilinx:adm-pcie-7v3:1ddr:3.0-0\|cnn\_GOOD | Read | OCL Region | 1 | 1 | 107913 | 107914 | 0.00333333 | 4002.151719 | 4002.151722 |
| 4002.151786 | Kernel\_Read\|xilinx:adm-pcie-7v3:1ddr:3.0-0\|cnn\_GOOD | Read | OCL Region | 1 | 1 | 107933 | 107934 | 0.00333333 | 4002.151786 | 4002.151789 |
| 4002.151849 | Kernel\_Read\|xilinx:adm-pcie-7v3:1ddr:3.0-0\|cnn\_GOOD | Read | OCL Region | 1 | 1 | 107952 | 107953 | 0.00333333 | 4002.151849 | 4002.151852 |
| 4002.151919 | Kernel\_Read\|xilinx:adm-pcie-7v3:1ddr:3.0-0\|cnn\_GOOD | Read | OCL Region | 1 | 1 | 107973 | 107974 | 0.00333333 | 4002.151919 | 4002.151922 |
| 4002.151986 | Kernel\_Read\|xilinx:adm-pcie-7v3:1ddr:3.0-0\|cnn\_GOOD | Read | OCL Region | 1 | 1 | 107993 | 107994 | 0.00333333 | 4002.151986 | 4002.151989 |
| 4002.152052 | Kernel\_Read\|xilinx:adm-pcie-7v3:1ddr:3.0-0\|cnn\_GOOD | Read | OCL Region | 1 | 1 | 108013 | 108014 | 0.00333333 | 4002.152052 | 4002.152056 |
| 4002.152119 | Kernel\_Read\|xilinx:adm-pcie-7v3:1ddr:3.0-0\|cnn\_GOOD | Read | OCL Region | 1 | 1 | 108033 | 108034 | 0.00333333 | 4002.152119 | 4002.152122 |
| 4002.152186 | Kernel\_Read\|xilinx:adm-pcie-7v3:1ddr:3.0-0\|cnn\_GOOD | Read | OCL Region | 1 | 1 | 108053 | 108054 | 0.00333333 | 4002.152186 | 4002.152189 |
| 4002.152249 | Kernel\_Read\|xilinx:adm-pcie-7v3:1ddr:3.0-0\|cnn\_GOOD | Read | OCL Region | 1 | 1 | 108072 | 108073 | 0.00333333 | 4002.152249 | 4002.152252 |
| 4002.152319 | Kernel\_Read\|xilinx:adm-pcie-7v3:1ddr:3.0-0\|cnn\_GOOD | Read | OCL Region | 1 | 1 | 108093 | 108094 | 0.00333333 | 4002.152319 | 4002.152322 |
| 4002.152386 | Kernel\_Read\|xilinx:adm-pcie-7v3:1ddr:3.0-0\|cnn\_GOOD | Read | OCL Region | 1 | 1 | 108113 | 108114 | 0.00333333 | 4002.152386 | 4002.152389 |
| 4002.152452 | Kernel\_Read\|xilinx:adm-pcie-7v3:1ddr:3.0-0\|cnn\_GOOD | Read | OCL Region | 1 | 1 | 108133 | 108134 | 0.00333333 | 4002.152452 | 4002.152456 |
| 4002.152519 | Kernel\_Read\|xilinx:adm-pcie-7v3:1ddr:3.0-0\|cnn\_GOOD | Read | OCL Region | 1 | 1 | 108153 | 108154 | 0.00333333 | 4002.152519 | 4002.152522 |
| 4002.152586 | Kernel\_Read\|xilinx:adm-pcie-7v3:1ddr:3.0-0\|cnn\_GOOD | Read | OCL Region | 1 | 1 | 108173 | 108174 | 0.00333333 | 4002.152586 | 4002.152589 |
| 4002.152649 | Kernel\_Read\|xilinx:adm-pcie-7v3:1ddr:3.0-0\|cnn\_GOOD | Read | OCL Region | 1 | 1 | 108192 | 108193 | 0.00333333 | 4002.152649 | 4002.152652 |
| 4002.152716 | Kernel\_Read\|xilinx:adm-pcie-7v3:1ddr:3.0-0\|cnn\_GOOD | Read | OCL Region | 1 | 1 | 108212 | 108213 | 0.00333333 | 4002.152716 | 4002.152719 |
| 4002.152786 | Kernel\_Read\|xilinx:adm-pcie-7v3:1ddr:3.0-0\|cnn\_GOOD | Read | OCL Region | 1 | 1 | 108233 | 108234 | 0.00333333 | 4002.152786 | 4002.152789 |
| 4002.152852 | Kernel\_Read\|xilinx:adm-pcie-7v3:1ddr:3.0-0\|cnn\_GOOD | Read | OCL Region | 1 | 1 | 108253 | 108254 | 0.00333333 | 4002.152852 | 4002.152856 |
| 4002.152919 | Kernel\_Read\|xilinx:adm-pcie-7v3:1ddr:3.0-0\|cnn\_GOOD | Read | OCL Region | 1 | 1 | 108273 | 108274 | 0.00333333 | 4002.152919 | 4002.152922 |
| 4002.152986 | Kernel\_Read\|xilinx:adm-pcie-7v3:1ddr:3.0-0\|cnn\_GOOD | Read | OCL Region | 1 | 1 | 108293 | 108294 | 0.00333333 | 4002.152986 | 4002.152989 |
| 4002.153049 | Kernel\_Read\|xilinx:adm-pcie-7v3:1ddr:3.0-0\|cnn\_GOOD | Read | OCL Region | 1 | 1 | 108312 | 108313 | 0.00333333 | 4002.153049 | 4002.153052 |
| 4002.153119 | Kernel\_Read\|xilinx:adm-pcie-7v3:1ddr:3.0-0\|cnn\_GOOD | Read | OCL Region | 1 | 1 | 108333 | 108334 | 0.00333333 | 4002.153119 | 4002.153122 |
| 4002.153186 | Kernel\_Read\|xilinx:adm-pcie-7v3:1ddr:3.0-0\|cnn\_GOOD | Read | OCL Region | 1 | 1 | 108353 | 108354 | 0.00333333 | 4002.153186 | 4002.153189 |
| 4002.153249 | Kernel\_Read\|xilinx:adm-pcie-7v3:1ddr:3.0-0\|cnn\_GOOD | Read | OCL Region | 1 | 1 | 108372 | 108373 | 0.00333333 | 4002.153249 | 4002.153252 |
| 4002.153319 | Kernel\_Read\|xilinx:adm-pcie-7v3:1ddr:3.0-0\|cnn\_GOOD | Read | OCL Region | 1 | 1 | 108393 | 108394 | 0.00333333 | 4002.153319 | 4002.153322 |
| 4002.153386 | Kernel\_Read\|xilinx:adm-pcie-7v3:1ddr:3.0-0\|cnn\_GOOD | Read | OCL Region | 1 | 1 | 108413 | 108414 | 0.00333333 | 4002.153386 | 4002.153389 |
| 4002.153449 | Kernel\_Read\|xilinx:adm-pcie-7v3:1ddr:3.0-0\|cnn\_GOOD | Read | OCL Region | 1 | 1 | 108432 | 108433 | 0.00333333 | 4002.153449 | 4002.153452 |
| 4002.153529 | Kernel\_Read\|xilinx:adm-pcie-7v3:1ddr:3.0-0\|cnn\_GOOD | Read | OCL Region | 1 | 1 | 108456 | 108457 | 0.00333333 | 4002.153529 | 4002.153532 |
| 4002.153586 | Kernel\_Read\|xilinx:adm-pcie-7v3:1ddr:3.0-0\|cnn\_GOOD | Read | OCL Region | 1 | 1 | 108473 | 108474 | 0.00333333 | 4002.153586 | 4002.153589 |
| 4002.153652 | Kernel\_Read\|xilinx:adm-pcie-7v3:1ddr:3.0-0\|cnn\_GOOD | Read | OCL Region | 1 | 1 | 108493 | 108494 | 0.00333333 | 4002.153652 | 4002.153656 |
| 4002.153719 | Kernel\_Read\|xilinx:adm-pcie-7v3:1ddr:3.0-0\|cnn\_GOOD | Read | OCL Region | 1 | 1 | 108513 | 108514 | 0.00333333 | 4002.153719 | 4002.153722 |
| 4002.153786 | Kernel\_Read\|xilinx:adm-pcie-7v3:1ddr:3.0-0\|cnn\_GOOD | Read | OCL Region | 1 | 1 | 108533 | 108534 | 0.00333333 | 4002.153786 | 4002.153789 |
| 4002.153849 | Kernel\_Read\|xilinx:adm-pcie-7v3:1ddr:3.0-0\|cnn\_GOOD | Read | OCL Region | 1 | 1 | 108552 | 108553 | 0.00333333 | 4002.153849 | 4002.153852 |
| 4002.153919 | Kernel\_Read\|xilinx:adm-pcie-7v3:1ddr:3.0-0\|cnn\_GOOD | Read | OCL Region | 1 | 1 | 108573 | 108574 | 0.00333333 | 4002.153919 | 4002.153922 |
| 4002.153986 | Kernel\_Read\|xilinx:adm-pcie-7v3:1ddr:3.0-0\|cnn\_GOOD | Read | OCL Region | 1 | 1 | 108593 | 108594 | 0.00333333 | 4002.153986 | 4002.153989 |
| 4002.154049 | Kernel\_Read\|xilinx:adm-pcie-7v3:1ddr:3.0-0\|cnn\_GOOD | Read | OCL Region | 1 | 1 | 108612 | 108613 | 0.00333333 | 4002.154049 | 4002.154052 |
| 4002.154119 | Kernel\_Read\|xilinx:adm-pcie-7v3:1ddr:3.0-0\|cnn\_GOOD | Read | OCL Region | 1 | 1 | 108633 | 108634 | 0.00333333 | 4002.154119 | 4002.154122 |
| 4002.154186 | Kernel\_Read\|xilinx:adm-pcie-7v3:1ddr:3.0-0\|cnn\_GOOD | Read | OCL Region | 1 | 1 | 108653 | 108654 | 0.00333333 | 4002.154186 | 4002.154189 |
| 4002.154249 | Kernel\_Read\|xilinx:adm-pcie-7v3:1ddr:3.0-0\|cnn\_GOOD | Read | OCL Region | 1 | 1 | 108672 | 108673 | 0.00333333 | 4002.154249 | 4002.154252 |
| 4002.154319 | Kernel\_Read\|xilinx:adm-pcie-7v3:1ddr:3.0-0\|cnn\_GOOD | Read | OCL Region | 1 | 1 | 108693 | 108694 | 0.00333333 | 4002.154319 | 4002.154322 |
| 4002.154386 | Kernel\_Read\|xilinx:adm-pcie-7v3:1ddr:3.0-0\|cnn\_GOOD | Read | OCL Region | 1 | 1 | 108713 | 108714 | 0.00333333 | 4002.154386 | 4002.154389 |
| 4002.154449 | Kernel\_Read\|xilinx:adm-pcie-7v3:1ddr:3.0-0\|cnn\_GOOD | Read | OCL Region | 1 | 1 | 108732 | 108733 | 0.00333333 | 4002.154449 | 4002.154452 |
| 4002.154519 | Kernel\_Read\|xilinx:adm-pcie-7v3:1ddr:3.0-0\|cnn\_GOOD | Read | OCL Region | 1 | 1 | 108753 | 108754 | 0.00333333 | 4002.154519 | 4002.154522 |
| 4002.154586 | Kernel\_Read\|xilinx:adm-pcie-7v3:1ddr:3.0-0\|cnn\_GOOD | Read | OCL Region | 1 | 1 | 108773 | 108774 | 0.00333333 | 4002.154586 | 4002.154589 |
| 4002.154649 | Kernel\_Read\|xilinx:adm-pcie-7v3:1ddr:3.0-0\|cnn\_GOOD | Read | OCL Region | 1 | 1 | 108792 | 108793 | 0.00333333 | 4002.154649 | 4002.154652 |
| 4002.154719 | Kernel\_Read\|xilinx:adm-pcie-7v3:1ddr:3.0-0\|cnn\_GOOD | Read | OCL Region | 1 | 1 | 108813 | 108814 | 0.00333333 | 4002.154719 | 4002.154722 |
| 4002.154786 | Kernel\_Read\|xilinx:adm-pcie-7v3:1ddr:3.0-0\|cnn\_GOOD | Read | OCL Region | 1 | 1 | 108833 | 108834 | 0.00333333 | 4002.154786 | 4002.154789 |
| 4002.154849 | Kernel\_Read\|xilinx:adm-pcie-7v3:1ddr:3.0-0\|cnn\_GOOD | Read | OCL Region | 1 | 1 | 108852 | 108853 | 0.00333333 | 4002.154849 | 4002.154852 |
| 4002.154919 | Kernel\_Read\|xilinx:adm-pcie-7v3:1ddr:3.0-0\|cnn\_GOOD | Read | OCL Region | 1 | 1 | 108873 | 108874 | 0.00333333 | 4002.154919 | 4002.154922 |
| 4002.154986 | Kernel\_Read\|xilinx:adm-pcie-7v3:1ddr:3.0-0\|cnn\_GOOD | Read | OCL Region | 1 | 1 | 108893 | 108894 | 0.00333333 | 4002.154986 | 4002.154989 |
| 4002.155049 | Kernel\_Read\|xilinx:adm-pcie-7v3:1ddr:3.0-0\|cnn\_GOOD | Read | OCL Region | 1 | 1 | 108912 | 108913 | 0.00333333 | 4002.155049 | 4002.155052 |
| 4002.155119 | Kernel\_Read\|xilinx:adm-pcie-7v3:1ddr:3.0-0\|cnn\_GOOD | Read | OCL Region | 1 | 1 | 108933 | 108934 | 0.00333333 | 4002.155119 | 4002.155122 |
| 4002.155186 | Kernel\_Read\|xilinx:adm-pcie-7v3:1ddr:3.0-0\|cnn\_GOOD | Read | OCL Region | 1 | 1 | 108953 | 108954 | 0.00333333 | 4002.155186 | 4002.155189 |
| 4002.155249 | Kernel\_Read\|xilinx:adm-pcie-7v3:1ddr:3.0-0\|cnn\_GOOD | Read | OCL Region | 1 | 1 | 108972 | 108973 | 0.00333333 | 4002.155249 | 4002.155252 |
| 4002.155319 | Kernel\_Read\|xilinx:adm-pcie-7v3:1ddr:3.0-0\|cnn\_GOOD | Read | OCL Region | 1 | 1 | 108993 | 108994 | 0.00333333 | 4002.155319 | 4002.155322 |
| 4002.155386 | Kernel\_Read\|xilinx:adm-pcie-7v3:1ddr:3.0-0\|cnn\_GOOD | Read | OCL Region | 1 | 1 | 109013 | 109014 | 0.00333333 | 4002.155386 | 4002.155389 |
| 4002.155449 | Kernel\_Read\|xilinx:adm-pcie-7v3:1ddr:3.0-0\|cnn\_GOOD | Read | OCL Region | 1 | 1 | 109032 | 109033 | 0.00333333 | 4002.155449 | 4002.155452 |
| 4002.155519 | Kernel\_Read\|xilinx:adm-pcie-7v3:1ddr:3.0-0\|cnn\_GOOD | Read | OCL Region | 1 | 1 | 109053 | 109054 | 0.00333333 | 4002.155519 | 4002.155522 |
| 4002.155586 | Kernel\_Read\|xilinx:adm-pcie-7v3:1ddr:3.0-0\|cnn\_GOOD | Read | OCL Region | 1 | 1 | 109073 | 109074 | 0.00333333 | 4002.155586 | 4002.155589 |
| 4002.155649 | Kernel\_Read\|xilinx:adm-pcie-7v3:1ddr:3.0-0\|cnn\_GOOD | Read | OCL Region | 1 | 1 | 109092 | 109093 | 0.00333333 | 4002.155649 | 4002.155652 |
| 4002.155719 | Kernel\_Read\|xilinx:adm-pcie-7v3:1ddr:3.0-0\|cnn\_GOOD | Read | OCL Region | 1 | 1 | 109113 | 109114 | 0.00333333 | 4002.155719 | 4002.155722 |
| 4002.155786 | Kernel\_Read\|xilinx:adm-pcie-7v3:1ddr:3.0-0\|cnn\_GOOD | Read | OCL Region | 1 | 1 | 109133 | 109134 | 0.00333333 | 4002.155786 | 4002.155789 |
| 4002.155849 | Kernel\_Read\|xilinx:adm-pcie-7v3:1ddr:3.0-0\|cnn\_GOOD | Read | OCL Region | 1 | 1 | 109152 | 109153 | 0.00333333 | 4002.155849 | 4002.155852 |
| 4002.155919 | Kernel\_Read\|xilinx:adm-pcie-7v3:1ddr:3.0-0\|cnn\_GOOD | Read | OCL Region | 1 | 1 | 109173 | 109174 | 0.00333333 | 4002.155919 | 4002.155922 |
| 4002.155986 | Kernel\_Read\|xilinx:adm-pcie-7v3:1ddr:3.0-0\|cnn\_GOOD | Read | OCL Region | 1 | 1 | 109193 | 109194 | 0.00333333 | 4002.155986 | 4002.155989 |
| 4002.156049 | Kernel\_Read\|xilinx:adm-pcie-7v3:1ddr:3.0-0\|cnn\_GOOD | Read | OCL Region | 1 | 1 | 109212 | 109213 | 0.00333333 | 4002.156049 | 4002.156052 |
| 4002.156119 | Kernel\_Read\|xilinx:adm-pcie-7v3:1ddr:3.0-0\|cnn\_GOOD | Read | OCL Region | 1 | 1 | 109233 | 109234 | 0.00333333 | 4002.156119 | 4002.156122 |
| 4002.156186 | Kernel\_Read\|xilinx:adm-pcie-7v3:1ddr:3.0-0\|cnn\_GOOD | Read | OCL Region | 1 | 1 | 109253 | 109254 | 0.00333333 | 4002.156186 | 4002.156189 |
| 4002.156259 | Kernel\_Read\|xilinx:adm-pcie-7v3:1ddr:3.0-0\|cnn\_GOOD | Read | OCL Region | 1 | 1 | 109275 | 109276 | 0.00333333 | 4002.156259 | 4002.156262 |
| 4002.156542 | Kernel\_Read\|xilinx:adm-pcie-7v3:1ddr:3.0-0\|cnn\_GOOD | Read | OCL Region | 1 | 1 | 109360 | 109361 | 0.00333333 | 4002.156542 | 4002.156546 |
| 4002.156546 | Kernel\_Read\|xilinx:adm-pcie-7v3:1ddr:3.0-0\|cnn\_GOOD | Read | OCL Region | 1 | 1 | 109361 | 109362 | 0.00333333 | 4002.156546 | 4002.156549 |
| 4002.156549 | Kernel\_Read\|xilinx:adm-pcie-7v3:1ddr:3.0-0\|cnn\_GOOD | Read | OCL Region | 1 | 1 | 109362 | 109363 | 0.00333333 | 4002.156549 | 4002.156552 |
| 4002.156559 | Kernel\_Read\|xilinx:adm-pcie-7v3:1ddr:3.0-0\|cnn\_GOOD | Read | OCL Region | 1 | 1 | 109365 | 109366 | 0.00333333 | 4002.156559 | 4002.156562 |
| 4002.156569 | Kernel\_Read\|xilinx:adm-pcie-7v3:1ddr:3.0-0\|cnn\_GOOD | Read | OCL Region | 1 | 1 | 109368 | 109369 | 0.00333333 | 4002.156569 | 4002.156572 |
| 4002.156649 | Kernel\_Read\|xilinx:adm-pcie-7v3:1ddr:3.0-0\|cnn\_GOOD | Read | OCL Region | 1 | 1 | 109392 | 109393 | 0.00333333 | 4002.156649 | 4002.156652 |
| 4002.156719 | Kernel\_Read\|xilinx:adm-pcie-7v3:1ddr:3.0-0\|cnn\_GOOD | Read | OCL Region | 1 | 1 | 109413 | 109414 | 0.00333333 | 4002.156719 | 4002.156722 |
| 4002.156786 | Kernel\_Read\|xilinx:adm-pcie-7v3:1ddr:3.0-0\|cnn\_GOOD | Read | OCL Region | 1 | 1 | 109433 | 109434 | 0.00333333 | 4002.156786 | 4002.156789 |
| 4002.156849 | Kernel\_Read\|xilinx:adm-pcie-7v3:1ddr:3.0-0\|cnn\_GOOD | Read | OCL Region | 1 | 1 | 109452 | 109453 | 0.00333333 | 4002.156849 | 4002.156852 |
| 4002.156919 | Kernel\_Read\|xilinx:adm-pcie-7v3:1ddr:3.0-0\|cnn\_GOOD | Read | OCL Region | 1 | 1 | 109473 | 109474 | 0.00333333 | 4002.156919 | 4002.156922 |
| 4002.156986 | Kernel\_Read\|xilinx:adm-pcie-7v3:1ddr:3.0-0\|cnn\_GOOD | Read | OCL Region | 1 | 1 | 109493 | 109494 | 0.00333333 | 4002.156986 | 4002.156989 |
| 4002.157059 | Kernel\_Read\|xilinx:adm-pcie-7v3:1ddr:3.0-0\|cnn\_GOOD | Read | OCL Region | 1 | 1 | 109515 | 109516 | 0.00333333 | 4002.157059 | 4002.157062 |
| 4002.157119 | Kernel\_Read\|xilinx:adm-pcie-7v3:1ddr:3.0-0\|cnn\_GOOD | Read | OCL Region | 1 | 1 | 109533 | 109534 | 0.00333333 | 4002.157119 | 4002.157122 |
| 4002.157186 | Kernel\_Read\|xilinx:adm-pcie-7v3:1ddr:3.0-0\|cnn\_GOOD | Read | OCL Region | 1 | 1 | 109553 | 109554 | 0.00333333 | 4002.157186 | 4002.157189 |
| 4002.157259 | Kernel\_Read\|xilinx:adm-pcie-7v3:1ddr:3.0-0\|cnn\_GOOD | Read | OCL Region | 1 | 1 | 109575 | 109576 | 0.00333333 | 4002.157259 | 4002.157262 |
| 4002.157319 | Kernel\_Read\|xilinx:adm-pcie-7v3:1ddr:3.0-0\|cnn\_GOOD | Read | OCL Region | 1 | 1 | 109593 | 109594 | 0.00333333 | 4002.157319 | 4002.157322 |
| 4002.157386 | Kernel\_Read\|xilinx:adm-pcie-7v3:1ddr:3.0-0\|cnn\_GOOD | Read | OCL Region | 1 | 1 | 109613 | 109614 | 0.00333333 | 4002.157386 | 4002.157389 |
| 4002.157449 | Kernel\_Read\|xilinx:adm-pcie-7v3:1ddr:3.0-0\|cnn\_GOOD | Read | OCL Region | 1 | 1 | 109632 | 109633 | 0.00333333 | 4002.157449 | 4002.157452 |
| 4002.157519 | Kernel\_Read\|xilinx:adm-pcie-7v3:1ddr:3.0-0\|cnn\_GOOD | Read | OCL Region | 1 | 1 | 109653 | 109654 | 0.00333333 | 4002.157519 | 4002.157522 |
| 4002.157586 | Kernel\_Read\|xilinx:adm-pcie-7v3:1ddr:3.0-0\|cnn\_GOOD | Read | OCL Region | 1 | 1 | 109673 | 109674 | 0.00333333 | 4002.157586 | 4002.157589 |
| 4002.157659 | Kernel\_Read\|xilinx:adm-pcie-7v3:1ddr:3.0-0\|cnn\_GOOD | Read | OCL Region | 1 | 1 | 109695 | 109696 | 0.00333333 | 4002.157659 | 4002.157662 |
| 4002.157719 | Kernel\_Read\|xilinx:adm-pcie-7v3:1ddr:3.0-0\|cnn\_GOOD | Read | OCL Region | 1 | 1 | 109713 | 109714 | 0.00333333 | 4002.157719 | 4002.157722 |
| 4002.157786 | Kernel\_Read\|xilinx:adm-pcie-7v3:1ddr:3.0-0\|cnn\_GOOD | Read | OCL Region | 1 | 1 | 109733 | 109734 | 0.00333333 | 4002.157786 | 4002.157789 |
| 4002.157859 | Kernel\_Read\|xilinx:adm-pcie-7v3:1ddr:3.0-0\|cnn\_GOOD | Read | OCL Region | 1 | 1 | 109755 | 109756 | 0.00333333 | 4002.157859 | 4002.157862 |
| 4002.157919 | Kernel\_Read\|xilinx:adm-pcie-7v3:1ddr:3.0-0\|cnn\_GOOD | Read | OCL Region | 1 | 1 | 109773 | 109774 | 0.00333333 | 4002.157919 | 4002.157922 |
| 4002.157986 | Kernel\_Read\|xilinx:adm-pcie-7v3:1ddr:3.0-0\|cnn\_GOOD | Read | OCL Region | 1 | 1 | 109793 | 109794 | 0.00333333 | 4002.157986 | 4002.157989 |
| 4002.158059 | Kernel\_Read\|xilinx:adm-pcie-7v3:1ddr:3.0-0\|cnn\_GOOD | Read | OCL Region | 1 | 1 | 109815 | 109816 | 0.00333333 | 4002.158059 | 4002.158062 |
| 4002.158119 | Kernel\_Read\|xilinx:adm-pcie-7v3:1ddr:3.0-0\|cnn\_GOOD | Read | OCL Region | 1 | 1 | 109833 | 109834 | 0.00333333 | 4002.158119 | 4002.158122 |
| 4002.158186 | Kernel\_Read\|xilinx:adm-pcie-7v3:1ddr:3.0-0\|cnn\_GOOD | Read | OCL Region | 1 | 1 | 109853 | 109854 | 0.00333333 | 4002.158186 | 4002.158189 |
| 4002.158249 | Kernel\_Read\|xilinx:adm-pcie-7v3:1ddr:3.0-0\|cnn\_GOOD | Read | OCL Region | 1 | 1 | 109872 | 109873 | 0.00333333 | 4002.158249 | 4002.158252 |
| 4002.158319 | Kernel\_Read\|xilinx:adm-pcie-7v3:1ddr:3.0-0\|cnn\_GOOD | Read | OCL Region | 1 | 1 | 109893 | 109894 | 0.00333333 | 4002.158319 | 4002.158322 |
| 4002.158386 | Kernel\_Read\|xilinx:adm-pcie-7v3:1ddr:3.0-0\|cnn\_GOOD | Read | OCL Region | 1 | 1 | 109913 | 109914 | 0.00333333 | 4002.158386 | 4002.158389 |
| 4002.158459 | Kernel\_Read\|xilinx:adm-pcie-7v3:1ddr:3.0-0\|cnn\_GOOD | Read | OCL Region | 1 | 1 | 109935 | 109936 | 0.00333333 | 4002.158459 | 4002.158462 |
| 4002.158519 | Kernel\_Read\|xilinx:adm-pcie-7v3:1ddr:3.0-0\|cnn\_GOOD | Read | OCL Region | 1 | 1 | 109953 | 109954 | 0.00333333 | 4002.158519 | 4002.158522 |
| 4002.158586 | Kernel\_Read\|xilinx:adm-pcie-7v3:1ddr:3.0-0\|cnn\_GOOD | Read | OCL Region | 1 | 1 | 109973 | 109974 | 0.00333333 | 4002.158586 | 4002.158589 |
| 4002.158659 | Kernel\_Read\|xilinx:adm-pcie-7v3:1ddr:3.0-0\|cnn\_GOOD | Read | OCL Region | 1 | 1 | 109995 | 109996 | 0.00333333 | 4002.158659 | 4002.158662 |
| 4002.158719 | Kernel\_Read\|xilinx:adm-pcie-7v3:1ddr:3.0-0\|cnn\_GOOD | Read | OCL Region | 1 | 1 | 110013 | 110014 | 0.00333333 | 4002.158719 | 4002.158722 |
| 4002.158786 | Kernel\_Read\|xilinx:adm-pcie-7v3:1ddr:3.0-0\|cnn\_GOOD | Read | OCL Region | 1 | 1 | 110033 | 110034 | 0.00333333 | 4002.158786 | 4002.158789 |
| 4002.158859 | Kernel\_Read\|xilinx:adm-pcie-7v3:1ddr:3.0-0\|cnn\_GOOD | Read | OCL Region | 1 | 1 | 110055 | 110056 | 0.00333333 | 4002.158859 | 4002.158862 |
| 4002.158919 | Kernel\_Read\|xilinx:adm-pcie-7v3:1ddr:3.0-0\|cnn\_GOOD | Read | OCL Region | 1 | 1 | 110073 | 110074 | 0.00333333 | 4002.158919 | 4002.158922 |
| 4002.158986 | Kernel\_Read\|xilinx:adm-pcie-7v3:1ddr:3.0-0\|cnn\_GOOD | Read | OCL Region | 1 | 1 | 110093 | 110094 | 0.00333333 | 4002.158986 | 4002.158989 |
| 4002.159059 | Kernel\_Read\|xilinx:adm-pcie-7v3:1ddr:3.0-0\|cnn\_GOOD | Read | OCL Region | 1 | 1 | 110115 | 110116 | 0.00333333 | 4002.159059 | 4002.159062 |
| 4002.159119 | Kernel\_Read\|xilinx:adm-pcie-7v3:1ddr:3.0-0\|cnn\_GOOD | Read | OCL Region | 1 | 1 | 110133 | 110134 | 0.00333333 | 4002.159119 | 4002.159122 |
| 4002.159186 | Kernel\_Read\|xilinx:adm-pcie-7v3:1ddr:3.0-0\|cnn\_GOOD | Read | OCL Region | 1 | 1 | 110153 | 110154 | 0.00333333 | 4002.159186 | 4002.159189 |
| 4002.159259 | Kernel\_Read\|xilinx:adm-pcie-7v3:1ddr:3.0-0\|cnn\_GOOD | Read | OCL Region | 1 | 1 | 110175 | 110176 | 0.00333333 | 4002.159259 | 4002.159262 |
| 4002.159319 | Kernel\_Read\|xilinx:adm-pcie-7v3:1ddr:3.0-0\|cnn\_GOOD | Read | OCL Region | 1 | 1 | 110193 | 110194 | 0.00333333 | 4002.159319 | 4002.159322 |
| 4002.159386 | Kernel\_Read\|xilinx:adm-pcie-7v3:1ddr:3.0-0\|cnn\_GOOD | Read | OCL Region | 1 | 1 | 110213 | 110214 | 0.00333333 | 4002.159386 | 4002.159389 |
| 4002.159459 | Kernel\_Read\|xilinx:adm-pcie-7v3:1ddr:3.0-0\|cnn\_GOOD | Read | OCL Region | 1 | 1 | 110235 | 110236 | 0.00333333 | 4002.159459 | 4002.159462 |
| 4002.159519 | Kernel\_Read\|xilinx:adm-pcie-7v3:1ddr:3.0-0\|cnn\_GOOD | Read | OCL Region | 1 | 1 | 110253 | 110254 | 0.00333333 | 4002.159519 | 4002.159522 |
| 4002.159586 | Kernel\_Read\|xilinx:adm-pcie-7v3:1ddr:3.0-0\|cnn\_GOOD | Read | OCL Region | 1 | 1 | 110273 | 110274 | 0.00333333 | 4002.159586 | 4002.159589 |
| 4002.159659 | Kernel\_Read\|xilinx:adm-pcie-7v3:1ddr:3.0-0\|cnn\_GOOD | Read | OCL Region | 1 | 1 | 110295 | 110296 | 0.00333333 | 4002.159659 | 4002.159662 |
| 4002.159719 | Kernel\_Read\|xilinx:adm-pcie-7v3:1ddr:3.0-0\|cnn\_GOOD | Read | OCL Region | 1 | 1 | 110313 | 110314 | 0.00333333 | 4002.159719 | 4002.159722 |
| 4002.159796 | Kernel\_Read\|xilinx:adm-pcie-7v3:1ddr:3.0-0\|cnn\_GOOD | Read | OCL Region | 1 | 1 | 110336 | 110337 | 0.00333333 | 4002.159796 | 4002.159799 |
| 4002.159859 | Kernel\_Read\|xilinx:adm-pcie-7v3:1ddr:3.0-0\|cnn\_GOOD | Read | OCL Region | 1 | 1 | 110355 | 110356 | 0.00333333 | 4002.159859 | 4002.159862 |
| 4002.159919 | Kernel\_Read\|xilinx:adm-pcie-7v3:1ddr:3.0-0\|cnn\_GOOD | Read | OCL Region | 1 | 1 | 110373 | 110374 | 0.00333333 | 4002.159919 | 4002.159922 |
| 4002.159986 | Kernel\_Read\|xilinx:adm-pcie-7v3:1ddr:3.0-0\|cnn\_GOOD | Read | OCL Region | 1 | 1 | 110393 | 110394 | 0.00333333 | 4002.159986 | 4002.159989 |
| 4002.160059 | Kernel\_Read\|xilinx:adm-pcie-7v3:1ddr:3.0-0\|cnn\_GOOD | Read | OCL Region | 1 | 1 | 110415 | 110416 | 0.00333333 | 4002.160059 | 4002.160062 |
| 4002.160119 | Kernel\_Read\|xilinx:adm-pcie-7v3:1ddr:3.0-0\|cnn\_GOOD | Read | OCL Region | 1 | 1 | 110433 | 110434 | 0.00333333 | 4002.160119 | 4002.160122 |
| 4002.160186 | Kernel\_Read\|xilinx:adm-pcie-7v3:1ddr:3.0-0\|cnn\_GOOD | Read | OCL Region | 1 | 1 | 110453 | 110454 | 0.00333333 | 4002.160186 | 4002.160189 |
| 4002.160259 | Kernel\_Read\|xilinx:adm-pcie-7v3:1ddr:3.0-0\|cnn\_GOOD | Read | OCL Region | 1 | 1 | 110475 | 110476 | 0.00333333 | 4002.160259 | 4002.160262 |
| 4002.160319 | Kernel\_Read\|xilinx:adm-pcie-7v3:1ddr:3.0-0\|cnn\_GOOD | Read | OCL Region | 1 | 1 | 110493 | 110494 | 0.00333333 | 4002.160319 | 4002.160322 |
| 4002.160386 | Kernel\_Read\|xilinx:adm-pcie-7v3:1ddr:3.0-0\|cnn\_GOOD | Read | OCL Region | 1 | 1 | 110513 | 110514 | 0.00333333 | 4002.160386 | 4002.160389 |
| 4002.160459 | Kernel\_Read\|xilinx:adm-pcie-7v3:1ddr:3.0-0\|cnn\_GOOD | Read | OCL Region | 1 | 1 | 110535 | 110536 | 0.00333333 | 4002.160459 | 4002.160462 |
| 4002.160519 | Kernel\_Read\|xilinx:adm-pcie-7v3:1ddr:3.0-0\|cnn\_GOOD | Read | OCL Region | 1 | 1 | 110553 | 110554 | 0.00333333 | 4002.160519 | 4002.160522 |
| 4002.160586 | Kernel\_Read\|xilinx:adm-pcie-7v3:1ddr:3.0-0\|cnn\_GOOD | Read | OCL Region | 1 | 1 | 110573 | 110574 | 0.00333333 | 4002.160586 | 4002.160589 |
| 4002.160659 | Kernel\_Read\|xilinx:adm-pcie-7v3:1ddr:3.0-0\|cnn\_GOOD | Read | OCL Region | 1 | 1 | 110595 | 110596 | 0.00333333 | 4002.160659 | 4002.160662 |
| 4002.160719 | Kernel\_Read\|xilinx:adm-pcie-7v3:1ddr:3.0-0\|cnn\_GOOD | Read | OCL Region | 1 | 1 | 110613 | 110614 | 0.00333333 | 4002.160719 | 4002.160722 |
| 4002.160786 | Kernel\_Read\|xilinx:adm-pcie-7v3:1ddr:3.0-0\|cnn\_GOOD | Read | OCL Region | 1 | 1 | 110633 | 110634 | 0.00333333 | 4002.160786 | 4002.160789 |
| 4002.160859 | Kernel\_Read\|xilinx:adm-pcie-7v3:1ddr:3.0-0\|cnn\_GOOD | Read | OCL Region | 1 | 1 | 110655 | 110656 | 0.00333333 | 4002.160859 | 4002.160862 |
| 4002.160919 | Kernel\_Read\|xilinx:adm-pcie-7v3:1ddr:3.0-0\|cnn\_GOOD | Read | OCL Region | 1 | 1 | 110673 | 110674 | 0.00333333 | 4002.160919 | 4002.160922 |
| 4002.160986 | Kernel\_Read\|xilinx:adm-pcie-7v3:1ddr:3.0-0\|cnn\_GOOD | Read | OCL Region | 1 | 1 | 110693 | 110694 | 0.00333333 | 4002.160986 | 4002.160989 |
| 4002.161059 | Kernel\_Read\|xilinx:adm-pcie-7v3:1ddr:3.0-0\|cnn\_GOOD | Read | OCL Region | 1 | 1 | 110715 | 110716 | 0.00333333 | 4002.161059 | 4002.161062 |
| 4002.161119 | Kernel\_Read\|xilinx:adm-pcie-7v3:1ddr:3.0-0\|cnn\_GOOD | Read | OCL Region | 1 | 1 | 110733 | 110734 | 0.00333333 | 4002.161119 | 4002.161122 |
| 4002.161186 | Kernel\_Read\|xilinx:adm-pcie-7v3:1ddr:3.0-0\|cnn\_GOOD | Read | OCL Region | 1 | 1 | 110753 | 110754 | 0.00333333 | 4002.161186 | 4002.161189 |
| 4002.161259 | Kernel\_Read\|xilinx:adm-pcie-7v3:1ddr:3.0-0\|cnn\_GOOD | Read | OCL Region | 1 | 1 | 110775 | 110776 | 0.00333333 | 4002.161259 | 4002.161262 |
| 4002.161319 | Kernel\_Read\|xilinx:adm-pcie-7v3:1ddr:3.0-0\|cnn\_GOOD | Read | OCL Region | 1 | 1 | 110793 | 110794 | 0.00333333 | 4002.161319 | 4002.161322 |
| 4002.161386 | Kernel\_Read\|xilinx:adm-pcie-7v3:1ddr:3.0-0\|cnn\_GOOD | Read | OCL Region | 1 | 1 | 110813 | 110814 | 0.00333333 | 4002.161386 | 4002.161389 |
| 4002.161449 | Kernel\_Read\|xilinx:adm-pcie-7v3:1ddr:3.0-0\|cnn\_GOOD | Read | OCL Region | 1 | 1 | 110832 | 110833 | 0.00333333 | 4002.161449 | 4002.161452 |
| 4002.161519 | Kernel\_Read\|xilinx:adm-pcie-7v3:1ddr:3.0-0\|cnn\_GOOD | Read | OCL Region | 1 | 1 | 110853 | 110854 | 0.00333333 | 4002.161519 | 4002.161522 |
| 4002.161586 | Kernel\_Read\|xilinx:adm-pcie-7v3:1ddr:3.0-0\|cnn\_GOOD | Read | OCL Region | 1 | 1 | 110873 | 110874 | 0.00333333 | 4002.161586 | 4002.161589 |
| 4002.161659 | Kernel\_Read\|xilinx:adm-pcie-7v3:1ddr:3.0-0\|cnn\_GOOD | Read | OCL Region | 1 | 1 | 110895 | 110896 | 0.00333333 | 4002.161659 | 4002.161662 |
| 4002.161719 | Kernel\_Read\|xilinx:adm-pcie-7v3:1ddr:3.0-0\|cnn\_GOOD | Read | OCL Region | 1 | 1 | 110913 | 110914 | 0.00333333 | 4002.161719 | 4002.161722 |
| 4002.161786 | Kernel\_Read\|xilinx:adm-pcie-7v3:1ddr:3.0-0\|cnn\_GOOD | Read | OCL Region | 1 | 1 | 110933 | 110934 | 0.00333333 | 4002.161786 | 4002.161789 |
| 4002.161859 | Kernel\_Read\|xilinx:adm-pcie-7v3:1ddr:3.0-0\|cnn\_GOOD | Read | OCL Region | 1 | 1 | 110955 | 110956 | 0.00333333 | 4002.161859 | 4002.161862 |
| 4002.161919 | Kernel\_Read\|xilinx:adm-pcie-7v3:1ddr:3.0-0\|cnn\_GOOD | Read | OCL Region | 1 | 1 | 110973 | 110974 | 0.00333333 | 4002.161919 | 4002.161922 |
| 4002.161986 | Kernel\_Read\|xilinx:adm-pcie-7v3:1ddr:3.0-0\|cnn\_GOOD | Read | OCL Region | 1 | 1 | 110993 | 110994 | 0.00333333 | 4002.161986 | 4002.161989 |
| 4002.162059 | Kernel\_Read\|xilinx:adm-pcie-7v3:1ddr:3.0-0\|cnn\_GOOD | Read | OCL Region | 1 | 1 | 111015 | 111016 | 0.00333333 | 4002.162059 | 4002.162062 |
| 4002.162119 | Kernel\_Read\|xilinx:adm-pcie-7v3:1ddr:3.0-0\|cnn\_GOOD | Read | OCL Region | 1 | 1 | 111033 | 111034 | 0.00333333 | 4002.162119 | 4002.162122 |
| 4002.162186 | Kernel\_Read\|xilinx:adm-pcie-7v3:1ddr:3.0-0\|cnn\_GOOD | Read | OCL Region | 1 | 1 | 111053 | 111054 | 0.00333333 | 4002.162186 | 4002.162189 |
| 4002.162259 | Kernel\_Read\|xilinx:adm-pcie-7v3:1ddr:3.0-0\|cnn\_GOOD | Read | OCL Region | 1 | 1 | 111075 | 111076 | 0.00333333 | 4002.162259 | 4002.162262 |
| 4002.162319 | Kernel\_Read\|xilinx:adm-pcie-7v3:1ddr:3.0-0\|cnn\_GOOD | Read | OCL Region | 1 | 1 | 111093 | 111094 | 0.00333333 | 4002.162319 | 4002.162322 |
| 4002.162386 | Kernel\_Read\|xilinx:adm-pcie-7v3:1ddr:3.0-0\|cnn\_GOOD | Read | OCL Region | 1 | 1 | 111113 | 111114 | 0.00333333 | 4002.162386 | 4002.162389 |
| 4002.162459 | Kernel\_Read\|xilinx:adm-pcie-7v3:1ddr:3.0-0\|cnn\_GOOD | Read | OCL Region | 1 | 1 | 111135 | 111136 | 0.00333333 | 4002.162459 | 4002.162462 |
| 4002.162519 | Kernel\_Read\|xilinx:adm-pcie-7v3:1ddr:3.0-0\|cnn\_GOOD | Read | OCL Region | 1 | 1 | 111153 | 111154 | 0.00333333 | 4002.162519 | 4002.162522 |
| 4002.162586 | Kernel\_Read\|xilinx:adm-pcie-7v3:1ddr:3.0-0\|cnn\_GOOD | Read | OCL Region | 1 | 1 | 111173 | 111174 | 0.00333333 | 4002.162586 | 4002.162589 |
| 4002.162862 | Kernel\_Read\|xilinx:adm-pcie-7v3:1ddr:3.0-0\|cnn\_GOOD | Read | OCL Region | 1 | 1 | 111256 | 111257 | 0.00333333 | 4002.162862 | 4002.162866 |
| 4002.162866 | Kernel\_Read\|xilinx:adm-pcie-7v3:1ddr:3.0-0\|cnn\_GOOD | Read | OCL Region | 1 | 1 | 111257 | 111258 | 0.00333333 | 4002.162866 | 4002.162869 |
| 4002.162869 | Kernel\_Read\|xilinx:adm-pcie-7v3:1ddr:3.0-0\|cnn\_GOOD | Read | OCL Region | 1 | 1 | 111258 | 111259 | 0.00333333 | 4002.162869 | 4002.162872 |
| 4002.162876 | Kernel\_Read\|xilinx:adm-pcie-7v3:1ddr:3.0-0\|cnn\_GOOD | Read | OCL Region | 1 | 1 | 111260 | 111261 | 0.00333333 | 4002.162876 | 4002.162879 |
| 4002.162936 | Kernel\_Read\|xilinx:adm-pcie-7v3:1ddr:3.0-0\|cnn\_GOOD | Read | OCL Region | 1 | 1 | 111278 | 111279 | 0.00333333 | 4002.162936 | 4002.162939 |
| 4002.162992 | Kernel\_Read\|xilinx:adm-pcie-7v3:1ddr:3.0-0\|cnn\_GOOD | Read | OCL Region | 1 | 1 | 111295 | 111296 | 0.00333333 | 4002.162992 | 4002.162996 |
| 4002.163059 | Kernel\_Read\|xilinx:adm-pcie-7v3:1ddr:3.0-0\|cnn\_GOOD | Read | OCL Region | 1 | 1 | 111315 | 111316 | 0.00333333 | 4002.163059 | 4002.163062 |
| 4002.163119 | Kernel\_Read\|xilinx:adm-pcie-7v3:1ddr:3.0-0\|cnn\_GOOD | Read | OCL Region | 1 | 1 | 111333 | 111334 | 0.00333333 | 4002.163119 | 4002.163122 |
| 4002.163186 | Kernel\_Read\|xilinx:adm-pcie-7v3:1ddr:3.0-0\|cnn\_GOOD | Read | OCL Region | 1 | 1 | 111353 | 111354 | 0.00333333 | 4002.163186 | 4002.163189 |
| 4002.163259 | Kernel\_Read\|xilinx:adm-pcie-7v3:1ddr:3.0-0\|cnn\_GOOD | Read | OCL Region | 1 | 1 | 111375 | 111376 | 0.00333333 | 4002.163259 | 4002.163262 |
| 4002.163319 | Kernel\_Read\|xilinx:adm-pcie-7v3:1ddr:3.0-0\|cnn\_GOOD | Read | OCL Region | 1 | 1 | 111393 | 111394 | 0.00333333 | 4002.163319 | 4002.163322 |
| 4002.163386 | Kernel\_Read\|xilinx:adm-pcie-7v3:1ddr:3.0-0\|cnn\_GOOD | Read | OCL Region | 1 | 1 | 111413 | 111414 | 0.00333333 | 4002.163386 | 4002.163389 |
| 4002.163459 | Kernel\_Read\|xilinx:adm-pcie-7v3:1ddr:3.0-0\|cnn\_GOOD | Read | OCL Region | 1 | 1 | 111435 | 111436 | 0.00333333 | 4002.163459 | 4002.163462 |
| 4002.163519 | Kernel\_Read\|xilinx:adm-pcie-7v3:1ddr:3.0-0\|cnn\_GOOD | Read | OCL Region | 1 | 1 | 111453 | 111454 | 0.00333333 | 4002.163519 | 4002.163522 |
| 4002.163586 | Kernel\_Read\|xilinx:adm-pcie-7v3:1ddr:3.0-0\|cnn\_GOOD | Read | OCL Region | 1 | 1 | 111473 | 111474 | 0.00333333 | 4002.163586 | 4002.163589 |
| 4002.163659 | Kernel\_Read\|xilinx:adm-pcie-7v3:1ddr:3.0-0\|cnn\_GOOD | Read | OCL Region | 1 | 1 | 111495 | 111496 | 0.00333333 | 4002.163659 | 4002.163662 |
| 4002.163719 | Kernel\_Read\|xilinx:adm-pcie-7v3:1ddr:3.0-0\|cnn\_GOOD | Read | OCL Region | 1 | 1 | 111513 | 111514 | 0.00333333 | 4002.163719 | 4002.163722 |
| 4002.163792 | Kernel\_Read\|xilinx:adm-pcie-7v3:1ddr:3.0-0\|cnn\_GOOD | Read | OCL Region | 1 | 1 | 111535 | 111536 | 0.00333333 | 4002.163792 | 4002.163796 |
| 4002.163859 | Kernel\_Read\|xilinx:adm-pcie-7v3:1ddr:3.0-0\|cnn\_GOOD | Read | OCL Region | 1 | 1 | 111555 | 111556 | 0.00333333 | 4002.163859 | 4002.163862 |
| 4002.163919 | Kernel\_Read\|xilinx:adm-pcie-7v3:1ddr:3.0-0\|cnn\_GOOD | Read | OCL Region | 1 | 1 | 111573 | 111574 | 0.00333333 | 4002.163919 | 4002.163922 |
| 4002.163986 | Kernel\_Read\|xilinx:adm-pcie-7v3:1ddr:3.0-0\|cnn\_GOOD | Read | OCL Region | 1 | 1 | 111593 | 111594 | 0.00333333 | 4002.163986 | 4002.163989 |
| 4002.164059 | Kernel\_Read\|xilinx:adm-pcie-7v3:1ddr:3.0-0\|cnn\_GOOD | Read | OCL Region | 1 | 1 | 111615 | 111616 | 0.00333333 | 4002.164059 | 4002.164062 |
| 4002.164119 | Kernel\_Read\|xilinx:adm-pcie-7v3:1ddr:3.0-0\|cnn\_GOOD | Read | OCL Region | 1 | 1 | 111633 | 111634 | 0.00333333 | 4002.164119 | 4002.164122 |
| 4002.164186 | Kernel\_Read\|xilinx:adm-pcie-7v3:1ddr:3.0-0\|cnn\_GOOD | Read | OCL Region | 1 | 1 | 111653 | 111654 | 0.00333333 | 4002.164186 | 4002.164189 |
| 4002.164259 | Kernel\_Read\|xilinx:adm-pcie-7v3:1ddr:3.0-0\|cnn\_GOOD | Read | OCL Region | 1 | 1 | 111675 | 111676 | 0.00333333 | 4002.164259 | 4002.164262 |
| 4002.164319 | Kernel\_Read\|xilinx:adm-pcie-7v3:1ddr:3.0-0\|cnn\_GOOD | Read | OCL Region | 1 | 1 | 111693 | 111694 | 0.00333333 | 4002.164319 | 4002.164322 |
| 4002.164386 | Kernel\_Read\|xilinx:adm-pcie-7v3:1ddr:3.0-0\|cnn\_GOOD | Read | OCL Region | 1 | 1 | 111713 | 111714 | 0.00333333 | 4002.164386 | 4002.164389 |
| 4002.164459 | Kernel\_Read\|xilinx:adm-pcie-7v3:1ddr:3.0-0\|cnn\_GOOD | Read | OCL Region | 1 | 1 | 111735 | 111736 | 0.00333333 | 4002.164459 | 4002.164462 |
| 4002.164519 | Kernel\_Read\|xilinx:adm-pcie-7v3:1ddr:3.0-0\|cnn\_GOOD | Read | OCL Region | 1 | 1 | 111753 | 111754 | 0.00333333 | 4002.164519 | 4002.164522 |
| 4002.164586 | Kernel\_Read\|xilinx:adm-pcie-7v3:1ddr:3.0-0\|cnn\_GOOD | Read | OCL Region | 1 | 1 | 111773 | 111774 | 0.00333333 | 4002.164586 | 4002.164589 |
| 4002.164659 | Kernel\_Read\|xilinx:adm-pcie-7v3:1ddr:3.0-0\|cnn\_GOOD | Read | OCL Region | 1 | 1 | 111795 | 111796 | 0.00333333 | 4002.164659 | 4002.164662 |
| 4002.164719 | Kernel\_Read\|xilinx:adm-pcie-7v3:1ddr:3.0-0\|cnn\_GOOD | Read | OCL Region | 1 | 1 | 111813 | 111814 | 0.00333333 | 4002.164719 | 4002.164722 |
| 4002.164786 | Kernel\_Read\|xilinx:adm-pcie-7v3:1ddr:3.0-0\|cnn\_GOOD | Read | OCL Region | 1 | 1 | 111833 | 111834 | 0.00333333 | 4002.164786 | 4002.164789 |
| 4002.164859 | Kernel\_Read\|xilinx:adm-pcie-7v3:1ddr:3.0-0\|cnn\_GOOD | Read | OCL Region | 1 | 1 | 111855 | 111856 | 0.00333333 | 4002.164859 | 4002.164862 |
| 4002.164919 | Kernel\_Read\|xilinx:adm-pcie-7v3:1ddr:3.0-0\|cnn\_GOOD | Read | OCL Region | 1 | 1 | 111873 | 111874 | 0.00333333 | 4002.164919 | 4002.164922 |
| 4002.164986 | Kernel\_Read\|xilinx:adm-pcie-7v3:1ddr:3.0-0\|cnn\_GOOD | Read | OCL Region | 1 | 1 | 111893 | 111894 | 0.00333333 | 4002.164986 | 4002.164989 |
| 4002.165059 | Kernel\_Read\|xilinx:adm-pcie-7v3:1ddr:3.0-0\|cnn\_GOOD | Read | OCL Region | 1 | 1 | 111915 | 111916 | 0.00333333 | 4002.165059 | 4002.165062 |
| 4002.165119 | Kernel\_Read\|xilinx:adm-pcie-7v3:1ddr:3.0-0\|cnn\_GOOD | Read | OCL Region | 1 | 1 | 111933 | 111934 | 0.00333333 | 4002.165119 | 4002.165122 |
| 4002.165186 | Kernel\_Read\|xilinx:adm-pcie-7v3:1ddr:3.0-0\|cnn\_GOOD | Read | OCL Region | 1 | 1 | 111953 | 111954 | 0.00333333 | 4002.165186 | 4002.165189 |
| 4002.165259 | Kernel\_Read\|xilinx:adm-pcie-7v3:1ddr:3.0-0\|cnn\_GOOD | Read | OCL Region | 1 | 1 | 111975 | 111976 | 0.00333333 | 4002.165259 | 4002.165262 |
| 4002.165319 | Kernel\_Read\|xilinx:adm-pcie-7v3:1ddr:3.0-0\|cnn\_GOOD | Read | OCL Region | 1 | 1 | 111993 | 111994 | 0.00333333 | 4002.165319 | 4002.165322 |
| 4002.165386 | Kernel\_Read\|xilinx:adm-pcie-7v3:1ddr:3.0-0\|cnn\_GOOD | Read | OCL Region | 1 | 1 | 112013 | 112014 | 0.00333333 | 4002.165386 | 4002.165389 |
| 4002.165459 | Kernel\_Read\|xilinx:adm-pcie-7v3:1ddr:3.0-0\|cnn\_GOOD | Read | OCL Region | 1 | 1 | 112035 | 112036 | 0.00333333 | 4002.165459 | 4002.165462 |
| 4002.165519 | Kernel\_Read\|xilinx:adm-pcie-7v3:1ddr:3.0-0\|cnn\_GOOD | Read | OCL Region | 1 | 1 | 112053 | 112054 | 0.00333333 | 4002.165519 | 4002.165522 |
| 4002.165586 | Kernel\_Read\|xilinx:adm-pcie-7v3:1ddr:3.0-0\|cnn\_GOOD | Read | OCL Region | 1 | 1 | 112073 | 112074 | 0.00333333 | 4002.165586 | 4002.165589 |
| 4002.165659 | Kernel\_Read\|xilinx:adm-pcie-7v3:1ddr:3.0-0\|cnn\_GOOD | Read | OCL Region | 1 | 1 | 112095 | 112096 | 0.00333333 | 4002.165659 | 4002.165662 |
| 4002.165719 | Kernel\_Read\|xilinx:adm-pcie-7v3:1ddr:3.0-0\|cnn\_GOOD | Read | OCL Region | 1 | 1 | 112113 | 112114 | 0.00333333 | 4002.165719 | 4002.165722 |
| 4002.165786 | Kernel\_Read\|xilinx:adm-pcie-7v3:1ddr:3.0-0\|cnn\_GOOD | Read | OCL Region | 1 | 1 | 112133 | 112134 | 0.00333333 | 4002.165786 | 4002.165789 |
| 4002.165859 | Kernel\_Read\|xilinx:adm-pcie-7v3:1ddr:3.0-0\|cnn\_GOOD | Read | OCL Region | 1 | 1 | 112155 | 112156 | 0.00333333 | 4002.165859 | 4002.165862 |
| 4002.165919 | Kernel\_Read\|xilinx:adm-pcie-7v3:1ddr:3.0-0\|cnn\_GOOD | Read | OCL Region | 1 | 1 | 112173 | 112174 | 0.00333333 | 4002.165919 | 4002.165922 |
| 4002.165986 | Kernel\_Read\|xilinx:adm-pcie-7v3:1ddr:3.0-0\|cnn\_GOOD | Read | OCL Region | 1 | 1 | 112193 | 112194 | 0.00333333 | 4002.165986 | 4002.165989 |
| 4002.166059 | Kernel\_Read\|xilinx:adm-pcie-7v3:1ddr:3.0-0\|cnn\_GOOD | Read | OCL Region | 1 | 1 | 112215 | 112216 | 0.00333333 | 4002.166059 | 4002.166062 |
| 4002.166119 | Kernel\_Read\|xilinx:adm-pcie-7v3:1ddr:3.0-0\|cnn\_GOOD | Read | OCL Region | 1 | 1 | 112233 | 112234 | 0.00333333 | 4002.166119 | 4002.166122 |
| 4002.166186 | Kernel\_Read\|xilinx:adm-pcie-7v3:1ddr:3.0-0\|cnn\_GOOD | Read | OCL Region | 1 | 1 | 112253 | 112254 | 0.00333333 | 4002.166186 | 4002.166189 |
| 4002.166266 | Kernel\_Read\|xilinx:adm-pcie-7v3:1ddr:3.0-0\|cnn\_GOOD | Read | OCL Region | 1 | 1 | 112277 | 112278 | 0.00333333 | 4002.166266 | 4002.166269 |
| 4002.166319 | Kernel\_Read\|xilinx:adm-pcie-7v3:1ddr:3.0-0\|cnn\_GOOD | Read | OCL Region | 1 | 1 | 112293 | 112294 | 0.00333333 | 4002.166319 | 4002.166322 |
| 4002.166386 | Kernel\_Read\|xilinx:adm-pcie-7v3:1ddr:3.0-0\|cnn\_GOOD | Read | OCL Region | 1 | 1 | 112313 | 112314 | 0.00333333 | 4002.166386 | 4002.166389 |
| 4002.166459 | Kernel\_Read\|xilinx:adm-pcie-7v3:1ddr:3.0-0\|cnn\_GOOD | Read | OCL Region | 1 | 1 | 112335 | 112336 | 0.00333333 | 4002.166459 | 4002.166462 |
| 4002.166519 | Kernel\_Read\|xilinx:adm-pcie-7v3:1ddr:3.0-0\|cnn\_GOOD | Read | OCL Region | 1 | 1 | 112353 | 112354 | 0.00333333 | 4002.166519 | 4002.166522 |
| 4002.166586 | Kernel\_Read\|xilinx:adm-pcie-7v3:1ddr:3.0-0\|cnn\_GOOD | Read | OCL Region | 1 | 1 | 112373 | 112374 | 0.00333333 | 4002.166586 | 4002.166589 |
| 4002.166659 | Kernel\_Read\|xilinx:adm-pcie-7v3:1ddr:3.0-0\|cnn\_GOOD | Read | OCL Region | 1 | 1 | 112395 | 112396 | 0.00333333 | 4002.166659 | 4002.166662 |
| 4002.166719 | Kernel\_Read\|xilinx:adm-pcie-7v3:1ddr:3.0-0\|cnn\_GOOD | Read | OCL Region | 1 | 1 | 112413 | 112414 | 0.00333333 | 4002.166719 | 4002.166722 |
| 4002.166786 | Kernel\_Read\|xilinx:adm-pcie-7v3:1ddr:3.0-0\|cnn\_GOOD | Read | OCL Region | 1 | 1 | 112433 | 112434 | 0.00333333 | 4002.166786 | 4002.166789 |
| 4002.166859 | Kernel\_Read\|xilinx:adm-pcie-7v3:1ddr:3.0-0\|cnn\_GOOD | Read | OCL Region | 1 | 1 | 112455 | 112456 | 0.00333333 | 4002.166859 | 4002.166862 |
| 4002.166919 | Kernel\_Read\|xilinx:adm-pcie-7v3:1ddr:3.0-0\|cnn\_GOOD | Read | OCL Region | 1 | 1 | 112473 | 112474 | 0.00333333 | 4002.166919 | 4002.166922 |
| 4002.166986 | Kernel\_Read\|xilinx:adm-pcie-7v3:1ddr:3.0-0\|cnn\_GOOD | Read | OCL Region | 1 | 1 | 112493 | 112494 | 0.00333333 | 4002.166986 | 4002.166989 |
| 4002.167059 | Kernel\_Read\|xilinx:adm-pcie-7v3:1ddr:3.0-0\|cnn\_GOOD | Read | OCL Region | 1 | 1 | 112515 | 112516 | 0.00333333 | 4002.167059 | 4002.167062 |
| 4002.167119 | Kernel\_Read\|xilinx:adm-pcie-7v3:1ddr:3.0-0\|cnn\_GOOD | Read | OCL Region | 1 | 1 | 112533 | 112534 | 0.00333333 | 4002.167119 | 4002.167122 |
| 4002.167186 | Kernel\_Read\|xilinx:adm-pcie-7v3:1ddr:3.0-0\|cnn\_GOOD | Read | OCL Region | 1 | 1 | 112553 | 112554 | 0.00333333 | 4002.167186 | 4002.167189 |
| 4002.167259 | Kernel\_Read\|xilinx:adm-pcie-7v3:1ddr:3.0-0\|cnn\_GOOD | Read | OCL Region | 1 | 1 | 112575 | 112576 | 0.00333333 | 4002.167259 | 4002.167262 |
| 4002.167319 | Kernel\_Read\|xilinx:adm-pcie-7v3:1ddr:3.0-0\|cnn\_GOOD | Read | OCL Region | 1 | 1 | 112593 | 112594 | 0.00333333 | 4002.167319 | 4002.167322 |
| 4002.167386 | Kernel\_Read\|xilinx:adm-pcie-7v3:1ddr:3.0-0\|cnn\_GOOD | Read | OCL Region | 1 | 1 | 112613 | 112614 | 0.00333333 | 4002.167386 | 4002.167389 |
| 4002.167459 | Kernel\_Read\|xilinx:adm-pcie-7v3:1ddr:3.0-0\|cnn\_GOOD | Read | OCL Region | 1 | 1 | 112635 | 112636 | 0.00333333 | 4002.167459 | 4002.167462 |
| 4002.167519 | Kernel\_Read\|xilinx:adm-pcie-7v3:1ddr:3.0-0\|cnn\_GOOD | Read | OCL Region | 1 | 1 | 112653 | 112654 | 0.00333333 | 4002.167519 | 4002.167522 |
| 4002.167586 | Kernel\_Read\|xilinx:adm-pcie-7v3:1ddr:3.0-0\|cnn\_GOOD | Read | OCL Region | 1 | 1 | 112673 | 112674 | 0.00333333 | 4002.167586 | 4002.167589 |
| 4002.167659 | Kernel\_Read\|xilinx:adm-pcie-7v3:1ddr:3.0-0\|cnn\_GOOD | Read | OCL Region | 1 | 1 | 112695 | 112696 | 0.00333333 | 4002.167659 | 4002.167662 |
| 4002.167719 | Kernel\_Read\|xilinx:adm-pcie-7v3:1ddr:3.0-0\|cnn\_GOOD | Read | OCL Region | 1 | 1 | 112713 | 112714 | 0.00333333 | 4002.167719 | 4002.167722 |
| 4002.167786 | Kernel\_Read\|xilinx:adm-pcie-7v3:1ddr:3.0-0\|cnn\_GOOD | Read | OCL Region | 1 | 1 | 112733 | 112734 | 0.00333333 | 4002.167786 | 4002.167789 |
| 4002.167859 | Kernel\_Read\|xilinx:adm-pcie-7v3:1ddr:3.0-0\|cnn\_GOOD | Read | OCL Region | 1 | 1 | 112755 | 112756 | 0.00333333 | 4002.167859 | 4002.167862 |
| 4002.167919 | Kernel\_Read\|xilinx:adm-pcie-7v3:1ddr:3.0-0\|cnn\_GOOD | Read | OCL Region | 1 | 1 | 112773 | 112774 | 0.00333333 | 4002.167919 | 4002.167922 |
| 4002.167986 | Kernel\_Read\|xilinx:adm-pcie-7v3:1ddr:3.0-0\|cnn\_GOOD | Read | OCL Region | 1 | 1 | 112793 | 112794 | 0.00333333 | 4002.167986 | 4002.167989 |
| 4002.168059 | Kernel\_Read\|xilinx:adm-pcie-7v3:1ddr:3.0-0\|cnn\_GOOD | Read | OCL Region | 1 | 1 | 112815 | 112816 | 0.00333333 | 4002.168059 | 4002.168062 |
| 4002.168119 | Kernel\_Read\|xilinx:adm-pcie-7v3:1ddr:3.0-0\|cnn\_GOOD | Read | OCL Region | 1 | 1 | 112833 | 112834 | 0.00333333 | 4002.168119 | 4002.168122 |
| 4002.168186 | Kernel\_Read\|xilinx:adm-pcie-7v3:1ddr:3.0-0\|cnn\_GOOD | Read | OCL Region | 1 | 1 | 112853 | 112854 | 0.00333333 | 4002.168186 | 4002.168189 |
| 4002.168259 | Kernel\_Read\|xilinx:adm-pcie-7v3:1ddr:3.0-0\|cnn\_GOOD | Read | OCL Region | 1 | 1 | 112875 | 112876 | 0.00333333 | 4002.168259 | 4002.168262 |
| 4002.168319 | Kernel\_Read\|xilinx:adm-pcie-7v3:1ddr:3.0-0\|cnn\_GOOD | Read | OCL Region | 1 | 1 | 112893 | 112894 | 0.00333333 | 4002.168319 | 4002.168322 |
| 4002.168386 | Kernel\_Read\|xilinx:adm-pcie-7v3:1ddr:3.0-0\|cnn\_GOOD | Read | OCL Region | 1 | 1 | 112913 | 112914 | 0.00333333 | 4002.168386 | 4002.168389 |
| 4002.168459 | Kernel\_Read\|xilinx:adm-pcie-7v3:1ddr:3.0-0\|cnn\_GOOD | Read | OCL Region | 1 | 1 | 112935 | 112936 | 0.00333333 | 4002.168459 | 4002.168462 |
| 4002.168519 | Kernel\_Read\|xilinx:adm-pcie-7v3:1ddr:3.0-0\|cnn\_GOOD | Read | OCL Region | 1 | 1 | 112953 | 112954 | 0.00333333 | 4002.168519 | 4002.168522 |
| 4002.168586 | Kernel\_Read\|xilinx:adm-pcie-7v3:1ddr:3.0-0\|cnn\_GOOD | Read | OCL Region | 1 | 1 | 112973 | 112974 | 0.00333333 | 4002.168586 | 4002.168589 |
| 4002.168659 | Kernel\_Read\|xilinx:adm-pcie-7v3:1ddr:3.0-0\|cnn\_GOOD | Read | OCL Region | 1 | 1 | 112995 | 112996 | 0.00333333 | 4002.168659 | 4002.168662 |
| 4002.168719 | Kernel\_Read\|xilinx:adm-pcie-7v3:1ddr:3.0-0\|cnn\_GOOD | Read | OCL Region | 1 | 1 | 113013 | 113014 | 0.00333333 | 4002.168719 | 4002.168722 |
| 4002.168786 | Kernel\_Read\|xilinx:adm-pcie-7v3:1ddr:3.0-0\|cnn\_GOOD | Read | OCL Region | 1 | 1 | 113033 | 113034 | 0.00333333 | 4002.168786 | 4002.168789 |
| 4002.168859 | Kernel\_Read\|xilinx:adm-pcie-7v3:1ddr:3.0-0\|cnn\_GOOD | Read | OCL Region | 1 | 1 | 113055 | 113056 | 0.00333333 | 4002.168859 | 4002.168862 |
| 4002.169132 | Kernel\_Read\|xilinx:adm-pcie-7v3:1ddr:3.0-0\|cnn\_GOOD | Read | OCL Region | 1 | 1 | 113137 | 113138 | 0.00333333 | 4002.169132 | 4002.169136 |
| 4002.169136 | Kernel\_Read\|xilinx:adm-pcie-7v3:1ddr:3.0-0\|cnn\_GOOD | Read | OCL Region | 1 | 1 | 113138 | 113139 | 0.00333333 | 4002.169136 | 4002.169139 |
| 4002.169142 | Kernel\_Read\|xilinx:adm-pcie-7v3:1ddr:3.0-0\|cnn\_GOOD | Read | OCL Region | 1 | 1 | 113140 | 113141 | 0.00333333 | 4002.169142 | 4002.169146 |
| 4002.169146 | Kernel\_Read\|xilinx:adm-pcie-7v3:1ddr:3.0-0\|cnn\_GOOD | Read | OCL Region | 1 | 1 | 113141 | 113142 | 0.00333333 | 4002.169146 | 4002.169149 |
| 4002.169209 | Kernel\_Read\|xilinx:adm-pcie-7v3:1ddr:3.0-0\|cnn\_GOOD | Read | OCL Region | 1 | 1 | 113160 | 113161 | 0.00333333 | 4002.169209 | 4002.169212 |
| 4002.169262 | Kernel\_Read\|xilinx:adm-pcie-7v3:1ddr:3.0-0\|cnn\_GOOD | Read | OCL Region | 1 | 1 | 113176 | 113177 | 0.00333333 | 4002.169262 | 4002.169266 |
| 4002.169319 | Kernel\_Read\|xilinx:adm-pcie-7v3:1ddr:3.0-0\|cnn\_GOOD | Read | OCL Region | 1 | 1 | 113193 | 113194 | 0.00333333 | 4002.169319 | 4002.169322 |
| 4002.169386 | Kernel\_Read\|xilinx:adm-pcie-7v3:1ddr:3.0-0\|cnn\_GOOD | Read | OCL Region | 1 | 1 | 113213 | 113214 | 0.00333333 | 4002.169386 | 4002.169389 |
| 4002.169459 | Kernel\_Read\|xilinx:adm-pcie-7v3:1ddr:3.0-0\|cnn\_GOOD | Read | OCL Region | 1 | 1 | 113235 | 113236 | 0.00333333 | 4002.169459 | 4002.169462 |
| 4002.169519 | Kernel\_Read\|xilinx:adm-pcie-7v3:1ddr:3.0-0\|cnn\_GOOD | Read | OCL Region | 1 | 1 | 113253 | 113254 | 0.00333333 | 4002.169519 | 4002.169522 |
| 4002.169586 | Kernel\_Read\|xilinx:adm-pcie-7v3:1ddr:3.0-0\|cnn\_GOOD | Read | OCL Region | 1 | 1 | 113273 | 113274 | 0.00333333 | 4002.169586 | 4002.169589 |
| 4002.169659 | Kernel\_Read\|xilinx:adm-pcie-7v3:1ddr:3.0-0\|cnn\_GOOD | Read | OCL Region | 1 | 1 | 113295 | 113296 | 0.00333333 | 4002.169659 | 4002.169662 |
| 4002.169719 | Kernel\_Read\|xilinx:adm-pcie-7v3:1ddr:3.0-0\|cnn\_GOOD | Read | OCL Region | 1 | 1 | 113313 | 113314 | 0.00333333 | 4002.169719 | 4002.169722 |
| 4002.169786 | Kernel\_Read\|xilinx:adm-pcie-7v3:1ddr:3.0-0\|cnn\_GOOD | Read | OCL Region | 1 | 1 | 113333 | 113334 | 0.00333333 | 4002.169786 | 4002.169789 |
| 4002.169859 | Kernel\_Read\|xilinx:adm-pcie-7v3:1ddr:3.0-0\|cnn\_GOOD | Read | OCL Region | 1 | 1 | 113355 | 113356 | 0.00333333 | 4002.169859 | 4002.169862 |
| 4002.169919 | Kernel\_Read\|xilinx:adm-pcie-7v3:1ddr:3.0-0\|cnn\_GOOD | Read | OCL Region | 1 | 1 | 113373 | 113374 | 0.00333333 | 4002.169919 | 4002.169922 |
| 4002.169986 | Kernel\_Read\|xilinx:adm-pcie-7v3:1ddr:3.0-0\|cnn\_GOOD | Read | OCL Region | 1 | 1 | 113393 | 113394 | 0.00333333 | 4002.169986 | 4002.169989 |
| 4002.170062 | Kernel\_Read\|xilinx:adm-pcie-7v3:1ddr:3.0-0\|cnn\_GOOD | Read | OCL Region | 1 | 1 | 113416 | 113417 | 0.00333333 | 4002.170062 | 4002.170066 |
| 4002.170119 | Kernel\_Read\|xilinx:adm-pcie-7v3:1ddr:3.0-0\|cnn\_GOOD | Read | OCL Region | 1 | 1 | 113433 | 113434 | 0.00333333 | 4002.170119 | 4002.170122 |
| 4002.170186 | Kernel\_Read\|xilinx:adm-pcie-7v3:1ddr:3.0-0\|cnn\_GOOD | Read | OCL Region | 1 | 1 | 113453 | 113454 | 0.00333333 | 4002.170186 | 4002.170189 |
| 4002.170259 | Kernel\_Read\|xilinx:adm-pcie-7v3:1ddr:3.0-0\|cnn\_GOOD | Read | OCL Region | 1 | 1 | 113475 | 113476 | 0.00333333 | 4002.170259 | 4002.170262 |
| 4002.170319 | Kernel\_Read\|xilinx:adm-pcie-7v3:1ddr:3.0-0\|cnn\_GOOD | Read | OCL Region | 1 | 1 | 113493 | 113494 | 0.00333333 | 4002.170319 | 4002.170322 |
| 4002.170386 | Kernel\_Read\|xilinx:adm-pcie-7v3:1ddr:3.0-0\|cnn\_GOOD | Read | OCL Region | 1 | 1 | 113513 | 113514 | 0.00333333 | 4002.170386 | 4002.170389 |
| 4002.170459 | Kernel\_Read\|xilinx:adm-pcie-7v3:1ddr:3.0-0\|cnn\_GOOD | Read | OCL Region | 1 | 1 | 113535 | 113536 | 0.00333333 | 4002.170459 | 4002.170462 |
| 4002.170519 | Kernel\_Read\|xilinx:adm-pcie-7v3:1ddr:3.0-0\|cnn\_GOOD | Read | OCL Region | 1 | 1 | 113553 | 113554 | 0.00333333 | 4002.170519 | 4002.170522 |
| 4002.170586 | Kernel\_Read\|xilinx:adm-pcie-7v3:1ddr:3.0-0\|cnn\_GOOD | Read | OCL Region | 1 | 1 | 113573 | 113574 | 0.00333333 | 4002.170586 | 4002.170589 |
| 4002.170659 | Kernel\_Read\|xilinx:adm-pcie-7v3:1ddr:3.0-0\|cnn\_GOOD | Read | OCL Region | 1 | 1 | 113595 | 113596 | 0.00333333 | 4002.170659 | 4002.170662 |
| 4002.170719 | Kernel\_Read\|xilinx:adm-pcie-7v3:1ddr:3.0-0\|cnn\_GOOD | Read | OCL Region | 1 | 1 | 113613 | 113614 | 0.00333333 | 4002.170719 | 4002.170722 |
| 4002.170786 | Kernel\_Read\|xilinx:adm-pcie-7v3:1ddr:3.0-0\|cnn\_GOOD | Read | OCL Region | 1 | 1 | 113633 | 113634 | 0.00333333 | 4002.170786 | 4002.170789 |
| 4002.170849 | Kernel\_Read\|xilinx:adm-pcie-7v3:1ddr:3.0-0\|cnn\_GOOD | Read | OCL Region | 1 | 1 | 113652 | 113653 | 0.00333333 | 4002.170849 | 4002.170852 |
| 4002.170919 | Kernel\_Read\|xilinx:adm-pcie-7v3:1ddr:3.0-0\|cnn\_GOOD | Read | OCL Region | 1 | 1 | 113673 | 113674 | 0.00333333 | 4002.170919 | 4002.170922 |
| 4002.170986 | Kernel\_Read\|xilinx:adm-pcie-7v3:1ddr:3.0-0\|cnn\_GOOD | Read | OCL Region | 1 | 1 | 113693 | 113694 | 0.00333333 | 4002.170986 | 4002.170989 |
| 4002.171059 | Kernel\_Read\|xilinx:adm-pcie-7v3:1ddr:3.0-0\|cnn\_GOOD | Read | OCL Region | 1 | 1 | 113715 | 113716 | 0.00333333 | 4002.171059 | 4002.171062 |
| 4002.171119 | Kernel\_Read\|xilinx:adm-pcie-7v3:1ddr:3.0-0\|cnn\_GOOD | Read | OCL Region | 1 | 1 | 113733 | 113734 | 0.00333333 | 4002.171119 | 4002.171122 |
| 4002.171186 | Kernel\_Read\|xilinx:adm-pcie-7v3:1ddr:3.0-0\|cnn\_GOOD | Read | OCL Region | 1 | 1 | 113753 | 113754 | 0.00333333 | 4002.171186 | 4002.171189 |
| 4002.171259 | Kernel\_Read\|xilinx:adm-pcie-7v3:1ddr:3.0-0\|cnn\_GOOD | Read | OCL Region | 1 | 1 | 113775 | 113776 | 0.00333333 | 4002.171259 | 4002.171262 |
| 4002.171319 | Kernel\_Read\|xilinx:adm-pcie-7v3:1ddr:3.0-0\|cnn\_GOOD | Read | OCL Region | 1 | 1 | 113793 | 113794 | 0.00333333 | 4002.171319 | 4002.171322 |
| 4002.171386 | Kernel\_Read\|xilinx:adm-pcie-7v3:1ddr:3.0-0\|cnn\_GOOD | Read | OCL Region | 1 | 1 | 113813 | 113814 | 0.00333333 | 4002.171386 | 4002.171389 |
| 4002.171459 | Kernel\_Read\|xilinx:adm-pcie-7v3:1ddr:3.0-0\|cnn\_GOOD | Read | OCL Region | 1 | 1 | 113835 | 113836 | 0.00333333 | 4002.171459 | 4002.171462 |
| 4002.171519 | Kernel\_Read\|xilinx:adm-pcie-7v3:1ddr:3.0-0\|cnn\_GOOD | Read | OCL Region | 1 | 1 | 113853 | 113854 | 0.00333333 | 4002.171519 | 4002.171522 |
| 4002.171586 | Kernel\_Read\|xilinx:adm-pcie-7v3:1ddr:3.0-0\|cnn\_GOOD | Read | OCL Region | 1 | 1 | 113873 | 113874 | 0.00333333 | 4002.171586 | 4002.171589 |
| 4002.171659 | Kernel\_Read\|xilinx:adm-pcie-7v3:1ddr:3.0-0\|cnn\_GOOD | Read | OCL Region | 1 | 1 | 113895 | 113896 | 0.00333333 | 4002.171659 | 4002.171662 |
| 4002.171719 | Kernel\_Read\|xilinx:adm-pcie-7v3:1ddr:3.0-0\|cnn\_GOOD | Read | OCL Region | 1 | 1 | 113913 | 113914 | 0.00333333 | 4002.171719 | 4002.171722 |
| 4002.171786 | Kernel\_Read\|xilinx:adm-pcie-7v3:1ddr:3.0-0\|cnn\_GOOD | Read | OCL Region | 1 | 1 | 113933 | 113934 | 0.00333333 | 4002.171786 | 4002.171789 |
| 4002.171859 | Kernel\_Read\|xilinx:adm-pcie-7v3:1ddr:3.0-0\|cnn\_GOOD | Read | OCL Region | 1 | 1 | 113955 | 113956 | 0.00333333 | 4002.171859 | 4002.171862 |
| 4002.171919 | Kernel\_Read\|xilinx:adm-pcie-7v3:1ddr:3.0-0\|cnn\_GOOD | Read | OCL Region | 1 | 1 | 113973 | 113974 | 0.00333333 | 4002.171919 | 4002.171922 |
| 4002.171986 | Kernel\_Read\|xilinx:adm-pcie-7v3:1ddr:3.0-0\|cnn\_GOOD | Read | OCL Region | 1 | 1 | 113993 | 113994 | 0.00333333 | 4002.171986 | 4002.171989 |
| 4002.172059 | Kernel\_Read\|xilinx:adm-pcie-7v3:1ddr:3.0-0\|cnn\_GOOD | Read | OCL Region | 1 | 1 | 114015 | 114016 | 0.00333333 | 4002.172059 | 4002.172062 |
| 4002.172119 | Kernel\_Read\|xilinx:adm-pcie-7v3:1ddr:3.0-0\|cnn\_GOOD | Read | OCL Region | 1 | 1 | 114033 | 114034 | 0.00333333 | 4002.172119 | 4002.172122 |
| 4002.172186 | Kernel\_Read\|xilinx:adm-pcie-7v3:1ddr:3.0-0\|cnn\_GOOD | Read | OCL Region | 1 | 1 | 114053 | 114054 | 0.00333333 | 4002.172186 | 4002.172189 |
| 4002.172259 | Kernel\_Read\|xilinx:adm-pcie-7v3:1ddr:3.0-0\|cnn\_GOOD | Read | OCL Region | 1 | 1 | 114075 | 114076 | 0.00333333 | 4002.172259 | 4002.172262 |
| 4002.172319 | Kernel\_Read\|xilinx:adm-pcie-7v3:1ddr:3.0-0\|cnn\_GOOD | Read | OCL Region | 1 | 1 | 114093 | 114094 | 0.00333333 | 4002.172319 | 4002.172322 |
| 4002.172386 | Kernel\_Read\|xilinx:adm-pcie-7v3:1ddr:3.0-0\|cnn\_GOOD | Read | OCL Region | 1 | 1 | 114113 | 114114 | 0.00333333 | 4002.172386 | 4002.172389 |
| 4002.172459 | Kernel\_Read\|xilinx:adm-pcie-7v3:1ddr:3.0-0\|cnn\_GOOD | Read | OCL Region | 1 | 1 | 114135 | 114136 | 0.00333333 | 4002.172459 | 4002.172462 |
| 4002.172536 | Kernel\_Read\|xilinx:adm-pcie-7v3:1ddr:3.0-0\|cnn\_GOOD | Read | OCL Region | 1 | 1 | 114158 | 114159 | 0.00333333 | 4002.172536 | 4002.172539 |
| 4002.172592 | Kernel\_Read\|xilinx:adm-pcie-7v3:1ddr:3.0-0\|cnn\_GOOD | Read | OCL Region | 1 | 1 | 114175 | 114176 | 0.00333333 | 4002.172592 | 4002.172596 |
| 4002.172659 | Kernel\_Read\|xilinx:adm-pcie-7v3:1ddr:3.0-0\|cnn\_GOOD | Read | OCL Region | 1 | 1 | 114195 | 114196 | 0.00333333 | 4002.172659 | 4002.172662 |
| 4002.172719 | Kernel\_Read\|xilinx:adm-pcie-7v3:1ddr:3.0-0\|cnn\_GOOD | Read | OCL Region | 1 | 1 | 114213 | 114214 | 0.00333333 | 4002.172719 | 4002.172722 |
| 4002.172786 | Kernel\_Read\|xilinx:adm-pcie-7v3:1ddr:3.0-0\|cnn\_GOOD | Read | OCL Region | 1 | 1 | 114233 | 114234 | 0.00333333 | 4002.172786 | 4002.172789 |
| 4002.172859 | Kernel\_Read\|xilinx:adm-pcie-7v3:1ddr:3.0-0\|cnn\_GOOD | Read | OCL Region | 1 | 1 | 114255 | 114256 | 0.00333333 | 4002.172859 | 4002.172862 |
| 4002.172919 | Kernel\_Read\|xilinx:adm-pcie-7v3:1ddr:3.0-0\|cnn\_GOOD | Read | OCL Region | 1 | 1 | 114273 | 114274 | 0.00333333 | 4002.172919 | 4002.172922 |
| 4002.172986 | Kernel\_Read\|xilinx:adm-pcie-7v3:1ddr:3.0-0\|cnn\_GOOD | Read | OCL Region | 1 | 1 | 114293 | 114294 | 0.00333333 | 4002.172986 | 4002.172989 |
| 4002.173059 | Kernel\_Read\|xilinx:adm-pcie-7v3:1ddr:3.0-0\|cnn\_GOOD | Read | OCL Region | 1 | 1 | 114315 | 114316 | 0.00333333 | 4002.173059 | 4002.173062 |
| 4002.173119 | Kernel\_Read\|xilinx:adm-pcie-7v3:1ddr:3.0-0\|cnn\_GOOD | Read | OCL Region | 1 | 1 | 114333 | 114334 | 0.00333333 | 4002.173119 | 4002.173122 |
| 4002.173186 | Kernel\_Read\|xilinx:adm-pcie-7v3:1ddr:3.0-0\|cnn\_GOOD | Read | OCL Region | 1 | 1 | 114353 | 114354 | 0.00333333 | 4002.173186 | 4002.173189 |
| 4002.173259 | Kernel\_Read\|xilinx:adm-pcie-7v3:1ddr:3.0-0\|cnn\_GOOD | Read | OCL Region | 1 | 1 | 114375 | 114376 | 0.00333333 | 4002.173259 | 4002.173262 |
| 4002.173326 | Kernel\_Read\|xilinx:adm-pcie-7v3:1ddr:3.0-0\|cnn\_GOOD | Read | OCL Region | 1 | 1 | 114395 | 114396 | 0.00333333 | 4002.173326 | 4002.173329 |
| 4002.173386 | Kernel\_Read\|xilinx:adm-pcie-7v3:1ddr:3.0-0\|cnn\_GOOD | Read | OCL Region | 1 | 1 | 114413 | 114414 | 0.00333333 | 4002.173386 | 4002.173389 |
| 4002.173459 | Kernel\_Read\|xilinx:adm-pcie-7v3:1ddr:3.0-0\|cnn\_GOOD | Read | OCL Region | 1 | 1 | 114435 | 114436 | 0.00333333 | 4002.173459 | 4002.173462 |
| 4002.173519 | Kernel\_Read\|xilinx:adm-pcie-7v3:1ddr:3.0-0\|cnn\_GOOD | Read | OCL Region | 1 | 1 | 114453 | 114454 | 0.00333333 | 4002.173519 | 4002.173522 |
| 4002.173586 | Kernel\_Read\|xilinx:adm-pcie-7v3:1ddr:3.0-0\|cnn\_GOOD | Read | OCL Region | 1 | 1 | 114473 | 114474 | 0.00333333 | 4002.173586 | 4002.173589 |
| 4002.173659 | Kernel\_Read\|xilinx:adm-pcie-7v3:1ddr:3.0-0\|cnn\_GOOD | Read | OCL Region | 1 | 1 | 114495 | 114496 | 0.00333333 | 4002.173659 | 4002.173662 |
| 4002.173719 | Kernel\_Read\|xilinx:adm-pcie-7v3:1ddr:3.0-0\|cnn\_GOOD | Read | OCL Region | 1 | 1 | 114513 | 114514 | 0.00333333 | 4002.173719 | 4002.173722 |
| 4002.173786 | Kernel\_Read\|xilinx:adm-pcie-7v3:1ddr:3.0-0\|cnn\_GOOD | Read | OCL Region | 1 | 1 | 114533 | 114534 | 0.00333333 | 4002.173786 | 4002.173789 |
| 4002.173859 | Kernel\_Read\|xilinx:adm-pcie-7v3:1ddr:3.0-0\|cnn\_GOOD | Read | OCL Region | 1 | 1 | 114555 | 114556 | 0.00333333 | 4002.173859 | 4002.173862 |
| 4002.173919 | Kernel\_Read\|xilinx:adm-pcie-7v3:1ddr:3.0-0\|cnn\_GOOD | Read | OCL Region | 1 | 1 | 114573 | 114574 | 0.00333333 | 4002.173919 | 4002.173922 |
| 4002.173986 | Kernel\_Read\|xilinx:adm-pcie-7v3:1ddr:3.0-0\|cnn\_GOOD | Read | OCL Region | 1 | 1 | 114593 | 114594 | 0.00333333 | 4002.173986 | 4002.173989 |
| 4002.174059 | Kernel\_Read\|xilinx:adm-pcie-7v3:1ddr:3.0-0\|cnn\_GOOD | Read | OCL Region | 1 | 1 | 114615 | 114616 | 0.00333333 | 4002.174059 | 4002.174062 |
| 4002.174119 | Kernel\_Read\|xilinx:adm-pcie-7v3:1ddr:3.0-0\|cnn\_GOOD | Read | OCL Region | 1 | 1 | 114633 | 114634 | 0.00333333 | 4002.174119 | 4002.174122 |
| 4002.174186 | Kernel\_Read\|xilinx:adm-pcie-7v3:1ddr:3.0-0\|cnn\_GOOD | Read | OCL Region | 1 | 1 | 114653 | 114654 | 0.00333333 | 4002.174186 | 4002.174189 |
| 4002.174259 | Kernel\_Read\|xilinx:adm-pcie-7v3:1ddr:3.0-0\|cnn\_GOOD | Read | OCL Region | 1 | 1 | 114675 | 114676 | 0.00333333 | 4002.174259 | 4002.174262 |
| 4002.174319 | Kernel\_Read\|xilinx:adm-pcie-7v3:1ddr:3.0-0\|cnn\_GOOD | Read | OCL Region | 1 | 1 | 114693 | 114694 | 0.00333333 | 4002.174319 | 4002.174322 |
| 4002.174386 | Kernel\_Read\|xilinx:adm-pcie-7v3:1ddr:3.0-0\|cnn\_GOOD | Read | OCL Region | 1 | 1 | 114713 | 114714 | 0.00333333 | 4002.174386 | 4002.174389 |
| 4002.174459 | Kernel\_Read\|xilinx:adm-pcie-7v3:1ddr:3.0-0\|cnn\_GOOD | Read | OCL Region | 1 | 1 | 114735 | 114736 | 0.00333333 | 4002.174459 | 4002.174462 |
| 4002.174519 | Kernel\_Read\|xilinx:adm-pcie-7v3:1ddr:3.0-0\|cnn\_GOOD | Read | OCL Region | 1 | 1 | 114753 | 114754 | 0.00333333 | 4002.174519 | 4002.174522 |
| 4002.174586 | Kernel\_Read\|xilinx:adm-pcie-7v3:1ddr:3.0-0\|cnn\_GOOD | Read | OCL Region | 1 | 1 | 114773 | 114774 | 0.00333333 | 4002.174586 | 4002.174589 |
| 4002.174659 | Kernel\_Read\|xilinx:adm-pcie-7v3:1ddr:3.0-0\|cnn\_GOOD | Read | OCL Region | 1 | 1 | 114795 | 114796 | 0.00333333 | 4002.174659 | 4002.174662 |
| 4002.174719 | Kernel\_Read\|xilinx:adm-pcie-7v3:1ddr:3.0-0\|cnn\_GOOD | Read | OCL Region | 1 | 1 | 114813 | 114814 | 0.00333333 | 4002.174719 | 4002.174722 |
| 4002.174786 | Kernel\_Read\|xilinx:adm-pcie-7v3:1ddr:3.0-0\|cnn\_GOOD | Read | OCL Region | 1 | 1 | 114833 | 114834 | 0.00333333 | 4002.174786 | 4002.174789 |
| 4002.174859 | Kernel\_Read\|xilinx:adm-pcie-7v3:1ddr:3.0-0\|cnn\_GOOD | Read | OCL Region | 1 | 1 | 114855 | 114856 | 0.00333333 | 4002.174859 | 4002.174862 |
| 4002.174919 | Kernel\_Read\|xilinx:adm-pcie-7v3:1ddr:3.0-0\|cnn\_GOOD | Read | OCL Region | 1 | 1 | 114873 | 114874 | 0.00333333 | 4002.174919 | 4002.174922 |
| 4002.174986 | Kernel\_Read\|xilinx:adm-pcie-7v3:1ddr:3.0-0\|cnn\_GOOD | Read | OCL Region | 1 | 1 | 114893 | 114894 | 0.00333333 | 4002.174986 | 4002.174989 |
| 4002.175059 | Kernel\_Read\|xilinx:adm-pcie-7v3:1ddr:3.0-0\|cnn\_GOOD | Read | OCL Region | 1 | 1 | 114915 | 114916 | 0.00333333 | 4002.175059 | 4002.175062 |
| 4002.175119 | Kernel\_Read\|xilinx:adm-pcie-7v3:1ddr:3.0-0\|cnn\_GOOD | Read | OCL Region | 1 | 1 | 114933 | 114934 | 0.00333333 | 4002.175119 | 4002.175122 |
| 4002.175396 | Kernel\_Read\|xilinx:adm-pcie-7v3:1ddr:3.0-0\|cnn\_GOOD | Read | OCL Region | 1 | 1 | 115016 | 115017 | 0.00333333 | 4002.175396 | 4002.175399 |
| 4002.175399 | Kernel\_Read\|xilinx:adm-pcie-7v3:1ddr:3.0-0\|cnn\_GOOD | Read | OCL Region | 1 | 1 | 115017 | 115018 | 0.00333333 | 4002.175399 | 4002.175402 |
| 4002.175409 | Kernel\_Read\|xilinx:adm-pcie-7v3:1ddr:3.0-0\|cnn\_GOOD | Read | OCL Region | 1 | 1 | 115020 | 115021 | 0.00333333 | 4002.175409 | 4002.175412 |
| 4002.175412 | Kernel\_Read\|xilinx:adm-pcie-7v3:1ddr:3.0-0\|cnn\_GOOD | Read | OCL Region | 1 | 1 | 115021 | 115022 | 0.00333333 | 4002.175412 | 4002.175416 |
| 4002.175469 | Kernel\_Read\|xilinx:adm-pcie-7v3:1ddr:3.0-0\|cnn\_GOOD | Read | OCL Region | 1 | 1 | 115038 | 115039 | 0.00333333 | 4002.175469 | 4002.175472 |
| 4002.175526 | Kernel\_Read\|xilinx:adm-pcie-7v3:1ddr:3.0-0\|cnn\_GOOD | Read | OCL Region | 1 | 1 | 115055 | 115056 | 0.00333333 | 4002.175526 | 4002.175529 |
| 4002.175586 | Kernel\_Read\|xilinx:adm-pcie-7v3:1ddr:3.0-0\|cnn\_GOOD | Read | OCL Region | 1 | 1 | 115073 | 115074 | 0.00333333 | 4002.175586 | 4002.175589 |
| 4002.175659 | Kernel\_Read\|xilinx:adm-pcie-7v3:1ddr:3.0-0\|cnn\_GOOD | Read | OCL Region | 1 | 1 | 115095 | 115096 | 0.00333333 | 4002.175659 | 4002.175662 |
| 4002.175719 | Kernel\_Read\|xilinx:adm-pcie-7v3:1ddr:3.0-0\|cnn\_GOOD | Read | OCL Region | 1 | 1 | 115113 | 115114 | 0.00333333 | 4002.175719 | 4002.175722 |
| 4002.175786 | Kernel\_Read\|xilinx:adm-pcie-7v3:1ddr:3.0-0\|cnn\_GOOD | Read | OCL Region | 1 | 1 | 115133 | 115134 | 0.00333333 | 4002.175786 | 4002.175789 |
| 4002.175859 | Kernel\_Read\|xilinx:adm-pcie-7v3:1ddr:3.0-0\|cnn\_GOOD | Read | OCL Region | 1 | 1 | 115155 | 115156 | 0.00333333 | 4002.175859 | 4002.175862 |
| 4002.175919 | Kernel\_Read\|xilinx:adm-pcie-7v3:1ddr:3.0-0\|cnn\_GOOD | Read | OCL Region | 1 | 1 | 115173 | 115174 | 0.00333333 | 4002.175919 | 4002.175922 |
| 4002.175986 | Kernel\_Read\|xilinx:adm-pcie-7v3:1ddr:3.0-0\|cnn\_GOOD | Read | OCL Region | 1 | 1 | 115193 | 115194 | 0.00333333 | 4002.175986 | 4002.175989 |
| 4002.176059 | Kernel\_Read\|xilinx:adm-pcie-7v3:1ddr:3.0-0\|cnn\_GOOD | Read | OCL Region | 1 | 1 | 115215 | 115216 | 0.00333333 | 4002.176059 | 4002.176062 |
| 4002.176132 | Kernel\_Read\|xilinx:adm-pcie-7v3:1ddr:3.0-0\|cnn\_GOOD | Read | OCL Region | 1 | 1 | 115237 | 115238 | 0.00333333 | 4002.176132 | 4002.176136 |
| 4002.176186 | Kernel\_Read\|xilinx:adm-pcie-7v3:1ddr:3.0-0\|cnn\_GOOD | Read | OCL Region | 1 | 1 | 115253 | 115254 | 0.00333333 | 4002.176186 | 4002.176189 |
| 4002.176259 | Kernel\_Read\|xilinx:adm-pcie-7v3:1ddr:3.0-0\|cnn\_GOOD | Read | OCL Region | 1 | 1 | 115275 | 115276 | 0.00333333 | 4002.176259 | 4002.176262 |
| 4002.176319 | Kernel\_Read\|xilinx:adm-pcie-7v3:1ddr:3.0-0\|cnn\_GOOD | Read | OCL Region | 1 | 1 | 115293 | 115294 | 0.00333333 | 4002.176319 | 4002.176322 |
| 4002.176386 | Kernel\_Read\|xilinx:adm-pcie-7v3:1ddr:3.0-0\|cnn\_GOOD | Read | OCL Region | 1 | 1 | 115313 | 115314 | 0.00333333 | 4002.176386 | 4002.176389 |
| 4002.176459 | Kernel\_Read\|xilinx:adm-pcie-7v3:1ddr:3.0-0\|cnn\_GOOD | Read | OCL Region | 1 | 1 | 115335 | 115336 | 0.00333333 | 4002.176459 | 4002.176462 |
| 4002.176519 | Kernel\_Read\|xilinx:adm-pcie-7v3:1ddr:3.0-0\|cnn\_GOOD | Read | OCL Region | 1 | 1 | 115353 | 115354 | 0.00333333 | 4002.176519 | 4002.176522 |
| 4002.176586 | Kernel\_Read\|xilinx:adm-pcie-7v3:1ddr:3.0-0\|cnn\_GOOD | Read | OCL Region | 1 | 1 | 115373 | 115374 | 0.00333333 | 4002.176586 | 4002.176589 |
| 4002.176659 | Kernel\_Read\|xilinx:adm-pcie-7v3:1ddr:3.0-0\|cnn\_GOOD | Read | OCL Region | 1 | 1 | 115395 | 115396 | 0.00333333 | 4002.176659 | 4002.176662 |
| 4002.176719 | Kernel\_Read\|xilinx:adm-pcie-7v3:1ddr:3.0-0\|cnn\_GOOD | Read | OCL Region | 1 | 1 | 115413 | 115414 | 0.00333333 | 4002.176719 | 4002.176722 |
| 4002.176786 | Kernel\_Read\|xilinx:adm-pcie-7v3:1ddr:3.0-0\|cnn\_GOOD | Read | OCL Region | 1 | 1 | 115433 | 115434 | 0.00333333 | 4002.176786 | 4002.176789 |
| 4002.176859 | Kernel\_Read\|xilinx:adm-pcie-7v3:1ddr:3.0-0\|cnn\_GOOD | Read | OCL Region | 1 | 1 | 115455 | 115456 | 0.00333333 | 4002.176859 | 4002.176862 |
| 4002.176919 | Kernel\_Read\|xilinx:adm-pcie-7v3:1ddr:3.0-0\|cnn\_GOOD | Read | OCL Region | 1 | 1 | 115473 | 115474 | 0.00333333 | 4002.176919 | 4002.176922 |
| 4002.176992 | Kernel\_Read\|xilinx:adm-pcie-7v3:1ddr:3.0-0\|cnn\_GOOD | Read | OCL Region | 1 | 1 | 115495 | 115496 | 0.00333333 | 4002.176992 | 4002.176996 |
| 4002.177059 | Kernel\_Read\|xilinx:adm-pcie-7v3:1ddr:3.0-0\|cnn\_GOOD | Read | OCL Region | 1 | 1 | 115515 | 115516 | 0.00333333 | 4002.177059 | 4002.177062 |
| 4002.177119 | Kernel\_Read\|xilinx:adm-pcie-7v3:1ddr:3.0-0\|cnn\_GOOD | Read | OCL Region | 1 | 1 | 115533 | 115534 | 0.00333333 | 4002.177119 | 4002.177122 |
| 4002.177186 | Kernel\_Read\|xilinx:adm-pcie-7v3:1ddr:3.0-0\|cnn\_GOOD | Read | OCL Region | 1 | 1 | 115553 | 115554 | 0.00333333 | 4002.177186 | 4002.177189 |
| 4002.177259 | Kernel\_Read\|xilinx:adm-pcie-7v3:1ddr:3.0-0\|cnn\_GOOD | Read | OCL Region | 1 | 1 | 115575 | 115576 | 0.00333333 | 4002.177259 | 4002.177262 |
| 4002.177319 | Kernel\_Read\|xilinx:adm-pcie-7v3:1ddr:3.0-0\|cnn\_GOOD | Read | OCL Region | 1 | 1 | 115593 | 115594 | 0.00333333 | 4002.177319 | 4002.177322 |
| 4002.177386 | Kernel\_Read\|xilinx:adm-pcie-7v3:1ddr:3.0-0\|cnn\_GOOD | Read | OCL Region | 1 | 1 | 115613 | 115614 | 0.00333333 | 4002.177386 | 4002.177389 |
| 4002.177459 | Kernel\_Read\|xilinx:adm-pcie-7v3:1ddr:3.0-0\|cnn\_GOOD | Read | OCL Region | 1 | 1 | 115635 | 115636 | 0.00333333 | 4002.177459 | 4002.177462 |
| 4002.177519 | Kernel\_Read\|xilinx:adm-pcie-7v3:1ddr:3.0-0\|cnn\_GOOD | Read | OCL Region | 1 | 1 | 115653 | 115654 | 0.00333333 | 4002.177519 | 4002.177522 |
| 4002.177586 | Kernel\_Read\|xilinx:adm-pcie-7v3:1ddr:3.0-0\|cnn\_GOOD | Read | OCL Region | 1 | 1 | 115673 | 115674 | 0.00333333 | 4002.177586 | 4002.177589 |
| 4002.177659 | Kernel\_Read\|xilinx:adm-pcie-7v3:1ddr:3.0-0\|cnn\_GOOD | Read | OCL Region | 1 | 1 | 115695 | 115696 | 0.00333333 | 4002.177659 | 4002.177662 |
| 4002.177719 | Kernel\_Read\|xilinx:adm-pcie-7v3:1ddr:3.0-0\|cnn\_GOOD | Read | OCL Region | 1 | 1 | 115713 | 115714 | 0.00333333 | 4002.177719 | 4002.177722 |
| 4002.177786 | Kernel\_Read\|xilinx:adm-pcie-7v3:1ddr:3.0-0\|cnn\_GOOD | Read | OCL Region | 1 | 1 | 115733 | 115734 | 0.00333333 | 4002.177786 | 4002.177789 |
| 4002.177859 | Kernel\_Read\|xilinx:adm-pcie-7v3:1ddr:3.0-0\|cnn\_GOOD | Read | OCL Region | 1 | 1 | 115755 | 115756 | 0.00333333 | 4002.177859 | 4002.177862 |
| 4002.177919 | Kernel\_Read\|xilinx:adm-pcie-7v3:1ddr:3.0-0\|cnn\_GOOD | Read | OCL Region | 1 | 1 | 115773 | 115774 | 0.00333333 | 4002.177919 | 4002.177922 |
| 4002.177986 | Kernel\_Read\|xilinx:adm-pcie-7v3:1ddr:3.0-0\|cnn\_GOOD | Read | OCL Region | 1 | 1 | 115793 | 115794 | 0.00333333 | 4002.177986 | 4002.177989 |
| 4002.178059 | Kernel\_Read\|xilinx:adm-pcie-7v3:1ddr:3.0-0\|cnn\_GOOD | Read | OCL Region | 1 | 1 | 115815 | 115816 | 0.00333333 | 4002.178059 | 4002.178062 |
| 4002.178119 | Kernel\_Read\|xilinx:adm-pcie-7v3:1ddr:3.0-0\|cnn\_GOOD | Read | OCL Region | 1 | 1 | 115833 | 115834 | 0.00333333 | 4002.178119 | 4002.178122 |
| 4002.178186 | Kernel\_Read\|xilinx:adm-pcie-7v3:1ddr:3.0-0\|cnn\_GOOD | Read | OCL Region | 1 | 1 | 115853 | 115854 | 0.00333333 | 4002.178186 | 4002.178189 |
| 4002.178259 | Kernel\_Read\|xilinx:adm-pcie-7v3:1ddr:3.0-0\|cnn\_GOOD | Read | OCL Region | 1 | 1 | 115875 | 115876 | 0.00333333 | 4002.178259 | 4002.178262 |
| 4002.178319 | Kernel\_Read\|xilinx:adm-pcie-7v3:1ddr:3.0-0\|cnn\_GOOD | Read | OCL Region | 1 | 1 | 115893 | 115894 | 0.00333333 | 4002.178319 | 4002.178322 |
| 4002.178386 | Kernel\_Read\|xilinx:adm-pcie-7v3:1ddr:3.0-0\|cnn\_GOOD | Read | OCL Region | 1 | 1 | 115913 | 115914 | 0.00333333 | 4002.178386 | 4002.178389 |
| 4002.178459 | Kernel\_Read\|xilinx:adm-pcie-7v3:1ddr:3.0-0\|cnn\_GOOD | Read | OCL Region | 1 | 1 | 115935 | 115936 | 0.00333333 | 4002.178459 | 4002.178462 |
| 4002.178519 | Kernel\_Read\|xilinx:adm-pcie-7v3:1ddr:3.0-0\|cnn\_GOOD | Read | OCL Region | 1 | 1 | 115953 | 115954 | 0.00333333 | 4002.178519 | 4002.178522 |
| 4002.178586 | Kernel\_Read\|xilinx:adm-pcie-7v3:1ddr:3.0-0\|cnn\_GOOD | Read | OCL Region | 1 | 1 | 115973 | 115974 | 0.00333333 | 4002.178586 | 4002.178589 |
| 4002.178659 | Kernel\_Read\|xilinx:adm-pcie-7v3:1ddr:3.0-0\|cnn\_GOOD | Read | OCL Region | 1 | 1 | 115995 | 115996 | 0.00333333 | 4002.178659 | 4002.178662 |
| 4002.178719 | Kernel\_Read\|xilinx:adm-pcie-7v3:1ddr:3.0-0\|cnn\_GOOD | Read | OCL Region | 1 | 1 | 116013 | 116014 | 0.00333333 | 4002.178719 | 4002.178722 |
| 4002.178786 | Kernel\_Read\|xilinx:adm-pcie-7v3:1ddr:3.0-0\|cnn\_GOOD | Read | OCL Region | 1 | 1 | 116033 | 116034 | 0.00333333 | 4002.178786 | 4002.178789 |
| 4002.178859 | Kernel\_Read\|xilinx:adm-pcie-7v3:1ddr:3.0-0\|cnn\_GOOD | Read | OCL Region | 1 | 1 | 116055 | 116056 | 0.00333333 | 4002.178859 | 4002.178862 |
| 4002.178919 | Kernel\_Read\|xilinx:adm-pcie-7v3:1ddr:3.0-0\|cnn\_GOOD | Read | OCL Region | 1 | 1 | 116073 | 116074 | 0.00333333 | 4002.178919 | 4002.178922 |
| 4002.178986 | Kernel\_Read\|xilinx:adm-pcie-7v3:1ddr:3.0-0\|cnn\_GOOD | Read | OCL Region | 1 | 1 | 116093 | 116094 | 0.00333333 | 4002.178986 | 4002.178989 |
| 4002.179059 | Kernel\_Read\|xilinx:adm-pcie-7v3:1ddr:3.0-0\|cnn\_GOOD | Read | OCL Region | 1 | 1 | 116115 | 116116 | 0.00333333 | 4002.179059 | 4002.179062 |
| 4002.179119 | Kernel\_Read\|xilinx:adm-pcie-7v3:1ddr:3.0-0\|cnn\_GOOD | Read | OCL Region | 1 | 1 | 116133 | 116134 | 0.00333333 | 4002.179119 | 4002.179122 |
| 4002.179186 | Kernel\_Read\|xilinx:adm-pcie-7v3:1ddr:3.0-0\|cnn\_GOOD | Read | OCL Region | 1 | 1 | 116153 | 116154 | 0.00333333 | 4002.179186 | 4002.179189 |
| 4002.179259 | Kernel\_Read\|xilinx:adm-pcie-7v3:1ddr:3.0-0\|cnn\_GOOD | Read | OCL Region | 1 | 1 | 116175 | 116176 | 0.00333333 | 4002.179259 | 4002.179262 |
| 4002.179319 | Kernel\_Read\|xilinx:adm-pcie-7v3:1ddr:3.0-0\|cnn\_GOOD | Read | OCL Region | 1 | 1 | 116193 | 116194 | 0.00333333 | 4002.179319 | 4002.179322 |
| 4002.179386 | Kernel\_Read\|xilinx:adm-pcie-7v3:1ddr:3.0-0\|cnn\_GOOD | Read | OCL Region | 1 | 1 | 116213 | 116214 | 0.00333333 | 4002.179386 | 4002.179389 |
| 4002.179466 | Kernel\_Read\|xilinx:adm-pcie-7v3:1ddr:3.0-0\|cnn\_GOOD | Read | OCL Region | 1 | 1 | 116237 | 116238 | 0.00333333 | 4002.179466 | 4002.179469 |
| 4002.179519 | Kernel\_Read\|xilinx:adm-pcie-7v3:1ddr:3.0-0\|cnn\_GOOD | Read | OCL Region | 1 | 1 | 116253 | 116254 | 0.00333333 | 4002.179519 | 4002.179522 |
| 4002.179586 | Kernel\_Read\|xilinx:adm-pcie-7v3:1ddr:3.0-0\|cnn\_GOOD | Read | OCL Region | 1 | 1 | 116273 | 116274 | 0.00333333 | 4002.179586 | 4002.179589 |
| 4002.179659 | Kernel\_Read\|xilinx:adm-pcie-7v3:1ddr:3.0-0\|cnn\_GOOD | Read | OCL Region | 1 | 1 | 116295 | 116296 | 0.00333333 | 4002.179659 | 4002.179662 |
| 4002.179719 | Kernel\_Read\|xilinx:adm-pcie-7v3:1ddr:3.0-0\|cnn\_GOOD | Read | OCL Region | 1 | 1 | 116313 | 116314 | 0.00333333 | 4002.179719 | 4002.179722 |
| 4002.179786 | Kernel\_Read\|xilinx:adm-pcie-7v3:1ddr:3.0-0\|cnn\_GOOD | Read | OCL Region | 1 | 1 | 116333 | 116334 | 0.00333333 | 4002.179786 | 4002.179789 |
| 4002.179859 | Kernel\_Read\|xilinx:adm-pcie-7v3:1ddr:3.0-0\|cnn\_GOOD | Read | OCL Region | 1 | 1 | 116355 | 116356 | 0.00333333 | 4002.179859 | 4002.179862 |
| 4002.179919 | Kernel\_Read\|xilinx:adm-pcie-7v3:1ddr:3.0-0\|cnn\_GOOD | Read | OCL Region | 1 | 1 | 116373 | 116374 | 0.00333333 | 4002.179919 | 4002.179922 |
| 4002.179986 | Kernel\_Read\|xilinx:adm-pcie-7v3:1ddr:3.0-0\|cnn\_GOOD | Read | OCL Region | 1 | 1 | 116393 | 116394 | 0.00333333 | 4002.179986 | 4002.179989 |
| 4002.180059 | Kernel\_Read\|xilinx:adm-pcie-7v3:1ddr:3.0-0\|cnn\_GOOD | Read | OCL Region | 1 | 1 | 116415 | 116416 | 0.00333333 | 4002.180059 | 4002.180062 |
| 4002.180119 | Kernel\_Read\|xilinx:adm-pcie-7v3:1ddr:3.0-0\|cnn\_GOOD | Read | OCL Region | 1 | 1 | 116433 | 116434 | 0.00333333 | 4002.180119 | 4002.180122 |
| 4002.180186 | Kernel\_Read\|xilinx:adm-pcie-7v3:1ddr:3.0-0\|cnn\_GOOD | Read | OCL Region | 1 | 1 | 116453 | 116454 | 0.00333333 | 4002.180186 | 4002.180189 |
| 4002.180299 | Kernel\_Read\|xilinx:adm-pcie-7v3:1ddr:3.0-0\|cnn\_GOOD | Read | OCL Region | 1 | 1 | 116487 | 116488 | 0.00333333 | 4002.180299 | 4002.180302 |
| 4002.180309 | Kernel\_Read\|xilinx:adm-pcie-7v3:1ddr:3.0-0\|cnn\_GOOD | Read | OCL Region | 1 | 1 | 116490 | 116491 | 0.00333333 | 4002.180309 | 4002.180312 |
| 4002.180386 | Kernel\_Read\|xilinx:adm-pcie-7v3:1ddr:3.0-0\|cnn\_GOOD | Read | OCL Region | 1 | 1 | 116513 | 116514 | 0.00333333 | 4002.180386 | 4002.180389 |
| 4002.180459 | Kernel\_Read\|xilinx:adm-pcie-7v3:1ddr:3.0-0\|cnn\_GOOD | Read | OCL Region | 1 | 1 | 116535 | 116536 | 0.00333333 | 4002.180459 | 4002.180462 |
| 4002.180519 | Kernel\_Read\|xilinx:adm-pcie-7v3:1ddr:3.0-0\|cnn\_GOOD | Read | OCL Region | 1 | 1 | 116553 | 116554 | 0.00333333 | 4002.180519 | 4002.180522 |
| 4002.180586 | Kernel\_Read\|xilinx:adm-pcie-7v3:1ddr:3.0-0\|cnn\_GOOD | Read | OCL Region | 1 | 1 | 116573 | 116574 | 0.00333333 | 4002.180586 | 4002.180589 |
| 4002.180659 | Kernel\_Read\|xilinx:adm-pcie-7v3:1ddr:3.0-0\|cnn\_GOOD | Read | OCL Region | 1 | 1 | 116595 | 116596 | 0.00333333 | 4002.180659 | 4002.180662 |
| 4002.180719 | Kernel\_Read\|xilinx:adm-pcie-7v3:1ddr:3.0-0\|cnn\_GOOD | Read | OCL Region | 1 | 1 | 116613 | 116614 | 0.00333333 | 4002.180719 | 4002.180722 |
| 4002.180786 | Kernel\_Read\|xilinx:adm-pcie-7v3:1ddr:3.0-0\|cnn\_GOOD | Read | OCL Region | 1 | 1 | 116633 | 116634 | 0.00333333 | 4002.180786 | 4002.180789 |
| 4002.180859 | Kernel\_Read\|xilinx:adm-pcie-7v3:1ddr:3.0-0\|cnn\_GOOD | Read | OCL Region | 1 | 1 | 116655 | 116656 | 0.00333333 | 4002.180859 | 4002.180862 |
| 4002.180919 | Kernel\_Read\|xilinx:adm-pcie-7v3:1ddr:3.0-0\|cnn\_GOOD | Read | OCL Region | 1 | 1 | 116673 | 116674 | 0.00333333 | 4002.180919 | 4002.180922 |
| 4002.180986 | Kernel\_Read\|xilinx:adm-pcie-7v3:1ddr:3.0-0\|cnn\_GOOD | Read | OCL Region | 1 | 1 | 116693 | 116694 | 0.00333333 | 4002.180986 | 4002.180989 |
| 4002.181059 | Kernel\_Read\|xilinx:adm-pcie-7v3:1ddr:3.0-0\|cnn\_GOOD | Read | OCL Region | 1 | 1 | 116715 | 116716 | 0.00333333 | 4002.181059 | 4002.181062 |
| 4002.181119 | Kernel\_Read\|xilinx:adm-pcie-7v3:1ddr:3.0-0\|cnn\_GOOD | Read | OCL Region | 1 | 1 | 116733 | 116734 | 0.00333333 | 4002.181119 | 4002.181122 |
| 4002.181186 | Kernel\_Read\|xilinx:adm-pcie-7v3:1ddr:3.0-0\|cnn\_GOOD | Read | OCL Region | 1 | 1 | 116753 | 116754 | 0.00333333 | 4002.181186 | 4002.181189 |
| 4002.181259 | Kernel\_Read\|xilinx:adm-pcie-7v3:1ddr:3.0-0\|cnn\_GOOD | Read | OCL Region | 1 | 1 | 116775 | 116776 | 0.00333333 | 4002.181259 | 4002.181262 |
| 4002.181319 | Kernel\_Read\|xilinx:adm-pcie-7v3:1ddr:3.0-0\|cnn\_GOOD | Read | OCL Region | 1 | 1 | 116793 | 116794 | 0.00333333 | 4002.181319 | 4002.181322 |
| 4002.181386 | Kernel\_Read\|xilinx:adm-pcie-7v3:1ddr:3.0-0\|cnn\_GOOD | Read | OCL Region | 1 | 1 | 116813 | 116814 | 0.00333333 | 4002.181386 | 4002.181389 |
| 4002.181662 | Kernel\_Read\|xilinx:adm-pcie-7v3:1ddr:3.0-0\|cnn\_GOOD | Read | OCL Region | 1 | 1 | 116896 | 116897 | 0.00333333 | 4002.181662 | 4002.181666 |
| 4002.181666 | Kernel\_Read\|xilinx:adm-pcie-7v3:1ddr:3.0-0\|cnn\_GOOD | Read | OCL Region | 1 | 1 | 116897 | 116898 | 0.00333333 | 4002.181666 | 4002.181669 |
| 4002.181669 | Kernel\_Read\|xilinx:adm-pcie-7v3:1ddr:3.0-0\|cnn\_GOOD | Read | OCL Region | 1 | 1 | 116898 | 116899 | 0.00333333 | 4002.181669 | 4002.181672 |
| 4002.181676 | Kernel\_Read\|xilinx:adm-pcie-7v3:1ddr:3.0-0\|cnn\_GOOD | Read | OCL Region | 1 | 1 | 116900 | 116901 | 0.00333333 | 4002.181676 | 4002.181679 |
| 4002.181736 | Kernel\_Read\|xilinx:adm-pcie-7v3:1ddr:3.0-0\|cnn\_GOOD | Read | OCL Region | 1 | 1 | 116918 | 116919 | 0.00333333 | 4002.181736 | 4002.181739 |
| 4002.181792 | Kernel\_Read\|xilinx:adm-pcie-7v3:1ddr:3.0-0\|cnn\_GOOD | Read | OCL Region | 1 | 1 | 116935 | 116936 | 0.00333333 | 4002.181792 | 4002.181796 |
| 4002.181859 | Kernel\_Read\|xilinx:adm-pcie-7v3:1ddr:3.0-0\|cnn\_GOOD | Read | OCL Region | 1 | 1 | 116955 | 116956 | 0.00333333 | 4002.181859 | 4002.181862 |
| 4002.181919 | Kernel\_Read\|xilinx:adm-pcie-7v3:1ddr:3.0-0\|cnn\_GOOD | Read | OCL Region | 1 | 1 | 116973 | 116974 | 0.00333333 | 4002.181919 | 4002.181922 |
| 4002.181986 | Kernel\_Read\|xilinx:adm-pcie-7v3:1ddr:3.0-0\|cnn\_GOOD | Read | OCL Region | 1 | 1 | 116993 | 116994 | 0.00333333 | 4002.181986 | 4002.181989 |
| 4002.182059 | Kernel\_Read\|xilinx:adm-pcie-7v3:1ddr:3.0-0\|cnn\_GOOD | Read | OCL Region | 1 | 1 | 117015 | 117016 | 0.00333333 | 4002.182059 | 4002.182062 |
| 4002.182119 | Kernel\_Read\|xilinx:adm-pcie-7v3:1ddr:3.0-0\|cnn\_GOOD | Read | OCL Region | 1 | 1 | 117033 | 117034 | 0.00333333 | 4002.182119 | 4002.182122 |
| 4002.182186 | Kernel\_Read\|xilinx:adm-pcie-7v3:1ddr:3.0-0\|cnn\_GOOD | Read | OCL Region | 1 | 1 | 117053 | 117054 | 0.00333333 | 4002.182186 | 4002.182189 |
| 4002.182259 | Kernel\_Read\|xilinx:adm-pcie-7v3:1ddr:3.0-0\|cnn\_GOOD | Read | OCL Region | 1 | 1 | 117075 | 117076 | 0.00333333 | 4002.182259 | 4002.182262 |
| 4002.182319 | Kernel\_Read\|xilinx:adm-pcie-7v3:1ddr:3.0-0\|cnn\_GOOD | Read | OCL Region | 1 | 1 | 117093 | 117094 | 0.00333333 | 4002.182319 | 4002.182322 |
| 4002.182386 | Kernel\_Read\|xilinx:adm-pcie-7v3:1ddr:3.0-0\|cnn\_GOOD | Read | OCL Region | 1 | 1 | 117113 | 117114 | 0.00333333 | 4002.182386 | 4002.182389 |
| 4002.182459 | Kernel\_Read\|xilinx:adm-pcie-7v3:1ddr:3.0-0\|cnn\_GOOD | Read | OCL Region | 1 | 1 | 117135 | 117136 | 0.00333333 | 4002.182459 | 4002.182462 |
| 4002.182519 | Kernel\_Read\|xilinx:adm-pcie-7v3:1ddr:3.0-0\|cnn\_GOOD | Read | OCL Region | 1 | 1 | 117153 | 117154 | 0.00333333 | 4002.182519 | 4002.182522 |
| 4002.182586 | Kernel\_Read\|xilinx:adm-pcie-7v3:1ddr:3.0-0\|cnn\_GOOD | Read | OCL Region | 1 | 1 | 117173 | 117174 | 0.00333333 | 4002.182586 | 4002.182589 |
| 4002.182659 | Kernel\_Read\|xilinx:adm-pcie-7v3:1ddr:3.0-0\|cnn\_GOOD | Read | OCL Region | 1 | 1 | 117195 | 117196 | 0.00333333 | 4002.182659 | 4002.182662 |
| 4002.182719 | Kernel\_Read\|xilinx:adm-pcie-7v3:1ddr:3.0-0\|cnn\_GOOD | Read | OCL Region | 1 | 1 | 117213 | 117214 | 0.00333333 | 4002.182719 | 4002.182722 |
| 4002.182786 | Kernel\_Read\|xilinx:adm-pcie-7v3:1ddr:3.0-0\|cnn\_GOOD | Read | OCL Region | 1 | 1 | 117233 | 117234 | 0.00333333 | 4002.182786 | 4002.182789 |
| 4002.182859 | Kernel\_Read\|xilinx:adm-pcie-7v3:1ddr:3.0-0\|cnn\_GOOD | Read | OCL Region | 1 | 1 | 117255 | 117256 | 0.00333333 | 4002.182859 | 4002.182862 |
| 4002.182919 | Kernel\_Read\|xilinx:adm-pcie-7v3:1ddr:3.0-0\|cnn\_GOOD | Read | OCL Region | 1 | 1 | 117273 | 117274 | 0.00333333 | 4002.182919 | 4002.182922 |
| 4002.182986 | Kernel\_Read\|xilinx:adm-pcie-7v3:1ddr:3.0-0\|cnn\_GOOD | Read | OCL Region | 1 | 1 | 117293 | 117294 | 0.00333333 | 4002.182986 | 4002.182989 |
| 4002.183059 | Kernel\_Read\|xilinx:adm-pcie-7v3:1ddr:3.0-0\|cnn\_GOOD | Read | OCL Region | 1 | 1 | 117315 | 117316 | 0.00333333 | 4002.183059 | 4002.183062 |
| 4002.183119 | Kernel\_Read\|xilinx:adm-pcie-7v3:1ddr:3.0-0\|cnn\_GOOD | Read | OCL Region | 1 | 1 | 117333 | 117334 | 0.00333333 | 4002.183119 | 4002.183122 |
| 4002.183186 | Kernel\_Read\|xilinx:adm-pcie-7v3:1ddr:3.0-0\|cnn\_GOOD | Read | OCL Region | 1 | 1 | 117353 | 117354 | 0.00333333 | 4002.183186 | 4002.183189 |
| 4002.183262 | Kernel\_Read\|xilinx:adm-pcie-7v3:1ddr:3.0-0\|cnn\_GOOD | Read | OCL Region | 1 | 1 | 117376 | 117377 | 0.00333333 | 4002.183262 | 4002.183266 |
| 4002.183319 | Kernel\_Read\|xilinx:adm-pcie-7v3:1ddr:3.0-0\|cnn\_GOOD | Read | OCL Region | 1 | 1 | 117393 | 117394 | 0.00333333 | 4002.183319 | 4002.183322 |
| 4002.183386 | Kernel\_Read\|xilinx:adm-pcie-7v3:1ddr:3.0-0\|cnn\_GOOD | Read | OCL Region | 1 | 1 | 117413 | 117414 | 0.00333333 | 4002.183386 | 4002.183389 |
| 4002.183459 | Kernel\_Read\|xilinx:adm-pcie-7v3:1ddr:3.0-0\|cnn\_GOOD | Read | OCL Region | 1 | 1 | 117435 | 117436 | 0.00333333 | 4002.183459 | 4002.183462 |
| 4002.183519 | Kernel\_Read\|xilinx:adm-pcie-7v3:1ddr:3.0-0\|cnn\_GOOD | Read | OCL Region | 1 | 1 | 117453 | 117454 | 0.00333333 | 4002.183519 | 4002.183522 |
| 4002.183586 | Kernel\_Read\|xilinx:adm-pcie-7v3:1ddr:3.0-0\|cnn\_GOOD | Read | OCL Region | 1 | 1 | 117473 | 117474 | 0.00333333 | 4002.183586 | 4002.183589 |
| 4002.183659 | Kernel\_Read\|xilinx:adm-pcie-7v3:1ddr:3.0-0\|cnn\_GOOD | Read | OCL Region | 1 | 1 | 117495 | 117496 | 0.00333333 | 4002.183659 | 4002.183662 |
| 4002.183719 | Kernel\_Read\|xilinx:adm-pcie-7v3:1ddr:3.0-0\|cnn\_GOOD | Read | OCL Region | 1 | 1 | 117513 | 117514 | 0.00333333 | 4002.183719 | 4002.183722 |
| 4002.183786 | Kernel\_Read\|xilinx:adm-pcie-7v3:1ddr:3.0-0\|cnn\_GOOD | Read | OCL Region | 1 | 1 | 117533 | 117534 | 0.00333333 | 4002.183786 | 4002.183789 |
| 4002.183859 | Kernel\_Read\|xilinx:adm-pcie-7v3:1ddr:3.0-0\|cnn\_GOOD | Read | OCL Region | 1 | 1 | 117555 | 117556 | 0.00333333 | 4002.183859 | 4002.183862 |
| 4002.183919 | Kernel\_Read\|xilinx:adm-pcie-7v3:1ddr:3.0-0\|cnn\_GOOD | Read | OCL Region | 1 | 1 | 117573 | 117574 | 0.00333333 | 4002.183919 | 4002.183922 |
| 4002.183986 | Kernel\_Read\|xilinx:adm-pcie-7v3:1ddr:3.0-0\|cnn\_GOOD | Read | OCL Region | 1 | 1 | 117593 | 117594 | 0.00333333 | 4002.183986 | 4002.183989 |
| 4002.184049 | Kernel\_Read\|xilinx:adm-pcie-7v3:1ddr:3.0-0\|cnn\_GOOD | Read | OCL Region | 1 | 1 | 117612 | 117613 | 0.00333333 | 4002.184049 | 4002.184052 |
| 4002.184119 | Kernel\_Read\|xilinx:adm-pcie-7v3:1ddr:3.0-0\|cnn\_GOOD | Read | OCL Region | 1 | 1 | 117633 | 117634 | 0.00333333 | 4002.184119 | 4002.184122 |
| 4002.184186 | Kernel\_Read\|xilinx:adm-pcie-7v3:1ddr:3.0-0\|cnn\_GOOD | Read | OCL Region | 1 | 1 | 117653 | 117654 | 0.00333333 | 4002.184186 | 4002.184189 |
| 4002.184259 | Kernel\_Read\|xilinx:adm-pcie-7v3:1ddr:3.0-0\|cnn\_GOOD | Read | OCL Region | 1 | 1 | 117675 | 117676 | 0.00333333 | 4002.184259 | 4002.184262 |
| 4002.184319 | Kernel\_Read\|xilinx:adm-pcie-7v3:1ddr:3.0-0\|cnn\_GOOD | Read | OCL Region | 1 | 1 | 117693 | 117694 | 0.00333333 | 4002.184319 | 4002.184322 |
| 4002.184386 | Kernel\_Read\|xilinx:adm-pcie-7v3:1ddr:3.0-0\|cnn\_GOOD | Read | OCL Region | 1 | 1 | 117713 | 117714 | 0.00333333 | 4002.184386 | 4002.184389 |
| 4002.184459 | Kernel\_Read\|xilinx:adm-pcie-7v3:1ddr:3.0-0\|cnn\_GOOD | Read | OCL Region | 1 | 1 | 117735 | 117736 | 0.00333333 | 4002.184459 | 4002.184462 |
| 4002.184519 | Kernel\_Read\|xilinx:adm-pcie-7v3:1ddr:3.0-0\|cnn\_GOOD | Read | OCL Region | 1 | 1 | 117753 | 117754 | 0.00333333 | 4002.184519 | 4002.184522 |
| 4002.184586 | Kernel\_Read\|xilinx:adm-pcie-7v3:1ddr:3.0-0\|cnn\_GOOD | Read | OCL Region | 1 | 1 | 117773 | 117774 | 0.00333333 | 4002.184586 | 4002.184589 |
| 4002.184659 | Kernel\_Read\|xilinx:adm-pcie-7v3:1ddr:3.0-0\|cnn\_GOOD | Read | OCL Region | 1 | 1 | 117795 | 117796 | 0.00333333 | 4002.184659 | 4002.184662 |
| 4002.184719 | Kernel\_Read\|xilinx:adm-pcie-7v3:1ddr:3.0-0\|cnn\_GOOD | Read | OCL Region | 1 | 1 | 117813 | 117814 | 0.00333333 | 4002.184719 | 4002.184722 |
| 4002.184786 | Kernel\_Read\|xilinx:adm-pcie-7v3:1ddr:3.0-0\|cnn\_GOOD | Read | OCL Region | 1 | 1 | 117833 | 117834 | 0.00333333 | 4002.184786 | 4002.184789 |
| 4002.184859 | Kernel\_Read\|xilinx:adm-pcie-7v3:1ddr:3.0-0\|cnn\_GOOD | Read | OCL Region | 1 | 1 | 117855 | 117856 | 0.00333333 | 4002.184859 | 4002.184862 |
| 4002.184919 | Kernel\_Read\|xilinx:adm-pcie-7v3:1ddr:3.0-0\|cnn\_GOOD | Read | OCL Region | 1 | 1 | 117873 | 117874 | 0.00333333 | 4002.184919 | 4002.184922 |
| 4002.184986 | Kernel\_Read\|xilinx:adm-pcie-7v3:1ddr:3.0-0\|cnn\_GOOD | Read | OCL Region | 1 | 1 | 117893 | 117894 | 0.00333333 | 4002.184986 | 4002.184989 |
| 4002.185059 | Kernel\_Read\|xilinx:adm-pcie-7v3:1ddr:3.0-0\|cnn\_GOOD | Read | OCL Region | 1 | 1 | 117915 | 117916 | 0.00333333 | 4002.185059 | 4002.185062 |
| 4002.185119 | Kernel\_Read\|xilinx:adm-pcie-7v3:1ddr:3.0-0\|cnn\_GOOD | Read | OCL Region | 1 | 1 | 117933 | 117934 | 0.00333333 | 4002.185119 | 4002.185122 |
| 4002.185186 | Kernel\_Read\|xilinx:adm-pcie-7v3:1ddr:3.0-0\|cnn\_GOOD | Read | OCL Region | 1 | 1 | 117953 | 117954 | 0.00333333 | 4002.185186 | 4002.185189 |
| 4002.185259 | Kernel\_Read\|xilinx:adm-pcie-7v3:1ddr:3.0-0\|cnn\_GOOD | Read | OCL Region | 1 | 1 | 117975 | 117976 | 0.00333333 | 4002.185259 | 4002.185262 |
| 4002.185319 | Kernel\_Read\|xilinx:adm-pcie-7v3:1ddr:3.0-0\|cnn\_GOOD | Read | OCL Region | 1 | 1 | 117993 | 117994 | 0.00333333 | 4002.185319 | 4002.185322 |
| 4002.185386 | Kernel\_Read\|xilinx:adm-pcie-7v3:1ddr:3.0-0\|cnn\_GOOD | Read | OCL Region | 1 | 1 | 118013 | 118014 | 0.00333333 | 4002.185386 | 4002.185389 |
| 4002.185459 | Kernel\_Read\|xilinx:adm-pcie-7v3:1ddr:3.0-0\|cnn\_GOOD | Read | OCL Region | 1 | 1 | 118035 | 118036 | 0.00333333 | 4002.185459 | 4002.185462 |
| 4002.185519 | Kernel\_Read\|xilinx:adm-pcie-7v3:1ddr:3.0-0\|cnn\_GOOD | Read | OCL Region | 1 | 1 | 118053 | 118054 | 0.00333333 | 4002.185519 | 4002.185522 |
| 4002.185586 | Kernel\_Read\|xilinx:adm-pcie-7v3:1ddr:3.0-0\|cnn\_GOOD | Read | OCL Region | 1 | 1 | 118073 | 118074 | 0.00333333 | 4002.185586 | 4002.185589 |
| 4002.185659 | Kernel\_Read\|xilinx:adm-pcie-7v3:1ddr:3.0-0\|cnn\_GOOD | Read | OCL Region | 1 | 1 | 118095 | 118096 | 0.00333333 | 4002.185659 | 4002.185662 |
| 4002.185736 | Kernel\_Read\|xilinx:adm-pcie-7v3:1ddr:3.0-0\|cnn\_GOOD | Read | OCL Region | 1 | 1 | 118118 | 118119 | 0.00333333 | 4002.185736 | 4002.185739 |
| 4002.185792 | Kernel\_Read\|xilinx:adm-pcie-7v3:1ddr:3.0-0\|cnn\_GOOD | Read | OCL Region | 1 | 1 | 118135 | 118136 | 0.00333333 | 4002.185792 | 4002.185796 |
| 4002.185859 | Kernel\_Read\|xilinx:adm-pcie-7v3:1ddr:3.0-0\|cnn\_GOOD | Read | OCL Region | 1 | 1 | 118155 | 118156 | 0.00333333 | 4002.185859 | 4002.185862 |
| 4002.185919 | Kernel\_Read\|xilinx:adm-pcie-7v3:1ddr:3.0-0\|cnn\_GOOD | Read | OCL Region | 1 | 1 | 118173 | 118174 | 0.00333333 | 4002.185919 | 4002.185922 |
| 4002.185986 | Kernel\_Read\|xilinx:adm-pcie-7v3:1ddr:3.0-0\|cnn\_GOOD | Read | OCL Region | 1 | 1 | 118193 | 118194 | 0.00333333 | 4002.185986 | 4002.185989 |
| 4002.186059 | Kernel\_Read\|xilinx:adm-pcie-7v3:1ddr:3.0-0\|cnn\_GOOD | Read | OCL Region | 1 | 1 | 118215 | 118216 | 0.00333333 | 4002.186059 | 4002.186062 |
| 4002.186119 | Kernel\_Read\|xilinx:adm-pcie-7v3:1ddr:3.0-0\|cnn\_GOOD | Read | OCL Region | 1 | 1 | 118233 | 118234 | 0.00333333 | 4002.186119 | 4002.186122 |
| 4002.186186 | Kernel\_Read\|xilinx:adm-pcie-7v3:1ddr:3.0-0\|cnn\_GOOD | Read | OCL Region | 1 | 1 | 118253 | 118254 | 0.00333333 | 4002.186186 | 4002.186189 |
| 4002.186259 | Kernel\_Read\|xilinx:adm-pcie-7v3:1ddr:3.0-0\|cnn\_GOOD | Read | OCL Region | 1 | 1 | 118275 | 118276 | 0.00333333 | 4002.186259 | 4002.186262 |
| 4002.186319 | Kernel\_Read\|xilinx:adm-pcie-7v3:1ddr:3.0-0\|cnn\_GOOD | Read | OCL Region | 1 | 1 | 118293 | 118294 | 0.00333333 | 4002.186319 | 4002.186322 |
| 4002.186386 | Kernel\_Read\|xilinx:adm-pcie-7v3:1ddr:3.0-0\|cnn\_GOOD | Read | OCL Region | 1 | 1 | 118313 | 118314 | 0.00333333 | 4002.186386 | 4002.186389 |
| 4002.186459 | Kernel\_Read\|xilinx:adm-pcie-7v3:1ddr:3.0-0\|cnn\_GOOD | Read | OCL Region | 1 | 1 | 118335 | 118336 | 0.00333333 | 4002.186459 | 4002.186462 |
| 4002.186526 | Kernel\_Read\|xilinx:adm-pcie-7v3:1ddr:3.0-0\|cnn\_GOOD | Read | OCL Region | 1 | 1 | 118355 | 118356 | 0.00333333 | 4002.186526 | 4002.186529 |
| 4002.186586 | Kernel\_Read\|xilinx:adm-pcie-7v3:1ddr:3.0-0\|cnn\_GOOD | Read | OCL Region | 1 | 1 | 118373 | 118374 | 0.00333333 | 4002.186586 | 4002.186589 |
| 4002.186659 | Kernel\_Read\|xilinx:adm-pcie-7v3:1ddr:3.0-0\|cnn\_GOOD | Read | OCL Region | 1 | 1 | 118395 | 118396 | 0.00333333 | 4002.186659 | 4002.186662 |
| 4002.186719 | Kernel\_Read\|xilinx:adm-pcie-7v3:1ddr:3.0-0\|cnn\_GOOD | Read | OCL Region | 1 | 1 | 118413 | 118414 | 0.00333333 | 4002.186719 | 4002.186722 |
| 4002.186786 | Kernel\_Read\|xilinx:adm-pcie-7v3:1ddr:3.0-0\|cnn\_GOOD | Read | OCL Region | 1 | 1 | 118433 | 118434 | 0.00333333 | 4002.186786 | 4002.186789 |
| 4002.186859 | Kernel\_Read\|xilinx:adm-pcie-7v3:1ddr:3.0-0\|cnn\_GOOD | Read | OCL Region | 1 | 1 | 118455 | 118456 | 0.00333333 | 4002.186859 | 4002.186862 |
| 4002.186919 | Kernel\_Read\|xilinx:adm-pcie-7v3:1ddr:3.0-0\|cnn\_GOOD | Read | OCL Region | 1 | 1 | 118473 | 118474 | 0.00333333 | 4002.186919 | 4002.186922 |
| 4002.186986 | Kernel\_Read\|xilinx:adm-pcie-7v3:1ddr:3.0-0\|cnn\_GOOD | Read | OCL Region | 1 | 1 | 118493 | 118494 | 0.00333333 | 4002.186986 | 4002.186989 |
| 4002.187059 | Kernel\_Read\|xilinx:adm-pcie-7v3:1ddr:3.0-0\|cnn\_GOOD | Read | OCL Region | 1 | 1 | 118515 | 118516 | 0.00333333 | 4002.187059 | 4002.187062 |
| 4002.187119 | Kernel\_Read\|xilinx:adm-pcie-7v3:1ddr:3.0-0\|cnn\_GOOD | Read | OCL Region | 1 | 1 | 118533 | 118534 | 0.00333333 | 4002.187119 | 4002.187122 |
| 4002.187186 | Kernel\_Read\|xilinx:adm-pcie-7v3:1ddr:3.0-0\|cnn\_GOOD | Read | OCL Region | 1 | 1 | 118553 | 118554 | 0.00333333 | 4002.187186 | 4002.187189 |
| 4002.187259 | Kernel\_Read\|xilinx:adm-pcie-7v3:1ddr:3.0-0\|cnn\_GOOD | Read | OCL Region | 1 | 1 | 118575 | 118576 | 0.00333333 | 4002.187259 | 4002.187262 |
| 4002.187319 | Kernel\_Read\|xilinx:adm-pcie-7v3:1ddr:3.0-0\|cnn\_GOOD | Read | OCL Region | 1 | 1 | 118593 | 118594 | 0.00333333 | 4002.187319 | 4002.187322 |
| 4002.187386 | Kernel\_Read\|xilinx:adm-pcie-7v3:1ddr:3.0-0\|cnn\_GOOD | Read | OCL Region | 1 | 1 | 118613 | 118614 | 0.00333333 | 4002.187386 | 4002.187389 |
| 4002.187459 | Kernel\_Read\|xilinx:adm-pcie-7v3:1ddr:3.0-0\|cnn\_GOOD | Read | OCL Region | 1 | 1 | 118635 | 118636 | 0.00333333 | 4002.187459 | 4002.187462 |
| 4002.187519 | Kernel\_Read\|xilinx:adm-pcie-7v3:1ddr:3.0-0\|cnn\_GOOD | Read | OCL Region | 1 | 1 | 118653 | 118654 | 0.00333333 | 4002.187519 | 4002.187522 |
| 4002.187586 | Kernel\_Read\|xilinx:adm-pcie-7v3:1ddr:3.0-0\|cnn\_GOOD | Read | OCL Region | 1 | 1 | 118673 | 118674 | 0.00333333 | 4002.187586 | 4002.187589 |
| 4002.187659 | Kernel\_Read\|xilinx:adm-pcie-7v3:1ddr:3.0-0\|cnn\_GOOD | Read | OCL Region | 1 | 1 | 118695 | 118696 | 0.00333333 | 4002.187659 | 4002.187662 |
| 4002.187932 | Kernel\_Read\|xilinx:adm-pcie-7v3:1ddr:3.0-0\|cnn\_GOOD | Read | OCL Region | 1 | 1 | 118777 | 118778 | 0.00333333 | 4002.187932 | 4002.187936 |
| 4002.187936 | Kernel\_Read\|xilinx:adm-pcie-7v3:1ddr:3.0-0\|cnn\_GOOD | Read | OCL Region | 1 | 1 | 118778 | 118779 | 0.00333333 | 4002.187936 | 4002.187939 |
| 4002.187942 | Kernel\_Read\|xilinx:adm-pcie-7v3:1ddr:3.0-0\|cnn\_GOOD | Read | OCL Region | 1 | 1 | 118780 | 118781 | 0.00333333 | 4002.187942 | 4002.187946 |
| 4002.187946 | Kernel\_Read\|xilinx:adm-pcie-7v3:1ddr:3.0-0\|cnn\_GOOD | Read | OCL Region | 1 | 1 | 118781 | 118782 | 0.00333333 | 4002.187946 | 4002.187949 |
| 4002.188009 | Kernel\_Read\|xilinx:adm-pcie-7v3:1ddr:3.0-0\|cnn\_GOOD | Read | OCL Region | 1 | 1 | 118800 | 118801 | 0.00333333 | 4002.188009 | 4002.188012 |
| 4002.188062 | Kernel\_Read\|xilinx:adm-pcie-7v3:1ddr:3.0-0\|cnn\_GOOD | Read | OCL Region | 1 | 1 | 118816 | 118817 | 0.00333333 | 4002.188062 | 4002.188066 |
| 4002.188119 | Kernel\_Read\|xilinx:adm-pcie-7v3:1ddr:3.0-0\|cnn\_GOOD | Read | OCL Region | 1 | 1 | 118833 | 118834 | 0.00333333 | 4002.188119 | 4002.188122 |
| 4002.188186 | Kernel\_Read\|xilinx:adm-pcie-7v3:1ddr:3.0-0\|cnn\_GOOD | Read | OCL Region | 1 | 1 | 118853 | 118854 | 0.00333333 | 4002.188186 | 4002.188189 |
| 4002.188259 | Kernel\_Read\|xilinx:adm-pcie-7v3:1ddr:3.0-0\|cnn\_GOOD | Read | OCL Region | 1 | 1 | 118875 | 118876 | 0.00333333 | 4002.188259 | 4002.188262 |
| 4002.188319 | Kernel\_Read\|xilinx:adm-pcie-7v3:1ddr:3.0-0\|cnn\_GOOD | Read | OCL Region | 1 | 1 | 118893 | 118894 | 0.00333333 | 4002.188319 | 4002.188322 |
| 4002.188386 | Kernel\_Read\|xilinx:adm-pcie-7v3:1ddr:3.0-0\|cnn\_GOOD | Read | OCL Region | 1 | 1 | 118913 | 118914 | 0.00333333 | 4002.188386 | 4002.188389 |
| 4002.188452 | Kernel\_Read\|xilinx:adm-pcie-7v3:1ddr:3.0-0\|cnn\_GOOD | Read | OCL Region | 1 | 1 | 118933 | 118934 | 0.00333333 | 4002.188452 | 4002.188456 |
| 4002.188519 | Kernel\_Read\|xilinx:adm-pcie-7v3:1ddr:3.0-0\|cnn\_GOOD | Read | OCL Region | 1 | 1 | 118953 | 118954 | 0.00333333 | 4002.188519 | 4002.188522 |
| 4002.188586 | Kernel\_Read\|xilinx:adm-pcie-7v3:1ddr:3.0-0\|cnn\_GOOD | Read | OCL Region | 1 | 1 | 118973 | 118974 | 0.00333333 | 4002.188586 | 4002.188589 |
| 4002.188659 | Kernel\_Read\|xilinx:adm-pcie-7v3:1ddr:3.0-0\|cnn\_GOOD | Read | OCL Region | 1 | 1 | 118995 | 118996 | 0.00333333 | 4002.188659 | 4002.188662 |
| 4002.188719 | Kernel\_Read\|xilinx:adm-pcie-7v3:1ddr:3.0-0\|cnn\_GOOD | Read | OCL Region | 1 | 1 | 119013 | 119014 | 0.00333333 | 4002.188719 | 4002.188722 |
| 4002.188786 | Kernel\_Read\|xilinx:adm-pcie-7v3:1ddr:3.0-0\|cnn\_GOOD | Read | OCL Region | 1 | 1 | 119033 | 119034 | 0.00333333 | 4002.188786 | 4002.188789 |
| 4002.188859 | Kernel\_Read\|xilinx:adm-pcie-7v3:1ddr:3.0-0\|cnn\_GOOD | Read | OCL Region | 1 | 1 | 119055 | 119056 | 0.00333333 | 4002.188859 | 4002.188862 |
| 4002.188919 | Kernel\_Read\|xilinx:adm-pcie-7v3:1ddr:3.0-0\|cnn\_GOOD | Read | OCL Region | 1 | 1 | 119073 | 119074 | 0.00333333 | 4002.188919 | 4002.188922 |
| 4002.188986 | Kernel\_Read\|xilinx:adm-pcie-7v3:1ddr:3.0-0\|cnn\_GOOD | Read | OCL Region | 1 | 1 | 119093 | 119094 | 0.00333333 | 4002.188986 | 4002.188989 |
| 4002.189059 | Kernel\_Read\|xilinx:adm-pcie-7v3:1ddr:3.0-0\|cnn\_GOOD | Read | OCL Region | 1 | 1 | 119115 | 119116 | 0.00333333 | 4002.189059 | 4002.189062 |
| 4002.189119 | Kernel\_Read\|xilinx:adm-pcie-7v3:1ddr:3.0-0\|cnn\_GOOD | Read | OCL Region | 1 | 1 | 119133 | 119134 | 0.00333333 | 4002.189119 | 4002.189122 |
| 4002.189186 | Kernel\_Read\|xilinx:adm-pcie-7v3:1ddr:3.0-0\|cnn\_GOOD | Read | OCL Region | 1 | 1 | 119153 | 119154 | 0.00333333 | 4002.189186 | 4002.189189 |
| 4002.189259 | Kernel\_Read\|xilinx:adm-pcie-7v3:1ddr:3.0-0\|cnn\_GOOD | Read | OCL Region | 1 | 1 | 119175 | 119176 | 0.00333333 | 4002.189259 | 4002.189262 |
| 4002.189319 | Kernel\_Read\|xilinx:adm-pcie-7v3:1ddr:3.0-0\|cnn\_GOOD | Read | OCL Region | 1 | 1 | 119193 | 119194 | 0.00333333 | 4002.189319 | 4002.189322 |
| 4002.189386 | Kernel\_Read\|xilinx:adm-pcie-7v3:1ddr:3.0-0\|cnn\_GOOD | Read | OCL Region | 1 | 1 | 119213 | 119214 | 0.00333333 | 4002.189386 | 4002.189389 |
| 4002.189459 | Kernel\_Read\|xilinx:adm-pcie-7v3:1ddr:3.0-0\|cnn\_GOOD | Read | OCL Region | 1 | 1 | 119235 | 119236 | 0.00333333 | 4002.189459 | 4002.189462 |
| 4002.189529 | Kernel\_Read\|xilinx:adm-pcie-7v3:1ddr:3.0-0\|cnn\_GOOD | Read | OCL Region | 1 | 1 | 119256 | 119257 | 0.00333333 | 4002.189529 | 4002.189532 |
| 4002.189586 | Kernel\_Read\|xilinx:adm-pcie-7v3:1ddr:3.0-0\|cnn\_GOOD | Read | OCL Region | 1 | 1 | 119273 | 119274 | 0.00333333 | 4002.189586 | 4002.189589 |
| 4002.189659 | Kernel\_Read\|xilinx:adm-pcie-7v3:1ddr:3.0-0\|cnn\_GOOD | Read | OCL Region | 1 | 1 | 119295 | 119296 | 0.00333333 | 4002.189659 | 4002.189662 |
| 4002.189719 | Kernel\_Read\|xilinx:adm-pcie-7v3:1ddr:3.0-0\|cnn\_GOOD | Read | OCL Region | 1 | 1 | 119313 | 119314 | 0.00333333 | 4002.189719 | 4002.189722 |
| 4002.189786 | Kernel\_Read\|xilinx:adm-pcie-7v3:1ddr:3.0-0\|cnn\_GOOD | Read | OCL Region | 1 | 1 | 119333 | 119334 | 0.00333333 | 4002.189786 | 4002.189789 |
| 4002.189852 | Kernel\_Read\|xilinx:adm-pcie-7v3:1ddr:3.0-0\|cnn\_GOOD | Read | OCL Region | 1 | 1 | 119353 | 119354 | 0.00333333 | 4002.189852 | 4002.189856 |
| 4002.189919 | Kernel\_Read\|xilinx:adm-pcie-7v3:1ddr:3.0-0\|cnn\_GOOD | Read | OCL Region | 1 | 1 | 119373 | 119374 | 0.00333333 | 4002.189919 | 4002.189922 |
| 4002.189986 | Kernel\_Read\|xilinx:adm-pcie-7v3:1ddr:3.0-0\|cnn\_GOOD | Read | OCL Region | 1 | 1 | 119393 | 119394 | 0.00333333 | 4002.189986 | 4002.189989 |
| 4002.190052 | Kernel\_Read\|xilinx:adm-pcie-7v3:1ddr:3.0-0\|cnn\_GOOD | Read | OCL Region | 1 | 1 | 119413 | 119414 | 0.00333333 | 4002.190052 | 4002.190056 |
| 4002.190119 | Kernel\_Read\|xilinx:adm-pcie-7v3:1ddr:3.0-0\|cnn\_GOOD | Read | OCL Region | 1 | 1 | 119433 | 119434 | 0.00333333 | 4002.190119 | 4002.190122 |
| 4002.190186 | Kernel\_Read\|xilinx:adm-pcie-7v3:1ddr:3.0-0\|cnn\_GOOD | Read | OCL Region | 1 | 1 | 119453 | 119454 | 0.00333333 | 4002.190186 | 4002.190189 |
| 4002.190259 | Kernel\_Read\|xilinx:adm-pcie-7v3:1ddr:3.0-0\|cnn\_GOOD | Read | OCL Region | 1 | 1 | 119475 | 119476 | 0.00333333 | 4002.190259 | 4002.190262 |
| 4002.190319 | Kernel\_Read\|xilinx:adm-pcie-7v3:1ddr:3.0-0\|cnn\_GOOD | Read | OCL Region | 1 | 1 | 119493 | 119494 | 0.00333333 | 4002.190319 | 4002.190322 |
| 4002.190386 | Kernel\_Read\|xilinx:adm-pcie-7v3:1ddr:3.0-0\|cnn\_GOOD | Read | OCL Region | 1 | 1 | 119513 | 119514 | 0.00333333 | 4002.190386 | 4002.190389 |
| 4002.190459 | Kernel\_Read\|xilinx:adm-pcie-7v3:1ddr:3.0-0\|cnn\_GOOD | Read | OCL Region | 1 | 1 | 119535 | 119536 | 0.00333333 | 4002.190459 | 4002.190462 |
| 4002.190519 | Kernel\_Read\|xilinx:adm-pcie-7v3:1ddr:3.0-0\|cnn\_GOOD | Read | OCL Region | 1 | 1 | 119553 | 119554 | 0.00333333 | 4002.190519 | 4002.190522 |
| 4002.190586 | Kernel\_Read\|xilinx:adm-pcie-7v3:1ddr:3.0-0\|cnn\_GOOD | Read | OCL Region | 1 | 1 | 119573 | 119574 | 0.00333333 | 4002.190586 | 4002.190589 |
| 4002.190659 | Kernel\_Read\|xilinx:adm-pcie-7v3:1ddr:3.0-0\|cnn\_GOOD | Read | OCL Region | 1 | 1 | 119595 | 119596 | 0.00333333 | 4002.190659 | 4002.190662 |
| 4002.190719 | Kernel\_Read\|xilinx:adm-pcie-7v3:1ddr:3.0-0\|cnn\_GOOD | Read | OCL Region | 1 | 1 | 119613 | 119614 | 0.00333333 | 4002.190719 | 4002.190722 |
| 4002.190786 | Kernel\_Read\|xilinx:adm-pcie-7v3:1ddr:3.0-0\|cnn\_GOOD | Read | OCL Region | 1 | 1 | 119633 | 119634 | 0.00333333 | 4002.190786 | 4002.190789 |
| 4002.190859 | Kernel\_Read\|xilinx:adm-pcie-7v3:1ddr:3.0-0\|cnn\_GOOD | Read | OCL Region | 1 | 1 | 119655 | 119656 | 0.00333333 | 4002.190859 | 4002.190862 |
| 4002.190919 | Kernel\_Read\|xilinx:adm-pcie-7v3:1ddr:3.0-0\|cnn\_GOOD | Read | OCL Region | 1 | 1 | 119673 | 119674 | 0.00333333 | 4002.190919 | 4002.190922 |
| 4002.190986 | Kernel\_Read\|xilinx:adm-pcie-7v3:1ddr:3.0-0\|cnn\_GOOD | Read | OCL Region | 1 | 1 | 119693 | 119694 | 0.00333333 | 4002.190986 | 4002.190989 |
| 4002.191059 | Kernel\_Read\|xilinx:adm-pcie-7v3:1ddr:3.0-0\|cnn\_GOOD | Read | OCL Region | 1 | 1 | 119715 | 119716 | 0.00333333 | 4002.191059 | 4002.191062 |
| 4002.191119 | Kernel\_Read\|xilinx:adm-pcie-7v3:1ddr:3.0-0\|cnn\_GOOD | Read | OCL Region | 1 | 1 | 119733 | 119734 | 0.00333333 | 4002.191119 | 4002.191122 |
| 4002.191186 | Kernel\_Read\|xilinx:adm-pcie-7v3:1ddr:3.0-0\|cnn\_GOOD | Read | OCL Region | 1 | 1 | 119753 | 119754 | 0.00333333 | 4002.191186 | 4002.191189 |
| 4002.191259 | Kernel\_Read\|xilinx:adm-pcie-7v3:1ddr:3.0-0\|cnn\_GOOD | Read | OCL Region | 1 | 1 | 119775 | 119776 | 0.00333333 | 4002.191259 | 4002.191262 |
| 4002.191319 | Kernel\_Read\|xilinx:adm-pcie-7v3:1ddr:3.0-0\|cnn\_GOOD | Read | OCL Region | 1 | 1 | 119793 | 119794 | 0.00333333 | 4002.191319 | 4002.191322 |
| 4002.191386 | Kernel\_Read\|xilinx:adm-pcie-7v3:1ddr:3.0-0\|cnn\_GOOD | Read | OCL Region | 1 | 1 | 119813 | 119814 | 0.00333333 | 4002.191386 | 4002.191389 |
| 4002.191459 | Kernel\_Read\|xilinx:adm-pcie-7v3:1ddr:3.0-0\|cnn\_GOOD | Read | OCL Region | 1 | 1 | 119835 | 119836 | 0.00333333 | 4002.191459 | 4002.191462 |
| 4002.191519 | Kernel\_Read\|xilinx:adm-pcie-7v3:1ddr:3.0-0\|cnn\_GOOD | Read | OCL Region | 1 | 1 | 119853 | 119854 | 0.00333333 | 4002.191519 | 4002.191522 |
| 4002.191586 | Kernel\_Read\|xilinx:adm-pcie-7v3:1ddr:3.0-0\|cnn\_GOOD | Read | OCL Region | 1 | 1 | 119873 | 119874 | 0.00333333 | 4002.191586 | 4002.191589 |
| 4002.191652 | Kernel\_Read\|xilinx:adm-pcie-7v3:1ddr:3.0-0\|cnn\_GOOD | Read | OCL Region | 1 | 1 | 119893 | 119894 | 0.00333333 | 4002.191652 | 4002.191656 |
| 4002.191719 | Kernel\_Read\|xilinx:adm-pcie-7v3:1ddr:3.0-0\|cnn\_GOOD | Read | OCL Region | 1 | 1 | 119913 | 119914 | 0.00333333 | 4002.191719 | 4002.191722 |
| 4002.191786 | Kernel\_Read\|xilinx:adm-pcie-7v3:1ddr:3.0-0\|cnn\_GOOD | Read | OCL Region | 1 | 1 | 119933 | 119934 | 0.00333333 | 4002.191786 | 4002.191789 |
| 4002.191859 | Kernel\_Read\|xilinx:adm-pcie-7v3:1ddr:3.0-0\|cnn\_GOOD | Read | OCL Region | 1 | 1 | 119955 | 119956 | 0.00333333 | 4002.191859 | 4002.191862 |
| 4002.191919 | Kernel\_Read\|xilinx:adm-pcie-7v3:1ddr:3.0-0\|cnn\_GOOD | Read | OCL Region | 1 | 1 | 119973 | 119974 | 0.00333333 | 4002.191919 | 4002.191922 |
| 4002.191986 | Kernel\_Read\|xilinx:adm-pcie-7v3:1ddr:3.0-0\|cnn\_GOOD | Read | OCL Region | 1 | 1 | 119993 | 119994 | 0.00333333 | 4002.191986 | 4002.191989 |
| 4002.192059 | Kernel\_Read\|xilinx:adm-pcie-7v3:1ddr:3.0-0\|cnn\_GOOD | Read | OCL Region | 1 | 1 | 120015 | 120016 | 0.00333333 | 4002.192059 | 4002.192062 |
| 4002.192119 | Kernel\_Read\|xilinx:adm-pcie-7v3:1ddr:3.0-0\|cnn\_GOOD | Read | OCL Region | 1 | 1 | 120033 | 120034 | 0.00333333 | 4002.192119 | 4002.192122 |
| 4002.192186 | Kernel\_Read\|xilinx:adm-pcie-7v3:1ddr:3.0-0\|cnn\_GOOD | Read | OCL Region | 1 | 1 | 120053 | 120054 | 0.00333333 | 4002.192186 | 4002.192189 |
| 4002.192259 | Kernel\_Read\|xilinx:adm-pcie-7v3:1ddr:3.0-0\|cnn\_GOOD | Read | OCL Region | 1 | 1 | 120075 | 120076 | 0.00333333 | 4002.192259 | 4002.192262 |
| 4002.192319 | Kernel\_Read\|xilinx:adm-pcie-7v3:1ddr:3.0-0\|cnn\_GOOD | Read | OCL Region | 1 | 1 | 120093 | 120094 | 0.00333333 | 4002.192319 | 4002.192322 |
| 4002.192386 | Kernel\_Read\|xilinx:adm-pcie-7v3:1ddr:3.0-0\|cnn\_GOOD | Read | OCL Region | 1 | 1 | 120113 | 120114 | 0.00333333 | 4002.192386 | 4002.192389 |
| 4002.192459 | Kernel\_Read\|xilinx:adm-pcie-7v3:1ddr:3.0-0\|cnn\_GOOD | Read | OCL Region | 1 | 1 | 120135 | 120136 | 0.00333333 | 4002.192459 | 4002.192462 |
| 4002.192519 | Kernel\_Read\|xilinx:adm-pcie-7v3:1ddr:3.0-0\|cnn\_GOOD | Read | OCL Region | 1 | 1 | 120153 | 120154 | 0.00333333 | 4002.192519 | 4002.192522 |
| 4002.192586 | Kernel\_Read\|xilinx:adm-pcie-7v3:1ddr:3.0-0\|cnn\_GOOD | Read | OCL Region | 1 | 1 | 120173 | 120174 | 0.00333333 | 4002.192586 | 4002.192589 |
| 4002.192659 | Kernel\_Read\|xilinx:adm-pcie-7v3:1ddr:3.0-0\|cnn\_GOOD | Read | OCL Region | 1 | 1 | 120195 | 120196 | 0.00333333 | 4002.192659 | 4002.192662 |
| 4002.192719 | Kernel\_Read\|xilinx:adm-pcie-7v3:1ddr:3.0-0\|cnn\_GOOD | Read | OCL Region | 1 | 1 | 120213 | 120214 | 0.00333333 | 4002.192719 | 4002.192722 |
| 4002.192796 | Kernel\_Read\|xilinx:adm-pcie-7v3:1ddr:3.0-0\|cnn\_GOOD | Read | OCL Region | 1 | 1 | 120236 | 120237 | 0.00333333 | 4002.192796 | 4002.192799 |
| 4002.192859 | Kernel\_Read\|xilinx:adm-pcie-7v3:1ddr:3.0-0\|cnn\_GOOD | Read | OCL Region | 1 | 1 | 120255 | 120256 | 0.00333333 | 4002.192859 | 4002.192862 |
| 4002.192919 | Kernel\_Read\|xilinx:adm-pcie-7v3:1ddr:3.0-0\|cnn\_GOOD | Read | OCL Region | 1 | 1 | 120273 | 120274 | 0.00333333 | 4002.192919 | 4002.192922 |
| 4002.192986 | Kernel\_Read\|xilinx:adm-pcie-7v3:1ddr:3.0-0\|cnn\_GOOD | Read | OCL Region | 1 | 1 | 120293 | 120294 | 0.00333333 | 4002.192986 | 4002.192989 |
| 4002.193059 | Kernel\_Read\|xilinx:adm-pcie-7v3:1ddr:3.0-0\|cnn\_GOOD | Read | OCL Region | 1 | 1 | 120315 | 120316 | 0.00333333 | 4002.193059 | 4002.193062 |
| 4002.193119 | Kernel\_Read\|xilinx:adm-pcie-7v3:1ddr:3.0-0\|cnn\_GOOD | Read | OCL Region | 1 | 1 | 120333 | 120334 | 0.00333333 | 4002.193119 | 4002.193122 |
| 4002.193186 | Kernel\_Read\|xilinx:adm-pcie-7v3:1ddr:3.0-0\|cnn\_GOOD | Read | OCL Region | 1 | 1 | 120353 | 120354 | 0.00333333 | 4002.193186 | 4002.193189 |
| 4002.193252 | Kernel\_Read\|xilinx:adm-pcie-7v3:1ddr:3.0-0\|cnn\_GOOD | Read | OCL Region | 1 | 1 | 120373 | 120374 | 0.00333333 | 4002.193252 | 4002.193256 |
| 4002.193319 | Kernel\_Read\|xilinx:adm-pcie-7v3:1ddr:3.0-0\|cnn\_GOOD | Read | OCL Region | 1 | 1 | 120393 | 120394 | 0.00333333 | 4002.193319 | 4002.193322 |
| 4002.193386 | Kernel\_Read\|xilinx:adm-pcie-7v3:1ddr:3.0-0\|cnn\_GOOD | Read | OCL Region | 1 | 1 | 120413 | 120414 | 0.00333333 | 4002.193386 | 4002.193389 |
| 4002.193459 | Kernel\_Read\|xilinx:adm-pcie-7v3:1ddr:3.0-0\|cnn\_GOOD | Read | OCL Region | 1 | 1 | 120435 | 120436 | 0.00333333 | 4002.193459 | 4002.193462 |
| 4002.193519 | Kernel\_Read\|xilinx:adm-pcie-7v3:1ddr:3.0-0\|cnn\_GOOD | Read | OCL Region | 1 | 1 | 120453 | 120454 | 0.00333333 | 4002.193519 | 4002.193522 |
| 4002.193586 | Kernel\_Read\|xilinx:adm-pcie-7v3:1ddr:3.0-0\|cnn\_GOOD | Read | OCL Region | 1 | 1 | 120473 | 120474 | 0.00333333 | 4002.193586 | 4002.193589 |
| 4002.193659 | Kernel\_Read\|xilinx:adm-pcie-7v3:1ddr:3.0-0\|cnn\_GOOD | Read | OCL Region | 1 | 1 | 120495 | 120496 | 0.00333333 | 4002.193659 | 4002.193662 |
| 4002.193719 | Kernel\_Read\|xilinx:adm-pcie-7v3:1ddr:3.0-0\|cnn\_GOOD | Read | OCL Region | 1 | 1 | 120513 | 120514 | 0.00333333 | 4002.193719 | 4002.193722 |
| 4002.193786 | Kernel\_Read\|xilinx:adm-pcie-7v3:1ddr:3.0-0\|cnn\_GOOD | Read | OCL Region | 1 | 1 | 120533 | 120534 | 0.00333333 | 4002.193786 | 4002.193789 |
| 4002.193859 | Kernel\_Read\|xilinx:adm-pcie-7v3:1ddr:3.0-0\|cnn\_GOOD | Read | OCL Region | 1 | 1 | 120555 | 120556 | 0.00333333 | 4002.193859 | 4002.193862 |
| 4002.193919 | Kernel\_Read\|xilinx:adm-pcie-7v3:1ddr:3.0-0\|cnn\_GOOD | Read | OCL Region | 1 | 1 | 120573 | 120574 | 0.00333333 | 4002.193919 | 4002.193922 |
| 4002.194196 | Kernel\_Read\|xilinx:adm-pcie-7v3:1ddr:3.0-0\|cnn\_GOOD | Read | OCL Region | 1 | 1 | 120656 | 120657 | 0.00333333 | 4002.194196 | 4002.194199 |
| 4002.194199 | Kernel\_Read\|xilinx:adm-pcie-7v3:1ddr:3.0-0\|cnn\_GOOD | Read | OCL Region | 1 | 1 | 120657 | 120658 | 0.00333333 | 4002.194199 | 4002.194202 |
| 4002.194209 | Kernel\_Read\|xilinx:adm-pcie-7v3:1ddr:3.0-0\|cnn\_GOOD | Read | OCL Region | 1 | 1 | 120660 | 120661 | 0.00333333 | 4002.194209 | 4002.194212 |
| 4002.194212 | Kernel\_Read\|xilinx:adm-pcie-7v3:1ddr:3.0-0\|cnn\_GOOD | Read | OCL Region | 1 | 1 | 120661 | 120662 | 0.00333333 | 4002.194212 | 4002.194216 |
| 4002.194269 | Kernel\_Read\|xilinx:adm-pcie-7v3:1ddr:3.0-0\|cnn\_GOOD | Read | OCL Region | 1 | 1 | 120678 | 120679 | 0.00333333 | 4002.194269 | 4002.194272 |
| 4002.194326 | Kernel\_Read\|xilinx:adm-pcie-7v3:1ddr:3.0-0\|cnn\_GOOD | Read | OCL Region | 1 | 1 | 120695 | 120696 | 0.00333333 | 4002.194326 | 4002.194329 |
| 4002.194386 | Kernel\_Read\|xilinx:adm-pcie-7v3:1ddr:3.0-0\|cnn\_GOOD | Read | OCL Region | 1 | 1 | 120713 | 120714 | 0.00333333 | 4002.194386 | 4002.194389 |
| 4002.194459 | Kernel\_Read\|xilinx:adm-pcie-7v3:1ddr:3.0-0\|cnn\_GOOD | Read | OCL Region | 1 | 1 | 120735 | 120736 | 0.00333333 | 4002.194459 | 4002.194462 |
| 4002.194519 | Kernel\_Read\|xilinx:adm-pcie-7v3:1ddr:3.0-0\|cnn\_GOOD | Read | OCL Region | 1 | 1 | 120753 | 120754 | 0.00333333 | 4002.194519 | 4002.194522 |
| 4002.194586 | Kernel\_Read\|xilinx:adm-pcie-7v3:1ddr:3.0-0\|cnn\_GOOD | Read | OCL Region | 1 | 1 | 120773 | 120774 | 0.00333333 | 4002.194586 | 4002.194589 |
| 4002.194659 | Kernel\_Read\|xilinx:adm-pcie-7v3:1ddr:3.0-0\|cnn\_GOOD | Read | OCL Region | 1 | 1 | 120795 | 120796 | 0.00333333 | 4002.194659 | 4002.194662 |
| 4002.194719 | Kernel\_Read\|xilinx:adm-pcie-7v3:1ddr:3.0-0\|cnn\_GOOD | Read | OCL Region | 1 | 1 | 120813 | 120814 | 0.00333333 | 4002.194719 | 4002.194722 |
| 4002.194786 | Kernel\_Read\|xilinx:adm-pcie-7v3:1ddr:3.0-0\|cnn\_GOOD | Read | OCL Region | 1 | 1 | 120833 | 120834 | 0.00333333 | 4002.194786 | 4002.194789 |
| 4002.194852 | Kernel\_Read\|xilinx:adm-pcie-7v3:1ddr:3.0-0\|cnn\_GOOD | Read | OCL Region | 1 | 1 | 120853 | 120854 | 0.00333333 | 4002.194852 | 4002.194856 |
| 4002.194919 | Kernel\_Read\|xilinx:adm-pcie-7v3:1ddr:3.0-0\|cnn\_GOOD | Read | OCL Region | 1 | 1 | 120873 | 120874 | 0.00333333 | 4002.194919 | 4002.194922 |
| 4002.194986 | Kernel\_Read\|xilinx:adm-pcie-7v3:1ddr:3.0-0\|cnn\_GOOD | Read | OCL Region | 1 | 1 | 120893 | 120894 | 0.00333333 | 4002.194986 | 4002.194989 |
| 4002.195059 | Kernel\_Read\|xilinx:adm-pcie-7v3:1ddr:3.0-0\|cnn\_GOOD | Read | OCL Region | 1 | 1 | 120915 | 120916 | 0.00333333 | 4002.195059 | 4002.195062 |
| 4002.195119 | Kernel\_Read\|xilinx:adm-pcie-7v3:1ddr:3.0-0\|cnn\_GOOD | Read | OCL Region | 1 | 1 | 120933 | 120934 | 0.00333333 | 4002.195119 | 4002.195122 |
| 4002.195186 | Kernel\_Read\|xilinx:adm-pcie-7v3:1ddr:3.0-0\|cnn\_GOOD | Read | OCL Region | 1 | 1 | 120953 | 120954 | 0.00333333 | 4002.195186 | 4002.195189 |
| 4002.195259 | Kernel\_Read\|xilinx:adm-pcie-7v3:1ddr:3.0-0\|cnn\_GOOD | Read | OCL Region | 1 | 1 | 120975 | 120976 | 0.00333333 | 4002.195259 | 4002.195262 |
| 4002.195319 | Kernel\_Read\|xilinx:adm-pcie-7v3:1ddr:3.0-0\|cnn\_GOOD | Read | OCL Region | 1 | 1 | 120993 | 120994 | 0.00333333 | 4002.195319 | 4002.195322 |
| 4002.195386 | Kernel\_Read\|xilinx:adm-pcie-7v3:1ddr:3.0-0\|cnn\_GOOD | Read | OCL Region | 1 | 1 | 121013 | 121014 | 0.00333333 | 4002.195386 | 4002.195389 |
| 4002.195459 | Kernel\_Read\|xilinx:adm-pcie-7v3:1ddr:3.0-0\|cnn\_GOOD | Read | OCL Region | 1 | 1 | 121035 | 121036 | 0.00333333 | 4002.195459 | 4002.195462 |
| 4002.195519 | Kernel\_Read\|xilinx:adm-pcie-7v3:1ddr:3.0-0\|cnn\_GOOD | Read | OCL Region | 1 | 1 | 121053 | 121054 | 0.00333333 | 4002.195519 | 4002.195522 |
| 4002.195586 | Kernel\_Read\|xilinx:adm-pcie-7v3:1ddr:3.0-0\|cnn\_GOOD | Read | OCL Region | 1 | 1 | 121073 | 121074 | 0.00333333 | 4002.195586 | 4002.195589 |
| 4002.195659 | Kernel\_Read\|xilinx:adm-pcie-7v3:1ddr:3.0-0\|cnn\_GOOD | Read | OCL Region | 1 | 1 | 121095 | 121096 | 0.00333333 | 4002.195659 | 4002.195662 |
| 4002.195719 | Kernel\_Read\|xilinx:adm-pcie-7v3:1ddr:3.0-0\|cnn\_GOOD | Read | OCL Region | 1 | 1 | 121113 | 121114 | 0.00333333 | 4002.195719 | 4002.195722 |
| 4002.195799 | Kernel\_Read\|xilinx:adm-pcie-7v3:1ddr:3.0-0\|cnn\_GOOD | Read | OCL Region | 1 | 1 | 121137 | 121138 | 0.00333333 | 4002.195799 | 4002.195802 |
| 4002.195859 | Kernel\_Read\|xilinx:adm-pcie-7v3:1ddr:3.0-0\|cnn\_GOOD | Read | OCL Region | 1 | 1 | 121155 | 121156 | 0.00333333 | 4002.195859 | 4002.195862 |
| 4002.195919 | Kernel\_Read\|xilinx:adm-pcie-7v3:1ddr:3.0-0\|cnn\_GOOD | Read | OCL Region | 1 | 1 | 121173 | 121174 | 0.00333333 | 4002.195919 | 4002.195922 |
| 4002.195986 | Kernel\_Read\|xilinx:adm-pcie-7v3:1ddr:3.0-0\|cnn\_GOOD | Read | OCL Region | 1 | 1 | 121193 | 121194 | 0.00333333 | 4002.195986 | 4002.195989 |
| 4002.196059 | Kernel\_Read\|xilinx:adm-pcie-7v3:1ddr:3.0-0\|cnn\_GOOD | Read | OCL Region | 1 | 1 | 121215 | 121216 | 0.00333333 | 4002.196059 | 4002.196062 |
| 4002.196119 | Kernel\_Read\|xilinx:adm-pcie-7v3:1ddr:3.0-0\|cnn\_GOOD | Read | OCL Region | 1 | 1 | 121233 | 121234 | 0.00333333 | 4002.196119 | 4002.196122 |
| 4002.196186 | Kernel\_Read\|xilinx:adm-pcie-7v3:1ddr:3.0-0\|cnn\_GOOD | Read | OCL Region | 1 | 1 | 121253 | 121254 | 0.00333333 | 4002.196186 | 4002.196189 |
| 4002.196252 | Kernel\_Read\|xilinx:adm-pcie-7v3:1ddr:3.0-0\|cnn\_GOOD | Read | OCL Region | 1 | 1 | 121273 | 121274 | 0.00333333 | 4002.196252 | 4002.196256 |
| 4002.196319 | Kernel\_Read\|xilinx:adm-pcie-7v3:1ddr:3.0-0\|cnn\_GOOD | Read | OCL Region | 1 | 1 | 121293 | 121294 | 0.00333333 | 4002.196319 | 4002.196322 |
| 4002.196386 | Kernel\_Read\|xilinx:adm-pcie-7v3:1ddr:3.0-0\|cnn\_GOOD | Read | OCL Region | 1 | 1 | 121313 | 121314 | 0.00333333 | 4002.196386 | 4002.196389 |
| 4002.196452 | Kernel\_Read\|xilinx:adm-pcie-7v3:1ddr:3.0-0\|cnn\_GOOD | Read | OCL Region | 1 | 1 | 121333 | 121334 | 0.00333333 | 4002.196452 | 4002.196456 |
| 4002.196519 | Kernel\_Read\|xilinx:adm-pcie-7v3:1ddr:3.0-0\|cnn\_GOOD | Read | OCL Region | 1 | 1 | 121353 | 121354 | 0.00333333 | 4002.196519 | 4002.196522 |
| 4002.196592 | Kernel\_Read\|xilinx:adm-pcie-7v3:1ddr:3.0-0\|cnn\_GOOD | Read | OCL Region | 1 | 1 | 121375 | 121376 | 0.00333333 | 4002.196592 | 4002.196596 |
| 4002.196659 | Kernel\_Read\|xilinx:adm-pcie-7v3:1ddr:3.0-0\|cnn\_GOOD | Read | OCL Region | 1 | 1 | 121395 | 121396 | 0.00333333 | 4002.196659 | 4002.196662 |
| 4002.196719 | Kernel\_Read\|xilinx:adm-pcie-7v3:1ddr:3.0-0\|cnn\_GOOD | Read | OCL Region | 1 | 1 | 121413 | 121414 | 0.00333333 | 4002.196719 | 4002.196722 |
| 4002.196786 | Kernel\_Read\|xilinx:adm-pcie-7v3:1ddr:3.0-0\|cnn\_GOOD | Read | OCL Region | 1 | 1 | 121433 | 121434 | 0.00333333 | 4002.196786 | 4002.196789 |
| 4002.196859 | Kernel\_Read\|xilinx:adm-pcie-7v3:1ddr:3.0-0\|cnn\_GOOD | Read | OCL Region | 1 | 1 | 121455 | 121456 | 0.00333333 | 4002.196859 | 4002.196862 |
| 4002.196919 | Kernel\_Read\|xilinx:adm-pcie-7v3:1ddr:3.0-0\|cnn\_GOOD | Read | OCL Region | 1 | 1 | 121473 | 121474 | 0.00333333 | 4002.196919 | 4002.196922 |
| 4002.196986 | Kernel\_Read\|xilinx:adm-pcie-7v3:1ddr:3.0-0\|cnn\_GOOD | Read | OCL Region | 1 | 1 | 121493 | 121494 | 0.00333333 | 4002.196986 | 4002.196989 |
| 4002.197052 | Kernel\_Read\|xilinx:adm-pcie-7v3:1ddr:3.0-0\|cnn\_GOOD | Read | OCL Region | 1 | 1 | 121513 | 121514 | 0.00333333 | 4002.197052 | 4002.197056 |
| 4002.197119 | Kernel\_Read\|xilinx:adm-pcie-7v3:1ddr:3.0-0\|cnn\_GOOD | Read | OCL Region | 1 | 1 | 121533 | 121534 | 0.00333333 | 4002.197119 | 4002.197122 |
| 4002.197186 | Kernel\_Read\|xilinx:adm-pcie-7v3:1ddr:3.0-0\|cnn\_GOOD | Read | OCL Region | 1 | 1 | 121553 | 121554 | 0.00333333 | 4002.197186 | 4002.197189 |
| 4002.197259 | Kernel\_Read\|xilinx:adm-pcie-7v3:1ddr:3.0-0\|cnn\_GOOD | Read | OCL Region | 1 | 1 | 121575 | 121576 | 0.00333333 | 4002.197259 | 4002.197262 |
| 4002.197319 | Kernel\_Read\|xilinx:adm-pcie-7v3:1ddr:3.0-0\|cnn\_GOOD | Read | OCL Region | 1 | 1 | 121593 | 121594 | 0.00333333 | 4002.197319 | 4002.197322 |
| 4002.197386 | Kernel\_Read\|xilinx:adm-pcie-7v3:1ddr:3.0-0\|cnn\_GOOD | Read | OCL Region | 1 | 1 | 121613 | 121614 | 0.00333333 | 4002.197386 | 4002.197389 |
| 4002.197459 | Kernel\_Read\|xilinx:adm-pcie-7v3:1ddr:3.0-0\|cnn\_GOOD | Read | OCL Region | 1 | 1 | 121635 | 121636 | 0.00333333 | 4002.197459 | 4002.197462 |
| 4002.197519 | Kernel\_Read\|xilinx:adm-pcie-7v3:1ddr:3.0-0\|cnn\_GOOD | Read | OCL Region | 1 | 1 | 121653 | 121654 | 0.00333333 | 4002.197519 | 4002.197522 |
| 4002.197586 | Kernel\_Read\|xilinx:adm-pcie-7v3:1ddr:3.0-0\|cnn\_GOOD | Read | OCL Region | 1 | 1 | 121673 | 121674 | 0.00333333 | 4002.197586 | 4002.197589 |
| 4002.197659 | Kernel\_Read\|xilinx:adm-pcie-7v3:1ddr:3.0-0\|cnn\_GOOD | Read | OCL Region | 1 | 1 | 121695 | 121696 | 0.00333333 | 4002.197659 | 4002.197662 |
| 4002.197719 | Kernel\_Read\|xilinx:adm-pcie-7v3:1ddr:3.0-0\|cnn\_GOOD | Read | OCL Region | 1 | 1 | 121713 | 121714 | 0.00333333 | 4002.197719 | 4002.197722 |
| 4002.197786 | Kernel\_Read\|xilinx:adm-pcie-7v3:1ddr:3.0-0\|cnn\_GOOD | Read | OCL Region | 1 | 1 | 121733 | 121734 | 0.00333333 | 4002.197786 | 4002.197789 |
| 4002.197859 | Kernel\_Read\|xilinx:adm-pcie-7v3:1ddr:3.0-0\|cnn\_GOOD | Read | OCL Region | 1 | 1 | 121755 | 121756 | 0.00333333 | 4002.197859 | 4002.197862 |
| 4002.197919 | Kernel\_Read\|xilinx:adm-pcie-7v3:1ddr:3.0-0\|cnn\_GOOD | Read | OCL Region | 1 | 1 | 121773 | 121774 | 0.00333333 | 4002.197919 | 4002.197922 |
| 4002.197986 | Kernel\_Read\|xilinx:adm-pcie-7v3:1ddr:3.0-0\|cnn\_GOOD | Read | OCL Region | 1 | 1 | 121793 | 121794 | 0.00333333 | 4002.197986 | 4002.197989 |
| 4002.198052 | Kernel\_Read\|xilinx:adm-pcie-7v3:1ddr:3.0-0\|cnn\_GOOD | Read | OCL Region | 1 | 1 | 121813 | 121814 | 0.00333333 | 4002.198052 | 4002.198056 |
| 4002.198119 | Kernel\_Read\|xilinx:adm-pcie-7v3:1ddr:3.0-0\|cnn\_GOOD | Read | OCL Region | 1 | 1 | 121833 | 121834 | 0.00333333 | 4002.198119 | 4002.198122 |
| 4002.198186 | Kernel\_Read\|xilinx:adm-pcie-7v3:1ddr:3.0-0\|cnn\_GOOD | Read | OCL Region | 1 | 1 | 121853 | 121854 | 0.00333333 | 4002.198186 | 4002.198189 |
| 4002.198249 | Kernel\_Read\|xilinx:adm-pcie-7v3:1ddr:3.0-0\|cnn\_GOOD | Read | OCL Region | 1 | 1 | 121872 | 121873 | 0.00333333 | 4002.198249 | 4002.198252 |
| 4002.198319 | Kernel\_Read\|xilinx:adm-pcie-7v3:1ddr:3.0-0\|cnn\_GOOD | Read | OCL Region | 1 | 1 | 121893 | 121894 | 0.00333333 | 4002.198319 | 4002.198322 |
| 4002.198386 | Kernel\_Read\|xilinx:adm-pcie-7v3:1ddr:3.0-0\|cnn\_GOOD | Read | OCL Region | 1 | 1 | 121913 | 121914 | 0.00333333 | 4002.198386 | 4002.198389 |
| 4002.198459 | Kernel\_Read\|xilinx:adm-pcie-7v3:1ddr:3.0-0\|cnn\_GOOD | Read | OCL Region | 1 | 1 | 121935 | 121936 | 0.00333333 | 4002.198459 | 4002.198462 |
| 4002.198519 | Kernel\_Read\|xilinx:adm-pcie-7v3:1ddr:3.0-0\|cnn\_GOOD | Read | OCL Region | 1 | 1 | 121953 | 121954 | 0.00333333 | 4002.198519 | 4002.198522 |
| 4002.198586 | Kernel\_Read\|xilinx:adm-pcie-7v3:1ddr:3.0-0\|cnn\_GOOD | Read | OCL Region | 1 | 1 | 121973 | 121974 | 0.00333333 | 4002.198586 | 4002.198589 |
| 4002.198652 | Kernel\_Read\|xilinx:adm-pcie-7v3:1ddr:3.0-0\|cnn\_GOOD | Read | OCL Region | 1 | 1 | 121993 | 121994 | 0.00333333 | 4002.198652 | 4002.198656 |
| 4002.198719 | Kernel\_Read\|xilinx:adm-pcie-7v3:1ddr:3.0-0\|cnn\_GOOD | Read | OCL Region | 1 | 1 | 122013 | 122014 | 0.00333333 | 4002.198719 | 4002.198722 |
| 4002.198786 | Kernel\_Read\|xilinx:adm-pcie-7v3:1ddr:3.0-0\|cnn\_GOOD | Read | OCL Region | 1 | 1 | 122033 | 122034 | 0.00333333 | 4002.198786 | 4002.198789 |
| 4002.198859 | Kernel\_Read\|xilinx:adm-pcie-7v3:1ddr:3.0-0\|cnn\_GOOD | Read | OCL Region | 1 | 1 | 122055 | 122056 | 0.00333333 | 4002.198859 | 4002.198862 |
| 4002.198919 | Kernel\_Read\|xilinx:adm-pcie-7v3:1ddr:3.0-0\|cnn\_GOOD | Read | OCL Region | 1 | 1 | 122073 | 122074 | 0.00333333 | 4002.198919 | 4002.198922 |
| 4002.198986 | Kernel\_Read\|xilinx:adm-pcie-7v3:1ddr:3.0-0\|cnn\_GOOD | Read | OCL Region | 1 | 1 | 122093 | 122094 | 0.00333333 | 4002.198986 | 4002.198989 |
| 4002.199066 | Kernel\_Read\|xilinx:adm-pcie-7v3:1ddr:3.0-0\|cnn\_GOOD | Read | OCL Region | 1 | 1 | 122117 | 122118 | 0.00333333 | 4002.199066 | 4002.199069 |
| 4002.199119 | Kernel\_Read\|xilinx:adm-pcie-7v3:1ddr:3.0-0\|cnn\_GOOD | Read | OCL Region | 1 | 1 | 122133 | 122134 | 0.00333333 | 4002.199119 | 4002.199122 |
| 4002.199186 | Kernel\_Read\|xilinx:adm-pcie-7v3:1ddr:3.0-0\|cnn\_GOOD | Read | OCL Region | 1 | 1 | 122153 | 122154 | 0.00333333 | 4002.199186 | 4002.199189 |
| 4002.199259 | Kernel\_Read\|xilinx:adm-pcie-7v3:1ddr:3.0-0\|cnn\_GOOD | Read | OCL Region | 1 | 1 | 122175 | 122176 | 0.00333333 | 4002.199259 | 4002.199262 |
| 4002.199319 | Kernel\_Read\|xilinx:adm-pcie-7v3:1ddr:3.0-0\|cnn\_GOOD | Read | OCL Region | 1 | 1 | 122193 | 122194 | 0.00333333 | 4002.199319 | 4002.199322 |
| 4002.199386 | Kernel\_Read\|xilinx:adm-pcie-7v3:1ddr:3.0-0\|cnn\_GOOD | Read | OCL Region | 1 | 1 | 122213 | 122214 | 0.00333333 | 4002.199386 | 4002.199389 |
| 4002.199459 | Kernel\_Read\|xilinx:adm-pcie-7v3:1ddr:3.0-0\|cnn\_GOOD | Read | OCL Region | 1 | 1 | 122235 | 122236 | 0.00333333 | 4002.199459 | 4002.199462 |
| 4002.199519 | Kernel\_Read\|xilinx:adm-pcie-7v3:1ddr:3.0-0\|cnn\_GOOD | Read | OCL Region | 1 | 1 | 122253 | 122254 | 0.00333333 | 4002.199519 | 4002.199522 |
| 4002.199586 | Kernel\_Read\|xilinx:adm-pcie-7v3:1ddr:3.0-0\|cnn\_GOOD | Read | OCL Region | 1 | 1 | 122273 | 122274 | 0.00333333 | 4002.199586 | 4002.199589 |
| 4002.199652 | Kernel\_Read\|xilinx:adm-pcie-7v3:1ddr:3.0-0\|cnn\_GOOD | Read | OCL Region | 1 | 1 | 122293 | 122294 | 0.00333333 | 4002.199652 | 4002.199656 |
| 4002.199719 | Kernel\_Read\|xilinx:adm-pcie-7v3:1ddr:3.0-0\|cnn\_GOOD | Read | OCL Region | 1 | 1 | 122313 | 122314 | 0.00333333 | 4002.199719 | 4002.199722 |
| 4002.199786 | Kernel\_Read\|xilinx:adm-pcie-7v3:1ddr:3.0-0\|cnn\_GOOD | Read | OCL Region | 1 | 1 | 122333 | 122334 | 0.00333333 | 4002.199786 | 4002.199789 |
| 4002.199859 | Kernel\_Read\|xilinx:adm-pcie-7v3:1ddr:3.0-0\|cnn\_GOOD | Read | OCL Region | 1 | 1 | 122355 | 122356 | 0.00333333 | 4002.199859 | 4002.199862 |
| 4002.199926 | Kernel\_Read\|xilinx:adm-pcie-7v3:1ddr:3.0-0\|cnn\_GOOD | Read | OCL Region | 1 | 1 | 122375 | 122376 | 0.00333333 | 4002.199926 | 4002.199929 |
| 4002.199986 | Kernel\_Read\|xilinx:adm-pcie-7v3:1ddr:3.0-0\|cnn\_GOOD | Read | OCL Region | 1 | 1 | 122393 | 122394 | 0.00333333 | 4002.199986 | 4002.199989 |
| 4002.200059 | Kernel\_Read\|xilinx:adm-pcie-7v3:1ddr:3.0-0\|cnn\_GOOD | Read | OCL Region | 1 | 1 | 122415 | 122416 | 0.00333333 | 4002.200059 | 4002.200062 |
| 4002.200119 | Kernel\_Read\|xilinx:adm-pcie-7v3:1ddr:3.0-0\|cnn\_GOOD | Read | OCL Region | 1 | 1 | 122433 | 122434 | 0.00333333 | 4002.200119 | 4002.200122 |
| 4002.200186 | Kernel\_Read\|xilinx:adm-pcie-7v3:1ddr:3.0-0\|cnn\_GOOD | Read | OCL Region | 1 | 1 | 122453 | 122454 | 0.00333333 | 4002.200186 | 4002.200189 |
| 4002.200462 | Kernel\_Read\|xilinx:adm-pcie-7v3:1ddr:3.0-0\|cnn\_GOOD | Read | OCL Region | 1 | 1 | 122536 | 122537 | 0.00333333 | 4002.200462 | 4002.200466 |
| 4002.200466 | Kernel\_Read\|xilinx:adm-pcie-7v3:1ddr:3.0-0\|cnn\_GOOD | Read | OCL Region | 1 | 1 | 122537 | 122538 | 0.00333333 | 4002.200466 | 4002.200469 |
| 4002.200469 | Kernel\_Read\|xilinx:adm-pcie-7v3:1ddr:3.0-0\|cnn\_GOOD | Read | OCL Region | 1 | 1 | 122538 | 122539 | 0.00333333 | 4002.200469 | 4002.200472 |
| 4002.200476 | Kernel\_Read\|xilinx:adm-pcie-7v3:1ddr:3.0-0\|cnn\_GOOD | Read | OCL Region | 1 | 1 | 122540 | 122541 | 0.00333333 | 4002.200476 | 4002.200479 |
| 4002.200536 | Kernel\_Read\|xilinx:adm-pcie-7v3:1ddr:3.0-0\|cnn\_GOOD | Read | OCL Region | 1 | 1 | 122558 | 122559 | 0.00333333 | 4002.200536 | 4002.200539 |
| 4002.200592 | Kernel\_Read\|xilinx:adm-pcie-7v3:1ddr:3.0-0\|cnn\_GOOD | Read | OCL Region | 1 | 1 | 122575 | 122576 | 0.00333333 | 4002.200592 | 4002.200596 |
| 4002.200659 | Kernel\_Read\|xilinx:adm-pcie-7v3:1ddr:3.0-0\|cnn\_GOOD | Read | OCL Region | 1 | 1 | 122595 | 122596 | 0.00333333 | 4002.200659 | 4002.200662 |
| 4002.200719 | Kernel\_Read\|xilinx:adm-pcie-7v3:1ddr:3.0-0\|cnn\_GOOD | Read | OCL Region | 1 | 1 | 122613 | 122614 | 0.00333333 | 4002.200719 | 4002.200722 |
| 4002.200786 | Kernel\_Read\|xilinx:adm-pcie-7v3:1ddr:3.0-0\|cnn\_GOOD | Read | OCL Region | 1 | 1 | 122633 | 122634 | 0.00333333 | 4002.200786 | 4002.200789 |
| 4002.200859 | Kernel\_Read\|xilinx:adm-pcie-7v3:1ddr:3.0-0\|cnn\_GOOD | Read | OCL Region | 1 | 1 | 122655 | 122656 | 0.00333333 | 4002.200859 | 4002.200862 |
| 4002.200919 | Kernel\_Read\|xilinx:adm-pcie-7v3:1ddr:3.0-0\|cnn\_GOOD | Read | OCL Region | 1 | 1 | 122673 | 122674 | 0.00333333 | 4002.200919 | 4002.200922 |
| 4002.200986 | Kernel\_Read\|xilinx:adm-pcie-7v3:1ddr:3.0-0\|cnn\_GOOD | Read | OCL Region | 1 | 1 | 122693 | 122694 | 0.00333333 | 4002.200986 | 4002.200989 |
| 4002.201052 | Kernel\_Read\|xilinx:adm-pcie-7v3:1ddr:3.0-0\|cnn\_GOOD | Read | OCL Region | 1 | 1 | 122713 | 122714 | 0.00333333 | 4002.201052 | 4002.201056 |
| 4002.201119 | Kernel\_Read\|xilinx:adm-pcie-7v3:1ddr:3.0-0\|cnn\_GOOD | Read | OCL Region | 1 | 1 | 122733 | 122734 | 0.00333333 | 4002.201119 | 4002.201122 |
| 4002.201186 | Kernel\_Read\|xilinx:adm-pcie-7v3:1ddr:3.0-0\|cnn\_GOOD | Read | OCL Region | 1 | 1 | 122753 | 122754 | 0.00333333 | 4002.201186 | 4002.201189 |
| 4002.201252 | Kernel\_Read\|xilinx:adm-pcie-7v3:1ddr:3.0-0\|cnn\_GOOD | Read | OCL Region | 1 | 1 | 122773 | 122774 | 0.00333333 | 4002.201252 | 4002.201256 |
| 4002.201319 | Kernel\_Read\|xilinx:adm-pcie-7v3:1ddr:3.0-0\|cnn\_GOOD | Read | OCL Region | 1 | 1 | 122793 | 122794 | 0.00333333 | 4002.201319 | 4002.201322 |
| 4002.201386 | Kernel\_Read\|xilinx:adm-pcie-7v3:1ddr:3.0-0\|cnn\_GOOD | Read | OCL Region | 1 | 1 | 122813 | 122814 | 0.00333333 | 4002.201386 | 4002.201389 |
| 4002.201459 | Kernel\_Read\|xilinx:adm-pcie-7v3:1ddr:3.0-0\|cnn\_GOOD | Read | OCL Region | 1 | 1 | 122835 | 122836 | 0.00333333 | 4002.201459 | 4002.201462 |
| 4002.201519 | Kernel\_Read\|xilinx:adm-pcie-7v3:1ddr:3.0-0\|cnn\_GOOD | Read | OCL Region | 1 | 1 | 122853 | 122854 | 0.00333333 | 4002.201519 | 4002.201522 |
| 4002.201586 | Kernel\_Read\|xilinx:adm-pcie-7v3:1ddr:3.0-0\|cnn\_GOOD | Read | OCL Region | 1 | 1 | 122873 | 122874 | 0.00333333 | 4002.201586 | 4002.201589 |
| 4002.201659 | Kernel\_Read\|xilinx:adm-pcie-7v3:1ddr:3.0-0\|cnn\_GOOD | Read | OCL Region | 1 | 1 | 122895 | 122896 | 0.00333333 | 4002.201659 | 4002.201662 |
| 4002.201719 | Kernel\_Read\|xilinx:adm-pcie-7v3:1ddr:3.0-0\|cnn\_GOOD | Read | OCL Region | 1 | 1 | 122913 | 122914 | 0.00333333 | 4002.201719 | 4002.201722 |
| 4002.201786 | Kernel\_Read\|xilinx:adm-pcie-7v3:1ddr:3.0-0\|cnn\_GOOD | Read | OCL Region | 1 | 1 | 122933 | 122934 | 0.00333333 | 4002.201786 | 4002.201789 |
| 4002.201852 | Kernel\_Read\|xilinx:adm-pcie-7v3:1ddr:3.0-0\|cnn\_GOOD | Read | OCL Region | 1 | 1 | 122953 | 122954 | 0.00333333 | 4002.201852 | 4002.201856 |
| 4002.201919 | Kernel\_Read\|xilinx:adm-pcie-7v3:1ddr:3.0-0\|cnn\_GOOD | Read | OCL Region | 1 | 1 | 122973 | 122974 | 0.00333333 | 4002.201919 | 4002.201922 |
| 4002.201986 | Kernel\_Read\|xilinx:adm-pcie-7v3:1ddr:3.0-0\|cnn\_GOOD | Read | OCL Region | 1 | 1 | 122993 | 122994 | 0.00333333 | 4002.201986 | 4002.201989 |
| 4002.202049 | Kernel\_Read\|xilinx:adm-pcie-7v3:1ddr:3.0-0\|cnn\_GOOD | Read | OCL Region | 1 | 1 | 123012 | 123013 | 0.00333333 | 4002.202049 | 4002.202052 |
| 4002.202119 | Kernel\_Read\|xilinx:adm-pcie-7v3:1ddr:3.0-0\|cnn\_GOOD | Read | OCL Region | 1 | 1 | 123033 | 123034 | 0.00333333 | 4002.202119 | 4002.202122 |
| 4002.202186 | Kernel\_Read\|xilinx:adm-pcie-7v3:1ddr:3.0-0\|cnn\_GOOD | Read | OCL Region | 1 | 1 | 123053 | 123054 | 0.00333333 | 4002.202186 | 4002.202189 |
| 4002.202252 | Kernel\_Read\|xilinx:adm-pcie-7v3:1ddr:3.0-0\|cnn\_GOOD | Read | OCL Region | 1 | 1 | 123073 | 123074 | 0.00333333 | 4002.202252 | 4002.202256 |
| 4002.202319 | Kernel\_Read\|xilinx:adm-pcie-7v3:1ddr:3.0-0\|cnn\_GOOD | Read | OCL Region | 1 | 1 | 123093 | 123094 | 0.00333333 | 4002.202319 | 4002.202322 |
| 4002.202386 | Kernel\_Read\|xilinx:adm-pcie-7v3:1ddr:3.0-0\|cnn\_GOOD | Read | OCL Region | 1 | 1 | 123113 | 123114 | 0.00333333 | 4002.202386 | 4002.202389 |
| 4002.202459 | Kernel\_Read\|xilinx:adm-pcie-7v3:1ddr:3.0-0\|cnn\_GOOD | Read | OCL Region | 1 | 1 | 123135 | 123136 | 0.00333333 | 4002.202459 | 4002.202462 |
| 4002.202519 | Kernel\_Read\|xilinx:adm-pcie-7v3:1ddr:3.0-0\|cnn\_GOOD | Read | OCL Region | 1 | 1 | 123153 | 123154 | 0.00333333 | 4002.202519 | 4002.202522 |
| 4002.202586 | Kernel\_Read\|xilinx:adm-pcie-7v3:1ddr:3.0-0\|cnn\_GOOD | Read | OCL Region | 1 | 1 | 123173 | 123174 | 0.00333333 | 4002.202586 | 4002.202589 |
| 4002.202652 | Kernel\_Read\|xilinx:adm-pcie-7v3:1ddr:3.0-0\|cnn\_GOOD | Read | OCL Region | 1 | 1 | 123193 | 123194 | 0.00333333 | 4002.202652 | 4002.202656 |
| 4002.202719 | Kernel\_Read\|xilinx:adm-pcie-7v3:1ddr:3.0-0\|cnn\_GOOD | Read | OCL Region | 1 | 1 | 123213 | 123214 | 0.00333333 | 4002.202719 | 4002.202722 |
| 4002.202786 | Kernel\_Read\|xilinx:adm-pcie-7v3:1ddr:3.0-0\|cnn\_GOOD | Read | OCL Region | 1 | 1 | 123233 | 123234 | 0.00333333 | 4002.202786 | 4002.202789 |
| 4002.202862 | Kernel\_Read\|xilinx:adm-pcie-7v3:1ddr:3.0-0\|cnn\_GOOD | Read | OCL Region | 1 | 1 | 123256 | 123257 | 0.00333333 | 4002.202862 | 4002.202866 |
| 4002.202919 | Kernel\_Read\|xilinx:adm-pcie-7v3:1ddr:3.0-0\|cnn\_GOOD | Read | OCL Region | 1 | 1 | 123273 | 123274 | 0.00333333 | 4002.202919 | 4002.202922 |
| 4002.202986 | Kernel\_Read\|xilinx:adm-pcie-7v3:1ddr:3.0-0\|cnn\_GOOD | Read | OCL Region | 1 | 1 | 123293 | 123294 | 0.00333333 | 4002.202986 | 4002.202989 |
| 4002.203052 | Kernel\_Read\|xilinx:adm-pcie-7v3:1ddr:3.0-0\|cnn\_GOOD | Read | OCL Region | 1 | 1 | 123313 | 123314 | 0.00333333 | 4002.203052 | 4002.203056 |
| 4002.203119 | Kernel\_Read\|xilinx:adm-pcie-7v3:1ddr:3.0-0\|cnn\_GOOD | Read | OCL Region | 1 | 1 | 123333 | 123334 | 0.00333333 | 4002.203119 | 4002.203122 |
| 4002.203186 | Kernel\_Read\|xilinx:adm-pcie-7v3:1ddr:3.0-0\|cnn\_GOOD | Read | OCL Region | 1 | 1 | 123353 | 123354 | 0.00333333 | 4002.203186 | 4002.203189 |
| 4002.203259 | Kernel\_Read\|xilinx:adm-pcie-7v3:1ddr:3.0-0\|cnn\_GOOD | Read | OCL Region | 1 | 1 | 123375 | 123376 | 0.00333333 | 4002.203259 | 4002.203262 |
| 4002.203319 | Kernel\_Read\|xilinx:adm-pcie-7v3:1ddr:3.0-0\|cnn\_GOOD | Read | OCL Region | 1 | 1 | 123393 | 123394 | 0.00333333 | 4002.203319 | 4002.203322 |
| 4002.203386 | Kernel\_Read\|xilinx:adm-pcie-7v3:1ddr:3.0-0\|cnn\_GOOD | Read | OCL Region | 1 | 1 | 123413 | 123414 | 0.00333333 | 4002.203386 | 4002.203389 |
| 4002.203452 | Kernel\_Read\|xilinx:adm-pcie-7v3:1ddr:3.0-0\|cnn\_GOOD | Read | OCL Region | 1 | 1 | 123433 | 123434 | 0.00333333 | 4002.203452 | 4002.203456 |
| 4002.203519 | Kernel\_Read\|xilinx:adm-pcie-7v3:1ddr:3.0-0\|cnn\_GOOD | Read | OCL Region | 1 | 1 | 123453 | 123454 | 0.00333333 | 4002.203519 | 4002.203522 |
| 4002.203586 | Kernel\_Read\|xilinx:adm-pcie-7v3:1ddr:3.0-0\|cnn\_GOOD | Read | OCL Region | 1 | 1 | 123473 | 123474 | 0.00333333 | 4002.203586 | 4002.203589 |
| 4002.203659 | Kernel\_Read\|xilinx:adm-pcie-7v3:1ddr:3.0-0\|cnn\_GOOD | Read | OCL Region | 1 | 1 | 123495 | 123496 | 0.00333333 | 4002.203659 | 4002.203662 |
| 4002.203719 | Kernel\_Read\|xilinx:adm-pcie-7v3:1ddr:3.0-0\|cnn\_GOOD | Read | OCL Region | 1 | 1 | 123513 | 123514 | 0.00333333 | 4002.203719 | 4002.203722 |
| 4002.203786 | Kernel\_Read\|xilinx:adm-pcie-7v3:1ddr:3.0-0\|cnn\_GOOD | Read | OCL Region | 1 | 1 | 123533 | 123534 | 0.00333333 | 4002.203786 | 4002.203789 |
| 4002.203852 | Kernel\_Read\|xilinx:adm-pcie-7v3:1ddr:3.0-0\|cnn\_GOOD | Read | OCL Region | 1 | 1 | 123553 | 123554 | 0.00333333 | 4002.203852 | 4002.203856 |
| 4002.203919 | Kernel\_Read\|xilinx:adm-pcie-7v3:1ddr:3.0-0\|cnn\_GOOD | Read | OCL Region | 1 | 1 | 123573 | 123574 | 0.00333333 | 4002.203919 | 4002.203922 |
| 4002.203986 | Kernel\_Read\|xilinx:adm-pcie-7v3:1ddr:3.0-0\|cnn\_GOOD | Read | OCL Region | 1 | 1 | 123593 | 123594 | 0.00333333 | 4002.203986 | 4002.203989 |
| 4002.204059 | Kernel\_Read\|xilinx:adm-pcie-7v3:1ddr:3.0-0\|cnn\_GOOD | Read | OCL Region | 1 | 1 | 123615 | 123616 | 0.00333333 | 4002.204059 | 4002.204062 |
| 4002.204119 | Kernel\_Read\|xilinx:adm-pcie-7v3:1ddr:3.0-0\|cnn\_GOOD | Read | OCL Region | 1 | 1 | 123633 | 123634 | 0.00333333 | 4002.204119 | 4002.204122 |
| 4002.204186 | Kernel\_Read\|xilinx:adm-pcie-7v3:1ddr:3.0-0\|cnn\_GOOD | Read | OCL Region | 1 | 1 | 123653 | 123654 | 0.00333333 | 4002.204186 | 4002.204189 |
| 4002.204252 | Kernel\_Read\|xilinx:adm-pcie-7v3:1ddr:3.0-0\|cnn\_GOOD | Read | OCL Region | 1 | 1 | 123673 | 123674 | 0.00333333 | 4002.204252 | 4002.204256 |
| 4002.204319 | Kernel\_Read\|xilinx:adm-pcie-7v3:1ddr:3.0-0\|cnn\_GOOD | Read | OCL Region | 1 | 1 | 123693 | 123694 | 0.00333333 | 4002.204319 | 4002.204322 |
| 4002.204386 | Kernel\_Read\|xilinx:adm-pcie-7v3:1ddr:3.0-0\|cnn\_GOOD | Read | OCL Region | 1 | 1 | 123713 | 123714 | 0.00333333 | 4002.204386 | 4002.204389 |
| 4002.204452 | Kernel\_Read\|xilinx:adm-pcie-7v3:1ddr:3.0-0\|cnn\_GOOD | Read | OCL Region | 1 | 1 | 123733 | 123734 | 0.00333333 | 4002.204452 | 4002.204456 |
| 4002.204519 | Kernel\_Read\|xilinx:adm-pcie-7v3:1ddr:3.0-0\|cnn\_GOOD | Read | OCL Region | 1 | 1 | 123753 | 123754 | 0.00333333 | 4002.204519 | 4002.204522 |
| 4002.204586 | Kernel\_Read\|xilinx:adm-pcie-7v3:1ddr:3.0-0\|cnn\_GOOD | Read | OCL Region | 1 | 1 | 123773 | 123774 | 0.00333333 | 4002.204586 | 4002.204589 |
| 4002.204652 | Kernel\_Read\|xilinx:adm-pcie-7v3:1ddr:3.0-0\|cnn\_GOOD | Read | OCL Region | 1 | 1 | 123793 | 123794 | 0.00333333 | 4002.204652 | 4002.204656 |
| 4002.204719 | Kernel\_Read\|xilinx:adm-pcie-7v3:1ddr:3.0-0\|cnn\_GOOD | Read | OCL Region | 1 | 1 | 123813 | 123814 | 0.00333333 | 4002.204719 | 4002.204722 |
| 4002.204786 | Kernel\_Read\|xilinx:adm-pcie-7v3:1ddr:3.0-0\|cnn\_GOOD | Read | OCL Region | 1 | 1 | 123833 | 123834 | 0.00333333 | 4002.204786 | 4002.204789 |
| 4002.204859 | Kernel\_Read\|xilinx:adm-pcie-7v3:1ddr:3.0-0\|cnn\_GOOD | Read | OCL Region | 1 | 1 | 123855 | 123856 | 0.00333333 | 4002.204859 | 4002.204862 |
| 4002.204919 | Kernel\_Read\|xilinx:adm-pcie-7v3:1ddr:3.0-0\|cnn\_GOOD | Read | OCL Region | 1 | 1 | 123873 | 123874 | 0.00333333 | 4002.204919 | 4002.204922 |
| 4002.204986 | Kernel\_Read\|xilinx:adm-pcie-7v3:1ddr:3.0-0\|cnn\_GOOD | Read | OCL Region | 1 | 1 | 123893 | 123894 | 0.00333333 | 4002.204986 | 4002.204989 |
| 4002.205052 | Kernel\_Read\|xilinx:adm-pcie-7v3:1ddr:3.0-0\|cnn\_GOOD | Read | OCL Region | 1 | 1 | 123913 | 123914 | 0.00333333 | 4002.205052 | 4002.205056 |
| 4002.205119 | Kernel\_Read\|xilinx:adm-pcie-7v3:1ddr:3.0-0\|cnn\_GOOD | Read | OCL Region | 1 | 1 | 123933 | 123934 | 0.00333333 | 4002.205119 | 4002.205122 |
| 4002.205186 | Kernel\_Read\|xilinx:adm-pcie-7v3:1ddr:3.0-0\|cnn\_GOOD | Read | OCL Region | 1 | 1 | 123953 | 123954 | 0.00333333 | 4002.205186 | 4002.205189 |
| 4002.205252 | Kernel\_Read\|xilinx:adm-pcie-7v3:1ddr:3.0-0\|cnn\_GOOD | Read | OCL Region | 1 | 1 | 123973 | 123974 | 0.00333333 | 4002.205252 | 4002.205256 |
| 4002.205319 | Kernel\_Read\|xilinx:adm-pcie-7v3:1ddr:3.0-0\|cnn\_GOOD | Read | OCL Region | 1 | 1 | 123993 | 123994 | 0.00333333 | 4002.205319 | 4002.205322 |
| 4002.205386 | Kernel\_Read\|xilinx:adm-pcie-7v3:1ddr:3.0-0\|cnn\_GOOD | Read | OCL Region | 1 | 1 | 124013 | 124014 | 0.00333333 | 4002.205386 | 4002.205389 |
| 4002.205452 | Kernel\_Read\|xilinx:adm-pcie-7v3:1ddr:3.0-0\|cnn\_GOOD | Read | OCL Region | 1 | 1 | 124033 | 124034 | 0.00333333 | 4002.205452 | 4002.205456 |
| 4002.205519 | Kernel\_Read\|xilinx:adm-pcie-7v3:1ddr:3.0-0\|cnn\_GOOD | Read | OCL Region | 1 | 1 | 124053 | 124054 | 0.00333333 | 4002.205519 | 4002.205522 |
| 4002.205586 | Kernel\_Read\|xilinx:adm-pcie-7v3:1ddr:3.0-0\|cnn\_GOOD | Read | OCL Region | 1 | 1 | 124073 | 124074 | 0.00333333 | 4002.205586 | 4002.205589 |
| 4002.205659 | Kernel\_Read\|xilinx:adm-pcie-7v3:1ddr:3.0-0\|cnn\_GOOD | Read | OCL Region | 1 | 1 | 124095 | 124096 | 0.00333333 | 4002.205659 | 4002.205662 |
| 4002.205719 | Kernel\_Read\|xilinx:adm-pcie-7v3:1ddr:3.0-0\|cnn\_GOOD | Read | OCL Region | 1 | 1 | 124113 | 124114 | 0.00333333 | 4002.205719 | 4002.205722 |
| 4002.205786 | Kernel\_Read\|xilinx:adm-pcie-7v3:1ddr:3.0-0\|cnn\_GOOD | Read | OCL Region | 1 | 1 | 124133 | 124134 | 0.00333333 | 4002.205786 | 4002.205789 |
| 4002.205852 | Kernel\_Read\|xilinx:adm-pcie-7v3:1ddr:3.0-0\|cnn\_GOOD | Read | OCL Region | 1 | 1 | 124153 | 124154 | 0.00333333 | 4002.205852 | 4002.205856 |
| 4002.205919 | Kernel\_Read\|xilinx:adm-pcie-7v3:1ddr:3.0-0\|cnn\_GOOD | Read | OCL Region | 1 | 1 | 124173 | 124174 | 0.00333333 | 4002.205919 | 4002.205922 |
| 4002.205986 | Kernel\_Read\|xilinx:adm-pcie-7v3:1ddr:3.0-0\|cnn\_GOOD | Read | OCL Region | 1 | 1 | 124193 | 124194 | 0.00333333 | 4002.205986 | 4002.205989 |
| 4002.206052 | Kernel\_Read\|xilinx:adm-pcie-7v3:1ddr:3.0-0\|cnn\_GOOD | Read | OCL Region | 1 | 1 | 124213 | 124214 | 0.00333333 | 4002.206052 | 4002.206056 |
| 4002.206119 | Kernel\_Read\|xilinx:adm-pcie-7v3:1ddr:3.0-0\|cnn\_GOOD | Read | OCL Region | 1 | 1 | 124233 | 124234 | 0.00333333 | 4002.206119 | 4002.206122 |
| 4002.206196 | Kernel\_Read\|xilinx:adm-pcie-7v3:1ddr:3.0-0\|cnn\_GOOD | Read | OCL Region | 1 | 1 | 124256 | 124257 | 0.00333333 | 4002.206196 | 4002.206199 |
| 4002.206252 | Kernel\_Read\|xilinx:adm-pcie-7v3:1ddr:3.0-0\|cnn\_GOOD | Read | OCL Region | 1 | 1 | 124273 | 124274 | 0.00333333 | 4002.206252 | 4002.206256 |
| 4002.206319 | Kernel\_Read\|xilinx:adm-pcie-7v3:1ddr:3.0-0\|cnn\_GOOD | Read | OCL Region | 1 | 1 | 124293 | 124294 | 0.00333333 | 4002.206319 | 4002.206322 |
| 4002.206386 | Kernel\_Read\|xilinx:adm-pcie-7v3:1ddr:3.0-0\|cnn\_GOOD | Read | OCL Region | 1 | 1 | 124313 | 124314 | 0.00333333 | 4002.206386 | 4002.206389 |
| 4002.206459 | Kernel\_Read\|xilinx:adm-pcie-7v3:1ddr:3.0-0\|cnn\_GOOD | Read | OCL Region | 1 | 1 | 124335 | 124336 | 0.00333333 | 4002.206459 | 4002.206462 |
| 4002.206729 | Kernel\_Read\|xilinx:adm-pcie-7v3:1ddr:3.0-0\|cnn\_GOOD | Read | OCL Region | 1 | 1 | 124416 | 124417 | 0.00333333 | 4002.206729 | 4002.206732 |
| 4002.206736 | Kernel\_Read\|xilinx:adm-pcie-7v3:1ddr:3.0-0\|cnn\_GOOD | Read | OCL Region | 1 | 1 | 124418 | 124419 | 0.00333333 | 4002.206736 | 4002.206739 |
| 4002.206742 | Kernel\_Read\|xilinx:adm-pcie-7v3:1ddr:3.0-0\|cnn\_GOOD | Read | OCL Region | 1 | 1 | 124420 | 124421 | 0.00333333 | 4002.206742 | 4002.206746 |
| 4002.206746 | Kernel\_Read\|xilinx:adm-pcie-7v3:1ddr:3.0-0\|cnn\_GOOD | Read | OCL Region | 1 | 1 | 124421 | 124422 | 0.00333333 | 4002.206746 | 4002.206749 |
| 4002.206802 | Kernel\_Read\|xilinx:adm-pcie-7v3:1ddr:3.0-0\|cnn\_GOOD | Read | OCL Region | 1 | 1 | 124438 | 124439 | 0.00333333 | 4002.206802 | 4002.206806 |
| 4002.206862 | Kernel\_Read\|xilinx:adm-pcie-7v3:1ddr:3.0-0\|cnn\_GOOD | Read | OCL Region | 1 | 1 | 124456 | 124457 | 0.00333333 | 4002.206862 | 4002.206866 |
| 4002.206919 | Kernel\_Read\|xilinx:adm-pcie-7v3:1ddr:3.0-0\|cnn\_GOOD | Read | OCL Region | 1 | 1 | 124473 | 124474 | 0.00333333 | 4002.206919 | 4002.206922 |
| 4002.206986 | Kernel\_Read\|xilinx:adm-pcie-7v3:1ddr:3.0-0\|cnn\_GOOD | Read | OCL Region | 1 | 1 | 124493 | 124494 | 0.00333333 | 4002.206986 | 4002.206989 |
| 4002.207052 | Kernel\_Read\|xilinx:adm-pcie-7v3:1ddr:3.0-0\|cnn\_GOOD | Read | OCL Region | 1 | 1 | 124513 | 124514 | 0.00333333 | 4002.207052 | 4002.207056 |
| 4002.207119 | Kernel\_Read\|xilinx:adm-pcie-7v3:1ddr:3.0-0\|cnn\_GOOD | Read | OCL Region | 1 | 1 | 124533 | 124534 | 0.00333333 | 4002.207119 | 4002.207122 |
| 4002.207186 | Kernel\_Read\|xilinx:adm-pcie-7v3:1ddr:3.0-0\|cnn\_GOOD | Read | OCL Region | 1 | 1 | 124553 | 124554 | 0.00333333 | 4002.207186 | 4002.207189 |
| 4002.207259 | Kernel\_Read\|xilinx:adm-pcie-7v3:1ddr:3.0-0\|cnn\_GOOD | Read | OCL Region | 1 | 1 | 124575 | 124576 | 0.00333333 | 4002.207259 | 4002.207262 |
| 4002.207319 | Kernel\_Read\|xilinx:adm-pcie-7v3:1ddr:3.0-0\|cnn\_GOOD | Read | OCL Region | 1 | 1 | 124593 | 124594 | 0.00333333 | 4002.207319 | 4002.207322 |
| 4002.207386 | Kernel\_Read\|xilinx:adm-pcie-7v3:1ddr:3.0-0\|cnn\_GOOD | Read | OCL Region | 1 | 1 | 124613 | 124614 | 0.00333333 | 4002.207386 | 4002.207389 |
| 4002.207452 | Kernel\_Read\|xilinx:adm-pcie-7v3:1ddr:3.0-0\|cnn\_GOOD | Read | OCL Region | 1 | 1 | 124633 | 124634 | 0.00333333 | 4002.207452 | 4002.207456 |
| 4002.207519 | Kernel\_Read\|xilinx:adm-pcie-7v3:1ddr:3.0-0\|cnn\_GOOD | Read | OCL Region | 1 | 1 | 124653 | 124654 | 0.00333333 | 4002.207519 | 4002.207522 |
| 4002.207586 | Kernel\_Read\|xilinx:adm-pcie-7v3:1ddr:3.0-0\|cnn\_GOOD | Read | OCL Region | 1 | 1 | 124673 | 124674 | 0.00333333 | 4002.207586 | 4002.207589 |
| 4002.207652 | Kernel\_Read\|xilinx:adm-pcie-7v3:1ddr:3.0-0\|cnn\_GOOD | Read | OCL Region | 1 | 1 | 124693 | 124694 | 0.00333333 | 4002.207652 | 4002.207656 |
| 4002.207719 | Kernel\_Read\|xilinx:adm-pcie-7v3:1ddr:3.0-0\|cnn\_GOOD | Read | OCL Region | 1 | 1 | 124713 | 124714 | 0.00333333 | 4002.207719 | 4002.207722 |
| 4002.207786 | Kernel\_Read\|xilinx:adm-pcie-7v3:1ddr:3.0-0\|cnn\_GOOD | Read | OCL Region | 1 | 1 | 124733 | 124734 | 0.00333333 | 4002.207786 | 4002.207789 |
| 4002.207852 | Kernel\_Read\|xilinx:adm-pcie-7v3:1ddr:3.0-0\|cnn\_GOOD | Read | OCL Region | 1 | 1 | 124753 | 124754 | 0.00333333 | 4002.207852 | 4002.207856 |
| 4002.207919 | Kernel\_Read\|xilinx:adm-pcie-7v3:1ddr:3.0-0\|cnn\_GOOD | Read | OCL Region | 1 | 1 | 124773 | 124774 | 0.00333333 | 4002.207919 | 4002.207922 |
| 4002.207986 | Kernel\_Read\|xilinx:adm-pcie-7v3:1ddr:3.0-0\|cnn\_GOOD | Read | OCL Region | 1 | 1 | 124793 | 124794 | 0.00333333 | 4002.207986 | 4002.207989 |
| 4002.208059 | Kernel\_Read\|xilinx:adm-pcie-7v3:1ddr:3.0-0\|cnn\_GOOD | Read | OCL Region | 1 | 1 | 124815 | 124816 | 0.00333333 | 4002.208059 | 4002.208062 |
| 4002.208119 | Kernel\_Read\|xilinx:adm-pcie-7v3:1ddr:3.0-0\|cnn\_GOOD | Read | OCL Region | 1 | 1 | 124833 | 124834 | 0.00333333 | 4002.208119 | 4002.208122 |
| 4002.208186 | Kernel\_Read\|xilinx:adm-pcie-7v3:1ddr:3.0-0\|cnn\_GOOD | Read | OCL Region | 1 | 1 | 124853 | 124854 | 0.00333333 | 4002.208186 | 4002.208189 |
| 4002.208252 | Kernel\_Read\|xilinx:adm-pcie-7v3:1ddr:3.0-0\|cnn\_GOOD | Read | OCL Region | 1 | 1 | 124873 | 124874 | 0.00333333 | 4002.208252 | 4002.208256 |
| 4002.208319 | Kernel\_Read\|xilinx:adm-pcie-7v3:1ddr:3.0-0\|cnn\_GOOD | Read | OCL Region | 1 | 1 | 124893 | 124894 | 0.00333333 | 4002.208319 | 4002.208322 |
| 4002.208386 | Kernel\_Read\|xilinx:adm-pcie-7v3:1ddr:3.0-0\|cnn\_GOOD | Read | OCL Region | 1 | 1 | 124913 | 124914 | 0.00333333 | 4002.208386 | 4002.208389 |
| 4002.208452 | Kernel\_Read\|xilinx:adm-pcie-7v3:1ddr:3.0-0\|cnn\_GOOD | Read | OCL Region | 1 | 1 | 124933 | 124934 | 0.00333333 | 4002.208452 | 4002.208456 |
| 4002.208519 | Kernel\_Read\|xilinx:adm-pcie-7v3:1ddr:3.0-0\|cnn\_GOOD | Read | OCL Region | 1 | 1 | 124953 | 124954 | 0.00333333 | 4002.208519 | 4002.208522 |
| 4002.208586 | Kernel\_Read\|xilinx:adm-pcie-7v3:1ddr:3.0-0\|cnn\_GOOD | Read | OCL Region | 1 | 1 | 124973 | 124974 | 0.00333333 | 4002.208586 | 4002.208589 |
| 4002.208652 | Kernel\_Read\|xilinx:adm-pcie-7v3:1ddr:3.0-0\|cnn\_GOOD | Read | OCL Region | 1 | 1 | 124993 | 124994 | 0.00333333 | 4002.208652 | 4002.208656 |
| 4002.208719 | Kernel\_Read\|xilinx:adm-pcie-7v3:1ddr:3.0-0\|cnn\_GOOD | Read | OCL Region | 1 | 1 | 125013 | 125014 | 0.00333333 | 4002.208719 | 4002.208722 |
| 4002.208786 | Kernel\_Read\|xilinx:adm-pcie-7v3:1ddr:3.0-0\|cnn\_GOOD | Read | OCL Region | 1 | 1 | 125033 | 125034 | 0.00333333 | 4002.208786 | 4002.208789 |
| 4002.208852 | Kernel\_Read\|xilinx:adm-pcie-7v3:1ddr:3.0-0\|cnn\_GOOD | Read | OCL Region | 1 | 1 | 125053 | 125054 | 0.00333333 | 4002.208852 | 4002.208856 |
| 4002.208919 | Kernel\_Read\|xilinx:adm-pcie-7v3:1ddr:3.0-0\|cnn\_GOOD | Read | OCL Region | 1 | 1 | 125073 | 125074 | 0.00333333 | 4002.208919 | 4002.208922 |
| 4002.208986 | Kernel\_Read\|xilinx:adm-pcie-7v3:1ddr:3.0-0\|cnn\_GOOD | Read | OCL Region | 1 | 1 | 125093 | 125094 | 0.00333333 | 4002.208986 | 4002.208989 |
| 4002.209052 | Kernel\_Read\|xilinx:adm-pcie-7v3:1ddr:3.0-0\|cnn\_GOOD | Read | OCL Region | 1 | 1 | 125113 | 125114 | 0.00333333 | 4002.209052 | 4002.209056 |
| 4002.209132 | Kernel\_Read\|xilinx:adm-pcie-7v3:1ddr:3.0-0\|cnn\_GOOD | Read | OCL Region | 1 | 1 | 125137 | 125138 | 0.00333333 | 4002.209132 | 4002.209136 |
| 4002.209186 | Kernel\_Read\|xilinx:adm-pcie-7v3:1ddr:3.0-0\|cnn\_GOOD | Read | OCL Region | 1 | 1 | 125153 | 125154 | 0.00333333 | 4002.209186 | 4002.209189 |
| 4002.209252 | Kernel\_Read\|xilinx:adm-pcie-7v3:1ddr:3.0-0\|cnn\_GOOD | Read | OCL Region | 1 | 1 | 125173 | 125174 | 0.00333333 | 4002.209252 | 4002.209256 |
| 4002.209319 | Kernel\_Read\|xilinx:adm-pcie-7v3:1ddr:3.0-0\|cnn\_GOOD | Read | OCL Region | 1 | 1 | 125193 | 125194 | 0.00333333 | 4002.209319 | 4002.209322 |
| 4002.209386 | Kernel\_Read\|xilinx:adm-pcie-7v3:1ddr:3.0-0\|cnn\_GOOD | Read | OCL Region | 1 | 1 | 125213 | 125214 | 0.00333333 | 4002.209386 | 4002.209389 |
| 4002.209452 | Kernel\_Read\|xilinx:adm-pcie-7v3:1ddr:3.0-0\|cnn\_GOOD | Read | OCL Region | 1 | 1 | 125233 | 125234 | 0.00333333 | 4002.209452 | 4002.209456 |
| 4002.209519 | Kernel\_Read\|xilinx:adm-pcie-7v3:1ddr:3.0-0\|cnn\_GOOD | Read | OCL Region | 1 | 1 | 125253 | 125254 | 0.00333333 | 4002.209519 | 4002.209522 |
| 4002.209586 | Kernel\_Read\|xilinx:adm-pcie-7v3:1ddr:3.0-0\|cnn\_GOOD | Read | OCL Region | 1 | 1 | 125273 | 125274 | 0.00333333 | 4002.209586 | 4002.209589 |
| 4002.209659 | Kernel\_Read\|xilinx:adm-pcie-7v3:1ddr:3.0-0\|cnn\_GOOD | Read | OCL Region | 1 | 1 | 125295 | 125296 | 0.00333333 | 4002.209659 | 4002.209662 |
| 4002.209719 | Kernel\_Read\|xilinx:adm-pcie-7v3:1ddr:3.0-0\|cnn\_GOOD | Read | OCL Region | 1 | 1 | 125313 | 125314 | 0.00333333 | 4002.209719 | 4002.209722 |
| 4002.209786 | Kernel\_Read\|xilinx:adm-pcie-7v3:1ddr:3.0-0\|cnn\_GOOD | Read | OCL Region | 1 | 1 | 125333 | 125334 | 0.00333333 | 4002.209786 | 4002.209789 |
| 4002.209852 | Kernel\_Read\|xilinx:adm-pcie-7v3:1ddr:3.0-0\|cnn\_GOOD | Read | OCL Region | 1 | 1 | 125353 | 125354 | 0.00333333 | 4002.209852 | 4002.209856 |
| 4002.209919 | Kernel\_Read\|xilinx:adm-pcie-7v3:1ddr:3.0-0\|cnn\_GOOD | Read | OCL Region | 1 | 1 | 125373 | 125374 | 0.00333333 | 4002.209919 | 4002.209922 |
| 4002.209992 | Kernel\_Read\|xilinx:adm-pcie-7v3:1ddr:3.0-0\|cnn\_GOOD | Read | OCL Region | 1 | 1 | 125395 | 125396 | 0.00333333 | 4002.209992 | 4002.209996 |
| 4002.210052 | Kernel\_Read\|xilinx:adm-pcie-7v3:1ddr:3.0-0\|cnn\_GOOD | Read | OCL Region | 1 | 1 | 125413 | 125414 | 0.00333333 | 4002.210052 | 4002.210056 |
| 4002.210119 | Kernel\_Read\|xilinx:adm-pcie-7v3:1ddr:3.0-0\|cnn\_GOOD | Read | OCL Region | 1 | 1 | 125433 | 125434 | 0.00333333 | 4002.210119 | 4002.210122 |
| 4002.210186 | Kernel\_Read\|xilinx:adm-pcie-7v3:1ddr:3.0-0\|cnn\_GOOD | Read | OCL Region | 1 | 1 | 125453 | 125454 | 0.00333333 | 4002.210186 | 4002.210189 |
| 4002.210252 | Kernel\_Read\|xilinx:adm-pcie-7v3:1ddr:3.0-0\|cnn\_GOOD | Read | OCL Region | 1 | 1 | 125473 | 125474 | 0.00333333 | 4002.210252 | 4002.210256 |
| 4002.210319 | Kernel\_Read\|xilinx:adm-pcie-7v3:1ddr:3.0-0\|cnn\_GOOD | Read | OCL Region | 1 | 1 | 125493 | 125494 | 0.00333333 | 4002.210319 | 4002.210322 |
| 4002.210386 | Kernel\_Read\|xilinx:adm-pcie-7v3:1ddr:3.0-0\|cnn\_GOOD | Read | OCL Region | 1 | 1 | 125513 | 125514 | 0.00333333 | 4002.210386 | 4002.210389 |
| 4002.210452 | Kernel\_Read\|xilinx:adm-pcie-7v3:1ddr:3.0-0\|cnn\_GOOD | Read | OCL Region | 1 | 1 | 125533 | 125534 | 0.00333333 | 4002.210452 | 4002.210456 |
| 4002.210519 | Kernel\_Read\|xilinx:adm-pcie-7v3:1ddr:3.0-0\|cnn\_GOOD | Read | OCL Region | 1 | 1 | 125553 | 125554 | 0.00333333 | 4002.210519 | 4002.210522 |
| 4002.210586 | Kernel\_Read\|xilinx:adm-pcie-7v3:1ddr:3.0-0\|cnn\_GOOD | Read | OCL Region | 1 | 1 | 125573 | 125574 | 0.00333333 | 4002.210586 | 4002.210589 |
| 4002.210652 | Kernel\_Read\|xilinx:adm-pcie-7v3:1ddr:3.0-0\|cnn\_GOOD | Read | OCL Region | 1 | 1 | 125593 | 125594 | 0.00333333 | 4002.210652 | 4002.210656 |
| 4002.210719 | Kernel\_Read\|xilinx:adm-pcie-7v3:1ddr:3.0-0\|cnn\_GOOD | Read | OCL Region | 1 | 1 | 125613 | 125614 | 0.00333333 | 4002.210719 | 4002.210722 |
| 4002.210786 | Kernel\_Read\|xilinx:adm-pcie-7v3:1ddr:3.0-0\|cnn\_GOOD | Read | OCL Region | 1 | 1 | 125633 | 125634 | 0.00333333 | 4002.210786 | 4002.210789 |
| 4002.210852 | Kernel\_Read\|xilinx:adm-pcie-7v3:1ddr:3.0-0\|cnn\_GOOD | Read | OCL Region | 1 | 1 | 125653 | 125654 | 0.00333333 | 4002.210852 | 4002.210856 |
| 4002.210919 | Kernel\_Read\|xilinx:adm-pcie-7v3:1ddr:3.0-0\|cnn\_GOOD | Read | OCL Region | 1 | 1 | 125673 | 125674 | 0.00333333 | 4002.210919 | 4002.210922 |
| 4002.210986 | Kernel\_Read\|xilinx:adm-pcie-7v3:1ddr:3.0-0\|cnn\_GOOD | Read | OCL Region | 1 | 1 | 125693 | 125694 | 0.00333333 | 4002.210986 | 4002.210989 |
| 4002.211052 | Kernel\_Read\|xilinx:adm-pcie-7v3:1ddr:3.0-0\|cnn\_GOOD | Read | OCL Region | 1 | 1 | 125713 | 125714 | 0.00333333 | 4002.211052 | 4002.211056 |
| 4002.211119 | Kernel\_Read\|xilinx:adm-pcie-7v3:1ddr:3.0-0\|cnn\_GOOD | Read | OCL Region | 1 | 1 | 125733 | 125734 | 0.00333333 | 4002.211119 | 4002.211122 |
| 4002.211186 | Kernel\_Read\|xilinx:adm-pcie-7v3:1ddr:3.0-0\|cnn\_GOOD | Read | OCL Region | 1 | 1 | 125753 | 125754 | 0.00333333 | 4002.211186 | 4002.211189 |
| 4002.211259 | Kernel\_Read\|xilinx:adm-pcie-7v3:1ddr:3.0-0\|cnn\_GOOD | Read | OCL Region | 1 | 1 | 125775 | 125776 | 0.00333333 | 4002.211259 | 4002.211262 |
| 4002.211319 | Kernel\_Read\|xilinx:adm-pcie-7v3:1ddr:3.0-0\|cnn\_GOOD | Read | OCL Region | 1 | 1 | 125793 | 125794 | 0.00333333 | 4002.211319 | 4002.211322 |
| 4002.211386 | Kernel\_Read\|xilinx:adm-pcie-7v3:1ddr:3.0-0\|cnn\_GOOD | Read | OCL Region | 1 | 1 | 125813 | 125814 | 0.00333333 | 4002.211386 | 4002.211389 |
| 4002.211452 | Kernel\_Read\|xilinx:adm-pcie-7v3:1ddr:3.0-0\|cnn\_GOOD | Read | OCL Region | 1 | 1 | 125833 | 125834 | 0.00333333 | 4002.211452 | 4002.211456 |
| 4002.211519 | Kernel\_Read\|xilinx:adm-pcie-7v3:1ddr:3.0-0\|cnn\_GOOD | Read | OCL Region | 1 | 1 | 125853 | 125854 | 0.00333333 | 4002.211519 | 4002.211522 |
| 4002.211586 | Kernel\_Read\|xilinx:adm-pcie-7v3:1ddr:3.0-0\|cnn\_GOOD | Read | OCL Region | 1 | 1 | 125873 | 125874 | 0.00333333 | 4002.211586 | 4002.211589 |
| 4002.211652 | Kernel\_Read\|xilinx:adm-pcie-7v3:1ddr:3.0-0\|cnn\_GOOD | Read | OCL Region | 1 | 1 | 125893 | 125894 | 0.00333333 | 4002.211652 | 4002.211656 |
| 4002.211719 | Kernel\_Read\|xilinx:adm-pcie-7v3:1ddr:3.0-0\|cnn\_GOOD | Read | OCL Region | 1 | 1 | 125913 | 125914 | 0.00333333 | 4002.211719 | 4002.211722 |
| 4002.211786 | Kernel\_Read\|xilinx:adm-pcie-7v3:1ddr:3.0-0\|cnn\_GOOD | Read | OCL Region | 1 | 1 | 125933 | 125934 | 0.00333333 | 4002.211786 | 4002.211789 |
| 4002.211852 | Kernel\_Read\|xilinx:adm-pcie-7v3:1ddr:3.0-0\|cnn\_GOOD | Read | OCL Region | 1 | 1 | 125953 | 125954 | 0.00333333 | 4002.211852 | 4002.211856 |
| 4002.211919 | Kernel\_Read\|xilinx:adm-pcie-7v3:1ddr:3.0-0\|cnn\_GOOD | Read | OCL Region | 1 | 1 | 125973 | 125974 | 0.00333333 | 4002.211919 | 4002.211922 |
| 4002.211986 | Kernel\_Read\|xilinx:adm-pcie-7v3:1ddr:3.0-0\|cnn\_GOOD | Read | OCL Region | 1 | 1 | 125993 | 125994 | 0.00333333 | 4002.211986 | 4002.211989 |
| 4002.212052 | Kernel\_Read\|xilinx:adm-pcie-7v3:1ddr:3.0-0\|cnn\_GOOD | Read | OCL Region | 1 | 1 | 126013 | 126014 | 0.00333333 | 4002.212052 | 4002.212056 |
| 4002.212119 | Kernel\_Read\|xilinx:adm-pcie-7v3:1ddr:3.0-0\|cnn\_GOOD | Read | OCL Region | 1 | 1 | 126033 | 126034 | 0.00333333 | 4002.212119 | 4002.212122 |
| 4002.212186 | Kernel\_Read\|xilinx:adm-pcie-7v3:1ddr:3.0-0\|cnn\_GOOD | Read | OCL Region | 1 | 1 | 126053 | 126054 | 0.00333333 | 4002.212186 | 4002.212189 |
| 4002.212252 | Kernel\_Read\|xilinx:adm-pcie-7v3:1ddr:3.0-0\|cnn\_GOOD | Read | OCL Region | 1 | 1 | 126073 | 126074 | 0.00333333 | 4002.212252 | 4002.212256 |
| 4002.212319 | Kernel\_Read\|xilinx:adm-pcie-7v3:1ddr:3.0-0\|cnn\_GOOD | Read | OCL Region | 1 | 1 | 126093 | 126094 | 0.00333333 | 4002.212319 | 4002.212322 |
| 4002.212386 | Kernel\_Read\|xilinx:adm-pcie-7v3:1ddr:3.0-0\|cnn\_GOOD | Read | OCL Region | 1 | 1 | 126113 | 126114 | 0.00333333 | 4002.212386 | 4002.212389 |
| 4002.212466 | Kernel\_Read\|xilinx:adm-pcie-7v3:1ddr:3.0-0\|cnn\_GOOD | Read | OCL Region | 1 | 1 | 126137 | 126138 | 0.00333333 | 4002.212466 | 4002.212469 |
| 4002.212519 | Kernel\_Read\|xilinx:adm-pcie-7v3:1ddr:3.0-0\|cnn\_GOOD | Read | OCL Region | 1 | 1 | 126153 | 126154 | 0.00333333 | 4002.212519 | 4002.212522 |
| 4002.212586 | Kernel\_Read\|xilinx:adm-pcie-7v3:1ddr:3.0-0\|cnn\_GOOD | Read | OCL Region | 1 | 1 | 126173 | 126174 | 0.00333333 | 4002.212586 | 4002.212589 |
| 4002.212652 | Kernel\_Read\|xilinx:adm-pcie-7v3:1ddr:3.0-0\|cnn\_GOOD | Read | OCL Region | 1 | 1 | 126193 | 126194 | 0.00333333 | 4002.212652 | 4002.212656 |
| 4002.212719 | Kernel\_Read\|xilinx:adm-pcie-7v3:1ddr:3.0-0\|cnn\_GOOD | Read | OCL Region | 1 | 1 | 126213 | 126214 | 0.00333333 | 4002.212719 | 4002.212722 |
| 4002.212996 | Kernel\_Read\|xilinx:adm-pcie-7v3:1ddr:3.0-0\|cnn\_GOOD | Read | OCL Region | 1 | 1 | 126296 | 126297 | 0.00333333 | 4002.212996 | 4002.212999 |
| 4002.212999 | Kernel\_Read\|xilinx:adm-pcie-7v3:1ddr:3.0-0\|cnn\_GOOD | Read | OCL Region | 1 | 1 | 126297 | 126298 | 0.00333333 | 4002.212999 | 4002.213002 |
| 4002.213002 | Kernel\_Read\|xilinx:adm-pcie-7v3:1ddr:3.0-0\|cnn\_GOOD | Read | OCL Region | 1 | 1 | 126298 | 126299 | 0.00333333 | 4002.213002 | 4002.213006 |
| 4002.213012 | Kernel\_Read\|xilinx:adm-pcie-7v3:1ddr:3.0-0\|cnn\_GOOD | Read | OCL Region | 1 | 1 | 126301 | 126302 | 0.00333333 | 4002.213012 | 4002.213016 |
| 4002.213069 | Kernel\_Read\|xilinx:adm-pcie-7v3:1ddr:3.0-0\|cnn\_GOOD | Read | OCL Region | 1 | 1 | 126318 | 126319 | 0.00333333 | 4002.213069 | 4002.213072 |
| 4002.213126 | Kernel\_Read\|xilinx:adm-pcie-7v3:1ddr:3.0-0\|cnn\_GOOD | Read | OCL Region | 1 | 1 | 126335 | 126336 | 0.00333333 | 4002.213126 | 4002.213129 |
| 4002.213186 | Kernel\_Read\|xilinx:adm-pcie-7v3:1ddr:3.0-0\|cnn\_GOOD | Read | OCL Region | 1 | 1 | 126353 | 126354 | 0.00333333 | 4002.213186 | 4002.213189 |
| 4002.213252 | Kernel\_Read\|xilinx:adm-pcie-7v3:1ddr:3.0-0\|cnn\_GOOD | Read | OCL Region | 1 | 1 | 126373 | 126374 | 0.00333333 | 4002.213252 | 4002.213256 |
| 4002.213319 | Kernel\_Read\|xilinx:adm-pcie-7v3:1ddr:3.0-0\|cnn\_GOOD | Read | OCL Region | 1 | 1 | 126393 | 126394 | 0.00333333 | 4002.213319 | 4002.213322 |
| 4002.213386 | Kernel\_Read\|xilinx:adm-pcie-7v3:1ddr:3.0-0\|cnn\_GOOD | Read | OCL Region | 1 | 1 | 126413 | 126414 | 0.00333333 | 4002.213386 | 4002.213389 |
| 4002.213452 | Kernel\_Read\|xilinx:adm-pcie-7v3:1ddr:3.0-0\|cnn\_GOOD | Read | OCL Region | 1 | 1 | 126433 | 126434 | 0.00333333 | 4002.213452 | 4002.213456 |
| 4002.213519 | Kernel\_Read\|xilinx:adm-pcie-7v3:1ddr:3.0-0\|cnn\_GOOD | Read | OCL Region | 1 | 1 | 126453 | 126454 | 0.00333333 | 4002.213519 | 4002.213522 |
| 4002.213586 | Kernel\_Read\|xilinx:adm-pcie-7v3:1ddr:3.0-0\|cnn\_GOOD | Read | OCL Region | 1 | 1 | 126473 | 126474 | 0.00333333 | 4002.213586 | 4002.213589 |
| 4002.213652 | Kernel\_Read\|xilinx:adm-pcie-7v3:1ddr:3.0-0\|cnn\_GOOD | Read | OCL Region | 1 | 1 | 126493 | 126494 | 0.00333333 | 4002.213652 | 4002.213656 |
| 4002.213719 | Kernel\_Read\|xilinx:adm-pcie-7v3:1ddr:3.0-0\|cnn\_GOOD | Read | OCL Region | 1 | 1 | 126513 | 126514 | 0.00333333 | 4002.213719 | 4002.213722 |
| 4002.213786 | Kernel\_Read\|xilinx:adm-pcie-7v3:1ddr:3.0-0\|cnn\_GOOD | Read | OCL Region | 1 | 1 | 126533 | 126534 | 0.00333333 | 4002.213786 | 4002.213789 |
| 4002.213852 | Kernel\_Read\|xilinx:adm-pcie-7v3:1ddr:3.0-0\|cnn\_GOOD | Read | OCL Region | 1 | 1 | 126553 | 126554 | 0.00333333 | 4002.213852 | 4002.213856 |
| 4002.213919 | Kernel\_Read\|xilinx:adm-pcie-7v3:1ddr:3.0-0\|cnn\_GOOD | Read | OCL Region | 1 | 1 | 126573 | 126574 | 0.00333333 | 4002.213919 | 4002.213922 |
| 4002.213986 | Kernel\_Read\|xilinx:adm-pcie-7v3:1ddr:3.0-0\|cnn\_GOOD | Read | OCL Region | 1 | 1 | 126593 | 126594 | 0.00333333 | 4002.213986 | 4002.213989 |
| 4002.214052 | Kernel\_Read\|xilinx:adm-pcie-7v3:1ddr:3.0-0\|cnn\_GOOD | Read | OCL Region | 1 | 1 | 126613 | 126614 | 0.00333333 | 4002.214052 | 4002.214056 |
| 4002.214119 | Kernel\_Read\|xilinx:adm-pcie-7v3:1ddr:3.0-0\|cnn\_GOOD | Read | OCL Region | 1 | 1 | 126633 | 126634 | 0.00333333 | 4002.214119 | 4002.214122 |
| 4002.214186 | Kernel\_Read\|xilinx:adm-pcie-7v3:1ddr:3.0-0\|cnn\_GOOD | Read | OCL Region | 1 | 1 | 126653 | 126654 | 0.00333333 | 4002.214186 | 4002.214189 |
| 4002.214252 | Kernel\_Read\|xilinx:adm-pcie-7v3:1ddr:3.0-0\|cnn\_GOOD | Read | OCL Region | 1 | 1 | 126673 | 126674 | 0.00333333 | 4002.214252 | 4002.214256 |
| 4002.214319 | Kernel\_Read\|xilinx:adm-pcie-7v3:1ddr:3.0-0\|cnn\_GOOD | Read | OCL Region | 1 | 1 | 126693 | 126694 | 0.00333333 | 4002.214319 | 4002.214322 |
| 4002.214386 | Kernel\_Read\|xilinx:adm-pcie-7v3:1ddr:3.0-0\|cnn\_GOOD | Read | OCL Region | 1 | 1 | 126713 | 126714 | 0.00333333 | 4002.214386 | 4002.214389 |
| 4002.214452 | Kernel\_Read\|xilinx:adm-pcie-7v3:1ddr:3.0-0\|cnn\_GOOD | Read | OCL Region | 1 | 1 | 126733 | 126734 | 0.00333333 | 4002.214452 | 4002.214456 |
| 4002.214519 | Kernel\_Read\|xilinx:adm-pcie-7v3:1ddr:3.0-0\|cnn\_GOOD | Read | OCL Region | 1 | 1 | 126753 | 126754 | 0.00333333 | 4002.214519 | 4002.214522 |
| 4002.214586 | Kernel\_Read\|xilinx:adm-pcie-7v3:1ddr:3.0-0\|cnn\_GOOD | Read | OCL Region | 1 | 1 | 126773 | 126774 | 0.00333333 | 4002.214586 | 4002.214589 |
| 4002.214652 | Kernel\_Read\|xilinx:adm-pcie-7v3:1ddr:3.0-0\|cnn\_GOOD | Read | OCL Region | 1 | 1 | 126793 | 126794 | 0.00333333 | 4002.214652 | 4002.214656 |
| 4002.214719 | Kernel\_Read\|xilinx:adm-pcie-7v3:1ddr:3.0-0\|cnn\_GOOD | Read | OCL Region | 1 | 1 | 126813 | 126814 | 0.00333333 | 4002.214719 | 4002.214722 |
| 4002.214786 | Kernel\_Read\|xilinx:adm-pcie-7v3:1ddr:3.0-0\|cnn\_GOOD | Read | OCL Region | 1 | 1 | 126833 | 126834 | 0.00333333 | 4002.214786 | 4002.214789 |
| 4002.214852 | Kernel\_Read\|xilinx:adm-pcie-7v3:1ddr:3.0-0\|cnn\_GOOD | Read | OCL Region | 1 | 1 | 126853 | 126854 | 0.00333333 | 4002.214852 | 4002.214856 |
| 4002.214919 | Kernel\_Read\|xilinx:adm-pcie-7v3:1ddr:3.0-0\|cnn\_GOOD | Read | OCL Region | 1 | 1 | 126873 | 126874 | 0.00333333 | 4002.214919 | 4002.214922 |
| 4002.214986 | Kernel\_Read\|xilinx:adm-pcie-7v3:1ddr:3.0-0\|cnn\_GOOD | Read | OCL Region | 1 | 1 | 126893 | 126894 | 0.00333333 | 4002.214986 | 4002.214989 |
| 4002.215052 | Kernel\_Read\|xilinx:adm-pcie-7v3:1ddr:3.0-0\|cnn\_GOOD | Read | OCL Region | 1 | 1 | 126913 | 126914 | 0.00333333 | 4002.215052 | 4002.215056 |
| 4002.215119 | Kernel\_Read\|xilinx:adm-pcie-7v3:1ddr:3.0-0\|cnn\_GOOD | Read | OCL Region | 1 | 1 | 126933 | 126934 | 0.00333333 | 4002.215119 | 4002.215122 |
| 4002.215186 | Kernel\_Read\|xilinx:adm-pcie-7v3:1ddr:3.0-0\|cnn\_GOOD | Read | OCL Region | 1 | 1 | 126953 | 126954 | 0.00333333 | 4002.215186 | 4002.215189 |
| 4002.215252 | Kernel\_Read\|xilinx:adm-pcie-7v3:1ddr:3.0-0\|cnn\_GOOD | Read | OCL Region | 1 | 1 | 126973 | 126974 | 0.00333333 | 4002.215252 | 4002.215256 |
| 4002.215319 | Kernel\_Read\|xilinx:adm-pcie-7v3:1ddr:3.0-0\|cnn\_GOOD | Read | OCL Region | 1 | 1 | 126993 | 126994 | 0.00333333 | 4002.215319 | 4002.215322 |
| 4002.215386 | Kernel\_Read\|xilinx:adm-pcie-7v3:1ddr:3.0-0\|cnn\_GOOD | Read | OCL Region | 1 | 1 | 127013 | 127014 | 0.00333333 | 4002.215386 | 4002.215389 |
| 4002.215452 | Kernel\_Read\|xilinx:adm-pcie-7v3:1ddr:3.0-0\|cnn\_GOOD | Read | OCL Region | 1 | 1 | 127033 | 127034 | 0.00333333 | 4002.215452 | 4002.215456 |
| 4002.215519 | Kernel\_Read\|xilinx:adm-pcie-7v3:1ddr:3.0-0\|cnn\_GOOD | Read | OCL Region | 1 | 1 | 127053 | 127054 | 0.00333333 | 4002.215519 | 4002.215522 |
| 4002.215586 | Kernel\_Read\|xilinx:adm-pcie-7v3:1ddr:3.0-0\|cnn\_GOOD | Read | OCL Region | 1 | 1 | 127073 | 127074 | 0.00333333 | 4002.215586 | 4002.215589 |
| 4002.215652 | Kernel\_Read\|xilinx:adm-pcie-7v3:1ddr:3.0-0\|cnn\_GOOD | Read | OCL Region | 1 | 1 | 127093 | 127094 | 0.00333333 | 4002.215652 | 4002.215656 |
| 4002.215719 | Kernel\_Read\|xilinx:adm-pcie-7v3:1ddr:3.0-0\|cnn\_GOOD | Read | OCL Region | 1 | 1 | 127113 | 127114 | 0.00333333 | 4002.215719 | 4002.215722 |
| 4002.215786 | Kernel\_Read\|xilinx:adm-pcie-7v3:1ddr:3.0-0\|cnn\_GOOD | Read | OCL Region | 1 | 1 | 127133 | 127134 | 0.00333333 | 4002.215786 | 4002.215789 |
| 4002.215852 | Kernel\_Read\|xilinx:adm-pcie-7v3:1ddr:3.0-0\|cnn\_GOOD | Read | OCL Region | 1 | 1 | 127153 | 127154 | 0.00333333 | 4002.215852 | 4002.215856 |
| 4002.215919 | Kernel\_Read\|xilinx:adm-pcie-7v3:1ddr:3.0-0\|cnn\_GOOD | Read | OCL Region | 1 | 1 | 127173 | 127174 | 0.00333333 | 4002.215919 | 4002.215922 |
| 4002.215986 | Kernel\_Read\|xilinx:adm-pcie-7v3:1ddr:3.0-0\|cnn\_GOOD | Read | OCL Region | 1 | 1 | 127193 | 127194 | 0.00333333 | 4002.215986 | 4002.215989 |
| 4002.216059 | Kernel\_Read\|xilinx:adm-pcie-7v3:1ddr:3.0-0\|cnn\_GOOD | Read | OCL Region | 1 | 1 | 127215 | 127216 | 0.00333333 | 4002.216059 | 4002.216062 |
| 4002.216119 | Kernel\_Read\|xilinx:adm-pcie-7v3:1ddr:3.0-0\|cnn\_GOOD | Read | OCL Region | 1 | 1 | 127233 | 127234 | 0.00333333 | 4002.216119 | 4002.216122 |
| 4002.216186 | Kernel\_Read\|xilinx:adm-pcie-7v3:1ddr:3.0-0\|cnn\_GOOD | Read | OCL Region | 1 | 1 | 127253 | 127254 | 0.00333333 | 4002.216186 | 4002.216189 |
| 4002.216262 | Kernel\_Read\|xilinx:adm-pcie-7v3:1ddr:3.0-0\|cnn\_GOOD | Read | OCL Region | 1 | 1 | 127276 | 127277 | 0.00333333 | 4002.216262 | 4002.216266 |
| 4002.216319 | Kernel\_Read\|xilinx:adm-pcie-7v3:1ddr:3.0-0\|cnn\_GOOD | Read | OCL Region | 1 | 1 | 127293 | 127294 | 0.00333333 | 4002.216319 | 4002.216322 |
| 4002.216386 | Kernel\_Read\|xilinx:adm-pcie-7v3:1ddr:3.0-0\|cnn\_GOOD | Read | OCL Region | 1 | 1 | 127313 | 127314 | 0.00333333 | 4002.216386 | 4002.216389 |
| 4002.216452 | Kernel\_Read\|xilinx:adm-pcie-7v3:1ddr:3.0-0\|cnn\_GOOD | Read | OCL Region | 1 | 1 | 127333 | 127334 | 0.00333333 | 4002.216452 | 4002.216456 |
| 4002.216519 | Kernel\_Read\|xilinx:adm-pcie-7v3:1ddr:3.0-0\|cnn\_GOOD | Read | OCL Region | 1 | 1 | 127353 | 127354 | 0.00333333 | 4002.216519 | 4002.216522 |
| 4002.216586 | Kernel\_Read\|xilinx:adm-pcie-7v3:1ddr:3.0-0\|cnn\_GOOD | Read | OCL Region | 1 | 1 | 127373 | 127374 | 0.00333333 | 4002.216586 | 4002.216589 |
| 4002.216652 | Kernel\_Read\|xilinx:adm-pcie-7v3:1ddr:3.0-0\|cnn\_GOOD | Read | OCL Region | 1 | 1 | 127393 | 127394 | 0.00333333 | 4002.216652 | 4002.216656 |
| 4002.216719 | Kernel\_Read\|xilinx:adm-pcie-7v3:1ddr:3.0-0\|cnn\_GOOD | Read | OCL Region | 1 | 1 | 127413 | 127414 | 0.00333333 | 4002.216719 | 4002.216722 |
| 4002.216786 | Kernel\_Read\|xilinx:adm-pcie-7v3:1ddr:3.0-0\|cnn\_GOOD | Read | OCL Region | 1 | 1 | 127433 | 127434 | 0.00333333 | 4002.216786 | 4002.216789 |
| 4002.216852 | Kernel\_Read\|xilinx:adm-pcie-7v3:1ddr:3.0-0\|cnn\_GOOD | Read | OCL Region | 1 | 1 | 127453 | 127454 | 0.00333333 | 4002.216852 | 4002.216856 |
| 4002.216919 | Kernel\_Read\|xilinx:adm-pcie-7v3:1ddr:3.0-0\|cnn\_GOOD | Read | OCL Region | 1 | 1 | 127473 | 127474 | 0.00333333 | 4002.216919 | 4002.216922 |
| 4002.216986 | Kernel\_Read\|xilinx:adm-pcie-7v3:1ddr:3.0-0\|cnn\_GOOD | Read | OCL Region | 1 | 1 | 127493 | 127494 | 0.00333333 | 4002.216986 | 4002.216989 |
| 4002.217049 | Kernel\_Read\|xilinx:adm-pcie-7v3:1ddr:3.0-0\|cnn\_GOOD | Read | OCL Region | 1 | 1 | 127512 | 127513 | 0.00333333 | 4002.217049 | 4002.217052 |
| 4002.217119 | Kernel\_Read\|xilinx:adm-pcie-7v3:1ddr:3.0-0\|cnn\_GOOD | Read | OCL Region | 1 | 1 | 127533 | 127534 | 0.00333333 | 4002.217119 | 4002.217122 |
| 4002.217186 | Kernel\_Read\|xilinx:adm-pcie-7v3:1ddr:3.0-0\|cnn\_GOOD | Read | OCL Region | 1 | 1 | 127553 | 127554 | 0.00333333 | 4002.217186 | 4002.217189 |
| 4002.217252 | Kernel\_Read\|xilinx:adm-pcie-7v3:1ddr:3.0-0\|cnn\_GOOD | Read | OCL Region | 1 | 1 | 127573 | 127574 | 0.00333333 | 4002.217252 | 4002.217256 |
| 4002.217319 | Kernel\_Read\|xilinx:adm-pcie-7v3:1ddr:3.0-0\|cnn\_GOOD | Read | OCL Region | 1 | 1 | 127593 | 127594 | 0.00333333 | 4002.217319 | 4002.217322 |
| 4002.217386 | Kernel\_Read\|xilinx:adm-pcie-7v3:1ddr:3.0-0\|cnn\_GOOD | Read | OCL Region | 1 | 1 | 127613 | 127614 | 0.00333333 | 4002.217386 | 4002.217389 |
| 4002.217452 | Kernel\_Read\|xilinx:adm-pcie-7v3:1ddr:3.0-0\|cnn\_GOOD | Read | OCL Region | 1 | 1 | 127633 | 127634 | 0.00333333 | 4002.217452 | 4002.217456 |
| 4002.217519 | Kernel\_Read\|xilinx:adm-pcie-7v3:1ddr:3.0-0\|cnn\_GOOD | Read | OCL Region | 1 | 1 | 127653 | 127654 | 0.00333333 | 4002.217519 | 4002.217522 |
| 4002.217586 | Kernel\_Read\|xilinx:adm-pcie-7v3:1ddr:3.0-0\|cnn\_GOOD | Read | OCL Region | 1 | 1 | 127673 | 127674 | 0.00333333 | 4002.217586 | 4002.217589 |
| 4002.217659 | Kernel\_Read\|xilinx:adm-pcie-7v3:1ddr:3.0-0\|cnn\_GOOD | Read | OCL Region | 1 | 1 | 127695 | 127696 | 0.00333333 | 4002.217659 | 4002.217662 |
| 4002.217719 | Kernel\_Read\|xilinx:adm-pcie-7v3:1ddr:3.0-0\|cnn\_GOOD | Read | OCL Region | 1 | 1 | 127713 | 127714 | 0.00333333 | 4002.217719 | 4002.217722 |
| 4002.217786 | Kernel\_Read\|xilinx:adm-pcie-7v3:1ddr:3.0-0\|cnn\_GOOD | Read | OCL Region | 1 | 1 | 127733 | 127734 | 0.00333333 | 4002.217786 | 4002.217789 |
| 4002.217852 | Kernel\_Read\|xilinx:adm-pcie-7v3:1ddr:3.0-0\|cnn\_GOOD | Read | OCL Region | 1 | 1 | 127753 | 127754 | 0.00333333 | 4002.217852 | 4002.217856 |
| 4002.217919 | Kernel\_Read\|xilinx:adm-pcie-7v3:1ddr:3.0-0\|cnn\_GOOD | Read | OCL Region | 1 | 1 | 127773 | 127774 | 0.00333333 | 4002.217919 | 4002.217922 |
| 4002.217986 | Kernel\_Read\|xilinx:adm-pcie-7v3:1ddr:3.0-0\|cnn\_GOOD | Read | OCL Region | 1 | 1 | 127793 | 127794 | 0.00333333 | 4002.217986 | 4002.217989 |
| 4002.218052 | Kernel\_Read\|xilinx:adm-pcie-7v3:1ddr:3.0-0\|cnn\_GOOD | Read | OCL Region | 1 | 1 | 127813 | 127814 | 0.00333333 | 4002.218052 | 4002.218056 |
| 4002.218119 | Kernel\_Read\|xilinx:adm-pcie-7v3:1ddr:3.0-0\|cnn\_GOOD | Read | OCL Region | 1 | 1 | 127833 | 127834 | 0.00333333 | 4002.218119 | 4002.218122 |
| 4002.218186 | Kernel\_Read\|xilinx:adm-pcie-7v3:1ddr:3.0-0\|cnn\_GOOD | Read | OCL Region | 1 | 1 | 127853 | 127854 | 0.00333333 | 4002.218186 | 4002.218189 |
| 4002.218252 | Kernel\_Read\|xilinx:adm-pcie-7v3:1ddr:3.0-0\|cnn\_GOOD | Read | OCL Region | 1 | 1 | 127873 | 127874 | 0.00333333 | 4002.218252 | 4002.218256 |
| 4002.218319 | Kernel\_Read\|xilinx:adm-pcie-7v3:1ddr:3.0-0\|cnn\_GOOD | Read | OCL Region | 1 | 1 | 127893 | 127894 | 0.00333333 | 4002.218319 | 4002.218322 |
| 4002.218386 | Kernel\_Read\|xilinx:adm-pcie-7v3:1ddr:3.0-0\|cnn\_GOOD | Read | OCL Region | 1 | 1 | 127913 | 127914 | 0.00333333 | 4002.218386 | 4002.218389 |
| 4002.218452 | Kernel\_Read\|xilinx:adm-pcie-7v3:1ddr:3.0-0\|cnn\_GOOD | Read | OCL Region | 1 | 1 | 127933 | 127934 | 0.00333333 | 4002.218452 | 4002.218456 |
| 4002.218519 | Kernel\_Read\|xilinx:adm-pcie-7v3:1ddr:3.0-0\|cnn\_GOOD | Read | OCL Region | 1 | 1 | 127953 | 127954 | 0.00333333 | 4002.218519 | 4002.218522 |
| 4002.218586 | Kernel\_Read\|xilinx:adm-pcie-7v3:1ddr:3.0-0\|cnn\_GOOD | Read | OCL Region | 1 | 1 | 127973 | 127974 | 0.00333333 | 4002.218586 | 4002.218589 |
| 4002.218652 | Kernel\_Read\|xilinx:adm-pcie-7v3:1ddr:3.0-0\|cnn\_GOOD | Read | OCL Region | 1 | 1 | 127993 | 127994 | 0.00333333 | 4002.218652 | 4002.218656 |
| 4002.218736 | Kernel\_Read\|xilinx:adm-pcie-7v3:1ddr:3.0-0\|cnn\_GOOD | Read | OCL Region | 1 | 1 | 128018 | 128019 | 0.00333333 | 4002.218736 | 4002.218739 |
| 4002.218792 | Kernel\_Read\|xilinx:adm-pcie-7v3:1ddr:3.0-0\|cnn\_GOOD | Read | OCL Region | 1 | 1 | 128035 | 128036 | 0.00333333 | 4002.218792 | 4002.218796 |
| 4002.218852 | Kernel\_Read\|xilinx:adm-pcie-7v3:1ddr:3.0-0\|cnn\_GOOD | Read | OCL Region | 1 | 1 | 128053 | 128054 | 0.00333333 | 4002.218852 | 4002.218856 |
| 4002.218919 | Kernel\_Read\|xilinx:adm-pcie-7v3:1ddr:3.0-0\|cnn\_GOOD | Read | OCL Region | 1 | 1 | 128073 | 128074 | 0.00333333 | 4002.218919 | 4002.218922 |
| 4002.218986 | Kernel\_Read\|xilinx:adm-pcie-7v3:1ddr:3.0-0\|cnn\_GOOD | Read | OCL Region | 1 | 1 | 128093 | 128094 | 0.00333333 | 4002.218986 | 4002.218989 |
| 4002.219262 | Kernel\_Read\|xilinx:adm-pcie-7v3:1ddr:3.0-0\|cnn\_GOOD | Read | OCL Region | 1 | 1 | 128176 | 128177 | 0.00333333 | 4002.219262 | 4002.219266 |
| 4002.219266 | Kernel\_Read\|xilinx:adm-pcie-7v3:1ddr:3.0-0\|cnn\_GOOD | Read | OCL Region | 1 | 1 | 128177 | 128178 | 0.00333333 | 4002.219266 | 4002.219269 |
| 4002.219269 | Kernel\_Read\|xilinx:adm-pcie-7v3:1ddr:3.0-0\|cnn\_GOOD | Read | OCL Region | 1 | 1 | 128178 | 128179 | 0.00333333 | 4002.219269 | 4002.219272 |
| 4002.219276 | Kernel\_Read\|xilinx:adm-pcie-7v3:1ddr:3.0-0\|cnn\_GOOD | Read | OCL Region | 1 | 1 | 128180 | 128181 | 0.00333333 | 4002.219276 | 4002.219279 |
| 4002.219336 | Kernel\_Read\|xilinx:adm-pcie-7v3:1ddr:3.0-0\|cnn\_GOOD | Read | OCL Region | 1 | 1 | 128198 | 128199 | 0.00333333 | 4002.219336 | 4002.219339 |
| 4002.219392 | Kernel\_Read\|xilinx:adm-pcie-7v3:1ddr:3.0-0\|cnn\_GOOD | Read | OCL Region | 1 | 1 | 128215 | 128216 | 0.00333333 | 4002.219392 | 4002.219396 |
| 4002.219452 | Kernel\_Read\|xilinx:adm-pcie-7v3:1ddr:3.0-0\|cnn\_GOOD | Read | OCL Region | 1 | 1 | 128233 | 128234 | 0.00333333 | 4002.219452 | 4002.219456 |
| 4002.219519 | Kernel\_Read\|xilinx:adm-pcie-7v3:1ddr:3.0-0\|cnn\_GOOD | Read | OCL Region | 1 | 1 | 128253 | 128254 | 0.00333333 | 4002.219519 | 4002.219522 |
| 4002.219586 | Kernel\_Read\|xilinx:adm-pcie-7v3:1ddr:3.0-0\|cnn\_GOOD | Read | OCL Region | 1 | 1 | 128273 | 128274 | 0.00333333 | 4002.219586 | 4002.219589 |
| 4002.219652 | Kernel\_Read\|xilinx:adm-pcie-7v3:1ddr:3.0-0\|cnn\_GOOD | Read | OCL Region | 1 | 1 | 128293 | 128294 | 0.00333333 | 4002.219652 | 4002.219656 |
| 4002.219719 | Kernel\_Read\|xilinx:adm-pcie-7v3:1ddr:3.0-0\|cnn\_GOOD | Read | OCL Region | 1 | 1 | 128313 | 128314 | 0.00333333 | 4002.219719 | 4002.219722 |
| 4002.219786 | Kernel\_Read\|xilinx:adm-pcie-7v3:1ddr:3.0-0\|cnn\_GOOD | Read | OCL Region | 1 | 1 | 128333 | 128334 | 0.00333333 | 4002.219786 | 4002.219789 |
| 4002.219852 | Kernel\_Read\|xilinx:adm-pcie-7v3:1ddr:3.0-0\|cnn\_GOOD | Read | OCL Region | 1 | 1 | 128353 | 128354 | 0.00333333 | 4002.219852 | 4002.219856 |
| 4002.219919 | Kernel\_Read\|xilinx:adm-pcie-7v3:1ddr:3.0-0\|cnn\_GOOD | Read | OCL Region | 1 | 1 | 128373 | 128374 | 0.00333333 | 4002.219919 | 4002.219922 |
| 4002.219986 | Kernel\_Read\|xilinx:adm-pcie-7v3:1ddr:3.0-0\|cnn\_GOOD | Read | OCL Region | 1 | 1 | 128393 | 128394 | 0.00333333 | 4002.219986 | 4002.219989 |
| 4002.220052 | Kernel\_Read\|xilinx:adm-pcie-7v3:1ddr:3.0-0\|cnn\_GOOD | Read | OCL Region | 1 | 1 | 128413 | 128414 | 0.00333333 | 4002.220052 | 4002.220056 |
| 4002.220119 | Kernel\_Read\|xilinx:adm-pcie-7v3:1ddr:3.0-0\|cnn\_GOOD | Read | OCL Region | 1 | 1 | 128433 | 128434 | 0.00333333 | 4002.220119 | 4002.220122 |
| 4002.220186 | Kernel\_Read\|xilinx:adm-pcie-7v3:1ddr:3.0-0\|cnn\_GOOD | Read | OCL Region | 1 | 1 | 128453 | 128454 | 0.00333333 | 4002.220186 | 4002.220189 |
| 4002.220252 | Kernel\_Read\|xilinx:adm-pcie-7v3:1ddr:3.0-0\|cnn\_GOOD | Read | OCL Region | 1 | 1 | 128473 | 128474 | 0.00333333 | 4002.220252 | 4002.220256 |
| 4002.220319 | Kernel\_Read\|xilinx:adm-pcie-7v3:1ddr:3.0-0\|cnn\_GOOD | Read | OCL Region | 1 | 1 | 128493 | 128494 | 0.00333333 | 4002.220319 | 4002.220322 |
| 4002.220386 | Kernel\_Read\|xilinx:adm-pcie-7v3:1ddr:3.0-0\|cnn\_GOOD | Read | OCL Region | 1 | 1 | 128513 | 128514 | 0.00333333 | 4002.220386 | 4002.220389 |
| 4002.220452 | Kernel\_Read\|xilinx:adm-pcie-7v3:1ddr:3.0-0\|cnn\_GOOD | Read | OCL Region | 1 | 1 | 128533 | 128534 | 0.00333333 | 4002.220452 | 4002.220456 |
| 4002.220519 | Kernel\_Read\|xilinx:adm-pcie-7v3:1ddr:3.0-0\|cnn\_GOOD | Read | OCL Region | 1 | 1 | 128553 | 128554 | 0.00333333 | 4002.220519 | 4002.220522 |
| 4002.220586 | Kernel\_Read\|xilinx:adm-pcie-7v3:1ddr:3.0-0\|cnn\_GOOD | Read | OCL Region | 1 | 1 | 128573 | 128574 | 0.00333333 | 4002.220586 | 4002.220589 |
| 4002.220652 | Kernel\_Read\|xilinx:adm-pcie-7v3:1ddr:3.0-0\|cnn\_GOOD | Read | OCL Region | 1 | 1 | 128593 | 128594 | 0.00333333 | 4002.220652 | 4002.220656 |
| 4002.220719 | Kernel\_Read\|xilinx:adm-pcie-7v3:1ddr:3.0-0\|cnn\_GOOD | Read | OCL Region | 1 | 1 | 128613 | 128614 | 0.00333333 | 4002.220719 | 4002.220722 |
| 4002.220786 | Kernel\_Read\|xilinx:adm-pcie-7v3:1ddr:3.0-0\|cnn\_GOOD | Read | OCL Region | 1 | 1 | 128633 | 128634 | 0.00333333 | 4002.220786 | 4002.220789 |
| 4002.220849 | Kernel\_Read\|xilinx:adm-pcie-7v3:1ddr:3.0-0\|cnn\_GOOD | Read | OCL Region | 1 | 1 | 128652 | 128653 | 0.00333333 | 4002.220849 | 4002.220852 |
| 4002.220919 | Kernel\_Read\|xilinx:adm-pcie-7v3:1ddr:3.0-0\|cnn\_GOOD | Read | OCL Region | 1 | 1 | 128673 | 128674 | 0.00333333 | 4002.220919 | 4002.220922 |
| 4002.220986 | Kernel\_Read\|xilinx:adm-pcie-7v3:1ddr:3.0-0\|cnn\_GOOD | Read | OCL Region | 1 | 1 | 128693 | 128694 | 0.00333333 | 4002.220986 | 4002.220989 |
| 4002.221052 | Kernel\_Read\|xilinx:adm-pcie-7v3:1ddr:3.0-0\|cnn\_GOOD | Read | OCL Region | 1 | 1 | 128713 | 128714 | 0.00333333 | 4002.221052 | 4002.221056 |
| 4002.221119 | Kernel\_Read\|xilinx:adm-pcie-7v3:1ddr:3.0-0\|cnn\_GOOD | Read | OCL Region | 1 | 1 | 128733 | 128734 | 0.00333333 | 4002.221119 | 4002.221122 |
| 4002.221186 | Kernel\_Read\|xilinx:adm-pcie-7v3:1ddr:3.0-0\|cnn\_GOOD | Read | OCL Region | 1 | 1 | 128753 | 128754 | 0.00333333 | 4002.221186 | 4002.221189 |
| 4002.221252 | Kernel\_Read\|xilinx:adm-pcie-7v3:1ddr:3.0-0\|cnn\_GOOD | Read | OCL Region | 1 | 1 | 128773 | 128774 | 0.00333333 | 4002.221252 | 4002.221256 |
| 4002.221319 | Kernel\_Read\|xilinx:adm-pcie-7v3:1ddr:3.0-0\|cnn\_GOOD | Read | OCL Region | 1 | 1 | 128793 | 128794 | 0.00333333 | 4002.221319 | 4002.221322 |
| 4002.221386 | Kernel\_Read\|xilinx:adm-pcie-7v3:1ddr:3.0-0\|cnn\_GOOD | Read | OCL Region | 1 | 1 | 128813 | 128814 | 0.00333333 | 4002.221386 | 4002.221389 |
| 4002.221452 | Kernel\_Read\|xilinx:adm-pcie-7v3:1ddr:3.0-0\|cnn\_GOOD | Read | OCL Region | 1 | 1 | 128833 | 128834 | 0.00333333 | 4002.221452 | 4002.221456 |
| 4002.221519 | Kernel\_Read\|xilinx:adm-pcie-7v3:1ddr:3.0-0\|cnn\_GOOD | Read | OCL Region | 1 | 1 | 128853 | 128854 | 0.00333333 | 4002.221519 | 4002.221522 |
| 4002.221586 | Kernel\_Read\|xilinx:adm-pcie-7v3:1ddr:3.0-0\|cnn\_GOOD | Read | OCL Region | 1 | 1 | 128873 | 128874 | 0.00333333 | 4002.221586 | 4002.221589 |
| 4002.221652 | Kernel\_Read\|xilinx:adm-pcie-7v3:1ddr:3.0-0\|cnn\_GOOD | Read | OCL Region | 1 | 1 | 128893 | 128894 | 0.00333333 | 4002.221652 | 4002.221656 |
| 4002.221719 | Kernel\_Read\|xilinx:adm-pcie-7v3:1ddr:3.0-0\|cnn\_GOOD | Read | OCL Region | 1 | 1 | 128913 | 128914 | 0.00333333 | 4002.221719 | 4002.221722 |
| 4002.221786 | Kernel\_Read\|xilinx:adm-pcie-7v3:1ddr:3.0-0\|cnn\_GOOD | Read | OCL Region | 1 | 1 | 128933 | 128934 | 0.00333333 | 4002.221786 | 4002.221789 |
| 4002.221852 | Kernel\_Read\|xilinx:adm-pcie-7v3:1ddr:3.0-0\|cnn\_GOOD | Read | OCL Region | 1 | 1 | 128953 | 128954 | 0.00333333 | 4002.221852 | 4002.221856 |
| 4002.221919 | Kernel\_Read\|xilinx:adm-pcie-7v3:1ddr:3.0-0\|cnn\_GOOD | Read | OCL Region | 1 | 1 | 128973 | 128974 | 0.00333333 | 4002.221919 | 4002.221922 |
| 4002.221986 | Kernel\_Read\|xilinx:adm-pcie-7v3:1ddr:3.0-0\|cnn\_GOOD | Read | OCL Region | 1 | 1 | 128993 | 128994 | 0.00333333 | 4002.221986 | 4002.221989 |
| 4002.222052 | Kernel\_Read\|xilinx:adm-pcie-7v3:1ddr:3.0-0\|cnn\_GOOD | Read | OCL Region | 1 | 1 | 129013 | 129014 | 0.00333333 | 4002.222052 | 4002.222056 |
| 4002.222119 | Kernel\_Read\|xilinx:adm-pcie-7v3:1ddr:3.0-0\|cnn\_GOOD | Read | OCL Region | 1 | 1 | 129033 | 129034 | 0.00333333 | 4002.222119 | 4002.222122 |
| 4002.222186 | Kernel\_Read\|xilinx:adm-pcie-7v3:1ddr:3.0-0\|cnn\_GOOD | Read | OCL Region | 1 | 1 | 129053 | 129054 | 0.00333333 | 4002.222186 | 4002.222189 |
| 4002.222252 | Kernel\_Read\|xilinx:adm-pcie-7v3:1ddr:3.0-0\|cnn\_GOOD | Read | OCL Region | 1 | 1 | 129073 | 129074 | 0.00333333 | 4002.222252 | 4002.222256 |
| 4002.222319 | Kernel\_Read\|xilinx:adm-pcie-7v3:1ddr:3.0-0\|cnn\_GOOD | Read | OCL Region | 1 | 1 | 129093 | 129094 | 0.00333333 | 4002.222319 | 4002.222322 |
| 4002.222386 | Kernel\_Read\|xilinx:adm-pcie-7v3:1ddr:3.0-0\|cnn\_GOOD | Read | OCL Region | 1 | 1 | 129113 | 129114 | 0.00333333 | 4002.222386 | 4002.222389 |
| 4002.222452 | Kernel\_Read\|xilinx:adm-pcie-7v3:1ddr:3.0-0\|cnn\_GOOD | Read | OCL Region | 1 | 1 | 129133 | 129134 | 0.00333333 | 4002.222452 | 4002.222456 |
| 4002.222529 | Kernel\_Read\|xilinx:adm-pcie-7v3:1ddr:3.0-0\|cnn\_GOOD | Read | OCL Region | 1 | 1 | 129156 | 129157 | 0.00333333 | 4002.222529 | 4002.222532 |
| 4002.222586 | Kernel\_Read\|xilinx:adm-pcie-7v3:1ddr:3.0-0\|cnn\_GOOD | Read | OCL Region | 1 | 1 | 129173 | 129174 | 0.00333333 | 4002.222586 | 4002.222589 |
| 4002.222652 | Kernel\_Read\|xilinx:adm-pcie-7v3:1ddr:3.0-0\|cnn\_GOOD | Read | OCL Region | 1 | 1 | 129193 | 129194 | 0.00333333 | 4002.222652 | 4002.222656 |
| 4002.222719 | Kernel\_Read\|xilinx:adm-pcie-7v3:1ddr:3.0-0\|cnn\_GOOD | Read | OCL Region | 1 | 1 | 129213 | 129214 | 0.00333333 | 4002.222719 | 4002.222722 |
| 4002.222786 | Kernel\_Read\|xilinx:adm-pcie-7v3:1ddr:3.0-0\|cnn\_GOOD | Read | OCL Region | 1 | 1 | 129233 | 129234 | 0.00333333 | 4002.222786 | 4002.222789 |
| 4002.222852 | Kernel\_Read\|xilinx:adm-pcie-7v3:1ddr:3.0-0\|cnn\_GOOD | Read | OCL Region | 1 | 1 | 129253 | 129254 | 0.00333333 | 4002.222852 | 4002.222856 |
| 4002.222919 | Kernel\_Read\|xilinx:adm-pcie-7v3:1ddr:3.0-0\|cnn\_GOOD | Read | OCL Region | 1 | 1 | 129273 | 129274 | 0.00333333 | 4002.222919 | 4002.222922 |
| 4002.222969 | Kernel\_Read\|xilinx:adm-pcie-7v3:1ddr:3.0-0\|cnn\_GOOD | Read | OCL Region | 1 | 1 | 129288 | 129289 | 0.00333333 | 4002.222969 | 4002.222972 |
| 4002.222976 | Kernel\_Read\|xilinx:adm-pcie-7v3:1ddr:3.0-0\|cnn\_GOOD | Read | OCL Region | 1 | 1 | 129290 | 129291 | 0.00333333 | 4002.222976 | 4002.222979 |
| 4002.222979 | Kernel\_Read\|xilinx:adm-pcie-7v3:1ddr:3.0-0\|cnn\_GOOD | Read | OCL Region | 1 | 1 | 129291 | 129292 | 0.00333333 | 4002.222979 | 4002.222982 |
| 4002.223029 | Kernel\_Read\|xilinx:adm-pcie-7v3:1ddr:3.0-0\|cnn\_GOOD | Read | OCL Region | 1 | 1 | 129306 | 129307 | 0.00333333 | 4002.223029 | 4002.223032 |
| 4002.223079 | Kernel\_Read\|xilinx:adm-pcie-7v3:1ddr:3.0-0\|cnn\_GOOD | Read | OCL Region | 1 | 1 | 129321 | 129322 | 0.00333333 | 4002.223079 | 4002.223082 |
| 4002.223086 | Kernel\_Read\|xilinx:adm-pcie-7v3:1ddr:3.0-0\|cnn\_GOOD | Read | OCL Region | 1 | 1 | 129323 | 129324 | 0.00333333 | 4002.223086 | 4002.223089 |
| 4002.223136 | Kernel\_Read\|xilinx:adm-pcie-7v3:1ddr:3.0-0\|cnn\_GOOD | Read | OCL Region | 1 | 1 | 129338 | 129339 | 0.00333333 | 4002.223136 | 4002.223139 |
| 4002.223192 | Kernel\_Read\|xilinx:adm-pcie-7v3:1ddr:3.0-0\|cnn\_GOOD | Read | OCL Region | 1 | 1 | 129355 | 129356 | 0.00333333 | 4002.223192 | 4002.223196 |
| 4002.223246 | Kernel\_Read\|xilinx:adm-pcie-7v3:1ddr:3.0-0\|cnn\_GOOD | Read | OCL Region | 1 | 1 | 129371 | 129372 | 0.00333333 | 4002.223246 | 4002.223249 |
| 4002.223302 | Kernel\_Read\|xilinx:adm-pcie-7v3:1ddr:3.0-0\|cnn\_GOOD | Read | OCL Region | 1 | 1 | 129388 | 129389 | 0.00333333 | 4002.223302 | 4002.223306 |
| 4002.223369 | Kernel\_Read\|xilinx:adm-pcie-7v3:1ddr:3.0-0\|cnn\_GOOD | Read | OCL Region | 1 | 1 | 129408 | 129409 | 0.00333333 | 4002.223369 | 4002.223372 |
| 4002.223436 | Kernel\_Read\|xilinx:adm-pcie-7v3:1ddr:3.0-0\|cnn\_GOOD | Read | OCL Region | 1 | 1 | 129428 | 129429 | 0.00333333 | 4002.223436 | 4002.223439 |
| 4002.223502 | Kernel\_Read\|xilinx:adm-pcie-7v3:1ddr:3.0-0\|cnn\_GOOD | Read | OCL Region | 1 | 1 | 129448 | 129449 | 0.00333333 | 4002.223502 | 4002.223506 |
| 4002.223569 | Kernel\_Read\|xilinx:adm-pcie-7v3:1ddr:3.0-0\|cnn\_GOOD | Read | OCL Region | 1 | 1 | 129468 | 129469 | 0.00333333 | 4002.223569 | 4002.223572 |
| 4002.223636 | Kernel\_Read\|xilinx:adm-pcie-7v3:1ddr:3.0-0\|cnn\_GOOD | Read | OCL Region | 1 | 1 | 129488 | 129489 | 0.00333333 | 4002.223636 | 4002.223639 |
| 4002.223702 | Kernel\_Read\|xilinx:adm-pcie-7v3:1ddr:3.0-0\|cnn\_GOOD | Read | OCL Region | 1 | 1 | 129508 | 129509 | 0.00333333 | 4002.223702 | 4002.223706 |
| 4002.223759 | Kernel\_Read\|xilinx:adm-pcie-7v3:1ddr:3.0-0\|cnn\_GOOD | Read | OCL Region | 1 | 1 | 129525 | 129526 | 0.00333333 | 4002.223759 | 4002.223762 |
| 4002.223802 | Kernel\_Read\|xilinx:adm-pcie-7v3:1ddr:3.0-0\|cnn\_GOOD | Read | OCL Region | 1 | 1 | 129538 | 129539 | 0.00333333 | 4002.223802 | 4002.223806 |
| 4002.223852 | Kernel\_Read\|xilinx:adm-pcie-7v3:1ddr:3.0-0\|cnn\_GOOD | Read | OCL Region | 1 | 1 | 129553 | 129554 | 0.00333333 | 4002.223852 | 4002.223856 |
| 4002.223902 | Kernel\_Read\|xilinx:adm-pcie-7v3:1ddr:3.0-0\|cnn\_GOOD | Read | OCL Region | 1 | 1 | 129568 | 129569 | 0.00333333 | 4002.223902 | 4002.223906 |
| 4002.223966 | Kernel\_Read\|xilinx:adm-pcie-7v3:1ddr:3.0-0\|cnn\_GOOD | Read | OCL Region | 1 | 1 | 129587 | 129588 | 0.00333333 | 4002.223966 | 4002.223969 |
| 4002.224016 | Kernel\_Read\|xilinx:adm-pcie-7v3:1ddr:3.0-0\|cnn\_GOOD | Read | OCL Region | 1 | 1 | 129602 | 129603 | 0.00333333 | 4002.224016 | 4002.224019 |
| 4002.224066 | Kernel\_Read\|xilinx:adm-pcie-7v3:1ddr:3.0-0\|cnn\_GOOD | Read | OCL Region | 1 | 1 | 129617 | 129618 | 0.00333333 | 4002.224066 | 4002.224069 |
| 4002.224116 | Kernel\_Read\|xilinx:adm-pcie-7v3:1ddr:3.0-0\|cnn\_GOOD | Read | OCL Region | 1 | 1 | 129632 | 129633 | 0.00333333 | 4002.224116 | 4002.224119 |
| 4002.224166 | Kernel\_Read\|xilinx:adm-pcie-7v3:1ddr:3.0-0\|cnn\_GOOD | Read | OCL Region | 1 | 1 | 129647 | 129648 | 0.00333333 | 4002.224166 | 4002.224169 |
| 4002.224216 | Kernel\_Read\|xilinx:adm-pcie-7v3:1ddr:3.0-0\|cnn\_GOOD | Read | OCL Region | 1 | 1 | 129662 | 129663 | 0.00333333 | 4002.224216 | 4002.224219 |
| 4002.224266 | Kernel\_Read\|xilinx:adm-pcie-7v3:1ddr:3.0-0\|cnn\_GOOD | Read | OCL Region | 1 | 1 | 129677 | 129678 | 0.00333333 | 4002.224266 | 4002.224269 |
| 4002.224316 | Kernel\_Read\|xilinx:adm-pcie-7v3:1ddr:3.0-0\|cnn\_GOOD | Read | OCL Region | 1 | 1 | 129692 | 129693 | 0.00333333 | 4002.224316 | 4002.224319 |
| 4002.224366 | Kernel\_Read\|xilinx:adm-pcie-7v3:1ddr:3.0-0\|cnn\_GOOD | Read | OCL Region | 1 | 1 | 129707 | 129708 | 0.00333333 | 4002.224366 | 4002.224369 |
| 4002.224416 | Kernel\_Read\|xilinx:adm-pcie-7v3:1ddr:3.0-0\|cnn\_GOOD | Read | OCL Region | 1 | 1 | 129722 | 129723 | 0.00333333 | 4002.224416 | 4002.224419 |
| 4002.224466 | Kernel\_Read\|xilinx:adm-pcie-7v3:1ddr:3.0-0\|cnn\_GOOD | Read | OCL Region | 1 | 1 | 129737 | 129738 | 0.00333333 | 4002.224466 | 4002.224469 |
| 4002.224516 | Kernel\_Read\|xilinx:adm-pcie-7v3:1ddr:3.0-0\|cnn\_GOOD | Read | OCL Region | 1 | 1 | 129752 | 129753 | 0.00333333 | 4002.224516 | 4002.224519 |
| 4002.224566 | Kernel\_Read\|xilinx:adm-pcie-7v3:1ddr:3.0-0\|cnn\_GOOD | Read | OCL Region | 1 | 1 | 129767 | 129768 | 0.00333333 | 4002.224566 | 4002.224569 |
| 4002.224616 | Kernel\_Read\|xilinx:adm-pcie-7v3:1ddr:3.0-0\|cnn\_GOOD | Read | OCL Region | 1 | 1 | 129782 | 129783 | 0.00333333 | 4002.224616 | 4002.224619 |
| 4002.224666 | Kernel\_Read\|xilinx:adm-pcie-7v3:1ddr:3.0-0\|cnn\_GOOD | Read | OCL Region | 1 | 1 | 129797 | 129798 | 0.00333333 | 4002.224666 | 4002.224669 |
| 4002.224716 | Kernel\_Read\|xilinx:adm-pcie-7v3:1ddr:3.0-0\|cnn\_GOOD | Read | OCL Region | 1 | 1 | 129812 | 129813 | 0.00333333 | 4002.224716 | 4002.224719 |
| 4002.224766 | Kernel\_Read\|xilinx:adm-pcie-7v3:1ddr:3.0-0\|cnn\_GOOD | Read | OCL Region | 1 | 1 | 129827 | 129828 | 0.00333333 | 4002.224766 | 4002.224769 |
| 4002.224826 | Kernel\_Read\|xilinx:adm-pcie-7v3:1ddr:3.0-0\|cnn\_GOOD | Read | OCL Region | 1 | 1 | 129845 | 129846 | 0.00333333 | 4002.224826 | 4002.224829 |
| 4002.224876 | Kernel\_Read\|xilinx:adm-pcie-7v3:1ddr:3.0-0\|cnn\_GOOD | Read | OCL Region | 1 | 1 | 129860 | 129861 | 0.00333333 | 4002.224876 | 4002.224879 |
| 4002.224926 | Kernel\_Read\|xilinx:adm-pcie-7v3:1ddr:3.0-0\|cnn\_GOOD | Read | OCL Region | 1 | 1 | 129875 | 129876 | 0.00333333 | 4002.224926 | 4002.224929 |
| 4002.224976 | Kernel\_Read\|xilinx:adm-pcie-7v3:1ddr:3.0-0\|cnn\_GOOD | Read | OCL Region | 1 | 1 | 129890 | 129891 | 0.00333333 | 4002.224976 | 4002.224979 |
| 4002.225026 | Kernel\_Read\|xilinx:adm-pcie-7v3:1ddr:3.0-0\|cnn\_GOOD | Read | OCL Region | 1 | 1 | 129905 | 129906 | 0.00333333 | 4002.225026 | 4002.225029 |
| 4002.225076 | Kernel\_Read\|xilinx:adm-pcie-7v3:1ddr:3.0-0\|cnn\_GOOD | Read | OCL Region | 1 | 1 | 129920 | 129921 | 0.00333333 | 4002.225076 | 4002.225079 |
| 4002.225126 | Kernel\_Read\|xilinx:adm-pcie-7v3:1ddr:3.0-0\|cnn\_GOOD | Read | OCL Region | 1 | 1 | 129935 | 129936 | 0.00333333 | 4002.225126 | 4002.225129 |
| 4002.225176 | Kernel\_Read\|xilinx:adm-pcie-7v3:1ddr:3.0-0\|cnn\_GOOD | Read | OCL Region | 1 | 1 | 129950 | 129951 | 0.00333333 | 4002.225176 | 4002.225179 |
| 4002.225226 | Kernel\_Read\|xilinx:adm-pcie-7v3:1ddr:3.0-0\|cnn\_GOOD | Read | OCL Region | 1 | 1 | 129965 | 129966 | 0.00333333 | 4002.225226 | 4002.225229 |
| 4002.225276 | Kernel\_Read\|xilinx:adm-pcie-7v3:1ddr:3.0-0\|cnn\_GOOD | Read | OCL Region | 1 | 1 | 129980 | 129981 | 0.00333333 | 4002.225276 | 4002.225279 |
| 4002.225326 | Kernel\_Read\|xilinx:adm-pcie-7v3:1ddr:3.0-0\|cnn\_GOOD | Read | OCL Region | 1 | 1 | 129995 | 129996 | 0.00333333 | 4002.225326 | 4002.225329 |
| 4002.225376 | Kernel\_Read\|xilinx:adm-pcie-7v3:1ddr:3.0-0\|cnn\_GOOD | Read | OCL Region | 1 | 1 | 130010 | 130011 | 0.00333333 | 4002.225376 | 4002.225379 |
| 4002.225426 | Kernel\_Read\|xilinx:adm-pcie-7v3:1ddr:3.0-0\|cnn\_GOOD | Read | OCL Region | 1 | 1 | 130025 | 130026 | 0.00333333 | 4002.225426 | 4002.225429 |
| 4002.225696 | Kernel\_Read\|xilinx:adm-pcie-7v3:1ddr:3.0-0\|cnn\_GOOD | Read | OCL Region | 1 | 1 | 130106 | 130107 | 0.00333333 | 4002.225696 | 4002.225699 |
| 4002.225746 | Kernel\_Read\|xilinx:adm-pcie-7v3:1ddr:3.0-0\|cnn\_GOOD | Read | OCL Region | 1 | 1 | 130121 | 130122 | 0.00333333 | 4002.225746 | 4002.225749 |
| 4002.225796 | Kernel\_Read\|xilinx:adm-pcie-7v3:1ddr:3.0-0\|cnn\_GOOD | Read | OCL Region | 1 | 1 | 130136 | 130137 | 0.00333333 | 4002.225796 | 4002.225799 |
| 4002.225846 | Kernel\_Read\|xilinx:adm-pcie-7v3:1ddr:3.0-0\|cnn\_GOOD | Read | OCL Region | 1 | 1 | 130151 | 130152 | 0.00333333 | 4002.225846 | 4002.225849 |
| 4002.225912 | Kernel\_Read\|xilinx:adm-pcie-7v3:1ddr:3.0-0\|cnn\_GOOD | Read | OCL Region | 1 | 1 | 130171 | 130172 | 0.00333333 | 4002.225912 | 4002.225916 |
| 4002.225962 | Kernel\_Read\|xilinx:adm-pcie-7v3:1ddr:3.0-0\|cnn\_GOOD | Read | OCL Region | 1 | 1 | 130186 | 130187 | 0.00333333 | 4002.225962 | 4002.225966 |
| 4002.226012 | Kernel\_Read\|xilinx:adm-pcie-7v3:1ddr:3.0-0\|cnn\_GOOD | Read | OCL Region | 1 | 1 | 130201 | 130202 | 0.00333333 | 4002.226012 | 4002.226016 |
| 4002.226062 | Kernel\_Read\|xilinx:adm-pcie-7v3:1ddr:3.0-0\|cnn\_GOOD | Read | OCL Region | 1 | 1 | 130216 | 130217 | 0.00333333 | 4002.226062 | 4002.226066 |
| 4002.226112 | Kernel\_Read\|xilinx:adm-pcie-7v3:1ddr:3.0-0\|cnn\_GOOD | Read | OCL Region | 1 | 1 | 130231 | 130232 | 0.00333333 | 4002.226112 | 4002.226116 |
| 4002.226162 | Kernel\_Read\|xilinx:adm-pcie-7v3:1ddr:3.0-0\|cnn\_GOOD | Read | OCL Region | 1 | 1 | 130246 | 130247 | 0.00333333 | 4002.226162 | 4002.226166 |
| 4002.226212 | Kernel\_Read\|xilinx:adm-pcie-7v3:1ddr:3.0-0\|cnn\_GOOD | Read | OCL Region | 1 | 1 | 130261 | 130262 | 0.00333333 | 4002.226212 | 4002.226216 |
| 4002.226262 | Kernel\_Read\|xilinx:adm-pcie-7v3:1ddr:3.0-0\|cnn\_GOOD | Read | OCL Region | 1 | 1 | 130276 | 130277 | 0.00333333 | 4002.226262 | 4002.226266 |
| 4002.226312 | Kernel\_Read\|xilinx:adm-pcie-7v3:1ddr:3.0-0\|cnn\_GOOD | Read | OCL Region | 1 | 1 | 130291 | 130292 | 0.00333333 | 4002.226312 | 4002.226316 |
| 4002.226362 | Kernel\_Read\|xilinx:adm-pcie-7v3:1ddr:3.0-0\|cnn\_GOOD | Read | OCL Region | 1 | 1 | 130306 | 130307 | 0.00333333 | 4002.226362 | 4002.226366 |
| 4002.226412 | Kernel\_Read\|xilinx:adm-pcie-7v3:1ddr:3.0-0\|cnn\_GOOD | Read | OCL Region | 1 | 1 | 130321 | 130322 | 0.00333333 | 4002.226412 | 4002.226416 |
| 4002.226462 | Kernel\_Read\|xilinx:adm-pcie-7v3:1ddr:3.0-0\|cnn\_GOOD | Read | OCL Region | 1 | 1 | 130336 | 130337 | 0.00333333 | 4002.226462 | 4002.226466 |
| 4002.226512 | Kernel\_Read\|xilinx:adm-pcie-7v3:1ddr:3.0-0\|cnn\_GOOD | Read | OCL Region | 1 | 1 | 130351 | 130352 | 0.00333333 | 4002.226512 | 4002.226516 |
| 4002.226562 | Kernel\_Read\|xilinx:adm-pcie-7v3:1ddr:3.0-0\|cnn\_GOOD | Read | OCL Region | 1 | 1 | 130366 | 130367 | 0.00333333 | 4002.226562 | 4002.226566 |
| 4002.226612 | Kernel\_Read\|xilinx:adm-pcie-7v3:1ddr:3.0-0\|cnn\_GOOD | Read | OCL Region | 1 | 1 | 130381 | 130382 | 0.00333333 | 4002.226612 | 4002.226616 |
| 4002.226662 | Kernel\_Read\|xilinx:adm-pcie-7v3:1ddr:3.0-0\|cnn\_GOOD | Read | OCL Region | 1 | 1 | 130396 | 130397 | 0.00333333 | 4002.226662 | 4002.226666 |
| 4002.226712 | Kernel\_Read\|xilinx:adm-pcie-7v3:1ddr:3.0-0\|cnn\_GOOD | Read | OCL Region | 1 | 1 | 130411 | 130412 | 0.00333333 | 4002.226712 | 4002.226716 |
| 4002.226762 | Kernel\_Read\|xilinx:adm-pcie-7v3:1ddr:3.0-0\|cnn\_GOOD | Read | OCL Region | 1 | 1 | 130426 | 130427 | 0.00333333 | 4002.226762 | 4002.226766 |
| 4002.226812 | Kernel\_Read\|xilinx:adm-pcie-7v3:1ddr:3.0-0\|cnn\_GOOD | Read | OCL Region | 1 | 1 | 130441 | 130442 | 0.00333333 | 4002.226812 | 4002.226816 |
| 4002.226862 | Kernel\_Read\|xilinx:adm-pcie-7v3:1ddr:3.0-0\|cnn\_GOOD | Read | OCL Region | 1 | 1 | 130456 | 130457 | 0.00333333 | 4002.226862 | 4002.226866 |
| 4002.226912 | Kernel\_Read\|xilinx:adm-pcie-7v3:1ddr:3.0-0\|cnn\_GOOD | Read | OCL Region | 1 | 1 | 130471 | 130472 | 0.00333333 | 4002.226912 | 4002.226916 |
| 4002.226969 | Kernel\_Read\|xilinx:adm-pcie-7v3:1ddr:3.0-0\|cnn\_GOOD | Read | OCL Region | 1 | 1 | 130488 | 130489 | 0.00333333 | 4002.226969 | 4002.226972 |
| 4002.227019 | Kernel\_Read\|xilinx:adm-pcie-7v3:1ddr:3.0-0\|cnn\_GOOD | Read | OCL Region | 1 | 1 | 130503 | 130504 | 0.00333333 | 4002.227019 | 4002.227022 |
| 4002.227069 | Kernel\_Read\|xilinx:adm-pcie-7v3:1ddr:3.0-0\|cnn\_GOOD | Read | OCL Region | 1 | 1 | 130518 | 130519 | 0.00333333 | 4002.227069 | 4002.227072 |
| 4002.227119 | Kernel\_Read\|xilinx:adm-pcie-7v3:1ddr:3.0-0\|cnn\_GOOD | Read | OCL Region | 1 | 1 | 130533 | 130534 | 0.00333333 | 4002.227119 | 4002.227122 |
| 4002.227169 | Kernel\_Read\|xilinx:adm-pcie-7v3:1ddr:3.0-0\|cnn\_GOOD | Read | OCL Region | 1 | 1 | 130548 | 130549 | 0.00333333 | 4002.227169 | 4002.227172 |
| 4002.227219 | Kernel\_Read\|xilinx:adm-pcie-7v3:1ddr:3.0-0\|cnn\_GOOD | Read | OCL Region | 1 | 1 | 130563 | 130564 | 0.00333333 | 4002.227219 | 4002.227222 |
| 4002.227269 | Kernel\_Read\|xilinx:adm-pcie-7v3:1ddr:3.0-0\|cnn\_GOOD | Read | OCL Region | 1 | 1 | 130578 | 130579 | 0.00333333 | 4002.227269 | 4002.227272 |
| 4002.227319 | Kernel\_Read\|xilinx:adm-pcie-7v3:1ddr:3.0-0\|cnn\_GOOD | Read | OCL Region | 1 | 1 | 130593 | 130594 | 0.00333333 | 4002.227319 | 4002.227322 |
| 4002.227369 | Kernel\_Read\|xilinx:adm-pcie-7v3:1ddr:3.0-0\|cnn\_GOOD | Read | OCL Region | 1 | 1 | 130608 | 130609 | 0.00333333 | 4002.227369 | 4002.227372 |
| 4002.227419 | Kernel\_Read\|xilinx:adm-pcie-7v3:1ddr:3.0-0\|cnn\_GOOD | Read | OCL Region | 1 | 1 | 130623 | 130624 | 0.00333333 | 4002.227419 | 4002.227422 |
| 4002.227469 | Kernel\_Read\|xilinx:adm-pcie-7v3:1ddr:3.0-0\|cnn\_GOOD | Read | OCL Region | 1 | 1 | 130638 | 130639 | 0.00333333 | 4002.227469 | 4002.227472 |
| 4002.227519 | Kernel\_Read\|xilinx:adm-pcie-7v3:1ddr:3.0-0\|cnn\_GOOD | Read | OCL Region | 1 | 1 | 130653 | 130654 | 0.00333333 | 4002.227519 | 4002.227522 |
| 4002.227569 | Kernel\_Read\|xilinx:adm-pcie-7v3:1ddr:3.0-0\|cnn\_GOOD | Read | OCL Region | 1 | 1 | 130668 | 130669 | 0.00333333 | 4002.227569 | 4002.227572 |
| 4002.227619 | Kernel\_Read\|xilinx:adm-pcie-7v3:1ddr:3.0-0\|cnn\_GOOD | Read | OCL Region | 1 | 1 | 130683 | 130684 | 0.00333333 | 4002.227619 | 4002.227622 |
| 4002.227669 | Kernel\_Read\|xilinx:adm-pcie-7v3:1ddr:3.0-0\|cnn\_GOOD | Read | OCL Region | 1 | 1 | 130698 | 130699 | 0.00333333 | 4002.227669 | 4002.227672 |
| 4002.227719 | Kernel\_Read\|xilinx:adm-pcie-7v3:1ddr:3.0-0\|cnn\_GOOD | Read | OCL Region | 1 | 1 | 130713 | 130714 | 0.00333333 | 4002.227719 | 4002.227722 |
| 4002.227769 | Kernel\_Read\|xilinx:adm-pcie-7v3:1ddr:3.0-0\|cnn\_GOOD | Read | OCL Region | 1 | 1 | 130728 | 130729 | 0.00333333 | 4002.227769 | 4002.227772 |
| 4002.227819 | Kernel\_Read\|xilinx:adm-pcie-7v3:1ddr:3.0-0\|cnn\_GOOD | Read | OCL Region | 1 | 1 | 130743 | 130744 | 0.00333333 | 4002.227819 | 4002.227822 |
| 4002.227869 | Kernel\_Read\|xilinx:adm-pcie-7v3:1ddr:3.0-0\|cnn\_GOOD | Read | OCL Region | 1 | 1 | 130758 | 130759 | 0.00333333 | 4002.227869 | 4002.227872 |
| 4002.227929 | Kernel\_Read\|xilinx:adm-pcie-7v3:1ddr:3.0-0\|cnn\_GOOD | Read | OCL Region | 1 | 1 | 130776 | 130777 | 0.00333333 | 4002.227929 | 4002.227932 |
| 4002.227979 | Kernel\_Read\|xilinx:adm-pcie-7v3:1ddr:3.0-0\|cnn\_GOOD | Read | OCL Region | 1 | 1 | 130791 | 130792 | 0.00333333 | 4002.227979 | 4002.227982 |
| 4002.228029 | Kernel\_Read\|xilinx:adm-pcie-7v3:1ddr:3.0-0\|cnn\_GOOD | Read | OCL Region | 1 | 1 | 130806 | 130807 | 0.00333333 | 4002.228029 | 4002.228032 |
| 4002.228079 | Kernel\_Read\|xilinx:adm-pcie-7v3:1ddr:3.0-0\|cnn\_GOOD | Read | OCL Region | 1 | 1 | 130821 | 130822 | 0.00333333 | 4002.228079 | 4002.228082 |
| 4002.228129 | Kernel\_Read\|xilinx:adm-pcie-7v3:1ddr:3.0-0\|cnn\_GOOD | Read | OCL Region | 1 | 1 | 130836 | 130837 | 0.00333333 | 4002.228129 | 4002.228132 |
| 4002.228179 | Kernel\_Read\|xilinx:adm-pcie-7v3:1ddr:3.0-0\|cnn\_GOOD | Read | OCL Region | 1 | 1 | 130851 | 130852 | 0.00333333 | 4002.228179 | 4002.228182 |
| 4002.228229 | Kernel\_Read\|xilinx:adm-pcie-7v3:1ddr:3.0-0\|cnn\_GOOD | Read | OCL Region | 1 | 1 | 130866 | 130867 | 0.00333333 | 4002.228229 | 4002.228232 |
| 4002.228279 | Kernel\_Read\|xilinx:adm-pcie-7v3:1ddr:3.0-0\|cnn\_GOOD | Read | OCL Region | 1 | 1 | 130881 | 130882 | 0.00333333 | 4002.228279 | 4002.228282 |
| 4002.228329 | Kernel\_Read\|xilinx:adm-pcie-7v3:1ddr:3.0-0\|cnn\_GOOD | Read | OCL Region | 1 | 1 | 130896 | 130897 | 0.00333333 | 4002.228329 | 4002.228332 |
| 4002.228379 | Kernel\_Read\|xilinx:adm-pcie-7v3:1ddr:3.0-0\|cnn\_GOOD | Read | OCL Region | 1 | 1 | 130911 | 130912 | 0.00333333 | 4002.228379 | 4002.228382 |
| 4002.228432 | Kernel\_Read\|xilinx:adm-pcie-7v3:1ddr:3.0-0\|cnn\_GOOD | Read | OCL Region | 1 | 1 | 130927 | 130928 | 0.00333333 | 4002.228432 | 4002.228436 |
| 4002.228479 | Kernel\_Read\|xilinx:adm-pcie-7v3:1ddr:3.0-0\|cnn\_GOOD | Read | OCL Region | 1 | 1 | 130941 | 130942 | 0.00333333 | 4002.228479 | 4002.228482 |
| 4002.228529 | Kernel\_Read\|xilinx:adm-pcie-7v3:1ddr:3.0-0\|cnn\_GOOD | Read | OCL Region | 1 | 1 | 130956 | 130957 | 0.00333333 | 4002.228529 | 4002.228532 |
| 4002.228579 | Kernel\_Read\|xilinx:adm-pcie-7v3:1ddr:3.0-0\|cnn\_GOOD | Read | OCL Region | 1 | 1 | 130971 | 130972 | 0.00333333 | 4002.228579 | 4002.228582 |
| 4002.228629 | Kernel\_Read\|xilinx:adm-pcie-7v3:1ddr:3.0-0\|cnn\_GOOD | Read | OCL Region | 1 | 1 | 130986 | 130987 | 0.00333333 | 4002.228629 | 4002.228632 |
| 4002.228679 | Kernel\_Read\|xilinx:adm-pcie-7v3:1ddr:3.0-0\|cnn\_GOOD | Read | OCL Region | 1 | 1 | 131001 | 131002 | 0.00333333 | 4002.228679 | 4002.228682 |
| 4002.228729 | Kernel\_Read\|xilinx:adm-pcie-7v3:1ddr:3.0-0\|cnn\_GOOD | Read | OCL Region | 1 | 1 | 131016 | 131017 | 0.00333333 | 4002.228729 | 4002.228732 |
| 4002.228779 | Kernel\_Read\|xilinx:adm-pcie-7v3:1ddr:3.0-0\|cnn\_GOOD | Read | OCL Region | 1 | 1 | 131031 | 131032 | 0.00333333 | 4002.228779 | 4002.228782 |
| 4002.228832 | Kernel\_Read\|xilinx:adm-pcie-7v3:1ddr:3.0-0\|cnn\_GOOD | Read | OCL Region | 1 | 1 | 131047 | 131048 | 0.00333333 | 4002.228832 | 4002.228836 |
| 4002.228879 | Kernel\_Read\|xilinx:adm-pcie-7v3:1ddr:3.0-0\|cnn\_GOOD | Read | OCL Region | 1 | 1 | 131061 | 131062 | 0.00333333 | 4002.228879 | 4002.228882 |
| 4002.228942 | Kernel\_Read\|xilinx:adm-pcie-7v3:1ddr:3.0-0\|cnn\_GOOD | Read | OCL Region | 1 | 1 | 131080 | 131081 | 0.00333333 | 4002.228942 | 4002.228946 |
| 4002.228992 | Kernel\_Read\|xilinx:adm-pcie-7v3:1ddr:3.0-0\|cnn\_GOOD | Read | OCL Region | 1 | 1 | 131095 | 131096 | 0.00333333 | 4002.228992 | 4002.228996 |
| 4002.229042 | Kernel\_Read\|xilinx:adm-pcie-7v3:1ddr:3.0-0\|cnn\_GOOD | Read | OCL Region | 1 | 1 | 131110 | 131111 | 0.00333333 | 4002.229042 | 4002.229046 |
| 4002.229092 | Kernel\_Read\|xilinx:adm-pcie-7v3:1ddr:3.0-0\|cnn\_GOOD | Read | OCL Region | 1 | 1 | 131125 | 131126 | 0.00333333 | 4002.229092 | 4002.229096 |
| 4002.229142 | Kernel\_Read\|xilinx:adm-pcie-7v3:1ddr:3.0-0\|cnn\_GOOD | Read | OCL Region | 1 | 1 | 131140 | 131141 | 0.00333333 | 4002.229142 | 4002.229146 |
| 4002.229192 | Kernel\_Read\|xilinx:adm-pcie-7v3:1ddr:3.0-0\|cnn\_GOOD | Read | OCL Region | 1 | 1 | 131155 | 131156 | 0.00333333 | 4002.229192 | 4002.229196 |
| 4002.229242 | Kernel\_Read\|xilinx:adm-pcie-7v3:1ddr:3.0-0\|cnn\_GOOD | Read | OCL Region | 1 | 1 | 131170 | 131171 | 0.00333333 | 4002.229242 | 4002.229246 |
| 4002.229292 | Kernel\_Read\|xilinx:adm-pcie-7v3:1ddr:3.0-0\|cnn\_GOOD | Read | OCL Region | 1 | 1 | 131185 | 131186 | 0.00333333 | 4002.229292 | 4002.229296 |
| 4002.229342 | Kernel\_Read\|xilinx:adm-pcie-7v3:1ddr:3.0-0\|cnn\_GOOD | Read | OCL Region | 1 | 1 | 131200 | 131201 | 0.00333333 | 4002.229342 | 4002.229346 |
| 4002.229392 | Kernel\_Read\|xilinx:adm-pcie-7v3:1ddr:3.0-0\|cnn\_GOOD | Read | OCL Region | 1 | 1 | 131215 | 131216 | 0.00333333 | 4002.229392 | 4002.229396 |
| 4002.229442 | Kernel\_Read\|xilinx:adm-pcie-7v3:1ddr:3.0-0\|cnn\_GOOD | Read | OCL Region | 1 | 1 | 131230 | 131231 | 0.00333333 | 4002.229442 | 4002.229446 |
| 4002.229492 | Kernel\_Read\|xilinx:adm-pcie-7v3:1ddr:3.0-0\|cnn\_GOOD | Read | OCL Region | 1 | 1 | 131245 | 131246 | 0.00333333 | 4002.229492 | 4002.229496 |
| 4002.229542 | Kernel\_Read\|xilinx:adm-pcie-7v3:1ddr:3.0-0\|cnn\_GOOD | Read | OCL Region | 1 | 1 | 131260 | 131261 | 0.00333333 | 4002.229542 | 4002.229546 |
| 4002.229592 | Kernel\_Read\|xilinx:adm-pcie-7v3:1ddr:3.0-0\|cnn\_GOOD | Read | OCL Region | 1 | 1 | 131275 | 131276 | 0.00333333 | 4002.229592 | 4002.229596 |
| 4002.229642 | Kernel\_Read\|xilinx:adm-pcie-7v3:1ddr:3.0-0\|cnn\_GOOD | Read | OCL Region | 1 | 1 | 131290 | 131291 | 0.00333333 | 4002.229642 | 4002.229646 |
| 4002.229692 | Kernel\_Read\|xilinx:adm-pcie-7v3:1ddr:3.0-0\|cnn\_GOOD | Read | OCL Region | 1 | 1 | 131305 | 131306 | 0.00333333 | 4002.229692 | 4002.229696 |
| 4002.229742 | Kernel\_Read\|xilinx:adm-pcie-7v3:1ddr:3.0-0\|cnn\_GOOD | Read | OCL Region | 1 | 1 | 131320 | 131321 | 0.00333333 | 4002.229742 | 4002.229746 |
| 4002.229792 | Kernel\_Read\|xilinx:adm-pcie-7v3:1ddr:3.0-0\|cnn\_GOOD | Read | OCL Region | 1 | 1 | 131335 | 131336 | 0.00333333 | 4002.229792 | 4002.229796 |
| 4002.229842 | Kernel\_Read\|xilinx:adm-pcie-7v3:1ddr:3.0-0\|cnn\_GOOD | Read | OCL Region | 1 | 1 | 131350 | 131351 | 0.00333333 | 4002.229842 | 4002.229846 |
| 4002.229899 | Kernel\_Read\|xilinx:adm-pcie-7v3:1ddr:3.0-0\|cnn\_GOOD | Read | OCL Region | 1 | 1 | 131367 | 131368 | 0.00333333 | 4002.229899 | 4002.229902 |
| 4002.229949 | Kernel\_Read\|xilinx:adm-pcie-7v3:1ddr:3.0-0\|cnn\_GOOD | Read | OCL Region | 1 | 1 | 131382 | 131383 | 0.00333333 | 4002.229949 | 4002.229952 |
| 4002.229999 | Kernel\_Read\|xilinx:adm-pcie-7v3:1ddr:3.0-0\|cnn\_GOOD | Read | OCL Region | 1 | 1 | 131397 | 131398 | 0.00333333 | 4002.229999 | 4002.230002 |
| 4002.230049 | Kernel\_Read\|xilinx:adm-pcie-7v3:1ddr:3.0-0\|cnn\_GOOD | Read | OCL Region | 1 | 1 | 131412 | 131413 | 0.00333333 | 4002.230049 | 4002.230052 |
| 4002.230099 | Kernel\_Read\|xilinx:adm-pcie-7v3:1ddr:3.0-0\|cnn\_GOOD | Read | OCL Region | 1 | 1 | 131427 | 131428 | 0.00333333 | 4002.230099 | 4002.230102 |
| 4002.230149 | Kernel\_Read\|xilinx:adm-pcie-7v3:1ddr:3.0-0\|cnn\_GOOD | Read | OCL Region | 1 | 1 | 131442 | 131443 | 0.00333333 | 4002.230149 | 4002.230152 |
| 4002.230199 | Kernel\_Read\|xilinx:adm-pcie-7v3:1ddr:3.0-0\|cnn\_GOOD | Read | OCL Region | 1 | 1 | 131457 | 131458 | 0.00333333 | 4002.230199 | 4002.230202 |
| 4002.230249 | Kernel\_Read\|xilinx:adm-pcie-7v3:1ddr:3.0-0\|cnn\_GOOD | Read | OCL Region | 1 | 1 | 131472 | 131473 | 0.00333333 | 4002.230249 | 4002.230252 |
| 4002.230299 | Kernel\_Read\|xilinx:adm-pcie-7v3:1ddr:3.0-0\|cnn\_GOOD | Read | OCL Region | 1 | 1 | 131487 | 131488 | 0.00333333 | 4002.230299 | 4002.230302 |
| 4002.230349 | Kernel\_Read\|xilinx:adm-pcie-7v3:1ddr:3.0-0\|cnn\_GOOD | Read | OCL Region | 1 | 1 | 131502 | 131503 | 0.00333333 | 4002.230349 | 4002.230352 |
| 4002.230399 | Kernel\_Read\|xilinx:adm-pcie-7v3:1ddr:3.0-0\|cnn\_GOOD | Read | OCL Region | 1 | 1 | 131517 | 131518 | 0.00333333 | 4002.230399 | 4002.230402 |
| 4002.230449 | Kernel\_Read\|xilinx:adm-pcie-7v3:1ddr:3.0-0\|cnn\_GOOD | Read | OCL Region | 1 | 1 | 131532 | 131533 | 0.00333333 | 4002.230449 | 4002.230452 |
| 4002.230499 | Kernel\_Read\|xilinx:adm-pcie-7v3:1ddr:3.0-0\|cnn\_GOOD | Read | OCL Region | 1 | 1 | 131547 | 131548 | 0.00333333 | 4002.230499 | 4002.230502 |
| 4002.230549 | Kernel\_Read\|xilinx:adm-pcie-7v3:1ddr:3.0-0\|cnn\_GOOD | Read | OCL Region | 1 | 1 | 131562 | 131563 | 0.00333333 | 4002.230549 | 4002.230552 |
| 4002.230599 | Kernel\_Read\|xilinx:adm-pcie-7v3:1ddr:3.0-0\|cnn\_GOOD | Read | OCL Region | 1 | 1 | 131577 | 131578 | 0.00333333 | 4002.230599 | 4002.230602 |
| 4002.230649 | Kernel\_Read\|xilinx:adm-pcie-7v3:1ddr:3.0-0\|cnn\_GOOD | Read | OCL Region | 1 | 1 | 131592 | 131593 | 0.00333333 | 4002.230649 | 4002.230652 |
| 4002.230699 | Kernel\_Read\|xilinx:adm-pcie-7v3:1ddr:3.0-0\|cnn\_GOOD | Read | OCL Region | 1 | 1 | 131607 | 131608 | 0.00333333 | 4002.230699 | 4002.230702 |
| 4002.230749 | Kernel\_Read\|xilinx:adm-pcie-7v3:1ddr:3.0-0\|cnn\_GOOD | Read | OCL Region | 1 | 1 | 131622 | 131623 | 0.00333333 | 4002.230749 | 4002.230752 |
| 4002.230799 | Kernel\_Read\|xilinx:adm-pcie-7v3:1ddr:3.0-0\|cnn\_GOOD | Read | OCL Region | 1 | 1 | 131637 | 131638 | 0.00333333 | 4002.230799 | 4002.230802 |
| 4002.230849 | Kernel\_Read\|xilinx:adm-pcie-7v3:1ddr:3.0-0\|cnn\_GOOD | Read | OCL Region | 1 | 1 | 131652 | 131653 | 0.00333333 | 4002.230849 | 4002.230852 |
| 4002.230912 | Kernel\_Read\|xilinx:adm-pcie-7v3:1ddr:3.0-0\|cnn\_GOOD | Read | OCL Region | 1 | 1 | 131671 | 131672 | 0.00333333 | 4002.230912 | 4002.230916 |
| 4002.230962 | Kernel\_Read\|xilinx:adm-pcie-7v3:1ddr:3.0-0\|cnn\_GOOD | Read | OCL Region | 1 | 1 | 131686 | 131687 | 0.00333333 | 4002.230962 | 4002.230966 |
| 4002.231012 | Kernel\_Read\|xilinx:adm-pcie-7v3:1ddr:3.0-0\|cnn\_GOOD | Read | OCL Region | 1 | 1 | 131701 | 131702 | 0.00333333 | 4002.231012 | 4002.231016 |
| 4002.231062 | Kernel\_Read\|xilinx:adm-pcie-7v3:1ddr:3.0-0\|cnn\_GOOD | Read | OCL Region | 1 | 1 | 131716 | 131717 | 0.00333333 | 4002.231062 | 4002.231066 |
| 4002.231112 | Kernel\_Read\|xilinx:adm-pcie-7v3:1ddr:3.0-0\|cnn\_GOOD | Read | OCL Region | 1 | 1 | 131731 | 131732 | 0.00333333 | 4002.231112 | 4002.231116 |
| 4002.231162 | Kernel\_Read\|xilinx:adm-pcie-7v3:1ddr:3.0-0\|cnn\_GOOD | Read | OCL Region | 1 | 1 | 131746 | 131747 | 0.00333333 | 4002.231162 | 4002.231166 |
| 4002.231212 | Kernel\_Read\|xilinx:adm-pcie-7v3:1ddr:3.0-0\|cnn\_GOOD | Read | OCL Region | 1 | 1 | 131761 | 131762 | 0.00333333 | 4002.231212 | 4002.231216 |
| 4002.231262 | Kernel\_Read\|xilinx:adm-pcie-7v3:1ddr:3.0-0\|cnn\_GOOD | Read | OCL Region | 1 | 1 | 131776 | 131777 | 0.00333333 | 4002.231262 | 4002.231266 |
| 4002.231312 | Kernel\_Read\|xilinx:adm-pcie-7v3:1ddr:3.0-0\|cnn\_GOOD | Read | OCL Region | 1 | 1 | 131791 | 131792 | 0.00333333 | 4002.231312 | 4002.231316 |
| 4002.231362 | Kernel\_Read\|xilinx:adm-pcie-7v3:1ddr:3.0-0\|cnn\_GOOD | Read | OCL Region | 1 | 1 | 131806 | 131807 | 0.00333333 | 4002.231362 | 4002.231366 |
| 4002.231412 | Kernel\_Read\|xilinx:adm-pcie-7v3:1ddr:3.0-0\|cnn\_GOOD | Read | OCL Region | 1 | 1 | 131821 | 131822 | 0.00333333 | 4002.231412 | 4002.231416 |
| 4002.231462 | Kernel\_Read\|xilinx:adm-pcie-7v3:1ddr:3.0-0\|cnn\_GOOD | Read | OCL Region | 1 | 1 | 131836 | 131837 | 0.00333333 | 4002.231462 | 4002.231466 |
| 4002.231512 | Kernel\_Read\|xilinx:adm-pcie-7v3:1ddr:3.0-0\|cnn\_GOOD | Read | OCL Region | 1 | 1 | 131851 | 131852 | 0.00333333 | 4002.231512 | 4002.231516 |
| 4002.231562 | Kernel\_Read\|xilinx:adm-pcie-7v3:1ddr:3.0-0\|cnn\_GOOD | Read | OCL Region | 1 | 1 | 131866 | 131867 | 0.00333333 | 4002.231562 | 4002.231566 |
| 4002.231612 | Kernel\_Read\|xilinx:adm-pcie-7v3:1ddr:3.0-0\|cnn\_GOOD | Read | OCL Region | 1 | 1 | 131881 | 131882 | 0.00333333 | 4002.231612 | 4002.231616 |
| 4002.231662 | Kernel\_Read\|xilinx:adm-pcie-7v3:1ddr:3.0-0\|cnn\_GOOD | Read | OCL Region | 1 | 1 | 131896 | 131897 | 0.00333333 | 4002.231662 | 4002.231666 |
| 4002.231929 | Kernel\_Read\|xilinx:adm-pcie-7v3:1ddr:3.0-0\|cnn\_GOOD | Read | OCL Region | 1 | 1 | 131976 | 131977 | 0.00333333 | 4002.231929 | 4002.231932 |
| 4002.231979 | Kernel\_Read\|xilinx:adm-pcie-7v3:1ddr:3.0-0\|cnn\_GOOD | Read | OCL Region | 1 | 1 | 131991 | 131992 | 0.00333333 | 4002.231979 | 4002.231982 |
| 4002.232029 | Kernel\_Read\|xilinx:adm-pcie-7v3:1ddr:3.0-0\|cnn\_GOOD | Read | OCL Region | 1 | 1 | 132006 | 132007 | 0.00333333 | 4002.232029 | 4002.232032 |
| 4002.232079 | Kernel\_Read\|xilinx:adm-pcie-7v3:1ddr:3.0-0\|cnn\_GOOD | Read | OCL Region | 1 | 1 | 132021 | 132022 | 0.00333333 | 4002.232079 | 4002.232082 |
| 4002.232146 | Kernel\_Read\|xilinx:adm-pcie-7v3:1ddr:3.0-0\|cnn\_GOOD | Read | OCL Region | 1 | 1 | 132041 | 132042 | 0.00333333 | 4002.232146 | 4002.232149 |
| 4002.232196 | Kernel\_Read\|xilinx:adm-pcie-7v3:1ddr:3.0-0\|cnn\_GOOD | Read | OCL Region | 1 | 1 | 132056 | 132057 | 0.00333333 | 4002.232196 | 4002.232199 |
| 4002.232246 | Kernel\_Read\|xilinx:adm-pcie-7v3:1ddr:3.0-0\|cnn\_GOOD | Read | OCL Region | 1 | 1 | 132071 | 132072 | 0.00333333 | 4002.232246 | 4002.232249 |
| 4002.232296 | Kernel\_Read\|xilinx:adm-pcie-7v3:1ddr:3.0-0\|cnn\_GOOD | Read | OCL Region | 1 | 1 | 132086 | 132087 | 0.00333333 | 4002.232296 | 4002.232299 |
| 4002.232346 | Kernel\_Read\|xilinx:adm-pcie-7v3:1ddr:3.0-0\|cnn\_GOOD | Read | OCL Region | 1 | 1 | 132101 | 132102 | 0.00333333 | 4002.232346 | 4002.232349 |
| 4002.232396 | Kernel\_Read\|xilinx:adm-pcie-7v3:1ddr:3.0-0\|cnn\_GOOD | Read | OCL Region | 1 | 1 | 132116 | 132117 | 0.00333333 | 4002.232396 | 4002.232399 |
| 4002.232446 | Kernel\_Read\|xilinx:adm-pcie-7v3:1ddr:3.0-0\|cnn\_GOOD | Read | OCL Region | 1 | 1 | 132131 | 132132 | 0.00333333 | 4002.232446 | 4002.232449 |
| 4002.232496 | Kernel\_Read\|xilinx:adm-pcie-7v3:1ddr:3.0-0\|cnn\_GOOD | Read | OCL Region | 1 | 1 | 132146 | 132147 | 0.00333333 | 4002.232496 | 4002.232499 |
| 4002.232546 | Kernel\_Read\|xilinx:adm-pcie-7v3:1ddr:3.0-0\|cnn\_GOOD | Read | OCL Region | 1 | 1 | 132161 | 132162 | 0.00333333 | 4002.232546 | 4002.232549 |
| 4002.232596 | Kernel\_Read\|xilinx:adm-pcie-7v3:1ddr:3.0-0\|cnn\_GOOD | Read | OCL Region | 1 | 1 | 132176 | 132177 | 0.00333333 | 4002.232596 | 4002.232599 |
| 4002.232646 | Kernel\_Read\|xilinx:adm-pcie-7v3:1ddr:3.0-0\|cnn\_GOOD | Read | OCL Region | 1 | 1 | 132191 | 132192 | 0.00333333 | 4002.232646 | 4002.232649 |
| 4002.232696 | Kernel\_Read\|xilinx:adm-pcie-7v3:1ddr:3.0-0\|cnn\_GOOD | Read | OCL Region | 1 | 1 | 132206 | 132207 | 0.00333333 | 4002.232696 | 4002.232699 |
| 4002.232746 | Kernel\_Read\|xilinx:adm-pcie-7v3:1ddr:3.0-0\|cnn\_GOOD | Read | OCL Region | 1 | 1 | 132221 | 132222 | 0.00333333 | 4002.232746 | 4002.232749 |
| 4002.232796 | Kernel\_Read\|xilinx:adm-pcie-7v3:1ddr:3.0-0\|cnn\_GOOD | Read | OCL Region | 1 | 1 | 132236 | 132237 | 0.00333333 | 4002.232796 | 4002.232799 |
| 4002.232846 | Kernel\_Read\|xilinx:adm-pcie-7v3:1ddr:3.0-0\|cnn\_GOOD | Read | OCL Region | 1 | 1 | 132251 | 132252 | 0.00333333 | 4002.232846 | 4002.232849 |
| 4002.232896 | Kernel\_Read\|xilinx:adm-pcie-7v3:1ddr:3.0-0\|cnn\_GOOD | Read | OCL Region | 1 | 1 | 132266 | 132267 | 0.00333333 | 4002.232896 | 4002.232899 |
| 4002.232946 | Kernel\_Read\|xilinx:adm-pcie-7v3:1ddr:3.0-0\|cnn\_GOOD | Read | OCL Region | 1 | 1 | 132281 | 132282 | 0.00333333 | 4002.232946 | 4002.232949 |
| 4002.232996 | Kernel\_Read\|xilinx:adm-pcie-7v3:1ddr:3.0-0\|cnn\_GOOD | Read | OCL Region | 1 | 1 | 132296 | 132297 | 0.00333333 | 4002.232996 | 4002.232999 |
| 4002.233052 | Kernel\_Read\|xilinx:adm-pcie-7v3:1ddr:3.0-0\|cnn\_GOOD | Read | OCL Region | 1 | 1 | 132313 | 132314 | 0.00333333 | 4002.233052 | 4002.233056 |
| 4002.233109 | Kernel\_Read\|xilinx:adm-pcie-7v3:1ddr:3.0-0\|cnn\_GOOD | Read | OCL Region | 1 | 1 | 132330 | 132331 | 0.00333333 | 4002.233109 | 4002.233112 |
| 4002.233152 | Kernel\_Read\|xilinx:adm-pcie-7v3:1ddr:3.0-0\|cnn\_GOOD | Read | OCL Region | 1 | 1 | 132343 | 132344 | 0.00333333 | 4002.233152 | 4002.233156 |
| 4002.233202 | Kernel\_Read\|xilinx:adm-pcie-7v3:1ddr:3.0-0\|cnn\_GOOD | Read | OCL Region | 1 | 1 | 132358 | 132359 | 0.00333333 | 4002.233202 | 4002.233206 |
| 4002.233252 | Kernel\_Read\|xilinx:adm-pcie-7v3:1ddr:3.0-0\|cnn\_GOOD | Read | OCL Region | 1 | 1 | 132373 | 132374 | 0.00333333 | 4002.233252 | 4002.233256 |
| 4002.233302 | Kernel\_Read\|xilinx:adm-pcie-7v3:1ddr:3.0-0\|cnn\_GOOD | Read | OCL Region | 1 | 1 | 132388 | 132389 | 0.00333333 | 4002.233302 | 4002.233306 |
| 4002.233352 | Kernel\_Read\|xilinx:adm-pcie-7v3:1ddr:3.0-0\|cnn\_GOOD | Read | OCL Region | 1 | 1 | 132403 | 132404 | 0.00333333 | 4002.233352 | 4002.233356 |
| 4002.233402 | Kernel\_Read\|xilinx:adm-pcie-7v3:1ddr:3.0-0\|cnn\_GOOD | Read | OCL Region | 1 | 1 | 132418 | 132419 | 0.00333333 | 4002.233402 | 4002.233406 |
| 4002.233452 | Kernel\_Read\|xilinx:adm-pcie-7v3:1ddr:3.0-0\|cnn\_GOOD | Read | OCL Region | 1 | 1 | 132433 | 132434 | 0.00333333 | 4002.233452 | 4002.233456 |
| 4002.233509 | Kernel\_Read\|xilinx:adm-pcie-7v3:1ddr:3.0-0\|cnn\_GOOD | Read | OCL Region | 1 | 1 | 132450 | 132451 | 0.00333333 | 4002.233509 | 4002.233512 |
| 4002.233552 | Kernel\_Read\|xilinx:adm-pcie-7v3:1ddr:3.0-0\|cnn\_GOOD | Read | OCL Region | 1 | 1 | 132463 | 132464 | 0.00333333 | 4002.233552 | 4002.233556 |
| 4002.233602 | Kernel\_Read\|xilinx:adm-pcie-7v3:1ddr:3.0-0\|cnn\_GOOD | Read | OCL Region | 1 | 1 | 132478 | 132479 | 0.00333333 | 4002.233602 | 4002.233606 |
| 4002.233652 | Kernel\_Read\|xilinx:adm-pcie-7v3:1ddr:3.0-0\|cnn\_GOOD | Read | OCL Region | 1 | 1 | 132493 | 132494 | 0.00333333 | 4002.233652 | 4002.233656 |
| 4002.233702 | Kernel\_Read\|xilinx:adm-pcie-7v3:1ddr:3.0-0\|cnn\_GOOD | Read | OCL Region | 1 | 1 | 132508 | 132509 | 0.00333333 | 4002.233702 | 4002.233706 |
| 4002.233752 | Kernel\_Read\|xilinx:adm-pcie-7v3:1ddr:3.0-0\|cnn\_GOOD | Read | OCL Region | 1 | 1 | 132523 | 132524 | 0.00333333 | 4002.233752 | 4002.233756 |
| 4002.233802 | Kernel\_Read\|xilinx:adm-pcie-7v3:1ddr:3.0-0\|cnn\_GOOD | Read | OCL Region | 1 | 1 | 132538 | 132539 | 0.00333333 | 4002.233802 | 4002.233806 |
| 4002.233852 | Kernel\_Read\|xilinx:adm-pcie-7v3:1ddr:3.0-0\|cnn\_GOOD | Read | OCL Region | 1 | 1 | 132553 | 132554 | 0.00333333 | 4002.233852 | 4002.233856 |
| 4002.233902 | Kernel\_Read\|xilinx:adm-pcie-7v3:1ddr:3.0-0\|cnn\_GOOD | Read | OCL Region | 1 | 1 | 132568 | 132569 | 0.00333333 | 4002.233902 | 4002.233906 |
| 4002.233952 | Kernel\_Read\|xilinx:adm-pcie-7v3:1ddr:3.0-0\|cnn\_GOOD | Read | OCL Region | 1 | 1 | 132583 | 132584 | 0.00333333 | 4002.233952 | 4002.233956 |
| 4002.234002 | Kernel\_Read\|xilinx:adm-pcie-7v3:1ddr:3.0-0\|cnn\_GOOD | Read | OCL Region | 1 | 1 | 132598 | 132599 | 0.00333333 | 4002.234002 | 4002.234006 |
| 4002.234066 | Kernel\_Read\|xilinx:adm-pcie-7v3:1ddr:3.0-0\|cnn\_GOOD | Read | OCL Region | 1 | 1 | 132617 | 132618 | 0.00333333 | 4002.234066 | 4002.234069 |
| 4002.234116 | Kernel\_Read\|xilinx:adm-pcie-7v3:1ddr:3.0-0\|cnn\_GOOD | Read | OCL Region | 1 | 1 | 132632 | 132633 | 0.00333333 | 4002.234116 | 4002.234119 |
| 4002.234166 | Kernel\_Read\|xilinx:adm-pcie-7v3:1ddr:3.0-0\|cnn\_GOOD | Read | OCL Region | 1 | 1 | 132647 | 132648 | 0.00333333 | 4002.234166 | 4002.234169 |
| 4002.234216 | Kernel\_Read\|xilinx:adm-pcie-7v3:1ddr:3.0-0\|cnn\_GOOD | Read | OCL Region | 1 | 1 | 132662 | 132663 | 0.00333333 | 4002.234216 | 4002.234219 |
| 4002.234266 | Kernel\_Read\|xilinx:adm-pcie-7v3:1ddr:3.0-0\|cnn\_GOOD | Read | OCL Region | 1 | 1 | 132677 | 132678 | 0.00333333 | 4002.234266 | 4002.234269 |
| 4002.234316 | Kernel\_Read\|xilinx:adm-pcie-7v3:1ddr:3.0-0\|cnn\_GOOD | Read | OCL Region | 1 | 1 | 132692 | 132693 | 0.00333333 | 4002.234316 | 4002.234319 |
| 4002.234366 | Kernel\_Read\|xilinx:adm-pcie-7v3:1ddr:3.0-0\|cnn\_GOOD | Read | OCL Region | 1 | 1 | 132707 | 132708 | 0.00333333 | 4002.234366 | 4002.234369 |
| 4002.234416 | Kernel\_Read\|xilinx:adm-pcie-7v3:1ddr:3.0-0\|cnn\_GOOD | Read | OCL Region | 1 | 1 | 132722 | 132723 | 0.00333333 | 4002.234416 | 4002.234419 |
| 4002.234466 | Kernel\_Read\|xilinx:adm-pcie-7v3:1ddr:3.0-0\|cnn\_GOOD | Read | OCL Region | 1 | 1 | 132737 | 132738 | 0.00333333 | 4002.234466 | 4002.234469 |
| 4002.234516 | Kernel\_Read\|xilinx:adm-pcie-7v3:1ddr:3.0-0\|cnn\_GOOD | Read | OCL Region | 1 | 1 | 132752 | 132753 | 0.00333333 | 4002.234516 | 4002.234519 |
| 4002.234566 | Kernel\_Read\|xilinx:adm-pcie-7v3:1ddr:3.0-0\|cnn\_GOOD | Read | OCL Region | 1 | 1 | 132767 | 132768 | 0.00333333 | 4002.234566 | 4002.234569 |
| 4002.234616 | Kernel\_Read\|xilinx:adm-pcie-7v3:1ddr:3.0-0\|cnn\_GOOD | Read | OCL Region | 1 | 1 | 132782 | 132783 | 0.00333333 | 4002.234616 | 4002.234619 |
| 4002.234666 | Kernel\_Read\|xilinx:adm-pcie-7v3:1ddr:3.0-0\|cnn\_GOOD | Read | OCL Region | 1 | 1 | 132797 | 132798 | 0.00333333 | 4002.234666 | 4002.234669 |
| 4002.234716 | Kernel\_Read\|xilinx:adm-pcie-7v3:1ddr:3.0-0\|cnn\_GOOD | Read | OCL Region | 1 | 1 | 132812 | 132813 | 0.00333333 | 4002.234716 | 4002.234719 |
| 4002.234766 | Kernel\_Read\|xilinx:adm-pcie-7v3:1ddr:3.0-0\|cnn\_GOOD | Read | OCL Region | 1 | 1 | 132827 | 132828 | 0.00333333 | 4002.234766 | 4002.234769 |
| 4002.234816 | Kernel\_Read\|xilinx:adm-pcie-7v3:1ddr:3.0-0\|cnn\_GOOD | Read | OCL Region | 1 | 1 | 132842 | 132843 | 0.00333333 | 4002.234816 | 4002.234819 |
| 4002.234866 | Kernel\_Read\|xilinx:adm-pcie-7v3:1ddr:3.0-0\|cnn\_GOOD | Read | OCL Region | 1 | 1 | 132857 | 132858 | 0.00333333 | 4002.234866 | 4002.234869 |
| 4002.234916 | Kernel\_Read\|xilinx:adm-pcie-7v3:1ddr:3.0-0\|cnn\_GOOD | Read | OCL Region | 1 | 1 | 132872 | 132873 | 0.00333333 | 4002.234916 | 4002.234919 |
| 4002.234966 | Kernel\_Read\|xilinx:adm-pcie-7v3:1ddr:3.0-0\|cnn\_GOOD | Read | OCL Region | 1 | 1 | 132887 | 132888 | 0.00333333 | 4002.234966 | 4002.234969 |
| 4002.235026 | Kernel\_Read\|xilinx:adm-pcie-7v3:1ddr:3.0-0\|cnn\_GOOD | Read | OCL Region | 1 | 1 | 132905 | 132906 | 0.00333333 | 4002.235026 | 4002.235029 |
| 4002.235076 | Kernel\_Read\|xilinx:adm-pcie-7v3:1ddr:3.0-0\|cnn\_GOOD | Read | OCL Region | 1 | 1 | 132920 | 132921 | 0.00333333 | 4002.235076 | 4002.235079 |
| 4002.235126 | Kernel\_Read\|xilinx:adm-pcie-7v3:1ddr:3.0-0\|cnn\_GOOD | Read | OCL Region | 1 | 1 | 132935 | 132936 | 0.00333333 | 4002.235126 | 4002.235129 |
| 4002.235176 | Kernel\_Read\|xilinx:adm-pcie-7v3:1ddr:3.0-0\|cnn\_GOOD | Read | OCL Region | 1 | 1 | 132950 | 132951 | 0.00333333 | 4002.235176 | 4002.235179 |
| 4002.235226 | Kernel\_Read\|xilinx:adm-pcie-7v3:1ddr:3.0-0\|cnn\_GOOD | Read | OCL Region | 1 | 1 | 132965 | 132966 | 0.00333333 | 4002.235226 | 4002.235229 |
| 4002.235276 | Kernel\_Read\|xilinx:adm-pcie-7v3:1ddr:3.0-0\|cnn\_GOOD | Read | OCL Region | 1 | 1 | 132980 | 132981 | 0.00333333 | 4002.235276 | 4002.235279 |
| 4002.235326 | Kernel\_Read\|xilinx:adm-pcie-7v3:1ddr:3.0-0\|cnn\_GOOD | Read | OCL Region | 1 | 1 | 132995 | 132996 | 0.00333333 | 4002.235326 | 4002.235329 |
| 4002.235376 | Kernel\_Read\|xilinx:adm-pcie-7v3:1ddr:3.0-0\|cnn\_GOOD | Read | OCL Region | 1 | 1 | 133010 | 133011 | 0.00333333 | 4002.235376 | 4002.235379 |
| 4002.235426 | Kernel\_Read\|xilinx:adm-pcie-7v3:1ddr:3.0-0\|cnn\_GOOD | Read | OCL Region | 1 | 1 | 133025 | 133026 | 0.00333333 | 4002.235426 | 4002.235429 |
| 4002.235476 | Kernel\_Read\|xilinx:adm-pcie-7v3:1ddr:3.0-0\|cnn\_GOOD | Read | OCL Region | 1 | 1 | 133040 | 133041 | 0.00333333 | 4002.235476 | 4002.235479 |
| 4002.235526 | Kernel\_Read\|xilinx:adm-pcie-7v3:1ddr:3.0-0\|cnn\_GOOD | Read | OCL Region | 1 | 1 | 133055 | 133056 | 0.00333333 | 4002.235526 | 4002.235529 |
| 4002.235576 | Kernel\_Read\|xilinx:adm-pcie-7v3:1ddr:3.0-0\|cnn\_GOOD | Read | OCL Region | 1 | 1 | 133070 | 133071 | 0.00333333 | 4002.235576 | 4002.235579 |
| 4002.235626 | Kernel\_Read\|xilinx:adm-pcie-7v3:1ddr:3.0-0\|cnn\_GOOD | Read | OCL Region | 1 | 1 | 133085 | 133086 | 0.00333333 | 4002.235626 | 4002.235629 |
| 4002.235676 | Kernel\_Read\|xilinx:adm-pcie-7v3:1ddr:3.0-0\|cnn\_GOOD | Read | OCL Region | 1 | 1 | 133100 | 133101 | 0.00333333 | 4002.235676 | 4002.235679 |
| 4002.235726 | Kernel\_Read\|xilinx:adm-pcie-7v3:1ddr:3.0-0\|cnn\_GOOD | Read | OCL Region | 1 | 1 | 133115 | 133116 | 0.00333333 | 4002.235726 | 4002.235729 |
| 4002.235776 | Kernel\_Read\|xilinx:adm-pcie-7v3:1ddr:3.0-0\|cnn\_GOOD | Read | OCL Region | 1 | 1 | 133130 | 133131 | 0.00333333 | 4002.235776 | 4002.235779 |
| 4002.235826 | Kernel\_Read\|xilinx:adm-pcie-7v3:1ddr:3.0-0\|cnn\_GOOD | Read | OCL Region | 1 | 1 | 133145 | 133146 | 0.00333333 | 4002.235826 | 4002.235829 |
| 4002.235876 | Kernel\_Read\|xilinx:adm-pcie-7v3:1ddr:3.0-0\|cnn\_GOOD | Read | OCL Region | 1 | 1 | 133160 | 133161 | 0.00333333 | 4002.235876 | 4002.235879 |
| 4002.235926 | Kernel\_Read\|xilinx:adm-pcie-7v3:1ddr:3.0-0\|cnn\_GOOD | Read | OCL Region | 1 | 1 | 133175 | 133176 | 0.00333333 | 4002.235926 | 4002.235929 |
| 4002.235976 | Kernel\_Read\|xilinx:adm-pcie-7v3:1ddr:3.0-0\|cnn\_GOOD | Read | OCL Region | 1 | 1 | 133190 | 133191 | 0.00333333 | 4002.235976 | 4002.235979 |
| 4002.236036 | Kernel\_Read\|xilinx:adm-pcie-7v3:1ddr:3.0-0\|cnn\_GOOD | Read | OCL Region | 1 | 1 | 133208 | 133209 | 0.00333333 | 4002.236036 | 4002.236039 |
| 4002.236086 | Kernel\_Read\|xilinx:adm-pcie-7v3:1ddr:3.0-0\|cnn\_GOOD | Read | OCL Region | 1 | 1 | 133223 | 133224 | 0.00333333 | 4002.236086 | 4002.236089 |
| 4002.236136 | Kernel\_Read\|xilinx:adm-pcie-7v3:1ddr:3.0-0\|cnn\_GOOD | Read | OCL Region | 1 | 1 | 133238 | 133239 | 0.00333333 | 4002.236136 | 4002.236139 |
| 4002.236186 | Kernel\_Read\|xilinx:adm-pcie-7v3:1ddr:3.0-0\|cnn\_GOOD | Read | OCL Region | 1 | 1 | 133253 | 133254 | 0.00333333 | 4002.236186 | 4002.236189 |
| 4002.236236 | Kernel\_Read\|xilinx:adm-pcie-7v3:1ddr:3.0-0\|cnn\_GOOD | Read | OCL Region | 1 | 1 | 133268 | 133269 | 0.00333333 | 4002.236236 | 4002.236239 |
| 4002.236286 | Kernel\_Read\|xilinx:adm-pcie-7v3:1ddr:3.0-0\|cnn\_GOOD | Read | OCL Region | 1 | 1 | 133283 | 133284 | 0.00333333 | 4002.236286 | 4002.236289 |
| 4002.236336 | Kernel\_Read\|xilinx:adm-pcie-7v3:1ddr:3.0-0\|cnn\_GOOD | Read | OCL Region | 1 | 1 | 133298 | 133299 | 0.00333333 | 4002.236336 | 4002.236339 |
| 4002.236386 | Kernel\_Read\|xilinx:adm-pcie-7v3:1ddr:3.0-0\|cnn\_GOOD | Read | OCL Region | 1 | 1 | 133313 | 133314 | 0.00333333 | 4002.236386 | 4002.236389 |
| 4002.236436 | Kernel\_Read\|xilinx:adm-pcie-7v3:1ddr:3.0-0\|cnn\_GOOD | Read | OCL Region | 1 | 1 | 133328 | 133329 | 0.00333333 | 4002.236436 | 4002.236439 |
| 4002.236486 | Kernel\_Read\|xilinx:adm-pcie-7v3:1ddr:3.0-0\|cnn\_GOOD | Read | OCL Region | 1 | 1 | 133343 | 133344 | 0.00333333 | 4002.236486 | 4002.236489 |
| 4002.236536 | Kernel\_Read\|xilinx:adm-pcie-7v3:1ddr:3.0-0\|cnn\_GOOD | Read | OCL Region | 1 | 1 | 133358 | 133359 | 0.00333333 | 4002.236536 | 4002.236539 |
| 4002.236586 | Kernel\_Read\|xilinx:adm-pcie-7v3:1ddr:3.0-0\|cnn\_GOOD | Read | OCL Region | 1 | 1 | 133373 | 133374 | 0.00333333 | 4002.236586 | 4002.236589 |
| 4002.236636 | Kernel\_Read\|xilinx:adm-pcie-7v3:1ddr:3.0-0\|cnn\_GOOD | Read | OCL Region | 1 | 1 | 133388 | 133389 | 0.00333333 | 4002.236636 | 4002.236639 |
| 4002.236686 | Kernel\_Read\|xilinx:adm-pcie-7v3:1ddr:3.0-0\|cnn\_GOOD | Read | OCL Region | 1 | 1 | 133403 | 133404 | 0.00333333 | 4002.236686 | 4002.236689 |
| 4002.236736 | Kernel\_Read\|xilinx:adm-pcie-7v3:1ddr:3.0-0\|cnn\_GOOD | Read | OCL Region | 1 | 1 | 133418 | 133419 | 0.00333333 | 4002.236736 | 4002.236739 |
| 4002.236786 | Kernel\_Read\|xilinx:adm-pcie-7v3:1ddr:3.0-0\|cnn\_GOOD | Read | OCL Region | 1 | 1 | 133433 | 133434 | 0.00333333 | 4002.236786 | 4002.236789 |
| 4002.236836 | Kernel\_Read\|xilinx:adm-pcie-7v3:1ddr:3.0-0\|cnn\_GOOD | Read | OCL Region | 1 | 1 | 133448 | 133449 | 0.00333333 | 4002.236836 | 4002.236839 |
| 4002.236886 | Kernel\_Read\|xilinx:adm-pcie-7v3:1ddr:3.0-0\|cnn\_GOOD | Read | OCL Region | 1 | 1 | 133463 | 133464 | 0.00333333 | 4002.236886 | 4002.236889 |
| 4002.236936 | Kernel\_Read\|xilinx:adm-pcie-7v3:1ddr:3.0-0\|cnn\_GOOD | Read | OCL Region | 1 | 1 | 133478 | 133479 | 0.00333333 | 4002.236936 | 4002.236939 |
| 4002.236996 | Kernel\_Read\|xilinx:adm-pcie-7v3:1ddr:3.0-0\|cnn\_GOOD | Read | OCL Region | 1 | 1 | 133496 | 133497 | 0.00333333 | 4002.236996 | 4002.236999 |
| 4002.237046 | Kernel\_Read\|xilinx:adm-pcie-7v3:1ddr:3.0-0\|cnn\_GOOD | Read | OCL Region | 1 | 1 | 133511 | 133512 | 0.00333333 | 4002.237046 | 4002.237049 |
| 4002.237096 | Kernel\_Read\|xilinx:adm-pcie-7v3:1ddr:3.0-0\|cnn\_GOOD | Read | OCL Region | 1 | 1 | 133526 | 133527 | 0.00333333 | 4002.237096 | 4002.237099 |
| 4002.237146 | Kernel\_Read\|xilinx:adm-pcie-7v3:1ddr:3.0-0\|cnn\_GOOD | Read | OCL Region | 1 | 1 | 133541 | 133542 | 0.00333333 | 4002.237146 | 4002.237149 |
| 4002.237196 | Kernel\_Read\|xilinx:adm-pcie-7v3:1ddr:3.0-0\|cnn\_GOOD | Read | OCL Region | 1 | 1 | 133556 | 133557 | 0.00333333 | 4002.237196 | 4002.237199 |
| 4002.237246 | Kernel\_Read\|xilinx:adm-pcie-7v3:1ddr:3.0-0\|cnn\_GOOD | Read | OCL Region | 1 | 1 | 133571 | 133572 | 0.00333333 | 4002.237246 | 4002.237249 |
| 4002.237296 | Kernel\_Read\|xilinx:adm-pcie-7v3:1ddr:3.0-0\|cnn\_GOOD | Read | OCL Region | 1 | 1 | 133586 | 133587 | 0.00333333 | 4002.237296 | 4002.237299 |
| 4002.237346 | Kernel\_Read\|xilinx:adm-pcie-7v3:1ddr:3.0-0\|cnn\_GOOD | Read | OCL Region | 1 | 1 | 133601 | 133602 | 0.00333333 | 4002.237346 | 4002.237349 |
| 4002.237396 | Kernel\_Read\|xilinx:adm-pcie-7v3:1ddr:3.0-0\|cnn\_GOOD | Read | OCL Region | 1 | 1 | 133616 | 133617 | 0.00333333 | 4002.237396 | 4002.237399 |
| 4002.237446 | Kernel\_Read\|xilinx:adm-pcie-7v3:1ddr:3.0-0\|cnn\_GOOD | Read | OCL Region | 1 | 1 | 133631 | 133632 | 0.00333333 | 4002.237446 | 4002.237449 |
| 4002.237496 | Kernel\_Read\|xilinx:adm-pcie-7v3:1ddr:3.0-0\|cnn\_GOOD | Read | OCL Region | 1 | 1 | 133646 | 133647 | 0.00333333 | 4002.237496 | 4002.237499 |
| 4002.237546 | Kernel\_Read\|xilinx:adm-pcie-7v3:1ddr:3.0-0\|cnn\_GOOD | Read | OCL Region | 1 | 1 | 133661 | 133662 | 0.00333333 | 4002.237546 | 4002.237549 |
| 4002.237596 | Kernel\_Read\|xilinx:adm-pcie-7v3:1ddr:3.0-0\|cnn\_GOOD | Read | OCL Region | 1 | 1 | 133676 | 133677 | 0.00333333 | 4002.237596 | 4002.237599 |
| 4002.237646 | Kernel\_Read\|xilinx:adm-pcie-7v3:1ddr:3.0-0\|cnn\_GOOD | Read | OCL Region | 1 | 1 | 133691 | 133692 | 0.00333333 | 4002.237646 | 4002.237649 |
| 4002.237696 | Kernel\_Read\|xilinx:adm-pcie-7v3:1ddr:3.0-0\|cnn\_GOOD | Read | OCL Region | 1 | 1 | 133706 | 133707 | 0.00333333 | 4002.237696 | 4002.237699 |
| 4002.237746 | Kernel\_Read\|xilinx:adm-pcie-7v3:1ddr:3.0-0\|cnn\_GOOD | Read | OCL Region | 1 | 1 | 133721 | 133722 | 0.00333333 | 4002.237746 | 4002.237749 |
| 4002.237796 | Kernel\_Read\|xilinx:adm-pcie-7v3:1ddr:3.0-0\|cnn\_GOOD | Read | OCL Region | 1 | 1 | 133736 | 133737 | 0.00333333 | 4002.237796 | 4002.237799 |
| 4002.237846 | Kernel\_Read\|xilinx:adm-pcie-7v3:1ddr:3.0-0\|cnn\_GOOD | Read | OCL Region | 1 | 1 | 133751 | 133752 | 0.00333333 | 4002.237846 | 4002.237849 |
| 4002.237896 | Kernel\_Read\|xilinx:adm-pcie-7v3:1ddr:3.0-0\|cnn\_GOOD | Read | OCL Region | 1 | 1 | 133766 | 133767 | 0.00333333 | 4002.237896 | 4002.237899 |
| 4002.237946 | Kernel\_Read\|xilinx:adm-pcie-7v3:1ddr:3.0-0\|cnn\_GOOD | Read | OCL Region | 1 | 1 | 133781 | 133782 | 0.00333333 | 4002.237946 | 4002.237949 |
| 4002.238226 | Kernel\_Read\|xilinx:adm-pcie-7v3:1ddr:3.0-0\|cnn\_GOOD | Read | OCL Region | 1 | 1 | 133865 | 133866 | 0.00333333 | 4002.238226 | 4002.238229 |
| 4002.238276 | Kernel\_Read\|xilinx:adm-pcie-7v3:1ddr:3.0-0\|cnn\_GOOD | Read | OCL Region | 1 | 1 | 133880 | 133881 | 0.00333333 | 4002.238276 | 4002.238279 |
| 4002.238329 | Kernel\_Read\|xilinx:adm-pcie-7v3:1ddr:3.0-0\|cnn\_GOOD | Read | OCL Region | 1 | 1 | 133896 | 133897 | 0.00333333 | 4002.238329 | 4002.238332 |
| 4002.238379 | Kernel\_Read\|xilinx:adm-pcie-7v3:1ddr:3.0-0\|cnn\_GOOD | Read | OCL Region | 1 | 1 | 133911 | 133912 | 0.00333333 | 4002.238379 | 4002.238382 |
| 4002.238429 | Kernel\_Read\|xilinx:adm-pcie-7v3:1ddr:3.0-0\|cnn\_GOOD | Read | OCL Region | 1 | 1 | 133926 | 133927 | 0.00333333 | 4002.238429 | 4002.238432 |
| 4002.238479 | Kernel\_Read\|xilinx:adm-pcie-7v3:1ddr:3.0-0\|cnn\_GOOD | Read | OCL Region | 1 | 1 | 133941 | 133942 | 0.00333333 | 4002.238479 | 4002.238482 |
| 4002.238529 | Kernel\_Read\|xilinx:adm-pcie-7v3:1ddr:3.0-0\|cnn\_GOOD | Read | OCL Region | 1 | 1 | 133956 | 133957 | 0.00333333 | 4002.238529 | 4002.238532 |
| 4002.238579 | Kernel\_Read\|xilinx:adm-pcie-7v3:1ddr:3.0-0\|cnn\_GOOD | Read | OCL Region | 1 | 1 | 133971 | 133972 | 0.00333333 | 4002.238579 | 4002.238582 |
| 4002.238629 | Kernel\_Read\|xilinx:adm-pcie-7v3:1ddr:3.0-0\|cnn\_GOOD | Read | OCL Region | 1 | 1 | 133986 | 133987 | 0.00333333 | 4002.238629 | 4002.238632 |
| 4002.238679 | Kernel\_Read\|xilinx:adm-pcie-7v3:1ddr:3.0-0\|cnn\_GOOD | Read | OCL Region | 1 | 1 | 134001 | 134002 | 0.00333333 | 4002.238679 | 4002.238682 |
| 4002.238729 | Kernel\_Read\|xilinx:adm-pcie-7v3:1ddr:3.0-0\|cnn\_GOOD | Read | OCL Region | 1 | 1 | 134016 | 134017 | 0.00333333 | 4002.238729 | 4002.238732 |
| 4002.238779 | Kernel\_Read\|xilinx:adm-pcie-7v3:1ddr:3.0-0\|cnn\_GOOD | Read | OCL Region | 1 | 1 | 134031 | 134032 | 0.00333333 | 4002.238779 | 4002.238782 |
| 4002.238829 | Kernel\_Read\|xilinx:adm-pcie-7v3:1ddr:3.0-0\|cnn\_GOOD | Read | OCL Region | 1 | 1 | 134046 | 134047 | 0.00333333 | 4002.238829 | 4002.238832 |
| 4002.238879 | Kernel\_Read\|xilinx:adm-pcie-7v3:1ddr:3.0-0\|cnn\_GOOD | Read | OCL Region | 1 | 1 | 134061 | 134062 | 0.00333333 | 4002.238879 | 4002.238882 |
| 4002.238929 | Kernel\_Read\|xilinx:adm-pcie-7v3:1ddr:3.0-0\|cnn\_GOOD | Read | OCL Region | 1 | 1 | 134076 | 134077 | 0.00333333 | 4002.238929 | 4002.238932 |
| 4002.238979 | Kernel\_Read\|xilinx:adm-pcie-7v3:1ddr:3.0-0\|cnn\_GOOD | Read | OCL Region | 1 | 1 | 134091 | 134092 | 0.00333333 | 4002.238979 | 4002.238982 |
| 4002.239029 | Kernel\_Read\|xilinx:adm-pcie-7v3:1ddr:3.0-0\|cnn\_GOOD | Read | OCL Region | 1 | 1 | 134106 | 134107 | 0.00333333 | 4002.239029 | 4002.239032 |
| 4002.239079 | Kernel\_Read\|xilinx:adm-pcie-7v3:1ddr:3.0-0\|cnn\_GOOD | Read | OCL Region | 1 | 1 | 134121 | 134122 | 0.00333333 | 4002.239079 | 4002.239082 |
| 4002.239142 | Kernel\_Read\|xilinx:adm-pcie-7v3:1ddr:3.0-0\|cnn\_GOOD | Read | OCL Region | 1 | 1 | 134140 | 134141 | 0.00333333 | 4002.239142 | 4002.239146 |
| 4002.239192 | Kernel\_Read\|xilinx:adm-pcie-7v3:1ddr:3.0-0\|cnn\_GOOD | Read | OCL Region | 1 | 1 | 134155 | 134156 | 0.00333333 | 4002.239192 | 4002.239196 |
| 4002.239242 | Kernel\_Read\|xilinx:adm-pcie-7v3:1ddr:3.0-0\|cnn\_GOOD | Read | OCL Region | 1 | 1 | 134170 | 134171 | 0.00333333 | 4002.239242 | 4002.239246 |
| 4002.239292 | Kernel\_Read\|xilinx:adm-pcie-7v3:1ddr:3.0-0\|cnn\_GOOD | Read | OCL Region | 1 | 1 | 134185 | 134186 | 0.00333333 | 4002.239292 | 4002.239296 |
| 4002.239342 | Kernel\_Read\|xilinx:adm-pcie-7v3:1ddr:3.0-0\|cnn\_GOOD | Read | OCL Region | 1 | 1 | 134200 | 134201 | 0.00333333 | 4002.239342 | 4002.239346 |
| 4002.239392 | Kernel\_Read\|xilinx:adm-pcie-7v3:1ddr:3.0-0\|cnn\_GOOD | Read | OCL Region | 1 | 1 | 134215 | 134216 | 0.00333333 | 4002.239392 | 4002.239396 |
| 4002.239442 | Kernel\_Read\|xilinx:adm-pcie-7v3:1ddr:3.0-0\|cnn\_GOOD | Read | OCL Region | 1 | 1 | 134230 | 134231 | 0.00333333 | 4002.239442 | 4002.239446 |
| 4002.239492 | Kernel\_Read\|xilinx:adm-pcie-7v3:1ddr:3.0-0\|cnn\_GOOD | Read | OCL Region | 1 | 1 | 134245 | 134246 | 0.00333333 | 4002.239492 | 4002.239496 |
| 4002.239542 | Kernel\_Read\|xilinx:adm-pcie-7v3:1ddr:3.0-0\|cnn\_GOOD | Read | OCL Region | 1 | 1 | 134260 | 134261 | 0.00333333 | 4002.239542 | 4002.239546 |
| 4002.239592 | Kernel\_Read\|xilinx:adm-pcie-7v3:1ddr:3.0-0\|cnn\_GOOD | Read | OCL Region | 1 | 1 | 134275 | 134276 | 0.00333333 | 4002.239592 | 4002.239596 |
| 4002.239642 | Kernel\_Read\|xilinx:adm-pcie-7v3:1ddr:3.0-0\|cnn\_GOOD | Read | OCL Region | 1 | 1 | 134290 | 134291 | 0.00333333 | 4002.239642 | 4002.239646 |
| 4002.239692 | Kernel\_Read\|xilinx:adm-pcie-7v3:1ddr:3.0-0\|cnn\_GOOD | Read | OCL Region | 1 | 1 | 134305 | 134306 | 0.00333333 | 4002.239692 | 4002.239696 |
| 4002.239742 | Kernel\_Read\|xilinx:adm-pcie-7v3:1ddr:3.0-0\|cnn\_GOOD | Read | OCL Region | 1 | 1 | 134320 | 134321 | 0.00333333 | 4002.239742 | 4002.239746 |
| 4002.239792 | Kernel\_Read\|xilinx:adm-pcie-7v3:1ddr:3.0-0\|cnn\_GOOD | Read | OCL Region | 1 | 1 | 134335 | 134336 | 0.00333333 | 4002.239792 | 4002.239796 |
| 4002.239842 | Kernel\_Read\|xilinx:adm-pcie-7v3:1ddr:3.0-0\|cnn\_GOOD | Read | OCL Region | 1 | 1 | 134350 | 134351 | 0.00333333 | 4002.239842 | 4002.239846 |
| 4002.239892 | Kernel\_Read\|xilinx:adm-pcie-7v3:1ddr:3.0-0\|cnn\_GOOD | Read | OCL Region | 1 | 1 | 134365 | 134366 | 0.00333333 | 4002.239892 | 4002.239896 |
| 4002.239942 | Kernel\_Read\|xilinx:adm-pcie-7v3:1ddr:3.0-0\|cnn\_GOOD | Read | OCL Region | 1 | 1 | 134380 | 134381 | 0.00333333 | 4002.239942 | 4002.239946 |
| 4002.239992 | Kernel\_Read\|xilinx:adm-pcie-7v3:1ddr:3.0-0\|cnn\_GOOD | Read | OCL Region | 1 | 1 | 134395 | 134396 | 0.00333333 | 4002.239992 | 4002.239996 |
| 4002.240042 | Kernel\_Read\|xilinx:adm-pcie-7v3:1ddr:3.0-0\|cnn\_GOOD | Read | OCL Region | 1 | 1 | 134410 | 134411 | 0.00333333 | 4002.240042 | 4002.240046 |
| 4002.240092 | Kernel\_Read\|xilinx:adm-pcie-7v3:1ddr:3.0-0\|cnn\_GOOD | Read | OCL Region | 1 | 1 | 134425 | 134426 | 0.00333333 | 4002.240092 | 4002.240096 |
| 4002.240149 | Kernel\_Read\|xilinx:adm-pcie-7v3:1ddr:3.0-0\|cnn\_GOOD | Read | OCL Region | 1 | 1 | 134442 | 134443 | 0.00333333 | 4002.240149 | 4002.240152 |
| 4002.240199 | Kernel\_Read\|xilinx:adm-pcie-7v3:1ddr:3.0-0\|cnn\_GOOD | Read | OCL Region | 1 | 1 | 134457 | 134458 | 0.00333333 | 4002.240199 | 4002.240202 |
| 4002.240249 | Kernel\_Read\|xilinx:adm-pcie-7v3:1ddr:3.0-0\|cnn\_GOOD | Read | OCL Region | 1 | 1 | 134472 | 134473 | 0.00333333 | 4002.240249 | 4002.240252 |
| 4002.240299 | Kernel\_Read\|xilinx:adm-pcie-7v3:1ddr:3.0-0\|cnn\_GOOD | Read | OCL Region | 1 | 1 | 134487 | 134488 | 0.00333333 | 4002.240299 | 4002.240302 |
| 4002.240349 | Kernel\_Read\|xilinx:adm-pcie-7v3:1ddr:3.0-0\|cnn\_GOOD | Read | OCL Region | 1 | 1 | 134502 | 134503 | 0.00333333 | 4002.240349 | 4002.240352 |
| 4002.240399 | Kernel\_Read\|xilinx:adm-pcie-7v3:1ddr:3.0-0\|cnn\_GOOD | Read | OCL Region | 1 | 1 | 134517 | 134518 | 0.00333333 | 4002.240399 | 4002.240402 |
| 4002.240449 | Kernel\_Read\|xilinx:adm-pcie-7v3:1ddr:3.0-0\|cnn\_GOOD | Read | OCL Region | 1 | 1 | 134532 | 134533 | 0.00333333 | 4002.240449 | 4002.240452 |
| 4002.240499 | Kernel\_Read\|xilinx:adm-pcie-7v3:1ddr:3.0-0\|cnn\_GOOD | Read | OCL Region | 1 | 1 | 134547 | 134548 | 0.00333333 | 4002.240499 | 4002.240502 |
| 4002.240549 | Kernel\_Read\|xilinx:adm-pcie-7v3:1ddr:3.0-0\|cnn\_GOOD | Read | OCL Region | 1 | 1 | 134562 | 134563 | 0.00333333 | 4002.240549 | 4002.240552 |
| 4002.240599 | Kernel\_Read\|xilinx:adm-pcie-7v3:1ddr:3.0-0\|cnn\_GOOD | Read | OCL Region | 1 | 1 | 134577 | 134578 | 0.00333333 | 4002.240599 | 4002.240602 |
| 4002.240649 | Kernel\_Read\|xilinx:adm-pcie-7v3:1ddr:3.0-0\|cnn\_GOOD | Read | OCL Region | 1 | 1 | 134592 | 134593 | 0.00333333 | 4002.240649 | 4002.240652 |
| 4002.240699 | Kernel\_Read\|xilinx:adm-pcie-7v3:1ddr:3.0-0\|cnn\_GOOD | Read | OCL Region | 1 | 1 | 134607 | 134608 | 0.00333333 | 4002.240699 | 4002.240702 |
| 4002.240749 | Kernel\_Read\|xilinx:adm-pcie-7v3:1ddr:3.0-0\|cnn\_GOOD | Read | OCL Region | 1 | 1 | 134622 | 134623 | 0.00333333 | 4002.240749 | 4002.240752 |
| 4002.240799 | Kernel\_Read\|xilinx:adm-pcie-7v3:1ddr:3.0-0\|cnn\_GOOD | Read | OCL Region | 1 | 1 | 134637 | 134638 | 0.00333333 | 4002.240799 | 4002.240802 |
| 4002.240849 | Kernel\_Read\|xilinx:adm-pcie-7v3:1ddr:3.0-0\|cnn\_GOOD | Read | OCL Region | 1 | 1 | 134652 | 134653 | 0.00333333 | 4002.240849 | 4002.240852 |
| 4002.240899 | Kernel\_Read\|xilinx:adm-pcie-7v3:1ddr:3.0-0\|cnn\_GOOD | Read | OCL Region | 1 | 1 | 134667 | 134668 | 0.00333333 | 4002.240899 | 4002.240902 |
| 4002.240949 | Kernel\_Read\|xilinx:adm-pcie-7v3:1ddr:3.0-0\|cnn\_GOOD | Read | OCL Region | 1 | 1 | 134682 | 134683 | 0.00333333 | 4002.240949 | 4002.240952 |
| 4002.240999 | Kernel\_Read\|xilinx:adm-pcie-7v3:1ddr:3.0-0\|cnn\_GOOD | Read | OCL Region | 1 | 1 | 134697 | 134698 | 0.00333333 | 4002.240999 | 4002.241002 |
| 4002.241049 | Kernel\_Read\|xilinx:adm-pcie-7v3:1ddr:3.0-0\|cnn\_GOOD | Read | OCL Region | 1 | 1 | 134712 | 134713 | 0.00333333 | 4002.241049 | 4002.241052 |
| 4002.241099 | Kernel\_Read\|xilinx:adm-pcie-7v3:1ddr:3.0-0\|cnn\_GOOD | Read | OCL Region | 1 | 1 | 134727 | 134728 | 0.00333333 | 4002.241099 | 4002.241102 |
| 4002.241162 | Kernel\_Read\|xilinx:adm-pcie-7v3:1ddr:3.0-0\|cnn\_GOOD | Read | OCL Region | 1 | 1 | 134746 | 134747 | 0.00333333 | 4002.241162 | 4002.241166 |
| 4002.241212 | Kernel\_Read\|xilinx:adm-pcie-7v3:1ddr:3.0-0\|cnn\_GOOD | Read | OCL Region | 1 | 1 | 134761 | 134762 | 0.00333333 | 4002.241212 | 4002.241216 |
| 4002.241262 | Kernel\_Read\|xilinx:adm-pcie-7v3:1ddr:3.0-0\|cnn\_GOOD | Read | OCL Region | 1 | 1 | 134776 | 134777 | 0.00333333 | 4002.241262 | 4002.241266 |
| 4002.241312 | Kernel\_Read\|xilinx:adm-pcie-7v3:1ddr:3.0-0\|cnn\_GOOD | Read | OCL Region | 1 | 1 | 134791 | 134792 | 0.00333333 | 4002.241312 | 4002.241316 |
| 4002.241362 | Kernel\_Read\|xilinx:adm-pcie-7v3:1ddr:3.0-0\|cnn\_GOOD | Read | OCL Region | 1 | 1 | 134806 | 134807 | 0.00333333 | 4002.241362 | 4002.241366 |
| 4002.241412 | Kernel\_Read\|xilinx:adm-pcie-7v3:1ddr:3.0-0\|cnn\_GOOD | Read | OCL Region | 1 | 1 | 134821 | 134822 | 0.00333333 | 4002.241412 | 4002.241416 |
| 4002.241462 | Kernel\_Read\|xilinx:adm-pcie-7v3:1ddr:3.0-0\|cnn\_GOOD | Read | OCL Region | 1 | 1 | 134836 | 134837 | 0.00333333 | 4002.241462 | 4002.241466 |
| 4002.241512 | Kernel\_Read\|xilinx:adm-pcie-7v3:1ddr:3.0-0\|cnn\_GOOD | Read | OCL Region | 1 | 1 | 134851 | 134852 | 0.00333333 | 4002.241512 | 4002.241516 |
| 4002.241562 | Kernel\_Read\|xilinx:adm-pcie-7v3:1ddr:3.0-0\|cnn\_GOOD | Read | OCL Region | 1 | 1 | 134866 | 134867 | 0.00333333 | 4002.241562 | 4002.241566 |
| 4002.241612 | Kernel\_Read\|xilinx:adm-pcie-7v3:1ddr:3.0-0\|cnn\_GOOD | Read | OCL Region | 1 | 1 | 134881 | 134882 | 0.00333333 | 4002.241612 | 4002.241616 |
| 4002.241662 | Kernel\_Read\|xilinx:adm-pcie-7v3:1ddr:3.0-0\|cnn\_GOOD | Read | OCL Region | 1 | 1 | 134896 | 134897 | 0.00333333 | 4002.241662 | 4002.241666 |
| 4002.241712 | Kernel\_Read\|xilinx:adm-pcie-7v3:1ddr:3.0-0\|cnn\_GOOD | Read | OCL Region | 1 | 1 | 134911 | 134912 | 0.00333333 | 4002.241712 | 4002.241716 |
| 4002.241762 | Kernel\_Read\|xilinx:adm-pcie-7v3:1ddr:3.0-0\|cnn\_GOOD | Read | OCL Region | 1 | 1 | 134926 | 134927 | 0.00333333 | 4002.241762 | 4002.241766 |
| 4002.241812 | Kernel\_Read\|xilinx:adm-pcie-7v3:1ddr:3.0-0\|cnn\_GOOD | Read | OCL Region | 1 | 1 | 134941 | 134942 | 0.00333333 | 4002.241812 | 4002.241816 |
| 4002.241862 | Kernel\_Read\|xilinx:adm-pcie-7v3:1ddr:3.0-0\|cnn\_GOOD | Read | OCL Region | 1 | 1 | 134956 | 134957 | 0.00333333 | 4002.241862 | 4002.241866 |
| 4002.241912 | Kernel\_Read\|xilinx:adm-pcie-7v3:1ddr:3.0-0\|cnn\_GOOD | Read | OCL Region | 1 | 1 | 134971 | 134972 | 0.00333333 | 4002.241912 | 4002.241916 |
| 4002.241962 | Kernel\_Read\|xilinx:adm-pcie-7v3:1ddr:3.0-0\|cnn\_GOOD | Read | OCL Region | 1 | 1 | 134986 | 134987 | 0.00333333 | 4002.241962 | 4002.241966 |
| 4002.242012 | Kernel\_Read\|xilinx:adm-pcie-7v3:1ddr:3.0-0\|cnn\_GOOD | Read | OCL Region | 1 | 1 | 135001 | 135002 | 0.00333333 | 4002.242012 | 4002.242016 |
| 4002.242062 | Kernel\_Read\|xilinx:adm-pcie-7v3:1ddr:3.0-0\|cnn\_GOOD | Read | OCL Region | 1 | 1 | 135016 | 135017 | 0.00333333 | 4002.242062 | 4002.242066 |
| 4002.242119 | Kernel\_Read\|xilinx:adm-pcie-7v3:1ddr:3.0-0\|cnn\_GOOD | Read | OCL Region | 1 | 1 | 135033 | 135034 | 0.00333333 | 4002.242119 | 4002.242122 |
| 4002.242169 | Kernel\_Read\|xilinx:adm-pcie-7v3:1ddr:3.0-0\|cnn\_GOOD | Read | OCL Region | 1 | 1 | 135048 | 135049 | 0.00333333 | 4002.242169 | 4002.242172 |
| 4002.242219 | Kernel\_Read\|xilinx:adm-pcie-7v3:1ddr:3.0-0\|cnn\_GOOD | Read | OCL Region | 1 | 1 | 135063 | 135064 | 0.00333333 | 4002.242219 | 4002.242222 |
| 4002.242269 | Kernel\_Read\|xilinx:adm-pcie-7v3:1ddr:3.0-0\|cnn\_GOOD | Read | OCL Region | 1 | 1 | 135078 | 135079 | 0.00333333 | 4002.242269 | 4002.242272 |
| 4002.242319 | Kernel\_Read\|xilinx:adm-pcie-7v3:1ddr:3.0-0\|cnn\_GOOD | Read | OCL Region | 1 | 1 | 135093 | 135094 | 0.00333333 | 4002.242319 | 4002.242322 |
| 4002.242369 | Kernel\_Read\|xilinx:adm-pcie-7v3:1ddr:3.0-0\|cnn\_GOOD | Read | OCL Region | 1 | 1 | 135108 | 135109 | 0.00333333 | 4002.242369 | 4002.242372 |
| 4002.242419 | Kernel\_Read\|xilinx:adm-pcie-7v3:1ddr:3.0-0\|cnn\_GOOD | Read | OCL Region | 1 | 1 | 135123 | 135124 | 0.00333333 | 4002.242419 | 4002.242422 |
| 4002.242469 | Kernel\_Read\|xilinx:adm-pcie-7v3:1ddr:3.0-0\|cnn\_GOOD | Read | OCL Region | 1 | 1 | 135138 | 135139 | 0.00333333 | 4002.242469 | 4002.242472 |
| 4002.242519 | Kernel\_Read\|xilinx:adm-pcie-7v3:1ddr:3.0-0\|cnn\_GOOD | Read | OCL Region | 1 | 1 | 135153 | 135154 | 0.00333333 | 4002.242519 | 4002.242522 |
| 4002.242569 | Kernel\_Read\|xilinx:adm-pcie-7v3:1ddr:3.0-0\|cnn\_GOOD | Read | OCL Region | 1 | 1 | 135168 | 135169 | 0.00333333 | 4002.242569 | 4002.242572 |
| 4002.242619 | Kernel\_Read\|xilinx:adm-pcie-7v3:1ddr:3.0-0\|cnn\_GOOD | Read | OCL Region | 1 | 1 | 135183 | 135184 | 0.00333333 | 4002.242619 | 4002.242622 |
| 4002.242669 | Kernel\_Read\|xilinx:adm-pcie-7v3:1ddr:3.0-0\|cnn\_GOOD | Read | OCL Region | 1 | 1 | 135198 | 135199 | 0.00333333 | 4002.242669 | 4002.242672 |
| 4002.242719 | Kernel\_Read\|xilinx:adm-pcie-7v3:1ddr:3.0-0\|cnn\_GOOD | Read | OCL Region | 1 | 1 | 135213 | 135214 | 0.00333333 | 4002.242719 | 4002.242722 |
| 4002.242769 | Kernel\_Read\|xilinx:adm-pcie-7v3:1ddr:3.0-0\|cnn\_GOOD | Read | OCL Region | 1 | 1 | 135228 | 135229 | 0.00333333 | 4002.242769 | 4002.242772 |
| 4002.242819 | Kernel\_Read\|xilinx:adm-pcie-7v3:1ddr:3.0-0\|cnn\_GOOD | Read | OCL Region | 1 | 1 | 135243 | 135244 | 0.00333333 | 4002.242819 | 4002.242822 |
| 4002.242869 | Kernel\_Read\|xilinx:adm-pcie-7v3:1ddr:3.0-0\|cnn\_GOOD | Read | OCL Region | 1 | 1 | 135258 | 135259 | 0.00333333 | 4002.242869 | 4002.242872 |
| 4002.242919 | Kernel\_Read\|xilinx:adm-pcie-7v3:1ddr:3.0-0\|cnn\_GOOD | Read | OCL Region | 1 | 1 | 135273 | 135274 | 0.00333333 | 4002.242919 | 4002.242922 |
| 4002.242969 | Kernel\_Read\|xilinx:adm-pcie-7v3:1ddr:3.0-0\|cnn\_GOOD | Read | OCL Region | 1 | 1 | 135288 | 135289 | 0.00333333 | 4002.242969 | 4002.242972 |
| 4002.243019 | Kernel\_Read\|xilinx:adm-pcie-7v3:1ddr:3.0-0\|cnn\_GOOD | Read | OCL Region | 1 | 1 | 135303 | 135304 | 0.00333333 | 4002.243019 | 4002.243022 |
| 4002.243069 | Kernel\_Read\|xilinx:adm-pcie-7v3:1ddr:3.0-0\|cnn\_GOOD | Read | OCL Region | 1 | 1 | 135318 | 135319 | 0.00333333 | 4002.243069 | 4002.243072 |
| 4002.243129 | Kernel\_Read\|xilinx:adm-pcie-7v3:1ddr:3.0-0\|cnn\_GOOD | Read | OCL Region | 1 | 1 | 135336 | 135337 | 0.00333333 | 4002.243129 | 4002.243132 |
| 4002.243179 | Kernel\_Read\|xilinx:adm-pcie-7v3:1ddr:3.0-0\|cnn\_GOOD | Read | OCL Region | 1 | 1 | 135351 | 135352 | 0.00333333 | 4002.243179 | 4002.243182 |
| 4002.243232 | Kernel\_Read\|xilinx:adm-pcie-7v3:1ddr:3.0-0\|cnn\_GOOD | Read | OCL Region | 1 | 1 | 135367 | 135368 | 0.00333333 | 4002.243232 | 4002.243236 |
| 4002.243279 | Kernel\_Read\|xilinx:adm-pcie-7v3:1ddr:3.0-0\|cnn\_GOOD | Read | OCL Region | 1 | 1 | 135381 | 135382 | 0.00333333 | 4002.243279 | 4002.243282 |
| 4002.243329 | Kernel\_Read\|xilinx:adm-pcie-7v3:1ddr:3.0-0\|cnn\_GOOD | Read | OCL Region | 1 | 1 | 135396 | 135397 | 0.00333333 | 4002.243329 | 4002.243332 |
| 4002.243379 | Kernel\_Read\|xilinx:adm-pcie-7v3:1ddr:3.0-0\|cnn\_GOOD | Read | OCL Region | 1 | 1 | 135411 | 135412 | 0.00333333 | 4002.243379 | 4002.243382 |
| 4002.243432 | Kernel\_Read\|xilinx:adm-pcie-7v3:1ddr:3.0-0\|cnn\_GOOD | Read | OCL Region | 1 | 1 | 135427 | 135428 | 0.00333333 | 4002.243432 | 4002.243436 |
| 4002.243479 | Kernel\_Read\|xilinx:adm-pcie-7v3:1ddr:3.0-0\|cnn\_GOOD | Read | OCL Region | 1 | 1 | 135441 | 135442 | 0.00333333 | 4002.243479 | 4002.243482 |
| 4002.243529 | Kernel\_Read\|xilinx:adm-pcie-7v3:1ddr:3.0-0\|cnn\_GOOD | Read | OCL Region | 1 | 1 | 135456 | 135457 | 0.00333333 | 4002.243529 | 4002.243532 |
| 4002.243579 | Kernel\_Read\|xilinx:adm-pcie-7v3:1ddr:3.0-0\|cnn\_GOOD | Read | OCL Region | 1 | 1 | 135471 | 135472 | 0.00333333 | 4002.243579 | 4002.243582 |
| 4002.243632 | Kernel\_Read\|xilinx:adm-pcie-7v3:1ddr:3.0-0\|cnn\_GOOD | Read | OCL Region | 1 | 1 | 135487 | 135488 | 0.00333333 | 4002.243632 | 4002.243636 |
| 4002.243679 | Kernel\_Read\|xilinx:adm-pcie-7v3:1ddr:3.0-0\|cnn\_GOOD | Read | OCL Region | 1 | 1 | 135501 | 135502 | 0.00333333 | 4002.243679 | 4002.243682 |
| 4002.243732 | Kernel\_Read\|xilinx:adm-pcie-7v3:1ddr:3.0-0\|cnn\_GOOD | Read | OCL Region | 1 | 1 | 135517 | 135518 | 0.00333333 | 4002.243732 | 4002.243736 |
| 4002.243779 | Kernel\_Read\|xilinx:adm-pcie-7v3:1ddr:3.0-0\|cnn\_GOOD | Read | OCL Region | 1 | 1 | 135531 | 135532 | 0.00333333 | 4002.243779 | 4002.243782 |
| 4002.243832 | Kernel\_Read\|xilinx:adm-pcie-7v3:1ddr:3.0-0\|cnn\_GOOD | Read | OCL Region | 1 | 1 | 135547 | 135548 | 0.00333333 | 4002.243832 | 4002.243836 |
| 4002.243879 | Kernel\_Read\|xilinx:adm-pcie-7v3:1ddr:3.0-0\|cnn\_GOOD | Read | OCL Region | 1 | 1 | 135561 | 135562 | 0.00333333 | 4002.243879 | 4002.243882 |
| 4002.243929 | Kernel\_Read\|xilinx:adm-pcie-7v3:1ddr:3.0-0\|cnn\_GOOD | Read | OCL Region | 1 | 1 | 135576 | 135577 | 0.00333333 | 4002.243929 | 4002.243932 |
| 4002.243979 | Kernel\_Read\|xilinx:adm-pcie-7v3:1ddr:3.0-0\|cnn\_GOOD | Read | OCL Region | 1 | 1 | 135591 | 135592 | 0.00333333 | 4002.243979 | 4002.243982 |
| 4002.244032 | Kernel\_Read\|xilinx:adm-pcie-7v3:1ddr:3.0-0\|cnn\_GOOD | Read | OCL Region | 1 | 1 | 135607 | 135608 | 0.00333333 | 4002.244032 | 4002.244036 |
| 4002.244092 | Kernel\_Read\|xilinx:adm-pcie-7v3:1ddr:3.0-0\|cnn\_GOOD | Read | OCL Region | 1 | 1 | 135625 | 135626 | 0.00333333 | 4002.244092 | 4002.244096 |
| 4002.244142 | Kernel\_Read\|xilinx:adm-pcie-7v3:1ddr:3.0-0\|cnn\_GOOD | Read | OCL Region | 1 | 1 | 135640 | 135641 | 0.00333333 | 4002.244142 | 4002.244146 |
| 4002.244412 | Kernel\_Read\|xilinx:adm-pcie-7v3:1ddr:3.0-0\|cnn\_GOOD | Read | OCL Region | 1 | 1 | 135721 | 135722 | 0.00333333 | 4002.244412 | 4002.244416 |
| 4002.244462 | Kernel\_Read\|xilinx:adm-pcie-7v3:1ddr:3.0-0\|cnn\_GOOD | Read | OCL Region | 1 | 1 | 135736 | 135737 | 0.00333333 | 4002.244462 | 4002.244466 |
| 4002.244512 | Kernel\_Read\|xilinx:adm-pcie-7v3:1ddr:3.0-0\|cnn\_GOOD | Read | OCL Region | 1 | 1 | 135751 | 135752 | 0.00333333 | 4002.244512 | 4002.244516 |
| 4002.244562 | Kernel\_Read\|xilinx:adm-pcie-7v3:1ddr:3.0-0\|cnn\_GOOD | Read | OCL Region | 1 | 1 | 135766 | 135767 | 0.00333333 | 4002.244562 | 4002.244566 |
| 4002.244616 | Kernel\_Read\|xilinx:adm-pcie-7v3:1ddr:3.0-0\|cnn\_GOOD | Read | OCL Region | 1 | 1 | 135782 | 135783 | 0.00333333 | 4002.244616 | 4002.244619 |
| 4002.244666 | Kernel\_Read\|xilinx:adm-pcie-7v3:1ddr:3.0-0\|cnn\_GOOD | Read | OCL Region | 1 | 1 | 135797 | 135798 | 0.00333333 | 4002.244666 | 4002.244669 |
| 4002.244716 | Kernel\_Read\|xilinx:adm-pcie-7v3:1ddr:3.0-0\|cnn\_GOOD | Read | OCL Region | 1 | 1 | 135812 | 135813 | 0.00333333 | 4002.244716 | 4002.244719 |
| 4002.244766 | Kernel\_Read\|xilinx:adm-pcie-7v3:1ddr:3.0-0\|cnn\_GOOD | Read | OCL Region | 1 | 1 | 135827 | 135828 | 0.00333333 | 4002.244766 | 4002.244769 |
| 4002.244816 | Kernel\_Read\|xilinx:adm-pcie-7v3:1ddr:3.0-0\|cnn\_GOOD | Read | OCL Region | 1 | 1 | 135842 | 135843 | 0.00333333 | 4002.244816 | 4002.244819 |
| 4002.244866 | Kernel\_Read\|xilinx:adm-pcie-7v3:1ddr:3.0-0\|cnn\_GOOD | Read | OCL Region | 1 | 1 | 135857 | 135858 | 0.00333333 | 4002.244866 | 4002.244869 |
| 4002.244916 | Kernel\_Read\|xilinx:adm-pcie-7v3:1ddr:3.0-0\|cnn\_GOOD | Read | OCL Region | 1 | 1 | 135872 | 135873 | 0.00333333 | 4002.244916 | 4002.244919 |
| 4002.244966 | Kernel\_Read\|xilinx:adm-pcie-7v3:1ddr:3.0-0\|cnn\_GOOD | Read | OCL Region | 1 | 1 | 135887 | 135888 | 0.00333333 | 4002.244966 | 4002.244969 |
| 4002.245016 | Kernel\_Read\|xilinx:adm-pcie-7v3:1ddr:3.0-0\|cnn\_GOOD | Read | OCL Region | 1 | 1 | 135902 | 135903 | 0.00333333 | 4002.245016 | 4002.245019 |
| 4002.245066 | Kernel\_Read\|xilinx:adm-pcie-7v3:1ddr:3.0-0\|cnn\_GOOD | Read | OCL Region | 1 | 1 | 135917 | 135918 | 0.00333333 | 4002.245066 | 4002.245069 |
| 4002.245126 | Kernel\_Read\|xilinx:adm-pcie-7v3:1ddr:3.0-0\|cnn\_GOOD | Read | OCL Region | 1 | 1 | 135935 | 135936 | 0.00333333 | 4002.245126 | 4002.245129 |
| 4002.245176 | Kernel\_Read\|xilinx:adm-pcie-7v3:1ddr:3.0-0\|cnn\_GOOD | Read | OCL Region | 1 | 1 | 135950 | 135951 | 0.00333333 | 4002.245176 | 4002.245179 |
| 4002.245226 | Kernel\_Read\|xilinx:adm-pcie-7v3:1ddr:3.0-0\|cnn\_GOOD | Read | OCL Region | 1 | 1 | 135965 | 135966 | 0.00333333 | 4002.245226 | 4002.245229 |
| 4002.245276 | Kernel\_Read\|xilinx:adm-pcie-7v3:1ddr:3.0-0\|cnn\_GOOD | Read | OCL Region | 1 | 1 | 135980 | 135981 | 0.00333333 | 4002.245276 | 4002.245279 |
| 4002.245326 | Kernel\_Read\|xilinx:adm-pcie-7v3:1ddr:3.0-0\|cnn\_GOOD | Read | OCL Region | 1 | 1 | 135995 | 135996 | 0.00333333 | 4002.245326 | 4002.245329 |
| 4002.245376 | Kernel\_Read\|xilinx:adm-pcie-7v3:1ddr:3.0-0\|cnn\_GOOD | Read | OCL Region | 1 | 1 | 136010 | 136011 | 0.00333333 | 4002.245376 | 4002.245379 |
| 4002.245426 | Kernel\_Read\|xilinx:adm-pcie-7v3:1ddr:3.0-0\|cnn\_GOOD | Read | OCL Region | 1 | 1 | 136025 | 136026 | 0.00333333 | 4002.245426 | 4002.245429 |
| 4002.245476 | Kernel\_Read\|xilinx:adm-pcie-7v3:1ddr:3.0-0\|cnn\_GOOD | Read | OCL Region | 1 | 1 | 136040 | 136041 | 0.00333333 | 4002.245476 | 4002.245479 |
| 4002.245526 | Kernel\_Read\|xilinx:adm-pcie-7v3:1ddr:3.0-0\|cnn\_GOOD | Read | OCL Region | 1 | 1 | 136055 | 136056 | 0.00333333 | 4002.245526 | 4002.245529 |
| 4002.245576 | Kernel\_Read\|xilinx:adm-pcie-7v3:1ddr:3.0-0\|cnn\_GOOD | Read | OCL Region | 1 | 1 | 136070 | 136071 | 0.00333333 | 4002.245576 | 4002.245579 |
| 4002.245626 | Kernel\_Read\|xilinx:adm-pcie-7v3:1ddr:3.0-0\|cnn\_GOOD | Read | OCL Region | 1 | 1 | 136085 | 136086 | 0.00333333 | 4002.245626 | 4002.245629 |
| 4002.245676 | Kernel\_Read\|xilinx:adm-pcie-7v3:1ddr:3.0-0\|cnn\_GOOD | Read | OCL Region | 1 | 1 | 136100 | 136101 | 0.00333333 | 4002.245676 | 4002.245679 |
| 4002.245726 | Kernel\_Read\|xilinx:adm-pcie-7v3:1ddr:3.0-0\|cnn\_GOOD | Read | OCL Region | 1 | 1 | 136115 | 136116 | 0.00333333 | 4002.245726 | 4002.245729 |
| 4002.245776 | Kernel\_Read\|xilinx:adm-pcie-7v3:1ddr:3.0-0\|cnn\_GOOD | Read | OCL Region | 1 | 1 | 136130 | 136131 | 0.00333333 | 4002.245776 | 4002.245779 |
| 4002.245826 | Kernel\_Read\|xilinx:adm-pcie-7v3:1ddr:3.0-0\|cnn\_GOOD | Read | OCL Region | 1 | 1 | 136145 | 136146 | 0.00333333 | 4002.245826 | 4002.245829 |
| 4002.245876 | Kernel\_Read\|xilinx:adm-pcie-7v3:1ddr:3.0-0\|cnn\_GOOD | Read | OCL Region | 1 | 1 | 136160 | 136161 | 0.00333333 | 4002.245876 | 4002.245879 |
| 4002.245926 | Kernel\_Read\|xilinx:adm-pcie-7v3:1ddr:3.0-0\|cnn\_GOOD | Read | OCL Region | 1 | 1 | 136175 | 136176 | 0.00333333 | 4002.245926 | 4002.245929 |
| 4002.245976 | Kernel\_Read\|xilinx:adm-pcie-7v3:1ddr:3.0-0\|cnn\_GOOD | Read | OCL Region | 1 | 1 | 136190 | 136191 | 0.00333333 | 4002.245976 | 4002.245979 |
| 4002.246026 | Kernel\_Read\|xilinx:adm-pcie-7v3:1ddr:3.0-0\|cnn\_GOOD | Read | OCL Region | 1 | 1 | 136205 | 136206 | 0.00333333 | 4002.246026 | 4002.246029 |
| 4002.246086 | Kernel\_Read\|xilinx:adm-pcie-7v3:1ddr:3.0-0\|cnn\_GOOD | Read | OCL Region | 1 | 1 | 136223 | 136224 | 0.00333333 | 4002.246086 | 4002.246089 |
| 4002.246136 | Kernel\_Read\|xilinx:adm-pcie-7v3:1ddr:3.0-0\|cnn\_GOOD | Read | OCL Region | 1 | 1 | 136238 | 136239 | 0.00333333 | 4002.246136 | 4002.246139 |
| 4002.246186 | Kernel\_Read\|xilinx:adm-pcie-7v3:1ddr:3.0-0\|cnn\_GOOD | Read | OCL Region | 1 | 1 | 136253 | 136254 | 0.00333333 | 4002.246186 | 4002.246189 |
| 4002.246236 | Kernel\_Read\|xilinx:adm-pcie-7v3:1ddr:3.0-0\|cnn\_GOOD | Read | OCL Region | 1 | 1 | 136268 | 136269 | 0.00333333 | 4002.246236 | 4002.246239 |
| 4002.246286 | Kernel\_Read\|xilinx:adm-pcie-7v3:1ddr:3.0-0\|cnn\_GOOD | Read | OCL Region | 1 | 1 | 136283 | 136284 | 0.00333333 | 4002.246286 | 4002.246289 |
| 4002.246336 | Kernel\_Read\|xilinx:adm-pcie-7v3:1ddr:3.0-0\|cnn\_GOOD | Read | OCL Region | 1 | 1 | 136298 | 136299 | 0.00333333 | 4002.246336 | 4002.246339 |
| 4002.246386 | Kernel\_Read\|xilinx:adm-pcie-7v3:1ddr:3.0-0\|cnn\_GOOD | Read | OCL Region | 1 | 1 | 136313 | 136314 | 0.00333333 | 4002.246386 | 4002.246389 |
| 4002.246436 | Kernel\_Read\|xilinx:adm-pcie-7v3:1ddr:3.0-0\|cnn\_GOOD | Read | OCL Region | 1 | 1 | 136328 | 136329 | 0.00333333 | 4002.246436 | 4002.246439 |
| 4002.246486 | Kernel\_Read\|xilinx:adm-pcie-7v3:1ddr:3.0-0\|cnn\_GOOD | Read | OCL Region | 1 | 1 | 136343 | 136344 | 0.00333333 | 4002.246486 | 4002.246489 |
| 4002.246536 | Kernel\_Read\|xilinx:adm-pcie-7v3:1ddr:3.0-0\|cnn\_GOOD | Read | OCL Region | 1 | 1 | 136358 | 136359 | 0.00333333 | 4002.246536 | 4002.246539 |
| 4002.246586 | Kernel\_Read\|xilinx:adm-pcie-7v3:1ddr:3.0-0\|cnn\_GOOD | Read | OCL Region | 1 | 1 | 136373 | 136374 | 0.00333333 | 4002.246586 | 4002.246589 |
| 4002.246636 | Kernel\_Read\|xilinx:adm-pcie-7v3:1ddr:3.0-0\|cnn\_GOOD | Read | OCL Region | 1 | 1 | 136388 | 136389 | 0.00333333 | 4002.246636 | 4002.246639 |
| 4002.246686 | Kernel\_Read\|xilinx:adm-pcie-7v3:1ddr:3.0-0\|cnn\_GOOD | Read | OCL Region | 1 | 1 | 136403 | 136404 | 0.00333333 | 4002.246686 | 4002.246689 |
| 4002.246736 | Kernel\_Read\|xilinx:adm-pcie-7v3:1ddr:3.0-0\|cnn\_GOOD | Read | OCL Region | 1 | 1 | 136418 | 136419 | 0.00333333 | 4002.246736 | 4002.246739 |
| 4002.246786 | Kernel\_Read\|xilinx:adm-pcie-7v3:1ddr:3.0-0\|cnn\_GOOD | Read | OCL Region | 1 | 1 | 136433 | 136434 | 0.00333333 | 4002.246786 | 4002.246789 |
| 4002.246836 | Kernel\_Read\|xilinx:adm-pcie-7v3:1ddr:3.0-0\|cnn\_GOOD | Read | OCL Region | 1 | 1 | 136448 | 136449 | 0.00333333 | 4002.246836 | 4002.246839 |
| 4002.246886 | Kernel\_Read\|xilinx:adm-pcie-7v3:1ddr:3.0-0\|cnn\_GOOD | Read | OCL Region | 1 | 1 | 136463 | 136464 | 0.00333333 | 4002.246886 | 4002.246889 |
| 4002.246936 | Kernel\_Read\|xilinx:adm-pcie-7v3:1ddr:3.0-0\|cnn\_GOOD | Read | OCL Region | 1 | 1 | 136478 | 136479 | 0.00333333 | 4002.246936 | 4002.246939 |
| 4002.246986 | Kernel\_Read\|xilinx:adm-pcie-7v3:1ddr:3.0-0\|cnn\_GOOD | Read | OCL Region | 1 | 1 | 136493 | 136494 | 0.00333333 | 4002.246986 | 4002.246989 |
| 4002.247036 | Kernel\_Read\|xilinx:adm-pcie-7v3:1ddr:3.0-0\|cnn\_GOOD | Read | OCL Region | 1 | 1 | 136508 | 136509 | 0.00333333 | 4002.247036 | 4002.247039 |
| 4002.247096 | Kernel\_Read\|xilinx:adm-pcie-7v3:1ddr:3.0-0\|cnn\_GOOD | Read | OCL Region | 1 | 1 | 136526 | 136527 | 0.00333333 | 4002.247096 | 4002.247099 |
| 4002.247146 | Kernel\_Read\|xilinx:adm-pcie-7v3:1ddr:3.0-0\|cnn\_GOOD | Read | OCL Region | 1 | 1 | 136541 | 136542 | 0.00333333 | 4002.247146 | 4002.247149 |
| 4002.247196 | Kernel\_Read\|xilinx:adm-pcie-7v3:1ddr:3.0-0\|cnn\_GOOD | Read | OCL Region | 1 | 1 | 136556 | 136557 | 0.00333333 | 4002.247196 | 4002.247199 |
| 4002.247246 | Kernel\_Read\|xilinx:adm-pcie-7v3:1ddr:3.0-0\|cnn\_GOOD | Read | OCL Region | 1 | 1 | 136571 | 136572 | 0.00333333 | 4002.247246 | 4002.247249 |
| 4002.247296 | Kernel\_Read\|xilinx:adm-pcie-7v3:1ddr:3.0-0\|cnn\_GOOD | Read | OCL Region | 1 | 1 | 136586 | 136587 | 0.00333333 | 4002.247296 | 4002.247299 |
| 4002.247346 | Kernel\_Read\|xilinx:adm-pcie-7v3:1ddr:3.0-0\|cnn\_GOOD | Read | OCL Region | 1 | 1 | 136601 | 136602 | 0.00333333 | 4002.247346 | 4002.247349 |
| 4002.247396 | Kernel\_Read\|xilinx:adm-pcie-7v3:1ddr:3.0-0\|cnn\_GOOD | Read | OCL Region | 1 | 1 | 136616 | 136617 | 0.00333333 | 4002.247396 | 4002.247399 |
| 4002.247446 | Kernel\_Read\|xilinx:adm-pcie-7v3:1ddr:3.0-0\|cnn\_GOOD | Read | OCL Region | 1 | 1 | 136631 | 136632 | 0.00333333 | 4002.247446 | 4002.247449 |
| 4002.247496 | Kernel\_Read\|xilinx:adm-pcie-7v3:1ddr:3.0-0\|cnn\_GOOD | Read | OCL Region | 1 | 1 | 136646 | 136647 | 0.00333333 | 4002.247496 | 4002.247499 |
| 4002.247546 | Kernel\_Read\|xilinx:adm-pcie-7v3:1ddr:3.0-0\|cnn\_GOOD | Read | OCL Region | 1 | 1 | 136661 | 136662 | 0.00333333 | 4002.247546 | 4002.247549 |
| 4002.247596 | Kernel\_Read\|xilinx:adm-pcie-7v3:1ddr:3.0-0\|cnn\_GOOD | Read | OCL Region | 1 | 1 | 136676 | 136677 | 0.00333333 | 4002.247596 | 4002.247599 |
| 4002.247646 | Kernel\_Read\|xilinx:adm-pcie-7v3:1ddr:3.0-0\|cnn\_GOOD | Read | OCL Region | 1 | 1 | 136691 | 136692 | 0.00333333 | 4002.247646 | 4002.247649 |
| 4002.247696 | Kernel\_Read\|xilinx:adm-pcie-7v3:1ddr:3.0-0\|cnn\_GOOD | Read | OCL Region | 1 | 1 | 136706 | 136707 | 0.00333333 | 4002.247696 | 4002.247699 |
| 4002.247746 | Kernel\_Read\|xilinx:adm-pcie-7v3:1ddr:3.0-0\|cnn\_GOOD | Read | OCL Region | 1 | 1 | 136721 | 136722 | 0.00333333 | 4002.247746 | 4002.247749 |
| 4002.247796 | Kernel\_Read\|xilinx:adm-pcie-7v3:1ddr:3.0-0\|cnn\_GOOD | Read | OCL Region | 1 | 1 | 136736 | 136737 | 0.00333333 | 4002.247796 | 4002.247799 |
| 4002.247846 | Kernel\_Read\|xilinx:adm-pcie-7v3:1ddr:3.0-0\|cnn\_GOOD | Read | OCL Region | 1 | 1 | 136751 | 136752 | 0.00333333 | 4002.247846 | 4002.247849 |
| 4002.247896 | Kernel\_Read\|xilinx:adm-pcie-7v3:1ddr:3.0-0\|cnn\_GOOD | Read | OCL Region | 1 | 1 | 136766 | 136767 | 0.00333333 | 4002.247896 | 4002.247899 |
| 4002.247946 | Kernel\_Read\|xilinx:adm-pcie-7v3:1ddr:3.0-0\|cnn\_GOOD | Read | OCL Region | 1 | 1 | 136781 | 136782 | 0.00333333 | 4002.247946 | 4002.247949 |
| 4002.247996 | Kernel\_Read\|xilinx:adm-pcie-7v3:1ddr:3.0-0\|cnn\_GOOD | Read | OCL Region | 1 | 1 | 136796 | 136797 | 0.00333333 | 4002.247996 | 4002.247999 |
| 4002.248052 | Kernel\_Read\|xilinx:adm-pcie-7v3:1ddr:3.0-0\|cnn\_GOOD | Read | OCL Region | 1 | 1 | 136813 | 136814 | 0.00333333 | 4002.248052 | 4002.248056 |
| 4002.248109 | Kernel\_Read\|xilinx:adm-pcie-7v3:1ddr:3.0-0\|cnn\_GOOD | Read | OCL Region | 1 | 1 | 136830 | 136831 | 0.00333333 | 4002.248109 | 4002.248112 |
| 4002.248159 | Kernel\_Read\|xilinx:adm-pcie-7v3:1ddr:3.0-0\|cnn\_GOOD | Read | OCL Region | 1 | 1 | 136845 | 136846 | 0.00333333 | 4002.248159 | 4002.248162 |
| 4002.248202 | Kernel\_Read\|xilinx:adm-pcie-7v3:1ddr:3.0-0\|cnn\_GOOD | Read | OCL Region | 1 | 1 | 136858 | 136859 | 0.00333333 | 4002.248202 | 4002.248206 |
| 4002.248252 | Kernel\_Read\|xilinx:adm-pcie-7v3:1ddr:3.0-0\|cnn\_GOOD | Read | OCL Region | 1 | 1 | 136873 | 136874 | 0.00333333 | 4002.248252 | 4002.248256 |
| 4002.248309 | Kernel\_Read\|xilinx:adm-pcie-7v3:1ddr:3.0-0\|cnn\_GOOD | Read | OCL Region | 1 | 1 | 136890 | 136891 | 0.00333333 | 4002.248309 | 4002.248312 |
| 4002.248359 | Kernel\_Read\|xilinx:adm-pcie-7v3:1ddr:3.0-0\|cnn\_GOOD | Read | OCL Region | 1 | 1 | 136905 | 136906 | 0.00333333 | 4002.248359 | 4002.248362 |
| 4002.248409 | Kernel\_Read\|xilinx:adm-pcie-7v3:1ddr:3.0-0\|cnn\_GOOD | Read | OCL Region | 1 | 1 | 136920 | 136921 | 0.00333333 | 4002.248409 | 4002.248412 |
| 4002.248452 | Kernel\_Read\|xilinx:adm-pcie-7v3:1ddr:3.0-0\|cnn\_GOOD | Read | OCL Region | 1 | 1 | 136933 | 136934 | 0.00333333 | 4002.248452 | 4002.248456 |
| 4002.248509 | Kernel\_Read\|xilinx:adm-pcie-7v3:1ddr:3.0-0\|cnn\_GOOD | Read | OCL Region | 1 | 1 | 136950 | 136951 | 0.00333333 | 4002.248509 | 4002.248512 |
| 4002.248559 | Kernel\_Read\|xilinx:adm-pcie-7v3:1ddr:3.0-0\|cnn\_GOOD | Read | OCL Region | 1 | 1 | 136965 | 136966 | 0.00333333 | 4002.248559 | 4002.248562 |
| 4002.248602 | Kernel\_Read\|xilinx:adm-pcie-7v3:1ddr:3.0-0\|cnn\_GOOD | Read | OCL Region | 1 | 1 | 136978 | 136979 | 0.00333333 | 4002.248602 | 4002.248606 |
| 4002.248652 | Kernel\_Read\|xilinx:adm-pcie-7v3:1ddr:3.0-0\|cnn\_GOOD | Read | OCL Region | 1 | 1 | 136993 | 136994 | 0.00333333 | 4002.248652 | 4002.248656 |
| 4002.248709 | Kernel\_Read\|xilinx:adm-pcie-7v3:1ddr:3.0-0\|cnn\_GOOD | Read | OCL Region | 1 | 1 | 137010 | 137011 | 0.00333333 | 4002.248709 | 4002.248712 |
| 4002.248759 | Kernel\_Read\|xilinx:adm-pcie-7v3:1ddr:3.0-0\|cnn\_GOOD | Read | OCL Region | 1 | 1 | 137025 | 137026 | 0.00333333 | 4002.248759 | 4002.248762 |
| 4002.248809 | Kernel\_Read\|xilinx:adm-pcie-7v3:1ddr:3.0-0\|cnn\_GOOD | Read | OCL Region | 1 | 1 | 137040 | 137041 | 0.00333333 | 4002.248809 | 4002.248812 |
| 4002.248852 | Kernel\_Read\|xilinx:adm-pcie-7v3:1ddr:3.0-0\|cnn\_GOOD | Read | OCL Region | 1 | 1 | 137053 | 137054 | 0.00333333 | 4002.248852 | 4002.248856 |
| 4002.248909 | Kernel\_Read\|xilinx:adm-pcie-7v3:1ddr:3.0-0\|cnn\_GOOD | Read | OCL Region | 1 | 1 | 137070 | 137071 | 0.00333333 | 4002.248909 | 4002.248912 |
| 4002.248959 | Kernel\_Read\|xilinx:adm-pcie-7v3:1ddr:3.0-0\|cnn\_GOOD | Read | OCL Region | 1 | 1 | 137085 | 137086 | 0.00333333 | 4002.248959 | 4002.248962 |
| 4002.249002 | Kernel\_Read\|xilinx:adm-pcie-7v3:1ddr:3.0-0\|cnn\_GOOD | Read | OCL Region | 1 | 1 | 137098 | 137099 | 0.00333333 | 4002.249002 | 4002.249006 |
| 4002.249066 | Kernel\_Read\|xilinx:adm-pcie-7v3:1ddr:3.0-0\|cnn\_GOOD | Read | OCL Region | 1 | 1 | 137117 | 137118 | 0.00333333 | 4002.249066 | 4002.249069 |
| 4002.249116 | Kernel\_Read\|xilinx:adm-pcie-7v3:1ddr:3.0-0\|cnn\_GOOD | Read | OCL Region | 1 | 1 | 137132 | 137133 | 0.00333333 | 4002.249116 | 4002.249119 |
| 4002.249166 | Kernel\_Read\|xilinx:adm-pcie-7v3:1ddr:3.0-0\|cnn\_GOOD | Read | OCL Region | 1 | 1 | 137147 | 137148 | 0.00333333 | 4002.249166 | 4002.249169 |
| 4002.249216 | Kernel\_Read\|xilinx:adm-pcie-7v3:1ddr:3.0-0\|cnn\_GOOD | Read | OCL Region | 1 | 1 | 137162 | 137163 | 0.00333333 | 4002.249216 | 4002.249219 |
| 4002.249266 | Kernel\_Read\|xilinx:adm-pcie-7v3:1ddr:3.0-0\|cnn\_GOOD | Read | OCL Region | 1 | 1 | 137177 | 137178 | 0.00333333 | 4002.249266 | 4002.249269 |
| 4002.249316 | Kernel\_Read\|xilinx:adm-pcie-7v3:1ddr:3.0-0\|cnn\_GOOD | Read | OCL Region | 1 | 1 | 137192 | 137193 | 0.00333333 | 4002.249316 | 4002.249319 |
| 4002.249366 | Kernel\_Read\|xilinx:adm-pcie-7v3:1ddr:3.0-0\|cnn\_GOOD | Read | OCL Region | 1 | 1 | 137207 | 137208 | 0.00333333 | 4002.249366 | 4002.249369 |
| 4002.249416 | Kernel\_Read\|xilinx:adm-pcie-7v3:1ddr:3.0-0\|cnn\_GOOD | Read | OCL Region | 1 | 1 | 137222 | 137223 | 0.00333333 | 4002.249416 | 4002.249419 |
| 4002.249466 | Kernel\_Read\|xilinx:adm-pcie-7v3:1ddr:3.0-0\|cnn\_GOOD | Read | OCL Region | 1 | 1 | 137237 | 137238 | 0.00333333 | 4002.249466 | 4002.249469 |
| 4002.249516 | Kernel\_Read\|xilinx:adm-pcie-7v3:1ddr:3.0-0\|cnn\_GOOD | Read | OCL Region | 1 | 1 | 137252 | 137253 | 0.00333333 | 4002.249516 | 4002.249519 |
| 4002.249566 | Kernel\_Read\|xilinx:adm-pcie-7v3:1ddr:3.0-0\|cnn\_GOOD | Read | OCL Region | 1 | 1 | 137267 | 137268 | 0.00333333 | 4002.249566 | 4002.249569 |
| 4002.249616 | Kernel\_Read\|xilinx:adm-pcie-7v3:1ddr:3.0-0\|cnn\_GOOD | Read | OCL Region | 1 | 1 | 137282 | 137283 | 0.00333333 | 4002.249616 | 4002.249619 |
| 4002.249666 | Kernel\_Read\|xilinx:adm-pcie-7v3:1ddr:3.0-0\|cnn\_GOOD | Read | OCL Region | 1 | 1 | 137297 | 137298 | 0.00333333 | 4002.249666 | 4002.249669 |
| 4002.249716 | Kernel\_Read\|xilinx:adm-pcie-7v3:1ddr:3.0-0\|cnn\_GOOD | Read | OCL Region | 1 | 1 | 137312 | 137313 | 0.00333333 | 4002.249716 | 4002.249719 |
| 4002.249766 | Kernel\_Read\|xilinx:adm-pcie-7v3:1ddr:3.0-0\|cnn\_GOOD | Read | OCL Region | 1 | 1 | 137327 | 137328 | 0.00333333 | 4002.249766 | 4002.249769 |
| 4002.249816 | Kernel\_Read\|xilinx:adm-pcie-7v3:1ddr:3.0-0\|cnn\_GOOD | Read | OCL Region | 1 | 1 | 137342 | 137343 | 0.00333333 | 4002.249816 | 4002.249819 |
| 4002.249866 | Kernel\_Read\|xilinx:adm-pcie-7v3:1ddr:3.0-0\|cnn\_GOOD | Read | OCL Region | 1 | 1 | 137357 | 137358 | 0.00333333 | 4002.249866 | 4002.249869 |
| 4002.249916 | Kernel\_Read\|xilinx:adm-pcie-7v3:1ddr:3.0-0\|cnn\_GOOD | Read | OCL Region | 1 | 1 | 137372 | 137373 | 0.00333333 | 4002.249916 | 4002.249919 |
| 4002.249966 | Kernel\_Read\|xilinx:adm-pcie-7v3:1ddr:3.0-0\|cnn\_GOOD | Read | OCL Region | 1 | 1 | 137387 | 137388 | 0.00333333 | 4002.249966 | 4002.249969 |
| 4002.250016 | Kernel\_Read\|xilinx:adm-pcie-7v3:1ddr:3.0-0\|cnn\_GOOD | Read | OCL Region | 1 | 1 | 137402 | 137403 | 0.00333333 | 4002.250016 | 4002.250019 |
| 4002.250076 | Kernel\_Read\|xilinx:adm-pcie-7v3:1ddr:3.0-0\|cnn\_GOOD | Read | OCL Region | 1 | 1 | 137420 | 137421 | 0.00333333 | 4002.250076 | 4002.250079 |
| 4002.250126 | Kernel\_Read\|xilinx:adm-pcie-7v3:1ddr:3.0-0\|cnn\_GOOD | Read | OCL Region | 1 | 1 | 137435 | 137436 | 0.00333333 | 4002.250126 | 4002.250129 |
| 4002.250176 | Kernel\_Read\|xilinx:adm-pcie-7v3:1ddr:3.0-0\|cnn\_GOOD | Read | OCL Region | 1 | 1 | 137450 | 137451 | 0.00333333 | 4002.250176 | 4002.250179 |
| 4002.250226 | Kernel\_Read\|xilinx:adm-pcie-7v3:1ddr:3.0-0\|cnn\_GOOD | Read | OCL Region | 1 | 1 | 137465 | 137466 | 0.00333333 | 4002.250226 | 4002.250229 |
| 4002.250276 | Kernel\_Read\|xilinx:adm-pcie-7v3:1ddr:3.0-0\|cnn\_GOOD | Read | OCL Region | 1 | 1 | 137480 | 137481 | 0.00333333 | 4002.250276 | 4002.250279 |
| 4002.250326 | Kernel\_Read\|xilinx:adm-pcie-7v3:1ddr:3.0-0\|cnn\_GOOD | Read | OCL Region | 1 | 1 | 137495 | 137496 | 0.00333333 | 4002.250326 | 4002.250329 |
| 4002.250376 | Kernel\_Read\|xilinx:adm-pcie-7v3:1ddr:3.0-0\|cnn\_GOOD | Read | OCL Region | 1 | 1 | 137510 | 137511 | 0.00333333 | 4002.250376 | 4002.250379 |
| 4002.250426 | Kernel\_Read\|xilinx:adm-pcie-7v3:1ddr:3.0-0\|cnn\_GOOD | Read | OCL Region | 1 | 1 | 137525 | 137526 | 0.00333333 | 4002.250426 | 4002.250429 |
| 4002.250476 | Kernel\_Read\|xilinx:adm-pcie-7v3:1ddr:3.0-0\|cnn\_GOOD | Read | OCL Region | 1 | 1 | 137540 | 137541 | 0.00333333 | 4002.250476 | 4002.250479 |
| 4002.250746 | Kernel\_Read\|xilinx:adm-pcie-7v3:1ddr:3.0-0\|cnn\_GOOD | Read | OCL Region | 1 | 1 | 137621 | 137622 | 0.00333333 | 4002.250746 | 4002.250749 |
| 4002.250796 | Kernel\_Read\|xilinx:adm-pcie-7v3:1ddr:3.0-0\|cnn\_GOOD | Read | OCL Region | 1 | 1 | 137636 | 137637 | 0.00333333 | 4002.250796 | 4002.250799 |
| 4002.250846 | Kernel\_Read\|xilinx:adm-pcie-7v3:1ddr:3.0-0\|cnn\_GOOD | Read | OCL Region | 1 | 1 | 137651 | 137652 | 0.00333333 | 4002.250846 | 4002.250849 |
| 4002.250896 | Kernel\_Read\|xilinx:adm-pcie-7v3:1ddr:3.0-0\|cnn\_GOOD | Read | OCL Region | 1 | 1 | 137666 | 137667 | 0.00333333 | 4002.250896 | 4002.250899 |
| 4002.250949 | Kernel\_Read\|xilinx:adm-pcie-7v3:1ddr:3.0-0\|cnn\_GOOD | Read | OCL Region | 1 | 1 | 137682 | 137683 | 0.00333333 | 4002.250949 | 4002.250952 |
| 4002.250999 | Kernel\_Read\|xilinx:adm-pcie-7v3:1ddr:3.0-0\|cnn\_GOOD | Read | OCL Region | 1 | 1 | 137697 | 137698 | 0.00333333 | 4002.250999 | 4002.251002 |
| 4002.251062 | Kernel\_Read\|xilinx:adm-pcie-7v3:1ddr:3.0-0\|cnn\_GOOD | Read | OCL Region | 1 | 1 | 137716 | 137717 | 0.00333333 | 4002.251062 | 4002.251066 |
| 4002.251112 | Kernel\_Read\|xilinx:adm-pcie-7v3:1ddr:3.0-0\|cnn\_GOOD | Read | OCL Region | 1 | 1 | 137731 | 137732 | 0.00333333 | 4002.251112 | 4002.251116 |
| 4002.251162 | Kernel\_Read\|xilinx:adm-pcie-7v3:1ddr:3.0-0\|cnn\_GOOD | Read | OCL Region | 1 | 1 | 137746 | 137747 | 0.00333333 | 4002.251162 | 4002.251166 |
| 4002.251212 | Kernel\_Read\|xilinx:adm-pcie-7v3:1ddr:3.0-0\|cnn\_GOOD | Read | OCL Region | 1 | 1 | 137761 | 137762 | 0.00333333 | 4002.251212 | 4002.251216 |
| 4002.251262 | Kernel\_Read\|xilinx:adm-pcie-7v3:1ddr:3.0-0\|cnn\_GOOD | Read | OCL Region | 1 | 1 | 137776 | 137777 | 0.00333333 | 4002.251262 | 4002.251266 |
| 4002.251312 | Kernel\_Read\|xilinx:adm-pcie-7v3:1ddr:3.0-0\|cnn\_GOOD | Read | OCL Region | 1 | 1 | 137791 | 137792 | 0.00333333 | 4002.251312 | 4002.251316 |
| 4002.251362 | Kernel\_Read\|xilinx:adm-pcie-7v3:1ddr:3.0-0\|cnn\_GOOD | Read | OCL Region | 1 | 1 | 137806 | 137807 | 0.00333333 | 4002.251362 | 4002.251366 |
| 4002.251412 | Kernel\_Read\|xilinx:adm-pcie-7v3:1ddr:3.0-0\|cnn\_GOOD | Read | OCL Region | 1 | 1 | 137821 | 137822 | 0.00333333 | 4002.251412 | 4002.251416 |
| 4002.251462 | Kernel\_Read\|xilinx:adm-pcie-7v3:1ddr:3.0-0\|cnn\_GOOD | Read | OCL Region | 1 | 1 | 137836 | 137837 | 0.00333333 | 4002.251462 | 4002.251466 |
| 4002.251512 | Kernel\_Read\|xilinx:adm-pcie-7v3:1ddr:3.0-0\|cnn\_GOOD | Read | OCL Region | 1 | 1 | 137851 | 137852 | 0.00333333 | 4002.251512 | 4002.251516 |
| 4002.251562 | Kernel\_Read\|xilinx:adm-pcie-7v3:1ddr:3.0-0\|cnn\_GOOD | Read | OCL Region | 1 | 1 | 137866 | 137867 | 0.00333333 | 4002.251562 | 4002.251566 |
| 4002.251612 | Kernel\_Read\|xilinx:adm-pcie-7v3:1ddr:3.0-0\|cnn\_GOOD | Read | OCL Region | 1 | 1 | 137881 | 137882 | 0.00333333 | 4002.251612 | 4002.251616 |
| 4002.251662 | Kernel\_Read\|xilinx:adm-pcie-7v3:1ddr:3.0-0\|cnn\_GOOD | Read | OCL Region | 1 | 1 | 137896 | 137897 | 0.00333333 | 4002.251662 | 4002.251666 |
| 4002.251712 | Kernel\_Read\|xilinx:adm-pcie-7v3:1ddr:3.0-0\|cnn\_GOOD | Read | OCL Region | 1 | 1 | 137911 | 137912 | 0.00333333 | 4002.251712 | 4002.251716 |
| 4002.251762 | Kernel\_Read\|xilinx:adm-pcie-7v3:1ddr:3.0-0\|cnn\_GOOD | Read | OCL Region | 1 | 1 | 137926 | 137927 | 0.00333333 | 4002.251762 | 4002.251766 |
| 4002.251812 | Kernel\_Read\|xilinx:adm-pcie-7v3:1ddr:3.0-0\|cnn\_GOOD | Read | OCL Region | 1 | 1 | 137941 | 137942 | 0.00333333 | 4002.251812 | 4002.251816 |
| 4002.251862 | Kernel\_Read\|xilinx:adm-pcie-7v3:1ddr:3.0-0\|cnn\_GOOD | Read | OCL Region | 1 | 1 | 137956 | 137957 | 0.00333333 | 4002.251862 | 4002.251866 |
| 4002.251912 | Kernel\_Read\|xilinx:adm-pcie-7v3:1ddr:3.0-0\|cnn\_GOOD | Read | OCL Region | 1 | 1 | 137971 | 137972 | 0.00333333 | 4002.251912 | 4002.251916 |
| 4002.251962 | Kernel\_Read\|xilinx:adm-pcie-7v3:1ddr:3.0-0\|cnn\_GOOD | Read | OCL Region | 1 | 1 | 137986 | 137987 | 0.00333333 | 4002.251962 | 4002.251966 |
| 4002.252019 | Kernel\_Read\|xilinx:adm-pcie-7v3:1ddr:3.0-0\|cnn\_GOOD | Read | OCL Region | 1 | 1 | 138003 | 138004 | 0.00333333 | 4002.252019 | 4002.252022 |
| 4002.252069 | Kernel\_Read\|xilinx:adm-pcie-7v3:1ddr:3.0-0\|cnn\_GOOD | Read | OCL Region | 1 | 1 | 138018 | 138019 | 0.00333333 | 4002.252069 | 4002.252072 |
| 4002.252119 | Kernel\_Read\|xilinx:adm-pcie-7v3:1ddr:3.0-0\|cnn\_GOOD | Read | OCL Region | 1 | 1 | 138033 | 138034 | 0.00333333 | 4002.252119 | 4002.252122 |
| 4002.252169 | Kernel\_Read\|xilinx:adm-pcie-7v3:1ddr:3.0-0\|cnn\_GOOD | Read | OCL Region | 1 | 1 | 138048 | 138049 | 0.00333333 | 4002.252169 | 4002.252172 |
| 4002.252219 | Kernel\_Read\|xilinx:adm-pcie-7v3:1ddr:3.0-0\|cnn\_GOOD | Read | OCL Region | 1 | 1 | 138063 | 138064 | 0.00333333 | 4002.252219 | 4002.252222 |
| 4002.252269 | Kernel\_Read\|xilinx:adm-pcie-7v3:1ddr:3.0-0\|cnn\_GOOD | Read | OCL Region | 1 | 1 | 138078 | 138079 | 0.00333333 | 4002.252269 | 4002.252272 |
| 4002.252319 | Kernel\_Read\|xilinx:adm-pcie-7v3:1ddr:3.0-0\|cnn\_GOOD | Read | OCL Region | 1 | 1 | 138093 | 138094 | 0.00333333 | 4002.252319 | 4002.252322 |
| 4002.252369 | Kernel\_Read\|xilinx:adm-pcie-7v3:1ddr:3.0-0\|cnn\_GOOD | Read | OCL Region | 1 | 1 | 138108 | 138109 | 0.00333333 | 4002.252369 | 4002.252372 |
| 4002.252419 | Kernel\_Read\|xilinx:adm-pcie-7v3:1ddr:3.0-0\|cnn\_GOOD | Read | OCL Region | 1 | 1 | 138123 | 138124 | 0.00333333 | 4002.252419 | 4002.252422 |
| 4002.252469 | Kernel\_Read\|xilinx:adm-pcie-7v3:1ddr:3.0-0\|cnn\_GOOD | Read | OCL Region | 1 | 1 | 138138 | 138139 | 0.00333333 | 4002.252469 | 4002.252472 |
| 4002.252519 | Kernel\_Read\|xilinx:adm-pcie-7v3:1ddr:3.0-0\|cnn\_GOOD | Read | OCL Region | 1 | 1 | 138153 | 138154 | 0.00333333 | 4002.252519 | 4002.252522 |
| 4002.252569 | Kernel\_Read\|xilinx:adm-pcie-7v3:1ddr:3.0-0\|cnn\_GOOD | Read | OCL Region | 1 | 1 | 138168 | 138169 | 0.00333333 | 4002.252569 | 4002.252572 |
| 4002.252619 | Kernel\_Read\|xilinx:adm-pcie-7v3:1ddr:3.0-0\|cnn\_GOOD | Read | OCL Region | 1 | 1 | 138183 | 138184 | 0.00333333 | 4002.252619 | 4002.252622 |
| 4002.252669 | Kernel\_Read\|xilinx:adm-pcie-7v3:1ddr:3.0-0\|cnn\_GOOD | Read | OCL Region | 1 | 1 | 138198 | 138199 | 0.00333333 | 4002.252669 | 4002.252672 |
| 4002.252719 | Kernel\_Read\|xilinx:adm-pcie-7v3:1ddr:3.0-0\|cnn\_GOOD | Read | OCL Region | 1 | 1 | 138213 | 138214 | 0.00333333 | 4002.252719 | 4002.252722 |
| 4002.252769 | Kernel\_Read\|xilinx:adm-pcie-7v3:1ddr:3.0-0\|cnn\_GOOD | Read | OCL Region | 1 | 1 | 138228 | 138229 | 0.00333333 | 4002.252769 | 4002.252772 |
| 4002.252819 | Kernel\_Read\|xilinx:adm-pcie-7v3:1ddr:3.0-0\|cnn\_GOOD | Read | OCL Region | 1 | 1 | 138243 | 138244 | 0.00333333 | 4002.252819 | 4002.252822 |
| 4002.252869 | Kernel\_Read\|xilinx:adm-pcie-7v3:1ddr:3.0-0\|cnn\_GOOD | Read | OCL Region | 1 | 1 | 138258 | 138259 | 0.00333333 | 4002.252869 | 4002.252872 |
| 4002.252919 | Kernel\_Read\|xilinx:adm-pcie-7v3:1ddr:3.0-0\|cnn\_GOOD | Read | OCL Region | 1 | 1 | 138273 | 138274 | 0.00333333 | 4002.252919 | 4002.252922 |
| 4002.252969 | Kernel\_Read\|xilinx:adm-pcie-7v3:1ddr:3.0-0\|cnn\_GOOD | Read | OCL Region | 1 | 1 | 138288 | 138289 | 0.00333333 | 4002.252969 | 4002.252972 |
| 4002.253032 | Kernel\_Read\|xilinx:adm-pcie-7v3:1ddr:3.0-0\|cnn\_GOOD | Read | OCL Region | 1 | 1 | 138307 | 138308 | 0.00333333 | 4002.253032 | 4002.253036 |
| 4002.253082 | Kernel\_Read\|xilinx:adm-pcie-7v3:1ddr:3.0-0\|cnn\_GOOD | Read | OCL Region | 1 | 1 | 138322 | 138323 | 0.00333333 | 4002.253082 | 4002.253086 |
| 4002.253129 | Kernel\_Read\|xilinx:adm-pcie-7v3:1ddr:3.0-0\|cnn\_GOOD | Read | OCL Region | 1 | 1 | 138336 | 138337 | 0.00333333 | 4002.253129 | 4002.253132 |
| 4002.253182 | Kernel\_Read\|xilinx:adm-pcie-7v3:1ddr:3.0-0\|cnn\_GOOD | Read | OCL Region | 1 | 1 | 138352 | 138353 | 0.00333333 | 4002.253182 | 4002.253186 |
| 4002.253232 | Kernel\_Read\|xilinx:adm-pcie-7v3:1ddr:3.0-0\|cnn\_GOOD | Read | OCL Region | 1 | 1 | 138367 | 138368 | 0.00333333 | 4002.253232 | 4002.253236 |
| 4002.253279 | Kernel\_Read\|xilinx:adm-pcie-7v3:1ddr:3.0-0\|cnn\_GOOD | Read | OCL Region | 1 | 1 | 138381 | 138382 | 0.00333333 | 4002.253279 | 4002.253282 |
| 4002.253329 | Kernel\_Read\|xilinx:adm-pcie-7v3:1ddr:3.0-0\|cnn\_GOOD | Read | OCL Region | 1 | 1 | 138396 | 138397 | 0.00333333 | 4002.253329 | 4002.253332 |
| 4002.253382 | Kernel\_Read\|xilinx:adm-pcie-7v3:1ddr:3.0-0\|cnn\_GOOD | Read | OCL Region | 1 | 1 | 138412 | 138413 | 0.00333333 | 4002.253382 | 4002.253386 |
| 4002.253432 | Kernel\_Read\|xilinx:adm-pcie-7v3:1ddr:3.0-0\|cnn\_GOOD | Read | OCL Region | 1 | 1 | 138427 | 138428 | 0.00333333 | 4002.253432 | 4002.253436 |
| 4002.253482 | Kernel\_Read\|xilinx:adm-pcie-7v3:1ddr:3.0-0\|cnn\_GOOD | Read | OCL Region | 1 | 1 | 138442 | 138443 | 0.00333333 | 4002.253482 | 4002.253486 |
| 4002.253529 | Kernel\_Read\|xilinx:adm-pcie-7v3:1ddr:3.0-0\|cnn\_GOOD | Read | OCL Region | 1 | 1 | 138456 | 138457 | 0.00333333 | 4002.253529 | 4002.253532 |
| 4002.253582 | Kernel\_Read\|xilinx:adm-pcie-7v3:1ddr:3.0-0\|cnn\_GOOD | Read | OCL Region | 1 | 1 | 138472 | 138473 | 0.00333333 | 4002.253582 | 4002.253586 |
| 4002.253632 | Kernel\_Read\|xilinx:adm-pcie-7v3:1ddr:3.0-0\|cnn\_GOOD | Read | OCL Region | 1 | 1 | 138487 | 138488 | 0.00333333 | 4002.253632 | 4002.253636 |
| 4002.253679 | Kernel\_Read\|xilinx:adm-pcie-7v3:1ddr:3.0-0\|cnn\_GOOD | Read | OCL Region | 1 | 1 | 138501 | 138502 | 0.00333333 | 4002.253679 | 4002.253682 |
| 4002.253729 | Kernel\_Read\|xilinx:adm-pcie-7v3:1ddr:3.0-0\|cnn\_GOOD | Read | OCL Region | 1 | 1 | 138516 | 138517 | 0.00333333 | 4002.253729 | 4002.253732 |
| 4002.253782 | Kernel\_Read\|xilinx:adm-pcie-7v3:1ddr:3.0-0\|cnn\_GOOD | Read | OCL Region | 1 | 1 | 138532 | 138533 | 0.00333333 | 4002.253782 | 4002.253786 |
| 4002.253832 | Kernel\_Read\|xilinx:adm-pcie-7v3:1ddr:3.0-0\|cnn\_GOOD | Read | OCL Region | 1 | 1 | 138547 | 138548 | 0.00333333 | 4002.253832 | 4002.253836 |
| 4002.253882 | Kernel\_Read\|xilinx:adm-pcie-7v3:1ddr:3.0-0\|cnn\_GOOD | Read | OCL Region | 1 | 1 | 138562 | 138563 | 0.00333333 | 4002.253882 | 4002.253886 |
| 4002.253929 | Kernel\_Read\|xilinx:adm-pcie-7v3:1ddr:3.0-0\|cnn\_GOOD | Read | OCL Region | 1 | 1 | 138576 | 138577 | 0.00333333 | 4002.253929 | 4002.253932 |
| 4002.253992 | Kernel\_Read\|xilinx:adm-pcie-7v3:1ddr:3.0-0\|cnn\_GOOD | Read | OCL Region | 1 | 1 | 138595 | 138596 | 0.00333333 | 4002.253992 | 4002.253996 |
| 4002.254042 | Kernel\_Read\|xilinx:adm-pcie-7v3:1ddr:3.0-0\|cnn\_GOOD | Read | OCL Region | 1 | 1 | 138610 | 138611 | 0.00333333 | 4002.254042 | 4002.254046 |
| 4002.254092 | Kernel\_Read\|xilinx:adm-pcie-7v3:1ddr:3.0-0\|cnn\_GOOD | Read | OCL Region | 1 | 1 | 138625 | 138626 | 0.00333333 | 4002.254092 | 4002.254096 |
| 4002.254142 | Kernel\_Read\|xilinx:adm-pcie-7v3:1ddr:3.0-0\|cnn\_GOOD | Read | OCL Region | 1 | 1 | 138640 | 138641 | 0.00333333 | 4002.254142 | 4002.254146 |
| 4002.254192 | Kernel\_Read\|xilinx:adm-pcie-7v3:1ddr:3.0-0\|cnn\_GOOD | Read | OCL Region | 1 | 1 | 138655 | 138656 | 0.00333333 | 4002.254192 | 4002.254196 |
| 4002.254242 | Kernel\_Read\|xilinx:adm-pcie-7v3:1ddr:3.0-0\|cnn\_GOOD | Read | OCL Region | 1 | 1 | 138670 | 138671 | 0.00333333 | 4002.254242 | 4002.254246 |
| 4002.254292 | Kernel\_Read\|xilinx:adm-pcie-7v3:1ddr:3.0-0\|cnn\_GOOD | Read | OCL Region | 1 | 1 | 138685 | 138686 | 0.00333333 | 4002.254292 | 4002.254296 |
| 4002.254342 | Kernel\_Read\|xilinx:adm-pcie-7v3:1ddr:3.0-0\|cnn\_GOOD | Read | OCL Region | 1 | 1 | 138700 | 138701 | 0.00333333 | 4002.254342 | 4002.254346 |
| 4002.254392 | Kernel\_Read\|xilinx:adm-pcie-7v3:1ddr:3.0-0\|cnn\_GOOD | Read | OCL Region | 1 | 1 | 138715 | 138716 | 0.00333333 | 4002.254392 | 4002.254396 |
| 4002.254442 | Kernel\_Read\|xilinx:adm-pcie-7v3:1ddr:3.0-0\|cnn\_GOOD | Read | OCL Region | 1 | 1 | 138730 | 138731 | 0.00333333 | 4002.254442 | 4002.254446 |
| 4002.254492 | Kernel\_Read\|xilinx:adm-pcie-7v3:1ddr:3.0-0\|cnn\_GOOD | Read | OCL Region | 1 | 1 | 138745 | 138746 | 0.00333333 | 4002.254492 | 4002.254496 |
| 4002.254542 | Kernel\_Read\|xilinx:adm-pcie-7v3:1ddr:3.0-0\|cnn\_GOOD | Read | OCL Region | 1 | 1 | 138760 | 138761 | 0.00333333 | 4002.254542 | 4002.254546 |
| 4002.254592 | Kernel\_Read\|xilinx:adm-pcie-7v3:1ddr:3.0-0\|cnn\_GOOD | Read | OCL Region | 1 | 1 | 138775 | 138776 | 0.00333333 | 4002.254592 | 4002.254596 |
| 4002.254642 | Kernel\_Read\|xilinx:adm-pcie-7v3:1ddr:3.0-0\|cnn\_GOOD | Read | OCL Region | 1 | 1 | 138790 | 138791 | 0.00333333 | 4002.254642 | 4002.254646 |
| 4002.254692 | Kernel\_Read\|xilinx:adm-pcie-7v3:1ddr:3.0-0\|cnn\_GOOD | Read | OCL Region | 1 | 1 | 138805 | 138806 | 0.00333333 | 4002.254692 | 4002.254696 |
| 4002.254742 | Kernel\_Read\|xilinx:adm-pcie-7v3:1ddr:3.0-0\|cnn\_GOOD | Read | OCL Region | 1 | 1 | 138820 | 138821 | 0.00333333 | 4002.254742 | 4002.254746 |
| 4002.254792 | Kernel\_Read\|xilinx:adm-pcie-7v3:1ddr:3.0-0\|cnn\_GOOD | Read | OCL Region | 1 | 1 | 138835 | 138836 | 0.00333333 | 4002.254792 | 4002.254796 |
| 4002.254842 | Kernel\_Read\|xilinx:adm-pcie-7v3:1ddr:3.0-0\|cnn\_GOOD | Read | OCL Region | 1 | 1 | 138850 | 138851 | 0.00333333 | 4002.254842 | 4002.254846 |
| 4002.254892 | Kernel\_Read\|xilinx:adm-pcie-7v3:1ddr:3.0-0\|cnn\_GOOD | Read | OCL Region | 1 | 1 | 138865 | 138866 | 0.00333333 | 4002.254892 | 4002.254896 |
| 4002.254942 | Kernel\_Read\|xilinx:adm-pcie-7v3:1ddr:3.0-0\|cnn\_GOOD | Read | OCL Region | 1 | 1 | 138880 | 138881 | 0.00333333 | 4002.254942 | 4002.254946 |
| 4002.254999 | Kernel\_Read\|xilinx:adm-pcie-7v3:1ddr:3.0-0\|cnn\_GOOD | Read | OCL Region | 1 | 1 | 138897 | 138898 | 0.00333333 | 4002.254999 | 4002.255002 |
| 4002.255049 | Kernel\_Read\|xilinx:adm-pcie-7v3:1ddr:3.0-0\|cnn\_GOOD | Read | OCL Region | 1 | 1 | 138912 | 138913 | 0.00333333 | 4002.255049 | 4002.255052 |
| 4002.255099 | Kernel\_Read\|xilinx:adm-pcie-7v3:1ddr:3.0-0\|cnn\_GOOD | Read | OCL Region | 1 | 1 | 138927 | 138928 | 0.00333333 | 4002.255099 | 4002.255102 |
| 4002.255149 | Kernel\_Read\|xilinx:adm-pcie-7v3:1ddr:3.0-0\|cnn\_GOOD | Read | OCL Region | 1 | 1 | 138942 | 138943 | 0.00333333 | 4002.255149 | 4002.255152 |
| 4002.255199 | Kernel\_Read\|xilinx:adm-pcie-7v3:1ddr:3.0-0\|cnn\_GOOD | Read | OCL Region | 1 | 1 | 138957 | 138958 | 0.00333333 | 4002.255199 | 4002.255202 |
| 4002.255249 | Kernel\_Read\|xilinx:adm-pcie-7v3:1ddr:3.0-0\|cnn\_GOOD | Read | OCL Region | 1 | 1 | 138972 | 138973 | 0.00333333 | 4002.255249 | 4002.255252 |
| 4002.255299 | Kernel\_Read\|xilinx:adm-pcie-7v3:1ddr:3.0-0\|cnn\_GOOD | Read | OCL Region | 1 | 1 | 138987 | 138988 | 0.00333333 | 4002.255299 | 4002.255302 |
| 4002.255349 | Kernel\_Read\|xilinx:adm-pcie-7v3:1ddr:3.0-0\|cnn\_GOOD | Read | OCL Region | 1 | 1 | 139002 | 139003 | 0.00333333 | 4002.255349 | 4002.255352 |
| 4002.255399 | Kernel\_Read\|xilinx:adm-pcie-7v3:1ddr:3.0-0\|cnn\_GOOD | Read | OCL Region | 1 | 1 | 139017 | 139018 | 0.00333333 | 4002.255399 | 4002.255402 |
| 4002.255449 | Kernel\_Read\|xilinx:adm-pcie-7v3:1ddr:3.0-0\|cnn\_GOOD | Read | OCL Region | 1 | 1 | 139032 | 139033 | 0.00333333 | 4002.255449 | 4002.255452 |
| 4002.255499 | Kernel\_Read\|xilinx:adm-pcie-7v3:1ddr:3.0-0\|cnn\_GOOD | Read | OCL Region | 1 | 1 | 139047 | 139048 | 0.00333333 | 4002.255499 | 4002.255502 |
| 4002.255549 | Kernel\_Read\|xilinx:adm-pcie-7v3:1ddr:3.0-0\|cnn\_GOOD | Read | OCL Region | 1 | 1 | 139062 | 139063 | 0.00333333 | 4002.255549 | 4002.255552 |
| 4002.255599 | Kernel\_Read\|xilinx:adm-pcie-7v3:1ddr:3.0-0\|cnn\_GOOD | Read | OCL Region | 1 | 1 | 139077 | 139078 | 0.00333333 | 4002.255599 | 4002.255602 |
| 4002.255649 | Kernel\_Read\|xilinx:adm-pcie-7v3:1ddr:3.0-0\|cnn\_GOOD | Read | OCL Region | 1 | 1 | 139092 | 139093 | 0.00333333 | 4002.255649 | 4002.255652 |
| 4002.255699 | Kernel\_Read\|xilinx:adm-pcie-7v3:1ddr:3.0-0\|cnn\_GOOD | Read | OCL Region | 1 | 1 | 139107 | 139108 | 0.00333333 | 4002.255699 | 4002.255702 |
| 4002.255749 | Kernel\_Read\|xilinx:adm-pcie-7v3:1ddr:3.0-0\|cnn\_GOOD | Read | OCL Region | 1 | 1 | 139122 | 139123 | 0.00333333 | 4002.255749 | 4002.255752 |
| 4002.255799 | Kernel\_Read\|xilinx:adm-pcie-7v3:1ddr:3.0-0\|cnn\_GOOD | Read | OCL Region | 1 | 1 | 139137 | 139138 | 0.00333333 | 4002.255799 | 4002.255802 |
| 4002.255849 | Kernel\_Read\|xilinx:adm-pcie-7v3:1ddr:3.0-0\|cnn\_GOOD | Read | OCL Region | 1 | 1 | 139152 | 139153 | 0.00333333 | 4002.255849 | 4002.255852 |
| 4002.255899 | Kernel\_Read\|xilinx:adm-pcie-7v3:1ddr:3.0-0\|cnn\_GOOD | Read | OCL Region | 1 | 1 | 139167 | 139168 | 0.00333333 | 4002.255899 | 4002.255902 |
| 4002.255949 | Kernel\_Read\|xilinx:adm-pcie-7v3:1ddr:3.0-0\|cnn\_GOOD | Read | OCL Region | 1 | 1 | 139182 | 139183 | 0.00333333 | 4002.255949 | 4002.255952 |
| 4002.256012 | Kernel\_Read\|xilinx:adm-pcie-7v3:1ddr:3.0-0\|cnn\_GOOD | Read | OCL Region | 1 | 1 | 139201 | 139202 | 0.00333333 | 4002.256012 | 4002.256016 |
| 4002.256062 | Kernel\_Read\|xilinx:adm-pcie-7v3:1ddr:3.0-0\|cnn\_GOOD | Read | OCL Region | 1 | 1 | 139216 | 139217 | 0.00333333 | 4002.256062 | 4002.256066 |
| 4002.256112 | Kernel\_Read\|xilinx:adm-pcie-7v3:1ddr:3.0-0\|cnn\_GOOD | Read | OCL Region | 1 | 1 | 139231 | 139232 | 0.00333333 | 4002.256112 | 4002.256116 |
| 4002.256162 | Kernel\_Read\|xilinx:adm-pcie-7v3:1ddr:3.0-0\|cnn\_GOOD | Read | OCL Region | 1 | 1 | 139246 | 139247 | 0.00333333 | 4002.256162 | 4002.256166 |
| 4002.256212 | Kernel\_Read\|xilinx:adm-pcie-7v3:1ddr:3.0-0\|cnn\_GOOD | Read | OCL Region | 1 | 1 | 139261 | 139262 | 0.00333333 | 4002.256212 | 4002.256216 |
| 4002.256262 | Kernel\_Read\|xilinx:adm-pcie-7v3:1ddr:3.0-0\|cnn\_GOOD | Read | OCL Region | 1 | 1 | 139276 | 139277 | 0.00333333 | 4002.256262 | 4002.256266 |
| 4002.256312 | Kernel\_Read\|xilinx:adm-pcie-7v3:1ddr:3.0-0\|cnn\_GOOD | Read | OCL Region | 1 | 1 | 139291 | 139292 | 0.00333333 | 4002.256312 | 4002.256316 |
| 4002.256362 | Kernel\_Read\|xilinx:adm-pcie-7v3:1ddr:3.0-0\|cnn\_GOOD | Read | OCL Region | 1 | 1 | 139306 | 139307 | 0.00333333 | 4002.256362 | 4002.256366 |
| 4002.256412 | Kernel\_Read\|xilinx:adm-pcie-7v3:1ddr:3.0-0\|cnn\_GOOD | Read | OCL Region | 1 | 1 | 139321 | 139322 | 0.00333333 | 4002.256412 | 4002.256416 |
| 4002.256462 | Kernel\_Read\|xilinx:adm-pcie-7v3:1ddr:3.0-0\|cnn\_GOOD | Read | OCL Region | 1 | 1 | 139336 | 139337 | 0.00333333 | 4002.256462 | 4002.256466 |
| 4002.256512 | Kernel\_Read\|xilinx:adm-pcie-7v3:1ddr:3.0-0\|cnn\_GOOD | Read | OCL Region | 1 | 1 | 139351 | 139352 | 0.00333333 | 4002.256512 | 4002.256516 |
| 4002.256562 | Kernel\_Read\|xilinx:adm-pcie-7v3:1ddr:3.0-0\|cnn\_GOOD | Read | OCL Region | 1 | 1 | 139366 | 139367 | 0.00333333 | 4002.256562 | 4002.256566 |
| 4002.256612 | Kernel\_Read\|xilinx:adm-pcie-7v3:1ddr:3.0-0\|cnn\_GOOD | Read | OCL Region | 1 | 1 | 139381 | 139382 | 0.00333333 | 4002.256612 | 4002.256616 |
| 4002.256662 | Kernel\_Read\|xilinx:adm-pcie-7v3:1ddr:3.0-0\|cnn\_GOOD | Read | OCL Region | 1 | 1 | 139396 | 139397 | 0.00333333 | 4002.256662 | 4002.256666 |
| 4002.256712 | Kernel\_Read\|xilinx:adm-pcie-7v3:1ddr:3.0-0\|cnn\_GOOD | Read | OCL Region | 1 | 1 | 139411 | 139412 | 0.00333333 | 4002.256712 | 4002.256716 |
| 4002.256762 | Kernel\_Read\|xilinx:adm-pcie-7v3:1ddr:3.0-0\|cnn\_GOOD | Read | OCL Region | 1 | 1 | 139426 | 139427 | 0.00333333 | 4002.256762 | 4002.256766 |
| 4002.257032 | Kernel\_Read\|xilinx:adm-pcie-7v3:1ddr:3.0-0\|cnn\_GOOD | Read | OCL Region | 1 | 1 | 139507 | 139508 | 0.00333333 | 4002.257032 | 4002.257036 |
| 4002.257079 | Kernel\_Read\|xilinx:adm-pcie-7v3:1ddr:3.0-0\|cnn\_GOOD | Read | OCL Region | 1 | 1 | 139521 | 139522 | 0.00333333 | 4002.257079 | 4002.257082 |
| 4002.257129 | Kernel\_Read\|xilinx:adm-pcie-7v3:1ddr:3.0-0\|cnn\_GOOD | Read | OCL Region | 1 | 1 | 139536 | 139537 | 0.00333333 | 4002.257129 | 4002.257132 |
| 4002.257192 | Kernel\_Read\|xilinx:adm-pcie-7v3:1ddr:3.0-0\|cnn\_GOOD | Read | OCL Region | 1 | 1 | 139555 | 139556 | 0.00333333 | 4002.257192 | 4002.257196 |
| 4002.257242 | Kernel\_Read\|xilinx:adm-pcie-7v3:1ddr:3.0-0\|cnn\_GOOD | Read | OCL Region | 1 | 1 | 139570 | 139571 | 0.00333333 | 4002.257242 | 4002.257246 |
| 4002.257292 | Kernel\_Read\|xilinx:adm-pcie-7v3:1ddr:3.0-0\|cnn\_GOOD | Read | OCL Region | 1 | 1 | 139585 | 139586 | 0.00333333 | 4002.257292 | 4002.257296 |
| 4002.257342 | Kernel\_Read\|xilinx:adm-pcie-7v3:1ddr:3.0-0\|cnn\_GOOD | Read | OCL Region | 1 | 1 | 139600 | 139601 | 0.00333333 | 4002.257342 | 4002.257346 |
| 4002.257392 | Kernel\_Read\|xilinx:adm-pcie-7v3:1ddr:3.0-0\|cnn\_GOOD | Read | OCL Region | 1 | 1 | 139615 | 139616 | 0.00333333 | 4002.257392 | 4002.257396 |
| 4002.257442 | Kernel\_Read\|xilinx:adm-pcie-7v3:1ddr:3.0-0\|cnn\_GOOD | Read | OCL Region | 1 | 1 | 139630 | 139631 | 0.00333333 | 4002.257442 | 4002.257446 |
| 4002.257492 | Kernel\_Read\|xilinx:adm-pcie-7v3:1ddr:3.0-0\|cnn\_GOOD | Read | OCL Region | 1 | 1 | 139645 | 139646 | 0.00333333 | 4002.257492 | 4002.257496 |
| 4002.257542 | Kernel\_Read\|xilinx:adm-pcie-7v3:1ddr:3.0-0\|cnn\_GOOD | Read | OCL Region | 1 | 1 | 139660 | 139661 | 0.00333333 | 4002.257542 | 4002.257546 |
| 4002.257592 | Kernel\_Read\|xilinx:adm-pcie-7v3:1ddr:3.0-0\|cnn\_GOOD | Read | OCL Region | 1 | 1 | 139675 | 139676 | 0.00333333 | 4002.257592 | 4002.257596 |
| 4002.257642 | Kernel\_Read\|xilinx:adm-pcie-7v3:1ddr:3.0-0\|cnn\_GOOD | Read | OCL Region | 1 | 1 | 139690 | 139691 | 0.00333333 | 4002.257642 | 4002.257646 |
| 4002.257692 | Kernel\_Read\|xilinx:adm-pcie-7v3:1ddr:3.0-0\|cnn\_GOOD | Read | OCL Region | 1 | 1 | 139705 | 139706 | 0.00333333 | 4002.257692 | 4002.257696 |
| 4002.257742 | Kernel\_Read\|xilinx:adm-pcie-7v3:1ddr:3.0-0\|cnn\_GOOD | Read | OCL Region | 1 | 1 | 139720 | 139721 | 0.00333333 | 4002.257742 | 4002.257746 |
| 4002.257792 | Kernel\_Read\|xilinx:adm-pcie-7v3:1ddr:3.0-0\|cnn\_GOOD | Read | OCL Region | 1 | 1 | 139735 | 139736 | 0.00333333 | 4002.257792 | 4002.257796 |
| 4002.257842 | Kernel\_Read\|xilinx:adm-pcie-7v3:1ddr:3.0-0\|cnn\_GOOD | Read | OCL Region | 1 | 1 | 139750 | 139751 | 0.00333333 | 4002.257842 | 4002.257846 |
| 4002.257892 | Kernel\_Read\|xilinx:adm-pcie-7v3:1ddr:3.0-0\|cnn\_GOOD | Read | OCL Region | 1 | 1 | 139765 | 139766 | 0.00333333 | 4002.257892 | 4002.257896 |
| 4002.257942 | Kernel\_Read\|xilinx:adm-pcie-7v3:1ddr:3.0-0\|cnn\_GOOD | Read | OCL Region | 1 | 1 | 139780 | 139781 | 0.00333333 | 4002.257942 | 4002.257946 |
| 4002.257992 | Kernel\_Read\|xilinx:adm-pcie-7v3:1ddr:3.0-0\|cnn\_GOOD | Read | OCL Region | 1 | 1 | 139795 | 139796 | 0.00333333 | 4002.257992 | 4002.257996 |
| 4002.258042 | Kernel\_Read\|xilinx:adm-pcie-7v3:1ddr:3.0-0\|cnn\_GOOD | Read | OCL Region | 1 | 1 | 139810 | 139811 | 0.00333333 | 4002.258042 | 4002.258046 |
| 4002.258092 | Kernel\_Read\|xilinx:adm-pcie-7v3:1ddr:3.0-0\|cnn\_GOOD | Read | OCL Region | 1 | 1 | 139825 | 139826 | 0.00333333 | 4002.258092 | 4002.258096 |
| 4002.258149 | Kernel\_Read\|xilinx:adm-pcie-7v3:1ddr:3.0-0\|cnn\_GOOD | Read | OCL Region | 1 | 1 | 139842 | 139843 | 0.00333333 | 4002.258149 | 4002.258152 |
| 4002.258199 | Kernel\_Read\|xilinx:adm-pcie-7v3:1ddr:3.0-0\|cnn\_GOOD | Read | OCL Region | 1 | 1 | 139857 | 139858 | 0.00333333 | 4002.258199 | 4002.258202 |
| 4002.258249 | Kernel\_Read\|xilinx:adm-pcie-7v3:1ddr:3.0-0\|cnn\_GOOD | Read | OCL Region | 1 | 1 | 139872 | 139873 | 0.00333333 | 4002.258249 | 4002.258252 |
| 4002.258299 | Kernel\_Read\|xilinx:adm-pcie-7v3:1ddr:3.0-0\|cnn\_GOOD | Read | OCL Region | 1 | 1 | 139887 | 139888 | 0.00333333 | 4002.258299 | 4002.258302 |
| 4002.258349 | Kernel\_Read\|xilinx:adm-pcie-7v3:1ddr:3.0-0\|cnn\_GOOD | Read | OCL Region | 1 | 1 | 139902 | 139903 | 0.00333333 | 4002.258349 | 4002.258352 |
| 4002.258399 | Kernel\_Read\|xilinx:adm-pcie-7v3:1ddr:3.0-0\|cnn\_GOOD | Read | OCL Region | 1 | 1 | 139917 | 139918 | 0.00333333 | 4002.258399 | 4002.258402 |
| 4002.258449 | Kernel\_Read\|xilinx:adm-pcie-7v3:1ddr:3.0-0\|cnn\_GOOD | Read | OCL Region | 1 | 1 | 139932 | 139933 | 0.00333333 | 4002.258449 | 4002.258452 |
| 4002.258499 | Kernel\_Read\|xilinx:adm-pcie-7v3:1ddr:3.0-0\|cnn\_GOOD | Read | OCL Region | 1 | 1 | 139947 | 139948 | 0.00333333 | 4002.258499 | 4002.258502 |
| 4002.258549 | Kernel\_Read\|xilinx:adm-pcie-7v3:1ddr:3.0-0\|cnn\_GOOD | Read | OCL Region | 1 | 1 | 139962 | 139963 | 0.00333333 | 4002.258549 | 4002.258552 |
| 4002.258599 | Kernel\_Read\|xilinx:adm-pcie-7v3:1ddr:3.0-0\|cnn\_GOOD | Read | OCL Region | 1 | 1 | 139977 | 139978 | 0.00333333 | 4002.258599 | 4002.258602 |
| 4002.258649 | Kernel\_Read\|xilinx:adm-pcie-7v3:1ddr:3.0-0\|cnn\_GOOD | Read | OCL Region | 1 | 1 | 139992 | 139993 | 0.00333333 | 4002.258649 | 4002.258652 |
| 4002.258699 | Kernel\_Read\|xilinx:adm-pcie-7v3:1ddr:3.0-0\|cnn\_GOOD | Read | OCL Region | 1 | 1 | 140007 | 140008 | 0.00333333 | 4002.258699 | 4002.258702 |
| 4002.258749 | Kernel\_Read\|xilinx:adm-pcie-7v3:1ddr:3.0-0\|cnn\_GOOD | Read | OCL Region | 1 | 1 | 140022 | 140023 | 0.00333333 | 4002.258749 | 4002.258752 |
| 4002.258799 | Kernel\_Read\|xilinx:adm-pcie-7v3:1ddr:3.0-0\|cnn\_GOOD | Read | OCL Region | 1 | 1 | 140037 | 140038 | 0.00333333 | 4002.258799 | 4002.258802 |
| 4002.258849 | Kernel\_Read\|xilinx:adm-pcie-7v3:1ddr:3.0-0\|cnn\_GOOD | Read | OCL Region | 1 | 1 | 140052 | 140053 | 0.00333333 | 4002.258849 | 4002.258852 |
| 4002.258899 | Kernel\_Read\|xilinx:adm-pcie-7v3:1ddr:3.0-0\|cnn\_GOOD | Read | OCL Region | 1 | 1 | 140067 | 140068 | 0.00333333 | 4002.258899 | 4002.258902 |
| 4002.258949 | Kernel\_Read\|xilinx:adm-pcie-7v3:1ddr:3.0-0\|cnn\_GOOD | Read | OCL Region | 1 | 1 | 140082 | 140083 | 0.00333333 | 4002.258949 | 4002.258952 |
| 4002.258999 | Kernel\_Read\|xilinx:adm-pcie-7v3:1ddr:3.0-0\|cnn\_GOOD | Read | OCL Region | 1 | 1 | 140097 | 140098 | 0.00333333 | 4002.258999 | 4002.259002 |
| 4002.259049 | Kernel\_Read\|xilinx:adm-pcie-7v3:1ddr:3.0-0\|cnn\_GOOD | Read | OCL Region | 1 | 1 | 140112 | 140113 | 0.00333333 | 4002.259049 | 4002.259052 |
| 4002.259112 | Kernel\_Read\|xilinx:adm-pcie-7v3:1ddr:3.0-0\|cnn\_GOOD | Read | OCL Region | 1 | 1 | 140131 | 140132 | 0.00333333 | 4002.259112 | 4002.259116 |
| 4002.259162 | Kernel\_Read\|xilinx:adm-pcie-7v3:1ddr:3.0-0\|cnn\_GOOD | Read | OCL Region | 1 | 1 | 140146 | 140147 | 0.00333333 | 4002.259162 | 4002.259166 |
| 4002.259212 | Kernel\_Read\|xilinx:adm-pcie-7v3:1ddr:3.0-0\|cnn\_GOOD | Read | OCL Region | 1 | 1 | 140161 | 140162 | 0.00333333 | 4002.259212 | 4002.259216 |
| 4002.259262 | Kernel\_Read\|xilinx:adm-pcie-7v3:1ddr:3.0-0\|cnn\_GOOD | Read | OCL Region | 1 | 1 | 140176 | 140177 | 0.00333333 | 4002.259262 | 4002.259266 |
| 4002.259312 | Kernel\_Read\|xilinx:adm-pcie-7v3:1ddr:3.0-0\|cnn\_GOOD | Read | OCL Region | 1 | 1 | 140191 | 140192 | 0.00333333 | 4002.259312 | 4002.259316 |
| 4002.259362 | Kernel\_Read\|xilinx:adm-pcie-7v3:1ddr:3.0-0\|cnn\_GOOD | Read | OCL Region | 1 | 1 | 140206 | 140207 | 0.00333333 | 4002.259362 | 4002.259366 |
| 4002.259412 | Kernel\_Read\|xilinx:adm-pcie-7v3:1ddr:3.0-0\|cnn\_GOOD | Read | OCL Region | 1 | 1 | 140221 | 140222 | 0.00333333 | 4002.259412 | 4002.259416 |
| 4002.259462 | Kernel\_Read\|xilinx:adm-pcie-7v3:1ddr:3.0-0\|cnn\_GOOD | Read | OCL Region | 1 | 1 | 140236 | 140237 | 0.00333333 | 4002.259462 | 4002.259466 |
| 4002.259512 | Kernel\_Read\|xilinx:adm-pcie-7v3:1ddr:3.0-0\|cnn\_GOOD | Read | OCL Region | 1 | 1 | 140251 | 140252 | 0.00333333 | 4002.259512 | 4002.259516 |
| 4002.259562 | Kernel\_Read\|xilinx:adm-pcie-7v3:1ddr:3.0-0\|cnn\_GOOD | Read | OCL Region | 1 | 1 | 140266 | 140267 | 0.00333333 | 4002.259562 | 4002.259566 |
| 4002.259612 | Kernel\_Read\|xilinx:adm-pcie-7v3:1ddr:3.0-0\|cnn\_GOOD | Read | OCL Region | 1 | 1 | 140281 | 140282 | 0.00333333 | 4002.259612 | 4002.259616 |
| 4002.259662 | Kernel\_Read\|xilinx:adm-pcie-7v3:1ddr:3.0-0\|cnn\_GOOD | Read | OCL Region | 1 | 1 | 140296 | 140297 | 0.00333333 | 4002.259662 | 4002.259666 |
| 4002.259712 | Kernel\_Read\|xilinx:adm-pcie-7v3:1ddr:3.0-0\|cnn\_GOOD | Read | OCL Region | 1 | 1 | 140311 | 140312 | 0.00333333 | 4002.259712 | 4002.259716 |
| 4002.259762 | Kernel\_Read\|xilinx:adm-pcie-7v3:1ddr:3.0-0\|cnn\_GOOD | Read | OCL Region | 1 | 1 | 140326 | 140327 | 0.00333333 | 4002.259762 | 4002.259766 |
| 4002.259812 | Kernel\_Read\|xilinx:adm-pcie-7v3:1ddr:3.0-0\|cnn\_GOOD | Read | OCL Region | 1 | 1 | 140341 | 140342 | 0.00333333 | 4002.259812 | 4002.259816 |
| 4002.259862 | Kernel\_Read\|xilinx:adm-pcie-7v3:1ddr:3.0-0\|cnn\_GOOD | Read | OCL Region | 1 | 1 | 140356 | 140357 | 0.00333333 | 4002.259862 | 4002.259866 |
| 4002.259912 | Kernel\_Read\|xilinx:adm-pcie-7v3:1ddr:3.0-0\|cnn\_GOOD | Read | OCL Region | 1 | 1 | 140371 | 140372 | 0.00333333 | 4002.259912 | 4002.259916 |
| 4002.259962 | Kernel\_Read\|xilinx:adm-pcie-7v3:1ddr:3.0-0\|cnn\_GOOD | Read | OCL Region | 1 | 1 | 140386 | 140387 | 0.00333333 | 4002.259962 | 4002.259966 |
| 4002.260012 | Kernel\_Read\|xilinx:adm-pcie-7v3:1ddr:3.0-0\|cnn\_GOOD | Read | OCL Region | 1 | 1 | 140401 | 140402 | 0.00333333 | 4002.260012 | 4002.260016 |
| 4002.260062 | Kernel\_Read\|xilinx:adm-pcie-7v3:1ddr:3.0-0\|cnn\_GOOD | Read | OCL Region | 1 | 1 | 140416 | 140417 | 0.00333333 | 4002.260062 | 4002.260066 |
| 4002.260119 | Kernel\_Read\|xilinx:adm-pcie-7v3:1ddr:3.0-0\|cnn\_GOOD | Read | OCL Region | 1 | 1 | 140433 | 140434 | 0.00333333 | 4002.260119 | 4002.260122 |
| 4002.260169 | Kernel\_Read\|xilinx:adm-pcie-7v3:1ddr:3.0-0\|cnn\_GOOD | Read | OCL Region | 1 | 1 | 140448 | 140449 | 0.00333333 | 4002.260169 | 4002.260172 |
| 4002.260219 | Kernel\_Read\|xilinx:adm-pcie-7v3:1ddr:3.0-0\|cnn\_GOOD | Read | OCL Region | 1 | 1 | 140463 | 140464 | 0.00333333 | 4002.260219 | 4002.260222 |
| 4002.260269 | Kernel\_Read\|xilinx:adm-pcie-7v3:1ddr:3.0-0\|cnn\_GOOD | Read | OCL Region | 1 | 1 | 140478 | 140479 | 0.00333333 | 4002.260269 | 4002.260272 |
| 4002.260319 | Kernel\_Read\|xilinx:adm-pcie-7v3:1ddr:3.0-0\|cnn\_GOOD | Read | OCL Region | 1 | 1 | 140493 | 140494 | 0.00333333 | 4002.260319 | 4002.260322 |
| 4002.260369 | Kernel\_Read\|xilinx:adm-pcie-7v3:1ddr:3.0-0\|cnn\_GOOD | Read | OCL Region | 1 | 1 | 140508 | 140509 | 0.00333333 | 4002.260369 | 4002.260372 |
| 4002.260419 | Kernel\_Read\|xilinx:adm-pcie-7v3:1ddr:3.0-0\|cnn\_GOOD | Read | OCL Region | 1 | 1 | 140523 | 140524 | 0.00333333 | 4002.260419 | 4002.260422 |
| 4002.260469 | Kernel\_Read\|xilinx:adm-pcie-7v3:1ddr:3.0-0\|cnn\_GOOD | Read | OCL Region | 1 | 1 | 140538 | 140539 | 0.00333333 | 4002.260469 | 4002.260472 |
| 4002.260519 | Kernel\_Read\|xilinx:adm-pcie-7v3:1ddr:3.0-0\|cnn\_GOOD | Read | OCL Region | 1 | 1 | 140553 | 140554 | 0.00333333 | 4002.260519 | 4002.260522 |
| 4002.260569 | Kernel\_Read\|xilinx:adm-pcie-7v3:1ddr:3.0-0\|cnn\_GOOD | Read | OCL Region | 1 | 1 | 140568 | 140569 | 0.00333333 | 4002.260569 | 4002.260572 |
| 4002.260619 | Kernel\_Read\|xilinx:adm-pcie-7v3:1ddr:3.0-0\|cnn\_GOOD | Read | OCL Region | 1 | 1 | 140583 | 140584 | 0.00333333 | 4002.260619 | 4002.260622 |
| 4002.260669 | Kernel\_Read\|xilinx:adm-pcie-7v3:1ddr:3.0-0\|cnn\_GOOD | Read | OCL Region | 1 | 1 | 140598 | 140599 | 0.00333333 | 4002.260669 | 4002.260672 |
| 4002.260719 | Kernel\_Read\|xilinx:adm-pcie-7v3:1ddr:3.0-0\|cnn\_GOOD | Read | OCL Region | 1 | 1 | 140613 | 140614 | 0.00333333 | 4002.260719 | 4002.260722 |
| 4002.260769 | Kernel\_Read\|xilinx:adm-pcie-7v3:1ddr:3.0-0\|cnn\_GOOD | Read | OCL Region | 1 | 1 | 140628 | 140629 | 0.00333333 | 4002.260769 | 4002.260772 |
| 4002.260819 | Kernel\_Read\|xilinx:adm-pcie-7v3:1ddr:3.0-0\|cnn\_GOOD | Read | OCL Region | 1 | 1 | 140643 | 140644 | 0.00333333 | 4002.260819 | 4002.260822 |
| 4002.260869 | Kernel\_Read\|xilinx:adm-pcie-7v3:1ddr:3.0-0\|cnn\_GOOD | Read | OCL Region | 1 | 1 | 140658 | 140659 | 0.00333333 | 4002.260869 | 4002.260872 |
| 4002.260919 | Kernel\_Read\|xilinx:adm-pcie-7v3:1ddr:3.0-0\|cnn\_GOOD | Read | OCL Region | 1 | 1 | 140673 | 140674 | 0.00333333 | 4002.260919 | 4002.260922 |
| 4002.260969 | Kernel\_Read\|xilinx:adm-pcie-7v3:1ddr:3.0-0\|cnn\_GOOD | Read | OCL Region | 1 | 1 | 140688 | 140689 | 0.00333333 | 4002.260969 | 4002.260972 |
| 4002.261019 | Kernel\_Read\|xilinx:adm-pcie-7v3:1ddr:3.0-0\|cnn\_GOOD | Read | OCL Region | 1 | 1 | 140703 | 140704 | 0.00333333 | 4002.261019 | 4002.261022 |
| 4002.261069 | Kernel\_Read\|xilinx:adm-pcie-7v3:1ddr:3.0-0\|cnn\_GOOD | Read | OCL Region | 1 | 1 | 140718 | 140719 | 0.00333333 | 4002.261069 | 4002.261072 |
| 4002.261132 | Kernel\_Read\|xilinx:adm-pcie-7v3:1ddr:3.0-0\|cnn\_GOOD | Read | OCL Region | 1 | 1 | 140737 | 140738 | 0.00333333 | 4002.261132 | 4002.261136 |
| 4002.261179 | Kernel\_Read\|xilinx:adm-pcie-7v3:1ddr:3.0-0\|cnn\_GOOD | Read | OCL Region | 1 | 1 | 140751 | 140752 | 0.00333333 | 4002.261179 | 4002.261182 |
| 4002.261229 | Kernel\_Read\|xilinx:adm-pcie-7v3:1ddr:3.0-0\|cnn\_GOOD | Read | OCL Region | 1 | 1 | 140766 | 140767 | 0.00333333 | 4002.261229 | 4002.261232 |
| 4002.261282 | Kernel\_Read\|xilinx:adm-pcie-7v3:1ddr:3.0-0\|cnn\_GOOD | Read | OCL Region | 1 | 1 | 140782 | 140783 | 0.00333333 | 4002.261282 | 4002.261286 |
| 4002.261332 | Kernel\_Read\|xilinx:adm-pcie-7v3:1ddr:3.0-0\|cnn\_GOOD | Read | OCL Region | 1 | 1 | 140797 | 140798 | 0.00333333 | 4002.261332 | 4002.261336 |
| 4002.261382 | Kernel\_Read\|xilinx:adm-pcie-7v3:1ddr:3.0-0\|cnn\_GOOD | Read | OCL Region | 1 | 1 | 140812 | 140813 | 0.00333333 | 4002.261382 | 4002.261386 |
| 4002.261429 | Kernel\_Read\|xilinx:adm-pcie-7v3:1ddr:3.0-0\|cnn\_GOOD | Read | OCL Region | 1 | 1 | 140826 | 140827 | 0.00333333 | 4002.261429 | 4002.261432 |
| 4002.261482 | Kernel\_Read\|xilinx:adm-pcie-7v3:1ddr:3.0-0\|cnn\_GOOD | Read | OCL Region | 1 | 1 | 140842 | 140843 | 0.00333333 | 4002.261482 | 4002.261486 |
| 4002.261532 | Kernel\_Read\|xilinx:adm-pcie-7v3:1ddr:3.0-0\|cnn\_GOOD | Read | OCL Region | 1 | 1 | 140857 | 140858 | 0.00333333 | 4002.261532 | 4002.261536 |
| 4002.261579 | Kernel\_Read\|xilinx:adm-pcie-7v3:1ddr:3.0-0\|cnn\_GOOD | Read | OCL Region | 1 | 1 | 140871 | 140872 | 0.00333333 | 4002.261579 | 4002.261582 |
| 4002.261629 | Kernel\_Read\|xilinx:adm-pcie-7v3:1ddr:3.0-0\|cnn\_GOOD | Read | OCL Region | 1 | 1 | 140886 | 140887 | 0.00333333 | 4002.261629 | 4002.261632 |
| 4002.261682 | Kernel\_Read\|xilinx:adm-pcie-7v3:1ddr:3.0-0\|cnn\_GOOD | Read | OCL Region | 1 | 1 | 140902 | 140903 | 0.00333333 | 4002.261682 | 4002.261686 |
| 4002.261732 | Kernel\_Read\|xilinx:adm-pcie-7v3:1ddr:3.0-0\|cnn\_GOOD | Read | OCL Region | 1 | 1 | 140917 | 140918 | 0.00333333 | 4002.261732 | 4002.261736 |
| 4002.261782 | Kernel\_Read\|xilinx:adm-pcie-7v3:1ddr:3.0-0\|cnn\_GOOD | Read | OCL Region | 1 | 1 | 140932 | 140933 | 0.00333333 | 4002.261782 | 4002.261786 |
| 4002.261829 | Kernel\_Read\|xilinx:adm-pcie-7v3:1ddr:3.0-0\|cnn\_GOOD | Read | OCL Region | 1 | 1 | 140946 | 140947 | 0.00333333 | 4002.261829 | 4002.261832 |
| 4002.261882 | Kernel\_Read\|xilinx:adm-pcie-7v3:1ddr:3.0-0\|cnn\_GOOD | Read | OCL Region | 1 | 1 | 140962 | 140963 | 0.00333333 | 4002.261882 | 4002.261886 |
| 4002.261932 | Kernel\_Read\|xilinx:adm-pcie-7v3:1ddr:3.0-0\|cnn\_GOOD | Read | OCL Region | 1 | 1 | 140977 | 140978 | 0.00333333 | 4002.261932 | 4002.261936 |
| 4002.261979 | Kernel\_Read\|xilinx:adm-pcie-7v3:1ddr:3.0-0\|cnn\_GOOD | Read | OCL Region | 1 | 1 | 140991 | 140992 | 0.00333333 | 4002.261979 | 4002.261982 |
| 4002.262029 | Kernel\_Read\|xilinx:adm-pcie-7v3:1ddr:3.0-0\|cnn\_GOOD | Read | OCL Region | 1 | 1 | 141006 | 141007 | 0.00333333 | 4002.262029 | 4002.262032 |
| 4002.262092 | Kernel\_Read\|xilinx:adm-pcie-7v3:1ddr:3.0-0\|cnn\_GOOD | Read | OCL Region | 1 | 1 | 141025 | 141026 | 0.00333333 | 4002.262092 | 4002.262096 |
| 4002.262142 | Kernel\_Read\|xilinx:adm-pcie-7v3:1ddr:3.0-0\|cnn\_GOOD | Read | OCL Region | 1 | 1 | 141040 | 141041 | 0.00333333 | 4002.262142 | 4002.262146 |
| 4002.262192 | Kernel\_Read\|xilinx:adm-pcie-7v3:1ddr:3.0-0\|cnn\_GOOD | Read | OCL Region | 1 | 1 | 141055 | 141056 | 0.00333333 | 4002.262192 | 4002.262196 |
| 4002.262242 | Kernel\_Read\|xilinx:adm-pcie-7v3:1ddr:3.0-0\|cnn\_GOOD | Read | OCL Region | 1 | 1 | 141070 | 141071 | 0.00333333 | 4002.262242 | 4002.262246 |
| 4002.262292 | Kernel\_Read\|xilinx:adm-pcie-7v3:1ddr:3.0-0\|cnn\_GOOD | Read | OCL Region | 1 | 1 | 141085 | 141086 | 0.00333333 | 4002.262292 | 4002.262296 |
| 4002.262342 | Kernel\_Read\|xilinx:adm-pcie-7v3:1ddr:3.0-0\|cnn\_GOOD | Read | OCL Region | 1 | 1 | 141100 | 141101 | 0.00333333 | 4002.262342 | 4002.262346 |
| 4002.262392 | Kernel\_Read\|xilinx:adm-pcie-7v3:1ddr:3.0-0\|cnn\_GOOD | Read | OCL Region | 1 | 1 | 141115 | 141116 | 0.00333333 | 4002.262392 | 4002.262396 |
| 4002.262442 | Kernel\_Read\|xilinx:adm-pcie-7v3:1ddr:3.0-0\|cnn\_GOOD | Read | OCL Region | 1 | 1 | 141130 | 141131 | 0.00333333 | 4002.262442 | 4002.262446 |
| 4002.262492 | Kernel\_Read\|xilinx:adm-pcie-7v3:1ddr:3.0-0\|cnn\_GOOD | Read | OCL Region | 1 | 1 | 141145 | 141146 | 0.00333333 | 4002.262492 | 4002.262496 |
| 4002.262542 | Kernel\_Read\|xilinx:adm-pcie-7v3:1ddr:3.0-0\|cnn\_GOOD | Read | OCL Region | 1 | 1 | 141160 | 141161 | 0.00333333 | 4002.262542 | 4002.262546 |
| 4002.262592 | Kernel\_Read\|xilinx:adm-pcie-7v3:1ddr:3.0-0\|cnn\_GOOD | Read | OCL Region | 1 | 1 | 141175 | 141176 | 0.00333333 | 4002.262592 | 4002.262596 |
| 4002.262642 | Kernel\_Read\|xilinx:adm-pcie-7v3:1ddr:3.0-0\|cnn\_GOOD | Read | OCL Region | 1 | 1 | 141190 | 141191 | 0.00333333 | 4002.262642 | 4002.262646 |
| 4002.262692 | Kernel\_Read\|xilinx:adm-pcie-7v3:1ddr:3.0-0\|cnn\_GOOD | Read | OCL Region | 1 | 1 | 141205 | 141206 | 0.00333333 | 4002.262692 | 4002.262696 |
| 4002.262742 | Kernel\_Read\|xilinx:adm-pcie-7v3:1ddr:3.0-0\|cnn\_GOOD | Read | OCL Region | 1 | 1 | 141220 | 141221 | 0.00333333 | 4002.262742 | 4002.262746 |
| 4002.262792 | Kernel\_Read\|xilinx:adm-pcie-7v3:1ddr:3.0-0\|cnn\_GOOD | Read | OCL Region | 1 | 1 | 141235 | 141236 | 0.00333333 | 4002.262792 | 4002.262796 |
| 4002.262842 | Kernel\_Read\|xilinx:adm-pcie-7v3:1ddr:3.0-0\|cnn\_GOOD | Read | OCL Region | 1 | 1 | 141250 | 141251 | 0.00333333 | 4002.262842 | 4002.262846 |
| 4002.262892 | Kernel\_Read\|xilinx:adm-pcie-7v3:1ddr:3.0-0\|cnn\_GOOD | Read | OCL Region | 1 | 1 | 141265 | 141266 | 0.00333333 | 4002.262892 | 4002.262896 |
| 4002.262942 | Kernel\_Read\|xilinx:adm-pcie-7v3:1ddr:3.0-0\|cnn\_GOOD | Read | OCL Region | 1 | 1 | 141280 | 141281 | 0.00333333 | 4002.262942 | 4002.262946 |
| 4002.262992 | Kernel\_Read\|xilinx:adm-pcie-7v3:1ddr:3.0-0\|cnn\_GOOD | Read | OCL Region | 1 | 1 | 141295 | 141296 | 0.00333333 | 4002.262992 | 4002.262996 |
| 4002.263042 | Kernel\_Read\|xilinx:adm-pcie-7v3:1ddr:3.0-0\|cnn\_GOOD | Read | OCL Region | 1 | 1 | 141310 | 141311 | 0.00333333 | 4002.263042 | 4002.263046 |
| 4002.263319 | Kernel\_Read\|xilinx:adm-pcie-7v3:1ddr:3.0-0\|cnn\_GOOD | Read | OCL Region | 1 | 1 | 141393 | 141394 | 0.00333333 | 4002.263319 | 4002.263322 |
| 4002.263369 | Kernel\_Read\|xilinx:adm-pcie-7v3:1ddr:3.0-0\|cnn\_GOOD | Read | OCL Region | 1 | 1 | 141408 | 141409 | 0.00333333 | 4002.263369 | 4002.263372 |
| 4002.263426 | Kernel\_Read\|xilinx:adm-pcie-7v3:1ddr:3.0-0\|cnn\_GOOD | Read | OCL Region | 1 | 1 | 141425 | 141426 | 0.00333333 | 4002.263426 | 4002.263429 |
| 4002.263476 | Kernel\_Read\|xilinx:adm-pcie-7v3:1ddr:3.0-0\|cnn\_GOOD | Read | OCL Region | 1 | 1 | 141440 | 141441 | 0.00333333 | 4002.263476 | 4002.263479 |
| 4002.263526 | Kernel\_Read\|xilinx:adm-pcie-7v3:1ddr:3.0-0\|cnn\_GOOD | Read | OCL Region | 1 | 1 | 141455 | 141456 | 0.00333333 | 4002.263526 | 4002.263529 |
| 4002.263576 | Kernel\_Read\|xilinx:adm-pcie-7v3:1ddr:3.0-0\|cnn\_GOOD | Read | OCL Region | 1 | 1 | 141470 | 141471 | 0.00333333 | 4002.263576 | 4002.263579 |
| 4002.263626 | Kernel\_Read\|xilinx:adm-pcie-7v3:1ddr:3.0-0\|cnn\_GOOD | Read | OCL Region | 1 | 1 | 141485 | 141486 | 0.00333333 | 4002.263626 | 4002.263629 |
| 4002.263676 | Kernel\_Read\|xilinx:adm-pcie-7v3:1ddr:3.0-0\|cnn\_GOOD | Read | OCL Region | 1 | 1 | 141500 | 141501 | 0.00333333 | 4002.263676 | 4002.263679 |
| 4002.263726 | Kernel\_Read\|xilinx:adm-pcie-7v3:1ddr:3.0-0\|cnn\_GOOD | Read | OCL Region | 1 | 1 | 141515 | 141516 | 0.00333333 | 4002.263726 | 4002.263729 |
| 4002.263776 | Kernel\_Read\|xilinx:adm-pcie-7v3:1ddr:3.0-0\|cnn\_GOOD | Read | OCL Region | 1 | 1 | 141530 | 141531 | 0.00333333 | 4002.263776 | 4002.263779 |
| 4002.263826 | Kernel\_Read\|xilinx:adm-pcie-7v3:1ddr:3.0-0\|cnn\_GOOD | Read | OCL Region | 1 | 1 | 141545 | 141546 | 0.00333333 | 4002.263826 | 4002.263829 |
| 4002.263876 | Kernel\_Read\|xilinx:adm-pcie-7v3:1ddr:3.0-0\|cnn\_GOOD | Read | OCL Region | 1 | 1 | 141560 | 141561 | 0.00333333 | 4002.263876 | 4002.263879 |
| 4002.263926 | Kernel\_Read\|xilinx:adm-pcie-7v3:1ddr:3.0-0\|cnn\_GOOD | Read | OCL Region | 1 | 1 | 141575 | 141576 | 0.00333333 | 4002.263926 | 4002.263929 |
| 4002.263976 | Kernel\_Read\|xilinx:adm-pcie-7v3:1ddr:3.0-0\|cnn\_GOOD | Read | OCL Region | 1 | 1 | 141590 | 141591 | 0.00333333 | 4002.263976 | 4002.263979 |
| 4002.264026 | Kernel\_Read\|xilinx:adm-pcie-7v3:1ddr:3.0-0\|cnn\_GOOD | Read | OCL Region | 1 | 1 | 141605 | 141606 | 0.00333333 | 4002.264026 | 4002.264029 |
| 4002.264076 | Kernel\_Read\|xilinx:adm-pcie-7v3:1ddr:3.0-0\|cnn\_GOOD | Read | OCL Region | 1 | 1 | 141620 | 141621 | 0.00333333 | 4002.264076 | 4002.264079 |
| 4002.264126 | Kernel\_Read\|xilinx:adm-pcie-7v3:1ddr:3.0-0\|cnn\_GOOD | Read | OCL Region | 1 | 1 | 141635 | 141636 | 0.00333333 | 4002.264126 | 4002.264129 |
| 4002.264176 | Kernel\_Read\|xilinx:adm-pcie-7v3:1ddr:3.0-0\|cnn\_GOOD | Read | OCL Region | 1 | 1 | 141650 | 141651 | 0.00333333 | 4002.264176 | 4002.264179 |
| 4002.264236 | Kernel\_Read\|xilinx:adm-pcie-7v3:1ddr:3.0-0\|cnn\_GOOD | Read | OCL Region | 1 | 1 | 141668 | 141669 | 0.00333333 | 4002.264236 | 4002.264239 |
| 4002.264286 | Kernel\_Read\|xilinx:adm-pcie-7v3:1ddr:3.0-0\|cnn\_GOOD | Read | OCL Region | 1 | 1 | 141683 | 141684 | 0.00333333 | 4002.264286 | 4002.264289 |
| 4002.264336 | Kernel\_Read\|xilinx:adm-pcie-7v3:1ddr:3.0-0\|cnn\_GOOD | Read | OCL Region | 1 | 1 | 141698 | 141699 | 0.00333333 | 4002.264336 | 4002.264339 |
| 4002.264386 | Kernel\_Read\|xilinx:adm-pcie-7v3:1ddr:3.0-0\|cnn\_GOOD | Read | OCL Region | 1 | 1 | 141713 | 141714 | 0.00333333 | 4002.264386 | 4002.264389 |
| 4002.264436 | Kernel\_Read\|xilinx:adm-pcie-7v3:1ddr:3.0-0\|cnn\_GOOD | Read | OCL Region | 1 | 1 | 141728 | 141729 | 0.00333333 | 4002.264436 | 4002.264439 |
| 4002.264486 | Kernel\_Read\|xilinx:adm-pcie-7v3:1ddr:3.0-0\|cnn\_GOOD | Read | OCL Region | 1 | 1 | 141743 | 141744 | 0.00333333 | 4002.264486 | 4002.264489 |
| 4002.264536 | Kernel\_Read\|xilinx:adm-pcie-7v3:1ddr:3.0-0\|cnn\_GOOD | Read | OCL Region | 1 | 1 | 141758 | 141759 | 0.00333333 | 4002.264536 | 4002.264539 |
| 4002.264586 | Kernel\_Read\|xilinx:adm-pcie-7v3:1ddr:3.0-0\|cnn\_GOOD | Read | OCL Region | 1 | 1 | 141773 | 141774 | 0.00333333 | 4002.264586 | 4002.264589 |
| 4002.264636 | Kernel\_Read\|xilinx:adm-pcie-7v3:1ddr:3.0-0\|cnn\_GOOD | Read | OCL Region | 1 | 1 | 141788 | 141789 | 0.00333333 | 4002.264636 | 4002.264639 |
| 4002.264686 | Kernel\_Read\|xilinx:adm-pcie-7v3:1ddr:3.0-0\|cnn\_GOOD | Read | OCL Region | 1 | 1 | 141803 | 141804 | 0.00333333 | 4002.264686 | 4002.264689 |
| 4002.264736 | Kernel\_Read\|xilinx:adm-pcie-7v3:1ddr:3.0-0\|cnn\_GOOD | Read | OCL Region | 1 | 1 | 141818 | 141819 | 0.00333333 | 4002.264736 | 4002.264739 |
| 4002.264786 | Kernel\_Read\|xilinx:adm-pcie-7v3:1ddr:3.0-0\|cnn\_GOOD | Read | OCL Region | 1 | 1 | 141833 | 141834 | 0.00333333 | 4002.264786 | 4002.264789 |
| 4002.264836 | Kernel\_Read\|xilinx:adm-pcie-7v3:1ddr:3.0-0\|cnn\_GOOD | Read | OCL Region | 1 | 1 | 141848 | 141849 | 0.00333333 | 4002.264836 | 4002.264839 |
| 4002.264886 | Kernel\_Read\|xilinx:adm-pcie-7v3:1ddr:3.0-0\|cnn\_GOOD | Read | OCL Region | 1 | 1 | 141863 | 141864 | 0.00333333 | 4002.264886 | 4002.264889 |
| 4002.264936 | Kernel\_Read\|xilinx:adm-pcie-7v3:1ddr:3.0-0\|cnn\_GOOD | Read | OCL Region | 1 | 1 | 141878 | 141879 | 0.00333333 | 4002.264936 | 4002.264939 |
| 4002.264986 | Kernel\_Read\|xilinx:adm-pcie-7v3:1ddr:3.0-0\|cnn\_GOOD | Read | OCL Region | 1 | 1 | 141893 | 141894 | 0.00333333 | 4002.264986 | 4002.264989 |
| 4002.265036 | Kernel\_Read\|xilinx:adm-pcie-7v3:1ddr:3.0-0\|cnn\_GOOD | Read | OCL Region | 1 | 1 | 141908 | 141909 | 0.00333333 | 4002.265036 | 4002.265039 |
| 4002.265086 | Kernel\_Read\|xilinx:adm-pcie-7v3:1ddr:3.0-0\|cnn\_GOOD | Read | OCL Region | 1 | 1 | 141923 | 141924 | 0.00333333 | 4002.265086 | 4002.265089 |
| 4002.265136 | Kernel\_Read\|xilinx:adm-pcie-7v3:1ddr:3.0-0\|cnn\_GOOD | Read | OCL Region | 1 | 1 | 141938 | 141939 | 0.00333333 | 4002.265136 | 4002.265139 |
| 4002.265186 | Kernel\_Read\|xilinx:adm-pcie-7v3:1ddr:3.0-0\|cnn\_GOOD | Read | OCL Region | 1 | 1 | 141953 | 141954 | 0.00333333 | 4002.265186 | 4002.265189 |
| 4002.265246 | Kernel\_Read\|xilinx:adm-pcie-7v3:1ddr:3.0-0\|cnn\_GOOD | Read | OCL Region | 1 | 1 | 141971 | 141972 | 0.00333333 | 4002.265246 | 4002.265249 |
| 4002.265296 | Kernel\_Read\|xilinx:adm-pcie-7v3:1ddr:3.0-0\|cnn\_GOOD | Read | OCL Region | 1 | 1 | 141986 | 141987 | 0.00333333 | 4002.265296 | 4002.265299 |
| 4002.265346 | Kernel\_Read\|xilinx:adm-pcie-7v3:1ddr:3.0-0\|cnn\_GOOD | Read | OCL Region | 1 | 1 | 142001 | 142002 | 0.00333333 | 4002.265346 | 4002.265349 |
| 4002.265396 | Kernel\_Read\|xilinx:adm-pcie-7v3:1ddr:3.0-0\|cnn\_GOOD | Read | OCL Region | 1 | 1 | 142016 | 142017 | 0.00333333 | 4002.265396 | 4002.265399 |
| 4002.265446 | Kernel\_Read\|xilinx:adm-pcie-7v3:1ddr:3.0-0\|cnn\_GOOD | Read | OCL Region | 1 | 1 | 142031 | 142032 | 0.00333333 | 4002.265446 | 4002.265449 |
| 4002.265496 | Kernel\_Read\|xilinx:adm-pcie-7v3:1ddr:3.0-0\|cnn\_GOOD | Read | OCL Region | 1 | 1 | 142046 | 142047 | 0.00333333 | 4002.265496 | 4002.265499 |
| 4002.265546 | Kernel\_Read\|xilinx:adm-pcie-7v3:1ddr:3.0-0\|cnn\_GOOD | Read | OCL Region | 1 | 1 | 142061 | 142062 | 0.00333333 | 4002.265546 | 4002.265549 |
| 4002.265596 | Kernel\_Read\|xilinx:adm-pcie-7v3:1ddr:3.0-0\|cnn\_GOOD | Read | OCL Region | 1 | 1 | 142076 | 142077 | 0.00333333 | 4002.265596 | 4002.265599 |
| 4002.265646 | Kernel\_Read\|xilinx:adm-pcie-7v3:1ddr:3.0-0\|cnn\_GOOD | Read | OCL Region | 1 | 1 | 142091 | 142092 | 0.00333333 | 4002.265646 | 4002.265649 |
| 4002.265696 | Kernel\_Read\|xilinx:adm-pcie-7v3:1ddr:3.0-0\|cnn\_GOOD | Read | OCL Region | 1 | 1 | 142106 | 142107 | 0.00333333 | 4002.265696 | 4002.265699 |
| 4002.265746 | Kernel\_Read\|xilinx:adm-pcie-7v3:1ddr:3.0-0\|cnn\_GOOD | Read | OCL Region | 1 | 1 | 142121 | 142122 | 0.00333333 | 4002.265746 | 4002.265749 |
| 4002.265796 | Kernel\_Read\|xilinx:adm-pcie-7v3:1ddr:3.0-0\|cnn\_GOOD | Read | OCL Region | 1 | 1 | 142136 | 142137 | 0.00333333 | 4002.265796 | 4002.265799 |
| 4002.265846 | Kernel\_Read\|xilinx:adm-pcie-7v3:1ddr:3.0-0\|cnn\_GOOD | Read | OCL Region | 1 | 1 | 142151 | 142152 | 0.00333333 | 4002.265846 | 4002.265849 |
| 4002.265896 | Kernel\_Read\|xilinx:adm-pcie-7v3:1ddr:3.0-0\|cnn\_GOOD | Read | OCL Region | 1 | 1 | 142166 | 142167 | 0.00333333 | 4002.265896 | 4002.265899 |
| 4002.265946 | Kernel\_Read\|xilinx:adm-pcie-7v3:1ddr:3.0-0\|cnn\_GOOD | Read | OCL Region | 1 | 1 | 142181 | 142182 | 0.00333333 | 4002.265946 | 4002.265949 |
| 4002.265996 | Kernel\_Read\|xilinx:adm-pcie-7v3:1ddr:3.0-0\|cnn\_GOOD | Read | OCL Region | 1 | 1 | 142196 | 142197 | 0.00333333 | 4002.265996 | 4002.265999 |
| 4002.266046 | Kernel\_Read\|xilinx:adm-pcie-7v3:1ddr:3.0-0\|cnn\_GOOD | Read | OCL Region | 1 | 1 | 142211 | 142212 | 0.00333333 | 4002.266046 | 4002.266049 |
| 4002.266096 | Kernel\_Read\|xilinx:adm-pcie-7v3:1ddr:3.0-0\|cnn\_GOOD | Read | OCL Region | 1 | 1 | 142226 | 142227 | 0.00333333 | 4002.266096 | 4002.266099 |
| 4002.266146 | Kernel\_Read\|xilinx:adm-pcie-7v3:1ddr:3.0-0\|cnn\_GOOD | Read | OCL Region | 1 | 1 | 142241 | 142242 | 0.00333333 | 4002.266146 | 4002.266149 |
| 4002.266202 | Kernel\_Read\|xilinx:adm-pcie-7v3:1ddr:3.0-0\|cnn\_GOOD | Read | OCL Region | 1 | 1 | 142258 | 142259 | 0.00333333 | 4002.266202 | 4002.266206 |
| 4002.266252 | Kernel\_Read\|xilinx:adm-pcie-7v3:1ddr:3.0-0\|cnn\_GOOD | Read | OCL Region | 1 | 1 | 142273 | 142274 | 0.00333333 | 4002.266252 | 4002.266256 |
| 4002.266302 | Kernel\_Read\|xilinx:adm-pcie-7v3:1ddr:3.0-0\|cnn\_GOOD | Read | OCL Region | 1 | 1 | 142288 | 142289 | 0.00333333 | 4002.266302 | 4002.266306 |
| 4002.266359 | Kernel\_Read\|xilinx:adm-pcie-7v3:1ddr:3.0-0\|cnn\_GOOD | Read | OCL Region | 1 | 1 | 142305 | 142306 | 0.00333333 | 4002.266359 | 4002.266362 |
| 4002.266409 | Kernel\_Read\|xilinx:adm-pcie-7v3:1ddr:3.0-0\|cnn\_GOOD | Read | OCL Region | 1 | 1 | 142320 | 142321 | 0.00333333 | 4002.266409 | 4002.266412 |
| 4002.266459 | Kernel\_Read\|xilinx:adm-pcie-7v3:1ddr:3.0-0\|cnn\_GOOD | Read | OCL Region | 1 | 1 | 142335 | 142336 | 0.00333333 | 4002.266459 | 4002.266462 |
| 4002.266502 | Kernel\_Read\|xilinx:adm-pcie-7v3:1ddr:3.0-0\|cnn\_GOOD | Read | OCL Region | 1 | 1 | 142348 | 142349 | 0.00333333 | 4002.266502 | 4002.266506 |
| 4002.266559 | Kernel\_Read\|xilinx:adm-pcie-7v3:1ddr:3.0-0\|cnn\_GOOD | Read | OCL Region | 1 | 1 | 142365 | 142366 | 0.00333333 | 4002.266559 | 4002.266562 |
| 4002.266602 | Kernel\_Read\|xilinx:adm-pcie-7v3:1ddr:3.0-0\|cnn\_GOOD | Read | OCL Region | 1 | 1 | 142378 | 142379 | 0.00333333 | 4002.266602 | 4002.266606 |
| 4002.266652 | Kernel\_Read\|xilinx:adm-pcie-7v3:1ddr:3.0-0\|cnn\_GOOD | Read | OCL Region | 1 | 1 | 142393 | 142394 | 0.00333333 | 4002.266652 | 4002.266656 |
| 4002.266702 | Kernel\_Read\|xilinx:adm-pcie-7v3:1ddr:3.0-0\|cnn\_GOOD | Read | OCL Region | 1 | 1 | 142408 | 142409 | 0.00333333 | 4002.266702 | 4002.266706 |
| 4002.266759 | Kernel\_Read\|xilinx:adm-pcie-7v3:1ddr:3.0-0\|cnn\_GOOD | Read | OCL Region | 1 | 1 | 142425 | 142426 | 0.00333333 | 4002.266759 | 4002.266762 |
| 4002.266809 | Kernel\_Read\|xilinx:adm-pcie-7v3:1ddr:3.0-0\|cnn\_GOOD | Read | OCL Region | 1 | 1 | 142440 | 142441 | 0.00333333 | 4002.266809 | 4002.266812 |
| 4002.266859 | Kernel\_Read\|xilinx:adm-pcie-7v3:1ddr:3.0-0\|cnn\_GOOD | Read | OCL Region | 1 | 1 | 142455 | 142456 | 0.00333333 | 4002.266859 | 4002.266862 |
| 4002.266902 | Kernel\_Read\|xilinx:adm-pcie-7v3:1ddr:3.0-0\|cnn\_GOOD | Read | OCL Region | 1 | 1 | 142468 | 142469 | 0.00333333 | 4002.266902 | 4002.266906 |
| 4002.266959 | Kernel\_Read\|xilinx:adm-pcie-7v3:1ddr:3.0-0\|cnn\_GOOD | Read | OCL Region | 1 | 1 | 142485 | 142486 | 0.00333333 | 4002.266959 | 4002.266962 |
| 4002.267009 | Kernel\_Read\|xilinx:adm-pcie-7v3:1ddr:3.0-0\|cnn\_GOOD | Read | OCL Region | 1 | 1 | 142500 | 142501 | 0.00333333 | 4002.267009 | 4002.267012 |
| 4002.267052 | Kernel\_Read\|xilinx:adm-pcie-7v3:1ddr:3.0-0\|cnn\_GOOD | Read | OCL Region | 1 | 1 | 142513 | 142514 | 0.00333333 | 4002.267052 | 4002.267056 |
| 4002.267102 | Kernel\_Read\|xilinx:adm-pcie-7v3:1ddr:3.0-0\|cnn\_GOOD | Read | OCL Region | 1 | 1 | 142528 | 142529 | 0.00333333 | 4002.267102 | 4002.267106 |
| 4002.267159 | Kernel\_Read\|xilinx:adm-pcie-7v3:1ddr:3.0-0\|cnn\_GOOD | Read | OCL Region | 1 | 1 | 142545 | 142546 | 0.00333333 | 4002.267159 | 4002.267162 |
| 4002.267216 | Kernel\_Read\|xilinx:adm-pcie-7v3:1ddr:3.0-0\|cnn\_GOOD | Read | OCL Region | 1 | 1 | 142562 | 142563 | 0.00333333 | 4002.267216 | 4002.267219 |
| 4002.267266 | Kernel\_Read\|xilinx:adm-pcie-7v3:1ddr:3.0-0\|cnn\_GOOD | Read | OCL Region | 1 | 1 | 142577 | 142578 | 0.00333333 | 4002.267266 | 4002.267269 |
| 4002.267316 | Kernel\_Read\|xilinx:adm-pcie-7v3:1ddr:3.0-0\|cnn\_GOOD | Read | OCL Region | 1 | 1 | 142592 | 142593 | 0.00333333 | 4002.267316 | 4002.267319 |
| 4002.267366 | Kernel\_Read\|xilinx:adm-pcie-7v3:1ddr:3.0-0\|cnn\_GOOD | Read | OCL Region | 1 | 1 | 142607 | 142608 | 0.00333333 | 4002.267366 | 4002.267369 |
| 4002.267416 | Kernel\_Read\|xilinx:adm-pcie-7v3:1ddr:3.0-0\|cnn\_GOOD | Read | OCL Region | 1 | 1 | 142622 | 142623 | 0.00333333 | 4002.267416 | 4002.267419 |
| 4002.267466 | Kernel\_Read\|xilinx:adm-pcie-7v3:1ddr:3.0-0\|cnn\_GOOD | Read | OCL Region | 1 | 1 | 142637 | 142638 | 0.00333333 | 4002.267466 | 4002.267469 |
| 4002.267516 | Kernel\_Read\|xilinx:adm-pcie-7v3:1ddr:3.0-0\|cnn\_GOOD | Read | OCL Region | 1 | 1 | 142652 | 142653 | 0.00333333 | 4002.267516 | 4002.267519 |
| 4002.267566 | Kernel\_Read\|xilinx:adm-pcie-7v3:1ddr:3.0-0\|cnn\_GOOD | Read | OCL Region | 1 | 1 | 142667 | 142668 | 0.00333333 | 4002.267566 | 4002.267569 |
| 4002.267616 | Kernel\_Read\|xilinx:adm-pcie-7v3:1ddr:3.0-0\|cnn\_GOOD | Read | OCL Region | 1 | 1 | 142682 | 142683 | 0.00333333 | 4002.267616 | 4002.267619 |
| 4002.267666 | Kernel\_Read\|xilinx:adm-pcie-7v3:1ddr:3.0-0\|cnn\_GOOD | Read | OCL Region | 1 | 1 | 142697 | 142698 | 0.00333333 | 4002.267666 | 4002.267669 |
| 4002.267716 | Kernel\_Read\|xilinx:adm-pcie-7v3:1ddr:3.0-0\|cnn\_GOOD | Read | OCL Region | 1 | 1 | 142712 | 142713 | 0.00333333 | 4002.267716 | 4002.267719 |
| 4002.267766 | Kernel\_Read\|xilinx:adm-pcie-7v3:1ddr:3.0-0\|cnn\_GOOD | Read | OCL Region | 1 | 1 | 142727 | 142728 | 0.00333333 | 4002.267766 | 4002.267769 |
| 4002.267816 | Kernel\_Read\|xilinx:adm-pcie-7v3:1ddr:3.0-0\|cnn\_GOOD | Read | OCL Region | 1 | 1 | 142742 | 142743 | 0.00333333 | 4002.267816 | 4002.267819 |
| 4002.267866 | Kernel\_Read\|xilinx:adm-pcie-7v3:1ddr:3.0-0\|cnn\_GOOD | Read | OCL Region | 1 | 1 | 142757 | 142758 | 0.00333333 | 4002.267866 | 4002.267869 |
| 4002.267916 | Kernel\_Read\|xilinx:adm-pcie-7v3:1ddr:3.0-0\|cnn\_GOOD | Read | OCL Region | 1 | 1 | 142772 | 142773 | 0.00333333 | 4002.267916 | 4002.267919 |
| 4002.267966 | Kernel\_Read\|xilinx:adm-pcie-7v3:1ddr:3.0-0\|cnn\_GOOD | Read | OCL Region | 1 | 1 | 142787 | 142788 | 0.00333333 | 4002.267966 | 4002.267969 |
| 4002.268016 | Kernel\_Read\|xilinx:adm-pcie-7v3:1ddr:3.0-0\|cnn\_GOOD | Read | OCL Region | 1 | 1 | 142802 | 142803 | 0.00333333 | 4002.268016 | 4002.268019 |
| 4002.268066 | Kernel\_Read\|xilinx:adm-pcie-7v3:1ddr:3.0-0\|cnn\_GOOD | Read | OCL Region | 1 | 1 | 142817 | 142818 | 0.00333333 | 4002.268066 | 4002.268069 |
| 4002.268116 | Kernel\_Read\|xilinx:adm-pcie-7v3:1ddr:3.0-0\|cnn\_GOOD | Read | OCL Region | 1 | 1 | 142832 | 142833 | 0.00333333 | 4002.268116 | 4002.268119 |
| 4002.268166 | Kernel\_Read\|xilinx:adm-pcie-7v3:1ddr:3.0-0\|cnn\_GOOD | Read | OCL Region | 1 | 1 | 142847 | 142848 | 0.00333333 | 4002.268166 | 4002.268169 |
| 4002.268226 | Kernel\_Read\|xilinx:adm-pcie-7v3:1ddr:3.0-0\|cnn\_GOOD | Read | OCL Region | 1 | 1 | 142865 | 142866 | 0.00333333 | 4002.268226 | 4002.268229 |
| 4002.268276 | Kernel\_Read\|xilinx:adm-pcie-7v3:1ddr:3.0-0\|cnn\_GOOD | Read | OCL Region | 1 | 1 | 142880 | 142881 | 0.00333333 | 4002.268276 | 4002.268279 |
| 4002.268326 | Kernel\_Read\|xilinx:adm-pcie-7v3:1ddr:3.0-0\|cnn\_GOOD | Read | OCL Region | 1 | 1 | 142895 | 142896 | 0.00333333 | 4002.268326 | 4002.268329 |
| 4002.268376 | Kernel\_Read\|xilinx:adm-pcie-7v3:1ddr:3.0-0\|cnn\_GOOD | Read | OCL Region | 1 | 1 | 142910 | 142911 | 0.00333333 | 4002.268376 | 4002.268379 |
| 4002.268426 | Kernel\_Read\|xilinx:adm-pcie-7v3:1ddr:3.0-0\|cnn\_GOOD | Read | OCL Region | 1 | 1 | 142925 | 142926 | 0.00333333 | 4002.268426 | 4002.268429 |
| 4002.268476 | Kernel\_Read\|xilinx:adm-pcie-7v3:1ddr:3.0-0\|cnn\_GOOD | Read | OCL Region | 1 | 1 | 142940 | 142941 | 0.00333333 | 4002.268476 | 4002.268479 |
| 4002.268526 | Kernel\_Read\|xilinx:adm-pcie-7v3:1ddr:3.0-0\|cnn\_GOOD | Read | OCL Region | 1 | 1 | 142955 | 142956 | 0.00333333 | 4002.268526 | 4002.268529 |
| 4002.268576 | Kernel\_Read\|xilinx:adm-pcie-7v3:1ddr:3.0-0\|cnn\_GOOD | Read | OCL Region | 1 | 1 | 142970 | 142971 | 0.00333333 | 4002.268576 | 4002.268579 |
| 4002.268626 | Kernel\_Read\|xilinx:adm-pcie-7v3:1ddr:3.0-0\|cnn\_GOOD | Read | OCL Region | 1 | 1 | 142985 | 142986 | 0.00333333 | 4002.268626 | 4002.268629 |
| 4002.268676 | Kernel\_Read\|xilinx:adm-pcie-7v3:1ddr:3.0-0\|cnn\_GOOD | Read | OCL Region | 1 | 1 | 143000 | 143001 | 0.00333333 | 4002.268676 | 4002.268679 |
| 4002.268726 | Kernel\_Read\|xilinx:adm-pcie-7v3:1ddr:3.0-0\|cnn\_GOOD | Read | OCL Region | 1 | 1 | 143015 | 143016 | 0.00333333 | 4002.268726 | 4002.268729 |
| 4002.268776 | Kernel\_Read\|xilinx:adm-pcie-7v3:1ddr:3.0-0\|cnn\_GOOD | Read | OCL Region | 1 | 1 | 143030 | 143031 | 0.00333333 | 4002.268776 | 4002.268779 |
| 4002.268826 | Kernel\_Read\|xilinx:adm-pcie-7v3:1ddr:3.0-0\|cnn\_GOOD | Read | OCL Region | 1 | 1 | 143045 | 143046 | 0.00333333 | 4002.268826 | 4002.268829 |
| 4002.268876 | Kernel\_Read\|xilinx:adm-pcie-7v3:1ddr:3.0-0\|cnn\_GOOD | Read | OCL Region | 1 | 1 | 143060 | 143061 | 0.00333333 | 4002.268876 | 4002.268879 |
| 4002.268926 | Kernel\_Read\|xilinx:adm-pcie-7v3:1ddr:3.0-0\|cnn\_GOOD | Read | OCL Region | 1 | 1 | 143075 | 143076 | 0.00333333 | 4002.268926 | 4002.268929 |
| 4002.268976 | Kernel\_Read\|xilinx:adm-pcie-7v3:1ddr:3.0-0\|cnn\_GOOD | Read | OCL Region | 1 | 1 | 143090 | 143091 | 0.00333333 | 4002.268976 | 4002.268979 |
| 4002.269026 | Kernel\_Read\|xilinx:adm-pcie-7v3:1ddr:3.0-0\|cnn\_GOOD | Read | OCL Region | 1 | 1 | 143105 | 143106 | 0.00333333 | 4002.269026 | 4002.269029 |
| 4002.269076 | Kernel\_Read\|xilinx:adm-pcie-7v3:1ddr:3.0-0\|cnn\_GOOD | Read | OCL Region | 1 | 1 | 143120 | 143121 | 0.00333333 | 4002.269076 | 4002.269079 |
| 4002.269126 | Kernel\_Read\|xilinx:adm-pcie-7v3:1ddr:3.0-0\|cnn\_GOOD | Read | OCL Region | 1 | 1 | 143135 | 143136 | 0.00333333 | 4002.269126 | 4002.269129 |
| 4002.269186 | Kernel\_Read\|xilinx:adm-pcie-7v3:1ddr:3.0-0\|cnn\_GOOD | Read | OCL Region | 1 | 1 | 143153 | 143154 | 0.00333333 | 4002.269186 | 4002.269189 |
| 4002.269459 | Kernel\_Read\|xilinx:adm-pcie-7v3:1ddr:3.0-0\|cnn\_GOOD | Read | OCL Region | 1 | 1 | 143235 | 143236 | 0.00333333 | 4002.269459 | 4002.269462 |
| 4002.269509 | Kernel\_Read\|xilinx:adm-pcie-7v3:1ddr:3.0-0\|cnn\_GOOD | Read | OCL Region | 1 | 1 | 143250 | 143251 | 0.00333333 | 4002.269509 | 4002.269512 |
| 4002.269552 | Kernel\_Read\|xilinx:adm-pcie-7v3:1ddr:3.0-0\|cnn\_GOOD | Read | OCL Region | 1 | 1 | 143263 | 143264 | 0.00333333 | 4002.269552 | 4002.269556 |
| 4002.269609 | Kernel\_Read\|xilinx:adm-pcie-7v3:1ddr:3.0-0\|cnn\_GOOD | Read | OCL Region | 1 | 1 | 143280 | 143281 | 0.00333333 | 4002.269609 | 4002.269612 |
| 4002.269662 | Kernel\_Read\|xilinx:adm-pcie-7v3:1ddr:3.0-0\|cnn\_GOOD | Read | OCL Region | 1 | 1 | 143296 | 143297 | 0.00333333 | 4002.269662 | 4002.269666 |
| 4002.269712 | Kernel\_Read\|xilinx:adm-pcie-7v3:1ddr:3.0-0\|cnn\_GOOD | Read | OCL Region | 1 | 1 | 143311 | 143312 | 0.00333333 | 4002.269712 | 4002.269716 |
| 4002.269762 | Kernel\_Read\|xilinx:adm-pcie-7v3:1ddr:3.0-0\|cnn\_GOOD | Read | OCL Region | 1 | 1 | 143326 | 143327 | 0.00333333 | 4002.269762 | 4002.269766 |
| 4002.269812 | Kernel\_Read\|xilinx:adm-pcie-7v3:1ddr:3.0-0\|cnn\_GOOD | Read | OCL Region | 1 | 1 | 143341 | 143342 | 0.00333333 | 4002.269812 | 4002.269816 |
| 4002.269862 | Kernel\_Read\|xilinx:adm-pcie-7v3:1ddr:3.0-0\|cnn\_GOOD | Read | OCL Region | 1 | 1 | 143356 | 143357 | 0.00333333 | 4002.269862 | 4002.269866 |
| 4002.269912 | Kernel\_Read\|xilinx:adm-pcie-7v3:1ddr:3.0-0\|cnn\_GOOD | Read | OCL Region | 1 | 1 | 143371 | 143372 | 0.00333333 | 4002.269912 | 4002.269916 |
| 4002.269962 | Kernel\_Read\|xilinx:adm-pcie-7v3:1ddr:3.0-0\|cnn\_GOOD | Read | OCL Region | 1 | 1 | 143386 | 143387 | 0.00333333 | 4002.269962 | 4002.269966 |
| 4002.270012 | Kernel\_Read\|xilinx:adm-pcie-7v3:1ddr:3.0-0\|cnn\_GOOD | Read | OCL Region | 1 | 1 | 143401 | 143402 | 0.00333333 | 4002.270012 | 4002.270016 |
| 4002.270062 | Kernel\_Read\|xilinx:adm-pcie-7v3:1ddr:3.0-0\|cnn\_GOOD | Read | OCL Region | 1 | 1 | 143416 | 143417 | 0.00333333 | 4002.270062 | 4002.270066 |
| 4002.270112 | Kernel\_Read\|xilinx:adm-pcie-7v3:1ddr:3.0-0\|cnn\_GOOD | Read | OCL Region | 1 | 1 | 143431 | 143432 | 0.00333333 | 4002.270112 | 4002.270116 |
| 4002.270169 | Kernel\_Read\|xilinx:adm-pcie-7v3:1ddr:3.0-0\|cnn\_GOOD | Read | OCL Region | 1 | 1 | 143448 | 143449 | 0.00333333 | 4002.270169 | 4002.270172 |
| 4002.270219 | Kernel\_Read\|xilinx:adm-pcie-7v3:1ddr:3.0-0\|cnn\_GOOD | Read | OCL Region | 1 | 1 | 143463 | 143464 | 0.00333333 | 4002.270219 | 4002.270222 |
| 4002.270269 | Kernel\_Read\|xilinx:adm-pcie-7v3:1ddr:3.0-0\|cnn\_GOOD | Read | OCL Region | 1 | 1 | 143478 | 143479 | 0.00333333 | 4002.270269 | 4002.270272 |
| 4002.270319 | Kernel\_Read\|xilinx:adm-pcie-7v3:1ddr:3.0-0\|cnn\_GOOD | Read | OCL Region | 1 | 1 | 143493 | 143494 | 0.00333333 | 4002.270319 | 4002.270322 |
| 4002.270369 | Kernel\_Read\|xilinx:adm-pcie-7v3:1ddr:3.0-0\|cnn\_GOOD | Read | OCL Region | 1 | 1 | 143508 | 143509 | 0.00333333 | 4002.270369 | 4002.270372 |
| 4002.270419 | Kernel\_Read\|xilinx:adm-pcie-7v3:1ddr:3.0-0\|cnn\_GOOD | Read | OCL Region | 1 | 1 | 143523 | 143524 | 0.00333333 | 4002.270419 | 4002.270422 |
| 4002.270469 | Kernel\_Read\|xilinx:adm-pcie-7v3:1ddr:3.0-0\|cnn\_GOOD | Read | OCL Region | 1 | 1 | 143538 | 143539 | 0.00333333 | 4002.270469 | 4002.270472 |
| 4002.270519 | Kernel\_Read\|xilinx:adm-pcie-7v3:1ddr:3.0-0\|cnn\_GOOD | Read | OCL Region | 1 | 1 | 143553 | 143554 | 0.00333333 | 4002.270519 | 4002.270522 |
| 4002.270569 | Kernel\_Read\|xilinx:adm-pcie-7v3:1ddr:3.0-0\|cnn\_GOOD | Read | OCL Region | 1 | 1 | 143568 | 143569 | 0.00333333 | 4002.270569 | 4002.270572 |
| 4002.270619 | Kernel\_Read\|xilinx:adm-pcie-7v3:1ddr:3.0-0\|cnn\_GOOD | Read | OCL Region | 1 | 1 | 143583 | 143584 | 0.00333333 | 4002.270619 | 4002.270622 |
| 4002.270669 | Kernel\_Read\|xilinx:adm-pcie-7v3:1ddr:3.0-0\|cnn\_GOOD | Read | OCL Region | 1 | 1 | 143598 | 143599 | 0.00333333 | 4002.270669 | 4002.270672 |
| 4002.270719 | Kernel\_Read\|xilinx:adm-pcie-7v3:1ddr:3.0-0\|cnn\_GOOD | Read | OCL Region | 1 | 1 | 143613 | 143614 | 0.00333333 | 4002.270719 | 4002.270722 |
| 4002.270769 | Kernel\_Read\|xilinx:adm-pcie-7v3:1ddr:3.0-0\|cnn\_GOOD | Read | OCL Region | 1 | 1 | 143628 | 143629 | 0.00333333 | 4002.270769 | 4002.270772 |
| 4002.270819 | Kernel\_Read\|xilinx:adm-pcie-7v3:1ddr:3.0-0\|cnn\_GOOD | Read | OCL Region | 1 | 1 | 143643 | 143644 | 0.00333333 | 4002.270819 | 4002.270822 |
| 4002.270869 | Kernel\_Read\|xilinx:adm-pcie-7v3:1ddr:3.0-0\|cnn\_GOOD | Read | OCL Region | 1 | 1 | 143658 | 143659 | 0.00333333 | 4002.270869 | 4002.270872 |
| 4002.270919 | Kernel\_Read\|xilinx:adm-pcie-7v3:1ddr:3.0-0\|cnn\_GOOD | Read | OCL Region | 1 | 1 | 143673 | 143674 | 0.00333333 | 4002.270919 | 4002.270922 |
| 4002.270969 | Kernel\_Read\|xilinx:adm-pcie-7v3:1ddr:3.0-0\|cnn\_GOOD | Read | OCL Region | 1 | 1 | 143688 | 143689 | 0.00333333 | 4002.270969 | 4002.270972 |
| 4002.271019 | Kernel\_Read\|xilinx:adm-pcie-7v3:1ddr:3.0-0\|cnn\_GOOD | Read | OCL Region | 1 | 1 | 143703 | 143704 | 0.00333333 | 4002.271019 | 4002.271022 |
| 4002.271069 | Kernel\_Read\|xilinx:adm-pcie-7v3:1ddr:3.0-0\|cnn\_GOOD | Read | OCL Region | 1 | 1 | 143718 | 143719 | 0.00333333 | 4002.271069 | 4002.271072 |
| 4002.271119 | Kernel\_Read\|xilinx:adm-pcie-7v3:1ddr:3.0-0\|cnn\_GOOD | Read | OCL Region | 1 | 1 | 143733 | 143734 | 0.00333333 | 4002.271119 | 4002.271122 |
| 4002.271182 | Kernel\_Read\|xilinx:adm-pcie-7v3:1ddr:3.0-0\|cnn\_GOOD | Read | OCL Region | 1 | 1 | 143752 | 143753 | 0.00333333 | 4002.271182 | 4002.271186 |
| 4002.271232 | Kernel\_Read\|xilinx:adm-pcie-7v3:1ddr:3.0-0\|cnn\_GOOD | Read | OCL Region | 1 | 1 | 143767 | 143768 | 0.00333333 | 4002.271232 | 4002.271236 |
| 4002.271282 | Kernel\_Read\|xilinx:adm-pcie-7v3:1ddr:3.0-0\|cnn\_GOOD | Read | OCL Region | 1 | 1 | 143782 | 143783 | 0.00333333 | 4002.271282 | 4002.271286 |
| 4002.271329 | Kernel\_Read\|xilinx:adm-pcie-7v3:1ddr:3.0-0\|cnn\_GOOD | Read | OCL Region | 1 | 1 | 143796 | 143797 | 0.00333333 | 4002.271329 | 4002.271332 |
| 4002.271382 | Kernel\_Read\|xilinx:adm-pcie-7v3:1ddr:3.0-0\|cnn\_GOOD | Read | OCL Region | 1 | 1 | 143812 | 143813 | 0.00333333 | 4002.271382 | 4002.271386 |
| 4002.271432 | Kernel\_Read\|xilinx:adm-pcie-7v3:1ddr:3.0-0\|cnn\_GOOD | Read | OCL Region | 1 | 1 | 143827 | 143828 | 0.00333333 | 4002.271432 | 4002.271436 |
| 4002.271482 | Kernel\_Read\|xilinx:adm-pcie-7v3:1ddr:3.0-0\|cnn\_GOOD | Read | OCL Region | 1 | 1 | 143842 | 143843 | 0.00333333 | 4002.271482 | 4002.271486 |
| 4002.271532 | Kernel\_Read\|xilinx:adm-pcie-7v3:1ddr:3.0-0\|cnn\_GOOD | Read | OCL Region | 1 | 1 | 143857 | 143858 | 0.00333333 | 4002.271532 | 4002.271536 |
| 4002.271582 | Kernel\_Read\|xilinx:adm-pcie-7v3:1ddr:3.0-0\|cnn\_GOOD | Read | OCL Region | 1 | 1 | 143872 | 143873 | 0.00333333 | 4002.271582 | 4002.271586 |
| 4002.271632 | Kernel\_Read\|xilinx:adm-pcie-7v3:1ddr:3.0-0\|cnn\_GOOD | Read | OCL Region | 1 | 1 | 143887 | 143888 | 0.00333333 | 4002.271632 | 4002.271636 |
| 4002.271682 | Kernel\_Read\|xilinx:adm-pcie-7v3:1ddr:3.0-0\|cnn\_GOOD | Read | OCL Region | 1 | 1 | 143902 | 143903 | 0.00333333 | 4002.271682 | 4002.271686 |
| 4002.271732 | Kernel\_Read\|xilinx:adm-pcie-7v3:1ddr:3.0-0\|cnn\_GOOD | Read | OCL Region | 1 | 1 | 143917 | 143918 | 0.00333333 | 4002.271732 | 4002.271736 |
| 4002.271782 | Kernel\_Read\|xilinx:adm-pcie-7v3:1ddr:3.0-0\|cnn\_GOOD | Read | OCL Region | 1 | 1 | 143932 | 143933 | 0.00333333 | 4002.271782 | 4002.271786 |
| 4002.271832 | Kernel\_Read\|xilinx:adm-pcie-7v3:1ddr:3.0-0\|cnn\_GOOD | Read | OCL Region | 1 | 1 | 143947 | 143948 | 0.00333333 | 4002.271832 | 4002.271836 |
| 4002.271882 | Kernel\_Read\|xilinx:adm-pcie-7v3:1ddr:3.0-0\|cnn\_GOOD | Read | OCL Region | 1 | 1 | 143962 | 143963 | 0.00333333 | 4002.271882 | 4002.271886 |
| 4002.271932 | Kernel\_Read\|xilinx:adm-pcie-7v3:1ddr:3.0-0\|cnn\_GOOD | Read | OCL Region | 1 | 1 | 143977 | 143978 | 0.00333333 | 4002.271932 | 4002.271936 |
| 4002.271982 | Kernel\_Read\|xilinx:adm-pcie-7v3:1ddr:3.0-0\|cnn\_GOOD | Read | OCL Region | 1 | 1 | 143992 | 143993 | 0.00333333 | 4002.271982 | 4002.271986 |
| 4002.272032 | Kernel\_Read\|xilinx:adm-pcie-7v3:1ddr:3.0-0\|cnn\_GOOD | Read | OCL Region | 1 | 1 | 144007 | 144008 | 0.00333333 | 4002.272032 | 4002.272036 |
| 4002.272082 | Kernel\_Read\|xilinx:adm-pcie-7v3:1ddr:3.0-0\|cnn\_GOOD | Read | OCL Region | 1 | 1 | 144022 | 144023 | 0.00333333 | 4002.272082 | 4002.272086 |
| 4002.272142 | Kernel\_Read\|xilinx:adm-pcie-7v3:1ddr:3.0-0\|cnn\_GOOD | Read | OCL Region | 1 | 1 | 144040 | 144041 | 0.00333333 | 4002.272142 | 4002.272146 |
| 4002.272192 | Kernel\_Read\|xilinx:adm-pcie-7v3:1ddr:3.0-0\|cnn\_GOOD | Read | OCL Region | 1 | 1 | 144055 | 144056 | 0.00333333 | 4002.272192 | 4002.272196 |
| 4002.272242 | Kernel\_Read\|xilinx:adm-pcie-7v3:1ddr:3.0-0\|cnn\_GOOD | Read | OCL Region | 1 | 1 | 144070 | 144071 | 0.00333333 | 4002.272242 | 4002.272246 |
| 4002.272292 | Kernel\_Read\|xilinx:adm-pcie-7v3:1ddr:3.0-0\|cnn\_GOOD | Read | OCL Region | 1 | 1 | 144085 | 144086 | 0.00333333 | 4002.272292 | 4002.272296 |
| 4002.272342 | Kernel\_Read\|xilinx:adm-pcie-7v3:1ddr:3.0-0\|cnn\_GOOD | Read | OCL Region | 1 | 1 | 144100 | 144101 | 0.00333333 | 4002.272342 | 4002.272346 |
| 4002.272392 | Kernel\_Read\|xilinx:adm-pcie-7v3:1ddr:3.0-0\|cnn\_GOOD | Read | OCL Region | 1 | 1 | 144115 | 144116 | 0.00333333 | 4002.272392 | 4002.272396 |
| 4002.272442 | Kernel\_Read\|xilinx:adm-pcie-7v3:1ddr:3.0-0\|cnn\_GOOD | Read | OCL Region | 1 | 1 | 144130 | 144131 | 0.00333333 | 4002.272442 | 4002.272446 |
| 4002.272492 | Kernel\_Read\|xilinx:adm-pcie-7v3:1ddr:3.0-0\|cnn\_GOOD | Read | OCL Region | 1 | 1 | 144145 | 144146 | 0.00333333 | 4002.272492 | 4002.272496 |
| 4002.272542 | Kernel\_Read\|xilinx:adm-pcie-7v3:1ddr:3.0-0\|cnn\_GOOD | Read | OCL Region | 1 | 1 | 144160 | 144161 | 0.00333333 | 4002.272542 | 4002.272546 |
| 4002.272592 | Kernel\_Read\|xilinx:adm-pcie-7v3:1ddr:3.0-0\|cnn\_GOOD | Read | OCL Region | 1 | 1 | 144175 | 144176 | 0.00333333 | 4002.272592 | 4002.272596 |
| 4002.272642 | Kernel\_Read\|xilinx:adm-pcie-7v3:1ddr:3.0-0\|cnn\_GOOD | Read | OCL Region | 1 | 1 | 144190 | 144191 | 0.00333333 | 4002.272642 | 4002.272646 |
| 4002.272692 | Kernel\_Read\|xilinx:adm-pcie-7v3:1ddr:3.0-0\|cnn\_GOOD | Read | OCL Region | 1 | 1 | 144205 | 144206 | 0.00333333 | 4002.272692 | 4002.272696 |
| 4002.272742 | Kernel\_Read\|xilinx:adm-pcie-7v3:1ddr:3.0-0\|cnn\_GOOD | Read | OCL Region | 1 | 1 | 144220 | 144221 | 0.00333333 | 4002.272742 | 4002.272746 |
| 4002.272792 | Kernel\_Read\|xilinx:adm-pcie-7v3:1ddr:3.0-0\|cnn\_GOOD | Read | OCL Region | 1 | 1 | 144235 | 144236 | 0.00333333 | 4002.272792 | 4002.272796 |
| 4002.272842 | Kernel\_Read\|xilinx:adm-pcie-7v3:1ddr:3.0-0\|cnn\_GOOD | Read | OCL Region | 1 | 1 | 144250 | 144251 | 0.00333333 | 4002.272842 | 4002.272846 |
| 4002.272892 | Kernel\_Read\|xilinx:adm-pcie-7v3:1ddr:3.0-0\|cnn\_GOOD | Read | OCL Region | 1 | 1 | 144265 | 144266 | 0.00333333 | 4002.272892 | 4002.272896 |
| 4002.272942 | Kernel\_Read\|xilinx:adm-pcie-7v3:1ddr:3.0-0\|cnn\_GOOD | Read | OCL Region | 1 | 1 | 144280 | 144281 | 0.00333333 | 4002.272942 | 4002.272946 |
| 4002.272992 | Kernel\_Read\|xilinx:adm-pcie-7v3:1ddr:3.0-0\|cnn\_GOOD | Read | OCL Region | 1 | 1 | 144295 | 144296 | 0.00333333 | 4002.272992 | 4002.272996 |
| 4002.273042 | Kernel\_Read\|xilinx:adm-pcie-7v3:1ddr:3.0-0\|cnn\_GOOD | Read | OCL Region | 1 | 1 | 144310 | 144311 | 0.00333333 | 4002.273042 | 4002.273046 |
| 4002.273092 | Kernel\_Read\|xilinx:adm-pcie-7v3:1ddr:3.0-0\|cnn\_GOOD | Read | OCL Region | 1 | 1 | 144325 | 144326 | 0.00333333 | 4002.273092 | 4002.273096 |
| 4002.273149 | Kernel\_Read\|xilinx:adm-pcie-7v3:1ddr:3.0-0\|cnn\_GOOD | Read | OCL Region | 1 | 1 | 144342 | 144343 | 0.00333333 | 4002.273149 | 4002.273152 |
| 4002.273199 | Kernel\_Read\|xilinx:adm-pcie-7v3:1ddr:3.0-0\|cnn\_GOOD | Read | OCL Region | 1 | 1 | 144357 | 144358 | 0.00333333 | 4002.273199 | 4002.273202 |
| 4002.273249 | Kernel\_Read\|xilinx:adm-pcie-7v3:1ddr:3.0-0\|cnn\_GOOD | Read | OCL Region | 1 | 1 | 144372 | 144373 | 0.00333333 | 4002.273249 | 4002.273252 |
| 4002.273299 | Kernel\_Read\|xilinx:adm-pcie-7v3:1ddr:3.0-0\|cnn\_GOOD | Read | OCL Region | 1 | 1 | 144387 | 144388 | 0.00333333 | 4002.273299 | 4002.273302 |
| 4002.273349 | Kernel\_Read\|xilinx:adm-pcie-7v3:1ddr:3.0-0\|cnn\_GOOD | Read | OCL Region | 1 | 1 | 144402 | 144403 | 0.00333333 | 4002.273349 | 4002.273352 |
| 4002.273399 | Kernel\_Read\|xilinx:adm-pcie-7v3:1ddr:3.0-0\|cnn\_GOOD | Read | OCL Region | 1 | 1 | 144417 | 144418 | 0.00333333 | 4002.273399 | 4002.273402 |
| 4002.273449 | Kernel\_Read\|xilinx:adm-pcie-7v3:1ddr:3.0-0\|cnn\_GOOD | Read | OCL Region | 1 | 1 | 144432 | 144433 | 0.00333333 | 4002.273449 | 4002.273452 |
| 4002.273499 | Kernel\_Read\|xilinx:adm-pcie-7v3:1ddr:3.0-0\|cnn\_GOOD | Read | OCL Region | 1 | 1 | 144447 | 144448 | 0.00333333 | 4002.273499 | 4002.273502 |
| 4002.273549 | Kernel\_Read\|xilinx:adm-pcie-7v3:1ddr:3.0-0\|cnn\_GOOD | Read | OCL Region | 1 | 1 | 144462 | 144463 | 0.00333333 | 4002.273549 | 4002.273552 |
| 4002.273599 | Kernel\_Read\|xilinx:adm-pcie-7v3:1ddr:3.0-0\|cnn\_GOOD | Read | OCL Region | 1 | 1 | 144477 | 144478 | 0.00333333 | 4002.273599 | 4002.273602 |
| 4002.273649 | Kernel\_Read\|xilinx:adm-pcie-7v3:1ddr:3.0-0\|cnn\_GOOD | Read | OCL Region | 1 | 1 | 144492 | 144493 | 0.00333333 | 4002.273649 | 4002.273652 |
| 4002.273699 | Kernel\_Read\|xilinx:adm-pcie-7v3:1ddr:3.0-0\|cnn\_GOOD | Read | OCL Region | 1 | 1 | 144507 | 144508 | 0.00333333 | 4002.273699 | 4002.273702 |
| 4002.273749 | Kernel\_Read\|xilinx:adm-pcie-7v3:1ddr:3.0-0\|cnn\_GOOD | Read | OCL Region | 1 | 1 | 144522 | 144523 | 0.00333333 | 4002.273749 | 4002.273752 |
| 4002.273799 | Kernel\_Read\|xilinx:adm-pcie-7v3:1ddr:3.0-0\|cnn\_GOOD | Read | OCL Region | 1 | 1 | 144537 | 144538 | 0.00333333 | 4002.273799 | 4002.273802 |
| 4002.273849 | Kernel\_Read\|xilinx:adm-pcie-7v3:1ddr:3.0-0\|cnn\_GOOD | Read | OCL Region | 1 | 1 | 144552 | 144553 | 0.00333333 | 4002.273849 | 4002.273852 |
| 4002.273899 | Kernel\_Read\|xilinx:adm-pcie-7v3:1ddr:3.0-0\|cnn\_GOOD | Read | OCL Region | 1 | 1 | 144567 | 144568 | 0.00333333 | 4002.273899 | 4002.273902 |
| 4002.273949 | Kernel\_Read\|xilinx:adm-pcie-7v3:1ddr:3.0-0\|cnn\_GOOD | Read | OCL Region | 1 | 1 | 144582 | 144583 | 0.00333333 | 4002.273949 | 4002.273952 |
| 4002.273999 | Kernel\_Read\|xilinx:adm-pcie-7v3:1ddr:3.0-0\|cnn\_GOOD | Read | OCL Region | 1 | 1 | 144597 | 144598 | 0.00333333 | 4002.273999 | 4002.274002 |
| 4002.274049 | Kernel\_Read\|xilinx:adm-pcie-7v3:1ddr:3.0-0\|cnn\_GOOD | Read | OCL Region | 1 | 1 | 144612 | 144613 | 0.00333333 | 4002.274049 | 4002.274052 |
| 4002.274099 | Kernel\_Read\|xilinx:adm-pcie-7v3:1ddr:3.0-0\|cnn\_GOOD | Read | OCL Region | 1 | 1 | 144627 | 144628 | 0.00333333 | 4002.274099 | 4002.274102 |
| 4002.274162 | Kernel\_Read\|xilinx:adm-pcie-7v3:1ddr:3.0-0\|cnn\_GOOD | Read | OCL Region | 1 | 1 | 144646 | 144647 | 0.00333333 | 4002.274162 | 4002.274166 |
| 4002.274212 | Kernel\_Read\|xilinx:adm-pcie-7v3:1ddr:3.0-0\|cnn\_GOOD | Read | OCL Region | 1 | 1 | 144661 | 144662 | 0.00333333 | 4002.274212 | 4002.274216 |
| 4002.274262 | Kernel\_Read\|xilinx:adm-pcie-7v3:1ddr:3.0-0\|cnn\_GOOD | Read | OCL Region | 1 | 1 | 144676 | 144677 | 0.00333333 | 4002.274262 | 4002.274266 |
| 4002.274312 | Kernel\_Read\|xilinx:adm-pcie-7v3:1ddr:3.0-0\|cnn\_GOOD | Read | OCL Region | 1 | 1 | 144691 | 144692 | 0.00333333 | 4002.274312 | 4002.274316 |
| 4002.274362 | Kernel\_Read\|xilinx:adm-pcie-7v3:1ddr:3.0-0\|cnn\_GOOD | Read | OCL Region | 1 | 1 | 144706 | 144707 | 0.00333333 | 4002.274362 | 4002.274366 |
| 4002.274412 | Kernel\_Read\|xilinx:adm-pcie-7v3:1ddr:3.0-0\|cnn\_GOOD | Read | OCL Region | 1 | 1 | 144721 | 144722 | 0.00333333 | 4002.274412 | 4002.274416 |
| 4002.274462 | Kernel\_Read\|xilinx:adm-pcie-7v3:1ddr:3.0-0\|cnn\_GOOD | Read | OCL Region | 1 | 1 | 144736 | 144737 | 0.00333333 | 4002.274462 | 4002.274466 |
| 4002.274512 | Kernel\_Read\|xilinx:adm-pcie-7v3:1ddr:3.0-0\|cnn\_GOOD | Read | OCL Region | 1 | 1 | 144751 | 144752 | 0.00333333 | 4002.274512 | 4002.274516 |
| 4002.274562 | Kernel\_Read\|xilinx:adm-pcie-7v3:1ddr:3.0-0\|cnn\_GOOD | Read | OCL Region | 1 | 1 | 144766 | 144767 | 0.00333333 | 4002.274562 | 4002.274566 |
| 4002.274612 | Kernel\_Read\|xilinx:adm-pcie-7v3:1ddr:3.0-0\|cnn\_GOOD | Read | OCL Region | 1 | 1 | 144781 | 144782 | 0.00333333 | 4002.274612 | 4002.274616 |
| 4002.274662 | Kernel\_Read\|xilinx:adm-pcie-7v3:1ddr:3.0-0\|cnn\_GOOD | Read | OCL Region | 1 | 1 | 144796 | 144797 | 0.00333333 | 4002.274662 | 4002.274666 |
| 4002.274712 | Kernel\_Read\|xilinx:adm-pcie-7v3:1ddr:3.0-0\|cnn\_GOOD | Read | OCL Region | 1 | 1 | 144811 | 144812 | 0.00333333 | 4002.274712 | 4002.274716 |
| 4002.274762 | Kernel\_Read\|xilinx:adm-pcie-7v3:1ddr:3.0-0\|cnn\_GOOD | Read | OCL Region | 1 | 1 | 144826 | 144827 | 0.00333333 | 4002.274762 | 4002.274766 |
| 4002.274812 | Kernel\_Read\|xilinx:adm-pcie-7v3:1ddr:3.0-0\|cnn\_GOOD | Read | OCL Region | 1 | 1 | 144841 | 144842 | 0.00333333 | 4002.274812 | 4002.274816 |
| 4002.274862 | Kernel\_Read\|xilinx:adm-pcie-7v3:1ddr:3.0-0\|cnn\_GOOD | Read | OCL Region | 1 | 1 | 144856 | 144857 | 0.00333333 | 4002.274862 | 4002.274866 |
| 4002.274912 | Kernel\_Read\|xilinx:adm-pcie-7v3:1ddr:3.0-0\|cnn\_GOOD | Read | OCL Region | 1 | 1 | 144871 | 144872 | 0.00333333 | 4002.274912 | 4002.274916 |
| 4002.274962 | Kernel\_Read\|xilinx:adm-pcie-7v3:1ddr:3.0-0\|cnn\_GOOD | Read | OCL Region | 1 | 1 | 144886 | 144887 | 0.00333333 | 4002.274962 | 4002.274966 |
| 4002.275012 | Kernel\_Read\|xilinx:adm-pcie-7v3:1ddr:3.0-0\|cnn\_GOOD | Read | OCL Region | 1 | 1 | 144901 | 144902 | 0.00333333 | 4002.275012 | 4002.275016 |
| 4002.275062 | Kernel\_Read\|xilinx:adm-pcie-7v3:1ddr:3.0-0\|cnn\_GOOD | Read | OCL Region | 1 | 1 | 144916 | 144917 | 0.00333333 | 4002.275062 | 4002.275066 |
| 4002.275119 | Kernel\_Read\|xilinx:adm-pcie-7v3:1ddr:3.0-0\|cnn\_GOOD | Read | OCL Region | 1 | 1 | 144933 | 144934 | 0.00333333 | 4002.275119 | 4002.275122 |
| 4002.275169 | Kernel\_Read\|xilinx:adm-pcie-7v3:1ddr:3.0-0\|cnn\_GOOD | Read | OCL Region | 1 | 1 | 144948 | 144949 | 0.00333333 | 4002.275169 | 4002.275172 |
| 4002.275219 | Kernel\_Read\|xilinx:adm-pcie-7v3:1ddr:3.0-0\|cnn\_GOOD | Read | OCL Region | 1 | 1 | 144963 | 144964 | 0.00333333 | 4002.275219 | 4002.275222 |
| 4002.275269 | Kernel\_Read\|xilinx:adm-pcie-7v3:1ddr:3.0-0\|cnn\_GOOD | Read | OCL Region | 1 | 1 | 144978 | 144979 | 0.00333333 | 4002.275269 | 4002.275272 |
| 4002.275319 | Kernel\_Read\|xilinx:adm-pcie-7v3:1ddr:3.0-0\|cnn\_GOOD | Read | OCL Region | 1 | 1 | 144993 | 144994 | 0.00333333 | 4002.275319 | 4002.275322 |
| 4002.275369 | Kernel\_Read\|xilinx:adm-pcie-7v3:1ddr:3.0-0\|cnn\_GOOD | Read | OCL Region | 1 | 1 | 145008 | 145009 | 0.00333333 | 4002.275369 | 4002.275372 |
| 4002.275419 | Kernel\_Read\|xilinx:adm-pcie-7v3:1ddr:3.0-0\|cnn\_GOOD | Read | OCL Region | 1 | 1 | 145023 | 145024 | 0.00333333 | 4002.275419 | 4002.275422 |
| 4002.275469 | Kernel\_Read\|xilinx:adm-pcie-7v3:1ddr:3.0-0\|cnn\_GOOD | Read | OCL Region | 1 | 1 | 145038 | 145039 | 0.00333333 | 4002.275469 | 4002.275472 |
| 4002.275519 | Kernel\_Read\|xilinx:adm-pcie-7v3:1ddr:3.0-0\|cnn\_GOOD | Read | OCL Region | 1 | 1 | 145053 | 145054 | 0.00333333 | 4002.275519 | 4002.275522 |
| 4002.275569 | Kernel\_Read\|xilinx:adm-pcie-7v3:1ddr:3.0-0\|cnn\_GOOD | Read | OCL Region | 1 | 1 | 145068 | 145069 | 0.00333333 | 4002.275569 | 4002.275572 |
| 4002.275842 | Kernel\_Read\|xilinx:adm-pcie-7v3:1ddr:3.0-0\|cnn\_GOOD | Read | OCL Region | 1 | 1 | 145150 | 145151 | 0.00333333 | 4002.275842 | 4002.275846 |
| 4002.275892 | Kernel\_Read\|xilinx:adm-pcie-7v3:1ddr:3.0-0\|cnn\_GOOD | Read | OCL Region | 1 | 1 | 145165 | 145166 | 0.00333333 | 4002.275892 | 4002.275896 |
| 4002.275942 | Kernel\_Read\|xilinx:adm-pcie-7v3:1ddr:3.0-0\|cnn\_GOOD | Read | OCL Region | 1 | 1 | 145180 | 145181 | 0.00333333 | 4002.275942 | 4002.275946 |
| 4002.275992 | Kernel\_Read\|xilinx:adm-pcie-7v3:1ddr:3.0-0\|cnn\_GOOD | Read | OCL Region | 1 | 1 | 145195 | 145196 | 0.00333333 | 4002.275992 | 4002.275996 |
| 4002.276046 | Kernel\_Read\|xilinx:adm-pcie-7v3:1ddr:3.0-0\|cnn\_GOOD | Read | OCL Region | 1 | 1 | 145211 | 145212 | 0.00333333 | 4002.276046 | 4002.276049 |
| 4002.276109 | Kernel\_Read\|xilinx:adm-pcie-7v3:1ddr:3.0-0\|cnn\_GOOD | Read | OCL Region | 1 | 1 | 145230 | 145231 | 0.00333333 | 4002.276109 | 4002.276112 |
| 4002.276159 | Kernel\_Read\|xilinx:adm-pcie-7v3:1ddr:3.0-0\|cnn\_GOOD | Read | OCL Region | 1 | 1 | 145245 | 145246 | 0.00333333 | 4002.276159 | 4002.276162 |
| 4002.276209 | Kernel\_Read\|xilinx:adm-pcie-7v3:1ddr:3.0-0\|cnn\_GOOD | Read | OCL Region | 1 | 1 | 145260 | 145261 | 0.00333333 | 4002.276209 | 4002.276212 |
| 4002.276259 | Kernel\_Read\|xilinx:adm-pcie-7v3:1ddr:3.0-0\|cnn\_GOOD | Read | OCL Region | 1 | 1 | 145275 | 145276 | 0.00333333 | 4002.276259 | 4002.276262 |
| 4002.276309 | Kernel\_Read\|xilinx:adm-pcie-7v3:1ddr:3.0-0\|cnn\_GOOD | Read | OCL Region | 1 | 1 | 145290 | 145291 | 0.00333333 | 4002.276309 | 4002.276312 |
| 4002.276359 | Kernel\_Read\|xilinx:adm-pcie-7v3:1ddr:3.0-0\|cnn\_GOOD | Read | OCL Region | 1 | 1 | 145305 | 145306 | 0.00333333 | 4002.276359 | 4002.276362 |
| 4002.276409 | Kernel\_Read\|xilinx:adm-pcie-7v3:1ddr:3.0-0\|cnn\_GOOD | Read | OCL Region | 1 | 1 | 145320 | 145321 | 0.00333333 | 4002.276409 | 4002.276412 |
| 4002.276459 | Kernel\_Read\|xilinx:adm-pcie-7v3:1ddr:3.0-0\|cnn\_GOOD | Read | OCL Region | 1 | 1 | 145335 | 145336 | 0.00333333 | 4002.276459 | 4002.276462 |
| 4002.276509 | Kernel\_Read\|xilinx:adm-pcie-7v3:1ddr:3.0-0\|cnn\_GOOD | Read | OCL Region | 1 | 1 | 145350 | 145351 | 0.00333333 | 4002.276509 | 4002.276512 |
| 4002.276559 | Kernel\_Read\|xilinx:adm-pcie-7v3:1ddr:3.0-0\|cnn\_GOOD | Read | OCL Region | 1 | 1 | 145365 | 145366 | 0.00333333 | 4002.276559 | 4002.276562 |
| 4002.276609 | Kernel\_Read\|xilinx:adm-pcie-7v3:1ddr:3.0-0\|cnn\_GOOD | Read | OCL Region | 1 | 1 | 145380 | 145381 | 0.00333333 | 4002.276609 | 4002.276612 |
| 4002.276659 | Kernel\_Read\|xilinx:adm-pcie-7v3:1ddr:3.0-0\|cnn\_GOOD | Read | OCL Region | 1 | 1 | 145395 | 145396 | 0.00333333 | 4002.276659 | 4002.276662 |
| 4002.276709 | Kernel\_Read\|xilinx:adm-pcie-7v3:1ddr:3.0-0\|cnn\_GOOD | Read | OCL Region | 1 | 1 | 145410 | 145411 | 0.00333333 | 4002.276709 | 4002.276712 |
| 4002.276759 | Kernel\_Read\|xilinx:adm-pcie-7v3:1ddr:3.0-0\|cnn\_GOOD | Read | OCL Region | 1 | 1 | 145425 | 145426 | 0.00333333 | 4002.276759 | 4002.276762 |
| 4002.276809 | Kernel\_Read\|xilinx:adm-pcie-7v3:1ddr:3.0-0\|cnn\_GOOD | Read | OCL Region | 1 | 1 | 145440 | 145441 | 0.00333333 | 4002.276809 | 4002.276812 |
| 4002.276859 | Kernel\_Read\|xilinx:adm-pcie-7v3:1ddr:3.0-0\|cnn\_GOOD | Read | OCL Region | 1 | 1 | 145455 | 145456 | 0.00333333 | 4002.276859 | 4002.276862 |
| 4002.276909 | Kernel\_Read\|xilinx:adm-pcie-7v3:1ddr:3.0-0\|cnn\_GOOD | Read | OCL Region | 1 | 1 | 145470 | 145471 | 0.00333333 | 4002.276909 | 4002.276912 |
| 4002.276959 | Kernel\_Read\|xilinx:adm-pcie-7v3:1ddr:3.0-0\|cnn\_GOOD | Read | OCL Region | 1 | 1 | 145485 | 145486 | 0.00333333 | 4002.276959 | 4002.276962 |
| 4002.277009 | Kernel\_Read\|xilinx:adm-pcie-7v3:1ddr:3.0-0\|cnn\_GOOD | Read | OCL Region | 1 | 1 | 145500 | 145501 | 0.00333333 | 4002.277009 | 4002.277012 |
| 4002.277059 | Kernel\_Read\|xilinx:adm-pcie-7v3:1ddr:3.0-0\|cnn\_GOOD | Read | OCL Region | 1 | 1 | 145515 | 145516 | 0.00333333 | 4002.277059 | 4002.277062 |
| 4002.277116 | Kernel\_Read\|xilinx:adm-pcie-7v3:1ddr:3.0-0\|cnn\_GOOD | Read | OCL Region | 1 | 1 | 145532 | 145533 | 0.00333333 | 4002.277116 | 4002.277119 |
| 4002.277166 | Kernel\_Read\|xilinx:adm-pcie-7v3:1ddr:3.0-0\|cnn\_GOOD | Read | OCL Region | 1 | 1 | 145547 | 145548 | 0.00333333 | 4002.277166 | 4002.277169 |
| 4002.277216 | Kernel\_Read\|xilinx:adm-pcie-7v3:1ddr:3.0-0\|cnn\_GOOD | Read | OCL Region | 1 | 1 | 145562 | 145563 | 0.00333333 | 4002.277216 | 4002.277219 |
| 4002.277266 | Kernel\_Read\|xilinx:adm-pcie-7v3:1ddr:3.0-0\|cnn\_GOOD | Read | OCL Region | 1 | 1 | 145577 | 145578 | 0.00333333 | 4002.277266 | 4002.277269 |
| 4002.277316 | Kernel\_Read\|xilinx:adm-pcie-7v3:1ddr:3.0-0\|cnn\_GOOD | Read | OCL Region | 1 | 1 | 145592 | 145593 | 0.00333333 | 4002.277316 | 4002.277319 |
| 4002.277366 | Kernel\_Read\|xilinx:adm-pcie-7v3:1ddr:3.0-0\|cnn\_GOOD | Read | OCL Region | 1 | 1 | 145607 | 145608 | 0.00333333 | 4002.277366 | 4002.277369 |
| 4002.277416 | Kernel\_Read\|xilinx:adm-pcie-7v3:1ddr:3.0-0\|cnn\_GOOD | Read | OCL Region | 1 | 1 | 145622 | 145623 | 0.00333333 | 4002.277416 | 4002.277419 |
| 4002.277466 | Kernel\_Read\|xilinx:adm-pcie-7v3:1ddr:3.0-0\|cnn\_GOOD | Read | OCL Region | 1 | 1 | 145637 | 145638 | 0.00333333 | 4002.277466 | 4002.277469 |
| 4002.277516 | Kernel\_Read\|xilinx:adm-pcie-7v3:1ddr:3.0-0\|cnn\_GOOD | Read | OCL Region | 1 | 1 | 145652 | 145653 | 0.00333333 | 4002.277516 | 4002.277519 |
| 4002.277566 | Kernel\_Read\|xilinx:adm-pcie-7v3:1ddr:3.0-0\|cnn\_GOOD | Read | OCL Region | 1 | 1 | 145667 | 145668 | 0.00333333 | 4002.277566 | 4002.277569 |
| 4002.277616 | Kernel\_Read\|xilinx:adm-pcie-7v3:1ddr:3.0-0\|cnn\_GOOD | Read | OCL Region | 1 | 1 | 145682 | 145683 | 0.00333333 | 4002.277616 | 4002.277619 |
| 4002.277666 | Kernel\_Read\|xilinx:adm-pcie-7v3:1ddr:3.0-0\|cnn\_GOOD | Read | OCL Region | 1 | 1 | 145697 | 145698 | 0.00333333 | 4002.277666 | 4002.277669 |
| 4002.277716 | Kernel\_Read\|xilinx:adm-pcie-7v3:1ddr:3.0-0\|cnn\_GOOD | Read | OCL Region | 1 | 1 | 145712 | 145713 | 0.00333333 | 4002.277716 | 4002.277719 |
| 4002.277766 | Kernel\_Read\|xilinx:adm-pcie-7v3:1ddr:3.0-0\|cnn\_GOOD | Read | OCL Region | 1 | 1 | 145727 | 145728 | 0.00333333 | 4002.277766 | 4002.277769 |
| 4002.277816 | Kernel\_Read\|xilinx:adm-pcie-7v3:1ddr:3.0-0\|cnn\_GOOD | Read | OCL Region | 1 | 1 | 145742 | 145743 | 0.00333333 | 4002.277816 | 4002.277819 |
| 4002.277866 | Kernel\_Read\|xilinx:adm-pcie-7v3:1ddr:3.0-0\|cnn\_GOOD | Read | OCL Region | 1 | 1 | 145757 | 145758 | 0.00333333 | 4002.277866 | 4002.277869 |
| 4002.277916 | Kernel\_Read\|xilinx:adm-pcie-7v3:1ddr:3.0-0\|cnn\_GOOD | Read | OCL Region | 1 | 1 | 145772 | 145773 | 0.00333333 | 4002.277916 | 4002.277919 |
| 4002.277966 | Kernel\_Read\|xilinx:adm-pcie-7v3:1ddr:3.0-0\|cnn\_GOOD | Read | OCL Region | 1 | 1 | 145787 | 145788 | 0.00333333 | 4002.277966 | 4002.277969 |
| 4002.278016 | Kernel\_Read\|xilinx:adm-pcie-7v3:1ddr:3.0-0\|cnn\_GOOD | Read | OCL Region | 1 | 1 | 145802 | 145803 | 0.00333333 | 4002.278016 | 4002.278019 |
| 4002.278066 | Kernel\_Read\|xilinx:adm-pcie-7v3:1ddr:3.0-0\|cnn\_GOOD | Read | OCL Region | 1 | 1 | 145817 | 145818 | 0.00333333 | 4002.278066 | 4002.278069 |
| 4002.278126 | Kernel\_Read\|xilinx:adm-pcie-7v3:1ddr:3.0-0\|cnn\_GOOD | Read | OCL Region | 1 | 1 | 145835 | 145836 | 0.00333333 | 4002.278126 | 4002.278129 |
| 4002.278176 | Kernel\_Read\|xilinx:adm-pcie-7v3:1ddr:3.0-0\|cnn\_GOOD | Read | OCL Region | 1 | 1 | 145850 | 145851 | 0.00333333 | 4002.278176 | 4002.278179 |
| 4002.278226 | Kernel\_Read\|xilinx:adm-pcie-7v3:1ddr:3.0-0\|cnn\_GOOD | Read | OCL Region | 1 | 1 | 145865 | 145866 | 0.00333333 | 4002.278226 | 4002.278229 |
| 4002.278276 | Kernel\_Read\|xilinx:adm-pcie-7v3:1ddr:3.0-0\|cnn\_GOOD | Read | OCL Region | 1 | 1 | 145880 | 145881 | 0.00333333 | 4002.278276 | 4002.278279 |
| 4002.278326 | Kernel\_Read\|xilinx:adm-pcie-7v3:1ddr:3.0-0\|cnn\_GOOD | Read | OCL Region | 1 | 1 | 145895 | 145896 | 0.00333333 | 4002.278326 | 4002.278329 |
| 4002.278376 | Kernel\_Read\|xilinx:adm-pcie-7v3:1ddr:3.0-0\|cnn\_GOOD | Read | OCL Region | 1 | 1 | 145910 | 145911 | 0.00333333 | 4002.278376 | 4002.278379 |
| 4002.278426 | Kernel\_Read\|xilinx:adm-pcie-7v3:1ddr:3.0-0\|cnn\_GOOD | Read | OCL Region | 1 | 1 | 145925 | 145926 | 0.00333333 | 4002.278426 | 4002.278429 |
| 4002.278476 | Kernel\_Read\|xilinx:adm-pcie-7v3:1ddr:3.0-0\|cnn\_GOOD | Read | OCL Region | 1 | 1 | 145940 | 145941 | 0.00333333 | 4002.278476 | 4002.278479 |
| 4002.278526 | Kernel\_Read\|xilinx:adm-pcie-7v3:1ddr:3.0-0\|cnn\_GOOD | Read | OCL Region | 1 | 1 | 145955 | 145956 | 0.00333333 | 4002.278526 | 4002.278529 |
| 4002.278576 | Kernel\_Read\|xilinx:adm-pcie-7v3:1ddr:3.0-0\|cnn\_GOOD | Read | OCL Region | 1 | 1 | 145970 | 145971 | 0.00333333 | 4002.278576 | 4002.278579 |
| 4002.278626 | Kernel\_Read\|xilinx:adm-pcie-7v3:1ddr:3.0-0\|cnn\_GOOD | Read | OCL Region | 1 | 1 | 145985 | 145986 | 0.00333333 | 4002.278626 | 4002.278629 |
| 4002.278676 | Kernel\_Read\|xilinx:adm-pcie-7v3:1ddr:3.0-0\|cnn\_GOOD | Read | OCL Region | 1 | 1 | 146000 | 146001 | 0.00333333 | 4002.278676 | 4002.278679 |
| 4002.278726 | Kernel\_Read\|xilinx:adm-pcie-7v3:1ddr:3.0-0\|cnn\_GOOD | Read | OCL Region | 1 | 1 | 146015 | 146016 | 0.00333333 | 4002.278726 | 4002.278729 |
| 4002.278776 | Kernel\_Read\|xilinx:adm-pcie-7v3:1ddr:3.0-0\|cnn\_GOOD | Read | OCL Region | 1 | 1 | 146030 | 146031 | 0.00333333 | 4002.278776 | 4002.278779 |
| 4002.278826 | Kernel\_Read\|xilinx:adm-pcie-7v3:1ddr:3.0-0\|cnn\_GOOD | Read | OCL Region | 1 | 1 | 146045 | 146046 | 0.00333333 | 4002.278826 | 4002.278829 |
| 4002.278876 | Kernel\_Read\|xilinx:adm-pcie-7v3:1ddr:3.0-0\|cnn\_GOOD | Read | OCL Region | 1 | 1 | 146060 | 146061 | 0.00333333 | 4002.278876 | 4002.278879 |
| 4002.278926 | Kernel\_Read\|xilinx:adm-pcie-7v3:1ddr:3.0-0\|cnn\_GOOD | Read | OCL Region | 1 | 1 | 146075 | 146076 | 0.00333333 | 4002.278926 | 4002.278929 |
| 4002.278976 | Kernel\_Read\|xilinx:adm-pcie-7v3:1ddr:3.0-0\|cnn\_GOOD | Read | OCL Region | 1 | 1 | 146090 | 146091 | 0.00333333 | 4002.278976 | 4002.278979 |
| 4002.279026 | Kernel\_Read\|xilinx:adm-pcie-7v3:1ddr:3.0-0\|cnn\_GOOD | Read | OCL Region | 1 | 1 | 146105 | 146106 | 0.00333333 | 4002.279026 | 4002.279029 |
| 4002.279086 | Kernel\_Read\|xilinx:adm-pcie-7v3:1ddr:3.0-0\|cnn\_GOOD | Read | OCL Region | 1 | 1 | 146123 | 146124 | 0.00333333 | 4002.279086 | 4002.279089 |
| 4002.279136 | Kernel\_Read\|xilinx:adm-pcie-7v3:1ddr:3.0-0\|cnn\_GOOD | Read | OCL Region | 1 | 1 | 146138 | 146139 | 0.00333333 | 4002.279136 | 4002.279139 |
| 4002.279186 | Kernel\_Read\|xilinx:adm-pcie-7v3:1ddr:3.0-0\|cnn\_GOOD | Read | OCL Region | 1 | 1 | 146153 | 146154 | 0.00333333 | 4002.279186 | 4002.279189 |
| 4002.279236 | Kernel\_Read\|xilinx:adm-pcie-7v3:1ddr:3.0-0\|cnn\_GOOD | Read | OCL Region | 1 | 1 | 146168 | 146169 | 0.00333333 | 4002.279236 | 4002.279239 |
| 4002.279286 | Kernel\_Read\|xilinx:adm-pcie-7v3:1ddr:3.0-0\|cnn\_GOOD | Read | OCL Region | 1 | 1 | 146183 | 146184 | 0.00333333 | 4002.279286 | 4002.279289 |
| 4002.279336 | Kernel\_Read\|xilinx:adm-pcie-7v3:1ddr:3.0-0\|cnn\_GOOD | Read | OCL Region | 1 | 1 | 146198 | 146199 | 0.00333333 | 4002.279336 | 4002.279339 |
| 4002.279386 | Kernel\_Read\|xilinx:adm-pcie-7v3:1ddr:3.0-0\|cnn\_GOOD | Read | OCL Region | 1 | 1 | 146213 | 146214 | 0.00333333 | 4002.279386 | 4002.279389 |
| 4002.279436 | Kernel\_Read\|xilinx:adm-pcie-7v3:1ddr:3.0-0\|cnn\_GOOD | Read | OCL Region | 1 | 1 | 146228 | 146229 | 0.00333333 | 4002.279436 | 4002.279439 |
| 4002.279486 | Kernel\_Read\|xilinx:adm-pcie-7v3:1ddr:3.0-0\|cnn\_GOOD | Read | OCL Region | 1 | 1 | 146243 | 146244 | 0.00333333 | 4002.279486 | 4002.279489 |
| 4002.279536 | Kernel\_Read\|xilinx:adm-pcie-7v3:1ddr:3.0-0\|cnn\_GOOD | Read | OCL Region | 1 | 1 | 146258 | 146259 | 0.00333333 | 4002.279536 | 4002.279539 |
| 4002.279586 | Kernel\_Read\|xilinx:adm-pcie-7v3:1ddr:3.0-0\|cnn\_GOOD | Read | OCL Region | 1 | 1 | 146273 | 146274 | 0.00333333 | 4002.279586 | 4002.279589 |
| 4002.279636 | Kernel\_Read\|xilinx:adm-pcie-7v3:1ddr:3.0-0\|cnn\_GOOD | Read | OCL Region | 1 | 1 | 146288 | 146289 | 0.00333333 | 4002.279636 | 4002.279639 |
| 4002.279686 | Kernel\_Read\|xilinx:adm-pcie-7v3:1ddr:3.0-0\|cnn\_GOOD | Read | OCL Region | 1 | 1 | 146303 | 146304 | 0.00333333 | 4002.279686 | 4002.279689 |
| 4002.279736 | Kernel\_Read\|xilinx:adm-pcie-7v3:1ddr:3.0-0\|cnn\_GOOD | Read | OCL Region | 1 | 1 | 146318 | 146319 | 0.00333333 | 4002.279736 | 4002.279739 |
| 4002.279786 | Kernel\_Read\|xilinx:adm-pcie-7v3:1ddr:3.0-0\|cnn\_GOOD | Read | OCL Region | 1 | 1 | 146333 | 146334 | 0.00333333 | 4002.279786 | 4002.279789 |
| 4002.279836 | Kernel\_Read\|xilinx:adm-pcie-7v3:1ddr:3.0-0\|cnn\_GOOD | Read | OCL Region | 1 | 1 | 146348 | 146349 | 0.00333333 | 4002.279836 | 4002.279839 |
| 4002.279886 | Kernel\_Read\|xilinx:adm-pcie-7v3:1ddr:3.0-0\|cnn\_GOOD | Read | OCL Region | 1 | 1 | 146363 | 146364 | 0.00333333 | 4002.279886 | 4002.279889 |
| 4002.279936 | Kernel\_Read\|xilinx:adm-pcie-7v3:1ddr:3.0-0\|cnn\_GOOD | Read | OCL Region | 1 | 1 | 146378 | 146379 | 0.00333333 | 4002.279936 | 4002.279939 |
| 4002.279986 | Kernel\_Read\|xilinx:adm-pcie-7v3:1ddr:3.0-0\|cnn\_GOOD | Read | OCL Region | 1 | 1 | 146393 | 146394 | 0.00333333 | 4002.279986 | 4002.279989 |
| 4002.280036 | Kernel\_Read\|xilinx:adm-pcie-7v3:1ddr:3.0-0\|cnn\_GOOD | Read | OCL Region | 1 | 1 | 146408 | 146409 | 0.00333333 | 4002.280036 | 4002.280039 |
| 4002.280096 | Kernel\_Read\|xilinx:adm-pcie-7v3:1ddr:3.0-0\|cnn\_GOOD | Read | OCL Region | 1 | 1 | 146426 | 146427 | 0.00333333 | 4002.280096 | 4002.280099 |
| 4002.280146 | Kernel\_Read\|xilinx:adm-pcie-7v3:1ddr:3.0-0\|cnn\_GOOD | Read | OCL Region | 1 | 1 | 146441 | 146442 | 0.00333333 | 4002.280146 | 4002.280149 |
| 4002.280196 | Kernel\_Read\|xilinx:adm-pcie-7v3:1ddr:3.0-0\|cnn\_GOOD | Read | OCL Region | 1 | 1 | 146456 | 146457 | 0.00333333 | 4002.280196 | 4002.280199 |
| 4002.280246 | Kernel\_Read\|xilinx:adm-pcie-7v3:1ddr:3.0-0\|cnn\_GOOD | Read | OCL Region | 1 | 1 | 146471 | 146472 | 0.00333333 | 4002.280246 | 4002.280249 |
| 4002.280296 | Kernel\_Read\|xilinx:adm-pcie-7v3:1ddr:3.0-0\|cnn\_GOOD | Read | OCL Region | 1 | 1 | 146486 | 146487 | 0.00333333 | 4002.280296 | 4002.280299 |
| 4002.280346 | Kernel\_Read\|xilinx:adm-pcie-7v3:1ddr:3.0-0\|cnn\_GOOD | Read | OCL Region | 1 | 1 | 146501 | 146502 | 0.00333333 | 4002.280346 | 4002.280349 |
| 4002.280396 | Kernel\_Read\|xilinx:adm-pcie-7v3:1ddr:3.0-0\|cnn\_GOOD | Read | OCL Region | 1 | 1 | 146516 | 146517 | 0.00333333 | 4002.280396 | 4002.280399 |
| 4002.280446 | Kernel\_Read\|xilinx:adm-pcie-7v3:1ddr:3.0-0\|cnn\_GOOD | Read | OCL Region | 1 | 1 | 146531 | 146532 | 0.00333333 | 4002.280446 | 4002.280449 |
| 4002.280496 | Kernel\_Read\|xilinx:adm-pcie-7v3:1ddr:3.0-0\|cnn\_GOOD | Read | OCL Region | 1 | 1 | 146546 | 146547 | 0.00333333 | 4002.280496 | 4002.280499 |
| 4002.280546 | Kernel\_Read\|xilinx:adm-pcie-7v3:1ddr:3.0-0\|cnn\_GOOD | Read | OCL Region | 1 | 1 | 146561 | 146562 | 0.00333333 | 4002.280546 | 4002.280549 |
| 4002.280596 | Kernel\_Read\|xilinx:adm-pcie-7v3:1ddr:3.0-0\|cnn\_GOOD | Read | OCL Region | 1 | 1 | 146576 | 146577 | 0.00333333 | 4002.280596 | 4002.280599 |
| 4002.280646 | Kernel\_Read\|xilinx:adm-pcie-7v3:1ddr:3.0-0\|cnn\_GOOD | Read | OCL Region | 1 | 1 | 146591 | 146592 | 0.00333333 | 4002.280646 | 4002.280649 |
| 4002.280696 | Kernel\_Read\|xilinx:adm-pcie-7v3:1ddr:3.0-0\|cnn\_GOOD | Read | OCL Region | 1 | 1 | 146606 | 146607 | 0.00333333 | 4002.280696 | 4002.280699 |
| 4002.280746 | Kernel\_Read\|xilinx:adm-pcie-7v3:1ddr:3.0-0\|cnn\_GOOD | Read | OCL Region | 1 | 1 | 146621 | 146622 | 0.00333333 | 4002.280746 | 4002.280749 |
| 4002.280796 | Kernel\_Read\|xilinx:adm-pcie-7v3:1ddr:3.0-0\|cnn\_GOOD | Read | OCL Region | 1 | 1 | 146636 | 146637 | 0.00333333 | 4002.280796 | 4002.280799 |
| 4002.280846 | Kernel\_Read\|xilinx:adm-pcie-7v3:1ddr:3.0-0\|cnn\_GOOD | Read | OCL Region | 1 | 1 | 146651 | 146652 | 0.00333333 | 4002.280846 | 4002.280849 |
| 4002.280896 | Kernel\_Read\|xilinx:adm-pcie-7v3:1ddr:3.0-0\|cnn\_GOOD | Read | OCL Region | 1 | 1 | 146666 | 146667 | 0.00333333 | 4002.280896 | 4002.280899 |
| 4002.280946 | Kernel\_Read\|xilinx:adm-pcie-7v3:1ddr:3.0-0\|cnn\_GOOD | Read | OCL Region | 1 | 1 | 146681 | 146682 | 0.00333333 | 4002.280946 | 4002.280949 |
| 4002.280996 | Kernel\_Read\|xilinx:adm-pcie-7v3:1ddr:3.0-0\|cnn\_GOOD | Read | OCL Region | 1 | 1 | 146696 | 146697 | 0.00333333 | 4002.280996 | 4002.280999 |
| 4002.281059 | Kernel\_Read\|xilinx:adm-pcie-7v3:1ddr:3.0-0\|cnn\_GOOD | Read | OCL Region | 1 | 1 | 146715 | 146716 | 0.00333333 | 4002.281059 | 4002.281062 |
| 4002.281109 | Kernel\_Read\|xilinx:adm-pcie-7v3:1ddr:3.0-0\|cnn\_GOOD | Read | OCL Region | 1 | 1 | 146730 | 146731 | 0.00333333 | 4002.281109 | 4002.281112 |
| 4002.281159 | Kernel\_Read\|xilinx:adm-pcie-7v3:1ddr:3.0-0\|cnn\_GOOD | Read | OCL Region | 1 | 1 | 146745 | 146746 | 0.00333333 | 4002.281159 | 4002.281162 |
| 4002.281209 | Kernel\_Read\|xilinx:adm-pcie-7v3:1ddr:3.0-0\|cnn\_GOOD | Read | OCL Region | 1 | 1 | 146760 | 146761 | 0.00333333 | 4002.281209 | 4002.281212 |
| 4002.281259 | Kernel\_Read\|xilinx:adm-pcie-7v3:1ddr:3.0-0\|cnn\_GOOD | Read | OCL Region | 1 | 1 | 146775 | 146776 | 0.00333333 | 4002.281259 | 4002.281262 |
| 4002.281309 | Kernel\_Read\|xilinx:adm-pcie-7v3:1ddr:3.0-0\|cnn\_GOOD | Read | OCL Region | 1 | 1 | 146790 | 146791 | 0.00333333 | 4002.281309 | 4002.281312 |
| 4002.281359 | Kernel\_Read\|xilinx:adm-pcie-7v3:1ddr:3.0-0\|cnn\_GOOD | Read | OCL Region | 1 | 1 | 146805 | 146806 | 0.00333333 | 4002.281359 | 4002.281362 |
| 4002.281409 | Kernel\_Read\|xilinx:adm-pcie-7v3:1ddr:3.0-0\|cnn\_GOOD | Read | OCL Region | 1 | 1 | 146820 | 146821 | 0.00333333 | 4002.281409 | 4002.281412 |
| 4002.281459 | Kernel\_Read\|xilinx:adm-pcie-7v3:1ddr:3.0-0\|cnn\_GOOD | Read | OCL Region | 1 | 1 | 146835 | 146836 | 0.00333333 | 4002.281459 | 4002.281462 |
| 4002.281509 | Kernel\_Read\|xilinx:adm-pcie-7v3:1ddr:3.0-0\|cnn\_GOOD | Read | OCL Region | 1 | 1 | 146850 | 146851 | 0.00333333 | 4002.281509 | 4002.281512 |
| 4002.281559 | Kernel\_Read\|xilinx:adm-pcie-7v3:1ddr:3.0-0\|cnn\_GOOD | Read | OCL Region | 1 | 1 | 146865 | 146866 | 0.00333333 | 4002.281559 | 4002.281562 |
| 4002.281609 | Kernel\_Read\|xilinx:adm-pcie-7v3:1ddr:3.0-0\|cnn\_GOOD | Read | OCL Region | 1 | 1 | 146880 | 146881 | 0.00333333 | 4002.281609 | 4002.281612 |
| 4002.281659 | Kernel\_Read\|xilinx:adm-pcie-7v3:1ddr:3.0-0\|cnn\_GOOD | Read | OCL Region | 1 | 1 | 146895 | 146896 | 0.00333333 | 4002.281659 | 4002.281662 |
| 4002.281709 | Kernel\_Read\|xilinx:adm-pcie-7v3:1ddr:3.0-0\|cnn\_GOOD | Read | OCL Region | 1 | 1 | 146910 | 146911 | 0.00333333 | 4002.281709 | 4002.281712 |
| 4002.281759 | Kernel\_Read\|xilinx:adm-pcie-7v3:1ddr:3.0-0\|cnn\_GOOD | Read | OCL Region | 1 | 1 | 146925 | 146926 | 0.00333333 | 4002.281759 | 4002.281762 |
| 4002.281809 | Kernel\_Read\|xilinx:adm-pcie-7v3:1ddr:3.0-0\|cnn\_GOOD | Read | OCL Region | 1 | 1 | 146940 | 146941 | 0.00333333 | 4002.281809 | 4002.281812 |
| 4002.281859 | Kernel\_Read\|xilinx:adm-pcie-7v3:1ddr:3.0-0\|cnn\_GOOD | Read | OCL Region | 1 | 1 | 146955 | 146956 | 0.00333333 | 4002.281859 | 4002.281862 |
| 4002.282126 | Kernel\_Read\|xilinx:adm-pcie-7v3:1ddr:3.0-0\|cnn\_GOOD | Read | OCL Region | 1 | 1 | 147035 | 147036 | 0.00333333 | 4002.282126 | 4002.282129 |
| 4002.282176 | Kernel\_Read\|xilinx:adm-pcie-7v3:1ddr:3.0-0\|cnn\_GOOD | Read | OCL Region | 1 | 1 | 147050 | 147051 | 0.00333333 | 4002.282176 | 4002.282179 |
| 4002.282226 | Kernel\_Read\|xilinx:adm-pcie-7v3:1ddr:3.0-0\|cnn\_GOOD | Read | OCL Region | 1 | 1 | 147065 | 147066 | 0.00333333 | 4002.282226 | 4002.282229 |
| 4002.282286 | Kernel\_Read\|xilinx:adm-pcie-7v3:1ddr:3.0-0\|cnn\_GOOD | Read | OCL Region | 1 | 1 | 147083 | 147084 | 0.00333333 | 4002.282286 | 4002.282289 |
| 4002.282336 | Kernel\_Read\|xilinx:adm-pcie-7v3:1ddr:3.0-0\|cnn\_GOOD | Read | OCL Region | 1 | 1 | 147098 | 147099 | 0.00333333 | 4002.282336 | 4002.282339 |
| 4002.282386 | Kernel\_Read\|xilinx:adm-pcie-7v3:1ddr:3.0-0\|cnn\_GOOD | Read | OCL Region | 1 | 1 | 147113 | 147114 | 0.00333333 | 4002.282386 | 4002.282389 |
| 4002.282436 | Kernel\_Read\|xilinx:adm-pcie-7v3:1ddr:3.0-0\|cnn\_GOOD | Read | OCL Region | 1 | 1 | 147128 | 147129 | 0.00333333 | 4002.282436 | 4002.282439 |
| 4002.282486 | Kernel\_Read\|xilinx:adm-pcie-7v3:1ddr:3.0-0\|cnn\_GOOD | Read | OCL Region | 1 | 1 | 147143 | 147144 | 0.00333333 | 4002.282486 | 4002.282489 |
| 4002.282536 | Kernel\_Read\|xilinx:adm-pcie-7v3:1ddr:3.0-0\|cnn\_GOOD | Read | OCL Region | 1 | 1 | 147158 | 147159 | 0.00333333 | 4002.282536 | 4002.282539 |
| 4002.282586 | Kernel\_Read\|xilinx:adm-pcie-7v3:1ddr:3.0-0\|cnn\_GOOD | Read | OCL Region | 1 | 1 | 147173 | 147174 | 0.00333333 | 4002.282586 | 4002.282589 |
| 4002.282636 | Kernel\_Read\|xilinx:adm-pcie-7v3:1ddr:3.0-0\|cnn\_GOOD | Read | OCL Region | 1 | 1 | 147188 | 147189 | 0.00333333 | 4002.282636 | 4002.282639 |
| 4002.282686 | Kernel\_Read\|xilinx:adm-pcie-7v3:1ddr:3.0-0\|cnn\_GOOD | Read | OCL Region | 1 | 1 | 147203 | 147204 | 0.00333333 | 4002.282686 | 4002.282689 |
| 4002.282736 | Kernel\_Read\|xilinx:adm-pcie-7v3:1ddr:3.0-0\|cnn\_GOOD | Read | OCL Region | 1 | 1 | 147218 | 147219 | 0.00333333 | 4002.282736 | 4002.282739 |
| 4002.282786 | Kernel\_Read\|xilinx:adm-pcie-7v3:1ddr:3.0-0\|cnn\_GOOD | Read | OCL Region | 1 | 1 | 147233 | 147234 | 0.00333333 | 4002.282786 | 4002.282789 |
| 4002.282836 | Kernel\_Read\|xilinx:adm-pcie-7v3:1ddr:3.0-0\|cnn\_GOOD | Read | OCL Region | 1 | 1 | 147248 | 147249 | 0.00333333 | 4002.282836 | 4002.282839 |
| 4002.282886 | Kernel\_Read\|xilinx:adm-pcie-7v3:1ddr:3.0-0\|cnn\_GOOD | Read | OCL Region | 1 | 1 | 147263 | 147264 | 0.00333333 | 4002.282886 | 4002.282889 |
| 4002.282936 | Kernel\_Read\|xilinx:adm-pcie-7v3:1ddr:3.0-0\|cnn\_GOOD | Read | OCL Region | 1 | 1 | 147278 | 147279 | 0.00333333 | 4002.282936 | 4002.282939 |
| 4002.282986 | Kernel\_Read\|xilinx:adm-pcie-7v3:1ddr:3.0-0\|cnn\_GOOD | Read | OCL Region | 1 | 1 | 147293 | 147294 | 0.00333333 | 4002.282986 | 4002.282989 |
| 4002.283036 | Kernel\_Read\|xilinx:adm-pcie-7v3:1ddr:3.0-0\|cnn\_GOOD | Read | OCL Region | 1 | 1 | 147308 | 147309 | 0.00333333 | 4002.283036 | 4002.283039 |
| 4002.283086 | Kernel\_Read\|xilinx:adm-pcie-7v3:1ddr:3.0-0\|cnn\_GOOD | Read | OCL Region | 1 | 1 | 147323 | 147324 | 0.00333333 | 4002.283086 | 4002.283089 |
| 4002.283136 | Kernel\_Read\|xilinx:adm-pcie-7v3:1ddr:3.0-0\|cnn\_GOOD | Read | OCL Region | 1 | 1 | 147338 | 147339 | 0.00333333 | 4002.283136 | 4002.283139 |
| 4002.283196 | Kernel\_Read\|xilinx:adm-pcie-7v3:1ddr:3.0-0\|cnn\_GOOD | Read | OCL Region | 1 | 1 | 147356 | 147357 | 0.00333333 | 4002.283196 | 4002.283199 |
| 4002.283246 | Kernel\_Read\|xilinx:adm-pcie-7v3:1ddr:3.0-0\|cnn\_GOOD | Read | OCL Region | 1 | 1 | 147371 | 147372 | 0.00333333 | 4002.283246 | 4002.283249 |
| 4002.283296 | Kernel\_Read\|xilinx:adm-pcie-7v3:1ddr:3.0-0\|cnn\_GOOD | Read | OCL Region | 1 | 1 | 147386 | 147387 | 0.00333333 | 4002.283296 | 4002.283299 |
| 4002.283346 | Kernel\_Read\|xilinx:adm-pcie-7v3:1ddr:3.0-0\|cnn\_GOOD | Read | OCL Region | 1 | 1 | 147401 | 147402 | 0.00333333 | 4002.283346 | 4002.283349 |
| 4002.283396 | Kernel\_Read\|xilinx:adm-pcie-7v3:1ddr:3.0-0\|cnn\_GOOD | Read | OCL Region | 1 | 1 | 147416 | 147417 | 0.00333333 | 4002.283396 | 4002.283399 |
| 4002.283446 | Kernel\_Read\|xilinx:adm-pcie-7v3:1ddr:3.0-0\|cnn\_GOOD | Read | OCL Region | 1 | 1 | 147431 | 147432 | 0.00333333 | 4002.283446 | 4002.283449 |
| 4002.283496 | Kernel\_Read\|xilinx:adm-pcie-7v3:1ddr:3.0-0\|cnn\_GOOD | Read | OCL Region | 1 | 1 | 147446 | 147447 | 0.00333333 | 4002.283496 | 4002.283499 |
| 4002.283546 | Kernel\_Read\|xilinx:adm-pcie-7v3:1ddr:3.0-0\|cnn\_GOOD | Read | OCL Region | 1 | 1 | 147461 | 147462 | 0.00333333 | 4002.283546 | 4002.283549 |
| 4002.283596 | Kernel\_Read\|xilinx:adm-pcie-7v3:1ddr:3.0-0\|cnn\_GOOD | Read | OCL Region | 1 | 1 | 147476 | 147477 | 0.00333333 | 4002.283596 | 4002.283599 |
| 4002.283646 | Kernel\_Read\|xilinx:adm-pcie-7v3:1ddr:3.0-0\|cnn\_GOOD | Read | OCL Region | 1 | 1 | 147491 | 147492 | 0.00333333 | 4002.283646 | 4002.283649 |
| 4002.283696 | Kernel\_Read\|xilinx:adm-pcie-7v3:1ddr:3.0-0\|cnn\_GOOD | Read | OCL Region | 1 | 1 | 147506 | 147507 | 0.00333333 | 4002.283696 | 4002.283699 |
| 4002.283746 | Kernel\_Read\|xilinx:adm-pcie-7v3:1ddr:3.0-0\|cnn\_GOOD | Read | OCL Region | 1 | 1 | 147521 | 147522 | 0.00333333 | 4002.283746 | 4002.283749 |
| 4002.283796 | Kernel\_Read\|xilinx:adm-pcie-7v3:1ddr:3.0-0\|cnn\_GOOD | Read | OCL Region | 1 | 1 | 147536 | 147537 | 0.00333333 | 4002.283796 | 4002.283799 |
| 4002.283846 | Kernel\_Read\|xilinx:adm-pcie-7v3:1ddr:3.0-0\|cnn\_GOOD | Read | OCL Region | 1 | 1 | 147551 | 147552 | 0.00333333 | 4002.283846 | 4002.283849 |
| 4002.283896 | Kernel\_Read\|xilinx:adm-pcie-7v3:1ddr:3.0-0\|cnn\_GOOD | Read | OCL Region | 1 | 1 | 147566 | 147567 | 0.00333333 | 4002.283896 | 4002.283899 |
| 4002.283946 | Kernel\_Read\|xilinx:adm-pcie-7v3:1ddr:3.0-0\|cnn\_GOOD | Read | OCL Region | 1 | 1 | 147581 | 147582 | 0.00333333 | 4002.283946 | 4002.283949 |
| 4002.283996 | Kernel\_Read\|xilinx:adm-pcie-7v3:1ddr:3.0-0\|cnn\_GOOD | Read | OCL Region | 1 | 1 | 147596 | 147597 | 0.00333333 | 4002.283996 | 4002.283999 |
| 4002.284046 | Kernel\_Read\|xilinx:adm-pcie-7v3:1ddr:3.0-0\|cnn\_GOOD | Read | OCL Region | 1 | 1 | 147611 | 147612 | 0.00333333 | 4002.284046 | 4002.284049 |
| 4002.284096 | Kernel\_Read\|xilinx:adm-pcie-7v3:1ddr:3.0-0\|cnn\_GOOD | Read | OCL Region | 1 | 1 | 147626 | 147627 | 0.00333333 | 4002.284096 | 4002.284099 |
| 4002.284146 | Kernel\_Read\|xilinx:adm-pcie-7v3:1ddr:3.0-0\|cnn\_GOOD | Read | OCL Region | 1 | 1 | 147641 | 147642 | 0.00333333 | 4002.284146 | 4002.284149 |
| 4002.284209 | Kernel\_Read\|xilinx:adm-pcie-7v3:1ddr:3.0-0\|cnn\_GOOD | Read | OCL Region | 1 | 1 | 147660 | 147661 | 0.00333333 | 4002.284209 | 4002.284212 |
| 4002.284259 | Kernel\_Read\|xilinx:adm-pcie-7v3:1ddr:3.0-0\|cnn\_GOOD | Read | OCL Region | 1 | 1 | 147675 | 147676 | 0.00333333 | 4002.284259 | 4002.284262 |
| 4002.284309 | Kernel\_Read\|xilinx:adm-pcie-7v3:1ddr:3.0-0\|cnn\_GOOD | Read | OCL Region | 1 | 1 | 147690 | 147691 | 0.00333333 | 4002.284309 | 4002.284312 |
| 4002.284359 | Kernel\_Read\|xilinx:adm-pcie-7v3:1ddr:3.0-0\|cnn\_GOOD | Read | OCL Region | 1 | 1 | 147705 | 147706 | 0.00333333 | 4002.284359 | 4002.284362 |
| 4002.284409 | Kernel\_Read\|xilinx:adm-pcie-7v3:1ddr:3.0-0\|cnn\_GOOD | Read | OCL Region | 1 | 1 | 147720 | 147721 | 0.00333333 | 4002.284409 | 4002.284412 |
| 4002.284459 | Kernel\_Read\|xilinx:adm-pcie-7v3:1ddr:3.0-0\|cnn\_GOOD | Read | OCL Region | 1 | 1 | 147735 | 147736 | 0.00333333 | 4002.284459 | 4002.284462 |
| 4002.284509 | Kernel\_Read\|xilinx:adm-pcie-7v3:1ddr:3.0-0\|cnn\_GOOD | Read | OCL Region | 1 | 1 | 147750 | 147751 | 0.00333333 | 4002.284509 | 4002.284512 |
| 4002.284559 | Kernel\_Read\|xilinx:adm-pcie-7v3:1ddr:3.0-0\|cnn\_GOOD | Read | OCL Region | 1 | 1 | 147765 | 147766 | 0.00333333 | 4002.284559 | 4002.284562 |
| 4002.284609 | Kernel\_Read\|xilinx:adm-pcie-7v3:1ddr:3.0-0\|cnn\_GOOD | Read | OCL Region | 1 | 1 | 147780 | 147781 | 0.00333333 | 4002.284609 | 4002.284612 |
| 4002.284659 | Kernel\_Read\|xilinx:adm-pcie-7v3:1ddr:3.0-0\|cnn\_GOOD | Read | OCL Region | 1 | 1 | 147795 | 147796 | 0.00333333 | 4002.284659 | 4002.284662 |
| 4002.284709 | Kernel\_Read\|xilinx:adm-pcie-7v3:1ddr:3.0-0\|cnn\_GOOD | Read | OCL Region | 1 | 1 | 147810 | 147811 | 0.00333333 | 4002.284709 | 4002.284712 |
| 4002.284759 | Kernel\_Read\|xilinx:adm-pcie-7v3:1ddr:3.0-0\|cnn\_GOOD | Read | OCL Region | 1 | 1 | 147825 | 147826 | 0.00333333 | 4002.284759 | 4002.284762 |
| 4002.284809 | Kernel\_Read\|xilinx:adm-pcie-7v3:1ddr:3.0-0\|cnn\_GOOD | Read | OCL Region | 1 | 1 | 147840 | 147841 | 0.00333333 | 4002.284809 | 4002.284812 |
| 4002.284859 | Kernel\_Read\|xilinx:adm-pcie-7v3:1ddr:3.0-0\|cnn\_GOOD | Read | OCL Region | 1 | 1 | 147855 | 147856 | 0.00333333 | 4002.284859 | 4002.284862 |
| 4002.284909 | Kernel\_Read\|xilinx:adm-pcie-7v3:1ddr:3.0-0\|cnn\_GOOD | Read | OCL Region | 1 | 1 | 147870 | 147871 | 0.00333333 | 4002.284909 | 4002.284912 |
| 4002.284959 | Kernel\_Read\|xilinx:adm-pcie-7v3:1ddr:3.0-0\|cnn\_GOOD | Read | OCL Region | 1 | 1 | 147885 | 147886 | 0.00333333 | 4002.284959 | 4002.284962 |
| 4002.285009 | Kernel\_Read\|xilinx:adm-pcie-7v3:1ddr:3.0-0\|cnn\_GOOD | Read | OCL Region | 1 | 1 | 147900 | 147901 | 0.00333333 | 4002.285009 | 4002.285012 |
| 4002.285059 | Kernel\_Read\|xilinx:adm-pcie-7v3:1ddr:3.0-0\|cnn\_GOOD | Read | OCL Region | 1 | 1 | 147915 | 147916 | 0.00333333 | 4002.285059 | 4002.285062 |
| 4002.285109 | Kernel\_Read\|xilinx:adm-pcie-7v3:1ddr:3.0-0\|cnn\_GOOD | Read | OCL Region | 1 | 1 | 147930 | 147931 | 0.00333333 | 4002.285109 | 4002.285112 |
| 4002.285159 | Kernel\_Read\|xilinx:adm-pcie-7v3:1ddr:3.0-0\|cnn\_GOOD | Read | OCL Region | 1 | 1 | 147945 | 147946 | 0.00333333 | 4002.285159 | 4002.285162 |
| 4002.285216 | Kernel\_Read\|xilinx:adm-pcie-7v3:1ddr:3.0-0\|cnn\_GOOD | Read | OCL Region | 1 | 1 | 147962 | 147963 | 0.00333333 | 4002.285216 | 4002.285219 |
| 4002.285266 | Kernel\_Read\|xilinx:adm-pcie-7v3:1ddr:3.0-0\|cnn\_GOOD | Read | OCL Region | 1 | 1 | 147977 | 147978 | 0.00333333 | 4002.285266 | 4002.285269 |
| 4002.285316 | Kernel\_Read\|xilinx:adm-pcie-7v3:1ddr:3.0-0\|cnn\_GOOD | Read | OCL Region | 1 | 1 | 147992 | 147993 | 0.00333333 | 4002.285316 | 4002.285319 |
| 4002.285366 | Kernel\_Read\|xilinx:adm-pcie-7v3:1ddr:3.0-0\|cnn\_GOOD | Read | OCL Region | 1 | 1 | 148007 | 148008 | 0.00333333 | 4002.285366 | 4002.285369 |
| 4002.285416 | Kernel\_Read\|xilinx:adm-pcie-7v3:1ddr:3.0-0\|cnn\_GOOD | Read | OCL Region | 1 | 1 | 148022 | 148023 | 0.00333333 | 4002.285416 | 4002.285419 |
| 4002.285466 | Kernel\_Read\|xilinx:adm-pcie-7v3:1ddr:3.0-0\|cnn\_GOOD | Read | OCL Region | 1 | 1 | 148037 | 148038 | 0.00333333 | 4002.285466 | 4002.285469 |
| 4002.285516 | Kernel\_Read\|xilinx:adm-pcie-7v3:1ddr:3.0-0\|cnn\_GOOD | Read | OCL Region | 1 | 1 | 148052 | 148053 | 0.00333333 | 4002.285516 | 4002.285519 |
| 4002.285566 | Kernel\_Read\|xilinx:adm-pcie-7v3:1ddr:3.0-0\|cnn\_GOOD | Read | OCL Region | 1 | 1 | 148067 | 148068 | 0.00333333 | 4002.285566 | 4002.285569 |
| 4002.285616 | Kernel\_Read\|xilinx:adm-pcie-7v3:1ddr:3.0-0\|cnn\_GOOD | Read | OCL Region | 1 | 1 | 148082 | 148083 | 0.00333333 | 4002.285616 | 4002.285619 |
| 4002.285666 | Kernel\_Read\|xilinx:adm-pcie-7v3:1ddr:3.0-0\|cnn\_GOOD | Read | OCL Region | 1 | 1 | 148097 | 148098 | 0.00333333 | 4002.285666 | 4002.285669 |
| 4002.285716 | Kernel\_Read\|xilinx:adm-pcie-7v3:1ddr:3.0-0\|cnn\_GOOD | Read | OCL Region | 1 | 1 | 148112 | 148113 | 0.00333333 | 4002.285716 | 4002.285719 |
| 4002.285766 | Kernel\_Read\|xilinx:adm-pcie-7v3:1ddr:3.0-0\|cnn\_GOOD | Read | OCL Region | 1 | 1 | 148127 | 148128 | 0.00333333 | 4002.285766 | 4002.285769 |
| 4002.285816 | Kernel\_Read\|xilinx:adm-pcie-7v3:1ddr:3.0-0\|cnn\_GOOD | Read | OCL Region | 1 | 1 | 148142 | 148143 | 0.00333333 | 4002.285816 | 4002.285819 |
| 4002.285866 | Kernel\_Read\|xilinx:adm-pcie-7v3:1ddr:3.0-0\|cnn\_GOOD | Read | OCL Region | 1 | 1 | 148157 | 148158 | 0.00333333 | 4002.285866 | 4002.285869 |
| 4002.285916 | Kernel\_Read\|xilinx:adm-pcie-7v3:1ddr:3.0-0\|cnn\_GOOD | Read | OCL Region | 1 | 1 | 148172 | 148173 | 0.00333333 | 4002.285916 | 4002.285919 |
| 4002.285966 | Kernel\_Read\|xilinx:adm-pcie-7v3:1ddr:3.0-0\|cnn\_GOOD | Read | OCL Region | 1 | 1 | 148187 | 148188 | 0.00333333 | 4002.285966 | 4002.285969 |
| 4002.286016 | Kernel\_Read\|xilinx:adm-pcie-7v3:1ddr:3.0-0\|cnn\_GOOD | Read | OCL Region | 1 | 1 | 148202 | 148203 | 0.00333333 | 4002.286016 | 4002.286019 |
| 4002.286066 | Kernel\_Read\|xilinx:adm-pcie-7v3:1ddr:3.0-0\|cnn\_GOOD | Read | OCL Region | 1 | 1 | 148217 | 148218 | 0.00333333 | 4002.286066 | 4002.286069 |
| 4002.286116 | Kernel\_Read\|xilinx:adm-pcie-7v3:1ddr:3.0-0\|cnn\_GOOD | Read | OCL Region | 1 | 1 | 148232 | 148233 | 0.00333333 | 4002.286116 | 4002.286119 |
| 4002.286176 | Kernel\_Read\|xilinx:adm-pcie-7v3:1ddr:3.0-0\|cnn\_GOOD | Read | OCL Region | 1 | 1 | 148250 | 148251 | 0.00333333 | 4002.286176 | 4002.286179 |
| 4002.286226 | Kernel\_Read\|xilinx:adm-pcie-7v3:1ddr:3.0-0\|cnn\_GOOD | Read | OCL Region | 1 | 1 | 148265 | 148266 | 0.00333333 | 4002.286226 | 4002.286229 |
| 4002.286276 | Kernel\_Read\|xilinx:adm-pcie-7v3:1ddr:3.0-0\|cnn\_GOOD | Read | OCL Region | 1 | 1 | 148280 | 148281 | 0.00333333 | 4002.286276 | 4002.286279 |
| 4002.286326 | Kernel\_Read\|xilinx:adm-pcie-7v3:1ddr:3.0-0\|cnn\_GOOD | Read | OCL Region | 1 | 1 | 148295 | 148296 | 0.00333333 | 4002.286326 | 4002.286329 |
| 4002.286376 | Kernel\_Read\|xilinx:adm-pcie-7v3:1ddr:3.0-0\|cnn\_GOOD | Read | OCL Region | 1 | 1 | 148310 | 148311 | 0.00333333 | 4002.286376 | 4002.286379 |
| 4002.286426 | Kernel\_Read\|xilinx:adm-pcie-7v3:1ddr:3.0-0\|cnn\_GOOD | Read | OCL Region | 1 | 1 | 148325 | 148326 | 0.00333333 | 4002.286426 | 4002.286429 |
| 4002.286476 | Kernel\_Read\|xilinx:adm-pcie-7v3:1ddr:3.0-0\|cnn\_GOOD | Read | OCL Region | 1 | 1 | 148340 | 148341 | 0.00333333 | 4002.286476 | 4002.286479 |
| 4002.286526 | Kernel\_Read\|xilinx:adm-pcie-7v3:1ddr:3.0-0\|cnn\_GOOD | Read | OCL Region | 1 | 1 | 148355 | 148356 | 0.00333333 | 4002.286526 | 4002.286529 |
| 4002.286576 | Kernel\_Read\|xilinx:adm-pcie-7v3:1ddr:3.0-0\|cnn\_GOOD | Read | OCL Region | 1 | 1 | 148370 | 148371 | 0.00333333 | 4002.286576 | 4002.286579 |
| 4002.286626 | Kernel\_Read\|xilinx:adm-pcie-7v3:1ddr:3.0-0\|cnn\_GOOD | Read | OCL Region | 1 | 1 | 148385 | 148386 | 0.00333333 | 4002.286626 | 4002.286629 |
| 4002.286676 | Kernel\_Read\|xilinx:adm-pcie-7v3:1ddr:3.0-0\|cnn\_GOOD | Read | OCL Region | 1 | 1 | 148400 | 148401 | 0.00333333 | 4002.286676 | 4002.286679 |
| 4002.286726 | Kernel\_Read\|xilinx:adm-pcie-7v3:1ddr:3.0-0\|cnn\_GOOD | Read | OCL Region | 1 | 1 | 148415 | 148416 | 0.00333333 | 4002.286726 | 4002.286729 |
| 4002.286776 | Kernel\_Read\|xilinx:adm-pcie-7v3:1ddr:3.0-0\|cnn\_GOOD | Read | OCL Region | 1 | 1 | 148430 | 148431 | 0.00333333 | 4002.286776 | 4002.286779 |
| 4002.286826 | Kernel\_Read\|xilinx:adm-pcie-7v3:1ddr:3.0-0\|cnn\_GOOD | Read | OCL Region | 1 | 1 | 148445 | 148446 | 0.00333333 | 4002.286826 | 4002.286829 |
| 4002.286876 | Kernel\_Read\|xilinx:adm-pcie-7v3:1ddr:3.0-0\|cnn\_GOOD | Read | OCL Region | 1 | 1 | 148460 | 148461 | 0.00333333 | 4002.286876 | 4002.286879 |
| 4002.286926 | Kernel\_Read\|xilinx:adm-pcie-7v3:1ddr:3.0-0\|cnn\_GOOD | Read | OCL Region | 1 | 1 | 148475 | 148476 | 0.00333333 | 4002.286926 | 4002.286929 |
| 4002.286976 | Kernel\_Read\|xilinx:adm-pcie-7v3:1ddr:3.0-0\|cnn\_GOOD | Read | OCL Region | 1 | 1 | 148490 | 148491 | 0.00333333 | 4002.286976 | 4002.286979 |
| 4002.287026 | Kernel\_Read\|xilinx:adm-pcie-7v3:1ddr:3.0-0\|cnn\_GOOD | Read | OCL Region | 1 | 1 | 148505 | 148506 | 0.00333333 | 4002.287026 | 4002.287029 |
| 4002.287076 | Kernel\_Read\|xilinx:adm-pcie-7v3:1ddr:3.0-0\|cnn\_GOOD | Read | OCL Region | 1 | 1 | 148520 | 148521 | 0.00333333 | 4002.287076 | 4002.287079 |
| 4002.287126 | Kernel\_Read\|xilinx:adm-pcie-7v3:1ddr:3.0-0\|cnn\_GOOD | Read | OCL Region | 1 | 1 | 148535 | 148536 | 0.00333333 | 4002.287126 | 4002.287129 |
| 4002.287186 | Kernel\_Read\|xilinx:adm-pcie-7v3:1ddr:3.0-0\|cnn\_GOOD | Read | OCL Region | 1 | 1 | 148553 | 148554 | 0.00333333 | 4002.287186 | 4002.287189 |
| 4002.287236 | Kernel\_Read\|xilinx:adm-pcie-7v3:1ddr:3.0-0\|cnn\_GOOD | Read | OCL Region | 1 | 1 | 148568 | 148569 | 0.00333333 | 4002.287236 | 4002.287239 |
| 4002.287286 | Kernel\_Read\|xilinx:adm-pcie-7v3:1ddr:3.0-0\|cnn\_GOOD | Read | OCL Region | 1 | 1 | 148583 | 148584 | 0.00333333 | 4002.287286 | 4002.287289 |
| 4002.287336 | Kernel\_Read\|xilinx:adm-pcie-7v3:1ddr:3.0-0\|cnn\_GOOD | Read | OCL Region | 1 | 1 | 148598 | 148599 | 0.00333333 | 4002.287336 | 4002.287339 |
| 4002.287386 | Kernel\_Read\|xilinx:adm-pcie-7v3:1ddr:3.0-0\|cnn\_GOOD | Read | OCL Region | 1 | 1 | 148613 | 148614 | 0.00333333 | 4002.287386 | 4002.287389 |
| 4002.287436 | Kernel\_Read\|xilinx:adm-pcie-7v3:1ddr:3.0-0\|cnn\_GOOD | Read | OCL Region | 1 | 1 | 148628 | 148629 | 0.00333333 | 4002.287436 | 4002.287439 |
| 4002.287486 | Kernel\_Read\|xilinx:adm-pcie-7v3:1ddr:3.0-0\|cnn\_GOOD | Read | OCL Region | 1 | 1 | 148643 | 148644 | 0.00333333 | 4002.287486 | 4002.287489 |
| 4002.287536 | Kernel\_Read\|xilinx:adm-pcie-7v3:1ddr:3.0-0\|cnn\_GOOD | Read | OCL Region | 1 | 1 | 148658 | 148659 | 0.00333333 | 4002.287536 | 4002.287539 |
| 4002.287586 | Kernel\_Read\|xilinx:adm-pcie-7v3:1ddr:3.0-0\|cnn\_GOOD | Read | OCL Region | 1 | 1 | 148673 | 148674 | 0.00333333 | 4002.287586 | 4002.287589 |
| 4002.287636 | Kernel\_Read\|xilinx:adm-pcie-7v3:1ddr:3.0-0\|cnn\_GOOD | Read | OCL Region | 1 | 1 | 148688 | 148689 | 0.00333333 | 4002.287636 | 4002.287639 |
| 4002.287686 | Kernel\_Read\|xilinx:adm-pcie-7v3:1ddr:3.0-0\|cnn\_GOOD | Read | OCL Region | 1 | 1 | 148703 | 148704 | 0.00333333 | 4002.287686 | 4002.287689 |
| 4002.287736 | Kernel\_Read\|xilinx:adm-pcie-7v3:1ddr:3.0-0\|cnn\_GOOD | Read | OCL Region | 1 | 1 | 148718 | 148719 | 0.00333333 | 4002.287736 | 4002.287739 |
| 4002.287786 | Kernel\_Read\|xilinx:adm-pcie-7v3:1ddr:3.0-0\|cnn\_GOOD | Read | OCL Region | 1 | 1 | 148733 | 148734 | 0.00333333 | 4002.287786 | 4002.287789 |
| 4002.287836 | Kernel\_Read\|xilinx:adm-pcie-7v3:1ddr:3.0-0\|cnn\_GOOD | Read | OCL Region | 1 | 1 | 148748 | 148749 | 0.00333333 | 4002.287836 | 4002.287839 |
| 4002.287886 | Kernel\_Read\|xilinx:adm-pcie-7v3:1ddr:3.0-0\|cnn\_GOOD | Read | OCL Region | 1 | 1 | 148763 | 148764 | 0.00333333 | 4002.287886 | 4002.287889 |
| 4002.287936 | Kernel\_Read\|xilinx:adm-pcie-7v3:1ddr:3.0-0\|cnn\_GOOD | Read | OCL Region | 1 | 1 | 148778 | 148779 | 0.00333333 | 4002.287936 | 4002.287939 |
| 4002.287986 | Kernel\_Read\|xilinx:adm-pcie-7v3:1ddr:3.0-0\|cnn\_GOOD | Read | OCL Region | 1 | 1 | 148793 | 148794 | 0.00333333 | 4002.287986 | 4002.287989 |
| 4002.288036 | Kernel\_Read\|xilinx:adm-pcie-7v3:1ddr:3.0-0\|cnn\_GOOD | Read | OCL Region | 1 | 1 | 148808 | 148809 | 0.00333333 | 4002.288036 | 4002.288039 |
| 4002.288086 | Kernel\_Read\|xilinx:adm-pcie-7v3:1ddr:3.0-0\|cnn\_GOOD | Read | OCL Region | 1 | 1 | 148823 | 148824 | 0.00333333 | 4002.288086 | 4002.288089 |
| 4002.288366 | Kernel\_Read\|xilinx:adm-pcie-7v3:1ddr:3.0-0\|cnn\_GOOD | Read | OCL Region | 1 | 1 | 148907 | 148908 | 0.00333333 | 4002.288366 | 4002.288369 |
| 4002.288416 | Kernel\_Read\|xilinx:adm-pcie-7v3:1ddr:3.0-0\|cnn\_GOOD | Read | OCL Region | 1 | 1 | 148922 | 148923 | 0.00333333 | 4002.288416 | 4002.288419 |
| 4002.288469 | Kernel\_Read\|xilinx:adm-pcie-7v3:1ddr:3.0-0\|cnn\_GOOD | Read | OCL Region | 1 | 1 | 148938 | 148939 | 0.00333333 | 4002.288469 | 4002.288472 |
| 4002.288519 | Kernel\_Read\|xilinx:adm-pcie-7v3:1ddr:3.0-0\|cnn\_GOOD | Read | OCL Region | 1 | 1 | 148953 | 148954 | 0.00333333 | 4002.288519 | 4002.288522 |
| 4002.288569 | Kernel\_Read\|xilinx:adm-pcie-7v3:1ddr:3.0-0\|cnn\_GOOD | Read | OCL Region | 1 | 1 | 148968 | 148969 | 0.00333333 | 4002.288569 | 4002.288572 |
| 4002.288619 | Kernel\_Read\|xilinx:adm-pcie-7v3:1ddr:3.0-0\|cnn\_GOOD | Read | OCL Region | 1 | 1 | 148983 | 148984 | 0.00333333 | 4002.288619 | 4002.288622 |
| 4002.288669 | Kernel\_Read\|xilinx:adm-pcie-7v3:1ddr:3.0-0\|cnn\_GOOD | Read | OCL Region | 1 | 1 | 148998 | 148999 | 0.00333333 | 4002.288669 | 4002.288672 |
| 4002.288719 | Kernel\_Read\|xilinx:adm-pcie-7v3:1ddr:3.0-0\|cnn\_GOOD | Read | OCL Region | 1 | 1 | 149013 | 149014 | 0.00333333 | 4002.288719 | 4002.288722 |
| 4002.288769 | Kernel\_Read\|xilinx:adm-pcie-7v3:1ddr:3.0-0\|cnn\_GOOD | Read | OCL Region | 1 | 1 | 149028 | 149029 | 0.00333333 | 4002.288769 | 4002.288772 |
| 4002.288819 | Kernel\_Read\|xilinx:adm-pcie-7v3:1ddr:3.0-0\|cnn\_GOOD | Read | OCL Region | 1 | 1 | 149043 | 149044 | 0.00333333 | 4002.288819 | 4002.288822 |
| 4002.288869 | Kernel\_Read\|xilinx:adm-pcie-7v3:1ddr:3.0-0\|cnn\_GOOD | Read | OCL Region | 1 | 1 | 149058 | 149059 | 0.00333333 | 4002.288869 | 4002.288872 |
| 4002.288919 | Kernel\_Read\|xilinx:adm-pcie-7v3:1ddr:3.0-0\|cnn\_GOOD | Read | OCL Region | 1 | 1 | 149073 | 149074 | 0.00333333 | 4002.288919 | 4002.288922 |
| 4002.288969 | Kernel\_Read\|xilinx:adm-pcie-7v3:1ddr:3.0-0\|cnn\_GOOD | Read | OCL Region | 1 | 1 | 149088 | 149089 | 0.00333333 | 4002.288969 | 4002.288972 |
| 4002.289019 | Kernel\_Read\|xilinx:adm-pcie-7v3:1ddr:3.0-0\|cnn\_GOOD | Read | OCL Region | 1 | 1 | 149103 | 149104 | 0.00333333 | 4002.289019 | 4002.289022 |
| 4002.289069 | Kernel\_Read\|xilinx:adm-pcie-7v3:1ddr:3.0-0\|cnn\_GOOD | Read | OCL Region | 1 | 1 | 149118 | 149119 | 0.00333333 | 4002.289069 | 4002.289072 |
| 4002.289119 | Kernel\_Read\|xilinx:adm-pcie-7v3:1ddr:3.0-0\|cnn\_GOOD | Read | OCL Region | 1 | 1 | 149133 | 149134 | 0.00333333 | 4002.289119 | 4002.289122 |
| 4002.289169 | Kernel\_Read\|xilinx:adm-pcie-7v3:1ddr:3.0-0\|cnn\_GOOD | Read | OCL Region | 1 | 1 | 149148 | 149149 | 0.00333333 | 4002.289169 | 4002.289172 |
| 4002.289219 | Kernel\_Read\|xilinx:adm-pcie-7v3:1ddr:3.0-0\|cnn\_GOOD | Read | OCL Region | 1 | 1 | 149163 | 149164 | 0.00333333 | 4002.289219 | 4002.289222 |
| 4002.289269 | Kernel\_Read\|xilinx:adm-pcie-7v3:1ddr:3.0-0\|cnn\_GOOD | Read | OCL Region | 1 | 1 | 149178 | 149179 | 0.00333333 | 4002.289269 | 4002.289272 |
| 4002.289332 | Kernel\_Read\|xilinx:adm-pcie-7v3:1ddr:3.0-0\|cnn\_GOOD | Read | OCL Region | 1 | 1 | 149197 | 149198 | 0.00333333 | 4002.289332 | 4002.289336 |
