# Supplementary material for: Research on OpenCL optimization for FPGA deep learning application
Source: PLoS One. 2019 Oct 10;14(10):e0222984. doi: 10.1371/journal.pone.0222984 (PMC6786543; doi:10.1371/journal.pone.0222984)
Supplement: S1 File — (Xilinx and this paper). (ZIP) [file pone.0222984.s001.zip › SupportingFiles/Xlinx original data.zip!/Xlinx original data/sdaccel_profile_summary.html]

# SDAccel Profile Summary

  

### Generated on: 2019-02-02 00:51:07

### Profiled application: Xilinx\_fpgacnn\_best.exe

### Target platform: Xilinx

### Target devices: xilinx:adm-pcie-7v3:1ddr:3.0-0

### Flow mode: System Run

### Tool version: 2016.3

  
  

## OpenCL API Calls

| API Name | Number Of Calls | Total Time (ms) | Minimum Time (ms) | Average Time (ms) | Maximum Time (ms) |
| --- | --- | --- | --- | --- | --- |
| clCreateProgramWithBinary | 1 | 2288.47 | 2288.47 | 2288.47 | 2288.47 |
| clCreateContext | 1 | 1701.63 | 1701.63 | 1701.63 | 1701.63 |
| clFinish | 1 | 292.491 | 292.491 | 292.491 | 292.491 |
| clEnqueueWriteBuffer | 2 | 1.44548 | 0.361833 | 0.722739 | 1.08365 |
| clEnqueueReadBuffer | 1 | 0.831335 | 0.831335 | 0.831335 | 0.831335 |
| clGetPlatformIDs | 3 | 0.119324 | 0.008827 | 0.0397747 | 0.066607 |
| clSetKernelArg | 6 | 0.08021 | 0.009669 | 0.0133683 | 0.027591 |
| clCreateBuffer | 3 | 0.070162 | 0.014643 | 0.0233873 | 0.038768 |
| clEnqueueNDRangeKernel | 1 | 0.041138 | 0.041138 | 0.041138 | 0.041138 |
| clReleaseMemObject | 3 | 0.040184 | 0.00977 | 0.0133947 | 0.02058 |
| clCreateKernel | 1 | 0.02947 | 0.02947 | 0.02947 | 0.02947 |
| clGetEventProfilingInfo | 2 | 0.021922 | 0.009161 | 0.010961 | 0.012761 |
| clGetDeviceInfo | 2 | 0.020414 | 0.008489 | 0.010207 | 0.011925 |
| clGetPlatformInfo | 2 | 0.019594 | 0.008493 | 0.009797 | 0.011101 |
| clBuildProgram | 1 | 0.0181 | 0.0181 | 0.0181 | 0.0181 |
| clCreateCommandQueue | 1 | 0.014736 | 0.014736 | 0.014736 | 0.014736 |
| clReleaseProgram | 1 | 0.011084 | 0.011084 | 0.011084 | 0.011084 |
| clReleaseKernel | 1 | 0.011003 | 0.011003 | 0.011003 | 0.011003 |
| clReleaseCommandQueue | 1 | 0.010915 | 0.010915 | 0.010915 | 0.010915 |
| clReleaseContext | 1 | 0.010687 | 0.010687 | 0.010687 | 0.010687 |
| clGetDeviceIDs | 1 | 0.010335 | 0.010335 | 0.010335 | 0.010335 |

  

## Kernel Execution

| Kernel | Number Of Enqueues | Total Time (ms) | Minimum Time (ms) | Average Time (ms) | Maximum Time (ms) |
| --- | --- | --- | --- | --- | --- |
| good\_cnn | 1 | 291.977 | 291.977 | 291.977 | 291.977 |

  

## Compute Unit Utilization

| Device | Compute Unit | Kernel | Global Work Size | Local Work Size | Number Of Calls | Total Time (ms) | Minimum Time (ms) | Average Time (ms) | Maximum Time (ms) |
| --- | --- | --- | --- | --- | --- | --- | --- | --- | --- |
| xilinx:adm-pcie-7v3:1ddr:3.0-0 | good\_cnn\_1 | good\_cnn | 4:1:1 | 1:1:1 | 1 | 290.751 | 290.751 | 290.751 | 290.751 |
| xilinx:adm-pcie-7v3:1ddr:3.0-0 | good\_cnn\_2 | good\_cnn | 4:1:1 | 1:1:1 | 1 | 290.859 | 290.859 | 290.859 | 290.859 |
| xilinx:adm-pcie-7v3:1ddr:3.0-0 | good\_cnn\_3 | good\_cnn | 4:1:1 | 1:1:1 | 1 | 291.721 | 291.721 | 291.721 | 291.721 |
| xilinx:adm-pcie-7v3:1ddr:3.0-0 | good\_cnn\_4 | good\_cnn | 4:1:1 | 1:1:1 | 1 | 291.684 | 291.684 | 291.684 | 291.684 |

  

## Data Transfer: Host and Global Memory

| Context:Number of Devices | Transfer Type | Number Of Transfers | Transfer Rate (MB/s) | Average Bandwidth Utilization (%) | Average Size (KB) | Total Time (ms) | Average Time (ms) |
| --- | --- | --- | --- | --- | --- | --- | --- |
| context0:1 | READ | 1 | 1152.869990 | 12.009062 | 746.496 | 0.647511 | 0.647511 |
| context0:1 | WRITE | 2 | 1376.163503 | 14.335036 | 684.384 | 0.994626 | 0.497313 |

  

## Data Transfer: Kernels and Global Memory

| Device | Transfer Type | Number Of Transfers | Transfer Rate (MB/s) | Average Bandwidth Utilization (%) | Average Size (KB) | Average Time (ns) |
| --- | --- | --- | --- | --- | --- | --- |
| xilinx:adm-pcie-7v3:1ddr:3.0-0 | READ | 27948 | 6.12607 | 0.0531777 | 0.064 | 686.174 |
| xilinx:adm-pcie-7v3:1ddr:3.0-0 | WRITE | 11863 | 2.55669 | 0.0221935 | 0.0629264 | 59.989 |

  

## Top Data Transfer: Kernels and Global Memory

| Device | Kernel Name | Number of Transfers | Average Bytes per Transfer | Transfer Efficiency (%) | Total Data Transfer (MB) | Total Write (MB) | Total Read (MB) | Transfer Rate (MB/s) | Average Bandwidth Utilization (%) |
| --- | --- | --- | --- | --- | --- | --- | --- | --- | --- |
| xilinx:adm-pcie-7v3:1ddr:3.0-0 | ALL | 39811 | 63.6801 | 1.55469 | 2.53517 | 0.746496 | 1.78867 | 4.34138 | 0.0376856 |
