# Supplementary material for: Research on OpenCL optimization for FPGA deep learning application
Source: PLoS One. 2019 Oct 10;14(10):e0222984. doi: 10.1371/journal.pone.0222984 (PMC6786543; doi:10.1371/journal.pone.0222984)
Supplement: S1 File — (Xilinx and this paper). (ZIP) [file pone.0222984.s001.zip › SupportingFiles/ optimized data.zip!/ optimized data/sdaccel_timeline_trace.html]

# SDAccel Timeline Trace

  

### Generated on: 2018-02-01 14:59:09

### Profiled application: Xilinx\_fpgacnn\_burst.exe

### Target platform: Xilinx

  

| Time (msec) | Name | Event | Address/Port | Size (Bytes or Num) | Latency (cycles) | Start (cycles) | End (cycles) | Latency (usec) | Start (msec) | End (msec) |
| --- | --- | --- | --- | --- | --- | --- | --- | --- | --- | --- |
| 0.765968 | clGetPlatformIDs\|General | START |  |  |  |  |  |  |  |  |
| 0.820173 | clGetPlatformIDs\|General | END |  |  |  |  |  |  |  |  |
| 0.834849 | clGetPlatformIDs\|General | START |  |  |  |  |  |  |  |  |
| 0.845779 | clGetPlatformIDs\|General | END |  |  |  |  |  |  |  |  |
| 0.857921 | clGetPlatformInfo\|General | START |  |  |  |  |  |  |  |  |
| 0.872151 | clGetPlatformInfo\|General | END |  |  |  |  |  |  |  |  |
| 0.883407 | clGetPlatformInfo\|General | START |  |  |  |  |  |  |  |  |
| 0.894472 | clGetPlatformInfo\|General | END |  |  |  |  |  |  |  |  |
| 0.914704 | clGetDeviceIDs\|General | START |  |  |  |  |  |  |  |  |
| 0.928382 | clGetDeviceIDs\|General | END |  |  |  |  |  |  |  |  |
| 0.943624 | clGetDeviceInfo\|General | START |  |  |  |  |  |  |  |  |
| 0.958746 | clGetDeviceInfo\|General | END |  |  |  |  |  |  |  |  |
| 0.970016 | clGetDeviceInfo\|General | START |  |  |  |  |  |  |  |  |
| 0.980994 | clGetDeviceInfo\|General | END |  |  |  |  |  |  |  |  |
| 0.995723 | clCreateContext\|General | START |  |  |  |  |  |  |  |  |
| 2026.910888 | clGetPlatformIDs\|General | START |  |  |  |  |  |  |  |  |
| 2026.967954 | clGetPlatformIDs\|General | END |  |  |  |  |  |  |  |  |
| 2027.002874 | clCreateContext\|General | END |  |  |  |  |  |  |  |  |
| 2027.030156 | clCreateCommandQueue\|General | START |  |  |  |  |  |  |  |  |
| 2027.05787 | clCreateCommandQueue\|General | END |  |  |  |  |  |  |  |  |
| 2046.223792 | clCreateProgramWithBinary\|General | START |  |  |  |  |  |  |  |  |
| 5268.175284 | clCreateProgramWithBinary\|General | END |  |  |  |  |  |  |  |  |
| 5268.273206 | clBuildProgram\|General | START |  |  |  |  |  |  |  |  |
| 5268.306176 | clBuildProgram\|General | END |  |  |  |  |  |  |  |  |
| 5268.902036 | clCreateKernel\|General | START |  |  |  |  |  |  |  |  |
| 5268.957999 | clCreateKernel\|General | END |  |  |  |  |  |  |  |  |
| 5269.027205 | clCreateBuffer\|General | START |  |  |  |  |  |  |  |  |
| 5269.093651 | clCreateBuffer\|General | END |  |  |  |  |  |  |  |  |
| 5269.118127 | clCreateBuffer\|General | START |  |  |  |  |  |  |  |  |
| 5269.153537 | clCreateBuffer\|General | END |  |  |  |  |  |  |  |  |
| 5269.176471 | clCreateBuffer\|General | START |  |  |  |  |  |  |  |  |
| 5269.203926 | clCreateBuffer\|General | END |  |  |  |  |  |  |  |  |
| 5269.254493 | clEnqueueWriteBuffer\|30303368 | START |  |  |  |  |  |  |  |  |
| 5269.323079 | WRITE\_BUFFER | QUEUE | 0X1CE6F50 | 139968 |  |  |  |  |  |  |
| 5269.413971 | WRITE\_BUFFER | SUBMIT | 0X1CE6F50 | 139968 |  |  |  |  |  |  |
| 5269.600187 | WRITE\_BUFFER | START | 0X1CE6F50 | 139968 |  |  |  |  |  |  |
| 5269.841091 | WRITE\_BUFFER | END | 0X1CE6F50 | 139968 |  |  |  |  |  |  |
| 5269.914125 | clEnqueueWriteBuffer\|30303368 | END |  |  |  |  |  |  |  |  |
| 5269.970107 | clEnqueueWriteBuffer\|30303368 | START |  |  |  |  |  |  |  |  |
| 5270.00491 | WRITE\_BUFFER | QUEUE | 0X1CFFB30 | 1228800 |  |  |  |  |  |  |
| 5270.050166 | WRITE\_BUFFER | SUBMIT | 0X1CFFB30 | 1228800 |  |  |  |  |  |  |
| 5270.182102 | WRITE\_BUFFER | START | 0X1CFFB30 | 1228800 |  |  |  |  |  |  |
| 5271.73461 | WRITE\_BUFFER | END | 0X1CFFB30 | 1228800 |  |  |  |  |  |  |
| 5271.782759 | clEnqueueWriteBuffer\|30303368 | END |  |  |  |  |  |  |  |  |
| 5271.849169 | clSetKernelArg\|General | START |  |  |  |  |  |  |  |  |
| 5271.881984 | clSetKernelArg\|General | END |  |  |  |  |  |  |  |  |
| 5271.903631 | clSetKernelArg\|General | START |  |  |  |  |  |  |  |  |
| 5271.924548 | clSetKernelArg\|General | END |  |  |  |  |  |  |  |  |
| 5271.951001 | clSetKernelArg\|General | START |  |  |  |  |  |  |  |  |
| 5271.971694 | clSetKernelArg\|General | END |  |  |  |  |  |  |  |  |
| 5271.991087 | clSetKernelArg\|General | START |  |  |  |  |  |  |  |  |
| 5272.011113 | clSetKernelArg\|General | END |  |  |  |  |  |  |  |  |
| 5272.030677 | clSetKernelArg\|General | START |  |  |  |  |  |  |  |  |
| 5272.050653 | clSetKernelArg\|General | END |  |  |  |  |  |  |  |  |
| 5272.069407 | clSetKernelArg\|General | START |  |  |  |  |  |  |  |  |
| 5272.08914 | clSetKernelArg\|General | END |  |  |  |  |  |  |  |  |
| 5272.201569 | clEnqueueNDRangeKernel\|30303368 | START |  |  |  |  |  |  |  |  |
| 5272.310328 | clEnqueueNDRangeKernel\|30303368 | END |  |  |  |  |  |  |  |  |
| 5272.341671 | clFinish\|General | START |  |  |  |  |  |  |  |  |
| 5273.055309 | KERNEL\|xilinx:adm-pcie-7v3:1ddr:3.0-0\|cnn\_GOOD\|good\_cnn\|1:1:1\|good\_cnn\_1 | START | 0X1D009D8 | 1 |  |  |  |  |  |  |
| 5273.297912 | KERNEL\|xilinx:adm-pcie-7v3:1ddr:3.0-0\|cnn\_GOOD\|good\_cnn\|1:1:1\|good\_cnn\_2 | START | 0X1D009D8 | 1 |  |  |  |  |  |  |
| 5273.435841 | KERNEL\|xilinx:adm-pcie-7v3:1ddr:3.0-0\|cnn\_GOOD\|good\_cnn\|1:1:1\|good\_cnn\_3 | START | 0X1D009D8 | 1 |  |  |  |  |  |  |
| 5273.525074 | KERNEL\|xilinx:adm-pcie-7v3:1ddr:3.0-0\|cnn\_GOOD\|good\_cnn\|1:1:1\|good\_cnn\_4 | START | 0X1D009D8 | 1 |  |  |  |  |  |  |
| 5273.611806 | KERNEL\|xilinx:adm-pcie-7v3:1ddr:3.0-0\|cnn\_GOOD\|good\_cnn\|1:1:1\|good\_cnn\_5 | START | 0X1D009D8 | 1 |  |  |  |  |  |  |
| 5273.699352 | KERNEL\|xilinx:adm-pcie-7v3:1ddr:3.0-0\|cnn\_GOOD\|good\_cnn\|1:1:1\|good\_cnn\_6 | START | 0X1D009D8 | 1 |  |  |  |  |  |  |
| 5282.648964 | KERNEL\|xilinx:adm-pcie-7v3:1ddr:3.0-0\|cnn\_GOOD\|good\_cnn\|1:1:1\|good\_cnn\_1 | END | 0X1D009D8 | 1 |  |  |  |  |  |  |
| 5283.292441 | KERNEL\|xilinx:adm-pcie-7v3:1ddr:3.0-0\|cnn\_GOOD\|good\_cnn\|1:1:1\|good\_cnn\_2 | END | 0X1D009D8 | 1 |  |  |  |  |  |  |
| 5283.508575 | KERNEL\|xilinx:adm-pcie-7v3:1ddr:3.0-0\|cnn\_GOOD\|good\_cnn\|1:1:1\|good\_cnn\_4 | END | 0X1D009D8 | 1 |  |  |  |  |  |  |
| 5283.689663 | KERNEL\|xilinx:adm-pcie-7v3:1ddr:3.0-0\|cnn\_GOOD\|good\_cnn\|1:1:1\|good\_cnn\_3 | END | 0X1D009D8 | 1 |  |  |  |  |  |  |
| 5283.727276 | KERNEL\|xilinx:adm-pcie-7v3:1ddr:3.0-0\|cnn\_GOOD\|good\_cnn\|1:1:1\|good\_cnn\_5 | END | 0X1D009D8 | 1 |  |  |  |  |  |  |
| 5283.864662 | KERNEL\|xilinx:adm-pcie-7v3:1ddr:3.0-0\|cnn\_GOOD\|good\_cnn\|1:1:1\|good\_cnn\_6 | END | 0X1D009D8 | 1 |  |  |  |  |  |  |
| 5283.978204 | clFinish\|General | END |  |  |  |  |  |  |  |  |
| 5284.037627 | clGetEventProfilingInfo\|General | START |  |  |  |  |  |  |  |  |
| 5284.062974 | clGetEventProfilingInfo\|General | END |  |  |  |  |  |  |  |  |
| 5284.083736 | clGetEventProfilingInfo\|General | START |  |  |  |  |  |  |  |  |
| 5284.103603 | clGetEventProfilingInfo\|General | END |  |  |  |  |  |  |  |  |
| 5284.139576 | clEnqueueReadBuffer\|30303368 | START |  |  |  |  |  |  |  |  |
| 5284.177385 | READ\_BUFFER | QUEUE | 0X1D01D60 | 746496 |  |  |  |  |  |  |
| 5284.222272 | READ\_BUFFER | SUBMIT | 0X1D01D60 | 746496 |  |  |  |  |  |  |
| 5284.349371 | READ\_BUFFER | START | 0X1D01D60 | 746496 |  |  |  |  |  |  |
| 5285.222545 | READ\_BUFFER | END | 0X1D01D60 | 746496 |  |  |  |  |  |  |
| 5285.277539 | clEnqueueReadBuffer\|30303368 | END |  |  |  |  |  |  |  |  |
| 5285.372661 | clReleaseMemObject\|General | START |  |  |  |  |  |  |  |  |
| 5285.401344 | clReleaseMemObject\|General | END |  |  |  |  |  |  |  |  |
| 5285.42241 | clReleaseMemObject\|General | START |  |  |  |  |  |  |  |  |
| 5285.442763 | clReleaseMemObject\|General | END |  |  |  |  |  |  |  |  |
| 5285.462417 | clReleaseMemObject\|General | START |  |  |  |  |  |  |  |  |
| 5285.481877 | clReleaseMemObject\|General | END |  |  |  |  |  |  |  |  |
| 5285.518122 | clReleaseKernel\|General | START |  |  |  |  |  |  |  |  |
| 5285.539542 | clReleaseKernel\|General | END |  |  |  |  |  |  |  |  |
| 5285.575092 | clReleaseProgram\|General | START |  |  |  |  |  |  |  |  |
| 5285.596416 | clReleaseProgram\|General | END |  |  |  |  |  |  |  |  |
| 5285.624928 | clReleaseCommandQueue\|General | START |  |  |  |  |  |  |  |  |
| 5285.646315 | clReleaseCommandQueue\|General | END |  |  |  |  |  |  |  |  |
| 5285.668661 | clReleaseContext\|General | START |  |  |  |  |  |  |  |  |
| 5285.689628 | clReleaseContext\|General | END |  |  |  |  |  |  |  |  |
| 5273.055582 | Kernel\_Read\|xilinx:adm-pcie-7v3:1ddr:3.0-0\|cnn\_GOOD | Read | OCL Region | 1 | 1 | 87827 | 87828 | 0.00333333 | 5273.055582 | 5273.055586 |
| 5273.055589 | Kernel\_Read\|xilinx:adm-pcie-7v3:1ddr:3.0-0\|cnn\_GOOD | Read | OCL Region | 1 | 1 | 87829 | 87830 | 0.00333333 | 5273.055589 | 5273.055592 |
| 5273.055606 | Kernel\_Read\|xilinx:adm-pcie-7v3:1ddr:3.0-0\|cnn\_GOOD | Read | OCL Region | 1 | 1 | 87834 | 87835 | 0.00333333 | 5273.055606 | 5273.055609 |
| 5273.055616 | Kernel\_Read\|xilinx:adm-pcie-7v3:1ddr:3.0-0\|cnn\_GOOD | Read | OCL Region | 1 | 1 | 87837 | 87838 | 0.00333333 | 5273.055616 | 5273.055619 |
| 5273.055632 | Kernel\_Read\|xilinx:adm-pcie-7v3:1ddr:3.0-0\|cnn\_GOOD | Read | OCL Region | 1 | 1 | 87842 | 87843 | 0.00333333 | 5273.055632 | 5273.055636 |
| 5273.055656 | Kernel\_Read\|xilinx:adm-pcie-7v3:1ddr:3.0-0\|cnn\_GOOD | Read | OCL Region | 1 | 1 | 87849 | 87850 | 0.00333333 | 5273.055656 | 5273.055659 |
| 5273.055666 | Kernel\_Read\|xilinx:adm-pcie-7v3:1ddr:3.0-0\|cnn\_GOOD | Read | OCL Region | 1 | 1 | 87852 | 87853 | 0.00333333 | 5273.055666 | 5273.055669 |
| 5273.055682 | Kernel\_Read\|xilinx:adm-pcie-7v3:1ddr:3.0-0\|cnn\_GOOD | Read | OCL Region | 1 | 1 | 87857 | 87858 | 0.00333333 | 5273.055682 | 5273.055686 |
| 5273.055706 | Kernel\_Read\|xilinx:adm-pcie-7v3:1ddr:3.0-0\|cnn\_GOOD | Read | OCL Region | 1 | 1 | 87864 | 87865 | 0.00333333 | 5273.055706 | 5273.055709 |
| 5273.055716 | Kernel\_Read\|xilinx:adm-pcie-7v3:1ddr:3.0-0\|cnn\_GOOD | Read | OCL Region | 1 | 1 | 87867 | 87868 | 0.00333333 | 5273.055716 | 5273.055719 |
| 5273.055732 | Kernel\_Read\|xilinx:adm-pcie-7v3:1ddr:3.0-0\|cnn\_GOOD | Read | OCL Region | 1 | 1 | 87872 | 87873 | 0.00333333 | 5273.055732 | 5273.055736 |
| 5273.055756 | Kernel\_Read\|xilinx:adm-pcie-7v3:1ddr:3.0-0\|cnn\_GOOD | Read | OCL Region | 1 | 1 | 87879 | 87880 | 0.00333333 | 5273.055756 | 5273.055759 |
| 5273.055766 | Kernel\_Read\|xilinx:adm-pcie-7v3:1ddr:3.0-0\|cnn\_GOOD | Read | OCL Region | 1 | 1 | 87882 | 87883 | 0.00333333 | 5273.055766 | 5273.055769 |
| 5273.055782 | Kernel\_Read\|xilinx:adm-pcie-7v3:1ddr:3.0-0\|cnn\_GOOD | Read | OCL Region | 1 | 1 | 87887 | 87888 | 0.00333333 | 5273.055782 | 5273.055786 |
| 5273.055806 | Kernel\_Read\|xilinx:adm-pcie-7v3:1ddr:3.0-0\|cnn\_GOOD | Read | OCL Region | 1 | 1 | 87894 | 87895 | 0.00333333 | 5273.055806 | 5273.055809 |
| 5273.055816 | Kernel\_Read\|xilinx:adm-pcie-7v3:1ddr:3.0-0\|cnn\_GOOD | Read | OCL Region | 1 | 1 | 87897 | 87898 | 0.00333333 | 5273.055816 | 5273.055819 |
| 5273.056182 | Kernel\_Read\|xilinx:adm-pcie-7v3:1ddr:3.0-0\|cnn\_GOOD | Read | OCL Region | 1 | 1 | 88007 | 88008 | 0.00333333 | 5273.056182 | 5273.056186 |
| 5273.056249 | Kernel\_Read\|xilinx:adm-pcie-7v3:1ddr:3.0-0\|cnn\_GOOD | Read | OCL Region | 1 | 1 | 88027 | 88028 | 0.00333333 | 5273.056249 | 5273.056252 |
| 5273.056316 | Kernel\_Read\|xilinx:adm-pcie-7v3:1ddr:3.0-0\|cnn\_GOOD | Read | OCL Region | 1 | 1 | 88047 | 88048 | 0.00333333 | 5273.056316 | 5273.056319 |
| 5273.056382 | Kernel\_Read\|xilinx:adm-pcie-7v3:1ddr:3.0-0\|cnn\_GOOD | Read | OCL Region | 1 | 1 | 88067 | 88068 | 0.00333333 | 5273.056382 | 5273.056386 |
| 5273.056449 | Kernel\_Read\|xilinx:adm-pcie-7v3:1ddr:3.0-0\|cnn\_GOOD | Read | OCL Region | 1 | 1 | 88087 | 88088 | 0.00333333 | 5273.056449 | 5273.056452 |
| 5273.056516 | Kernel\_Read\|xilinx:adm-pcie-7v3:1ddr:3.0-0\|cnn\_GOOD | Read | OCL Region | 1 | 1 | 88107 | 88108 | 0.00333333 | 5273.056516 | 5273.056519 |
| 5273.056582 | Kernel\_Read\|xilinx:adm-pcie-7v3:1ddr:3.0-0\|cnn\_GOOD | Read | OCL Region | 1 | 1 | 88127 | 88128 | 0.00333333 | 5273.056582 | 5273.056586 |
| 5273.056649 | Kernel\_Read\|xilinx:adm-pcie-7v3:1ddr:3.0-0\|cnn\_GOOD | Read | OCL Region | 1 | 1 | 88147 | 88148 | 0.00333333 | 5273.056649 | 5273.056652 |
| 5273.056716 | Kernel\_Read\|xilinx:adm-pcie-7v3:1ddr:3.0-0\|cnn\_GOOD | Read | OCL Region | 1 | 1 | 88167 | 88168 | 0.00333333 | 5273.056716 | 5273.056719 |
| 5273.056782 | Kernel\_Read\|xilinx:adm-pcie-7v3:1ddr:3.0-0\|cnn\_GOOD | Read | OCL Region | 1 | 1 | 88187 | 88188 | 0.00333333 | 5273.056782 | 5273.056786 |
| 5273.056849 | Kernel\_Read\|xilinx:adm-pcie-7v3:1ddr:3.0-0\|cnn\_GOOD | Read | OCL Region | 1 | 1 | 88207 | 88208 | 0.00333333 | 5273.056849 | 5273.056852 |
| 5273.056916 | Kernel\_Read\|xilinx:adm-pcie-7v3:1ddr:3.0-0\|cnn\_GOOD | Read | OCL Region | 1 | 1 | 88227 | 88228 | 0.00333333 | 5273.056916 | 5273.056919 |
| 5273.056982 | Kernel\_Read\|xilinx:adm-pcie-7v3:1ddr:3.0-0\|cnn\_GOOD | Read | OCL Region | 1 | 1 | 88247 | 88248 | 0.00333333 | 5273.056982 | 5273.056986 |
| 5273.057049 | Kernel\_Read\|xilinx:adm-pcie-7v3:1ddr:3.0-0\|cnn\_GOOD | Read | OCL Region | 1 | 1 | 88267 | 88268 | 0.00333333 | 5273.057049 | 5273.057052 |
| 5273.057116 | Kernel\_Read\|xilinx:adm-pcie-7v3:1ddr:3.0-0\|cnn\_GOOD | Read | OCL Region | 1 | 1 | 88287 | 88288 | 0.00333333 | 5273.057116 | 5273.057119 |
| 5273.057182 | Kernel\_Read\|xilinx:adm-pcie-7v3:1ddr:3.0-0\|cnn\_GOOD | Read | OCL Region | 1 | 1 | 88307 | 88308 | 0.00333333 | 5273.057182 | 5273.057186 |
| 5273.057249 | Kernel\_Read\|xilinx:adm-pcie-7v3:1ddr:3.0-0\|cnn\_GOOD | Read | OCL Region | 1 | 1 | 88327 | 88328 | 0.00333333 | 5273.057249 | 5273.057252 |
| 5273.057316 | Kernel\_Read\|xilinx:adm-pcie-7v3:1ddr:3.0-0\|cnn\_GOOD | Read | OCL Region | 1 | 1 | 88347 | 88348 | 0.00333333 | 5273.057316 | 5273.057319 |
| 5273.057382 | Kernel\_Read\|xilinx:adm-pcie-7v3:1ddr:3.0-0\|cnn\_GOOD | Read | OCL Region | 1 | 1 | 88367 | 88368 | 0.00333333 | 5273.057382 | 5273.057386 |
| 5273.057449 | Kernel\_Read\|xilinx:adm-pcie-7v3:1ddr:3.0-0\|cnn\_GOOD | Read | OCL Region | 1 | 1 | 88387 | 88388 | 0.00333333 | 5273.057449 | 5273.057452 |
| 5273.057516 | Kernel\_Read\|xilinx:adm-pcie-7v3:1ddr:3.0-0\|cnn\_GOOD | Read | OCL Region | 1 | 1 | 88407 | 88408 | 0.00333333 | 5273.057516 | 5273.057519 |
| 5273.057582 | Kernel\_Read\|xilinx:adm-pcie-7v3:1ddr:3.0-0\|cnn\_GOOD | Read | OCL Region | 1 | 1 | 88427 | 88428 | 0.00333333 | 5273.057582 | 5273.057586 |
| 5273.057649 | Kernel\_Read\|xilinx:adm-pcie-7v3:1ddr:3.0-0\|cnn\_GOOD | Read | OCL Region | 1 | 1 | 88447 | 88448 | 0.00333333 | 5273.057649 | 5273.057652 |
| 5273.057716 | Kernel\_Read\|xilinx:adm-pcie-7v3:1ddr:3.0-0\|cnn\_GOOD | Read | OCL Region | 1 | 1 | 88467 | 88468 | 0.00333333 | 5273.057716 | 5273.057719 |
| 5273.057782 | Kernel\_Read\|xilinx:adm-pcie-7v3:1ddr:3.0-0\|cnn\_GOOD | Read | OCL Region | 1 | 1 | 88487 | 88488 | 0.00333333 | 5273.057782 | 5273.057786 |
| 5273.057849 | Kernel\_Read\|xilinx:adm-pcie-7v3:1ddr:3.0-0\|cnn\_GOOD | Read | OCL Region | 1 | 1 | 88507 | 88508 | 0.00333333 | 5273.057849 | 5273.057852 |
| 5273.057916 | Kernel\_Read\|xilinx:adm-pcie-7v3:1ddr:3.0-0\|cnn\_GOOD | Read | OCL Region | 1 | 1 | 88527 | 88528 | 0.00333333 | 5273.057916 | 5273.057919 |
| 5273.057982 | Kernel\_Read\|xilinx:adm-pcie-7v3:1ddr:3.0-0\|cnn\_GOOD | Read | OCL Region | 1 | 1 | 88547 | 88548 | 0.00333333 | 5273.057982 | 5273.057986 |
| 5273.058049 | Kernel\_Read\|xilinx:adm-pcie-7v3:1ddr:3.0-0\|cnn\_GOOD | Read | OCL Region | 1 | 1 | 88567 | 88568 | 0.00333333 | 5273.058049 | 5273.058052 |
| 5273.058129 | Kernel\_Read\|xilinx:adm-pcie-7v3:1ddr:3.0-0\|cnn\_GOOD | Read | OCL Region | 1 | 1 | 88591 | 88592 | 0.00333333 | 5273.058129 | 5273.058132 |
| 5273.058182 | Kernel\_Read\|xilinx:adm-pcie-7v3:1ddr:3.0-0\|cnn\_GOOD | Read | OCL Region | 1 | 1 | 88607 | 88608 | 0.00333333 | 5273.058182 | 5273.058186 |
| 5273.058249 | Kernel\_Read\|xilinx:adm-pcie-7v3:1ddr:3.0-0\|cnn\_GOOD | Read | OCL Region | 1 | 1 | 88627 | 88628 | 0.00333333 | 5273.058249 | 5273.058252 |
| 5273.058316 | Kernel\_Read\|xilinx:adm-pcie-7v3:1ddr:3.0-0\|cnn\_GOOD | Read | OCL Region | 1 | 1 | 88647 | 88648 | 0.00333333 | 5273.058316 | 5273.058319 |
| 5273.058382 | Kernel\_Read\|xilinx:adm-pcie-7v3:1ddr:3.0-0\|cnn\_GOOD | Read | OCL Region | 1 | 1 | 88667 | 88668 | 0.00333333 | 5273.058382 | 5273.058386 |
| 5273.058449 | Kernel\_Read\|xilinx:adm-pcie-7v3:1ddr:3.0-0\|cnn\_GOOD | Read | OCL Region | 1 | 1 | 88687 | 88688 | 0.00333333 | 5273.058449 | 5273.058452 |
| 5273.058516 | Kernel\_Read\|xilinx:adm-pcie-7v3:1ddr:3.0-0\|cnn\_GOOD | Read | OCL Region | 1 | 1 | 88707 | 88708 | 0.00333333 | 5273.058516 | 5273.058519 |
| 5273.058582 | Kernel\_Read\|xilinx:adm-pcie-7v3:1ddr:3.0-0\|cnn\_GOOD | Read | OCL Region | 1 | 1 | 88727 | 88728 | 0.00333333 | 5273.058582 | 5273.058586 |
| 5273.058649 | Kernel\_Read\|xilinx:adm-pcie-7v3:1ddr:3.0-0\|cnn\_GOOD | Read | OCL Region | 1 | 1 | 88747 | 88748 | 0.00333333 | 5273.058649 | 5273.058652 |
| 5273.058716 | Kernel\_Read\|xilinx:adm-pcie-7v3:1ddr:3.0-0\|cnn\_GOOD | Read | OCL Region | 1 | 1 | 88767 | 88768 | 0.00333333 | 5273.058716 | 5273.058719 |
| 5273.058782 | Kernel\_Read\|xilinx:adm-pcie-7v3:1ddr:3.0-0\|cnn\_GOOD | Read | OCL Region | 1 | 1 | 88787 | 88788 | 0.00333333 | 5273.058782 | 5273.058786 |
| 5273.058849 | Kernel\_Read\|xilinx:adm-pcie-7v3:1ddr:3.0-0\|cnn\_GOOD | Read | OCL Region | 1 | 1 | 88807 | 88808 | 0.00333333 | 5273.058849 | 5273.058852 |
| 5273.058916 | Kernel\_Read\|xilinx:adm-pcie-7v3:1ddr:3.0-0\|cnn\_GOOD | Read | OCL Region | 1 | 1 | 88827 | 88828 | 0.00333333 | 5273.058916 | 5273.058919 |
| 5273.058982 | Kernel\_Read\|xilinx:adm-pcie-7v3:1ddr:3.0-0\|cnn\_GOOD | Read | OCL Region | 1 | 1 | 88847 | 88848 | 0.00333333 | 5273.058982 | 5273.058986 |
| 5273.059049 | Kernel\_Read\|xilinx:adm-pcie-7v3:1ddr:3.0-0\|cnn\_GOOD | Read | OCL Region | 1 | 1 | 88867 | 88868 | 0.00333333 | 5273.059049 | 5273.059052 |
| 5273.059116 | Kernel\_Read\|xilinx:adm-pcie-7v3:1ddr:3.0-0\|cnn\_GOOD | Read | OCL Region | 1 | 1 | 88887 | 88888 | 0.00333333 | 5273.059116 | 5273.059119 |
| 5273.059182 | Kernel\_Read\|xilinx:adm-pcie-7v3:1ddr:3.0-0\|cnn\_GOOD | Read | OCL Region | 1 | 1 | 88907 | 88908 | 0.00333333 | 5273.059182 | 5273.059186 |
| 5273.059249 | Kernel\_Read\|xilinx:adm-pcie-7v3:1ddr:3.0-0\|cnn\_GOOD | Read | OCL Region | 1 | 1 | 88927 | 88928 | 0.00333333 | 5273.059249 | 5273.059252 |
| 5273.059316 | Kernel\_Read\|xilinx:adm-pcie-7v3:1ddr:3.0-0\|cnn\_GOOD | Read | OCL Region | 1 | 1 | 88947 | 88948 | 0.00333333 | 5273.059316 | 5273.059319 |
| 5273.059382 | Kernel\_Read\|xilinx:adm-pcie-7v3:1ddr:3.0-0\|cnn\_GOOD | Read | OCL Region | 1 | 1 | 88967 | 88968 | 0.00333333 | 5273.059382 | 5273.059386 |
| 5273.059449 | Kernel\_Read\|xilinx:adm-pcie-7v3:1ddr:3.0-0\|cnn\_GOOD | Read | OCL Region | 1 | 1 | 88987 | 88988 | 0.00333333 | 5273.059449 | 5273.059452 |
| 5273.059516 | Kernel\_Read\|xilinx:adm-pcie-7v3:1ddr:3.0-0\|cnn\_GOOD | Read | OCL Region | 1 | 1 | 89007 | 89008 | 0.00333333 | 5273.059516 | 5273.059519 |
| 5273.059582 | Kernel\_Read\|xilinx:adm-pcie-7v3:1ddr:3.0-0\|cnn\_GOOD | Read | OCL Region | 1 | 1 | 89027 | 89028 | 0.00333333 | 5273.059582 | 5273.059586 |
| 5273.059649 | Kernel\_Read\|xilinx:adm-pcie-7v3:1ddr:3.0-0\|cnn\_GOOD | Read | OCL Region | 1 | 1 | 89047 | 89048 | 0.00333333 | 5273.059649 | 5273.059652 |
| 5273.059716 | Kernel\_Read\|xilinx:adm-pcie-7v3:1ddr:3.0-0\|cnn\_GOOD | Read | OCL Region | 1 | 1 | 89067 | 89068 | 0.00333333 | 5273.059716 | 5273.059719 |
| 5273.059782 | Kernel\_Read\|xilinx:adm-pcie-7v3:1ddr:3.0-0\|cnn\_GOOD | Read | OCL Region | 1 | 1 | 89087 | 89088 | 0.00333333 | 5273.059782 | 5273.059786 |
| 5273.059849 | Kernel\_Read\|xilinx:adm-pcie-7v3:1ddr:3.0-0\|cnn\_GOOD | Read | OCL Region | 1 | 1 | 89107 | 89108 | 0.00333333 | 5273.059849 | 5273.059852 |
| 5273.059916 | Kernel\_Read\|xilinx:adm-pcie-7v3:1ddr:3.0-0\|cnn\_GOOD | Read | OCL Region | 1 | 1 | 89127 | 89128 | 0.00333333 | 5273.059916 | 5273.059919 |
| 5273.059982 | Kernel\_Read\|xilinx:adm-pcie-7v3:1ddr:3.0-0\|cnn\_GOOD | Read | OCL Region | 1 | 1 | 89147 | 89148 | 0.00333333 | 5273.059982 | 5273.059986 |
| 5273.060049 | Kernel\_Read\|xilinx:adm-pcie-7v3:1ddr:3.0-0\|cnn\_GOOD | Read | OCL Region | 1 | 1 | 89167 | 89168 | 0.00333333 | 5273.060049 | 5273.060052 |
| 5273.060116 | Kernel\_Read\|xilinx:adm-pcie-7v3:1ddr:3.0-0\|cnn\_GOOD | Read | OCL Region | 1 | 1 | 89187 | 89188 | 0.00333333 | 5273.060116 | 5273.060119 |
| 5273.060182 | Kernel\_Read\|xilinx:adm-pcie-7v3:1ddr:3.0-0\|cnn\_GOOD | Read | OCL Region | 1 | 1 | 89207 | 89208 | 0.00333333 | 5273.060182 | 5273.060186 |
| 5273.060249 | Kernel\_Read\|xilinx:adm-pcie-7v3:1ddr:3.0-0\|cnn\_GOOD | Read | OCL Region | 1 | 1 | 89227 | 89228 | 0.00333333 | 5273.060249 | 5273.060252 |
| 5273.060316 | Kernel\_Read\|xilinx:adm-pcie-7v3:1ddr:3.0-0\|cnn\_GOOD | Read | OCL Region | 1 | 1 | 89247 | 89248 | 0.00333333 | 5273.060316 | 5273.060319 |
| 5273.060382 | Kernel\_Read\|xilinx:adm-pcie-7v3:1ddr:3.0-0\|cnn\_GOOD | Read | OCL Region | 1 | 1 | 89267 | 89268 | 0.00333333 | 5273.060382 | 5273.060386 |
| 5273.060449 | Kernel\_Read\|xilinx:adm-pcie-7v3:1ddr:3.0-0\|cnn\_GOOD | Read | OCL Region | 1 | 1 | 89287 | 89288 | 0.00333333 | 5273.060449 | 5273.060452 |
| 5273.060516 | Kernel\_Read\|xilinx:adm-pcie-7v3:1ddr:3.0-0\|cnn\_GOOD | Read | OCL Region | 1 | 1 | 89307 | 89308 | 0.00333333 | 5273.060516 | 5273.060519 |
| 5273.060582 | Kernel\_Read\|xilinx:adm-pcie-7v3:1ddr:3.0-0\|cnn\_GOOD | Read | OCL Region | 1 | 1 | 89327 | 89328 | 0.00333333 | 5273.060582 | 5273.060586 |
| 5273.060649 | Kernel\_Read\|xilinx:adm-pcie-7v3:1ddr:3.0-0\|cnn\_GOOD | Read | OCL Region | 1 | 1 | 89347 | 89348 | 0.00333333 | 5273.060649 | 5273.060652 |
| 5273.060716 | Kernel\_Read\|xilinx:adm-pcie-7v3:1ddr:3.0-0\|cnn\_GOOD | Read | OCL Region | 1 | 1 | 89367 | 89368 | 0.00333333 | 5273.060716 | 5273.060719 |
| 5273.060782 | Kernel\_Read\|xilinx:adm-pcie-7v3:1ddr:3.0-0\|cnn\_GOOD | Read | OCL Region | 1 | 1 | 89387 | 89388 | 0.00333333 | 5273.060782 | 5273.060786 |
| 5273.060849 | Kernel\_Read\|xilinx:adm-pcie-7v3:1ddr:3.0-0\|cnn\_GOOD | Read | OCL Region | 1 | 1 | 89407 | 89408 | 0.00333333 | 5273.060849 | 5273.060852 |
| 5273.060916 | Kernel\_Read\|xilinx:adm-pcie-7v3:1ddr:3.0-0\|cnn\_GOOD | Read | OCL Region | 1 | 1 | 89427 | 89428 | 0.00333333 | 5273.060916 | 5273.060919 |
| 5273.060982 | Kernel\_Read\|xilinx:adm-pcie-7v3:1ddr:3.0-0\|cnn\_GOOD | Read | OCL Region | 1 | 1 | 89447 | 89448 | 0.00333333 | 5273.060982 | 5273.060986 |
| 5273.061049 | Kernel\_Read\|xilinx:adm-pcie-7v3:1ddr:3.0-0\|cnn\_GOOD | Read | OCL Region | 1 | 1 | 89467 | 89468 | 0.00333333 | 5273.061049 | 5273.061052 |
| 5273.061116 | Kernel\_Read\|xilinx:adm-pcie-7v3:1ddr:3.0-0\|cnn\_GOOD | Read | OCL Region | 1 | 1 | 89487 | 89488 | 0.00333333 | 5273.061116 | 5273.061119 |
| 5273.061182 | Kernel\_Read\|xilinx:adm-pcie-7v3:1ddr:3.0-0\|cnn\_GOOD | Read | OCL Region | 1 | 1 | 89507 | 89508 | 0.00333333 | 5273.061182 | 5273.061186 |
| 5273.061249 | Kernel\_Read\|xilinx:adm-pcie-7v3:1ddr:3.0-0\|cnn\_GOOD | Read | OCL Region | 1 | 1 | 89527 | 89528 | 0.00333333 | 5273.061249 | 5273.061252 |
| 5273.061316 | Kernel\_Read\|xilinx:adm-pcie-7v3:1ddr:3.0-0\|cnn\_GOOD | Read | OCL Region | 1 | 1 | 89547 | 89548 | 0.00333333 | 5273.061316 | 5273.061319 |
| 5273.061392 | Kernel\_Read\|xilinx:adm-pcie-7v3:1ddr:3.0-0\|cnn\_GOOD | Read | OCL Region | 1 | 1 | 89570 | 89571 | 0.00333333 | 5273.061392 | 5273.061396 |
| 5273.061449 | Kernel\_Read\|xilinx:adm-pcie-7v3:1ddr:3.0-0\|cnn\_GOOD | Read | OCL Region | 1 | 1 | 89587 | 89588 | 0.00333333 | 5273.061449 | 5273.061452 |
| 5273.061516 | Kernel\_Read\|xilinx:adm-pcie-7v3:1ddr:3.0-0\|cnn\_GOOD | Read | OCL Region | 1 | 1 | 89607 | 89608 | 0.00333333 | 5273.061516 | 5273.061519 |
| 5273.061792 | Kernel\_Read\|xilinx:adm-pcie-7v3:1ddr:3.0-0\|cnn\_GOOD | Read | OCL Region | 1 | 1 | 89690 | 89691 | 0.00333333 | 5273.061792 | 5273.061796 |
| 5273.061799 | Kernel\_Read\|xilinx:adm-pcie-7v3:1ddr:3.0-0\|cnn\_GOOD | Read | OCL Region | 1 | 1 | 89692 | 89693 | 0.00333333 | 5273.061799 | 5273.061802 |
| 5273.061806 | Kernel\_Read\|xilinx:adm-pcie-7v3:1ddr:3.0-0\|cnn\_GOOD | Read | OCL Region | 1 | 1 | 89694 | 89695 | 0.00333333 | 5273.061806 | 5273.061809 |
| 5273.061809 | Kernel\_Read\|xilinx:adm-pcie-7v3:1ddr:3.0-0\|cnn\_GOOD | Read | OCL Region | 1 | 1 | 89695 | 89696 | 0.00333333 | 5273.061809 | 5273.061812 |
| 5273.061866 | Kernel\_Read\|xilinx:adm-pcie-7v3:1ddr:3.0-0\|cnn\_GOOD | Read | OCL Region | 1 | 1 | 89712 | 89713 | 0.00333333 | 5273.061866 | 5273.061869 |
| 5273.061926 | Kernel\_Read\|xilinx:adm-pcie-7v3:1ddr:3.0-0\|cnn\_GOOD | Read | OCL Region | 1 | 1 | 89730 | 89731 | 0.00333333 | 5273.061926 | 5273.061929 |
| 5273.061982 | Kernel\_Read\|xilinx:adm-pcie-7v3:1ddr:3.0-0\|cnn\_GOOD | Read | OCL Region | 1 | 1 | 89747 | 89748 | 0.00333333 | 5273.061982 | 5273.061986 |
| 5273.062049 | Kernel\_Read\|xilinx:adm-pcie-7v3:1ddr:3.0-0\|cnn\_GOOD | Read | OCL Region | 1 | 1 | 89767 | 89768 | 0.00333333 | 5273.062049 | 5273.062052 |
| 5273.062116 | Kernel\_Read\|xilinx:adm-pcie-7v3:1ddr:3.0-0\|cnn\_GOOD | Read | OCL Region | 1 | 1 | 89787 | 89788 | 0.00333333 | 5273.062116 | 5273.062119 |
| 5273.062182 | Kernel\_Read\|xilinx:adm-pcie-7v3:1ddr:3.0-0\|cnn\_GOOD | Read | OCL Region | 1 | 1 | 89807 | 89808 | 0.00333333 | 5273.062182 | 5273.062186 |
| 5273.062249 | Kernel\_Read\|xilinx:adm-pcie-7v3:1ddr:3.0-0\|cnn\_GOOD | Read | OCL Region | 1 | 1 | 89827 | 89828 | 0.00333333 | 5273.062249 | 5273.062252 |
| 5273.062316 | Kernel\_Read\|xilinx:adm-pcie-7v3:1ddr:3.0-0\|cnn\_GOOD | Read | OCL Region | 1 | 1 | 89847 | 89848 | 0.00333333 | 5273.062316 | 5273.062319 |
| 5273.062382 | Kernel\_Read\|xilinx:adm-pcie-7v3:1ddr:3.0-0\|cnn\_GOOD | Read | OCL Region | 1 | 1 | 89867 | 89868 | 0.00333333 | 5273.062382 | 5273.062386 |
| 5273.062449 | Kernel\_Read\|xilinx:adm-pcie-7v3:1ddr:3.0-0\|cnn\_GOOD | Read | OCL Region | 1 | 1 | 89887 | 89888 | 0.00333333 | 5273.062449 | 5273.062452 |
| 5273.062516 | Kernel\_Read\|xilinx:adm-pcie-7v3:1ddr:3.0-0\|cnn\_GOOD | Read | OCL Region | 1 | 1 | 89907 | 89908 | 0.00333333 | 5273.062516 | 5273.062519 |
| 5273.062582 | Kernel\_Read\|xilinx:adm-pcie-7v3:1ddr:3.0-0\|cnn\_GOOD | Read | OCL Region | 1 | 1 | 89927 | 89928 | 0.00333333 | 5273.062582 | 5273.062586 |
| 5273.062649 | Kernel\_Read\|xilinx:adm-pcie-7v3:1ddr:3.0-0\|cnn\_GOOD | Read | OCL Region | 1 | 1 | 89947 | 89948 | 0.00333333 | 5273.062649 | 5273.062652 |
| 5273.062716 | Kernel\_Read\|xilinx:adm-pcie-7v3:1ddr:3.0-0\|cnn\_GOOD | Read | OCL Region | 1 | 1 | 89967 | 89968 | 0.00333333 | 5273.062716 | 5273.062719 |
| 5273.062782 | Kernel\_Read\|xilinx:adm-pcie-7v3:1ddr:3.0-0\|cnn\_GOOD | Read | OCL Region | 1 | 1 | 89987 | 89988 | 0.00333333 | 5273.062782 | 5273.062786 |
| 5273.062849 | Kernel\_Read\|xilinx:adm-pcie-7v3:1ddr:3.0-0\|cnn\_GOOD | Read | OCL Region | 1 | 1 | 90007 | 90008 | 0.00333333 | 5273.062849 | 5273.062852 |
| 5273.062916 | Kernel\_Read\|xilinx:adm-pcie-7v3:1ddr:3.0-0\|cnn\_GOOD | Read | OCL Region | 1 | 1 | 90027 | 90028 | 0.00333333 | 5273.062916 | 5273.062919 |
| 5273.062982 | Kernel\_Read\|xilinx:adm-pcie-7v3:1ddr:3.0-0\|cnn\_GOOD | Read | OCL Region | 1 | 1 | 90047 | 90048 | 0.00333333 | 5273.062982 | 5273.062986 |
| 5273.063049 | Kernel\_Read\|xilinx:adm-pcie-7v3:1ddr:3.0-0\|cnn\_GOOD | Read | OCL Region | 1 | 1 | 90067 | 90068 | 0.00333333 | 5273.063049 | 5273.063052 |
| 5273.063116 | Kernel\_Read\|xilinx:adm-pcie-7v3:1ddr:3.0-0\|cnn\_GOOD | Read | OCL Region | 1 | 1 | 90087 | 90088 | 0.00333333 | 5273.063116 | 5273.063119 |
| 5273.063182 | Kernel\_Read\|xilinx:adm-pcie-7v3:1ddr:3.0-0\|cnn\_GOOD | Read | OCL Region | 1 | 1 | 90107 | 90108 | 0.00333333 | 5273.063182 | 5273.063186 |
| 5273.063249 | Kernel\_Read\|xilinx:adm-pcie-7v3:1ddr:3.0-0\|cnn\_GOOD | Read | OCL Region | 1 | 1 | 90127 | 90128 | 0.00333333 | 5273.063249 | 5273.063252 |
| 5273.063316 | Kernel\_Read\|xilinx:adm-pcie-7v3:1ddr:3.0-0\|cnn\_GOOD | Read | OCL Region | 1 | 1 | 90147 | 90148 | 0.00333333 | 5273.063316 | 5273.063319 |
| 5273.063382 | Kernel\_Read\|xilinx:adm-pcie-7v3:1ddr:3.0-0\|cnn\_GOOD | Read | OCL Region | 1 | 1 | 90167 | 90168 | 0.00333333 | 5273.063382 | 5273.063386 |
| 5273.063449 | Kernel\_Read\|xilinx:adm-pcie-7v3:1ddr:3.0-0\|cnn\_GOOD | Read | OCL Region | 1 | 1 | 90187 | 90188 | 0.00333333 | 5273.063449 | 5273.063452 |
| 5273.063516 | Kernel\_Read\|xilinx:adm-pcie-7v3:1ddr:3.0-0\|cnn\_GOOD | Read | OCL Region | 1 | 1 | 90207 | 90208 | 0.00333333 | 5273.063516 | 5273.063519 |
| 5273.063582 | Kernel\_Read\|xilinx:adm-pcie-7v3:1ddr:3.0-0\|cnn\_GOOD | Read | OCL Region | 1 | 1 | 90227 | 90228 | 0.00333333 | 5273.063582 | 5273.063586 |
| 5273.063649 | Kernel\_Read\|xilinx:adm-pcie-7v3:1ddr:3.0-0\|cnn\_GOOD | Read | OCL Region | 1 | 1 | 90247 | 90248 | 0.00333333 | 5273.063649 | 5273.063652 |
| 5273.063716 | Kernel\_Read\|xilinx:adm-pcie-7v3:1ddr:3.0-0\|cnn\_GOOD | Read | OCL Region | 1 | 1 | 90267 | 90268 | 0.00333333 | 5273.063716 | 5273.063719 |
| 5273.063782 | Kernel\_Read\|xilinx:adm-pcie-7v3:1ddr:3.0-0\|cnn\_GOOD | Read | OCL Region | 1 | 1 | 90287 | 90288 | 0.00333333 | 5273.063782 | 5273.063786 |
| 5273.063849 | Kernel\_Read\|xilinx:adm-pcie-7v3:1ddr:3.0-0\|cnn\_GOOD | Read | OCL Region | 1 | 1 | 90307 | 90308 | 0.00333333 | 5273.063849 | 5273.063852 |
| 5273.063916 | Kernel\_Read\|xilinx:adm-pcie-7v3:1ddr:3.0-0\|cnn\_GOOD | Read | OCL Region | 1 | 1 | 90327 | 90328 | 0.00333333 | 5273.063916 | 5273.063919 |
| 5273.063982 | Kernel\_Read\|xilinx:adm-pcie-7v3:1ddr:3.0-0\|cnn\_GOOD | Read | OCL Region | 1 | 1 | 90347 | 90348 | 0.00333333 | 5273.063982 | 5273.063986 |
| 5273.064049 | Kernel\_Read\|xilinx:adm-pcie-7v3:1ddr:3.0-0\|cnn\_GOOD | Read | OCL Region | 1 | 1 | 90367 | 90368 | 0.00333333 | 5273.064049 | 5273.064052 |
| 5273.064116 | Kernel\_Read\|xilinx:adm-pcie-7v3:1ddr:3.0-0\|cnn\_GOOD | Read | OCL Region | 1 | 1 | 90387 | 90388 | 0.00333333 | 5273.064116 | 5273.064119 |
| 5273.064182 | Kernel\_Read\|xilinx:adm-pcie-7v3:1ddr:3.0-0\|cnn\_GOOD | Read | OCL Region | 1 | 1 | 90407 | 90408 | 0.00333333 | 5273.064182 | 5273.064186 |
| 5273.064249 | Kernel\_Read\|xilinx:adm-pcie-7v3:1ddr:3.0-0\|cnn\_GOOD | Read | OCL Region | 1 | 1 | 90427 | 90428 | 0.00333333 | 5273.064249 | 5273.064252 |
| 5273.064316 | Kernel\_Read\|xilinx:adm-pcie-7v3:1ddr:3.0-0\|cnn\_GOOD | Read | OCL Region | 1 | 1 | 90447 | 90448 | 0.00333333 | 5273.064316 | 5273.064319 |
| 5273.064382 | Kernel\_Read\|xilinx:adm-pcie-7v3:1ddr:3.0-0\|cnn\_GOOD | Read | OCL Region | 1 | 1 | 90467 | 90468 | 0.00333333 | 5273.064382 | 5273.064386 |
| 5273.064449 | Kernel\_Read\|xilinx:adm-pcie-7v3:1ddr:3.0-0\|cnn\_GOOD | Read | OCL Region | 1 | 1 | 90487 | 90488 | 0.00333333 | 5273.064449 | 5273.064452 |
| 5273.064516 | Kernel\_Read\|xilinx:adm-pcie-7v3:1ddr:3.0-0\|cnn\_GOOD | Read | OCL Region | 1 | 1 | 90507 | 90508 | 0.00333333 | 5273.064516 | 5273.064519 |
| 5273.064582 | Kernel\_Read\|xilinx:adm-pcie-7v3:1ddr:3.0-0\|cnn\_GOOD | Read | OCL Region | 1 | 1 | 90527 | 90528 | 0.00333333 | 5273.064582 | 5273.064586 |
| 5273.064649 | Kernel\_Read\|xilinx:adm-pcie-7v3:1ddr:3.0-0\|cnn\_GOOD | Read | OCL Region | 1 | 1 | 90547 | 90548 | 0.00333333 | 5273.064649 | 5273.064652 |
| 5273.064716 | Kernel\_Read\|xilinx:adm-pcie-7v3:1ddr:3.0-0\|cnn\_GOOD | Read | OCL Region | 1 | 1 | 90567 | 90568 | 0.00333333 | 5273.064716 | 5273.064719 |
| 5273.064782 | Kernel\_Read\|xilinx:adm-pcie-7v3:1ddr:3.0-0\|cnn\_GOOD | Read | OCL Region | 1 | 1 | 90587 | 90588 | 0.00333333 | 5273.064782 | 5273.064786 |
| 5273.064849 | Kernel\_Read\|xilinx:adm-pcie-7v3:1ddr:3.0-0\|cnn\_GOOD | Read | OCL Region | 1 | 1 | 90607 | 90608 | 0.00333333 | 5273.064849 | 5273.064852 |
| 5273.064916 | Kernel\_Read\|xilinx:adm-pcie-7v3:1ddr:3.0-0\|cnn\_GOOD | Read | OCL Region | 1 | 1 | 90627 | 90628 | 0.00333333 | 5273.064916 | 5273.064919 |
| 5273.064982 | Kernel\_Read\|xilinx:adm-pcie-7v3:1ddr:3.0-0\|cnn\_GOOD | Read | OCL Region | 1 | 1 | 90647 | 90648 | 0.00333333 | 5273.064982 | 5273.064986 |
| 5273.065059 | Kernel\_Read\|xilinx:adm-pcie-7v3:1ddr:3.0-0\|cnn\_GOOD | Read | OCL Region | 1 | 1 | 90670 | 90671 | 0.00333333 | 5273.065059 | 5273.065062 |
| 5273.065116 | Kernel\_Read\|xilinx:adm-pcie-7v3:1ddr:3.0-0\|cnn\_GOOD | Read | OCL Region | 1 | 1 | 90687 | 90688 | 0.00333333 | 5273.065116 | 5273.065119 |
| 5273.065182 | Kernel\_Read\|xilinx:adm-pcie-7v3:1ddr:3.0-0\|cnn\_GOOD | Read | OCL Region | 1 | 1 | 90707 | 90708 | 0.00333333 | 5273.065182 | 5273.065186 |
| 5273.065249 | Kernel\_Read\|xilinx:adm-pcie-7v3:1ddr:3.0-0\|cnn\_GOOD | Read | OCL Region | 1 | 1 | 90727 | 90728 | 0.00333333 | 5273.065249 | 5273.065252 |
| 5273.065316 | Kernel\_Read\|xilinx:adm-pcie-7v3:1ddr:3.0-0\|cnn\_GOOD | Read | OCL Region | 1 | 1 | 90747 | 90748 | 0.00333333 | 5273.065316 | 5273.065319 |
| 5273.065382 | Kernel\_Read\|xilinx:adm-pcie-7v3:1ddr:3.0-0\|cnn\_GOOD | Read | OCL Region | 1 | 1 | 90767 | 90768 | 0.00333333 | 5273.065382 | 5273.065386 |
| 5273.065449 | Kernel\_Read\|xilinx:adm-pcie-7v3:1ddr:3.0-0\|cnn\_GOOD | Read | OCL Region | 1 | 1 | 90787 | 90788 | 0.00333333 | 5273.065449 | 5273.065452 |
| 5273.065516 | Kernel\_Read\|xilinx:adm-pcie-7v3:1ddr:3.0-0\|cnn\_GOOD | Read | OCL Region | 1 | 1 | 90807 | 90808 | 0.00333333 | 5273.065516 | 5273.065519 |
| 5273.065582 | Kernel\_Read\|xilinx:adm-pcie-7v3:1ddr:3.0-0\|cnn\_GOOD | Read | OCL Region | 1 | 1 | 90827 | 90828 | 0.00333333 | 5273.065582 | 5273.065586 |
| 5273.065649 | Kernel\_Read\|xilinx:adm-pcie-7v3:1ddr:3.0-0\|cnn\_GOOD | Read | OCL Region | 1 | 1 | 90847 | 90848 | 0.00333333 | 5273.065649 | 5273.065652 |
| 5273.065716 | Kernel\_Read\|xilinx:adm-pcie-7v3:1ddr:3.0-0\|cnn\_GOOD | Read | OCL Region | 1 | 1 | 90867 | 90868 | 0.00333333 | 5273.065716 | 5273.065719 |
| 5273.065782 | Kernel\_Read\|xilinx:adm-pcie-7v3:1ddr:3.0-0\|cnn\_GOOD | Read | OCL Region | 1 | 1 | 90887 | 90888 | 0.00333333 | 5273.065782 | 5273.065786 |
| 5273.065849 | Kernel\_Read\|xilinx:adm-pcie-7v3:1ddr:3.0-0\|cnn\_GOOD | Read | OCL Region | 1 | 1 | 90907 | 90908 | 0.00333333 | 5273.065849 | 5273.065852 |
| 5273.065916 | Kernel\_Read\|xilinx:adm-pcie-7v3:1ddr:3.0-0\|cnn\_GOOD | Read | OCL Region | 1 | 1 | 90927 | 90928 | 0.00333333 | 5273.065916 | 5273.065919 |
| 5273.065982 | Kernel\_Read\|xilinx:adm-pcie-7v3:1ddr:3.0-0\|cnn\_GOOD | Read | OCL Region | 1 | 1 | 90947 | 90948 | 0.00333333 | 5273.065982 | 5273.065986 |
| 5273.066049 | Kernel\_Read\|xilinx:adm-pcie-7v3:1ddr:3.0-0\|cnn\_GOOD | Read | OCL Region | 1 | 1 | 90967 | 90968 | 0.00333333 | 5273.066049 | 5273.066052 |
| 5273.066116 | Kernel\_Read\|xilinx:adm-pcie-7v3:1ddr:3.0-0\|cnn\_GOOD | Read | OCL Region | 1 | 1 | 90987 | 90988 | 0.00333333 | 5273.066116 | 5273.066119 |
| 5273.066182 | Kernel\_Read\|xilinx:adm-pcie-7v3:1ddr:3.0-0\|cnn\_GOOD | Read | OCL Region | 1 | 1 | 91007 | 91008 | 0.00333333 | 5273.066182 | 5273.066186 |
| 5273.066249 | Kernel\_Read\|xilinx:adm-pcie-7v3:1ddr:3.0-0\|cnn\_GOOD | Read | OCL Region | 1 | 1 | 91027 | 91028 | 0.00333333 | 5273.066249 | 5273.066252 |
| 5273.066316 | Kernel\_Read\|xilinx:adm-pcie-7v3:1ddr:3.0-0\|cnn\_GOOD | Read | OCL Region | 1 | 1 | 91047 | 91048 | 0.00333333 | 5273.066316 | 5273.066319 |
| 5273.066382 | Kernel\_Read\|xilinx:adm-pcie-7v3:1ddr:3.0-0\|cnn\_GOOD | Read | OCL Region | 1 | 1 | 91067 | 91068 | 0.00333333 | 5273.066382 | 5273.066386 |
| 5273.066449 | Kernel\_Read\|xilinx:adm-pcie-7v3:1ddr:3.0-0\|cnn\_GOOD | Read | OCL Region | 1 | 1 | 91087 | 91088 | 0.00333333 | 5273.066449 | 5273.066452 |
| 5273.066516 | Kernel\_Read\|xilinx:adm-pcie-7v3:1ddr:3.0-0\|cnn\_GOOD | Read | OCL Region | 1 | 1 | 91107 | 91108 | 0.00333333 | 5273.066516 | 5273.066519 |
| 5273.066582 | Kernel\_Read\|xilinx:adm-pcie-7v3:1ddr:3.0-0\|cnn\_GOOD | Read | OCL Region | 1 | 1 | 91127 | 91128 | 0.00333333 | 5273.066582 | 5273.066586 |
| 5273.066649 | Kernel\_Read\|xilinx:adm-pcie-7v3:1ddr:3.0-0\|cnn\_GOOD | Read | OCL Region | 1 | 1 | 91147 | 91148 | 0.00333333 | 5273.066649 | 5273.066652 |
| 5273.066716 | Kernel\_Read\|xilinx:adm-pcie-7v3:1ddr:3.0-0\|cnn\_GOOD | Read | OCL Region | 1 | 1 | 91167 | 91168 | 0.00333333 | 5273.066716 | 5273.066719 |
| 5273.066782 | Kernel\_Read\|xilinx:adm-pcie-7v3:1ddr:3.0-0\|cnn\_GOOD | Read | OCL Region | 1 | 1 | 91187 | 91188 | 0.00333333 | 5273.066782 | 5273.066786 |
| 5273.066849 | Kernel\_Read\|xilinx:adm-pcie-7v3:1ddr:3.0-0\|cnn\_GOOD | Read | OCL Region | 1 | 1 | 91207 | 91208 | 0.00333333 | 5273.066849 | 5273.066852 |
| 5273.066916 | Kernel\_Read\|xilinx:adm-pcie-7v3:1ddr:3.0-0\|cnn\_GOOD | Read | OCL Region | 1 | 1 | 91227 | 91228 | 0.00333333 | 5273.066916 | 5273.066919 |
| 5273.066982 | Kernel\_Read\|xilinx:adm-pcie-7v3:1ddr:3.0-0\|cnn\_GOOD | Read | OCL Region | 1 | 1 | 91247 | 91248 | 0.00333333 | 5273.066982 | 5273.066986 |
| 5273.067049 | Kernel\_Read\|xilinx:adm-pcie-7v3:1ddr:3.0-0\|cnn\_GOOD | Read | OCL Region | 1 | 1 | 91267 | 91268 | 0.00333333 | 5273.067049 | 5273.067052 |
| 5273.067116 | Kernel\_Read\|xilinx:adm-pcie-7v3:1ddr:3.0-0\|cnn\_GOOD | Read | OCL Region | 1 | 1 | 91287 | 91288 | 0.00333333 | 5273.067116 | 5273.067119 |
| 5273.067182 | Kernel\_Read\|xilinx:adm-pcie-7v3:1ddr:3.0-0\|cnn\_GOOD | Read | OCL Region | 1 | 1 | 91307 | 91308 | 0.00333333 | 5273.067182 | 5273.067186 |
| 5273.067249 | Kernel\_Read\|xilinx:adm-pcie-7v3:1ddr:3.0-0\|cnn\_GOOD | Read | OCL Region | 1 | 1 | 91327 | 91328 | 0.00333333 | 5273.067249 | 5273.067252 |
| 5273.067316 | Kernel\_Read\|xilinx:adm-pcie-7v3:1ddr:3.0-0\|cnn\_GOOD | Read | OCL Region | 1 | 1 | 91347 | 91348 | 0.00333333 | 5273.067316 | 5273.067319 |
| 5273.067382 | Kernel\_Read\|xilinx:adm-pcie-7v3:1ddr:3.0-0\|cnn\_GOOD | Read | OCL Region | 1 | 1 | 91367 | 91368 | 0.00333333 | 5273.067382 | 5273.067386 |
| 5273.067449 | Kernel\_Read\|xilinx:adm-pcie-7v3:1ddr:3.0-0\|cnn\_GOOD | Read | OCL Region | 1 | 1 | 91387 | 91388 | 0.00333333 | 5273.067449 | 5273.067452 |
| 5273.067512 | Kernel\_Read\|xilinx:adm-pcie-7v3:1ddr:3.0-0\|cnn\_GOOD | Read | OCL Region | 1 | 1 | 91406 | 91407 | 0.00333333 | 5273.067512 | 5273.067516 |
| 5273.067582 | Kernel\_Read\|xilinx:adm-pcie-7v3:1ddr:3.0-0\|cnn\_GOOD | Read | OCL Region | 1 | 1 | 91427 | 91428 | 0.00333333 | 5273.067582 | 5273.067586 |
| 5273.067649 | Kernel\_Read\|xilinx:adm-pcie-7v3:1ddr:3.0-0\|cnn\_GOOD | Read | OCL Region | 1 | 1 | 91447 | 91448 | 0.00333333 | 5273.067649 | 5273.067652 |
| 5273.067716 | Kernel\_Read\|xilinx:adm-pcie-7v3:1ddr:3.0-0\|cnn\_GOOD | Read | OCL Region | 1 | 1 | 91467 | 91468 | 0.00333333 | 5273.067716 | 5273.067719 |
| 5273.067782 | Kernel\_Read\|xilinx:adm-pcie-7v3:1ddr:3.0-0\|cnn\_GOOD | Read | OCL Region | 1 | 1 | 91487 | 91488 | 0.00333333 | 5273.067782 | 5273.067786 |
| 5273.068059 | Kernel\_Read\|xilinx:adm-pcie-7v3:1ddr:3.0-0\|cnn\_GOOD | Read | OCL Region | 1 | 1 | 91570 | 91571 | 0.00333333 | 5273.068059 | 5273.068062 |
| 5273.068062 | Kernel\_Read\|xilinx:adm-pcie-7v3:1ddr:3.0-0\|cnn\_GOOD | Read | OCL Region | 1 | 1 | 91571 | 91572 | 0.00333333 | 5273.068062 | 5273.068066 |
| 5273.068066 | Kernel\_Read\|xilinx:adm-pcie-7v3:1ddr:3.0-0\|cnn\_GOOD | Read | OCL Region | 1 | 1 | 91572 | 91573 | 0.00333333 | 5273.068066 | 5273.068069 |
| 5273.068076 | Kernel\_Read\|xilinx:adm-pcie-7v3:1ddr:3.0-0\|cnn\_GOOD | Read | OCL Region | 1 | 1 | 91575 | 91576 | 0.00333333 | 5273.068076 | 5273.068079 |
| 5273.068132 | Kernel\_Read\|xilinx:adm-pcie-7v3:1ddr:3.0-0\|cnn\_GOOD | Read | OCL Region | 1 | 1 | 91592 | 91593 | 0.00333333 | 5273.068132 | 5273.068136 |
| 5273.068189 | Kernel\_Read\|xilinx:adm-pcie-7v3:1ddr:3.0-0\|cnn\_GOOD | Read | OCL Region | 1 | 1 | 91609 | 91610 | 0.00333333 | 5273.068189 | 5273.068192 |
| 5273.068249 | Kernel\_Read\|xilinx:adm-pcie-7v3:1ddr:3.0-0\|cnn\_GOOD | Read | OCL Region | 1 | 1 | 91627 | 91628 | 0.00333333 | 5273.068249 | 5273.068252 |
| 5273.068316 | Kernel\_Read\|xilinx:adm-pcie-7v3:1ddr:3.0-0\|cnn\_GOOD | Read | OCL Region | 1 | 1 | 91647 | 91648 | 0.00333333 | 5273.068316 | 5273.068319 |
| 5273.068382 | Kernel\_Read\|xilinx:adm-pcie-7v3:1ddr:3.0-0\|cnn\_GOOD | Read | OCL Region | 1 | 1 | 91667 | 91668 | 0.00333333 | 5273.068382 | 5273.068386 |
| 5273.068449 | Kernel\_Read\|xilinx:adm-pcie-7v3:1ddr:3.0-0\|cnn\_GOOD | Read | OCL Region | 1 | 1 | 91687 | 91688 | 0.00333333 | 5273.068449 | 5273.068452 |
| 5273.068516 | Kernel\_Read\|xilinx:adm-pcie-7v3:1ddr:3.0-0\|cnn\_GOOD | Read | OCL Region | 1 | 1 | 91707 | 91708 | 0.00333333 | 5273.068516 | 5273.068519 |
| 5273.068582 | Kernel\_Read\|xilinx:adm-pcie-7v3:1ddr:3.0-0\|cnn\_GOOD | Read | OCL Region | 1 | 1 | 91727 | 91728 | 0.00333333 | 5273.068582 | 5273.068586 |
| 5273.068649 | Kernel\_Read\|xilinx:adm-pcie-7v3:1ddr:3.0-0\|cnn\_GOOD | Read | OCL Region | 1 | 1 | 91747 | 91748 | 0.00333333 | 5273.068649 | 5273.068652 |
| 5273.068716 | Kernel\_Read\|xilinx:adm-pcie-7v3:1ddr:3.0-0\|cnn\_GOOD | Read | OCL Region | 1 | 1 | 91767 | 91768 | 0.00333333 | 5273.068716 | 5273.068719 |
| 5273.068782 | Kernel\_Read\|xilinx:adm-pcie-7v3:1ddr:3.0-0\|cnn\_GOOD | Read | OCL Region | 1 | 1 | 91787 | 91788 | 0.00333333 | 5273.068782 | 5273.068786 |
| 5273.068856 | Kernel\_Read\|xilinx:adm-pcie-7v3:1ddr:3.0-0\|cnn\_GOOD | Read | OCL Region | 1 | 1 | 91809 | 91810 | 0.00333333 | 5273.068856 | 5273.068859 |
| 5273.068916 | Kernel\_Read\|xilinx:adm-pcie-7v3:1ddr:3.0-0\|cnn\_GOOD | Read | OCL Region | 1 | 1 | 91827 | 91828 | 0.00333333 | 5273.068916 | 5273.068919 |
| 5273.068982 | Kernel\_Read\|xilinx:adm-pcie-7v3:1ddr:3.0-0\|cnn\_GOOD | Read | OCL Region | 1 | 1 | 91847 | 91848 | 0.00333333 | 5273.068982 | 5273.068986 |
| 5273.069049 | Kernel\_Read\|xilinx:adm-pcie-7v3:1ddr:3.0-0\|cnn\_GOOD | Read | OCL Region | 1 | 1 | 91867 | 91868 | 0.00333333 | 5273.069049 | 5273.069052 |
| 5273.069116 | Kernel\_Read\|xilinx:adm-pcie-7v3:1ddr:3.0-0\|cnn\_GOOD | Read | OCL Region | 1 | 1 | 91887 | 91888 | 0.00333333 | 5273.069116 | 5273.069119 |
| 5273.069182 | Kernel\_Read\|xilinx:adm-pcie-7v3:1ddr:3.0-0\|cnn\_GOOD | Read | OCL Region | 1 | 1 | 91907 | 91908 | 0.00333333 | 5273.069182 | 5273.069186 |
| 5273.069249 | Kernel\_Read\|xilinx:adm-pcie-7v3:1ddr:3.0-0\|cnn\_GOOD | Read | OCL Region | 1 | 1 | 91927 | 91928 | 0.00333333 | 5273.069249 | 5273.069252 |
| 5273.069316 | Kernel\_Read\|xilinx:adm-pcie-7v3:1ddr:3.0-0\|cnn\_GOOD | Read | OCL Region | 1 | 1 | 91947 | 91948 | 0.00333333 | 5273.069316 | 5273.069319 |
| 5273.069382 | Kernel\_Read\|xilinx:adm-pcie-7v3:1ddr:3.0-0\|cnn\_GOOD | Read | OCL Region | 1 | 1 | 91967 | 91968 | 0.00333333 | 5273.069382 | 5273.069386 |
| 5273.069449 | Kernel\_Read\|xilinx:adm-pcie-7v3:1ddr:3.0-0\|cnn\_GOOD | Read | OCL Region | 1 | 1 | 91987 | 91988 | 0.00333333 | 5273.069449 | 5273.069452 |
| 5273.069516 | Kernel\_Read\|xilinx:adm-pcie-7v3:1ddr:3.0-0\|cnn\_GOOD | Read | OCL Region | 1 | 1 | 92007 | 92008 | 0.00333333 | 5273.069516 | 5273.069519 |
| 5273.069582 | Kernel\_Read\|xilinx:adm-pcie-7v3:1ddr:3.0-0\|cnn\_GOOD | Read | OCL Region | 1 | 1 | 92027 | 92028 | 0.00333333 | 5273.069582 | 5273.069586 |
| 5273.069649 | Kernel\_Read\|xilinx:adm-pcie-7v3:1ddr:3.0-0\|cnn\_GOOD | Read | OCL Region | 1 | 1 | 92047 | 92048 | 0.00333333 | 5273.069649 | 5273.069652 |
| 5273.069716 | Kernel\_Read\|xilinx:adm-pcie-7v3:1ddr:3.0-0\|cnn\_GOOD | Read | OCL Region | 1 | 1 | 92067 | 92068 | 0.00333333 | 5273.069716 | 5273.069719 |
| 5273.069782 | Kernel\_Read\|xilinx:adm-pcie-7v3:1ddr:3.0-0\|cnn\_GOOD | Read | OCL Region | 1 | 1 | 92087 | 92088 | 0.00333333 | 5273.069782 | 5273.069786 |
| 5273.069849 | Kernel\_Read\|xilinx:adm-pcie-7v3:1ddr:3.0-0\|cnn\_GOOD | Read | OCL Region | 1 | 1 | 92107 | 92108 | 0.00333333 | 5273.069849 | 5273.069852 |
| 5273.069916 | Kernel\_Read\|xilinx:adm-pcie-7v3:1ddr:3.0-0\|cnn\_GOOD | Read | OCL Region | 1 | 1 | 92127 | 92128 | 0.00333333 | 5273.069916 | 5273.069919 |
| 5273.069982 | Kernel\_Read\|xilinx:adm-pcie-7v3:1ddr:3.0-0\|cnn\_GOOD | Read | OCL Region | 1 | 1 | 92147 | 92148 | 0.00333333 | 5273.069982 | 5273.069986 |
| 5273.070049 | Kernel\_Read\|xilinx:adm-pcie-7v3:1ddr:3.0-0\|cnn\_GOOD | Read | OCL Region | 1 | 1 | 92167 | 92168 | 0.00333333 | 5273.070049 | 5273.070052 |
| 5273.070116 | Kernel\_Read\|xilinx:adm-pcie-7v3:1ddr:3.0-0\|cnn\_GOOD | Read | OCL Region | 1 | 1 | 92187 | 92188 | 0.00333333 | 5273.070116 | 5273.070119 |
| 5273.070182 | Kernel\_Read\|xilinx:adm-pcie-7v3:1ddr:3.0-0\|cnn\_GOOD | Read | OCL Region | 1 | 1 | 92207 | 92208 | 0.00333333 | 5273.070182 | 5273.070186 |
| 5273.070249 | Kernel\_Read\|xilinx:adm-pcie-7v3:1ddr:3.0-0\|cnn\_GOOD | Read | OCL Region | 1 | 1 | 92227 | 92228 | 0.00333333 | 5273.070249 | 5273.070252 |
| 5273.070316 | Kernel\_Read\|xilinx:adm-pcie-7v3:1ddr:3.0-0\|cnn\_GOOD | Read | OCL Region | 1 | 1 | 92247 | 92248 | 0.00333333 | 5273.070316 | 5273.070319 |
| 5273.070382 | Kernel\_Read\|xilinx:adm-pcie-7v3:1ddr:3.0-0\|cnn\_GOOD | Read | OCL Region | 1 | 1 | 92267 | 92268 | 0.00333333 | 5273.070382 | 5273.070386 |
| 5273.070449 | Kernel\_Read\|xilinx:adm-pcie-7v3:1ddr:3.0-0\|cnn\_GOOD | Read | OCL Region | 1 | 1 | 92287 | 92288 | 0.00333333 | 5273.070449 | 5273.070452 |
| 5273.070516 | Kernel\_Read\|xilinx:adm-pcie-7v3:1ddr:3.0-0\|cnn\_GOOD | Read | OCL Region | 1 | 1 | 92307 | 92308 | 0.00333333 | 5273.070516 | 5273.070519 |
| 5273.070582 | Kernel\_Read\|xilinx:adm-pcie-7v3:1ddr:3.0-0\|cnn\_GOOD | Read | OCL Region | 1 | 1 | 92327 | 92328 | 0.00333333 | 5273.070582 | 5273.070586 |
| 5273.070649 | Kernel\_Read\|xilinx:adm-pcie-7v3:1ddr:3.0-0\|cnn\_GOOD | Read | OCL Region | 1 | 1 | 92347 | 92348 | 0.00333333 | 5273.070649 | 5273.070652 |
| 5273.070716 | Kernel\_Read\|xilinx:adm-pcie-7v3:1ddr:3.0-0\|cnn\_GOOD | Read | OCL Region | 1 | 1 | 92367 | 92368 | 0.00333333 | 5273.070716 | 5273.070719 |
| 5273.070782 | Kernel\_Read\|xilinx:adm-pcie-7v3:1ddr:3.0-0\|cnn\_GOOD | Read | OCL Region | 1 | 1 | 92387 | 92388 | 0.00333333 | 5273.070782 | 5273.070786 |
| 5273.070849 | Kernel\_Read\|xilinx:adm-pcie-7v3:1ddr:3.0-0\|cnn\_GOOD | Read | OCL Region | 1 | 1 | 92407 | 92408 | 0.00333333 | 5273.070849 | 5273.070852 |
| 5273.070916 | Kernel\_Read\|xilinx:adm-pcie-7v3:1ddr:3.0-0\|cnn\_GOOD | Read | OCL Region | 1 | 1 | 92427 | 92428 | 0.00333333 | 5273.070916 | 5273.070919 |
| 5273.070982 | Kernel\_Read\|xilinx:adm-pcie-7v3:1ddr:3.0-0\|cnn\_GOOD | Read | OCL Region | 1 | 1 | 92447 | 92448 | 0.00333333 | 5273.070982 | 5273.070986 |
| 5273.071049 | Kernel\_Read\|xilinx:adm-pcie-7v3:1ddr:3.0-0\|cnn\_GOOD | Read | OCL Region | 1 | 1 | 92467 | 92468 | 0.00333333 | 5273.071049 | 5273.071052 |
| 5273.071116 | Kernel\_Read\|xilinx:adm-pcie-7v3:1ddr:3.0-0\|cnn\_GOOD | Read | OCL Region | 1 | 1 | 92487 | 92488 | 0.00333333 | 5273.071116 | 5273.071119 |
| 5273.071182 | Kernel\_Read\|xilinx:adm-pcie-7v3:1ddr:3.0-0\|cnn\_GOOD | Read | OCL Region | 1 | 1 | 92507 | 92508 | 0.00333333 | 5273.071182 | 5273.071186 |
| 5273.071249 | Kernel\_Read\|xilinx:adm-pcie-7v3:1ddr:3.0-0\|cnn\_GOOD | Read | OCL Region | 1 | 1 | 92527 | 92528 | 0.00333333 | 5273.071249 | 5273.071252 |
| 5273.071329 | Kernel\_Read\|xilinx:adm-pcie-7v3:1ddr:3.0-0\|cnn\_GOOD | Read | OCL Region | 1 | 1 | 92551 | 92552 | 0.00333333 | 5273.071329 | 5273.071332 |
| 5273.071382 | Kernel\_Read\|xilinx:adm-pcie-7v3:1ddr:3.0-0\|cnn\_GOOD | Read | OCL Region | 1 | 1 | 92567 | 92568 | 0.00333333 | 5273.071382 | 5273.071386 |
| 5273.071449 | Kernel\_Read\|xilinx:adm-pcie-7v3:1ddr:3.0-0\|cnn\_GOOD | Read | OCL Region | 1 | 1 | 92587 | 92588 | 0.00333333 | 5273.071449 | 5273.071452 |
| 5273.071516 | Kernel\_Read\|xilinx:adm-pcie-7v3:1ddr:3.0-0\|cnn\_GOOD | Read | OCL Region | 1 | 1 | 92607 | 92608 | 0.00333333 | 5273.071516 | 5273.071519 |
| 5273.071582 | Kernel\_Read\|xilinx:adm-pcie-7v3:1ddr:3.0-0\|cnn\_GOOD | Read | OCL Region | 1 | 1 | 92627 | 92628 | 0.00333333 | 5273.071582 | 5273.071586 |
| 5273.071649 | Kernel\_Read\|xilinx:adm-pcie-7v3:1ddr:3.0-0\|cnn\_GOOD | Read | OCL Region | 1 | 1 | 92647 | 92648 | 0.00333333 | 5273.071649 | 5273.071652 |
| 5273.071716 | Kernel\_Read\|xilinx:adm-pcie-7v3:1ddr:3.0-0\|cnn\_GOOD | Read | OCL Region | 1 | 1 | 92667 | 92668 | 0.00333333 | 5273.071716 | 5273.071719 |
| 5273.071782 | Kernel\_Read\|xilinx:adm-pcie-7v3:1ddr:3.0-0\|cnn\_GOOD | Read | OCL Region | 1 | 1 | 92687 | 92688 | 0.00333333 | 5273.071782 | 5273.071786 |
| 5273.071849 | Kernel\_Read\|xilinx:adm-pcie-7v3:1ddr:3.0-0\|cnn\_GOOD | Read | OCL Region | 1 | 1 | 92707 | 92708 | 0.00333333 | 5273.071849 | 5273.071852 |
| 5273.071916 | Kernel\_Read\|xilinx:adm-pcie-7v3:1ddr:3.0-0\|cnn\_GOOD | Read | OCL Region | 1 | 1 | 92727 | 92728 | 0.00333333 | 5273.071916 | 5273.071919 |
| 5273.071982 | Kernel\_Read\|xilinx:adm-pcie-7v3:1ddr:3.0-0\|cnn\_GOOD | Read | OCL Region | 1 | 1 | 92747 | 92748 | 0.00333333 | 5273.071982 | 5273.071986 |
| 5273.072049 | Kernel\_Read\|xilinx:adm-pcie-7v3:1ddr:3.0-0\|cnn\_GOOD | Read | OCL Region | 1 | 1 | 92767 | 92768 | 0.00333333 | 5273.072049 | 5273.072052 |
| 5273.072116 | Kernel\_Read\|xilinx:adm-pcie-7v3:1ddr:3.0-0\|cnn\_GOOD | Read | OCL Region | 1 | 1 | 92787 | 92788 | 0.00333333 | 5273.072116 | 5273.072119 |
| 5273.072182 | Kernel\_Read\|xilinx:adm-pcie-7v3:1ddr:3.0-0\|cnn\_GOOD | Read | OCL Region | 1 | 1 | 92807 | 92808 | 0.00333333 | 5273.072182 | 5273.072186 |
| 5273.072249 | Kernel\_Read\|xilinx:adm-pcie-7v3:1ddr:3.0-0\|cnn\_GOOD | Read | OCL Region | 1 | 1 | 92827 | 92828 | 0.00333333 | 5273.072249 | 5273.072252 |
| 5273.072316 | Kernel\_Read\|xilinx:adm-pcie-7v3:1ddr:3.0-0\|cnn\_GOOD | Read | OCL Region | 1 | 1 | 92847 | 92848 | 0.00333333 | 5273.072316 | 5273.072319 |
| 5273.072382 | Kernel\_Read\|xilinx:adm-pcie-7v3:1ddr:3.0-0\|cnn\_GOOD | Read | OCL Region | 1 | 1 | 92867 | 92868 | 0.00333333 | 5273.072382 | 5273.072386 |
| 5273.072449 | Kernel\_Read\|xilinx:adm-pcie-7v3:1ddr:3.0-0\|cnn\_GOOD | Read | OCL Region | 1 | 1 | 92887 | 92888 | 0.00333333 | 5273.072449 | 5273.072452 |
| 5273.072516 | Kernel\_Read\|xilinx:adm-pcie-7v3:1ddr:3.0-0\|cnn\_GOOD | Read | OCL Region | 1 | 1 | 92907 | 92908 | 0.00333333 | 5273.072516 | 5273.072519 |
| 5273.072582 | Kernel\_Read\|xilinx:adm-pcie-7v3:1ddr:3.0-0\|cnn\_GOOD | Read | OCL Region | 1 | 1 | 92927 | 92928 | 0.00333333 | 5273.072582 | 5273.072586 |
| 5273.072649 | Kernel\_Read\|xilinx:adm-pcie-7v3:1ddr:3.0-0\|cnn\_GOOD | Read | OCL Region | 1 | 1 | 92947 | 92948 | 0.00333333 | 5273.072649 | 5273.072652 |
| 5273.072716 | Kernel\_Read\|xilinx:adm-pcie-7v3:1ddr:3.0-0\|cnn\_GOOD | Read | OCL Region | 1 | 1 | 92967 | 92968 | 0.00333333 | 5273.072716 | 5273.072719 |
| 5273.072782 | Kernel\_Read\|xilinx:adm-pcie-7v3:1ddr:3.0-0\|cnn\_GOOD | Read | OCL Region | 1 | 1 | 92987 | 92988 | 0.00333333 | 5273.072782 | 5273.072786 |
| 5273.072849 | Kernel\_Read\|xilinx:adm-pcie-7v3:1ddr:3.0-0\|cnn\_GOOD | Read | OCL Region | 1 | 1 | 93007 | 93008 | 0.00333333 | 5273.072849 | 5273.072852 |
| 5273.072916 | Kernel\_Read\|xilinx:adm-pcie-7v3:1ddr:3.0-0\|cnn\_GOOD | Read | OCL Region | 1 | 1 | 93027 | 93028 | 0.00333333 | 5273.072916 | 5273.072919 |
| 5273.072982 | Kernel\_Read\|xilinx:adm-pcie-7v3:1ddr:3.0-0\|cnn\_GOOD | Read | OCL Region | 1 | 1 | 93047 | 93048 | 0.00333333 | 5273.072982 | 5273.072986 |
| 5273.073049 | Kernel\_Read\|xilinx:adm-pcie-7v3:1ddr:3.0-0\|cnn\_GOOD | Read | OCL Region | 1 | 1 | 93067 | 93068 | 0.00333333 | 5273.073049 | 5273.073052 |
| 5273.073116 | Kernel\_Read\|xilinx:adm-pcie-7v3:1ddr:3.0-0\|cnn\_GOOD | Read | OCL Region | 1 | 1 | 93087 | 93088 | 0.00333333 | 5273.073116 | 5273.073119 |
| 5273.073182 | Kernel\_Read\|xilinx:adm-pcie-7v3:1ddr:3.0-0\|cnn\_GOOD | Read | OCL Region | 1 | 1 | 93107 | 93108 | 0.00333333 | 5273.073182 | 5273.073186 |
| 5273.073249 | Kernel\_Read\|xilinx:adm-pcie-7v3:1ddr:3.0-0\|cnn\_GOOD | Read | OCL Region | 1 | 1 | 93127 | 93128 | 0.00333333 | 5273.073249 | 5273.073252 |
| 5273.073316 | Kernel\_Read\|xilinx:adm-pcie-7v3:1ddr:3.0-0\|cnn\_GOOD | Read | OCL Region | 1 | 1 | 93147 | 93148 | 0.00333333 | 5273.073316 | 5273.073319 |
| 5273.073382 | Kernel\_Read\|xilinx:adm-pcie-7v3:1ddr:3.0-0\|cnn\_GOOD | Read | OCL Region | 1 | 1 | 93167 | 93168 | 0.00333333 | 5273.073382 | 5273.073386 |
| 5273.073449 | Kernel\_Read\|xilinx:adm-pcie-7v3:1ddr:3.0-0\|cnn\_GOOD | Read | OCL Region | 1 | 1 | 93187 | 93188 | 0.00333333 | 5273.073449 | 5273.073452 |
| 5273.073516 | Kernel\_Read\|xilinx:adm-pcie-7v3:1ddr:3.0-0\|cnn\_GOOD | Read | OCL Region | 1 | 1 | 93207 | 93208 | 0.00333333 | 5273.073516 | 5273.073519 |
| 5273.073582 | Kernel\_Read\|xilinx:adm-pcie-7v3:1ddr:3.0-0\|cnn\_GOOD | Read | OCL Region | 1 | 1 | 93227 | 93228 | 0.00333333 | 5273.073582 | 5273.073586 |
| 5273.073649 | Kernel\_Read\|xilinx:adm-pcie-7v3:1ddr:3.0-0\|cnn\_GOOD | Read | OCL Region | 1 | 1 | 93247 | 93248 | 0.00333333 | 5273.073649 | 5273.073652 |
| 5273.073716 | Kernel\_Read\|xilinx:adm-pcie-7v3:1ddr:3.0-0\|cnn\_GOOD | Read | OCL Region | 1 | 1 | 93267 | 93268 | 0.00333333 | 5273.073716 | 5273.073719 |
| 5273.073782 | Kernel\_Read\|xilinx:adm-pcie-7v3:1ddr:3.0-0\|cnn\_GOOD | Read | OCL Region | 1 | 1 | 93287 | 93288 | 0.00333333 | 5273.073782 | 5273.073786 |
| 5273.073849 | Kernel\_Read\|xilinx:adm-pcie-7v3:1ddr:3.0-0\|cnn\_GOOD | Read | OCL Region | 1 | 1 | 93307 | 93308 | 0.00333333 | 5273.073849 | 5273.073852 |
| 5273.073916 | Kernel\_Read\|xilinx:adm-pcie-7v3:1ddr:3.0-0\|cnn\_GOOD | Read | OCL Region | 1 | 1 | 93327 | 93328 | 0.00333333 | 5273.073916 | 5273.073919 |
| 5273.073982 | Kernel\_Read\|xilinx:adm-pcie-7v3:1ddr:3.0-0\|cnn\_GOOD | Read | OCL Region | 1 | 1 | 93347 | 93348 | 0.00333333 | 5273.073982 | 5273.073986 |
| 5273.074049 | Kernel\_Read\|xilinx:adm-pcie-7v3:1ddr:3.0-0\|cnn\_GOOD | Read | OCL Region | 1 | 1 | 93367 | 93368 | 0.00333333 | 5273.074049 | 5273.074052 |
| 5273.074326 | Kernel\_Read\|xilinx:adm-pcie-7v3:1ddr:3.0-0\|cnn\_GOOD | Read | OCL Region | 1 | 1 | 93450 | 93451 | 0.00333333 | 5273.074326 | 5273.074329 |
| 5273.074329 | Kernel\_Read\|xilinx:adm-pcie-7v3:1ddr:3.0-0\|cnn\_GOOD | Read | OCL Region | 1 | 1 | 93451 | 93452 | 0.00333333 | 5273.074329 | 5273.074332 |
| 5273.074332 | Kernel\_Read\|xilinx:adm-pcie-7v3:1ddr:3.0-0\|cnn\_GOOD | Read | OCL Region | 1 | 1 | 93452 | 93453 | 0.00333333 | 5273.074332 | 5273.074336 |
| 5273.074339 | Kernel\_Read\|xilinx:adm-pcie-7v3:1ddr:3.0-0\|cnn\_GOOD | Read | OCL Region | 1 | 1 | 93454 | 93455 | 0.00333333 | 5273.074339 | 5273.074342 |
| 5273.074399 | Kernel\_Read\|xilinx:adm-pcie-7v3:1ddr:3.0-0\|cnn\_GOOD | Read | OCL Region | 1 | 1 | 93472 | 93473 | 0.00333333 | 5273.074399 | 5273.074402 |
| 5273.074456 | Kernel\_Read\|xilinx:adm-pcie-7v3:1ddr:3.0-0\|cnn\_GOOD | Read | OCL Region | 1 | 1 | 93489 | 93490 | 0.00333333 | 5273.074456 | 5273.074459 |
| 5273.074516 | Kernel\_Read\|xilinx:adm-pcie-7v3:1ddr:3.0-0\|cnn\_GOOD | Read | OCL Region | 1 | 1 | 93507 | 93508 | 0.00333333 | 5273.074516 | 5273.074519 |
| 5273.074582 | Kernel\_Read\|xilinx:adm-pcie-7v3:1ddr:3.0-0\|cnn\_GOOD | Read | OCL Region | 1 | 1 | 93527 | 93528 | 0.00333333 | 5273.074582 | 5273.074586 |
| 5273.074649 | Kernel\_Read\|xilinx:adm-pcie-7v3:1ddr:3.0-0\|cnn\_GOOD | Read | OCL Region | 1 | 1 | 93547 | 93548 | 0.00333333 | 5273.074649 | 5273.074652 |
| 5273.074716 | Kernel\_Read\|xilinx:adm-pcie-7v3:1ddr:3.0-0\|cnn\_GOOD | Read | OCL Region | 1 | 1 | 93567 | 93568 | 0.00333333 | 5273.074716 | 5273.074719 |
| 5273.074782 | Kernel\_Read\|xilinx:adm-pcie-7v3:1ddr:3.0-0\|cnn\_GOOD | Read | OCL Region | 1 | 1 | 93587 | 93588 | 0.00333333 | 5273.074782 | 5273.074786 |
| 5273.074849 | Kernel\_Read\|xilinx:adm-pcie-7v3:1ddr:3.0-0\|cnn\_GOOD | Read | OCL Region | 1 | 1 | 93607 | 93608 | 0.00333333 | 5273.074849 | 5273.074852 |
| 5273.074916 | Kernel\_Read\|xilinx:adm-pcie-7v3:1ddr:3.0-0\|cnn\_GOOD | Read | OCL Region | 1 | 1 | 93627 | 93628 | 0.00333333 | 5273.074916 | 5273.074919 |
| 5273.074982 | Kernel\_Read\|xilinx:adm-pcie-7v3:1ddr:3.0-0\|cnn\_GOOD | Read | OCL Region | 1 | 1 | 93647 | 93648 | 0.00333333 | 5273.074982 | 5273.074986 |
| 5273.075049 | Kernel\_Read\|xilinx:adm-pcie-7v3:1ddr:3.0-0\|cnn\_GOOD | Read | OCL Region | 1 | 1 | 93667 | 93668 | 0.00333333 | 5273.075049 | 5273.075052 |
| 5273.075126 | Kernel\_Read\|xilinx:adm-pcie-7v3:1ddr:3.0-0\|cnn\_GOOD | Read | OCL Region | 1 | 1 | 93690 | 93691 | 0.00333333 | 5273.075126 | 5273.075129 |
| 5273.075182 | Kernel\_Read\|xilinx:adm-pcie-7v3:1ddr:3.0-0\|cnn\_GOOD | Read | OCL Region | 1 | 1 | 93707 | 93708 | 0.00333333 | 5273.075182 | 5273.075186 |
| 5273.075249 | Kernel\_Read\|xilinx:adm-pcie-7v3:1ddr:3.0-0\|cnn\_GOOD | Read | OCL Region | 1 | 1 | 93727 | 93728 | 0.00333333 | 5273.075249 | 5273.075252 |
| 5273.075316 | Kernel\_Read\|xilinx:adm-pcie-7v3:1ddr:3.0-0\|cnn\_GOOD | Read | OCL Region | 1 | 1 | 93747 | 93748 | 0.00333333 | 5273.075316 | 5273.075319 |
| 5273.075382 | Kernel\_Read\|xilinx:adm-pcie-7v3:1ddr:3.0-0\|cnn\_GOOD | Read | OCL Region | 1 | 1 | 93767 | 93768 | 0.00333333 | 5273.075382 | 5273.075386 |
| 5273.075449 | Kernel\_Read\|xilinx:adm-pcie-7v3:1ddr:3.0-0\|cnn\_GOOD | Read | OCL Region | 1 | 1 | 93787 | 93788 | 0.00333333 | 5273.075449 | 5273.075452 |
| 5273.075516 | Kernel\_Read\|xilinx:adm-pcie-7v3:1ddr:3.0-0\|cnn\_GOOD | Read | OCL Region | 1 | 1 | 93807 | 93808 | 0.00333333 | 5273.075516 | 5273.075519 |
| 5273.075582 | Kernel\_Read\|xilinx:adm-pcie-7v3:1ddr:3.0-0\|cnn\_GOOD | Read | OCL Region | 1 | 1 | 93827 | 93828 | 0.00333333 | 5273.075582 | 5273.075586 |
| 5273.075649 | Kernel\_Read\|xilinx:adm-pcie-7v3:1ddr:3.0-0\|cnn\_GOOD | Read | OCL Region | 1 | 1 | 93847 | 93848 | 0.00333333 | 5273.075649 | 5273.075652 |
| 5273.075716 | Kernel\_Read\|xilinx:adm-pcie-7v3:1ddr:3.0-0\|cnn\_GOOD | Read | OCL Region | 1 | 1 | 93867 | 93868 | 0.00333333 | 5273.075716 | 5273.075719 |
| 5273.075782 | Kernel\_Read\|xilinx:adm-pcie-7v3:1ddr:3.0-0\|cnn\_GOOD | Read | OCL Region | 1 | 1 | 93887 | 93888 | 0.00333333 | 5273.075782 | 5273.075786 |
| 5273.075849 | Kernel\_Read\|xilinx:adm-pcie-7v3:1ddr:3.0-0\|cnn\_GOOD | Read | OCL Region | 1 | 1 | 93907 | 93908 | 0.00333333 | 5273.075849 | 5273.075852 |
| 5273.075912 | Kernel\_Read\|xilinx:adm-pcie-7v3:1ddr:3.0-0\|cnn\_GOOD | Read | OCL Region | 1 | 1 | 93926 | 93927 | 0.00333333 | 5273.075912 | 5273.075916 |
| 5273.075982 | Kernel\_Read\|xilinx:adm-pcie-7v3:1ddr:3.0-0\|cnn\_GOOD | Read | OCL Region | 1 | 1 | 93947 | 93948 | 0.00333333 | 5273.075982 | 5273.075986 |
| 5273.076049 | Kernel\_Read\|xilinx:adm-pcie-7v3:1ddr:3.0-0\|cnn\_GOOD | Read | OCL Region | 1 | 1 | 93967 | 93968 | 0.00333333 | 5273.076049 | 5273.076052 |
| 5273.076116 | Kernel\_Read\|xilinx:adm-pcie-7v3:1ddr:3.0-0\|cnn\_GOOD | Read | OCL Region | 1 | 1 | 93987 | 93988 | 0.00333333 | 5273.076116 | 5273.076119 |
| 5273.076182 | Kernel\_Read\|xilinx:adm-pcie-7v3:1ddr:3.0-0\|cnn\_GOOD | Read | OCL Region | 1 | 1 | 94007 | 94008 | 0.00333333 | 5273.076182 | 5273.076186 |
| 5273.076249 | Kernel\_Read\|xilinx:adm-pcie-7v3:1ddr:3.0-0\|cnn\_GOOD | Read | OCL Region | 1 | 1 | 94027 | 94028 | 0.00333333 | 5273.076249 | 5273.076252 |
| 5273.076316 | Kernel\_Read\|xilinx:adm-pcie-7v3:1ddr:3.0-0\|cnn\_GOOD | Read | OCL Region | 1 | 1 | 94047 | 94048 | 0.00333333 | 5273.076316 | 5273.076319 |
| 5273.076382 | Kernel\_Read\|xilinx:adm-pcie-7v3:1ddr:3.0-0\|cnn\_GOOD | Read | OCL Region | 1 | 1 | 94067 | 94068 | 0.00333333 | 5273.076382 | 5273.076386 |
| 5273.076449 | Kernel\_Read\|xilinx:adm-pcie-7v3:1ddr:3.0-0\|cnn\_GOOD | Read | OCL Region | 1 | 1 | 94087 | 94088 | 0.00333333 | 5273.076449 | 5273.076452 |
| 5273.076516 | Kernel\_Read\|xilinx:adm-pcie-7v3:1ddr:3.0-0\|cnn\_GOOD | Read | OCL Region | 1 | 1 | 94107 | 94108 | 0.00333333 | 5273.076516 | 5273.076519 |
| 5273.076582 | Kernel\_Read\|xilinx:adm-pcie-7v3:1ddr:3.0-0\|cnn\_GOOD | Read | OCL Region | 1 | 1 | 94127 | 94128 | 0.00333333 | 5273.076582 | 5273.076586 |
| 5273.076649 | Kernel\_Read\|xilinx:adm-pcie-7v3:1ddr:3.0-0\|cnn\_GOOD | Read | OCL Region | 1 | 1 | 94147 | 94148 | 0.00333333 | 5273.076649 | 5273.076652 |
| 5273.076716 | Kernel\_Read\|xilinx:adm-pcie-7v3:1ddr:3.0-0\|cnn\_GOOD | Read | OCL Region | 1 | 1 | 94167 | 94168 | 0.00333333 | 5273.076716 | 5273.076719 |
| 5273.076782 | Kernel\_Read\|xilinx:adm-pcie-7v3:1ddr:3.0-0\|cnn\_GOOD | Read | OCL Region | 1 | 1 | 94187 | 94188 | 0.00333333 | 5273.076782 | 5273.076786 |
| 5273.076849 | Kernel\_Read\|xilinx:adm-pcie-7v3:1ddr:3.0-0\|cnn\_GOOD | Read | OCL Region | 1 | 1 | 94207 | 94208 | 0.00333333 | 5273.076849 | 5273.076852 |
| 5273.076916 | Kernel\_Read\|xilinx:adm-pcie-7v3:1ddr:3.0-0\|cnn\_GOOD | Read | OCL Region | 1 | 1 | 94227 | 94228 | 0.00333333 | 5273.076916 | 5273.076919 |
| 5273.076982 | Kernel\_Read\|xilinx:adm-pcie-7v3:1ddr:3.0-0\|cnn\_GOOD | Read | OCL Region | 1 | 1 | 94247 | 94248 | 0.00333333 | 5273.076982 | 5273.076986 |
| 5273.077049 | Kernel\_Read\|xilinx:adm-pcie-7v3:1ddr:3.0-0\|cnn\_GOOD | Read | OCL Region | 1 | 1 | 94267 | 94268 | 0.00333333 | 5273.077049 | 5273.077052 |
| 5273.077116 | Kernel\_Read\|xilinx:adm-pcie-7v3:1ddr:3.0-0\|cnn\_GOOD | Read | OCL Region | 1 | 1 | 94287 | 94288 | 0.00333333 | 5273.077116 | 5273.077119 |
| 5273.077182 | Kernel\_Read\|xilinx:adm-pcie-7v3:1ddr:3.0-0\|cnn\_GOOD | Read | OCL Region | 1 | 1 | 94307 | 94308 | 0.00333333 | 5273.077182 | 5273.077186 |
| 5273.077249 | Kernel\_Read\|xilinx:adm-pcie-7v3:1ddr:3.0-0\|cnn\_GOOD | Read | OCL Region | 1 | 1 | 94327 | 94328 | 0.00333333 | 5273.077249 | 5273.077252 |
| 5273.077316 | Kernel\_Read\|xilinx:adm-pcie-7v3:1ddr:3.0-0\|cnn\_GOOD | Read | OCL Region | 1 | 1 | 94347 | 94348 | 0.00333333 | 5273.077316 | 5273.077319 |
| 5273.077382 | Kernel\_Read\|xilinx:adm-pcie-7v3:1ddr:3.0-0\|cnn\_GOOD | Read | OCL Region | 1 | 1 | 94367 | 94368 | 0.00333333 | 5273.077382 | 5273.077386 |
| 5273.077449 | Kernel\_Read\|xilinx:adm-pcie-7v3:1ddr:3.0-0\|cnn\_GOOD | Read | OCL Region | 1 | 1 | 94387 | 94388 | 0.00333333 | 5273.077449 | 5273.077452 |
| 5273.077516 | Kernel\_Read\|xilinx:adm-pcie-7v3:1ddr:3.0-0\|cnn\_GOOD | Read | OCL Region | 1 | 1 | 94407 | 94408 | 0.00333333 | 5273.077516 | 5273.077519 |
| 5273.077599 | Kernel\_Read\|xilinx:adm-pcie-7v3:1ddr:3.0-0\|cnn\_GOOD | Read | OCL Region | 1 | 1 | 94432 | 94433 | 0.00333333 | 5273.077599 | 5273.077602 |
| 5273.077656 | Kernel\_Read\|xilinx:adm-pcie-7v3:1ddr:3.0-0\|cnn\_GOOD | Read | OCL Region | 1 | 1 | 94449 | 94450 | 0.00333333 | 5273.077656 | 5273.077659 |
| 5273.077716 | Kernel\_Read\|xilinx:adm-pcie-7v3:1ddr:3.0-0\|cnn\_GOOD | Read | OCL Region | 1 | 1 | 94467 | 94468 | 0.00333333 | 5273.077716 | 5273.077719 |
| 5273.077782 | Kernel\_Read\|xilinx:adm-pcie-7v3:1ddr:3.0-0\|cnn\_GOOD | Read | OCL Region | 1 | 1 | 94487 | 94488 | 0.00333333 | 5273.077782 | 5273.077786 |
| 5273.077849 | Kernel\_Read\|xilinx:adm-pcie-7v3:1ddr:3.0-0\|cnn\_GOOD | Read | OCL Region | 1 | 1 | 94507 | 94508 | 0.00333333 | 5273.077849 | 5273.077852 |
| 5273.077916 | Kernel\_Read\|xilinx:adm-pcie-7v3:1ddr:3.0-0\|cnn\_GOOD | Read | OCL Region | 1 | 1 | 94527 | 94528 | 0.00333333 | 5273.077916 | 5273.077919 |
| 5273.077982 | Kernel\_Read\|xilinx:adm-pcie-7v3:1ddr:3.0-0\|cnn\_GOOD | Read | OCL Region | 1 | 1 | 94547 | 94548 | 0.00333333 | 5273.077982 | 5273.077986 |
| 5273.078049 | Kernel\_Read\|xilinx:adm-pcie-7v3:1ddr:3.0-0\|cnn\_GOOD | Read | OCL Region | 1 | 1 | 94567 | 94568 | 0.00333333 | 5273.078049 | 5273.078052 |
| 5273.078116 | Kernel\_Read\|xilinx:adm-pcie-7v3:1ddr:3.0-0\|cnn\_GOOD | Read | OCL Region | 1 | 1 | 94587 | 94588 | 0.00333333 | 5273.078116 | 5273.078119 |
| 5273.078182 | Kernel\_Read\|xilinx:adm-pcie-7v3:1ddr:3.0-0\|cnn\_GOOD | Read | OCL Region | 1 | 1 | 94607 | 94608 | 0.00333333 | 5273.078182 | 5273.078186 |
| 5273.078249 | Kernel\_Read\|xilinx:adm-pcie-7v3:1ddr:3.0-0\|cnn\_GOOD | Read | OCL Region | 1 | 1 | 94627 | 94628 | 0.00333333 | 5273.078249 | 5273.078252 |
| 5273.078316 | Kernel\_Read\|xilinx:adm-pcie-7v3:1ddr:3.0-0\|cnn\_GOOD | Read | OCL Region | 1 | 1 | 94647 | 94648 | 0.00333333 | 5273.078316 | 5273.078319 |
| 5273.078389 | Kernel\_Read\|xilinx:adm-pcie-7v3:1ddr:3.0-0\|cnn\_GOOD | Read | OCL Region | 1 | 1 | 94669 | 94670 | 0.00333333 | 5273.078389 | 5273.078392 |
| 5273.078449 | Kernel\_Read\|xilinx:adm-pcie-7v3:1ddr:3.0-0\|cnn\_GOOD | Read | OCL Region | 1 | 1 | 94687 | 94688 | 0.00333333 | 5273.078449 | 5273.078452 |
| 5273.078516 | Kernel\_Read\|xilinx:adm-pcie-7v3:1ddr:3.0-0\|cnn\_GOOD | Read | OCL Region | 1 | 1 | 94707 | 94708 | 0.00333333 | 5273.078516 | 5273.078519 |
| 5273.078582 | Kernel\_Read\|xilinx:adm-pcie-7v3:1ddr:3.0-0\|cnn\_GOOD | Read | OCL Region | 1 | 1 | 94727 | 94728 | 0.00333333 | 5273.078582 | 5273.078586 |
| 5273.078649 | Kernel\_Read\|xilinx:adm-pcie-7v3:1ddr:3.0-0\|cnn\_GOOD | Read | OCL Region | 1 | 1 | 94747 | 94748 | 0.00333333 | 5273.078649 | 5273.078652 |
| 5273.078716 | Kernel\_Read\|xilinx:adm-pcie-7v3:1ddr:3.0-0\|cnn\_GOOD | Read | OCL Region | 1 | 1 | 94767 | 94768 | 0.00333333 | 5273.078716 | 5273.078719 |
| 5273.078782 | Kernel\_Read\|xilinx:adm-pcie-7v3:1ddr:3.0-0\|cnn\_GOOD | Read | OCL Region | 1 | 1 | 94787 | 94788 | 0.00333333 | 5273.078782 | 5273.078786 |
| 5273.078849 | Kernel\_Read\|xilinx:adm-pcie-7v3:1ddr:3.0-0\|cnn\_GOOD | Read | OCL Region | 1 | 1 | 94807 | 94808 | 0.00333333 | 5273.078849 | 5273.078852 |
| 5273.078916 | Kernel\_Read\|xilinx:adm-pcie-7v3:1ddr:3.0-0\|cnn\_GOOD | Read | OCL Region | 1 | 1 | 94827 | 94828 | 0.00333333 | 5273.078916 | 5273.078919 |
| 5273.078982 | Kernel\_Read\|xilinx:adm-pcie-7v3:1ddr:3.0-0\|cnn\_GOOD | Read | OCL Region | 1 | 1 | 94847 | 94848 | 0.00333333 | 5273.078982 | 5273.078986 |
| 5273.079049 | Kernel\_Read\|xilinx:adm-pcie-7v3:1ddr:3.0-0\|cnn\_GOOD | Read | OCL Region | 1 | 1 | 94867 | 94868 | 0.00333333 | 5273.079049 | 5273.079052 |
| 5273.079116 | Kernel\_Read\|xilinx:adm-pcie-7v3:1ddr:3.0-0\|cnn\_GOOD | Read | OCL Region | 1 | 1 | 94887 | 94888 | 0.00333333 | 5273.079116 | 5273.079119 |
| 5273.079182 | Kernel\_Read\|xilinx:adm-pcie-7v3:1ddr:3.0-0\|cnn\_GOOD | Read | OCL Region | 1 | 1 | 94907 | 94908 | 0.00333333 | 5273.079182 | 5273.079186 |
| 5273.079249 | Kernel\_Read\|xilinx:adm-pcie-7v3:1ddr:3.0-0\|cnn\_GOOD | Read | OCL Region | 1 | 1 | 94927 | 94928 | 0.00333333 | 5273.079249 | 5273.079252 |
| 5273.079316 | Kernel\_Read\|xilinx:adm-pcie-7v3:1ddr:3.0-0\|cnn\_GOOD | Read | OCL Region | 1 | 1 | 94947 | 94948 | 0.00333333 | 5273.079316 | 5273.079319 |
| 5273.079382 | Kernel\_Read\|xilinx:adm-pcie-7v3:1ddr:3.0-0\|cnn\_GOOD | Read | OCL Region | 1 | 1 | 94967 | 94968 | 0.00333333 | 5273.079382 | 5273.079386 |
| 5273.079449 | Kernel\_Read\|xilinx:adm-pcie-7v3:1ddr:3.0-0\|cnn\_GOOD | Read | OCL Region | 1 | 1 | 94987 | 94988 | 0.00333333 | 5273.079449 | 5273.079452 |
| 5273.079516 | Kernel\_Read\|xilinx:adm-pcie-7v3:1ddr:3.0-0\|cnn\_GOOD | Read | OCL Region | 1 | 1 | 95007 | 95008 | 0.00333333 | 5273.079516 | 5273.079519 |
| 5273.079582 | Kernel\_Read\|xilinx:adm-pcie-7v3:1ddr:3.0-0\|cnn\_GOOD | Read | OCL Region | 1 | 1 | 95027 | 95028 | 0.00333333 | 5273.079582 | 5273.079586 |
| 5273.079649 | Kernel\_Read\|xilinx:adm-pcie-7v3:1ddr:3.0-0\|cnn\_GOOD | Read | OCL Region | 1 | 1 | 95047 | 95048 | 0.00333333 | 5273.079649 | 5273.079652 |
| 5273.079716 | Kernel\_Read\|xilinx:adm-pcie-7v3:1ddr:3.0-0\|cnn\_GOOD | Read | OCL Region | 1 | 1 | 95067 | 95068 | 0.00333333 | 5273.079716 | 5273.079719 |
| 5273.079782 | Kernel\_Read\|xilinx:adm-pcie-7v3:1ddr:3.0-0\|cnn\_GOOD | Read | OCL Region | 1 | 1 | 95087 | 95088 | 0.00333333 | 5273.079782 | 5273.079786 |
| 5273.079849 | Kernel\_Read\|xilinx:adm-pcie-7v3:1ddr:3.0-0\|cnn\_GOOD | Read | OCL Region | 1 | 1 | 95107 | 95108 | 0.00333333 | 5273.079849 | 5273.079852 |
| 5273.079916 | Kernel\_Read\|xilinx:adm-pcie-7v3:1ddr:3.0-0\|cnn\_GOOD | Read | OCL Region | 1 | 1 | 95127 | 95128 | 0.00333333 | 5273.079916 | 5273.079919 |
| 5273.079982 | Kernel\_Read\|xilinx:adm-pcie-7v3:1ddr:3.0-0\|cnn\_GOOD | Read | OCL Region | 1 | 1 | 95147 | 95148 | 0.00333333 | 5273.079982 | 5273.079986 |
| 5273.080049 | Kernel\_Read\|xilinx:adm-pcie-7v3:1ddr:3.0-0\|cnn\_GOOD | Read | OCL Region | 1 | 1 | 95167 | 95168 | 0.00333333 | 5273.080049 | 5273.080052 |
| 5273.080116 | Kernel\_Read\|xilinx:adm-pcie-7v3:1ddr:3.0-0\|cnn\_GOOD | Read | OCL Region | 1 | 1 | 95187 | 95188 | 0.00333333 | 5273.080116 | 5273.080119 |
| 5273.080182 | Kernel\_Read\|xilinx:adm-pcie-7v3:1ddr:3.0-0\|cnn\_GOOD | Read | OCL Region | 1 | 1 | 95207 | 95208 | 0.00333333 | 5273.080182 | 5273.080186 |
| 5273.080249 | Kernel\_Read\|xilinx:adm-pcie-7v3:1ddr:3.0-0\|cnn\_GOOD | Read | OCL Region | 1 | 1 | 95227 | 95228 | 0.00333333 | 5273.080249 | 5273.080252 |
| 5273.080316 | Kernel\_Read\|xilinx:adm-pcie-7v3:1ddr:3.0-0\|cnn\_GOOD | Read | OCL Region | 1 | 1 | 95247 | 95248 | 0.00333333 | 5273.080316 | 5273.080319 |
| 5273.080592 | Kernel\_Read\|xilinx:adm-pcie-7v3:1ddr:3.0-0\|cnn\_GOOD | Read | OCL Region | 1 | 1 | 95330 | 95331 | 0.00333333 | 5273.080592 | 5273.080596 |
| 5273.080599 | Kernel\_Read\|xilinx:adm-pcie-7v3:1ddr:3.0-0\|cnn\_GOOD | Read | OCL Region | 1 | 1 | 95332 | 95333 | 0.00333333 | 5273.080599 | 5273.080602 |
| 5273.080606 | Kernel\_Read\|xilinx:adm-pcie-7v3:1ddr:3.0-0\|cnn\_GOOD | Read | OCL Region | 1 | 1 | 95334 | 95335 | 0.00333333 | 5273.080606 | 5273.080609 |
| 5273.080609 | Kernel\_Read\|xilinx:adm-pcie-7v3:1ddr:3.0-0\|cnn\_GOOD | Read | OCL Region | 1 | 1 | 95335 | 95336 | 0.00333333 | 5273.080609 | 5273.080612 |
| 5273.080666 | Kernel\_Read\|xilinx:adm-pcie-7v3:1ddr:3.0-0\|cnn\_GOOD | Read | OCL Region | 1 | 1 | 95352 | 95353 | 0.00333333 | 5273.080666 | 5273.080669 |
| 5273.080726 | Kernel\_Read\|xilinx:adm-pcie-7v3:1ddr:3.0-0\|cnn\_GOOD | Read | OCL Region | 1 | 1 | 95370 | 95371 | 0.00333333 | 5273.080726 | 5273.080729 |
| 5273.080782 | Kernel\_Read\|xilinx:adm-pcie-7v3:1ddr:3.0-0\|cnn\_GOOD | Read | OCL Region | 1 | 1 | 95387 | 95388 | 0.00333333 | 5273.080782 | 5273.080786 |
| 5273.080849 | Kernel\_Read\|xilinx:adm-pcie-7v3:1ddr:3.0-0\|cnn\_GOOD | Read | OCL Region | 1 | 1 | 95407 | 95408 | 0.00333333 | 5273.080849 | 5273.080852 |
| 5273.080916 | Kernel\_Read\|xilinx:adm-pcie-7v3:1ddr:3.0-0\|cnn\_GOOD | Read | OCL Region | 1 | 1 | 95427 | 95428 | 0.00333333 | 5273.080916 | 5273.080919 |
| 5273.080982 | Kernel\_Read\|xilinx:adm-pcie-7v3:1ddr:3.0-0\|cnn\_GOOD | Read | OCL Region | 1 | 1 | 95447 | 95448 | 0.00333333 | 5273.080982 | 5273.080986 |
| 5273.081049 | Kernel\_Read\|xilinx:adm-pcie-7v3:1ddr:3.0-0\|cnn\_GOOD | Read | OCL Region | 1 | 1 | 95467 | 95468 | 0.00333333 | 5273.081049 | 5273.081052 |
| 5273.081116 | Kernel\_Read\|xilinx:adm-pcie-7v3:1ddr:3.0-0\|cnn\_GOOD | Read | OCL Region | 1 | 1 | 95487 | 95488 | 0.00333333 | 5273.081116 | 5273.081119 |
| 5273.081182 | Kernel\_Read\|xilinx:adm-pcie-7v3:1ddr:3.0-0\|cnn\_GOOD | Read | OCL Region | 1 | 1 | 95507 | 95508 | 0.00333333 | 5273.081182 | 5273.081186 |
| 5273.081249 | Kernel\_Read\|xilinx:adm-pcie-7v3:1ddr:3.0-0\|cnn\_GOOD | Read | OCL Region | 1 | 1 | 95527 | 95528 | 0.00333333 | 5273.081249 | 5273.081252 |
| 5273.081322 | Kernel\_Read\|xilinx:adm-pcie-7v3:1ddr:3.0-0\|cnn\_GOOD | Read | OCL Region | 1 | 1 | 95549 | 95550 | 0.00333333 | 5273.081322 | 5273.081326 |
| 5273.081392 | Kernel\_Read\|xilinx:adm-pcie-7v3:1ddr:3.0-0\|cnn\_GOOD | Read | OCL Region | 1 | 1 | 95570 | 95571 | 0.00333333 | 5273.081392 | 5273.081396 |
| 5273.081449 | Kernel\_Read\|xilinx:adm-pcie-7v3:1ddr:3.0-0\|cnn\_GOOD | Read | OCL Region | 1 | 1 | 95587 | 95588 | 0.00333333 | 5273.081449 | 5273.081452 |
| 5273.081516 | Kernel\_Read\|xilinx:adm-pcie-7v3:1ddr:3.0-0\|cnn\_GOOD | Read | OCL Region | 1 | 1 | 95607 | 95608 | 0.00333333 | 5273.081516 | 5273.081519 |
| 5273.081582 | Kernel\_Read\|xilinx:adm-pcie-7v3:1ddr:3.0-0\|cnn\_GOOD | Read | OCL Region | 1 | 1 | 95627 | 95628 | 0.00333333 | 5273.081582 | 5273.081586 |
| 5273.081649 | Kernel\_Read\|xilinx:adm-pcie-7v3:1ddr:3.0-0\|cnn\_GOOD | Read | OCL Region | 1 | 1 | 95647 | 95648 | 0.00333333 | 5273.081649 | 5273.081652 |
| 5273.081722 | Kernel\_Read\|xilinx:adm-pcie-7v3:1ddr:3.0-0\|cnn\_GOOD | Read | OCL Region | 1 | 1 | 95669 | 95670 | 0.00333333 | 5273.081722 | 5273.081726 |
| 5273.081782 | Kernel\_Read\|xilinx:adm-pcie-7v3:1ddr:3.0-0\|cnn\_GOOD | Read | OCL Region | 1 | 1 | 95687 | 95688 | 0.00333333 | 5273.081782 | 5273.081786 |
| 5273.081849 | Kernel\_Read\|xilinx:adm-pcie-7v3:1ddr:3.0-0\|cnn\_GOOD | Read | OCL Region | 1 | 1 | 95707 | 95708 | 0.00333333 | 5273.081849 | 5273.081852 |
| 5273.081916 | Kernel\_Read\|xilinx:adm-pcie-7v3:1ddr:3.0-0\|cnn\_GOOD | Read | OCL Region | 1 | 1 | 95727 | 95728 | 0.00333333 | 5273.081916 | 5273.081919 |
| 5273.081982 | Kernel\_Read\|xilinx:adm-pcie-7v3:1ddr:3.0-0\|cnn\_GOOD | Read | OCL Region | 1 | 1 | 95747 | 95748 | 0.00333333 | 5273.081982 | 5273.081986 |
| 5273.082049 | Kernel\_Read\|xilinx:adm-pcie-7v3:1ddr:3.0-0\|cnn\_GOOD | Read | OCL Region | 1 | 1 | 95767 | 95768 | 0.00333333 | 5273.082049 | 5273.082052 |
| 5273.082122 | Kernel\_Read\|xilinx:adm-pcie-7v3:1ddr:3.0-0\|cnn\_GOOD | Read | OCL Region | 1 | 1 | 95789 | 95790 | 0.00333333 | 5273.082122 | 5273.082126 |
| 5273.082182 | Kernel\_Read\|xilinx:adm-pcie-7v3:1ddr:3.0-0\|cnn\_GOOD | Read | OCL Region | 1 | 1 | 95807 | 95808 | 0.00333333 | 5273.082182 | 5273.082186 |
| 5273.082249 | Kernel\_Read\|xilinx:adm-pcie-7v3:1ddr:3.0-0\|cnn\_GOOD | Read | OCL Region | 1 | 1 | 95827 | 95828 | 0.00333333 | 5273.082249 | 5273.082252 |
| 5273.082316 | Kernel\_Read\|xilinx:adm-pcie-7v3:1ddr:3.0-0\|cnn\_GOOD | Read | OCL Region | 1 | 1 | 95847 | 95848 | 0.00333333 | 5273.082316 | 5273.082319 |
| 5273.082382 | Kernel\_Read\|xilinx:adm-pcie-7v3:1ddr:3.0-0\|cnn\_GOOD | Read | OCL Region | 1 | 1 | 95867 | 95868 | 0.00333333 | 5273.082382 | 5273.082386 |
| 5273.082449 | Kernel\_Read\|xilinx:adm-pcie-7v3:1ddr:3.0-0\|cnn\_GOOD | Read | OCL Region | 1 | 1 | 95887 | 95888 | 0.00333333 | 5273.082449 | 5273.082452 |
| 5273.082516 | Kernel\_Read\|xilinx:adm-pcie-7v3:1ddr:3.0-0\|cnn\_GOOD | Read | OCL Region | 1 | 1 | 95907 | 95908 | 0.00333333 | 5273.082516 | 5273.082519 |
| 5273.082582 | Kernel\_Read\|xilinx:adm-pcie-7v3:1ddr:3.0-0\|cnn\_GOOD | Read | OCL Region | 1 | 1 | 95927 | 95928 | 0.00333333 | 5273.082582 | 5273.082586 |
| 5273.082649 | Kernel\_Read\|xilinx:adm-pcie-7v3:1ddr:3.0-0\|cnn\_GOOD | Read | OCL Region | 1 | 1 | 95947 | 95948 | 0.00333333 | 5273.082649 | 5273.082652 |
| 5273.082716 | Kernel\_Read\|xilinx:adm-pcie-7v3:1ddr:3.0-0\|cnn\_GOOD | Read | OCL Region | 1 | 1 | 95967 | 95968 | 0.00333333 | 5273.082716 | 5273.082719 |
| 5273.082782 | Kernel\_Read\|xilinx:adm-pcie-7v3:1ddr:3.0-0\|cnn\_GOOD | Read | OCL Region | 1 | 1 | 95987 | 95988 | 0.00333333 | 5273.082782 | 5273.082786 |
| 5273.082849 | Kernel\_Read\|xilinx:adm-pcie-7v3:1ddr:3.0-0\|cnn\_GOOD | Read | OCL Region | 1 | 1 | 96007 | 96008 | 0.00333333 | 5273.082849 | 5273.082852 |
| 5273.082922 | Kernel\_Read\|xilinx:adm-pcie-7v3:1ddr:3.0-0\|cnn\_GOOD | Read | OCL Region | 1 | 1 | 96029 | 96030 | 0.00333333 | 5273.082922 | 5273.082926 |
| 5273.082982 | Kernel\_Read\|xilinx:adm-pcie-7v3:1ddr:3.0-0\|cnn\_GOOD | Read | OCL Region | 1 | 1 | 96047 | 96048 | 0.00333333 | 5273.082982 | 5273.082986 |
| 5273.083049 | Kernel\_Read\|xilinx:adm-pcie-7v3:1ddr:3.0-0\|cnn\_GOOD | Read | OCL Region | 1 | 1 | 96067 | 96068 | 0.00333333 | 5273.083049 | 5273.083052 |
| 5273.083116 | Kernel\_Read\|xilinx:adm-pcie-7v3:1ddr:3.0-0\|cnn\_GOOD | Read | OCL Region | 1 | 1 | 96087 | 96088 | 0.00333333 | 5273.083116 | 5273.083119 |
| 5273.083182 | Kernel\_Read\|xilinx:adm-pcie-7v3:1ddr:3.0-0\|cnn\_GOOD | Read | OCL Region | 1 | 1 | 96107 | 96108 | 0.00333333 | 5273.083182 | 5273.083186 |
| 5273.083249 | Kernel\_Read\|xilinx:adm-pcie-7v3:1ddr:3.0-0\|cnn\_GOOD | Read | OCL Region | 1 | 1 | 96127 | 96128 | 0.00333333 | 5273.083249 | 5273.083252 |
| 5273.083316 | Kernel\_Read\|xilinx:adm-pcie-7v3:1ddr:3.0-0\|cnn\_GOOD | Read | OCL Region | 1 | 1 | 96147 | 96148 | 0.00333333 | 5273.083316 | 5273.083319 |
| 5273.083382 | Kernel\_Read\|xilinx:adm-pcie-7v3:1ddr:3.0-0\|cnn\_GOOD | Read | OCL Region | 1 | 1 | 96167 | 96168 | 0.00333333 | 5273.083382 | 5273.083386 |
| 5273.083449 | Kernel\_Read\|xilinx:adm-pcie-7v3:1ddr:3.0-0\|cnn\_GOOD | Read | OCL Region | 1 | 1 | 96187 | 96188 | 0.00333333 | 5273.083449 | 5273.083452 |
| 5273.083522 | Kernel\_Read\|xilinx:adm-pcie-7v3:1ddr:3.0-0\|cnn\_GOOD | Read | OCL Region | 1 | 1 | 96209 | 96210 | 0.00333333 | 5273.083522 | 5273.083526 |
| 5273.083582 | Kernel\_Read\|xilinx:adm-pcie-7v3:1ddr:3.0-0\|cnn\_GOOD | Read | OCL Region | 1 | 1 | 96227 | 96228 | 0.00333333 | 5273.083582 | 5273.083586 |
| 5273.083649 | Kernel\_Read\|xilinx:adm-pcie-7v3:1ddr:3.0-0\|cnn\_GOOD | Read | OCL Region | 1 | 1 | 96247 | 96248 | 0.00333333 | 5273.083649 | 5273.083652 |
| 5273.083722 | Kernel\_Read\|xilinx:adm-pcie-7v3:1ddr:3.0-0\|cnn\_GOOD | Read | OCL Region | 1 | 1 | 96269 | 96270 | 0.00333333 | 5273.083722 | 5273.083726 |
| 5273.083782 | Kernel\_Read\|xilinx:adm-pcie-7v3:1ddr:3.0-0\|cnn\_GOOD | Read | OCL Region | 1 | 1 | 96287 | 96288 | 0.00333333 | 5273.083782 | 5273.083786 |
| 5273.083849 | Kernel\_Read\|xilinx:adm-pcie-7v3:1ddr:3.0-0\|cnn\_GOOD | Read | OCL Region | 1 | 1 | 96307 | 96308 | 0.00333333 | 5273.083849 | 5273.083852 |
| 5273.083916 | Kernel\_Read\|xilinx:adm-pcie-7v3:1ddr:3.0-0\|cnn\_GOOD | Read | OCL Region | 1 | 1 | 96327 | 96328 | 0.00333333 | 5273.083916 | 5273.083919 |
| 5273.083982 | Kernel\_Read\|xilinx:adm-pcie-7v3:1ddr:3.0-0\|cnn\_GOOD | Read | OCL Region | 1 | 1 | 96347 | 96348 | 0.00333333 | 5273.083982 | 5273.083986 |
| 5273.084049 | Kernel\_Read\|xilinx:adm-pcie-7v3:1ddr:3.0-0\|cnn\_GOOD | Read | OCL Region | 1 | 1 | 96367 | 96368 | 0.00333333 | 5273.084049 | 5273.084052 |
| 5273.084116 | Kernel\_Read\|xilinx:adm-pcie-7v3:1ddr:3.0-0\|cnn\_GOOD | Read | OCL Region | 1 | 1 | 96387 | 96388 | 0.00333333 | 5273.084116 | 5273.084119 |
| 5273.084182 | Kernel\_Read\|xilinx:adm-pcie-7v3:1ddr:3.0-0\|cnn\_GOOD | Read | OCL Region | 1 | 1 | 96407 | 96408 | 0.00333333 | 5273.084182 | 5273.084186 |
| 5273.084249 | Kernel\_Read\|xilinx:adm-pcie-7v3:1ddr:3.0-0\|cnn\_GOOD | Read | OCL Region | 1 | 1 | 96427 | 96428 | 0.00333333 | 5273.084249 | 5273.084252 |
| 5273.084316 | Kernel\_Read\|xilinx:adm-pcie-7v3:1ddr:3.0-0\|cnn\_GOOD | Read | OCL Region | 1 | 1 | 96447 | 96448 | 0.00333333 | 5273.084316 | 5273.084319 |
| 5273.084382 | Kernel\_Read\|xilinx:adm-pcie-7v3:1ddr:3.0-0\|cnn\_GOOD | Read | OCL Region | 1 | 1 | 96467 | 96468 | 0.00333333 | 5273.084382 | 5273.084386 |
| 5273.084449 | Kernel\_Read\|xilinx:adm-pcie-7v3:1ddr:3.0-0\|cnn\_GOOD | Read | OCL Region | 1 | 1 | 96487 | 96488 | 0.00333333 | 5273.084449 | 5273.084452 |
| 5273.084522 | Kernel\_Read\|xilinx:adm-pcie-7v3:1ddr:3.0-0\|cnn\_GOOD | Read | OCL Region | 1 | 1 | 96509 | 96510 | 0.00333333 | 5273.084522 | 5273.084526 |
| 5273.084582 | Kernel\_Read\|xilinx:adm-pcie-7v3:1ddr:3.0-0\|cnn\_GOOD | Read | OCL Region | 1 | 1 | 96527 | 96528 | 0.00333333 | 5273.084582 | 5273.084586 |
| 5273.084659 | Kernel\_Read\|xilinx:adm-pcie-7v3:1ddr:3.0-0\|cnn\_GOOD | Read | OCL Region | 1 | 1 | 96550 | 96551 | 0.00333333 | 5273.084659 | 5273.084662 |
| 5273.084716 | Kernel\_Read\|xilinx:adm-pcie-7v3:1ddr:3.0-0\|cnn\_GOOD | Read | OCL Region | 1 | 1 | 96567 | 96568 | 0.00333333 | 5273.084716 | 5273.084719 |
| 5273.084782 | Kernel\_Read\|xilinx:adm-pcie-7v3:1ddr:3.0-0\|cnn\_GOOD | Read | OCL Region | 1 | 1 | 96587 | 96588 | 0.00333333 | 5273.084782 | 5273.084786 |
| 5273.084849 | Kernel\_Read\|xilinx:adm-pcie-7v3:1ddr:3.0-0\|cnn\_GOOD | Read | OCL Region | 1 | 1 | 96607 | 96608 | 0.00333333 | 5273.084849 | 5273.084852 |
| 5273.084922 | Kernel\_Read\|xilinx:adm-pcie-7v3:1ddr:3.0-0\|cnn\_GOOD | Read | OCL Region | 1 | 1 | 96629 | 96630 | 0.00333333 | 5273.084922 | 5273.084926 |
| 5273.084982 | Kernel\_Read\|xilinx:adm-pcie-7v3:1ddr:3.0-0\|cnn\_GOOD | Read | OCL Region | 1 | 1 | 96647 | 96648 | 0.00333333 | 5273.084982 | 5273.084986 |
| 5273.085049 | Kernel\_Read\|xilinx:adm-pcie-7v3:1ddr:3.0-0\|cnn\_GOOD | Read | OCL Region | 1 | 1 | 96667 | 96668 | 0.00333333 | 5273.085049 | 5273.085052 |
| 5273.085122 | Kernel\_Read\|xilinx:adm-pcie-7v3:1ddr:3.0-0\|cnn\_GOOD | Read | OCL Region | 1 | 1 | 96689 | 96690 | 0.00333333 | 5273.085122 | 5273.085126 |
| 5273.085182 | Kernel\_Read\|xilinx:adm-pcie-7v3:1ddr:3.0-0\|cnn\_GOOD | Read | OCL Region | 1 | 1 | 96707 | 96708 | 0.00333333 | 5273.085182 | 5273.085186 |
| 5273.085249 | Kernel\_Read\|xilinx:adm-pcie-7v3:1ddr:3.0-0\|cnn\_GOOD | Read | OCL Region | 1 | 1 | 96727 | 96728 | 0.00333333 | 5273.085249 | 5273.085252 |
| 5273.085322 | Kernel\_Read\|xilinx:adm-pcie-7v3:1ddr:3.0-0\|cnn\_GOOD | Read | OCL Region | 1 | 1 | 96749 | 96750 | 0.00333333 | 5273.085322 | 5273.085326 |
| 5273.085382 | Kernel\_Read\|xilinx:adm-pcie-7v3:1ddr:3.0-0\|cnn\_GOOD | Read | OCL Region | 1 | 1 | 96767 | 96768 | 0.00333333 | 5273.085382 | 5273.085386 |
| 5273.085449 | Kernel\_Read\|xilinx:adm-pcie-7v3:1ddr:3.0-0\|cnn\_GOOD | Read | OCL Region | 1 | 1 | 96787 | 96788 | 0.00333333 | 5273.085449 | 5273.085452 |
| 5273.085516 | Kernel\_Read\|xilinx:adm-pcie-7v3:1ddr:3.0-0\|cnn\_GOOD | Read | OCL Region | 1 | 1 | 96807 | 96808 | 0.00333333 | 5273.085516 | 5273.085519 |
| 5273.085582 | Kernel\_Read\|xilinx:adm-pcie-7v3:1ddr:3.0-0\|cnn\_GOOD | Read | OCL Region | 1 | 1 | 96827 | 96828 | 0.00333333 | 5273.085582 | 5273.085586 |
| 5273.085649 | Kernel\_Read\|xilinx:adm-pcie-7v3:1ddr:3.0-0\|cnn\_GOOD | Read | OCL Region | 1 | 1 | 96847 | 96848 | 0.00333333 | 5273.085649 | 5273.085652 |
| 5273.085716 | Kernel\_Read\|xilinx:adm-pcie-7v3:1ddr:3.0-0\|cnn\_GOOD | Read | OCL Region | 1 | 1 | 96867 | 96868 | 0.00333333 | 5273.085716 | 5273.085719 |
| 5273.085782 | Kernel\_Read\|xilinx:adm-pcie-7v3:1ddr:3.0-0\|cnn\_GOOD | Read | OCL Region | 1 | 1 | 96887 | 96888 | 0.00333333 | 5273.085782 | 5273.085786 |
| 5273.085849 | Kernel\_Read\|xilinx:adm-pcie-7v3:1ddr:3.0-0\|cnn\_GOOD | Read | OCL Region | 1 | 1 | 96907 | 96908 | 0.00333333 | 5273.085849 | 5273.085852 |
| 5273.085922 | Kernel\_Read\|xilinx:adm-pcie-7v3:1ddr:3.0-0\|cnn\_GOOD | Read | OCL Region | 1 | 1 | 96929 | 96930 | 0.00333333 | 5273.085922 | 5273.085926 |
| 5273.085982 | Kernel\_Read\|xilinx:adm-pcie-7v3:1ddr:3.0-0\|cnn\_GOOD | Read | OCL Region | 1 | 1 | 96947 | 96948 | 0.00333333 | 5273.085982 | 5273.085986 |
| 5273.086049 | Kernel\_Read\|xilinx:adm-pcie-7v3:1ddr:3.0-0\|cnn\_GOOD | Read | OCL Region | 1 | 1 | 96967 | 96968 | 0.00333333 | 5273.086049 | 5273.086052 |
| 5273.086122 | Kernel\_Read\|xilinx:adm-pcie-7v3:1ddr:3.0-0\|cnn\_GOOD | Read | OCL Region | 1 | 1 | 96989 | 96990 | 0.00333333 | 5273.086122 | 5273.086126 |
| 5273.086182 | Kernel\_Read\|xilinx:adm-pcie-7v3:1ddr:3.0-0\|cnn\_GOOD | Read | OCL Region | 1 | 1 | 97007 | 97008 | 0.00333333 | 5273.086182 | 5273.086186 |
| 5273.086249 | Kernel\_Read\|xilinx:adm-pcie-7v3:1ddr:3.0-0\|cnn\_GOOD | Read | OCL Region | 1 | 1 | 97027 | 97028 | 0.00333333 | 5273.086249 | 5273.086252 |
| 5273.086312 | Kernel\_Read\|xilinx:adm-pcie-7v3:1ddr:3.0-0\|cnn\_GOOD | Read | OCL Region | 1 | 1 | 97046 | 97047 | 0.00333333 | 5273.086312 | 5273.086316 |
| 5273.086382 | Kernel\_Read\|xilinx:adm-pcie-7v3:1ddr:3.0-0\|cnn\_GOOD | Read | OCL Region | 1 | 1 | 97067 | 97068 | 0.00333333 | 5273.086382 | 5273.086386 |
| 5273.086449 | Kernel\_Read\|xilinx:adm-pcie-7v3:1ddr:3.0-0\|cnn\_GOOD | Read | OCL Region | 1 | 1 | 97087 | 97088 | 0.00333333 | 5273.086449 | 5273.086452 |
| 5273.086522 | Kernel\_Read\|xilinx:adm-pcie-7v3:1ddr:3.0-0\|cnn\_GOOD | Read | OCL Region | 1 | 1 | 97109 | 97110 | 0.00333333 | 5273.086522 | 5273.086526 |
| 5273.086582 | Kernel\_Read\|xilinx:adm-pcie-7v3:1ddr:3.0-0\|cnn\_GOOD | Read | OCL Region | 1 | 1 | 97127 | 97128 | 0.00333333 | 5273.086582 | 5273.086586 |
| 5273.086859 | Kernel\_Read\|xilinx:adm-pcie-7v3:1ddr:3.0-0\|cnn\_GOOD | Read | OCL Region | 1 | 1 | 97210 | 97211 | 0.00333333 | 5273.086859 | 5273.086862 |
| 5273.086862 | Kernel\_Read\|xilinx:adm-pcie-7v3:1ddr:3.0-0\|cnn\_GOOD | Read | OCL Region | 1 | 1 | 97211 | 97212 | 0.00333333 | 5273.086862 | 5273.086866 |
| 5273.086866 | Kernel\_Read\|xilinx:adm-pcie-7v3:1ddr:3.0-0\|cnn\_GOOD | Read | OCL Region | 1 | 1 | 97212 | 97213 | 0.00333333 | 5273.086866 | 5273.086869 |
| 5273.086876 | Kernel\_Read\|xilinx:adm-pcie-7v3:1ddr:3.0-0\|cnn\_GOOD | Read | OCL Region | 1 | 1 | 97215 | 97216 | 0.00333333 | 5273.086876 | 5273.086879 |
| 5273.086932 | Kernel\_Read\|xilinx:adm-pcie-7v3:1ddr:3.0-0\|cnn\_GOOD | Read | OCL Region | 1 | 1 | 97232 | 97233 | 0.00333333 | 5273.086932 | 5273.086936 |
| 5273.086989 | Kernel\_Read\|xilinx:adm-pcie-7v3:1ddr:3.0-0\|cnn\_GOOD | Read | OCL Region | 1 | 1 | 97249 | 97250 | 0.00333333 | 5273.086989 | 5273.086992 |
| 5273.087049 | Kernel\_Read\|xilinx:adm-pcie-7v3:1ddr:3.0-0\|cnn\_GOOD | Read | OCL Region | 1 | 1 | 97267 | 97268 | 0.00333333 | 5273.087049 | 5273.087052 |
| 5273.087116 | Kernel\_Read\|xilinx:adm-pcie-7v3:1ddr:3.0-0\|cnn\_GOOD | Read | OCL Region | 1 | 1 | 97287 | 97288 | 0.00333333 | 5273.087116 | 5273.087119 |
| 5273.087182 | Kernel\_Read\|xilinx:adm-pcie-7v3:1ddr:3.0-0\|cnn\_GOOD | Read | OCL Region | 1 | 1 | 97307 | 97308 | 0.00333333 | 5273.087182 | 5273.087186 |
| 5273.087249 | Kernel\_Read\|xilinx:adm-pcie-7v3:1ddr:3.0-0\|cnn\_GOOD | Read | OCL Region | 1 | 1 | 97327 | 97328 | 0.00333333 | 5273.087249 | 5273.087252 |
| 5273.087316 | Kernel\_Read\|xilinx:adm-pcie-7v3:1ddr:3.0-0\|cnn\_GOOD | Read | OCL Region | 1 | 1 | 97347 | 97348 | 0.00333333 | 5273.087316 | 5273.087319 |
| 5273.087382 | Kernel\_Read\|xilinx:adm-pcie-7v3:1ddr:3.0-0\|cnn\_GOOD | Read | OCL Region | 1 | 1 | 97367 | 97368 | 0.00333333 | 5273.087382 | 5273.087386 |
| 5273.087449 | Kernel\_Read\|xilinx:adm-pcie-7v3:1ddr:3.0-0\|cnn\_GOOD | Read | OCL Region | 1 | 1 | 97387 | 97388 | 0.00333333 | 5273.087449 | 5273.087452 |
| 5273.087522 | Kernel\_Read\|xilinx:adm-pcie-7v3:1ddr:3.0-0\|cnn\_GOOD | Read | OCL Region | 1 | 1 | 97409 | 97410 | 0.00333333 | 5273.087522 | 5273.087526 |
| 5273.087582 | Kernel\_Read\|xilinx:adm-pcie-7v3:1ddr:3.0-0\|cnn\_GOOD | Read | OCL Region | 1 | 1 | 97427 | 97428 | 0.00333333 | 5273.087582 | 5273.087586 |
| 5273.087662 | Kernel\_Read\|xilinx:adm-pcie-7v3:1ddr:3.0-0\|cnn\_GOOD | Read | OCL Region | 1 | 1 | 97451 | 97452 | 0.00333333 | 5273.087662 | 5273.087666 |
| 5273.087722 | Kernel\_Read\|xilinx:adm-pcie-7v3:1ddr:3.0-0\|cnn\_GOOD | Read | OCL Region | 1 | 1 | 97469 | 97470 | 0.00333333 | 5273.087722 | 5273.087726 |
| 5273.087782 | Kernel\_Read\|xilinx:adm-pcie-7v3:1ddr:3.0-0\|cnn\_GOOD | Read | OCL Region | 1 | 1 | 97487 | 97488 | 0.00333333 | 5273.087782 | 5273.087786 |
| 5273.087849 | Kernel\_Read\|xilinx:adm-pcie-7v3:1ddr:3.0-0\|cnn\_GOOD | Read | OCL Region | 1 | 1 | 97507 | 97508 | 0.00333333 | 5273.087849 | 5273.087852 |
| 5273.087922 | Kernel\_Read\|xilinx:adm-pcie-7v3:1ddr:3.0-0\|cnn\_GOOD | Read | OCL Region | 1 | 1 | 97529 | 97530 | 0.00333333 | 5273.087922 | 5273.087926 |
| 5273.087982 | Kernel\_Read\|xilinx:adm-pcie-7v3:1ddr:3.0-0\|cnn\_GOOD | Read | OCL Region | 1 | 1 | 97547 | 97548 | 0.00333333 | 5273.087982 | 5273.087986 |
| 5273.088049 | Kernel\_Read\|xilinx:adm-pcie-7v3:1ddr:3.0-0\|cnn\_GOOD | Read | OCL Region | 1 | 1 | 97567 | 97568 | 0.00333333 | 5273.088049 | 5273.088052 |
| 5273.088122 | Kernel\_Read\|xilinx:adm-pcie-7v3:1ddr:3.0-0\|cnn\_GOOD | Read | OCL Region | 1 | 1 | 97589 | 97590 | 0.00333333 | 5273.088122 | 5273.088126 |
| 5273.088182 | Kernel\_Read\|xilinx:adm-pcie-7v3:1ddr:3.0-0\|cnn\_GOOD | Read | OCL Region | 1 | 1 | 97607 | 97608 | 0.00333333 | 5273.088182 | 5273.088186 |
| 5273.088249 | Kernel\_Read\|xilinx:adm-pcie-7v3:1ddr:3.0-0\|cnn\_GOOD | Read | OCL Region | 1 | 1 | 97627 | 97628 | 0.00333333 | 5273.088249 | 5273.088252 |
| 5273.088322 | Kernel\_Read\|xilinx:adm-pcie-7v3:1ddr:3.0-0\|cnn\_GOOD | Read | OCL Region | 1 | 1 | 97649 | 97650 | 0.00333333 | 5273.088322 | 5273.088326 |
| 5273.088382 | Kernel\_Read\|xilinx:adm-pcie-7v3:1ddr:3.0-0\|cnn\_GOOD | Read | OCL Region | 1 | 1 | 97667 | 97668 | 0.00333333 | 5273.088382 | 5273.088386 |
| 5273.088456 | Kernel\_Read\|xilinx:adm-pcie-7v3:1ddr:3.0-0\|cnn\_GOOD | Read | OCL Region | 1 | 1 | 97689 | 97690 | 0.00333333 | 5273.088456 | 5273.088459 |
| 5273.088522 | Kernel\_Read\|xilinx:adm-pcie-7v3:1ddr:3.0-0\|cnn\_GOOD | Read | OCL Region | 1 | 1 | 97709 | 97710 | 0.00333333 | 5273.088522 | 5273.088526 |
| 5273.088582 | Kernel\_Read\|xilinx:adm-pcie-7v3:1ddr:3.0-0\|cnn\_GOOD | Read | OCL Region | 1 | 1 | 97727 | 97728 | 0.00333333 | 5273.088582 | 5273.088586 |
| 5273.088649 | Kernel\_Read\|xilinx:adm-pcie-7v3:1ddr:3.0-0\|cnn\_GOOD | Read | OCL Region | 1 | 1 | 97747 | 97748 | 0.00333333 | 5273.088649 | 5273.088652 |
| 5273.088722 | Kernel\_Read\|xilinx:adm-pcie-7v3:1ddr:3.0-0\|cnn\_GOOD | Read | OCL Region | 1 | 1 | 97769 | 97770 | 0.00333333 | 5273.088722 | 5273.088726 |
| 5273.088782 | Kernel\_Read\|xilinx:adm-pcie-7v3:1ddr:3.0-0\|cnn\_GOOD | Read | OCL Region | 1 | 1 | 97787 | 97788 | 0.00333333 | 5273.088782 | 5273.088786 |
| 5273.088849 | Kernel\_Read\|xilinx:adm-pcie-7v3:1ddr:3.0-0\|cnn\_GOOD | Read | OCL Region | 1 | 1 | 97807 | 97808 | 0.00333333 | 5273.088849 | 5273.088852 |
| 5273.088916 | Kernel\_Read\|xilinx:adm-pcie-7v3:1ddr:3.0-0\|cnn\_GOOD | Read | OCL Region | 1 | 1 | 97827 | 97828 | 0.00333333 | 5273.088916 | 5273.088919 |
| 5273.088982 | Kernel\_Read\|xilinx:adm-pcie-7v3:1ddr:3.0-0\|cnn\_GOOD | Read | OCL Region | 1 | 1 | 97847 | 97848 | 0.00333333 | 5273.088982 | 5273.088986 |
| 5273.089049 | Kernel\_Read\|xilinx:adm-pcie-7v3:1ddr:3.0-0\|cnn\_GOOD | Read | OCL Region | 1 | 1 | 97867 | 97868 | 0.00333333 | 5273.089049 | 5273.089052 |
| 5273.089122 | Kernel\_Read\|xilinx:adm-pcie-7v3:1ddr:3.0-0\|cnn\_GOOD | Read | OCL Region | 1 | 1 | 97889 | 97890 | 0.00333333 | 5273.089122 | 5273.089126 |
| 5273.089182 | Kernel\_Read\|xilinx:adm-pcie-7v3:1ddr:3.0-0\|cnn\_GOOD | Read | OCL Region | 1 | 1 | 97907 | 97908 | 0.00333333 | 5273.089182 | 5273.089186 |
| 5273.089249 | Kernel\_Read\|xilinx:adm-pcie-7v3:1ddr:3.0-0\|cnn\_GOOD | Read | OCL Region | 1 | 1 | 97927 | 97928 | 0.00333333 | 5273.089249 | 5273.089252 |
| 5273.089322 | Kernel\_Read\|xilinx:adm-pcie-7v3:1ddr:3.0-0\|cnn\_GOOD | Read | OCL Region | 1 | 1 | 97949 | 97950 | 0.00333333 | 5273.089322 | 5273.089326 |
| 5273.089382 | Kernel\_Read\|xilinx:adm-pcie-7v3:1ddr:3.0-0\|cnn\_GOOD | Read | OCL Region | 1 | 1 | 97967 | 97968 | 0.00333333 | 5273.089382 | 5273.089386 |
| 5273.089449 | Kernel\_Read\|xilinx:adm-pcie-7v3:1ddr:3.0-0\|cnn\_GOOD | Read | OCL Region | 1 | 1 | 97987 | 97988 | 0.00333333 | 5273.089449 | 5273.089452 |
| 5273.089522 | Kernel\_Read\|xilinx:adm-pcie-7v3:1ddr:3.0-0\|cnn\_GOOD | Read | OCL Region | 1 | 1 | 98009 | 98010 | 0.00333333 | 5273.089522 | 5273.089526 |
| 5273.089582 | Kernel\_Read\|xilinx:adm-pcie-7v3:1ddr:3.0-0\|cnn\_GOOD | Read | OCL Region | 1 | 1 | 98027 | 98028 | 0.00333333 | 5273.089582 | 5273.089586 |
| 5273.089649 | Kernel\_Read\|xilinx:adm-pcie-7v3:1ddr:3.0-0\|cnn\_GOOD | Read | OCL Region | 1 | 1 | 98047 | 98048 | 0.00333333 | 5273.089649 | 5273.089652 |
| 5273.089722 | Kernel\_Read\|xilinx:adm-pcie-7v3:1ddr:3.0-0\|cnn\_GOOD | Read | OCL Region | 1 | 1 | 98069 | 98070 | 0.00333333 | 5273.089722 | 5273.089726 |
| 5273.089782 | Kernel\_Read\|xilinx:adm-pcie-7v3:1ddr:3.0-0\|cnn\_GOOD | Read | OCL Region | 1 | 1 | 98087 | 98088 | 0.00333333 | 5273.089782 | 5273.089786 |
| 5273.089849 | Kernel\_Read\|xilinx:adm-pcie-7v3:1ddr:3.0-0\|cnn\_GOOD | Read | OCL Region | 1 | 1 | 98107 | 98108 | 0.00333333 | 5273.089849 | 5273.089852 |
| 5273.089922 | Kernel\_Read\|xilinx:adm-pcie-7v3:1ddr:3.0-0\|cnn\_GOOD | Read | OCL Region | 1 | 1 | 98129 | 98130 | 0.00333333 | 5273.089922 | 5273.089926 |
| 5273.089982 | Kernel\_Read\|xilinx:adm-pcie-7v3:1ddr:3.0-0\|cnn\_GOOD | Read | OCL Region | 1 | 1 | 98147 | 98148 | 0.00333333 | 5273.089982 | 5273.089986 |
| 5273.090049 | Kernel\_Read\|xilinx:adm-pcie-7v3:1ddr:3.0-0\|cnn\_GOOD | Read | OCL Region | 1 | 1 | 98167 | 98168 | 0.00333333 | 5273.090049 | 5273.090052 |
| 5273.090112 | Kernel\_Read\|xilinx:adm-pcie-7v3:1ddr:3.0-0\|cnn\_GOOD | Read | OCL Region | 1 | 1 | 98186 | 98187 | 0.00333333 | 5273.090112 | 5273.090116 |
| 5273.090182 | Kernel\_Read\|xilinx:adm-pcie-7v3:1ddr:3.0-0\|cnn\_GOOD | Read | OCL Region | 1 | 1 | 98207 | 98208 | 0.00333333 | 5273.090182 | 5273.090186 |
| 5273.090249 | Kernel\_Read\|xilinx:adm-pcie-7v3:1ddr:3.0-0\|cnn\_GOOD | Read | OCL Region | 1 | 1 | 98227 | 98228 | 0.00333333 | 5273.090249 | 5273.090252 |
| 5273.090322 | Kernel\_Read\|xilinx:adm-pcie-7v3:1ddr:3.0-0\|cnn\_GOOD | Read | OCL Region | 1 | 1 | 98249 | 98250 | 0.00333333 | 5273.090322 | 5273.090326 |
| 5273.090382 | Kernel\_Read\|xilinx:adm-pcie-7v3:1ddr:3.0-0\|cnn\_GOOD | Read | OCL Region | 1 | 1 | 98267 | 98268 | 0.00333333 | 5273.090382 | 5273.090386 |
| 5273.090449 | Kernel\_Read\|xilinx:adm-pcie-7v3:1ddr:3.0-0\|cnn\_GOOD | Read | OCL Region | 1 | 1 | 98287 | 98288 | 0.00333333 | 5273.090449 | 5273.090452 |
| 5273.090516 | Kernel\_Read\|xilinx:adm-pcie-7v3:1ddr:3.0-0\|cnn\_GOOD | Read | OCL Region | 1 | 1 | 98307 | 98308 | 0.00333333 | 5273.090516 | 5273.090519 |
| 5273.090582 | Kernel\_Read\|xilinx:adm-pcie-7v3:1ddr:3.0-0\|cnn\_GOOD | Read | OCL Region | 1 | 1 | 98327 | 98328 | 0.00333333 | 5273.090582 | 5273.090586 |
| 5273.090649 | Kernel\_Read\|xilinx:adm-pcie-7v3:1ddr:3.0-0\|cnn\_GOOD | Read | OCL Region | 1 | 1 | 98347 | 98348 | 0.00333333 | 5273.090649 | 5273.090652 |
| 5273.090722 | Kernel\_Read\|xilinx:adm-pcie-7v3:1ddr:3.0-0\|cnn\_GOOD | Read | OCL Region | 1 | 1 | 98369 | 98370 | 0.00333333 | 5273.090722 | 5273.090726 |
| 5273.090782 | Kernel\_Read\|xilinx:adm-pcie-7v3:1ddr:3.0-0\|cnn\_GOOD | Read | OCL Region | 1 | 1 | 98387 | 98388 | 0.00333333 | 5273.090782 | 5273.090786 |
| 5273.090849 | Kernel\_Read\|xilinx:adm-pcie-7v3:1ddr:3.0-0\|cnn\_GOOD | Read | OCL Region | 1 | 1 | 98407 | 98408 | 0.00333333 | 5273.090849 | 5273.090852 |
| 5273.090929 | Kernel\_Read\|xilinx:adm-pcie-7v3:1ddr:3.0-0\|cnn\_GOOD | Read | OCL Region | 1 | 1 | 98431 | 98432 | 0.00333333 | 5273.090929 | 5273.090932 |
| 5273.090982 | Kernel\_Read\|xilinx:adm-pcie-7v3:1ddr:3.0-0\|cnn\_GOOD | Read | OCL Region | 1 | 1 | 98447 | 98448 | 0.00333333 | 5273.090982 | 5273.090986 |
| 5273.091049 | Kernel\_Read\|xilinx:adm-pcie-7v3:1ddr:3.0-0\|cnn\_GOOD | Read | OCL Region | 1 | 1 | 98467 | 98468 | 0.00333333 | 5273.091049 | 5273.091052 |
| 5273.091122 | Kernel\_Read\|xilinx:adm-pcie-7v3:1ddr:3.0-0\|cnn\_GOOD | Read | OCL Region | 1 | 1 | 98489 | 98490 | 0.00333333 | 5273.091122 | 5273.091126 |
| 5273.091182 | Kernel\_Read\|xilinx:adm-pcie-7v3:1ddr:3.0-0\|cnn\_GOOD | Read | OCL Region | 1 | 1 | 98507 | 98508 | 0.00333333 | 5273.091182 | 5273.091186 |
| 5273.091249 | Kernel\_Read\|xilinx:adm-pcie-7v3:1ddr:3.0-0\|cnn\_GOOD | Read | OCL Region | 1 | 1 | 98527 | 98528 | 0.00333333 | 5273.091249 | 5273.091252 |
| 5273.091322 | Kernel\_Read\|xilinx:adm-pcie-7v3:1ddr:3.0-0\|cnn\_GOOD | Read | OCL Region | 1 | 1 | 98549 | 98550 | 0.00333333 | 5273.091322 | 5273.091326 |
| 5273.091382 | Kernel\_Read\|xilinx:adm-pcie-7v3:1ddr:3.0-0\|cnn\_GOOD | Read | OCL Region | 1 | 1 | 98567 | 98568 | 0.00333333 | 5273.091382 | 5273.091386 |
| 5273.091449 | Kernel\_Read\|xilinx:adm-pcie-7v3:1ddr:3.0-0\|cnn\_GOOD | Read | OCL Region | 1 | 1 | 98587 | 98588 | 0.00333333 | 5273.091449 | 5273.091452 |
| 5273.091522 | Kernel\_Read\|xilinx:adm-pcie-7v3:1ddr:3.0-0\|cnn\_GOOD | Read | OCL Region | 1 | 1 | 98609 | 98610 | 0.00333333 | 5273.091522 | 5273.091526 |
| 5273.091582 | Kernel\_Read\|xilinx:adm-pcie-7v3:1ddr:3.0-0\|cnn\_GOOD | Read | OCL Region | 1 | 1 | 98627 | 98628 | 0.00333333 | 5273.091582 | 5273.091586 |
| 5273.091649 | Kernel\_Read\|xilinx:adm-pcie-7v3:1ddr:3.0-0\|cnn\_GOOD | Read | OCL Region | 1 | 1 | 98647 | 98648 | 0.00333333 | 5273.091649 | 5273.091652 |
| 5273.091722 | Kernel\_Read\|xilinx:adm-pcie-7v3:1ddr:3.0-0\|cnn\_GOOD | Read | OCL Region | 1 | 1 | 98669 | 98670 | 0.00333333 | 5273.091722 | 5273.091726 |
| 5273.091789 | Kernel\_Read\|xilinx:adm-pcie-7v3:1ddr:3.0-0\|cnn\_GOOD | Read | OCL Region | 1 | 1 | 98689 | 98690 | 0.00333333 | 5273.091789 | 5273.091792 |
| 5273.091849 | Kernel\_Read\|xilinx:adm-pcie-7v3:1ddr:3.0-0\|cnn\_GOOD | Read | OCL Region | 1 | 1 | 98707 | 98708 | 0.00333333 | 5273.091849 | 5273.091852 |
| 5273.091916 | Kernel\_Read\|xilinx:adm-pcie-7v3:1ddr:3.0-0\|cnn\_GOOD | Read | OCL Region | 1 | 1 | 98727 | 98728 | 0.00333333 | 5273.091916 | 5273.091919 |
| 5273.091982 | Kernel\_Read\|xilinx:adm-pcie-7v3:1ddr:3.0-0\|cnn\_GOOD | Read | OCL Region | 1 | 1 | 98747 | 98748 | 0.00333333 | 5273.091982 | 5273.091986 |
| 5273.092049 | Kernel\_Read\|xilinx:adm-pcie-7v3:1ddr:3.0-0\|cnn\_GOOD | Read | OCL Region | 1 | 1 | 98767 | 98768 | 0.00333333 | 5273.092049 | 5273.092052 |
| 5273.092116 | Kernel\_Read\|xilinx:adm-pcie-7v3:1ddr:3.0-0\|cnn\_GOOD | Read | OCL Region | 1 | 1 | 98787 | 98788 | 0.00333333 | 5273.092116 | 5273.092119 |
| 5273.092182 | Kernel\_Read\|xilinx:adm-pcie-7v3:1ddr:3.0-0\|cnn\_GOOD | Read | OCL Region | 1 | 1 | 98807 | 98808 | 0.00333333 | 5273.092182 | 5273.092186 |
| 5273.092249 | Kernel\_Read\|xilinx:adm-pcie-7v3:1ddr:3.0-0\|cnn\_GOOD | Read | OCL Region | 1 | 1 | 98827 | 98828 | 0.00333333 | 5273.092249 | 5273.092252 |
| 5273.092322 | Kernel\_Read\|xilinx:adm-pcie-7v3:1ddr:3.0-0\|cnn\_GOOD | Read | OCL Region | 1 | 1 | 98849 | 98850 | 0.00333333 | 5273.092322 | 5273.092326 |
| 5273.092382 | Kernel\_Read\|xilinx:adm-pcie-7v3:1ddr:3.0-0\|cnn\_GOOD | Read | OCL Region | 1 | 1 | 98867 | 98868 | 0.00333333 | 5273.092382 | 5273.092386 |
| 5273.092449 | Kernel\_Read\|xilinx:adm-pcie-7v3:1ddr:3.0-0\|cnn\_GOOD | Read | OCL Region | 1 | 1 | 98887 | 98888 | 0.00333333 | 5273.092449 | 5273.092452 |
| 5273.092522 | Kernel\_Read\|xilinx:adm-pcie-7v3:1ddr:3.0-0\|cnn\_GOOD | Read | OCL Region | 1 | 1 | 98909 | 98910 | 0.00333333 | 5273.092522 | 5273.092526 |
| 5273.092582 | Kernel\_Read\|xilinx:adm-pcie-7v3:1ddr:3.0-0\|cnn\_GOOD | Read | OCL Region | 1 | 1 | 98927 | 98928 | 0.00333333 | 5273.092582 | 5273.092586 |
| 5273.092649 | Kernel\_Read\|xilinx:adm-pcie-7v3:1ddr:3.0-0\|cnn\_GOOD | Read | OCL Region | 1 | 1 | 98947 | 98948 | 0.00333333 | 5273.092649 | 5273.092652 |
| 5273.092716 | Kernel\_Read\|xilinx:adm-pcie-7v3:1ddr:3.0-0\|cnn\_GOOD | Read | OCL Region | 1 | 1 | 98967 | 98968 | 0.00333333 | 5273.092716 | 5273.092719 |
| 5273.092782 | Kernel\_Read\|xilinx:adm-pcie-7v3:1ddr:3.0-0\|cnn\_GOOD | Read | OCL Region | 1 | 1 | 98987 | 98988 | 0.00333333 | 5273.092782 | 5273.092786 |
| 5273.092849 | Kernel\_Read\|xilinx:adm-pcie-7v3:1ddr:3.0-0\|cnn\_GOOD | Read | OCL Region | 1 | 1 | 99007 | 99008 | 0.00333333 | 5273.092849 | 5273.092852 |
| 5273.093126 | Kernel\_Read\|xilinx:adm-pcie-7v3:1ddr:3.0-0\|cnn\_GOOD | Read | OCL Region | 1 | 1 | 99090 | 99091 | 0.00333333 | 5273.093126 | 5273.093129 |
| 5273.093129 | Kernel\_Read\|xilinx:adm-pcie-7v3:1ddr:3.0-0\|cnn\_GOOD | Read | OCL Region | 1 | 1 | 99091 | 99092 | 0.00333333 | 5273.093129 | 5273.093132 |
| 5273.093132 | Kernel\_Read\|xilinx:adm-pcie-7v3:1ddr:3.0-0\|cnn\_GOOD | Read | OCL Region | 1 | 1 | 99092 | 99093 | 0.00333333 | 5273.093132 | 5273.093136 |
| 5273.093139 | Kernel\_Read\|xilinx:adm-pcie-7v3:1ddr:3.0-0\|cnn\_GOOD | Read | OCL Region | 1 | 1 | 99094 | 99095 | 0.00333333 | 5273.093139 | 5273.093142 |
| 5273.093199 | Kernel\_Read\|xilinx:adm-pcie-7v3:1ddr:3.0-0\|cnn\_GOOD | Read | OCL Region | 1 | 1 | 99112 | 99113 | 0.00333333 | 5273.093199 | 5273.093202 |
| 5273.093256 | Kernel\_Read\|xilinx:adm-pcie-7v3:1ddr:3.0-0\|cnn\_GOOD | Read | OCL Region | 1 | 1 | 99129 | 99130 | 0.00333333 | 5273.093256 | 5273.093259 |
| 5273.093322 | Kernel\_Read\|xilinx:adm-pcie-7v3:1ddr:3.0-0\|cnn\_GOOD | Read | OCL Region | 1 | 1 | 99149 | 99150 | 0.00333333 | 5273.093322 | 5273.093326 |
| 5273.093382 | Kernel\_Read\|xilinx:adm-pcie-7v3:1ddr:3.0-0\|cnn\_GOOD | Read | OCL Region | 1 | 1 | 99167 | 99168 | 0.00333333 | 5273.093382 | 5273.093386 |
| 5273.093449 | Kernel\_Read\|xilinx:adm-pcie-7v3:1ddr:3.0-0\|cnn\_GOOD | Read | OCL Region | 1 | 1 | 99187 | 99188 | 0.00333333 | 5273.093449 | 5273.093452 |
| 5273.093522 | Kernel\_Read\|xilinx:adm-pcie-7v3:1ddr:3.0-0\|cnn\_GOOD | Read | OCL Region | 1 | 1 | 99209 | 99210 | 0.00333333 | 5273.093522 | 5273.093526 |
| 5273.093582 | Kernel\_Read\|xilinx:adm-pcie-7v3:1ddr:3.0-0\|cnn\_GOOD | Read | OCL Region | 1 | 1 | 99227 | 99228 | 0.00333333 | 5273.093582 | 5273.093586 |
| 5273.093649 | Kernel\_Read\|xilinx:adm-pcie-7v3:1ddr:3.0-0\|cnn\_GOOD | Read | OCL Region | 1 | 1 | 99247 | 99248 | 0.00333333 | 5273.093649 | 5273.093652 |
| 5273.093716 | Kernel\_Read\|xilinx:adm-pcie-7v3:1ddr:3.0-0\|cnn\_GOOD | Read | OCL Region | 1 | 1 | 99267 | 99268 | 0.00333333 | 5273.093716 | 5273.093719 |
| 5273.093782 | Kernel\_Read\|xilinx:adm-pcie-7v3:1ddr:3.0-0\|cnn\_GOOD | Read | OCL Region | 1 | 1 | 99287 | 99288 | 0.00333333 | 5273.093782 | 5273.093786 |
| 5273.093849 | Kernel\_Read\|xilinx:adm-pcie-7v3:1ddr:3.0-0\|cnn\_GOOD | Read | OCL Region | 1 | 1 | 99307 | 99308 | 0.00333333 | 5273.093849 | 5273.093852 |
| 5273.093912 | Kernel\_Read\|xilinx:adm-pcie-7v3:1ddr:3.0-0\|cnn\_GOOD | Read | OCL Region | 1 | 1 | 99326 | 99327 | 0.00333333 | 5273.093912 | 5273.093916 |
| 5273.093982 | Kernel\_Read\|xilinx:adm-pcie-7v3:1ddr:3.0-0\|cnn\_GOOD | Read | OCL Region | 1 | 1 | 99347 | 99348 | 0.00333333 | 5273.093982 | 5273.093986 |
| 5273.094049 | Kernel\_Read\|xilinx:adm-pcie-7v3:1ddr:3.0-0\|cnn\_GOOD | Read | OCL Region | 1 | 1 | 99367 | 99368 | 0.00333333 | 5273.094049 | 5273.094052 |
| 5273.094122 | Kernel\_Read\|xilinx:adm-pcie-7v3:1ddr:3.0-0\|cnn\_GOOD | Read | OCL Region | 1 | 1 | 99389 | 99390 | 0.00333333 | 5273.094122 | 5273.094126 |
| 5273.094182 | Kernel\_Read\|xilinx:adm-pcie-7v3:1ddr:3.0-0\|cnn\_GOOD | Read | OCL Region | 1 | 1 | 99407 | 99408 | 0.00333333 | 5273.094182 | 5273.094186 |
| 5273.094249 | Kernel\_Read\|xilinx:adm-pcie-7v3:1ddr:3.0-0\|cnn\_GOOD | Read | OCL Region | 1 | 1 | 99427 | 99428 | 0.00333333 | 5273.094249 | 5273.094252 |
| 5273.094316 | Kernel\_Read\|xilinx:adm-pcie-7v3:1ddr:3.0-0\|cnn\_GOOD | Read | OCL Region | 1 | 1 | 99447 | 99448 | 0.00333333 | 5273.094316 | 5273.094319 |
| 5273.094382 | Kernel\_Read\|xilinx:adm-pcie-7v3:1ddr:3.0-0\|cnn\_GOOD | Read | OCL Region | 1 | 1 | 99467 | 99468 | 0.00333333 | 5273.094382 | 5273.094386 |
| 5273.094449 | Kernel\_Read\|xilinx:adm-pcie-7v3:1ddr:3.0-0\|cnn\_GOOD | Read | OCL Region | 1 | 1 | 99487 | 99488 | 0.00333333 | 5273.094449 | 5273.094452 |
| 5273.094522 | Kernel\_Read\|xilinx:adm-pcie-7v3:1ddr:3.0-0\|cnn\_GOOD | Read | OCL Region | 1 | 1 | 99509 | 99510 | 0.00333333 | 5273.094522 | 5273.094526 |
| 5273.094582 | Kernel\_Read\|xilinx:adm-pcie-7v3:1ddr:3.0-0\|cnn\_GOOD | Read | OCL Region | 1 | 1 | 99527 | 99528 | 0.00333333 | 5273.094582 | 5273.094586 |
| 5273.094649 | Kernel\_Read\|xilinx:adm-pcie-7v3:1ddr:3.0-0\|cnn\_GOOD | Read | OCL Region | 1 | 1 | 99547 | 99548 | 0.00333333 | 5273.094649 | 5273.094652 |
| 5273.094726 | Kernel\_Read\|xilinx:adm-pcie-7v3:1ddr:3.0-0\|cnn\_GOOD | Read | OCL Region | 1 | 1 | 99570 | 99571 | 0.00333333 | 5273.094726 | 5273.094729 |
| 5273.094782 | Kernel\_Read\|xilinx:adm-pcie-7v3:1ddr:3.0-0\|cnn\_GOOD | Read | OCL Region | 1 | 1 | 99587 | 99588 | 0.00333333 | 5273.094782 | 5273.094786 |
| 5273.094849 | Kernel\_Read\|xilinx:adm-pcie-7v3:1ddr:3.0-0\|cnn\_GOOD | Read | OCL Region | 1 | 1 | 99607 | 99608 | 0.00333333 | 5273.094849 | 5273.094852 |
| 5273.094922 | Kernel\_Read\|xilinx:adm-pcie-7v3:1ddr:3.0-0\|cnn\_GOOD | Read | OCL Region | 1 | 1 | 99629 | 99630 | 0.00333333 | 5273.094922 | 5273.094926 |
| 5273.094982 | Kernel\_Read\|xilinx:adm-pcie-7v3:1ddr:3.0-0\|cnn\_GOOD | Read | OCL Region | 1 | 1 | 99647 | 99648 | 0.00333333 | 5273.094982 | 5273.094986 |
| 5273.095049 | Kernel\_Read\|xilinx:adm-pcie-7v3:1ddr:3.0-0\|cnn\_GOOD | Read | OCL Region | 1 | 1 | 99667 | 99668 | 0.00333333 | 5273.095049 | 5273.095052 |
| 5273.095122 | Kernel\_Read\|xilinx:adm-pcie-7v3:1ddr:3.0-0\|cnn\_GOOD | Read | OCL Region | 1 | 1 | 99689 | 99690 | 0.00333333 | 5273.095122 | 5273.095126 |
| 5273.095182 | Kernel\_Read\|xilinx:adm-pcie-7v3:1ddr:3.0-0\|cnn\_GOOD | Read | OCL Region | 1 | 1 | 99707 | 99708 | 0.00333333 | 5273.095182 | 5273.095186 |
| 5273.095249 | Kernel\_Read\|xilinx:adm-pcie-7v3:1ddr:3.0-0\|cnn\_GOOD | Read | OCL Region | 1 | 1 | 99727 | 99728 | 0.00333333 | 5273.095249 | 5273.095252 |
| 5273.095316 | Kernel\_Read\|xilinx:adm-pcie-7v3:1ddr:3.0-0\|cnn\_GOOD | Read | OCL Region | 1 | 1 | 99747 | 99748 | 0.00333333 | 5273.095316 | 5273.095319 |
| 5273.095382 | Kernel\_Read\|xilinx:adm-pcie-7v3:1ddr:3.0-0\|cnn\_GOOD | Read | OCL Region | 1 | 1 | 99767 | 99768 | 0.00333333 | 5273.095382 | 5273.095386 |
| 5273.095449 | Kernel\_Read\|xilinx:adm-pcie-7v3:1ddr:3.0-0\|cnn\_GOOD | Read | OCL Region | 1 | 1 | 99787 | 99788 | 0.00333333 | 5273.095449 | 5273.095452 |
| 5273.095522 | Kernel\_Read\|xilinx:adm-pcie-7v3:1ddr:3.0-0\|cnn\_GOOD | Read | OCL Region | 1 | 1 | 99809 | 99810 | 0.00333333 | 5273.095522 | 5273.095526 |
| 5273.095582 | Kernel\_Read\|xilinx:adm-pcie-7v3:1ddr:3.0-0\|cnn\_GOOD | Read | OCL Region | 1 | 1 | 99827 | 99828 | 0.00333333 | 5273.095582 | 5273.095586 |
| 5273.095649 | Kernel\_Read\|xilinx:adm-pcie-7v3:1ddr:3.0-0\|cnn\_GOOD | Read | OCL Region | 1 | 1 | 99847 | 99848 | 0.00333333 | 5273.095649 | 5273.095652 |
| 5273.095722 | Kernel\_Read\|xilinx:adm-pcie-7v3:1ddr:3.0-0\|cnn\_GOOD | Read | OCL Region | 1 | 1 | 99869 | 99870 | 0.00333333 | 5273.095722 | 5273.095726 |
| 5273.095782 | Kernel\_Read\|xilinx:adm-pcie-7v3:1ddr:3.0-0\|cnn\_GOOD | Read | OCL Region | 1 | 1 | 99887 | 99888 | 0.00333333 | 5273.095782 | 5273.095786 |
| 5273.095849 | Kernel\_Read\|xilinx:adm-pcie-7v3:1ddr:3.0-0\|cnn\_GOOD | Read | OCL Region | 1 | 1 | 99907 | 99908 | 0.00333333 | 5273.095849 | 5273.095852 |
| 5273.095922 | Kernel\_Read\|xilinx:adm-pcie-7v3:1ddr:3.0-0\|cnn\_GOOD | Read | OCL Region | 1 | 1 | 99929 | 99930 | 0.00333333 | 5273.095922 | 5273.095926 |
| 5273.095982 | Kernel\_Read\|xilinx:adm-pcie-7v3:1ddr:3.0-0\|cnn\_GOOD | Read | OCL Region | 1 | 1 | 99947 | 99948 | 0.00333333 | 5273.095982 | 5273.095986 |
| 5273.096049 | Kernel\_Read\|xilinx:adm-pcie-7v3:1ddr:3.0-0\|cnn\_GOOD | Read | OCL Region | 1 | 1 | 99967 | 99968 | 0.00333333 | 5273.096049 | 5273.096052 |
| 5273.096122 | Kernel\_Read\|xilinx:adm-pcie-7v3:1ddr:3.0-0\|cnn\_GOOD | Read | OCL Region | 1 | 1 | 99989 | 99990 | 0.00333333 | 5273.096122 | 5273.096126 |
| 5273.096182 | Kernel\_Read\|xilinx:adm-pcie-7v3:1ddr:3.0-0\|cnn\_GOOD | Read | OCL Region | 1 | 1 | 100007 | 100008 | 0.00333333 | 5273.096182 | 5273.096186 |
| 5273.096249 | Kernel\_Read\|xilinx:adm-pcie-7v3:1ddr:3.0-0\|cnn\_GOOD | Read | OCL Region | 1 | 1 | 100027 | 100028 | 0.00333333 | 5273.096249 | 5273.096252 |
| 5273.096322 | Kernel\_Read\|xilinx:adm-pcie-7v3:1ddr:3.0-0\|cnn\_GOOD | Read | OCL Region | 1 | 1 | 100049 | 100050 | 0.00333333 | 5273.096322 | 5273.096326 |
| 5273.096382 | Kernel\_Read\|xilinx:adm-pcie-7v3:1ddr:3.0-0\|cnn\_GOOD | Read | OCL Region | 1 | 1 | 100067 | 100068 | 0.00333333 | 5273.096382 | 5273.096386 |
| 5273.096449 | Kernel\_Read\|xilinx:adm-pcie-7v3:1ddr:3.0-0\|cnn\_GOOD | Read | OCL Region | 1 | 1 | 100087 | 100088 | 0.00333333 | 5273.096449 | 5273.096452 |
| 5273.096522 | Kernel\_Read\|xilinx:adm-pcie-7v3:1ddr:3.0-0\|cnn\_GOOD | Read | OCL Region | 1 | 1 | 100109 | 100110 | 0.00333333 | 5273.096522 | 5273.096526 |
| 5273.096582 | Kernel\_Read\|xilinx:adm-pcie-7v3:1ddr:3.0-0\|cnn\_GOOD | Read | OCL Region | 1 | 1 | 100127 | 100128 | 0.00333333 | 5273.096582 | 5273.096586 |
| 5273.096649 | Kernel\_Read\|xilinx:adm-pcie-7v3:1ddr:3.0-0\|cnn\_GOOD | Read | OCL Region | 1 | 1 | 100147 | 100148 | 0.00333333 | 5273.096649 | 5273.096652 |
| 5273.096722 | Kernel\_Read\|xilinx:adm-pcie-7v3:1ddr:3.0-0\|cnn\_GOOD | Read | OCL Region | 1 | 1 | 100169 | 100170 | 0.00333333 | 5273.096722 | 5273.096726 |
| 5273.096782 | Kernel\_Read\|xilinx:adm-pcie-7v3:1ddr:3.0-0\|cnn\_GOOD | Read | OCL Region | 1 | 1 | 100187 | 100188 | 0.00333333 | 5273.096782 | 5273.096786 |
| 5273.096849 | Kernel\_Read\|xilinx:adm-pcie-7v3:1ddr:3.0-0\|cnn\_GOOD | Read | OCL Region | 1 | 1 | 100207 | 100208 | 0.00333333 | 5273.096849 | 5273.096852 |
| 5273.096922 | Kernel\_Read\|xilinx:adm-pcie-7v3:1ddr:3.0-0\|cnn\_GOOD | Read | OCL Region | 1 | 1 | 100229 | 100230 | 0.00333333 | 5273.096922 | 5273.096926 |
| 5273.096982 | Kernel\_Read\|xilinx:adm-pcie-7v3:1ddr:3.0-0\|cnn\_GOOD | Read | OCL Region | 1 | 1 | 100247 | 100248 | 0.00333333 | 5273.096982 | 5273.096986 |
| 5273.097049 | Kernel\_Read\|xilinx:adm-pcie-7v3:1ddr:3.0-0\|cnn\_GOOD | Read | OCL Region | 1 | 1 | 100267 | 100268 | 0.00333333 | 5273.097049 | 5273.097052 |
| 5273.097122 | Kernel\_Read\|xilinx:adm-pcie-7v3:1ddr:3.0-0\|cnn\_GOOD | Read | OCL Region | 1 | 1 | 100289 | 100290 | 0.00333333 | 5273.097122 | 5273.097126 |
| 5273.097182 | Kernel\_Read\|xilinx:adm-pcie-7v3:1ddr:3.0-0\|cnn\_GOOD | Read | OCL Region | 1 | 1 | 100307 | 100308 | 0.00333333 | 5273.097182 | 5273.097186 |
| 5273.097249 | Kernel\_Read\|xilinx:adm-pcie-7v3:1ddr:3.0-0\|cnn\_GOOD | Read | OCL Region | 1 | 1 | 100327 | 100328 | 0.00333333 | 5273.097249 | 5273.097252 |
| 5273.097322 | Kernel\_Read\|xilinx:adm-pcie-7v3:1ddr:3.0-0\|cnn\_GOOD | Read | OCL Region | 1 | 1 | 100349 | 100350 | 0.00333333 | 5273.097322 | 5273.097326 |
| 5273.097382 | Kernel\_Read\|xilinx:adm-pcie-7v3:1ddr:3.0-0\|cnn\_GOOD | Read | OCL Region | 1 | 1 | 100367 | 100368 | 0.00333333 | 5273.097382 | 5273.097386 |
| 5273.097449 | Kernel\_Read\|xilinx:adm-pcie-7v3:1ddr:3.0-0\|cnn\_GOOD | Read | OCL Region | 1 | 1 | 100387 | 100388 | 0.00333333 | 5273.097449 | 5273.097452 |
| 5273.097522 | Kernel\_Read\|xilinx:adm-pcie-7v3:1ddr:3.0-0\|cnn\_GOOD | Read | OCL Region | 1 | 1 | 100409 | 100410 | 0.00333333 | 5273.097522 | 5273.097526 |
| 5273.097582 | Kernel\_Read\|xilinx:adm-pcie-7v3:1ddr:3.0-0\|cnn\_GOOD | Read | OCL Region | 1 | 1 | 100427 | 100428 | 0.00333333 | 5273.097582 | 5273.097586 |
| 5273.097649 | Kernel\_Read\|xilinx:adm-pcie-7v3:1ddr:3.0-0\|cnn\_GOOD | Read | OCL Region | 1 | 1 | 100447 | 100448 | 0.00333333 | 5273.097649 | 5273.097652 |
| 5273.097722 | Kernel\_Read\|xilinx:adm-pcie-7v3:1ddr:3.0-0\|cnn\_GOOD | Read | OCL Region | 1 | 1 | 100469 | 100470 | 0.00333333 | 5273.097722 | 5273.097726 |
| 5273.097782 | Kernel\_Read\|xilinx:adm-pcie-7v3:1ddr:3.0-0\|cnn\_GOOD | Read | OCL Region | 1 | 1 | 100487 | 100488 | 0.00333333 | 5273.097782 | 5273.097786 |
| 5273.097849 | Kernel\_Read\|xilinx:adm-pcie-7v3:1ddr:3.0-0\|cnn\_GOOD | Read | OCL Region | 1 | 1 | 100507 | 100508 | 0.00333333 | 5273.097849 | 5273.097852 |
| 5273.097922 | Kernel\_Read\|xilinx:adm-pcie-7v3:1ddr:3.0-0\|cnn\_GOOD | Read | OCL Region | 1 | 1 | 100529 | 100530 | 0.00333333 | 5273.097922 | 5273.097926 |
| 5273.097982 | Kernel\_Read\|xilinx:adm-pcie-7v3:1ddr:3.0-0\|cnn\_GOOD | Read | OCL Region | 1 | 1 | 100547 | 100548 | 0.00333333 | 5273.097982 | 5273.097986 |
| 5273.098059 | Kernel\_Read\|xilinx:adm-pcie-7v3:1ddr:3.0-0\|cnn\_GOOD | Read | OCL Region | 1 | 1 | 100570 | 100571 | 0.00333333 | 5273.098059 | 5273.098062 |
| 5273.098122 | Kernel\_Read\|xilinx:adm-pcie-7v3:1ddr:3.0-0\|cnn\_GOOD | Read | OCL Region | 1 | 1 | 100589 | 100590 | 0.00333333 | 5273.098122 | 5273.098126 |
| 5273.098182 | Kernel\_Read\|xilinx:adm-pcie-7v3:1ddr:3.0-0\|cnn\_GOOD | Read | OCL Region | 1 | 1 | 100607 | 100608 | 0.00333333 | 5273.098182 | 5273.098186 |
| 5273.098249 | Kernel\_Read\|xilinx:adm-pcie-7v3:1ddr:3.0-0\|cnn\_GOOD | Read | OCL Region | 1 | 1 | 100627 | 100628 | 0.00333333 | 5273.098249 | 5273.098252 |
| 5273.098322 | Kernel\_Read\|xilinx:adm-pcie-7v3:1ddr:3.0-0\|cnn\_GOOD | Read | OCL Region | 1 | 1 | 100649 | 100650 | 0.00333333 | 5273.098322 | 5273.098326 |
| 5273.098382 | Kernel\_Read\|xilinx:adm-pcie-7v3:1ddr:3.0-0\|cnn\_GOOD | Read | OCL Region | 1 | 1 | 100667 | 100668 | 0.00333333 | 5273.098382 | 5273.098386 |
| 5273.098449 | Kernel\_Read\|xilinx:adm-pcie-7v3:1ddr:3.0-0\|cnn\_GOOD | Read | OCL Region | 1 | 1 | 100687 | 100688 | 0.00333333 | 5273.098449 | 5273.098452 |
| 5273.098522 | Kernel\_Read\|xilinx:adm-pcie-7v3:1ddr:3.0-0\|cnn\_GOOD | Read | OCL Region | 1 | 1 | 100709 | 100710 | 0.00333333 | 5273.098522 | 5273.098526 |
| 5273.098582 | Kernel\_Read\|xilinx:adm-pcie-7v3:1ddr:3.0-0\|cnn\_GOOD | Read | OCL Region | 1 | 1 | 100727 | 100728 | 0.00333333 | 5273.098582 | 5273.098586 |
| 5273.098649 | Kernel\_Read\|xilinx:adm-pcie-7v3:1ddr:3.0-0\|cnn\_GOOD | Read | OCL Region | 1 | 1 | 100747 | 100748 | 0.00333333 | 5273.098649 | 5273.098652 |
| 5273.098722 | Kernel\_Read\|xilinx:adm-pcie-7v3:1ddr:3.0-0\|cnn\_GOOD | Read | OCL Region | 1 | 1 | 100769 | 100770 | 0.00333333 | 5273.098722 | 5273.098726 |
| 5273.098782 | Kernel\_Read\|xilinx:adm-pcie-7v3:1ddr:3.0-0\|cnn\_GOOD | Read | OCL Region | 1 | 1 | 100787 | 100788 | 0.00333333 | 5273.098782 | 5273.098786 |
| 5273.098849 | Kernel\_Read\|xilinx:adm-pcie-7v3:1ddr:3.0-0\|cnn\_GOOD | Read | OCL Region | 1 | 1 | 100807 | 100808 | 0.00333333 | 5273.098849 | 5273.098852 |
| 5273.098922 | Kernel\_Read\|xilinx:adm-pcie-7v3:1ddr:3.0-0\|cnn\_GOOD | Read | OCL Region | 1 | 1 | 100829 | 100830 | 0.00333333 | 5273.098922 | 5273.098926 |
| 5273.098982 | Kernel\_Read\|xilinx:adm-pcie-7v3:1ddr:3.0-0\|cnn\_GOOD | Read | OCL Region | 1 | 1 | 100847 | 100848 | 0.00333333 | 5273.098982 | 5273.098986 |
| 5273.099049 | Kernel\_Read\|xilinx:adm-pcie-7v3:1ddr:3.0-0\|cnn\_GOOD | Read | OCL Region | 1 | 1 | 100867 | 100868 | 0.00333333 | 5273.099049 | 5273.099052 |
| 5273.099122 | Kernel\_Read\|xilinx:adm-pcie-7v3:1ddr:3.0-0\|cnn\_GOOD | Read | OCL Region | 1 | 1 | 100889 | 100890 | 0.00333333 | 5273.099122 | 5273.099126 |
| 5273.099396 | Kernel\_Read\|xilinx:adm-pcie-7v3:1ddr:3.0-0\|cnn\_GOOD | Read | OCL Region | 1 | 1 | 100971 | 100972 | 0.00333333 | 5273.099396 | 5273.099399 |
| 5273.099399 | Kernel\_Read\|xilinx:adm-pcie-7v3:1ddr:3.0-0\|cnn\_GOOD | Read | OCL Region | 1 | 1 | 100972 | 100973 | 0.00333333 | 5273.099399 | 5273.099402 |
| 5273.099406 | Kernel\_Read\|xilinx:adm-pcie-7v3:1ddr:3.0-0\|cnn\_GOOD | Read | OCL Region | 1 | 1 | 100974 | 100975 | 0.00333333 | 5273.099406 | 5273.099409 |
| 5273.099409 | Kernel\_Read\|xilinx:adm-pcie-7v3:1ddr:3.0-0\|cnn\_GOOD | Read | OCL Region | 1 | 1 | 100975 | 100976 | 0.00333333 | 5273.099409 | 5273.099412 |
| 5273.099472 | Kernel\_Read\|xilinx:adm-pcie-7v3:1ddr:3.0-0\|cnn\_GOOD | Read | OCL Region | 1 | 1 | 100994 | 100995 | 0.00333333 | 5273.099472 | 5273.099476 |
| 5273.099526 | Kernel\_Read\|xilinx:adm-pcie-7v3:1ddr:3.0-0\|cnn\_GOOD | Read | OCL Region | 1 | 1 | 101010 | 101011 | 0.00333333 | 5273.099526 | 5273.099529 |
| 5273.099582 | Kernel\_Read\|xilinx:adm-pcie-7v3:1ddr:3.0-0\|cnn\_GOOD | Read | OCL Region | 1 | 1 | 101027 | 101028 | 0.00333333 | 5273.099582 | 5273.099586 |
| 5273.099649 | Kernel\_Read\|xilinx:adm-pcie-7v3:1ddr:3.0-0\|cnn\_GOOD | Read | OCL Region | 1 | 1 | 101047 | 101048 | 0.00333333 | 5273.099649 | 5273.099652 |
| 5273.099722 | Kernel\_Read\|xilinx:adm-pcie-7v3:1ddr:3.0-0\|cnn\_GOOD | Read | OCL Region | 1 | 1 | 101069 | 101070 | 0.00333333 | 5273.099722 | 5273.099726 |
| 5273.099782 | Kernel\_Read\|xilinx:adm-pcie-7v3:1ddr:3.0-0\|cnn\_GOOD | Read | OCL Region | 1 | 1 | 101087 | 101088 | 0.00333333 | 5273.099782 | 5273.099786 |
| 5273.099849 | Kernel\_Read\|xilinx:adm-pcie-7v3:1ddr:3.0-0\|cnn\_GOOD | Read | OCL Region | 1 | 1 | 101107 | 101108 | 0.00333333 | 5273.099849 | 5273.099852 |
| 5273.099922 | Kernel\_Read\|xilinx:adm-pcie-7v3:1ddr:3.0-0\|cnn\_GOOD | Read | OCL Region | 1 | 1 | 101129 | 101130 | 0.00333333 | 5273.099922 | 5273.099926 |
| 5273.099982 | Kernel\_Read\|xilinx:adm-pcie-7v3:1ddr:3.0-0\|cnn\_GOOD | Read | OCL Region | 1 | 1 | 101147 | 101148 | 0.00333333 | 5273.099982 | 5273.099986 |
| 5273.100049 | Kernel\_Read\|xilinx:adm-pcie-7v3:1ddr:3.0-0\|cnn\_GOOD | Read | OCL Region | 1 | 1 | 101167 | 101168 | 0.00333333 | 5273.100049 | 5273.100052 |
| 5273.100122 | Kernel\_Read\|xilinx:adm-pcie-7v3:1ddr:3.0-0\|cnn\_GOOD | Read | OCL Region | 1 | 1 | 101189 | 101190 | 0.00333333 | 5273.100122 | 5273.100126 |
| 5273.100182 | Kernel\_Read\|xilinx:adm-pcie-7v3:1ddr:3.0-0\|cnn\_GOOD | Read | OCL Region | 1 | 1 | 101207 | 101208 | 0.00333333 | 5273.100182 | 5273.100186 |
| 5273.100249 | Kernel\_Read\|xilinx:adm-pcie-7v3:1ddr:3.0-0\|cnn\_GOOD | Read | OCL Region | 1 | 1 | 101227 | 101228 | 0.00333333 | 5273.100249 | 5273.100252 |
| 5273.100322 | Kernel\_Read\|xilinx:adm-pcie-7v3:1ddr:3.0-0\|cnn\_GOOD | Read | OCL Region | 1 | 1 | 101249 | 101250 | 0.00333333 | 5273.100322 | 5273.100326 |
| 5273.100382 | Kernel\_Read\|xilinx:adm-pcie-7v3:1ddr:3.0-0\|cnn\_GOOD | Read | OCL Region | 1 | 1 | 101267 | 101268 | 0.00333333 | 5273.100382 | 5273.100386 |
| 5273.100449 | Kernel\_Read\|xilinx:adm-pcie-7v3:1ddr:3.0-0\|cnn\_GOOD | Read | OCL Region | 1 | 1 | 101287 | 101288 | 0.00333333 | 5273.100449 | 5273.100452 |
| 5273.100522 | Kernel\_Read\|xilinx:adm-pcie-7v3:1ddr:3.0-0\|cnn\_GOOD | Read | OCL Region | 1 | 1 | 101309 | 101310 | 0.00333333 | 5273.100522 | 5273.100526 |
| 5273.100582 | Kernel\_Read\|xilinx:adm-pcie-7v3:1ddr:3.0-0\|cnn\_GOOD | Read | OCL Region | 1 | 1 | 101327 | 101328 | 0.00333333 | 5273.100582 | 5273.100586 |
| 5273.100649 | Kernel\_Read\|xilinx:adm-pcie-7v3:1ddr:3.0-0\|cnn\_GOOD | Read | OCL Region | 1 | 1 | 101347 | 101348 | 0.00333333 | 5273.100649 | 5273.100652 |
| 5273.100722 | Kernel\_Read\|xilinx:adm-pcie-7v3:1ddr:3.0-0\|cnn\_GOOD | Read | OCL Region | 1 | 1 | 101369 | 101370 | 0.00333333 | 5273.100722 | 5273.100726 |
| 5273.100782 | Kernel\_Read\|xilinx:adm-pcie-7v3:1ddr:3.0-0\|cnn\_GOOD | Read | OCL Region | 1 | 1 | 101387 | 101388 | 0.00333333 | 5273.100782 | 5273.100786 |
| 5273.100849 | Kernel\_Read\|xilinx:adm-pcie-7v3:1ddr:3.0-0\|cnn\_GOOD | Read | OCL Region | 1 | 1 | 101407 | 101408 | 0.00333333 | 5273.100849 | 5273.100852 |
| 5273.100922 | Kernel\_Read\|xilinx:adm-pcie-7v3:1ddr:3.0-0\|cnn\_GOOD | Read | OCL Region | 1 | 1 | 101429 | 101430 | 0.00333333 | 5273.100922 | 5273.100926 |
| 5273.100996 | Kernel\_Read\|xilinx:adm-pcie-7v3:1ddr:3.0-0\|cnn\_GOOD | Read | OCL Region | 1 | 1 | 101451 | 101452 | 0.00333333 | 5273.100996 | 5273.100999 |
| 5273.101049 | Kernel\_Read\|xilinx:adm-pcie-7v3:1ddr:3.0-0\|cnn\_GOOD | Read | OCL Region | 1 | 1 | 101467 | 101468 | 0.00333333 | 5273.101049 | 5273.101052 |
| 5273.101122 | Kernel\_Read\|xilinx:adm-pcie-7v3:1ddr:3.0-0\|cnn\_GOOD | Read | OCL Region | 1 | 1 | 101489 | 101490 | 0.00333333 | 5273.101122 | 5273.101126 |
| 5273.101182 | Kernel\_Read\|xilinx:adm-pcie-7v3:1ddr:3.0-0\|cnn\_GOOD | Read | OCL Region | 1 | 1 | 101507 | 101508 | 0.00333333 | 5273.101182 | 5273.101186 |
| 5273.101249 | Kernel\_Read\|xilinx:adm-pcie-7v3:1ddr:3.0-0\|cnn\_GOOD | Read | OCL Region | 1 | 1 | 101527 | 101528 | 0.00333333 | 5273.101249 | 5273.101252 |
| 5273.101322 | Kernel\_Read\|xilinx:adm-pcie-7v3:1ddr:3.0-0\|cnn\_GOOD | Read | OCL Region | 1 | 1 | 101549 | 101550 | 0.00333333 | 5273.101322 | 5273.101326 |
| 5273.101382 | Kernel\_Read\|xilinx:adm-pcie-7v3:1ddr:3.0-0\|cnn\_GOOD | Read | OCL Region | 1 | 1 | 101567 | 101568 | 0.00333333 | 5273.101382 | 5273.101386 |
| 5273.101449 | Kernel\_Read\|xilinx:adm-pcie-7v3:1ddr:3.0-0\|cnn\_GOOD | Read | OCL Region | 1 | 1 | 101587 | 101588 | 0.00333333 | 5273.101449 | 5273.101452 |
| 5273.101522 | Kernel\_Read\|xilinx:adm-pcie-7v3:1ddr:3.0-0\|cnn\_GOOD | Read | OCL Region | 1 | 1 | 101609 | 101610 | 0.00333333 | 5273.101522 | 5273.101526 |
| 5273.101582 | Kernel\_Read\|xilinx:adm-pcie-7v3:1ddr:3.0-0\|cnn\_GOOD | Read | OCL Region | 1 | 1 | 101627 | 101628 | 0.00333333 | 5273.101582 | 5273.101586 |
| 5273.101649 | Kernel\_Read\|xilinx:adm-pcie-7v3:1ddr:3.0-0\|cnn\_GOOD | Read | OCL Region | 1 | 1 | 101647 | 101648 | 0.00333333 | 5273.101649 | 5273.101652 |
| 5273.101716 | Kernel\_Read\|xilinx:adm-pcie-7v3:1ddr:3.0-0\|cnn\_GOOD | Read | OCL Region | 1 | 1 | 101667 | 101668 | 0.00333333 | 5273.101716 | 5273.101719 |
| 5273.101782 | Kernel\_Read\|xilinx:adm-pcie-7v3:1ddr:3.0-0\|cnn\_GOOD | Read | OCL Region | 1 | 1 | 101687 | 101688 | 0.00333333 | 5273.101782 | 5273.101786 |
| 5273.101856 | Kernel\_Read\|xilinx:adm-pcie-7v3:1ddr:3.0-0\|cnn\_GOOD | Read | OCL Region | 1 | 1 | 101709 | 101710 | 0.00333333 | 5273.101856 | 5273.101859 |
| 5273.101922 | Kernel\_Read\|xilinx:adm-pcie-7v3:1ddr:3.0-0\|cnn\_GOOD | Read | OCL Region | 1 | 1 | 101729 | 101730 | 0.00333333 | 5273.101922 | 5273.101926 |
| 5273.101982 | Kernel\_Read\|xilinx:adm-pcie-7v3:1ddr:3.0-0\|cnn\_GOOD | Read | OCL Region | 1 | 1 | 101747 | 101748 | 0.00333333 | 5273.101982 | 5273.101986 |
| 5273.102049 | Kernel\_Read\|xilinx:adm-pcie-7v3:1ddr:3.0-0\|cnn\_GOOD | Read | OCL Region | 1 | 1 | 101767 | 101768 | 0.00333333 | 5273.102049 | 5273.102052 |
| 5273.102122 | Kernel\_Read\|xilinx:adm-pcie-7v3:1ddr:3.0-0\|cnn\_GOOD | Read | OCL Region | 1 | 1 | 101789 | 101790 | 0.00333333 | 5273.102122 | 5273.102126 |
| 5273.102182 | Kernel\_Read\|xilinx:adm-pcie-7v3:1ddr:3.0-0\|cnn\_GOOD | Read | OCL Region | 1 | 1 | 101807 | 101808 | 0.00333333 | 5273.102182 | 5273.102186 |
| 5273.102249 | Kernel\_Read\|xilinx:adm-pcie-7v3:1ddr:3.0-0\|cnn\_GOOD | Read | OCL Region | 1 | 1 | 101827 | 101828 | 0.00333333 | 5273.102249 | 5273.102252 |
| 5273.102322 | Kernel\_Read\|xilinx:adm-pcie-7v3:1ddr:3.0-0\|cnn\_GOOD | Read | OCL Region | 1 | 1 | 101849 | 101850 | 0.00333333 | 5273.102322 | 5273.102326 |
| 5273.102382 | Kernel\_Read\|xilinx:adm-pcie-7v3:1ddr:3.0-0\|cnn\_GOOD | Read | OCL Region | 1 | 1 | 101867 | 101868 | 0.00333333 | 5273.102382 | 5273.102386 |
| 5273.102449 | Kernel\_Read\|xilinx:adm-pcie-7v3:1ddr:3.0-0\|cnn\_GOOD | Read | OCL Region | 1 | 1 | 101887 | 101888 | 0.00333333 | 5273.102449 | 5273.102452 |
| 5273.102522 | Kernel\_Read\|xilinx:adm-pcie-7v3:1ddr:3.0-0\|cnn\_GOOD | Read | OCL Region | 1 | 1 | 101909 | 101910 | 0.00333333 | 5273.102522 | 5273.102526 |
| 5273.102582 | Kernel\_Read\|xilinx:adm-pcie-7v3:1ddr:3.0-0\|cnn\_GOOD | Read | OCL Region | 1 | 1 | 101927 | 101928 | 0.00333333 | 5273.102582 | 5273.102586 |
| 5273.102649 | Kernel\_Read\|xilinx:adm-pcie-7v3:1ddr:3.0-0\|cnn\_GOOD | Read | OCL Region | 1 | 1 | 101947 | 101948 | 0.00333333 | 5273.102649 | 5273.102652 |
| 5273.102722 | Kernel\_Read\|xilinx:adm-pcie-7v3:1ddr:3.0-0\|cnn\_GOOD | Read | OCL Region | 1 | 1 | 101969 | 101970 | 0.00333333 | 5273.102722 | 5273.102726 |
| 5273.102782 | Kernel\_Read\|xilinx:adm-pcie-7v3:1ddr:3.0-0\|cnn\_GOOD | Read | OCL Region | 1 | 1 | 101987 | 101988 | 0.00333333 | 5273.102782 | 5273.102786 |
| 5273.102849 | Kernel\_Read\|xilinx:adm-pcie-7v3:1ddr:3.0-0\|cnn\_GOOD | Read | OCL Region | 1 | 1 | 102007 | 102008 | 0.00333333 | 5273.102849 | 5273.102852 |
| 5273.102922 | Kernel\_Read\|xilinx:adm-pcie-7v3:1ddr:3.0-0\|cnn\_GOOD | Read | OCL Region | 1 | 1 | 102029 | 102030 | 0.00333333 | 5273.102922 | 5273.102926 |
| 5273.102982 | Kernel\_Read\|xilinx:adm-pcie-7v3:1ddr:3.0-0\|cnn\_GOOD | Read | OCL Region | 1 | 1 | 102047 | 102048 | 0.00333333 | 5273.102982 | 5273.102986 |
| 5273.103049 | Kernel\_Read\|xilinx:adm-pcie-7v3:1ddr:3.0-0\|cnn\_GOOD | Read | OCL Region | 1 | 1 | 102067 | 102068 | 0.00333333 | 5273.103049 | 5273.103052 |
| 5273.103122 | Kernel\_Read\|xilinx:adm-pcie-7v3:1ddr:3.0-0\|cnn\_GOOD | Read | OCL Region | 1 | 1 | 102089 | 102090 | 0.00333333 | 5273.103122 | 5273.103126 |
| 5273.103182 | Kernel\_Read\|xilinx:adm-pcie-7v3:1ddr:3.0-0\|cnn\_GOOD | Read | OCL Region | 1 | 1 | 102107 | 102108 | 0.00333333 | 5273.103182 | 5273.103186 |
| 5273.103249 | Kernel\_Read\|xilinx:adm-pcie-7v3:1ddr:3.0-0\|cnn\_GOOD | Read | OCL Region | 1 | 1 | 102127 | 102128 | 0.00333333 | 5273.103249 | 5273.103252 |
| 5273.103322 | Kernel\_Read\|xilinx:adm-pcie-7v3:1ddr:3.0-0\|cnn\_GOOD | Read | OCL Region | 1 | 1 | 102149 | 102150 | 0.00333333 | 5273.103322 | 5273.103326 |
| 5273.103382 | Kernel\_Read\|xilinx:adm-pcie-7v3:1ddr:3.0-0\|cnn\_GOOD | Read | OCL Region | 1 | 1 | 102167 | 102168 | 0.00333333 | 5273.103382 | 5273.103386 |
| 5273.103449 | Kernel\_Read\|xilinx:adm-pcie-7v3:1ddr:3.0-0\|cnn\_GOOD | Read | OCL Region | 1 | 1 | 102187 | 102188 | 0.00333333 | 5273.103449 | 5273.103452 |
| 5273.103522 | Kernel\_Read\|xilinx:adm-pcie-7v3:1ddr:3.0-0\|cnn\_GOOD | Read | OCL Region | 1 | 1 | 102209 | 102210 | 0.00333333 | 5273.103522 | 5273.103526 |
| 5273.103582 | Kernel\_Read\|xilinx:adm-pcie-7v3:1ddr:3.0-0\|cnn\_GOOD | Read | OCL Region | 1 | 1 | 102227 | 102228 | 0.00333333 | 5273.103582 | 5273.103586 |
| 5273.103649 | Kernel\_Read\|xilinx:adm-pcie-7v3:1ddr:3.0-0\|cnn\_GOOD | Read | OCL Region | 1 | 1 | 102247 | 102248 | 0.00333333 | 5273.103649 | 5273.103652 |
| 5273.103722 | Kernel\_Read\|xilinx:adm-pcie-7v3:1ddr:3.0-0\|cnn\_GOOD | Read | OCL Region | 1 | 1 | 102269 | 102270 | 0.00333333 | 5273.103722 | 5273.103726 |
| 5273.103782 | Kernel\_Read\|xilinx:adm-pcie-7v3:1ddr:3.0-0\|cnn\_GOOD | Read | OCL Region | 1 | 1 | 102287 | 102288 | 0.00333333 | 5273.103782 | 5273.103786 |
| 5273.103849 | Kernel\_Read\|xilinx:adm-pcie-7v3:1ddr:3.0-0\|cnn\_GOOD | Read | OCL Region | 1 | 1 | 102307 | 102308 | 0.00333333 | 5273.103849 | 5273.103852 |
| 5273.103922 | Kernel\_Read\|xilinx:adm-pcie-7v3:1ddr:3.0-0\|cnn\_GOOD | Read | OCL Region | 1 | 1 | 102329 | 102330 | 0.00333333 | 5273.103922 | 5273.103926 |
| 5273.103982 | Kernel\_Read\|xilinx:adm-pcie-7v3:1ddr:3.0-0\|cnn\_GOOD | Read | OCL Region | 1 | 1 | 102347 | 102348 | 0.00333333 | 5273.103982 | 5273.103986 |
| 5273.104049 | Kernel\_Read\|xilinx:adm-pcie-7v3:1ddr:3.0-0\|cnn\_GOOD | Read | OCL Region | 1 | 1 | 102367 | 102368 | 0.00333333 | 5273.104049 | 5273.104052 |
| 5273.104122 | Kernel\_Read\|xilinx:adm-pcie-7v3:1ddr:3.0-0\|cnn\_GOOD | Read | OCL Region | 1 | 1 | 102389 | 102390 | 0.00333333 | 5273.104122 | 5273.104126 |
| 5273.104182 | Kernel\_Read\|xilinx:adm-pcie-7v3:1ddr:3.0-0\|cnn\_GOOD | Read | OCL Region | 1 | 1 | 102407 | 102408 | 0.00333333 | 5273.104182 | 5273.104186 |
| 5273.104249 | Kernel\_Read\|xilinx:adm-pcie-7v3:1ddr:3.0-0\|cnn\_GOOD | Read | OCL Region | 1 | 1 | 102427 | 102428 | 0.00333333 | 5273.104249 | 5273.104252 |
| 5273.104329 | Kernel\_Read\|xilinx:adm-pcie-7v3:1ddr:3.0-0\|cnn\_GOOD | Read | OCL Region | 1 | 1 | 102451 | 102452 | 0.00333333 | 5273.104329 | 5273.104332 |
| 5273.104382 | Kernel\_Read\|xilinx:adm-pcie-7v3:1ddr:3.0-0\|cnn\_GOOD | Read | OCL Region | 1 | 1 | 102467 | 102468 | 0.00333333 | 5273.104382 | 5273.104386 |
| 5273.104449 | Kernel\_Read\|xilinx:adm-pcie-7v3:1ddr:3.0-0\|cnn\_GOOD | Read | OCL Region | 1 | 1 | 102487 | 102488 | 0.00333333 | 5273.104449 | 5273.104452 |
| 5273.104522 | Kernel\_Read\|xilinx:adm-pcie-7v3:1ddr:3.0-0\|cnn\_GOOD | Read | OCL Region | 1 | 1 | 102509 | 102510 | 0.00333333 | 5273.104522 | 5273.104526 |
| 5273.104582 | Kernel\_Read\|xilinx:adm-pcie-7v3:1ddr:3.0-0\|cnn\_GOOD | Read | OCL Region | 1 | 1 | 102527 | 102528 | 0.00333333 | 5273.104582 | 5273.104586 |
| 5273.104649 | Kernel\_Read\|xilinx:adm-pcie-7v3:1ddr:3.0-0\|cnn\_GOOD | Read | OCL Region | 1 | 1 | 102547 | 102548 | 0.00333333 | 5273.104649 | 5273.104652 |
| 5273.104722 | Kernel\_Read\|xilinx:adm-pcie-7v3:1ddr:3.0-0\|cnn\_GOOD | Read | OCL Region | 1 | 1 | 102569 | 102570 | 0.00333333 | 5273.104722 | 5273.104726 |
| 5273.104782 | Kernel\_Read\|xilinx:adm-pcie-7v3:1ddr:3.0-0\|cnn\_GOOD | Read | OCL Region | 1 | 1 | 102587 | 102588 | 0.00333333 | 5273.104782 | 5273.104786 |
| 5273.104849 | Kernel\_Read\|xilinx:adm-pcie-7v3:1ddr:3.0-0\|cnn\_GOOD | Read | OCL Region | 1 | 1 | 102607 | 102608 | 0.00333333 | 5273.104849 | 5273.104852 |
| 5273.104922 | Kernel\_Read\|xilinx:adm-pcie-7v3:1ddr:3.0-0\|cnn\_GOOD | Read | OCL Region | 1 | 1 | 102629 | 102630 | 0.00333333 | 5273.104922 | 5273.104926 |
| 5273.104982 | Kernel\_Read\|xilinx:adm-pcie-7v3:1ddr:3.0-0\|cnn\_GOOD | Read | OCL Region | 1 | 1 | 102647 | 102648 | 0.00333333 | 5273.104982 | 5273.104986 |
| 5273.105049 | Kernel\_Read\|xilinx:adm-pcie-7v3:1ddr:3.0-0\|cnn\_GOOD | Read | OCL Region | 1 | 1 | 102667 | 102668 | 0.00333333 | 5273.105049 | 5273.105052 |
| 5273.105122 | Kernel\_Read\|xilinx:adm-pcie-7v3:1ddr:3.0-0\|cnn\_GOOD | Read | OCL Region | 1 | 1 | 102689 | 102690 | 0.00333333 | 5273.105122 | 5273.105126 |
| 5273.105182 | Kernel\_Read\|xilinx:adm-pcie-7v3:1ddr:3.0-0\|cnn\_GOOD | Read | OCL Region | 1 | 1 | 102707 | 102708 | 0.00333333 | 5273.105182 | 5273.105186 |
| 5273.105249 | Kernel\_Read\|xilinx:adm-pcie-7v3:1ddr:3.0-0\|cnn\_GOOD | Read | OCL Region | 1 | 1 | 102727 | 102728 | 0.00333333 | 5273.105249 | 5273.105252 |
| 5273.105322 | Kernel\_Read\|xilinx:adm-pcie-7v3:1ddr:3.0-0\|cnn\_GOOD | Read | OCL Region | 1 | 1 | 102749 | 102750 | 0.00333333 | 5273.105322 | 5273.105326 |
| 5273.105382 | Kernel\_Read\|xilinx:adm-pcie-7v3:1ddr:3.0-0\|cnn\_GOOD | Read | OCL Region | 1 | 1 | 102767 | 102768 | 0.00333333 | 5273.105382 | 5273.105386 |
| 5273.105659 | Kernel\_Read\|xilinx:adm-pcie-7v3:1ddr:3.0-0\|cnn\_GOOD | Read | OCL Region | 1 | 1 | 102850 | 102851 | 0.00333333 | 5273.105659 | 5273.105662 |
| 5273.105662 | Kernel\_Read\|xilinx:adm-pcie-7v3:1ddr:3.0-0\|cnn\_GOOD | Read | OCL Region | 1 | 1 | 102851 | 102852 | 0.00333333 | 5273.105662 | 5273.105666 |
| 5273.105672 | Kernel\_Read\|xilinx:adm-pcie-7v3:1ddr:3.0-0\|cnn\_GOOD | Read | OCL Region | 1 | 1 | 102854 | 102855 | 0.00333333 | 5273.105672 | 5273.105676 |
| 5273.105676 | Kernel\_Read\|xilinx:adm-pcie-7v3:1ddr:3.0-0\|cnn\_GOOD | Read | OCL Region | 1 | 1 | 102855 | 102856 | 0.00333333 | 5273.105676 | 5273.105679 |
| 5273.105732 | Kernel\_Read\|xilinx:adm-pcie-7v3:1ddr:3.0-0\|cnn\_GOOD | Read | OCL Region | 1 | 1 | 102872 | 102873 | 0.00333333 | 5273.105732 | 5273.105736 |
| 5273.105789 | Kernel\_Read\|xilinx:adm-pcie-7v3:1ddr:3.0-0\|cnn\_GOOD | Read | OCL Region | 1 | 1 | 102889 | 102890 | 0.00333333 | 5273.105789 | 5273.105792 |
| 5273.105849 | Kernel\_Read\|xilinx:adm-pcie-7v3:1ddr:3.0-0\|cnn\_GOOD | Read | OCL Region | 1 | 1 | 102907 | 102908 | 0.00333333 | 5273.105849 | 5273.105852 |
| 5273.105922 | Kernel\_Read\|xilinx:adm-pcie-7v3:1ddr:3.0-0\|cnn\_GOOD | Read | OCL Region | 1 | 1 | 102929 | 102930 | 0.00333333 | 5273.105922 | 5273.105926 |
| 5273.105982 | Kernel\_Read\|xilinx:adm-pcie-7v3:1ddr:3.0-0\|cnn\_GOOD | Read | OCL Region | 1 | 1 | 102947 | 102948 | 0.00333333 | 5273.105982 | 5273.105986 |
| 5273.106049 | Kernel\_Read\|xilinx:adm-pcie-7v3:1ddr:3.0-0\|cnn\_GOOD | Read | OCL Region | 1 | 1 | 102967 | 102968 | 0.00333333 | 5273.106049 | 5273.106052 |
| 5273.106122 | Kernel\_Read\|xilinx:adm-pcie-7v3:1ddr:3.0-0\|cnn\_GOOD | Read | OCL Region | 1 | 1 | 102989 | 102990 | 0.00333333 | 5273.106122 | 5273.106126 |
| 5273.106182 | Kernel\_Read\|xilinx:adm-pcie-7v3:1ddr:3.0-0\|cnn\_GOOD | Read | OCL Region | 1 | 1 | 103007 | 103008 | 0.00333333 | 5273.106182 | 5273.106186 |
| 5273.106249 | Kernel\_Read\|xilinx:adm-pcie-7v3:1ddr:3.0-0\|cnn\_GOOD | Read | OCL Region | 1 | 1 | 103027 | 103028 | 0.00333333 | 5273.106249 | 5273.106252 |
| 5273.106322 | Kernel\_Read\|xilinx:adm-pcie-7v3:1ddr:3.0-0\|cnn\_GOOD | Read | OCL Region | 1 | 1 | 103049 | 103050 | 0.00333333 | 5273.106322 | 5273.106326 |
| 5273.106382 | Kernel\_Read\|xilinx:adm-pcie-7v3:1ddr:3.0-0\|cnn\_GOOD | Read | OCL Region | 1 | 1 | 103067 | 103068 | 0.00333333 | 5273.106382 | 5273.106386 |
| 5273.106449 | Kernel\_Read\|xilinx:adm-pcie-7v3:1ddr:3.0-0\|cnn\_GOOD | Read | OCL Region | 1 | 1 | 103087 | 103088 | 0.00333333 | 5273.106449 | 5273.106452 |
| 5273.106522 | Kernel\_Read\|xilinx:adm-pcie-7v3:1ddr:3.0-0\|cnn\_GOOD | Read | OCL Region | 1 | 1 | 103109 | 103110 | 0.00333333 | 5273.106522 | 5273.106526 |
| 5273.106582 | Kernel\_Read\|xilinx:adm-pcie-7v3:1ddr:3.0-0\|cnn\_GOOD | Read | OCL Region | 1 | 1 | 103127 | 103128 | 0.00333333 | 5273.106582 | 5273.106586 |
| 5273.106649 | Kernel\_Read\|xilinx:adm-pcie-7v3:1ddr:3.0-0\|cnn\_GOOD | Read | OCL Region | 1 | 1 | 103147 | 103148 | 0.00333333 | 5273.106649 | 5273.106652 |
| 5273.106722 | Kernel\_Read\|xilinx:adm-pcie-7v3:1ddr:3.0-0\|cnn\_GOOD | Read | OCL Region | 1 | 1 | 103169 | 103170 | 0.00333333 | 5273.106722 | 5273.106726 |
| 5273.106782 | Kernel\_Read\|xilinx:adm-pcie-7v3:1ddr:3.0-0\|cnn\_GOOD | Read | OCL Region | 1 | 1 | 103187 | 103188 | 0.00333333 | 5273.106782 | 5273.106786 |
| 5273.106849 | Kernel\_Read\|xilinx:adm-pcie-7v3:1ddr:3.0-0\|cnn\_GOOD | Read | OCL Region | 1 | 1 | 103207 | 103208 | 0.00333333 | 5273.106849 | 5273.106852 |
| 5273.106922 | Kernel\_Read\|xilinx:adm-pcie-7v3:1ddr:3.0-0\|cnn\_GOOD | Read | OCL Region | 1 | 1 | 103229 | 103230 | 0.00333333 | 5273.106922 | 5273.106926 |
| 5273.106982 | Kernel\_Read\|xilinx:adm-pcie-7v3:1ddr:3.0-0\|cnn\_GOOD | Read | OCL Region | 1 | 1 | 103247 | 103248 | 0.00333333 | 5273.106982 | 5273.106986 |
| 5273.107049 | Kernel\_Read\|xilinx:adm-pcie-7v3:1ddr:3.0-0\|cnn\_GOOD | Read | OCL Region | 1 | 1 | 103267 | 103268 | 0.00333333 | 5273.107049 | 5273.107052 |
| 5273.107122 | Kernel\_Read\|xilinx:adm-pcie-7v3:1ddr:3.0-0\|cnn\_GOOD | Read | OCL Region | 1 | 1 | 103289 | 103290 | 0.00333333 | 5273.107122 | 5273.107126 |
| 5273.107182 | Kernel\_Read\|xilinx:adm-pcie-7v3:1ddr:3.0-0\|cnn\_GOOD | Read | OCL Region | 1 | 1 | 103307 | 103308 | 0.00333333 | 5273.107182 | 5273.107186 |
| 5273.107249 | Kernel\_Read\|xilinx:adm-pcie-7v3:1ddr:3.0-0\|cnn\_GOOD | Read | OCL Region | 1 | 1 | 103327 | 103328 | 0.00333333 | 5273.107249 | 5273.107252 |
| 5273.107322 | Kernel\_Read\|xilinx:adm-pcie-7v3:1ddr:3.0-0\|cnn\_GOOD | Read | OCL Region | 1 | 1 | 103349 | 103350 | 0.00333333 | 5273.107322 | 5273.107326 |
| 5273.107382 | Kernel\_Read\|xilinx:adm-pcie-7v3:1ddr:3.0-0\|cnn\_GOOD | Read | OCL Region | 1 | 1 | 103367 | 103368 | 0.00333333 | 5273.107382 | 5273.107386 |
| 5273.107449 | Kernel\_Read\|xilinx:adm-pcie-7v3:1ddr:3.0-0\|cnn\_GOOD | Read | OCL Region | 1 | 1 | 103387 | 103388 | 0.00333333 | 5273.107449 | 5273.107452 |
| 5273.107522 | Kernel\_Read\|xilinx:adm-pcie-7v3:1ddr:3.0-0\|cnn\_GOOD | Read | OCL Region | 1 | 1 | 103409 | 103410 | 0.00333333 | 5273.107522 | 5273.107526 |
| 5273.107582 | Kernel\_Read\|xilinx:adm-pcie-7v3:1ddr:3.0-0\|cnn\_GOOD | Read | OCL Region | 1 | 1 | 103427 | 103428 | 0.00333333 | 5273.107582 | 5273.107586 |
| 5273.107649 | Kernel\_Read\|xilinx:adm-pcie-7v3:1ddr:3.0-0\|cnn\_GOOD | Read | OCL Region | 1 | 1 | 103447 | 103448 | 0.00333333 | 5273.107649 | 5273.107652 |
| 5273.107722 | Kernel\_Read\|xilinx:adm-pcie-7v3:1ddr:3.0-0\|cnn\_GOOD | Read | OCL Region | 1 | 1 | 103469 | 103470 | 0.00333333 | 5273.107722 | 5273.107726 |
| 5273.107782 | Kernel\_Read\|xilinx:adm-pcie-7v3:1ddr:3.0-0\|cnn\_GOOD | Read | OCL Region | 1 | 1 | 103487 | 103488 | 0.00333333 | 5273.107782 | 5273.107786 |
| 5273.107849 | Kernel\_Read\|xilinx:adm-pcie-7v3:1ddr:3.0-0\|cnn\_GOOD | Read | OCL Region | 1 | 1 | 103507 | 103508 | 0.00333333 | 5273.107849 | 5273.107852 |
| 5273.107922 | Kernel\_Read\|xilinx:adm-pcie-7v3:1ddr:3.0-0\|cnn\_GOOD | Read | OCL Region | 1 | 1 | 103529 | 103530 | 0.00333333 | 5273.107922 | 5273.107926 |
| 5273.107982 | Kernel\_Read\|xilinx:adm-pcie-7v3:1ddr:3.0-0\|cnn\_GOOD | Read | OCL Region | 1 | 1 | 103547 | 103548 | 0.00333333 | 5273.107982 | 5273.107986 |
| 5273.108049 | Kernel\_Read\|xilinx:adm-pcie-7v3:1ddr:3.0-0\|cnn\_GOOD | Read | OCL Region | 1 | 1 | 103567 | 103568 | 0.00333333 | 5273.108049 | 5273.108052 |
| 5273.108126 | Kernel\_Read\|xilinx:adm-pcie-7v3:1ddr:3.0-0\|cnn\_GOOD | Read | OCL Region | 1 | 1 | 103590 | 103591 | 0.00333333 | 5273.108126 | 5273.108129 |
| 5273.108182 | Kernel\_Read\|xilinx:adm-pcie-7v3:1ddr:3.0-0\|cnn\_GOOD | Read | OCL Region | 1 | 1 | 103607 | 103608 | 0.00333333 | 5273.108182 | 5273.108186 |
| 5273.108249 | Kernel\_Read\|xilinx:adm-pcie-7v3:1ddr:3.0-0\|cnn\_GOOD | Read | OCL Region | 1 | 1 | 103627 | 103628 | 0.00333333 | 5273.108249 | 5273.108252 |
| 5273.108322 | Kernel\_Read\|xilinx:adm-pcie-7v3:1ddr:3.0-0\|cnn\_GOOD | Read | OCL Region | 1 | 1 | 103649 | 103650 | 0.00333333 | 5273.108322 | 5273.108326 |
| 5273.108382 | Kernel\_Read\|xilinx:adm-pcie-7v3:1ddr:3.0-0\|cnn\_GOOD | Read | OCL Region | 1 | 1 | 103667 | 103668 | 0.00333333 | 5273.108382 | 5273.108386 |
| 5273.108449 | Kernel\_Read\|xilinx:adm-pcie-7v3:1ddr:3.0-0\|cnn\_GOOD | Read | OCL Region | 1 | 1 | 103687 | 103688 | 0.00333333 | 5273.108449 | 5273.108452 |
| 5273.108522 | Kernel\_Read\|xilinx:adm-pcie-7v3:1ddr:3.0-0\|cnn\_GOOD | Read | OCL Region | 1 | 1 | 103709 | 103710 | 0.00333333 | 5273.108522 | 5273.108526 |
| 5273.108582 | Kernel\_Read\|xilinx:adm-pcie-7v3:1ddr:3.0-0\|cnn\_GOOD | Read | OCL Region | 1 | 1 | 103727 | 103728 | 0.00333333 | 5273.108582 | 5273.108586 |
| 5273.108649 | Kernel\_Read\|xilinx:adm-pcie-7v3:1ddr:3.0-0\|cnn\_GOOD | Read | OCL Region | 1 | 1 | 103747 | 103748 | 0.00333333 | 5273.108649 | 5273.108652 |
| 5273.108722 | Kernel\_Read\|xilinx:adm-pcie-7v3:1ddr:3.0-0\|cnn\_GOOD | Read | OCL Region | 1 | 1 | 103769 | 103770 | 0.00333333 | 5273.108722 | 5273.108726 |
| 5273.108782 | Kernel\_Read\|xilinx:adm-pcie-7v3:1ddr:3.0-0\|cnn\_GOOD | Read | OCL Region | 1 | 1 | 103787 | 103788 | 0.00333333 | 5273.108782 | 5273.108786 |
| 5273.108849 | Kernel\_Read\|xilinx:adm-pcie-7v3:1ddr:3.0-0\|cnn\_GOOD | Read | OCL Region | 1 | 1 | 103807 | 103808 | 0.00333333 | 5273.108849 | 5273.108852 |
| 5273.108912 | Kernel\_Read\|xilinx:adm-pcie-7v3:1ddr:3.0-0\|cnn\_GOOD | Read | OCL Region | 1 | 1 | 103826 | 103827 | 0.00333333 | 5273.108912 | 5273.108916 |
| 5273.108982 | Kernel\_Read\|xilinx:adm-pcie-7v3:1ddr:3.0-0\|cnn\_GOOD | Read | OCL Region | 1 | 1 | 103847 | 103848 | 0.00333333 | 5273.108982 | 5273.108986 |
| 5273.109049 | Kernel\_Read\|xilinx:adm-pcie-7v3:1ddr:3.0-0\|cnn\_GOOD | Read | OCL Region | 1 | 1 | 103867 | 103868 | 0.00333333 | 5273.109049 | 5273.109052 |
| 5273.109122 | Kernel\_Read\|xilinx:adm-pcie-7v3:1ddr:3.0-0\|cnn\_GOOD | Read | OCL Region | 1 | 1 | 103889 | 103890 | 0.00333333 | 5273.109122 | 5273.109126 |
| 5273.109182 | Kernel\_Read\|xilinx:adm-pcie-7v3:1ddr:3.0-0\|cnn\_GOOD | Read | OCL Region | 1 | 1 | 103907 | 103908 | 0.00333333 | 5273.109182 | 5273.109186 |
| 5273.109249 | Kernel\_Read\|xilinx:adm-pcie-7v3:1ddr:3.0-0\|cnn\_GOOD | Read | OCL Region | 1 | 1 | 103927 | 103928 | 0.00333333 | 5273.109249 | 5273.109252 |
| 5273.109322 | Kernel\_Read\|xilinx:adm-pcie-7v3:1ddr:3.0-0\|cnn\_GOOD | Read | OCL Region | 1 | 1 | 103949 | 103950 | 0.00333333 | 5273.109322 | 5273.109326 |
| 5273.109382 | Kernel\_Read\|xilinx:adm-pcie-7v3:1ddr:3.0-0\|cnn\_GOOD | Read | OCL Region | 1 | 1 | 103967 | 103968 | 0.00333333 | 5273.109382 | 5273.109386 |
| 5273.109449 | Kernel\_Read\|xilinx:adm-pcie-7v3:1ddr:3.0-0\|cnn\_GOOD | Read | OCL Region | 1 | 1 | 103987 | 103988 | 0.00333333 | 5273.109449 | 5273.109452 |
| 5273.109522 | Kernel\_Read\|xilinx:adm-pcie-7v3:1ddr:3.0-0\|cnn\_GOOD | Read | OCL Region | 1 | 1 | 104009 | 104010 | 0.00333333 | 5273.109522 | 5273.109526 |
| 5273.109582 | Kernel\_Read\|xilinx:adm-pcie-7v3:1ddr:3.0-0\|cnn\_GOOD | Read | OCL Region | 1 | 1 | 104027 | 104028 | 0.00333333 | 5273.109582 | 5273.109586 |
| 5273.109649 | Kernel\_Read\|xilinx:adm-pcie-7v3:1ddr:3.0-0\|cnn\_GOOD | Read | OCL Region | 1 | 1 | 104047 | 104048 | 0.00333333 | 5273.109649 | 5273.109652 |
| 5273.109722 | Kernel\_Read\|xilinx:adm-pcie-7v3:1ddr:3.0-0\|cnn\_GOOD | Read | OCL Region | 1 | 1 | 104069 | 104070 | 0.00333333 | 5273.109722 | 5273.109726 |
| 5273.109782 | Kernel\_Read\|xilinx:adm-pcie-7v3:1ddr:3.0-0\|cnn\_GOOD | Read | OCL Region | 1 | 1 | 104087 | 104088 | 0.00333333 | 5273.109782 | 5273.109786 |
| 5273.109849 | Kernel\_Read\|xilinx:adm-pcie-7v3:1ddr:3.0-0\|cnn\_GOOD | Read | OCL Region | 1 | 1 | 104107 | 104108 | 0.00333333 | 5273.109849 | 5273.109852 |
| 5273.109922 | Kernel\_Read\|xilinx:adm-pcie-7v3:1ddr:3.0-0\|cnn\_GOOD | Read | OCL Region | 1 | 1 | 104129 | 104130 | 0.00333333 | 5273.109922 | 5273.109926 |
| 5273.109982 | Kernel\_Read\|xilinx:adm-pcie-7v3:1ddr:3.0-0\|cnn\_GOOD | Read | OCL Region | 1 | 1 | 104147 | 104148 | 0.00333333 | 5273.109982 | 5273.109986 |
| 5273.110049 | Kernel\_Read\|xilinx:adm-pcie-7v3:1ddr:3.0-0\|cnn\_GOOD | Read | OCL Region | 1 | 1 | 104167 | 104168 | 0.00333333 | 5273.110049 | 5273.110052 |
| 5273.110122 | Kernel\_Read\|xilinx:adm-pcie-7v3:1ddr:3.0-0\|cnn\_GOOD | Read | OCL Region | 1 | 1 | 104189 | 104190 | 0.00333333 | 5273.110122 | 5273.110126 |
| 5273.110182 | Kernel\_Read\|xilinx:adm-pcie-7v3:1ddr:3.0-0\|cnn\_GOOD | Read | OCL Region | 1 | 1 | 104207 | 104208 | 0.00333333 | 5273.110182 | 5273.110186 |
| 5273.110249 | Kernel\_Read\|xilinx:adm-pcie-7v3:1ddr:3.0-0\|cnn\_GOOD | Read | OCL Region | 1 | 1 | 104227 | 104228 | 0.00333333 | 5273.110249 | 5273.110252 |
| 5273.110322 | Kernel\_Read\|xilinx:adm-pcie-7v3:1ddr:3.0-0\|cnn\_GOOD | Read | OCL Region | 1 | 1 | 104249 | 104250 | 0.00333333 | 5273.110322 | 5273.110326 |
| 5273.110382 | Kernel\_Read\|xilinx:adm-pcie-7v3:1ddr:3.0-0\|cnn\_GOOD | Read | OCL Region | 1 | 1 | 104267 | 104268 | 0.00333333 | 5273.110382 | 5273.110386 |
| 5273.110449 | Kernel\_Read\|xilinx:adm-pcie-7v3:1ddr:3.0-0\|cnn\_GOOD | Read | OCL Region | 1 | 1 | 104287 | 104288 | 0.00333333 | 5273.110449 | 5273.110452 |
| 5273.110522 | Kernel\_Read\|xilinx:adm-pcie-7v3:1ddr:3.0-0\|cnn\_GOOD | Read | OCL Region | 1 | 1 | 104309 | 104310 | 0.00333333 | 5273.110522 | 5273.110526 |
| 5273.110599 | Kernel\_Read\|xilinx:adm-pcie-7v3:1ddr:3.0-0\|cnn\_GOOD | Read | OCL Region | 1 | 1 | 104332 | 104333 | 0.00333333 | 5273.110599 | 5273.110602 |
| 5273.110656 | Kernel\_Read\|xilinx:adm-pcie-7v3:1ddr:3.0-0\|cnn\_GOOD | Read | OCL Region | 1 | 1 | 104349 | 104350 | 0.00333333 | 5273.110656 | 5273.110659 |
| 5273.110722 | Kernel\_Read\|xilinx:adm-pcie-7v3:1ddr:3.0-0\|cnn\_GOOD | Read | OCL Region | 1 | 1 | 104369 | 104370 | 0.00333333 | 5273.110722 | 5273.110726 |
| 5273.110782 | Kernel\_Read\|xilinx:adm-pcie-7v3:1ddr:3.0-0\|cnn\_GOOD | Read | OCL Region | 1 | 1 | 104387 | 104388 | 0.00333333 | 5273.110782 | 5273.110786 |
| 5273.110849 | Kernel\_Read\|xilinx:adm-pcie-7v3:1ddr:3.0-0\|cnn\_GOOD | Read | OCL Region | 1 | 1 | 104407 | 104408 | 0.00333333 | 5273.110849 | 5273.110852 |
| 5273.110922 | Kernel\_Read\|xilinx:adm-pcie-7v3:1ddr:3.0-0\|cnn\_GOOD | Read | OCL Region | 1 | 1 | 104429 | 104430 | 0.00333333 | 5273.110922 | 5273.110926 |
| 5273.110982 | Kernel\_Read\|xilinx:adm-pcie-7v3:1ddr:3.0-0\|cnn\_GOOD | Read | OCL Region | 1 | 1 | 104447 | 104448 | 0.00333333 | 5273.110982 | 5273.110986 |
| 5273.111049 | Kernel\_Read\|xilinx:adm-pcie-7v3:1ddr:3.0-0\|cnn\_GOOD | Read | OCL Region | 1 | 1 | 104467 | 104468 | 0.00333333 | 5273.111049 | 5273.111052 |
| 5273.111122 | Kernel\_Read\|xilinx:adm-pcie-7v3:1ddr:3.0-0\|cnn\_GOOD | Read | OCL Region | 1 | 1 | 104489 | 104490 | 0.00333333 | 5273.111122 | 5273.111126 |
| 5273.111182 | Kernel\_Read\|xilinx:adm-pcie-7v3:1ddr:3.0-0\|cnn\_GOOD | Read | OCL Region | 1 | 1 | 104507 | 104508 | 0.00333333 | 5273.111182 | 5273.111186 |
| 5273.111249 | Kernel\_Read\|xilinx:adm-pcie-7v3:1ddr:3.0-0\|cnn\_GOOD | Read | OCL Region | 1 | 1 | 104527 | 104528 | 0.00333333 | 5273.111249 | 5273.111252 |
| 5273.111322 | Kernel\_Read\|xilinx:adm-pcie-7v3:1ddr:3.0-0\|cnn\_GOOD | Read | OCL Region | 1 | 1 | 104549 | 104550 | 0.00333333 | 5273.111322 | 5273.111326 |
| 5273.111389 | Kernel\_Read\|xilinx:adm-pcie-7v3:1ddr:3.0-0\|cnn\_GOOD | Read | OCL Region | 1 | 1 | 104569 | 104570 | 0.00333333 | 5273.111389 | 5273.111392 |
| 5273.111449 | Kernel\_Read\|xilinx:adm-pcie-7v3:1ddr:3.0-0\|cnn\_GOOD | Read | OCL Region | 1 | 1 | 104587 | 104588 | 0.00333333 | 5273.111449 | 5273.111452 |
| 5273.111522 | Kernel\_Read\|xilinx:adm-pcie-7v3:1ddr:3.0-0\|cnn\_GOOD | Read | OCL Region | 1 | 1 | 104609 | 104610 | 0.00333333 | 5273.111522 | 5273.111526 |
| 5273.111582 | Kernel\_Read\|xilinx:adm-pcie-7v3:1ddr:3.0-0\|cnn\_GOOD | Read | OCL Region | 1 | 1 | 104627 | 104628 | 0.00333333 | 5273.111582 | 5273.111586 |
| 5273.111649 | Kernel\_Read\|xilinx:adm-pcie-7v3:1ddr:3.0-0\|cnn\_GOOD | Read | OCL Region | 1 | 1 | 104647 | 104648 | 0.00333333 | 5273.111649 | 5273.111652 |
| 5273.111929 | Kernel\_Read\|xilinx:adm-pcie-7v3:1ddr:3.0-0\|cnn\_GOOD | Read | OCL Region | 1 | 1 | 104731 | 104732 | 0.00333333 | 5273.111929 | 5273.111932 |
| 5273.111932 | Kernel\_Read\|xilinx:adm-pcie-7v3:1ddr:3.0-0\|cnn\_GOOD | Read | OCL Region | 1 | 1 | 104732 | 104733 | 0.00333333 | 5273.111932 | 5273.111936 |
| 5273.111939 | Kernel\_Read\|xilinx:adm-pcie-7v3:1ddr:3.0-0\|cnn\_GOOD | Read | OCL Region | 1 | 1 | 104734 | 104735 | 0.00333333 | 5273.111939 | 5273.111942 |
| 5273.111946 | Kernel\_Read\|xilinx:adm-pcie-7v3:1ddr:3.0-0\|cnn\_GOOD | Read | OCL Region | 1 | 1 | 104736 | 104737 | 0.00333333 | 5273.111946 | 5273.111949 |
| 5273.112006 | Kernel\_Read\|xilinx:adm-pcie-7v3:1ddr:3.0-0\|cnn\_GOOD | Read | OCL Region | 1 | 1 | 104754 | 104755 | 0.00333333 | 5273.112006 | 5273.112009 |
| 5273.112059 | Kernel\_Read\|xilinx:adm-pcie-7v3:1ddr:3.0-0\|cnn\_GOOD | Read | OCL Region | 1 | 1 | 104770 | 104771 | 0.00333333 | 5273.112059 | 5273.112062 |
| 5273.112122 | Kernel\_Read\|xilinx:adm-pcie-7v3:1ddr:3.0-0\|cnn\_GOOD | Read | OCL Region | 1 | 1 | 104789 | 104790 | 0.00333333 | 5273.112122 | 5273.112126 |
| 5273.112182 | Kernel\_Read\|xilinx:adm-pcie-7v3:1ddr:3.0-0\|cnn\_GOOD | Read | OCL Region | 1 | 1 | 104807 | 104808 | 0.00333333 | 5273.112182 | 5273.112186 |
| 5273.112249 | Kernel\_Read\|xilinx:adm-pcie-7v3:1ddr:3.0-0\|cnn\_GOOD | Read | OCL Region | 1 | 1 | 104827 | 104828 | 0.00333333 | 5273.112249 | 5273.112252 |
| 5273.112322 | Kernel\_Read\|xilinx:adm-pcie-7v3:1ddr:3.0-0\|cnn\_GOOD | Read | OCL Region | 1 | 1 | 104849 | 104850 | 0.00333333 | 5273.112322 | 5273.112326 |
| 5273.112382 | Kernel\_Read\|xilinx:adm-pcie-7v3:1ddr:3.0-0\|cnn\_GOOD | Read | OCL Region | 1 | 1 | 104867 | 104868 | 0.00333333 | 5273.112382 | 5273.112386 |
| 5273.112449 | Kernel\_Read\|xilinx:adm-pcie-7v3:1ddr:3.0-0\|cnn\_GOOD | Read | OCL Region | 1 | 1 | 104887 | 104888 | 0.00333333 | 5273.112449 | 5273.112452 |
| 5273.112522 | Kernel\_Read\|xilinx:adm-pcie-7v3:1ddr:3.0-0\|cnn\_GOOD | Read | OCL Region | 1 | 1 | 104909 | 104910 | 0.00333333 | 5273.112522 | 5273.112526 |
| 5273.112582 | Kernel\_Read\|xilinx:adm-pcie-7v3:1ddr:3.0-0\|cnn\_GOOD | Read | OCL Region | 1 | 1 | 104927 | 104928 | 0.00333333 | 5273.112582 | 5273.112586 |
| 5273.112649 | Kernel\_Read\|xilinx:adm-pcie-7v3:1ddr:3.0-0\|cnn\_GOOD | Read | OCL Region | 1 | 1 | 104947 | 104948 | 0.00333333 | 5273.112649 | 5273.112652 |
| 5273.112712 | Kernel\_Read\|xilinx:adm-pcie-7v3:1ddr:3.0-0\|cnn\_GOOD | Read | OCL Region | 1 | 1 | 104966 | 104967 | 0.00333333 | 5273.112712 | 5273.112716 |
| 5273.112782 | Kernel\_Read\|xilinx:adm-pcie-7v3:1ddr:3.0-0\|cnn\_GOOD | Read | OCL Region | 1 | 1 | 104987 | 104988 | 0.00333333 | 5273.112782 | 5273.112786 |
| 5273.112849 | Kernel\_Read\|xilinx:adm-pcie-7v3:1ddr:3.0-0\|cnn\_GOOD | Read | OCL Region | 1 | 1 | 105007 | 105008 | 0.00333333 | 5273.112849 | 5273.112852 |
| 5273.112922 | Kernel\_Read\|xilinx:adm-pcie-7v3:1ddr:3.0-0\|cnn\_GOOD | Read | OCL Region | 1 | 1 | 105029 | 105030 | 0.00333333 | 5273.112922 | 5273.112926 |
| 5273.112982 | Kernel\_Read\|xilinx:adm-pcie-7v3:1ddr:3.0-0\|cnn\_GOOD | Read | OCL Region | 1 | 1 | 105047 | 105048 | 0.00333333 | 5273.112982 | 5273.112986 |
| 5273.113049 | Kernel\_Read\|xilinx:adm-pcie-7v3:1ddr:3.0-0\|cnn\_GOOD | Read | OCL Region | 1 | 1 | 105067 | 105068 | 0.00333333 | 5273.113049 | 5273.113052 |
| 5273.113122 | Kernel\_Read\|xilinx:adm-pcie-7v3:1ddr:3.0-0\|cnn\_GOOD | Read | OCL Region | 1 | 1 | 105089 | 105090 | 0.00333333 | 5273.113122 | 5273.113126 |
| 5273.113182 | Kernel\_Read\|xilinx:adm-pcie-7v3:1ddr:3.0-0\|cnn\_GOOD | Read | OCL Region | 1 | 1 | 105107 | 105108 | 0.00333333 | 5273.113182 | 5273.113186 |
| 5273.113249 | Kernel\_Read\|xilinx:adm-pcie-7v3:1ddr:3.0-0\|cnn\_GOOD | Read | OCL Region | 1 | 1 | 105127 | 105128 | 0.00333333 | 5273.113249 | 5273.113252 |
| 5273.113322 | Kernel\_Read\|xilinx:adm-pcie-7v3:1ddr:3.0-0\|cnn\_GOOD | Read | OCL Region | 1 | 1 | 105149 | 105150 | 0.00333333 | 5273.113322 | 5273.113326 |
| 5273.113382 | Kernel\_Read\|xilinx:adm-pcie-7v3:1ddr:3.0-0\|cnn\_GOOD | Read | OCL Region | 1 | 1 | 105167 | 105168 | 0.00333333 | 5273.113382 | 5273.113386 |
| 5273.113449 | Kernel\_Read\|xilinx:adm-pcie-7v3:1ddr:3.0-0\|cnn\_GOOD | Read | OCL Region | 1 | 1 | 105187 | 105188 | 0.00333333 | 5273.113449 | 5273.113452 |
| 5273.113522 | Kernel\_Read\|xilinx:adm-pcie-7v3:1ddr:3.0-0\|cnn\_GOOD | Read | OCL Region | 1 | 1 | 105209 | 105210 | 0.00333333 | 5273.113522 | 5273.113526 |
| 5273.113582 | Kernel\_Read\|xilinx:adm-pcie-7v3:1ddr:3.0-0\|cnn\_GOOD | Read | OCL Region | 1 | 1 | 105227 | 105228 | 0.00333333 | 5273.113582 | 5273.113586 |
| 5273.113649 | Kernel\_Read\|xilinx:adm-pcie-7v3:1ddr:3.0-0\|cnn\_GOOD | Read | OCL Region | 1 | 1 | 105247 | 105248 | 0.00333333 | 5273.113649 | 5273.113652 |
| 5273.113722 | Kernel\_Read\|xilinx:adm-pcie-7v3:1ddr:3.0-0\|cnn\_GOOD | Read | OCL Region | 1 | 1 | 105269 | 105270 | 0.00333333 | 5273.113722 | 5273.113726 |
| 5273.113782 | Kernel\_Read\|xilinx:adm-pcie-7v3:1ddr:3.0-0\|cnn\_GOOD | Read | OCL Region | 1 | 1 | 105287 | 105288 | 0.00333333 | 5273.113782 | 5273.113786 |
| 5273.113849 | Kernel\_Read\|xilinx:adm-pcie-7v3:1ddr:3.0-0\|cnn\_GOOD | Read | OCL Region | 1 | 1 | 105307 | 105308 | 0.00333333 | 5273.113849 | 5273.113852 |
| 5273.113922 | Kernel\_Read\|xilinx:adm-pcie-7v3:1ddr:3.0-0\|cnn\_GOOD | Read | OCL Region | 1 | 1 | 105329 | 105330 | 0.00333333 | 5273.113922 | 5273.113926 |
| 5273.113982 | Kernel\_Read\|xilinx:adm-pcie-7v3:1ddr:3.0-0\|cnn\_GOOD | Read | OCL Region | 1 | 1 | 105347 | 105348 | 0.00333333 | 5273.113982 | 5273.113986 |
| 5273.114049 | Kernel\_Read\|xilinx:adm-pcie-7v3:1ddr:3.0-0\|cnn\_GOOD | Read | OCL Region | 1 | 1 | 105367 | 105368 | 0.00333333 | 5273.114049 | 5273.114052 |
| 5273.114122 | Kernel\_Read\|xilinx:adm-pcie-7v3:1ddr:3.0-0\|cnn\_GOOD | Read | OCL Region | 1 | 1 | 105389 | 105390 | 0.00333333 | 5273.114122 | 5273.114126 |
| 5273.114182 | Kernel\_Read\|xilinx:adm-pcie-7v3:1ddr:3.0-0\|cnn\_GOOD | Read | OCL Region | 1 | 1 | 105407 | 105408 | 0.00333333 | 5273.114182 | 5273.114186 |
| 5273.114249 | Kernel\_Read\|xilinx:adm-pcie-7v3:1ddr:3.0-0\|cnn\_GOOD | Read | OCL Region | 1 | 1 | 105427 | 105428 | 0.00333333 | 5273.114249 | 5273.114252 |
| 5273.114322 | Kernel\_Read\|xilinx:adm-pcie-7v3:1ddr:3.0-0\|cnn\_GOOD | Read | OCL Region | 1 | 1 | 105449 | 105450 | 0.00333333 | 5273.114322 | 5273.114326 |
| 5273.114396 | Kernel\_Read\|xilinx:adm-pcie-7v3:1ddr:3.0-0\|cnn\_GOOD | Read | OCL Region | 1 | 1 | 105471 | 105472 | 0.00333333 | 5273.114396 | 5273.114399 |
| 5273.114449 | Kernel\_Read\|xilinx:adm-pcie-7v3:1ddr:3.0-0\|cnn\_GOOD | Read | OCL Region | 1 | 1 | 105487 | 105488 | 0.00333333 | 5273.114449 | 5273.114452 |
| 5273.114522 | Kernel\_Read\|xilinx:adm-pcie-7v3:1ddr:3.0-0\|cnn\_GOOD | Read | OCL Region | 1 | 1 | 105509 | 105510 | 0.00333333 | 5273.114522 | 5273.114526 |
| 5273.114582 | Kernel\_Read\|xilinx:adm-pcie-7v3:1ddr:3.0-0\|cnn\_GOOD | Read | OCL Region | 1 | 1 | 105527 | 105528 | 0.00333333 | 5273.114582 | 5273.114586 |
| 5273.114649 | Kernel\_Read\|xilinx:adm-pcie-7v3:1ddr:3.0-0\|cnn\_GOOD | Read | OCL Region | 1 | 1 | 105547 | 105548 | 0.00333333 | 5273.114649 | 5273.114652 |
| 5273.114722 | Kernel\_Read\|xilinx:adm-pcie-7v3:1ddr:3.0-0\|cnn\_GOOD | Read | OCL Region | 1 | 1 | 105569 | 105570 | 0.00333333 | 5273.114722 | 5273.114726 |
| 5273.114782 | Kernel\_Read\|xilinx:adm-pcie-7v3:1ddr:3.0-0\|cnn\_GOOD | Read | OCL Region | 1 | 1 | 105587 | 105588 | 0.00333333 | 5273.114782 | 5273.114786 |
| 5273.114849 | Kernel\_Read\|xilinx:adm-pcie-7v3:1ddr:3.0-0\|cnn\_GOOD | Read | OCL Region | 1 | 1 | 105607 | 105608 | 0.00333333 | 5273.114849 | 5273.114852 |
| 5273.114922 | Kernel\_Read\|xilinx:adm-pcie-7v3:1ddr:3.0-0\|cnn\_GOOD | Read | OCL Region | 1 | 1 | 105629 | 105630 | 0.00333333 | 5273.114922 | 5273.114926 |
| 5273.114982 | Kernel\_Read\|xilinx:adm-pcie-7v3:1ddr:3.0-0\|cnn\_GOOD | Read | OCL Region | 1 | 1 | 105647 | 105648 | 0.00333333 | 5273.114982 | 5273.114986 |
| 5273.115049 | Kernel\_Read\|xilinx:adm-pcie-7v3:1ddr:3.0-0\|cnn\_GOOD | Read | OCL Region | 1 | 1 | 105667 | 105668 | 0.00333333 | 5273.115049 | 5273.115052 |
| 5273.115122 | Kernel\_Read\|xilinx:adm-pcie-7v3:1ddr:3.0-0\|cnn\_GOOD | Read | OCL Region | 1 | 1 | 105689 | 105690 | 0.00333333 | 5273.115122 | 5273.115126 |
| 5273.115182 | Kernel\_Read\|xilinx:adm-pcie-7v3:1ddr:3.0-0\|cnn\_GOOD | Read | OCL Region | 1 | 1 | 105707 | 105708 | 0.00333333 | 5273.115182 | 5273.115186 |
| 5273.115249 | Kernel\_Read\|xilinx:adm-pcie-7v3:1ddr:3.0-0\|cnn\_GOOD | Read | OCL Region | 1 | 1 | 105727 | 105728 | 0.00333333 | 5273.115249 | 5273.115252 |
| 5273.115322 | Kernel\_Read\|xilinx:adm-pcie-7v3:1ddr:3.0-0\|cnn\_GOOD | Read | OCL Region | 1 | 1 | 105749 | 105750 | 0.00333333 | 5273.115322 | 5273.115326 |
| 5273.115382 | Kernel\_Read\|xilinx:adm-pcie-7v3:1ddr:3.0-0\|cnn\_GOOD | Read | OCL Region | 1 | 1 | 105767 | 105768 | 0.00333333 | 5273.115382 | 5273.115386 |
| 5273.115449 | Kernel\_Read\|xilinx:adm-pcie-7v3:1ddr:3.0-0\|cnn\_GOOD | Read | OCL Region | 1 | 1 | 105787 | 105788 | 0.00333333 | 5273.115449 | 5273.115452 |
| 5273.115522 | Kernel\_Read\|xilinx:adm-pcie-7v3:1ddr:3.0-0\|cnn\_GOOD | Read | OCL Region | 1 | 1 | 105809 | 105810 | 0.00333333 | 5273.115522 | 5273.115526 |
| 5273.115582 | Kernel\_Read\|xilinx:adm-pcie-7v3:1ddr:3.0-0\|cnn\_GOOD | Read | OCL Region | 1 | 1 | 105827 | 105828 | 0.00333333 | 5273.115582 | 5273.115586 |
| 5273.115649 | Kernel\_Read\|xilinx:adm-pcie-7v3:1ddr:3.0-0\|cnn\_GOOD | Read | OCL Region | 1 | 1 | 105847 | 105848 | 0.00333333 | 5273.115649 | 5273.115652 |
| 5273.115722 | Kernel\_Read\|xilinx:adm-pcie-7v3:1ddr:3.0-0\|cnn\_GOOD | Read | OCL Region | 1 | 1 | 105869 | 105870 | 0.00333333 | 5273.115722 | 5273.115726 |
| 5273.115782 | Kernel\_Read\|xilinx:adm-pcie-7v3:1ddr:3.0-0\|cnn\_GOOD | Read | OCL Region | 1 | 1 | 105887 | 105888 | 0.00333333 | 5273.115782 | 5273.115786 |
| 5273.115849 | Kernel\_Read\|xilinx:adm-pcie-7v3:1ddr:3.0-0\|cnn\_GOOD | Read | OCL Region | 1 | 1 | 105907 | 105908 | 0.00333333 | 5273.115849 | 5273.115852 |
| 5273.115922 | Kernel\_Read\|xilinx:adm-pcie-7v3:1ddr:3.0-0\|cnn\_GOOD | Read | OCL Region | 1 | 1 | 105929 | 105930 | 0.00333333 | 5273.115922 | 5273.115926 |
| 5273.115982 | Kernel\_Read\|xilinx:adm-pcie-7v3:1ddr:3.0-0\|cnn\_GOOD | Read | OCL Region | 1 | 1 | 105947 | 105948 | 0.00333333 | 5273.115982 | 5273.115986 |
| 5273.116049 | Kernel\_Read\|xilinx:adm-pcie-7v3:1ddr:3.0-0\|cnn\_GOOD | Read | OCL Region | 1 | 1 | 105967 | 105968 | 0.00333333 | 5273.116049 | 5273.116052 |
| 5273.116122 | Kernel\_Read\|xilinx:adm-pcie-7v3:1ddr:3.0-0\|cnn\_GOOD | Read | OCL Region | 1 | 1 | 105989 | 105990 | 0.00333333 | 5273.116122 | 5273.116126 |
| 5273.116182 | Kernel\_Read\|xilinx:adm-pcie-7v3:1ddr:3.0-0\|cnn\_GOOD | Read | OCL Region | 1 | 1 | 106007 | 106008 | 0.00333333 | 5273.116182 | 5273.116186 |
| 5273.116249 | Kernel\_Read\|xilinx:adm-pcie-7v3:1ddr:3.0-0\|cnn\_GOOD | Read | OCL Region | 1 | 1 | 106027 | 106028 | 0.00333333 | 5273.116249 | 5273.116252 |
| 5273.116322 | Kernel\_Read\|xilinx:adm-pcie-7v3:1ddr:3.0-0\|cnn\_GOOD | Read | OCL Region | 1 | 1 | 106049 | 106050 | 0.00333333 | 5273.116322 | 5273.116326 |
| 5273.116382 | Kernel\_Read\|xilinx:adm-pcie-7v3:1ddr:3.0-0\|cnn\_GOOD | Read | OCL Region | 1 | 1 | 106067 | 106068 | 0.00333333 | 5273.116382 | 5273.116386 |
| 5273.116449 | Kernel\_Read\|xilinx:adm-pcie-7v3:1ddr:3.0-0\|cnn\_GOOD | Read | OCL Region | 1 | 1 | 106087 | 106088 | 0.00333333 | 5273.116449 | 5273.116452 |
| 5273.116522 | Kernel\_Read\|xilinx:adm-pcie-7v3:1ddr:3.0-0\|cnn\_GOOD | Read | OCL Region | 1 | 1 | 106109 | 106110 | 0.00333333 | 5273.116522 | 5273.116526 |
| 5273.116582 | Kernel\_Read\|xilinx:adm-pcie-7v3:1ddr:3.0-0\|cnn\_GOOD | Read | OCL Region | 1 | 1 | 106127 | 106128 | 0.00333333 | 5273.116582 | 5273.116586 |
| 5273.116649 | Kernel\_Read\|xilinx:adm-pcie-7v3:1ddr:3.0-0\|cnn\_GOOD | Read | OCL Region | 1 | 1 | 106147 | 106148 | 0.00333333 | 5273.116649 | 5273.116652 |
| 5273.116722 | Kernel\_Read\|xilinx:adm-pcie-7v3:1ddr:3.0-0\|cnn\_GOOD | Read | OCL Region | 1 | 1 | 106169 | 106170 | 0.00333333 | 5273.116722 | 5273.116726 |
| 5273.116782 | Kernel\_Read\|xilinx:adm-pcie-7v3:1ddr:3.0-0\|cnn\_GOOD | Read | OCL Region | 1 | 1 | 106187 | 106188 | 0.00333333 | 5273.116782 | 5273.116786 |
| 5273.116849 | Kernel\_Read\|xilinx:adm-pcie-7v3:1ddr:3.0-0\|cnn\_GOOD | Read | OCL Region | 1 | 1 | 106207 | 106208 | 0.00333333 | 5273.116849 | 5273.116852 |
| 5273.116922 | Kernel\_Read\|xilinx:adm-pcie-7v3:1ddr:3.0-0\|cnn\_GOOD | Read | OCL Region | 1 | 1 | 106229 | 106230 | 0.00333333 | 5273.116922 | 5273.116926 |
| 5273.116982 | Kernel\_Read\|xilinx:adm-pcie-7v3:1ddr:3.0-0\|cnn\_GOOD | Read | OCL Region | 1 | 1 | 106247 | 106248 | 0.00333333 | 5273.116982 | 5273.116986 |
| 5273.117049 | Kernel\_Read\|xilinx:adm-pcie-7v3:1ddr:3.0-0\|cnn\_GOOD | Read | OCL Region | 1 | 1 | 106267 | 106268 | 0.00333333 | 5273.117049 | 5273.117052 |
| 5273.117122 | Kernel\_Read\|xilinx:adm-pcie-7v3:1ddr:3.0-0\|cnn\_GOOD | Read | OCL Region | 1 | 1 | 106289 | 106290 | 0.00333333 | 5273.117122 | 5273.117126 |
| 5273.117182 | Kernel\_Read\|xilinx:adm-pcie-7v3:1ddr:3.0-0\|cnn\_GOOD | Read | OCL Region | 1 | 1 | 106307 | 106308 | 0.00333333 | 5273.117182 | 5273.117186 |
| 5273.117249 | Kernel\_Read\|xilinx:adm-pcie-7v3:1ddr:3.0-0\|cnn\_GOOD | Read | OCL Region | 1 | 1 | 106327 | 106328 | 0.00333333 | 5273.117249 | 5273.117252 |
| 5273.117322 | Kernel\_Read\|xilinx:adm-pcie-7v3:1ddr:3.0-0\|cnn\_GOOD | Read | OCL Region | 1 | 1 | 106349 | 106350 | 0.00333333 | 5273.117322 | 5273.117326 |
| 5273.117382 | Kernel\_Read\|xilinx:adm-pcie-7v3:1ddr:3.0-0\|cnn\_GOOD | Read | OCL Region | 1 | 1 | 106367 | 106368 | 0.00333333 | 5273.117382 | 5273.117386 |
| 5273.117449 | Kernel\_Read\|xilinx:adm-pcie-7v3:1ddr:3.0-0\|cnn\_GOOD | Read | OCL Region | 1 | 1 | 106387 | 106388 | 0.00333333 | 5273.117449 | 5273.117452 |
| 5273.117522 | Kernel\_Read\|xilinx:adm-pcie-7v3:1ddr:3.0-0\|cnn\_GOOD | Read | OCL Region | 1 | 1 | 106409 | 106410 | 0.00333333 | 5273.117522 | 5273.117526 |
| 5273.117582 | Kernel\_Read\|xilinx:adm-pcie-7v3:1ddr:3.0-0\|cnn\_GOOD | Read | OCL Region | 1 | 1 | 106427 | 106428 | 0.00333333 | 5273.117582 | 5273.117586 |
| 5273.117659 | Kernel\_Read\|xilinx:adm-pcie-7v3:1ddr:3.0-0\|cnn\_GOOD | Read | OCL Region | 1 | 1 | 106450 | 106451 | 0.00333333 | 5273.117659 | 5273.117662 |
| 5273.117722 | Kernel\_Read\|xilinx:adm-pcie-7v3:1ddr:3.0-0\|cnn\_GOOD | Read | OCL Region | 1 | 1 | 106469 | 106470 | 0.00333333 | 5273.117722 | 5273.117726 |
| 5273.117782 | Kernel\_Read\|xilinx:adm-pcie-7v3:1ddr:3.0-0\|cnn\_GOOD | Read | OCL Region | 1 | 1 | 106487 | 106488 | 0.00333333 | 5273.117782 | 5273.117786 |
| 5273.117849 | Kernel\_Read\|xilinx:adm-pcie-7v3:1ddr:3.0-0\|cnn\_GOOD | Read | OCL Region | 1 | 1 | 106507 | 106508 | 0.00333333 | 5273.117849 | 5273.117852 |
| 5273.117922 | Kernel\_Read\|xilinx:adm-pcie-7v3:1ddr:3.0-0\|cnn\_GOOD | Read | OCL Region | 1 | 1 | 106529 | 106530 | 0.00333333 | 5273.117922 | 5273.117926 |
| 5273.118199 | Kernel\_Read\|xilinx:adm-pcie-7v3:1ddr:3.0-0\|cnn\_GOOD | Read | OCL Region | 1 | 1 | 106612 | 106613 | 0.00333333 | 5273.118199 | 5273.118202 |
| 5273.118206 | Kernel\_Read\|xilinx:adm-pcie-7v3:1ddr:3.0-0\|cnn\_GOOD | Read | OCL Region | 1 | 1 | 106614 | 106615 | 0.00333333 | 5273.118206 | 5273.118209 |
| 5273.118209 | Kernel\_Read\|xilinx:adm-pcie-7v3:1ddr:3.0-0\|cnn\_GOOD | Read | OCL Region | 1 | 1 | 106615 | 106616 | 0.00333333 | 5273.118209 | 5273.118212 |
| 5273.118212 | Kernel\_Read\|xilinx:adm-pcie-7v3:1ddr:3.0-0\|cnn\_GOOD | Read | OCL Region | 1 | 1 | 106616 | 106617 | 0.00333333 | 5273.118212 | 5273.118216 |
| 5273.118232 | Kernel\_Read\|xilinx:adm-pcie-7v3:1ddr:3.0-0\|cnn\_GOOD | Read | OCL Region | 1 | 1 | 106622 | 106623 | 0.00333333 | 5273.118232 | 5273.118236 |
| 5273.118322 | Kernel\_Read\|xilinx:adm-pcie-7v3:1ddr:3.0-0\|cnn\_GOOD | Read | OCL Region | 1 | 1 | 106649 | 106650 | 0.00333333 | 5273.118322 | 5273.118326 |
| 5273.118382 | Kernel\_Read\|xilinx:adm-pcie-7v3:1ddr:3.0-0\|cnn\_GOOD | Read | OCL Region | 1 | 1 | 106667 | 106668 | 0.00333333 | 5273.118382 | 5273.118386 |
| 5273.118449 | Kernel\_Read\|xilinx:adm-pcie-7v3:1ddr:3.0-0\|cnn\_GOOD | Read | OCL Region | 1 | 1 | 106687 | 106688 | 0.00333333 | 5273.118449 | 5273.118452 |
| 5273.118522 | Kernel\_Read\|xilinx:adm-pcie-7v3:1ddr:3.0-0\|cnn\_GOOD | Read | OCL Region | 1 | 1 | 106709 | 106710 | 0.00333333 | 5273.118522 | 5273.118526 |
| 5273.118582 | Kernel\_Read\|xilinx:adm-pcie-7v3:1ddr:3.0-0\|cnn\_GOOD | Read | OCL Region | 1 | 1 | 106727 | 106728 | 0.00333333 | 5273.118582 | 5273.118586 |
| 5273.118649 | Kernel\_Read\|xilinx:adm-pcie-7v3:1ddr:3.0-0\|cnn\_GOOD | Read | OCL Region | 1 | 1 | 106747 | 106748 | 0.00333333 | 5273.118649 | 5273.118652 |
| 5273.118722 | Kernel\_Read\|xilinx:adm-pcie-7v3:1ddr:3.0-0\|cnn\_GOOD | Read | OCL Region | 1 | 1 | 106769 | 106770 | 0.00333333 | 5273.118722 | 5273.118726 |
| 5273.118782 | Kernel\_Read\|xilinx:adm-pcie-7v3:1ddr:3.0-0\|cnn\_GOOD | Read | OCL Region | 1 | 1 | 106787 | 106788 | 0.00333333 | 5273.118782 | 5273.118786 |
| 5273.118849 | Kernel\_Read\|xilinx:adm-pcie-7v3:1ddr:3.0-0\|cnn\_GOOD | Read | OCL Region | 1 | 1 | 106807 | 106808 | 0.00333333 | 5273.118849 | 5273.118852 |
| 5273.118922 | Kernel\_Read\|xilinx:adm-pcie-7v3:1ddr:3.0-0\|cnn\_GOOD | Read | OCL Region | 1 | 1 | 106829 | 106830 | 0.00333333 | 5273.118922 | 5273.118926 |
| 5273.118982 | Kernel\_Read\|xilinx:adm-pcie-7v3:1ddr:3.0-0\|cnn\_GOOD | Read | OCL Region | 1 | 1 | 106847 | 106848 | 0.00333333 | 5273.118982 | 5273.118986 |
| 5273.119049 | Kernel\_Read\|xilinx:adm-pcie-7v3:1ddr:3.0-0\|cnn\_GOOD | Read | OCL Region | 1 | 1 | 106867 | 106868 | 0.00333333 | 5273.119049 | 5273.119052 |
| 5273.119122 | Kernel\_Read\|xilinx:adm-pcie-7v3:1ddr:3.0-0\|cnn\_GOOD | Read | OCL Region | 1 | 1 | 106889 | 106890 | 0.00333333 | 5273.119122 | 5273.119126 |
| 5273.119182 | Kernel\_Read\|xilinx:adm-pcie-7v3:1ddr:3.0-0\|cnn\_GOOD | Read | OCL Region | 1 | 1 | 106907 | 106908 | 0.00333333 | 5273.119182 | 5273.119186 |
| 5273.119249 | Kernel\_Read\|xilinx:adm-pcie-7v3:1ddr:3.0-0\|cnn\_GOOD | Read | OCL Region | 1 | 1 | 106927 | 106928 | 0.00333333 | 5273.119249 | 5273.119252 |
| 5273.119322 | Kernel\_Read\|xilinx:adm-pcie-7v3:1ddr:3.0-0\|cnn\_GOOD | Read | OCL Region | 1 | 1 | 106949 | 106950 | 0.00333333 | 5273.119322 | 5273.119326 |
| 5273.119382 | Kernel\_Read\|xilinx:adm-pcie-7v3:1ddr:3.0-0\|cnn\_GOOD | Read | OCL Region | 1 | 1 | 106967 | 106968 | 0.00333333 | 5273.119382 | 5273.119386 |
| 5273.119449 | Kernel\_Read\|xilinx:adm-pcie-7v3:1ddr:3.0-0\|cnn\_GOOD | Read | OCL Region | 1 | 1 | 106987 | 106988 | 0.00333333 | 5273.119449 | 5273.119452 |
| 5273.119522 | Kernel\_Read\|xilinx:adm-pcie-7v3:1ddr:3.0-0\|cnn\_GOOD | Read | OCL Region | 1 | 1 | 107009 | 107010 | 0.00333333 | 5273.119522 | 5273.119526 |
| 5273.119582 | Kernel\_Read\|xilinx:adm-pcie-7v3:1ddr:3.0-0\|cnn\_GOOD | Read | OCL Region | 1 | 1 | 107027 | 107028 | 0.00333333 | 5273.119582 | 5273.119586 |
| 5273.119649 | Kernel\_Read\|xilinx:adm-pcie-7v3:1ddr:3.0-0\|cnn\_GOOD | Read | OCL Region | 1 | 1 | 107047 | 107048 | 0.00333333 | 5273.119649 | 5273.119652 |
| 5273.119722 | Kernel\_Read\|xilinx:adm-pcie-7v3:1ddr:3.0-0\|cnn\_GOOD | Read | OCL Region | 1 | 1 | 107069 | 107070 | 0.00333333 | 5273.119722 | 5273.119726 |
| 5273.119782 | Kernel\_Read\|xilinx:adm-pcie-7v3:1ddr:3.0-0\|cnn\_GOOD | Read | OCL Region | 1 | 1 | 107087 | 107088 | 0.00333333 | 5273.119782 | 5273.119786 |
| 5273.119849 | Kernel\_Read\|xilinx:adm-pcie-7v3:1ddr:3.0-0\|cnn\_GOOD | Read | OCL Region | 1 | 1 | 107107 | 107108 | 0.00333333 | 5273.119849 | 5273.119852 |
| 5273.119922 | Kernel\_Read\|xilinx:adm-pcie-7v3:1ddr:3.0-0\|cnn\_GOOD | Read | OCL Region | 1 | 1 | 107129 | 107130 | 0.00333333 | 5273.119922 | 5273.119926 |
| 5273.119982 | Kernel\_Read\|xilinx:adm-pcie-7v3:1ddr:3.0-0\|cnn\_GOOD | Read | OCL Region | 1 | 1 | 107147 | 107148 | 0.00333333 | 5273.119982 | 5273.119986 |
| 5273.120049 | Kernel\_Read\|xilinx:adm-pcie-7v3:1ddr:3.0-0\|cnn\_GOOD | Read | OCL Region | 1 | 1 | 107167 | 107168 | 0.00333333 | 5273.120049 | 5273.120052 |
| 5273.120122 | Kernel\_Read\|xilinx:adm-pcie-7v3:1ddr:3.0-0\|cnn\_GOOD | Read | OCL Region | 1 | 1 | 107189 | 107190 | 0.00333333 | 5273.120122 | 5273.120126 |
| 5273.120182 | Kernel\_Read\|xilinx:adm-pcie-7v3:1ddr:3.0-0\|cnn\_GOOD | Read | OCL Region | 1 | 1 | 107207 | 107208 | 0.00333333 | 5273.120182 | 5273.120186 |
| 5273.120249 | Kernel\_Read\|xilinx:adm-pcie-7v3:1ddr:3.0-0\|cnn\_GOOD | Read | OCL Region | 1 | 1 | 107227 | 107228 | 0.00333333 | 5273.120249 | 5273.120252 |
| 5273.120322 | Kernel\_Read\|xilinx:adm-pcie-7v3:1ddr:3.0-0\|cnn\_GOOD | Read | OCL Region | 1 | 1 | 107249 | 107250 | 0.00333333 | 5273.120322 | 5273.120326 |
| 5273.120382 | Kernel\_Read\|xilinx:adm-pcie-7v3:1ddr:3.0-0\|cnn\_GOOD | Read | OCL Region | 1 | 1 | 107267 | 107268 | 0.00333333 | 5273.120382 | 5273.120386 |
| 5273.120449 | Kernel\_Read\|xilinx:adm-pcie-7v3:1ddr:3.0-0\|cnn\_GOOD | Read | OCL Region | 1 | 1 | 107287 | 107288 | 0.00333333 | 5273.120449 | 5273.120452 |
| 5273.120522 | Kernel\_Read\|xilinx:adm-pcie-7v3:1ddr:3.0-0\|cnn\_GOOD | Read | OCL Region | 1 | 1 | 107309 | 107310 | 0.00333333 | 5273.120522 | 5273.120526 |
| 5273.120582 | Kernel\_Read\|xilinx:adm-pcie-7v3:1ddr:3.0-0\|cnn\_GOOD | Read | OCL Region | 1 | 1 | 107327 | 107328 | 0.00333333 | 5273.120582 | 5273.120586 |
| 5273.120662 | Kernel\_Read\|xilinx:adm-pcie-7v3:1ddr:3.0-0\|cnn\_GOOD | Read | OCL Region | 1 | 1 | 107351 | 107352 | 0.00333333 | 5273.120662 | 5273.120666 |
| 5273.120722 | Kernel\_Read\|xilinx:adm-pcie-7v3:1ddr:3.0-0\|cnn\_GOOD | Read | OCL Region | 1 | 1 | 107369 | 107370 | 0.00333333 | 5273.120722 | 5273.120726 |
| 5273.120782 | Kernel\_Read\|xilinx:adm-pcie-7v3:1ddr:3.0-0\|cnn\_GOOD | Read | OCL Region | 1 | 1 | 107387 | 107388 | 0.00333333 | 5273.120782 | 5273.120786 |
| 5273.120849 | Kernel\_Read\|xilinx:adm-pcie-7v3:1ddr:3.0-0\|cnn\_GOOD | Read | OCL Region | 1 | 1 | 107407 | 107408 | 0.00333333 | 5273.120849 | 5273.120852 |
| 5273.120922 | Kernel\_Read\|xilinx:adm-pcie-7v3:1ddr:3.0-0\|cnn\_GOOD | Read | OCL Region | 1 | 1 | 107429 | 107430 | 0.00333333 | 5273.120922 | 5273.120926 |
| 5273.120982 | Kernel\_Read\|xilinx:adm-pcie-7v3:1ddr:3.0-0\|cnn\_GOOD | Read | OCL Region | 1 | 1 | 107447 | 107448 | 0.00333333 | 5273.120982 | 5273.120986 |
| 5273.121049 | Kernel\_Read\|xilinx:adm-pcie-7v3:1ddr:3.0-0\|cnn\_GOOD | Read | OCL Region | 1 | 1 | 107467 | 107468 | 0.00333333 | 5273.121049 | 5273.121052 |
| 5273.121122 | Kernel\_Read\|xilinx:adm-pcie-7v3:1ddr:3.0-0\|cnn\_GOOD | Read | OCL Region | 1 | 1 | 107489 | 107490 | 0.00333333 | 5273.121122 | 5273.121126 |
| 5273.121182 | Kernel\_Read\|xilinx:adm-pcie-7v3:1ddr:3.0-0\|cnn\_GOOD | Read | OCL Region | 1 | 1 | 107507 | 107508 | 0.00333333 | 5273.121182 | 5273.121186 |
| 5273.121249 | Kernel\_Read\|xilinx:adm-pcie-7v3:1ddr:3.0-0\|cnn\_GOOD | Read | OCL Region | 1 | 1 | 107527 | 107528 | 0.00333333 | 5273.121249 | 5273.121252 |
| 5273.121322 | Kernel\_Read\|xilinx:adm-pcie-7v3:1ddr:3.0-0\|cnn\_GOOD | Read | OCL Region | 1 | 1 | 107549 | 107550 | 0.00333333 | 5273.121322 | 5273.121326 |
| 5273.121382 | Kernel\_Read\|xilinx:adm-pcie-7v3:1ddr:3.0-0\|cnn\_GOOD | Read | OCL Region | 1 | 1 | 107567 | 107568 | 0.00333333 | 5273.121382 | 5273.121386 |
| 5273.121456 | Kernel\_Read\|xilinx:adm-pcie-7v3:1ddr:3.0-0\|cnn\_GOOD | Read | OCL Region | 1 | 1 | 107589 | 107590 | 0.00333333 | 5273.121456 | 5273.121459 |
| 5273.121522 | Kernel\_Read\|xilinx:adm-pcie-7v3:1ddr:3.0-0\|cnn\_GOOD | Read | OCL Region | 1 | 1 | 107609 | 107610 | 0.00333333 | 5273.121522 | 5273.121526 |
| 5273.121582 | Kernel\_Read\|xilinx:adm-pcie-7v3:1ddr:3.0-0\|cnn\_GOOD | Read | OCL Region | 1 | 1 | 107627 | 107628 | 0.00333333 | 5273.121582 | 5273.121586 |
| 5273.121649 | Kernel\_Read\|xilinx:adm-pcie-7v3:1ddr:3.0-0\|cnn\_GOOD | Read | OCL Region | 1 | 1 | 107647 | 107648 | 0.00333333 | 5273.121649 | 5273.121652 |
| 5273.121722 | Kernel\_Read\|xilinx:adm-pcie-7v3:1ddr:3.0-0\|cnn\_GOOD | Read | OCL Region | 1 | 1 | 107669 | 107670 | 0.00333333 | 5273.121722 | 5273.121726 |
| 5273.121782 | Kernel\_Read\|xilinx:adm-pcie-7v3:1ddr:3.0-0\|cnn\_GOOD | Read | OCL Region | 1 | 1 | 107687 | 107688 | 0.00333333 | 5273.121782 | 5273.121786 |
| 5273.121849 | Kernel\_Read\|xilinx:adm-pcie-7v3:1ddr:3.0-0\|cnn\_GOOD | Read | OCL Region | 1 | 1 | 107707 | 107708 | 0.00333333 | 5273.121849 | 5273.121852 |
| 5273.121922 | Kernel\_Read\|xilinx:adm-pcie-7v3:1ddr:3.0-0\|cnn\_GOOD | Read | OCL Region | 1 | 1 | 107729 | 107730 | 0.00333333 | 5273.121922 | 5273.121926 |
| 5273.121982 | Kernel\_Read\|xilinx:adm-pcie-7v3:1ddr:3.0-0\|cnn\_GOOD | Read | OCL Region | 1 | 1 | 107747 | 107748 | 0.00333333 | 5273.121982 | 5273.121986 |
| 5273.122049 | Kernel\_Read\|xilinx:adm-pcie-7v3:1ddr:3.0-0\|cnn\_GOOD | Read | OCL Region | 1 | 1 | 107767 | 107768 | 0.00333333 | 5273.122049 | 5273.122052 |
| 5273.122122 | Kernel\_Read\|xilinx:adm-pcie-7v3:1ddr:3.0-0\|cnn\_GOOD | Read | OCL Region | 1 | 1 | 107789 | 107790 | 0.00333333 | 5273.122122 | 5273.122126 |
| 5273.122182 | Kernel\_Read\|xilinx:adm-pcie-7v3:1ddr:3.0-0\|cnn\_GOOD | Read | OCL Region | 1 | 1 | 107807 | 107808 | 0.00333333 | 5273.122182 | 5273.122186 |
| 5273.122249 | Kernel\_Read\|xilinx:adm-pcie-7v3:1ddr:3.0-0\|cnn\_GOOD | Read | OCL Region | 1 | 1 | 107827 | 107828 | 0.00333333 | 5273.122249 | 5273.122252 |
| 5273.122322 | Kernel\_Read\|xilinx:adm-pcie-7v3:1ddr:3.0-0\|cnn\_GOOD | Read | OCL Region | 1 | 1 | 107849 | 107850 | 0.00333333 | 5273.122322 | 5273.122326 |
| 5273.122382 | Kernel\_Read\|xilinx:adm-pcie-7v3:1ddr:3.0-0\|cnn\_GOOD | Read | OCL Region | 1 | 1 | 107867 | 107868 | 0.00333333 | 5273.122382 | 5273.122386 |
| 5273.122449 | Kernel\_Read\|xilinx:adm-pcie-7v3:1ddr:3.0-0\|cnn\_GOOD | Read | OCL Region | 1 | 1 | 107887 | 107888 | 0.00333333 | 5273.122449 | 5273.122452 |
| 5273.122522 | Kernel\_Read\|xilinx:adm-pcie-7v3:1ddr:3.0-0\|cnn\_GOOD | Read | OCL Region | 1 | 1 | 107909 | 107910 | 0.00333333 | 5273.122522 | 5273.122526 |
| 5273.122582 | Kernel\_Read\|xilinx:adm-pcie-7v3:1ddr:3.0-0\|cnn\_GOOD | Read | OCL Region | 1 | 1 | 107927 | 107928 | 0.00333333 | 5273.122582 | 5273.122586 |
| 5273.122649 | Kernel\_Read\|xilinx:adm-pcie-7v3:1ddr:3.0-0\|cnn\_GOOD | Read | OCL Region | 1 | 1 | 107947 | 107948 | 0.00333333 | 5273.122649 | 5273.122652 |
| 5273.122722 | Kernel\_Read\|xilinx:adm-pcie-7v3:1ddr:3.0-0\|cnn\_GOOD | Read | OCL Region | 1 | 1 | 107969 | 107970 | 0.00333333 | 5273.122722 | 5273.122726 |
| 5273.122782 | Kernel\_Read\|xilinx:adm-pcie-7v3:1ddr:3.0-0\|cnn\_GOOD | Read | OCL Region | 1 | 1 | 107987 | 107988 | 0.00333333 | 5273.122782 | 5273.122786 |
| 5273.122849 | Kernel\_Read\|xilinx:adm-pcie-7v3:1ddr:3.0-0\|cnn\_GOOD | Read | OCL Region | 1 | 1 | 108007 | 108008 | 0.00333333 | 5273.122849 | 5273.122852 |
| 5273.122922 | Kernel\_Read\|xilinx:adm-pcie-7v3:1ddr:3.0-0\|cnn\_GOOD | Read | OCL Region | 1 | 1 | 108029 | 108030 | 0.00333333 | 5273.122922 | 5273.122926 |
| 5273.122982 | Kernel\_Read\|xilinx:adm-pcie-7v3:1ddr:3.0-0\|cnn\_GOOD | Read | OCL Region | 1 | 1 | 108047 | 108048 | 0.00333333 | 5273.122982 | 5273.122986 |
| 5273.123049 | Kernel\_Read\|xilinx:adm-pcie-7v3:1ddr:3.0-0\|cnn\_GOOD | Read | OCL Region | 1 | 1 | 108067 | 108068 | 0.00333333 | 5273.123049 | 5273.123052 |
| 5273.123112 | Kernel\_Read\|xilinx:adm-pcie-7v3:1ddr:3.0-0\|cnn\_GOOD | Read | OCL Region | 1 | 1 | 108086 | 108087 | 0.00333333 | 5273.123112 | 5273.123116 |
| 5273.123182 | Kernel\_Read\|xilinx:adm-pcie-7v3:1ddr:3.0-0\|cnn\_GOOD | Read | OCL Region | 1 | 1 | 108107 | 108108 | 0.00333333 | 5273.123182 | 5273.123186 |
| 5273.123249 | Kernel\_Read\|xilinx:adm-pcie-7v3:1ddr:3.0-0\|cnn\_GOOD | Read | OCL Region | 1 | 1 | 108127 | 108128 | 0.00333333 | 5273.123249 | 5273.123252 |
| 5273.123322 | Kernel\_Read\|xilinx:adm-pcie-7v3:1ddr:3.0-0\|cnn\_GOOD | Read | OCL Region | 1 | 1 | 108149 | 108150 | 0.00333333 | 5273.123322 | 5273.123326 |
| 5273.123382 | Kernel\_Read\|xilinx:adm-pcie-7v3:1ddr:3.0-0\|cnn\_GOOD | Read | OCL Region | 1 | 1 | 108167 | 108168 | 0.00333333 | 5273.123382 | 5273.123386 |
| 5273.123449 | Kernel\_Read\|xilinx:adm-pcie-7v3:1ddr:3.0-0\|cnn\_GOOD | Read | OCL Region | 1 | 1 | 108187 | 108188 | 0.00333333 | 5273.123449 | 5273.123452 |
| 5273.123522 | Kernel\_Read\|xilinx:adm-pcie-7v3:1ddr:3.0-0\|cnn\_GOOD | Read | OCL Region | 1 | 1 | 108209 | 108210 | 0.00333333 | 5273.123522 | 5273.123526 |
| 5273.123582 | Kernel\_Read\|xilinx:adm-pcie-7v3:1ddr:3.0-0\|cnn\_GOOD | Read | OCL Region | 1 | 1 | 108227 | 108228 | 0.00333333 | 5273.123582 | 5273.123586 |
| 5273.123649 | Kernel\_Read\|xilinx:adm-pcie-7v3:1ddr:3.0-0\|cnn\_GOOD | Read | OCL Region | 1 | 1 | 108247 | 108248 | 0.00333333 | 5273.123649 | 5273.123652 |
| 5273.123722 | Kernel\_Read\|xilinx:adm-pcie-7v3:1ddr:3.0-0\|cnn\_GOOD | Read | OCL Region | 1 | 1 | 108269 | 108270 | 0.00333333 | 5273.123722 | 5273.123726 |
| 5273.123782 | Kernel\_Read\|xilinx:adm-pcie-7v3:1ddr:3.0-0\|cnn\_GOOD | Read | OCL Region | 1 | 1 | 108287 | 108288 | 0.00333333 | 5273.123782 | 5273.123786 |
| 5273.123849 | Kernel\_Read\|xilinx:adm-pcie-7v3:1ddr:3.0-0\|cnn\_GOOD | Read | OCL Region | 1 | 1 | 108307 | 108308 | 0.00333333 | 5273.123849 | 5273.123852 |
| 5273.123929 | Kernel\_Read\|xilinx:adm-pcie-7v3:1ddr:3.0-0\|cnn\_GOOD | Read | OCL Region | 1 | 1 | 108331 | 108332 | 0.00333333 | 5273.123929 | 5273.123932 |
| 5273.123982 | Kernel\_Read\|xilinx:adm-pcie-7v3:1ddr:3.0-0\|cnn\_GOOD | Read | OCL Region | 1 | 1 | 108347 | 108348 | 0.00333333 | 5273.123982 | 5273.123986 |
| 5273.124049 | Kernel\_Read\|xilinx:adm-pcie-7v3:1ddr:3.0-0\|cnn\_GOOD | Read | OCL Region | 1 | 1 | 108367 | 108368 | 0.00333333 | 5273.124049 | 5273.124052 |
| 5273.124122 | Kernel\_Read\|xilinx:adm-pcie-7v3:1ddr:3.0-0\|cnn\_GOOD | Read | OCL Region | 1 | 1 | 108389 | 108390 | 0.00333333 | 5273.124122 | 5273.124126 |
| 5273.124182 | Kernel\_Read\|xilinx:adm-pcie-7v3:1ddr:3.0-0\|cnn\_GOOD | Read | OCL Region | 1 | 1 | 108407 | 108408 | 0.00333333 | 5273.124182 | 5273.124186 |
| 5273.124472 | Kernel\_Read\|xilinx:adm-pcie-7v3:1ddr:3.0-0\|cnn\_GOOD | Read | OCL Region | 1 | 1 | 108494 | 108495 | 0.00333333 | 5273.124472 | 5273.124476 |
| 5273.124476 | Kernel\_Read\|xilinx:adm-pcie-7v3:1ddr:3.0-0\|cnn\_GOOD | Read | OCL Region | 1 | 1 | 108495 | 108496 | 0.00333333 | 5273.124476 | 5273.124479 |
| 5273.124479 | Kernel\_Read\|xilinx:adm-pcie-7v3:1ddr:3.0-0\|cnn\_GOOD | Read | OCL Region | 1 | 1 | 108496 | 108497 | 0.00333333 | 5273.124479 | 5273.124482 |
| 5273.124482 | Kernel\_Read\|xilinx:adm-pcie-7v3:1ddr:3.0-0\|cnn\_GOOD | Read | OCL Region | 1 | 1 | 108497 | 108498 | 0.00333333 | 5273.124482 | 5273.124486 |
| 5273.124506 | Kernel\_Read\|xilinx:adm-pcie-7v3:1ddr:3.0-0\|cnn\_GOOD | Read | OCL Region | 1 | 1 | 108504 | 108505 | 0.00333333 | 5273.124506 | 5273.124509 |
| 5273.124582 | Kernel\_Read\|xilinx:adm-pcie-7v3:1ddr:3.0-0\|cnn\_GOOD | Read | OCL Region | 1 | 1 | 108527 | 108528 | 0.00333333 | 5273.124582 | 5273.124586 |
| 5273.124649 | Kernel\_Read\|xilinx:adm-pcie-7v3:1ddr:3.0-0\|cnn\_GOOD | Read | OCL Region | 1 | 1 | 108547 | 108548 | 0.00333333 | 5273.124649 | 5273.124652 |
| 5273.124722 | Kernel\_Read\|xilinx:adm-pcie-7v3:1ddr:3.0-0\|cnn\_GOOD | Read | OCL Region | 1 | 1 | 108569 | 108570 | 0.00333333 | 5273.124722 | 5273.124726 |
| 5273.124782 | Kernel\_Read\|xilinx:adm-pcie-7v3:1ddr:3.0-0\|cnn\_GOOD | Read | OCL Region | 1 | 1 | 108587 | 108588 | 0.00333333 | 5273.124782 | 5273.124786 |
| 5273.124849 | Kernel\_Read\|xilinx:adm-pcie-7v3:1ddr:3.0-0\|cnn\_GOOD | Read | OCL Region | 1 | 1 | 108607 | 108608 | 0.00333333 | 5273.124849 | 5273.124852 |
| 5273.124922 | Kernel\_Read\|xilinx:adm-pcie-7v3:1ddr:3.0-0\|cnn\_GOOD | Read | OCL Region | 1 | 1 | 108629 | 108630 | 0.00333333 | 5273.124922 | 5273.124926 |
| 5273.124982 | Kernel\_Read\|xilinx:adm-pcie-7v3:1ddr:3.0-0\|cnn\_GOOD | Read | OCL Region | 1 | 1 | 108647 | 108648 | 0.00333333 | 5273.124982 | 5273.124986 |
| 5273.125049 | Kernel\_Read\|xilinx:adm-pcie-7v3:1ddr:3.0-0\|cnn\_GOOD | Read | OCL Region | 1 | 1 | 108667 | 108668 | 0.00333333 | 5273.125049 | 5273.125052 |
| 5273.125122 | Kernel\_Read\|xilinx:adm-pcie-7v3:1ddr:3.0-0\|cnn\_GOOD | Read | OCL Region | 1 | 1 | 108689 | 108690 | 0.00333333 | 5273.125122 | 5273.125126 |
| 5273.125182 | Kernel\_Read\|xilinx:adm-pcie-7v3:1ddr:3.0-0\|cnn\_GOOD | Read | OCL Region | 1 | 1 | 108707 | 108708 | 0.00333333 | 5273.125182 | 5273.125186 |
| 5273.125249 | Kernel\_Read\|xilinx:adm-pcie-7v3:1ddr:3.0-0\|cnn\_GOOD | Read | OCL Region | 1 | 1 | 108727 | 108728 | 0.00333333 | 5273.125249 | 5273.125252 |
| 5273.125322 | Kernel\_Read\|xilinx:adm-pcie-7v3:1ddr:3.0-0\|cnn\_GOOD | Read | OCL Region | 1 | 1 | 108749 | 108750 | 0.00333333 | 5273.125322 | 5273.125326 |
| 5273.125382 | Kernel\_Read\|xilinx:adm-pcie-7v3:1ddr:3.0-0\|cnn\_GOOD | Read | OCL Region | 1 | 1 | 108767 | 108768 | 0.00333333 | 5273.125382 | 5273.125386 |
| 5273.125449 | Kernel\_Read\|xilinx:adm-pcie-7v3:1ddr:3.0-0\|cnn\_GOOD | Read | OCL Region | 1 | 1 | 108787 | 108788 | 0.00333333 | 5273.125449 | 5273.125452 |
| 5273.125522 | Kernel\_Read\|xilinx:adm-pcie-7v3:1ddr:3.0-0\|cnn\_GOOD | Read | OCL Region | 1 | 1 | 108809 | 108810 | 0.00333333 | 5273.125522 | 5273.125526 |
| 5273.125582 | Kernel\_Read\|xilinx:adm-pcie-7v3:1ddr:3.0-0\|cnn\_GOOD | Read | OCL Region | 1 | 1 | 108827 | 108828 | 0.00333333 | 5273.125582 | 5273.125586 |
| 5273.125649 | Kernel\_Read\|xilinx:adm-pcie-7v3:1ddr:3.0-0\|cnn\_GOOD | Read | OCL Region | 1 | 1 | 108847 | 108848 | 0.00333333 | 5273.125649 | 5273.125652 |
| 5273.125722 | Kernel\_Read\|xilinx:adm-pcie-7v3:1ddr:3.0-0\|cnn\_GOOD | Read | OCL Region | 1 | 1 | 108869 | 108870 | 0.00333333 | 5273.125722 | 5273.125726 |
| 5273.125782 | Kernel\_Read\|xilinx:adm-pcie-7v3:1ddr:3.0-0\|cnn\_GOOD | Read | OCL Region | 1 | 1 | 108887 | 108888 | 0.00333333 | 5273.125782 | 5273.125786 |
| 5273.125849 | Kernel\_Read\|xilinx:adm-pcie-7v3:1ddr:3.0-0\|cnn\_GOOD | Read | OCL Region | 1 | 1 | 108907 | 108908 | 0.00333333 | 5273.125849 | 5273.125852 |
| 5273.125922 | Kernel\_Read\|xilinx:adm-pcie-7v3:1ddr:3.0-0\|cnn\_GOOD | Read | OCL Region | 1 | 1 | 108929 | 108930 | 0.00333333 | 5273.125922 | 5273.125926 |
| 5273.125982 | Kernel\_Read\|xilinx:adm-pcie-7v3:1ddr:3.0-0\|cnn\_GOOD | Read | OCL Region | 1 | 1 | 108947 | 108948 | 0.00333333 | 5273.125982 | 5273.125986 |
| 5273.126049 | Kernel\_Read\|xilinx:adm-pcie-7v3:1ddr:3.0-0\|cnn\_GOOD | Read | OCL Region | 1 | 1 | 108967 | 108968 | 0.00333333 | 5273.126049 | 5273.126052 |
| 5273.126122 | Kernel\_Read\|xilinx:adm-pcie-7v3:1ddr:3.0-0\|cnn\_GOOD | Read | OCL Region | 1 | 1 | 108989 | 108990 | 0.00333333 | 5273.126122 | 5273.126126 |
| 5273.126182 | Kernel\_Read\|xilinx:adm-pcie-7v3:1ddr:3.0-0\|cnn\_GOOD | Read | OCL Region | 1 | 1 | 109007 | 109008 | 0.00333333 | 5273.126182 | 5273.126186 |
| 5273.126249 | Kernel\_Read\|xilinx:adm-pcie-7v3:1ddr:3.0-0\|cnn\_GOOD | Read | OCL Region | 1 | 1 | 109027 | 109028 | 0.00333333 | 5273.126249 | 5273.126252 |
| 5273.126322 | Kernel\_Read\|xilinx:adm-pcie-7v3:1ddr:3.0-0\|cnn\_GOOD | Read | OCL Region | 1 | 1 | 109049 | 109050 | 0.00333333 | 5273.126322 | 5273.126326 |
| 5273.126382 | Kernel\_Read\|xilinx:adm-pcie-7v3:1ddr:3.0-0\|cnn\_GOOD | Read | OCL Region | 1 | 1 | 109067 | 109068 | 0.00333333 | 5273.126382 | 5273.126386 |
| 5273.126449 | Kernel\_Read\|xilinx:adm-pcie-7v3:1ddr:3.0-0\|cnn\_GOOD | Read | OCL Region | 1 | 1 | 109087 | 109088 | 0.00333333 | 5273.126449 | 5273.126452 |
| 5273.126522 | Kernel\_Read\|xilinx:adm-pcie-7v3:1ddr:3.0-0\|cnn\_GOOD | Read | OCL Region | 1 | 1 | 109109 | 109110 | 0.00333333 | 5273.126522 | 5273.126526 |
| 5273.126582 | Kernel\_Read\|xilinx:adm-pcie-7v3:1ddr:3.0-0\|cnn\_GOOD | Read | OCL Region | 1 | 1 | 109127 | 109128 | 0.00333333 | 5273.126582 | 5273.126586 |
| 5273.126649 | Kernel\_Read\|xilinx:adm-pcie-7v3:1ddr:3.0-0\|cnn\_GOOD | Read | OCL Region | 1 | 1 | 109147 | 109148 | 0.00333333 | 5273.126649 | 5273.126652 |
| 5273.126722 | Kernel\_Read\|xilinx:adm-pcie-7v3:1ddr:3.0-0\|cnn\_GOOD | Read | OCL Region | 1 | 1 | 109169 | 109170 | 0.00333333 | 5273.126722 | 5273.126726 |
| 5273.126782 | Kernel\_Read\|xilinx:adm-pcie-7v3:1ddr:3.0-0\|cnn\_GOOD | Read | OCL Region | 1 | 1 | 109187 | 109188 | 0.00333333 | 5273.126782 | 5273.126786 |
| 5273.126849 | Kernel\_Read\|xilinx:adm-pcie-7v3:1ddr:3.0-0\|cnn\_GOOD | Read | OCL Region | 1 | 1 | 109207 | 109208 | 0.00333333 | 5273.126849 | 5273.126852 |
| 5273.126912 | Kernel\_Read\|xilinx:adm-pcie-7v3:1ddr:3.0-0\|cnn\_GOOD | Read | OCL Region | 1 | 1 | 109226 | 109227 | 0.00333333 | 5273.126912 | 5273.126916 |
| 5273.126982 | Kernel\_Read\|xilinx:adm-pcie-7v3:1ddr:3.0-0\|cnn\_GOOD | Read | OCL Region | 1 | 1 | 109247 | 109248 | 0.00333333 | 5273.126982 | 5273.126986 |
| 5273.127049 | Kernel\_Read\|xilinx:adm-pcie-7v3:1ddr:3.0-0\|cnn\_GOOD | Read | OCL Region | 1 | 1 | 109267 | 109268 | 0.00333333 | 5273.127049 | 5273.127052 |
| 5273.127122 | Kernel\_Read\|xilinx:adm-pcie-7v3:1ddr:3.0-0\|cnn\_GOOD | Read | OCL Region | 1 | 1 | 109289 | 109290 | 0.00333333 | 5273.127122 | 5273.127126 |
| 5273.127182 | Kernel\_Read\|xilinx:adm-pcie-7v3:1ddr:3.0-0\|cnn\_GOOD | Read | OCL Region | 1 | 1 | 109307 | 109308 | 0.00333333 | 5273.127182 | 5273.127186 |
| 5273.127249 | Kernel\_Read\|xilinx:adm-pcie-7v3:1ddr:3.0-0\|cnn\_GOOD | Read | OCL Region | 1 | 1 | 109327 | 109328 | 0.00333333 | 5273.127249 | 5273.127252 |
| 5273.127322 | Kernel\_Read\|xilinx:adm-pcie-7v3:1ddr:3.0-0\|cnn\_GOOD | Read | OCL Region | 1 | 1 | 109349 | 109350 | 0.00333333 | 5273.127322 | 5273.127326 |
| 5273.127382 | Kernel\_Read\|xilinx:adm-pcie-7v3:1ddr:3.0-0\|cnn\_GOOD | Read | OCL Region | 1 | 1 | 109367 | 109368 | 0.00333333 | 5273.127382 | 5273.127386 |
| 5273.127449 | Kernel\_Read\|xilinx:adm-pcie-7v3:1ddr:3.0-0\|cnn\_GOOD | Read | OCL Region | 1 | 1 | 109387 | 109388 | 0.00333333 | 5273.127449 | 5273.127452 |
| 5273.127522 | Kernel\_Read\|xilinx:adm-pcie-7v3:1ddr:3.0-0\|cnn\_GOOD | Read | OCL Region | 1 | 1 | 109409 | 109410 | 0.00333333 | 5273.127522 | 5273.127526 |
| 5273.127582 | Kernel\_Read\|xilinx:adm-pcie-7v3:1ddr:3.0-0\|cnn\_GOOD | Read | OCL Region | 1 | 1 | 109427 | 109428 | 0.00333333 | 5273.127582 | 5273.127586 |
| 5273.127649 | Kernel\_Read\|xilinx:adm-pcie-7v3:1ddr:3.0-0\|cnn\_GOOD | Read | OCL Region | 1 | 1 | 109447 | 109448 | 0.00333333 | 5273.127649 | 5273.127652 |
| 5273.127726 | Kernel\_Read\|xilinx:adm-pcie-7v3:1ddr:3.0-0\|cnn\_GOOD | Read | OCL Region | 1 | 1 | 109470 | 109471 | 0.00333333 | 5273.127726 | 5273.127729 |
| 5273.127782 | Kernel\_Read\|xilinx:adm-pcie-7v3:1ddr:3.0-0\|cnn\_GOOD | Read | OCL Region | 1 | 1 | 109487 | 109488 | 0.00333333 | 5273.127782 | 5273.127786 |
| 5273.127849 | Kernel\_Read\|xilinx:adm-pcie-7v3:1ddr:3.0-0\|cnn\_GOOD | Read | OCL Region | 1 | 1 | 109507 | 109508 | 0.00333333 | 5273.127849 | 5273.127852 |
| 5273.127922 | Kernel\_Read\|xilinx:adm-pcie-7v3:1ddr:3.0-0\|cnn\_GOOD | Read | OCL Region | 1 | 1 | 109529 | 109530 | 0.00333333 | 5273.127922 | 5273.127926 |
| 5273.127982 | Kernel\_Read\|xilinx:adm-pcie-7v3:1ddr:3.0-0\|cnn\_GOOD | Read | OCL Region | 1 | 1 | 109547 | 109548 | 0.00333333 | 5273.127982 | 5273.127986 |
| 5273.128049 | Kernel\_Read\|xilinx:adm-pcie-7v3:1ddr:3.0-0\|cnn\_GOOD | Read | OCL Region | 1 | 1 | 109567 | 109568 | 0.00333333 | 5273.128049 | 5273.128052 |
| 5273.128122 | Kernel\_Read\|xilinx:adm-pcie-7v3:1ddr:3.0-0\|cnn\_GOOD | Read | OCL Region | 1 | 1 | 109589 | 109590 | 0.00333333 | 5273.128122 | 5273.128126 |
| 5273.128182 | Kernel\_Read\|xilinx:adm-pcie-7v3:1ddr:3.0-0\|cnn\_GOOD | Read | OCL Region | 1 | 1 | 109607 | 109608 | 0.00333333 | 5273.128182 | 5273.128186 |
| 5273.128249 | Kernel\_Read\|xilinx:adm-pcie-7v3:1ddr:3.0-0\|cnn\_GOOD | Read | OCL Region | 1 | 1 | 109627 | 109628 | 0.00333333 | 5273.128249 | 5273.128252 |
| 5273.128322 | Kernel\_Read\|xilinx:adm-pcie-7v3:1ddr:3.0-0\|cnn\_GOOD | Read | OCL Region | 1 | 1 | 109649 | 109650 | 0.00333333 | 5273.128322 | 5273.128326 |
| 5273.128382 | Kernel\_Read\|xilinx:adm-pcie-7v3:1ddr:3.0-0\|cnn\_GOOD | Read | OCL Region | 1 | 1 | 109667 | 109668 | 0.00333333 | 5273.128382 | 5273.128386 |
| 5273.128449 | Kernel\_Read\|xilinx:adm-pcie-7v3:1ddr:3.0-0\|cnn\_GOOD | Read | OCL Region | 1 | 1 | 109687 | 109688 | 0.00333333 | 5273.128449 | 5273.128452 |
| 5273.128522 | Kernel\_Read\|xilinx:adm-pcie-7v3:1ddr:3.0-0\|cnn\_GOOD | Read | OCL Region | 1 | 1 | 109709 | 109710 | 0.00333333 | 5273.128522 | 5273.128526 |
| 5273.128582 | Kernel\_Read\|xilinx:adm-pcie-7v3:1ddr:3.0-0\|cnn\_GOOD | Read | OCL Region | 1 | 1 | 109727 | 109728 | 0.00333333 | 5273.128582 | 5273.128586 |
| 5273.128649 | Kernel\_Read\|xilinx:adm-pcie-7v3:1ddr:3.0-0\|cnn\_GOOD | Read | OCL Region | 1 | 1 | 109747 | 109748 | 0.00333333 | 5273.128649 | 5273.128652 |
| 5273.128722 | Kernel\_Read\|xilinx:adm-pcie-7v3:1ddr:3.0-0\|cnn\_GOOD | Read | OCL Region | 1 | 1 | 109769 | 109770 | 0.00333333 | 5273.128722 | 5273.128726 |
| 5273.128782 | Kernel\_Read\|xilinx:adm-pcie-7v3:1ddr:3.0-0\|cnn\_GOOD | Read | OCL Region | 1 | 1 | 109787 | 109788 | 0.00333333 | 5273.128782 | 5273.128786 |
| 5273.128849 | Kernel\_Read\|xilinx:adm-pcie-7v3:1ddr:3.0-0\|cnn\_GOOD | Read | OCL Region | 1 | 1 | 109807 | 109808 | 0.00333333 | 5273.128849 | 5273.128852 |
| 5273.128922 | Kernel\_Read\|xilinx:adm-pcie-7v3:1ddr:3.0-0\|cnn\_GOOD | Read | OCL Region | 1 | 1 | 109829 | 109830 | 0.00333333 | 5273.128922 | 5273.128926 |
| 5273.128982 | Kernel\_Read\|xilinx:adm-pcie-7v3:1ddr:3.0-0\|cnn\_GOOD | Read | OCL Region | 1 | 1 | 109847 | 109848 | 0.00333333 | 5273.128982 | 5273.128986 |
| 5273.129049 | Kernel\_Read\|xilinx:adm-pcie-7v3:1ddr:3.0-0\|cnn\_GOOD | Read | OCL Region | 1 | 1 | 109867 | 109868 | 0.00333333 | 5273.129049 | 5273.129052 |
| 5273.129122 | Kernel\_Read\|xilinx:adm-pcie-7v3:1ddr:3.0-0\|cnn\_GOOD | Read | OCL Region | 1 | 1 | 109889 | 109890 | 0.00333333 | 5273.129122 | 5273.129126 |
| 5273.129182 | Kernel\_Read\|xilinx:adm-pcie-7v3:1ddr:3.0-0\|cnn\_GOOD | Read | OCL Region | 1 | 1 | 109907 | 109908 | 0.00333333 | 5273.129182 | 5273.129186 |
| 5273.129249 | Kernel\_Read\|xilinx:adm-pcie-7v3:1ddr:3.0-0\|cnn\_GOOD | Read | OCL Region | 1 | 1 | 109927 | 109928 | 0.00333333 | 5273.129249 | 5273.129252 |
| 5273.129322 | Kernel\_Read\|xilinx:adm-pcie-7v3:1ddr:3.0-0\|cnn\_GOOD | Read | OCL Region | 1 | 1 | 109949 | 109950 | 0.00333333 | 5273.129322 | 5273.129326 |
| 5273.129382 | Kernel\_Read\|xilinx:adm-pcie-7v3:1ddr:3.0-0\|cnn\_GOOD | Read | OCL Region | 1 | 1 | 109967 | 109968 | 0.00333333 | 5273.129382 | 5273.129386 |
| 5273.129449 | Kernel\_Read\|xilinx:adm-pcie-7v3:1ddr:3.0-0\|cnn\_GOOD | Read | OCL Region | 1 | 1 | 109987 | 109988 | 0.00333333 | 5273.129449 | 5273.129452 |
| 5273.129522 | Kernel\_Read\|xilinx:adm-pcie-7v3:1ddr:3.0-0\|cnn\_GOOD | Read | OCL Region | 1 | 1 | 110009 | 110010 | 0.00333333 | 5273.129522 | 5273.129526 |
| 5273.129582 | Kernel\_Read\|xilinx:adm-pcie-7v3:1ddr:3.0-0\|cnn\_GOOD | Read | OCL Region | 1 | 1 | 110027 | 110028 | 0.00333333 | 5273.129582 | 5273.129586 |
| 5273.129649 | Kernel\_Read\|xilinx:adm-pcie-7v3:1ddr:3.0-0\|cnn\_GOOD | Read | OCL Region | 1 | 1 | 110047 | 110048 | 0.00333333 | 5273.129649 | 5273.129652 |
| 5273.129722 | Kernel\_Read\|xilinx:adm-pcie-7v3:1ddr:3.0-0\|cnn\_GOOD | Read | OCL Region | 1 | 1 | 110069 | 110070 | 0.00333333 | 5273.129722 | 5273.129726 |
| 5273.129782 | Kernel\_Read\|xilinx:adm-pcie-7v3:1ddr:3.0-0\|cnn\_GOOD | Read | OCL Region | 1 | 1 | 110087 | 110088 | 0.00333333 | 5273.129782 | 5273.129786 |
| 5273.129849 | Kernel\_Read\|xilinx:adm-pcie-7v3:1ddr:3.0-0\|cnn\_GOOD | Read | OCL Region | 1 | 1 | 110107 | 110108 | 0.00333333 | 5273.129849 | 5273.129852 |
| 5273.129922 | Kernel\_Read\|xilinx:adm-pcie-7v3:1ddr:3.0-0\|cnn\_GOOD | Read | OCL Region | 1 | 1 | 110129 | 110130 | 0.00333333 | 5273.129922 | 5273.129926 |
| 5273.129982 | Kernel\_Read\|xilinx:adm-pcie-7v3:1ddr:3.0-0\|cnn\_GOOD | Read | OCL Region | 1 | 1 | 110147 | 110148 | 0.00333333 | 5273.129982 | 5273.129986 |
| 5273.130049 | Kernel\_Read\|xilinx:adm-pcie-7v3:1ddr:3.0-0\|cnn\_GOOD | Read | OCL Region | 1 | 1 | 110167 | 110168 | 0.00333333 | 5273.130049 | 5273.130052 |
| 5273.130122 | Kernel\_Read\|xilinx:adm-pcie-7v3:1ddr:3.0-0\|cnn\_GOOD | Read | OCL Region | 1 | 1 | 110189 | 110190 | 0.00333333 | 5273.130122 | 5273.130126 |
| 5273.130182 | Kernel\_Read\|xilinx:adm-pcie-7v3:1ddr:3.0-0\|cnn\_GOOD | Read | OCL Region | 1 | 1 | 110207 | 110208 | 0.00333333 | 5273.130182 | 5273.130186 |
| 5273.130249 | Kernel\_Read\|xilinx:adm-pcie-7v3:1ddr:3.0-0\|cnn\_GOOD | Read | OCL Region | 1 | 1 | 110227 | 110228 | 0.00333333 | 5273.130249 | 5273.130252 |
| 5273.130322 | Kernel\_Read\|xilinx:adm-pcie-7v3:1ddr:3.0-0\|cnn\_GOOD | Read | OCL Region | 1 | 1 | 110249 | 110250 | 0.00333333 | 5273.130322 | 5273.130326 |
| 5273.130382 | Kernel\_Read\|xilinx:adm-pcie-7v3:1ddr:3.0-0\|cnn\_GOOD | Read | OCL Region | 1 | 1 | 110267 | 110268 | 0.00333333 | 5273.130382 | 5273.130386 |
| 5273.130449 | Kernel\_Read\|xilinx:adm-pcie-7v3:1ddr:3.0-0\|cnn\_GOOD | Read | OCL Region | 1 | 1 | 110287 | 110288 | 0.00333333 | 5273.130449 | 5273.130452 |
| 5273.130739 | Kernel\_Read\|xilinx:adm-pcie-7v3:1ddr:3.0-0\|cnn\_GOOD | Read | OCL Region | 1 | 1 | 110374 | 110375 | 0.00333333 | 5273.130739 | 5273.130742 |
| 5273.130746 | Kernel\_Read\|xilinx:adm-pcie-7v3:1ddr:3.0-0\|cnn\_GOOD | Read | OCL Region | 1 | 1 | 110376 | 110377 | 0.00333333 | 5273.130746 | 5273.130749 |
| 5273.130749 | Kernel\_Read\|xilinx:adm-pcie-7v3:1ddr:3.0-0\|cnn\_GOOD | Read | OCL Region | 1 | 1 | 110377 | 110378 | 0.00333333 | 5273.130749 | 5273.130752 |
| 5273.130756 | Kernel\_Read\|xilinx:adm-pcie-7v3:1ddr:3.0-0\|cnn\_GOOD | Read | OCL Region | 1 | 1 | 110379 | 110380 | 0.00333333 | 5273.130756 | 5273.130759 |
| 5273.130772 | Kernel\_Read\|xilinx:adm-pcie-7v3:1ddr:3.0-0\|cnn\_GOOD | Read | OCL Region | 1 | 1 | 110384 | 110385 | 0.00333333 | 5273.130772 | 5273.130776 |
| 5273.130849 | Kernel\_Read\|xilinx:adm-pcie-7v3:1ddr:3.0-0\|cnn\_GOOD | Read | OCL Region | 1 | 1 | 110407 | 110408 | 0.00333333 | 5273.130849 | 5273.130852 |
| 5273.130922 | Kernel\_Read\|xilinx:adm-pcie-7v3:1ddr:3.0-0\|cnn\_GOOD | Read | OCL Region | 1 | 1 | 110429 | 110430 | 0.00333333 | 5273.130922 | 5273.130926 |
| 5273.130982 | Kernel\_Read\|xilinx:adm-pcie-7v3:1ddr:3.0-0\|cnn\_GOOD | Read | OCL Region | 1 | 1 | 110447 | 110448 | 0.00333333 | 5273.130982 | 5273.130986 |
| 5273.131049 | Kernel\_Read\|xilinx:adm-pcie-7v3:1ddr:3.0-0\|cnn\_GOOD | Read | OCL Region | 1 | 1 | 110467 | 110468 | 0.00333333 | 5273.131049 | 5273.131052 |
| 5273.131122 | Kernel\_Read\|xilinx:adm-pcie-7v3:1ddr:3.0-0\|cnn\_GOOD | Read | OCL Region | 1 | 1 | 110489 | 110490 | 0.00333333 | 5273.131122 | 5273.131126 |
| 5273.131182 | Kernel\_Read\|xilinx:adm-pcie-7v3:1ddr:3.0-0\|cnn\_GOOD | Read | OCL Region | 1 | 1 | 110507 | 110508 | 0.00333333 | 5273.131182 | 5273.131186 |
| 5273.131249 | Kernel\_Read\|xilinx:adm-pcie-7v3:1ddr:3.0-0\|cnn\_GOOD | Read | OCL Region | 1 | 1 | 110527 | 110528 | 0.00333333 | 5273.131249 | 5273.131252 |
| 5273.131322 | Kernel\_Read\|xilinx:adm-pcie-7v3:1ddr:3.0-0\|cnn\_GOOD | Read | OCL Region | 1 | 1 | 110549 | 110550 | 0.00333333 | 5273.131322 | 5273.131326 |
| 5273.131382 | Kernel\_Read\|xilinx:adm-pcie-7v3:1ddr:3.0-0\|cnn\_GOOD | Read | OCL Region | 1 | 1 | 110567 | 110568 | 0.00333333 | 5273.131382 | 5273.131386 |
| 5273.131449 | Kernel\_Read\|xilinx:adm-pcie-7v3:1ddr:3.0-0\|cnn\_GOOD | Read | OCL Region | 1 | 1 | 110587 | 110588 | 0.00333333 | 5273.131449 | 5273.131452 |
| 5273.131522 | Kernel\_Read\|xilinx:adm-pcie-7v3:1ddr:3.0-0\|cnn\_GOOD | Read | OCL Region | 1 | 1 | 110609 | 110610 | 0.00333333 | 5273.131522 | 5273.131526 |
| 5273.131582 | Kernel\_Read\|xilinx:adm-pcie-7v3:1ddr:3.0-0\|cnn\_GOOD | Read | OCL Region | 1 | 1 | 110627 | 110628 | 0.00333333 | 5273.131582 | 5273.131586 |
| 5273.131649 | Kernel\_Read\|xilinx:adm-pcie-7v3:1ddr:3.0-0\|cnn\_GOOD | Read | OCL Region | 1 | 1 | 110647 | 110648 | 0.00333333 | 5273.131649 | 5273.131652 |
| 5273.131722 | Kernel\_Read\|xilinx:adm-pcie-7v3:1ddr:3.0-0\|cnn\_GOOD | Read | OCL Region | 1 | 1 | 110669 | 110670 | 0.00333333 | 5273.131722 | 5273.131726 |
| 5273.131782 | Kernel\_Read\|xilinx:adm-pcie-7v3:1ddr:3.0-0\|cnn\_GOOD | Read | OCL Region | 1 | 1 | 110687 | 110688 | 0.00333333 | 5273.131782 | 5273.131786 |
| 5273.131849 | Kernel\_Read\|xilinx:adm-pcie-7v3:1ddr:3.0-0\|cnn\_GOOD | Read | OCL Region | 1 | 1 | 110707 | 110708 | 0.00333333 | 5273.131849 | 5273.131852 |
| 5273.131922 | Kernel\_Read\|xilinx:adm-pcie-7v3:1ddr:3.0-0\|cnn\_GOOD | Read | OCL Region | 1 | 1 | 110729 | 110730 | 0.00333333 | 5273.131922 | 5273.131926 |
| 5273.131982 | Kernel\_Read\|xilinx:adm-pcie-7v3:1ddr:3.0-0\|cnn\_GOOD | Read | OCL Region | 1 | 1 | 110747 | 110748 | 0.00333333 | 5273.131982 | 5273.131986 |
| 5273.132049 | Kernel\_Read\|xilinx:adm-pcie-7v3:1ddr:3.0-0\|cnn\_GOOD | Read | OCL Region | 1 | 1 | 110767 | 110768 | 0.00333333 | 5273.132049 | 5273.132052 |
| 5273.132122 | Kernel\_Read\|xilinx:adm-pcie-7v3:1ddr:3.0-0\|cnn\_GOOD | Read | OCL Region | 1 | 1 | 110789 | 110790 | 0.00333333 | 5273.132122 | 5273.132126 |
| 5273.132182 | Kernel\_Read\|xilinx:adm-pcie-7v3:1ddr:3.0-0\|cnn\_GOOD | Read | OCL Region | 1 | 1 | 110807 | 110808 | 0.00333333 | 5273.132182 | 5273.132186 |
| 5273.132249 | Kernel\_Read\|xilinx:adm-pcie-7v3:1ddr:3.0-0\|cnn\_GOOD | Read | OCL Region | 1 | 1 | 110827 | 110828 | 0.00333333 | 5273.132249 | 5273.132252 |
| 5273.132322 | Kernel\_Read\|xilinx:adm-pcie-7v3:1ddr:3.0-0\|cnn\_GOOD | Read | OCL Region | 1 | 1 | 110849 | 110850 | 0.00333333 | 5273.132322 | 5273.132326 |
| 5273.132382 | Kernel\_Read\|xilinx:adm-pcie-7v3:1ddr:3.0-0\|cnn\_GOOD | Read | OCL Region | 1 | 1 | 110867 | 110868 | 0.00333333 | 5273.132382 | 5273.132386 |
| 5273.132449 | Kernel\_Read\|xilinx:adm-pcie-7v3:1ddr:3.0-0\|cnn\_GOOD | Read | OCL Region | 1 | 1 | 110887 | 110888 | 0.00333333 | 5273.132449 | 5273.132452 |
| 5273.132522 | Kernel\_Read\|xilinx:adm-pcie-7v3:1ddr:3.0-0\|cnn\_GOOD | Read | OCL Region | 1 | 1 | 110909 | 110910 | 0.00333333 | 5273.132522 | 5273.132526 |
| 5273.132582 | Kernel\_Read\|xilinx:adm-pcie-7v3:1ddr:3.0-0\|cnn\_GOOD | Read | OCL Region | 1 | 1 | 110927 | 110928 | 0.00333333 | 5273.132582 | 5273.132586 |
| 5273.132649 | Kernel\_Read\|xilinx:adm-pcie-7v3:1ddr:3.0-0\|cnn\_GOOD | Read | OCL Region | 1 | 1 | 110947 | 110948 | 0.00333333 | 5273.132649 | 5273.132652 |
| 5273.132722 | Kernel\_Read\|xilinx:adm-pcie-7v3:1ddr:3.0-0\|cnn\_GOOD | Read | OCL Region | 1 | 1 | 110969 | 110970 | 0.00333333 | 5273.132722 | 5273.132726 |
| 5273.132782 | Kernel\_Read\|xilinx:adm-pcie-7v3:1ddr:3.0-0\|cnn\_GOOD | Read | OCL Region | 1 | 1 | 110987 | 110988 | 0.00333333 | 5273.132782 | 5273.132786 |
| 5273.132849 | Kernel\_Read\|xilinx:adm-pcie-7v3:1ddr:3.0-0\|cnn\_GOOD | Read | OCL Region | 1 | 1 | 111007 | 111008 | 0.00333333 | 5273.132849 | 5273.132852 |
| 5273.132922 | Kernel\_Read\|xilinx:adm-pcie-7v3:1ddr:3.0-0\|cnn\_GOOD | Read | OCL Region | 1 | 1 | 111029 | 111030 | 0.00333333 | 5273.132922 | 5273.132926 |
| 5273.132982 | Kernel\_Read\|xilinx:adm-pcie-7v3:1ddr:3.0-0\|cnn\_GOOD | Read | OCL Region | 1 | 1 | 111047 | 111048 | 0.00333333 | 5273.132982 | 5273.132986 |
| 5273.133049 | Kernel\_Read\|xilinx:adm-pcie-7v3:1ddr:3.0-0\|cnn\_GOOD | Read | OCL Region | 1 | 1 | 111067 | 111068 | 0.00333333 | 5273.133049 | 5273.133052 |
| 5273.133122 | Kernel\_Read\|xilinx:adm-pcie-7v3:1ddr:3.0-0\|cnn\_GOOD | Read | OCL Region | 1 | 1 | 111089 | 111090 | 0.00333333 | 5273.133122 | 5273.133126 |
| 5273.133182 | Kernel\_Read\|xilinx:adm-pcie-7v3:1ddr:3.0-0\|cnn\_GOOD | Read | OCL Region | 1 | 1 | 111107 | 111108 | 0.00333333 | 5273.133182 | 5273.133186 |
| 5273.133249 | Kernel\_Read\|xilinx:adm-pcie-7v3:1ddr:3.0-0\|cnn\_GOOD | Read | OCL Region | 1 | 1 | 111127 | 111128 | 0.00333333 | 5273.133249 | 5273.133252 |
| 5273.133322 | Kernel\_Read\|xilinx:adm-pcie-7v3:1ddr:3.0-0\|cnn\_GOOD | Read | OCL Region | 1 | 1 | 111149 | 111150 | 0.00333333 | 5273.133322 | 5273.133326 |
| 5273.133382 | Kernel\_Read\|xilinx:adm-pcie-7v3:1ddr:3.0-0\|cnn\_GOOD | Read | OCL Region | 1 | 1 | 111167 | 111168 | 0.00333333 | 5273.133382 | 5273.133386 |
| 5273.133449 | Kernel\_Read\|xilinx:adm-pcie-7v3:1ddr:3.0-0\|cnn\_GOOD | Read | OCL Region | 1 | 1 | 111187 | 111188 | 0.00333333 | 5273.133449 | 5273.133452 |
| 5273.133522 | Kernel\_Read\|xilinx:adm-pcie-7v3:1ddr:3.0-0\|cnn\_GOOD | Read | OCL Region | 1 | 1 | 111209 | 111210 | 0.00333333 | 5273.133522 | 5273.133526 |
| 5273.133582 | Kernel\_Read\|xilinx:adm-pcie-7v3:1ddr:3.0-0\|cnn\_GOOD | Read | OCL Region | 1 | 1 | 111227 | 111228 | 0.00333333 | 5273.133582 | 5273.133586 |
| 5273.133649 | Kernel\_Read\|xilinx:adm-pcie-7v3:1ddr:3.0-0\|cnn\_GOOD | Read | OCL Region | 1 | 1 | 111247 | 111248 | 0.00333333 | 5273.133649 | 5273.133652 |
| 5273.133722 | Kernel\_Read\|xilinx:adm-pcie-7v3:1ddr:3.0-0\|cnn\_GOOD | Read | OCL Region | 1 | 1 | 111269 | 111270 | 0.00333333 | 5273.133722 | 5273.133726 |
| 5273.133782 | Kernel\_Read\|xilinx:adm-pcie-7v3:1ddr:3.0-0\|cnn\_GOOD | Read | OCL Region | 1 | 1 | 111287 | 111288 | 0.00333333 | 5273.133782 | 5273.133786 |
| 5273.133849 | Kernel\_Read\|xilinx:adm-pcie-7v3:1ddr:3.0-0\|cnn\_GOOD | Read | OCL Region | 1 | 1 | 111307 | 111308 | 0.00333333 | 5273.133849 | 5273.133852 |
| 5273.133922 | Kernel\_Read\|xilinx:adm-pcie-7v3:1ddr:3.0-0\|cnn\_GOOD | Read | OCL Region | 1 | 1 | 111329 | 111330 | 0.00333333 | 5273.133922 | 5273.133926 |
| 5273.133996 | Kernel\_Read\|xilinx:adm-pcie-7v3:1ddr:3.0-0\|cnn\_GOOD | Read | OCL Region | 1 | 1 | 111351 | 111352 | 0.00333333 | 5273.133996 | 5273.133999 |
| 5273.134049 | Kernel\_Read\|xilinx:adm-pcie-7v3:1ddr:3.0-0\|cnn\_GOOD | Read | OCL Region | 1 | 1 | 111367 | 111368 | 0.00333333 | 5273.134049 | 5273.134052 |
| 5273.134122 | Kernel\_Read\|xilinx:adm-pcie-7v3:1ddr:3.0-0\|cnn\_GOOD | Read | OCL Region | 1 | 1 | 111389 | 111390 | 0.00333333 | 5273.134122 | 5273.134126 |
| 5273.134182 | Kernel\_Read\|xilinx:adm-pcie-7v3:1ddr:3.0-0\|cnn\_GOOD | Read | OCL Region | 1 | 1 | 111407 | 111408 | 0.00333333 | 5273.134182 | 5273.134186 |
| 5273.134249 | Kernel\_Read\|xilinx:adm-pcie-7v3:1ddr:3.0-0\|cnn\_GOOD | Read | OCL Region | 1 | 1 | 111427 | 111428 | 0.00333333 | 5273.134249 | 5273.134252 |
| 5273.134322 | Kernel\_Read\|xilinx:adm-pcie-7v3:1ddr:3.0-0\|cnn\_GOOD | Read | OCL Region | 1 | 1 | 111449 | 111450 | 0.00333333 | 5273.134322 | 5273.134326 |
| 5273.134382 | Kernel\_Read\|xilinx:adm-pcie-7v3:1ddr:3.0-0\|cnn\_GOOD | Read | OCL Region | 1 | 1 | 111467 | 111468 | 0.00333333 | 5273.134382 | 5273.134386 |
| 5273.134449 | Kernel\_Read\|xilinx:adm-pcie-7v3:1ddr:3.0-0\|cnn\_GOOD | Read | OCL Region | 1 | 1 | 111487 | 111488 | 0.00333333 | 5273.134449 | 5273.134452 |
| 5273.134522 | Kernel\_Read\|xilinx:adm-pcie-7v3:1ddr:3.0-0\|cnn\_GOOD | Read | OCL Region | 1 | 1 | 111509 | 111510 | 0.00333333 | 5273.134522 | 5273.134526 |
| 5273.134582 | Kernel\_Read\|xilinx:adm-pcie-7v3:1ddr:3.0-0\|cnn\_GOOD | Read | OCL Region | 1 | 1 | 111527 | 111528 | 0.00333333 | 5273.134582 | 5273.134586 |
| 5273.134649 | Kernel\_Read\|xilinx:adm-pcie-7v3:1ddr:3.0-0\|cnn\_GOOD | Read | OCL Region | 1 | 1 | 111547 | 111548 | 0.00333333 | 5273.134649 | 5273.134652 |
| 5273.134722 | Kernel\_Read\|xilinx:adm-pcie-7v3:1ddr:3.0-0\|cnn\_GOOD | Read | OCL Region | 1 | 1 | 111569 | 111570 | 0.00333333 | 5273.134722 | 5273.134726 |
| 5273.134782 | Kernel\_Read\|xilinx:adm-pcie-7v3:1ddr:3.0-0\|cnn\_GOOD | Read | OCL Region | 1 | 1 | 111587 | 111588 | 0.00333333 | 5273.134782 | 5273.134786 |
| 5273.134856 | Kernel\_Read\|xilinx:adm-pcie-7v3:1ddr:3.0-0\|cnn\_GOOD | Read | OCL Region | 1 | 1 | 111609 | 111610 | 0.00333333 | 5273.134856 | 5273.134859 |
| 5273.134922 | Kernel\_Read\|xilinx:adm-pcie-7v3:1ddr:3.0-0\|cnn\_GOOD | Read | OCL Region | 1 | 1 | 111629 | 111630 | 0.00333333 | 5273.134922 | 5273.134926 |
| 5273.134982 | Kernel\_Read\|xilinx:adm-pcie-7v3:1ddr:3.0-0\|cnn\_GOOD | Read | OCL Region | 1 | 1 | 111647 | 111648 | 0.00333333 | 5273.134982 | 5273.134986 |
| 5273.135049 | Kernel\_Read\|xilinx:adm-pcie-7v3:1ddr:3.0-0\|cnn\_GOOD | Read | OCL Region | 1 | 1 | 111667 | 111668 | 0.00333333 | 5273.135049 | 5273.135052 |
| 5273.135122 | Kernel\_Read\|xilinx:adm-pcie-7v3:1ddr:3.0-0\|cnn\_GOOD | Read | OCL Region | 1 | 1 | 111689 | 111690 | 0.00333333 | 5273.135122 | 5273.135126 |
| 5273.135182 | Kernel\_Read\|xilinx:adm-pcie-7v3:1ddr:3.0-0\|cnn\_GOOD | Read | OCL Region | 1 | 1 | 111707 | 111708 | 0.00333333 | 5273.135182 | 5273.135186 |
| 5273.135249 | Kernel\_Read\|xilinx:adm-pcie-7v3:1ddr:3.0-0\|cnn\_GOOD | Read | OCL Region | 1 | 1 | 111727 | 111728 | 0.00333333 | 5273.135249 | 5273.135252 |
| 5273.135322 | Kernel\_Read\|xilinx:adm-pcie-7v3:1ddr:3.0-0\|cnn\_GOOD | Read | OCL Region | 1 | 1 | 111749 | 111750 | 0.00333333 | 5273.135322 | 5273.135326 |
| 5273.135382 | Kernel\_Read\|xilinx:adm-pcie-7v3:1ddr:3.0-0\|cnn\_GOOD | Read | OCL Region | 1 | 1 | 111767 | 111768 | 0.00333333 | 5273.135382 | 5273.135386 |
| 5273.135449 | Kernel\_Read\|xilinx:adm-pcie-7v3:1ddr:3.0-0\|cnn\_GOOD | Read | OCL Region | 1 | 1 | 111787 | 111788 | 0.00333333 | 5273.135449 | 5273.135452 |
| 5273.135522 | Kernel\_Read\|xilinx:adm-pcie-7v3:1ddr:3.0-0\|cnn\_GOOD | Read | OCL Region | 1 | 1 | 111809 | 111810 | 0.00333333 | 5273.135522 | 5273.135526 |
| 5273.135582 | Kernel\_Read\|xilinx:adm-pcie-7v3:1ddr:3.0-0\|cnn\_GOOD | Read | OCL Region | 1 | 1 | 111827 | 111828 | 0.00333333 | 5273.135582 | 5273.135586 |
| 5273.135649 | Kernel\_Read\|xilinx:adm-pcie-7v3:1ddr:3.0-0\|cnn\_GOOD | Read | OCL Region | 1 | 1 | 111847 | 111848 | 0.00333333 | 5273.135649 | 5273.135652 |
| 5273.135722 | Kernel\_Read\|xilinx:adm-pcie-7v3:1ddr:3.0-0\|cnn\_GOOD | Read | OCL Region | 1 | 1 | 111869 | 111870 | 0.00333333 | 5273.135722 | 5273.135726 |
| 5273.135782 | Kernel\_Read\|xilinx:adm-pcie-7v3:1ddr:3.0-0\|cnn\_GOOD | Read | OCL Region | 1 | 1 | 111887 | 111888 | 0.00333333 | 5273.135782 | 5273.135786 |
| 5273.135849 | Kernel\_Read\|xilinx:adm-pcie-7v3:1ddr:3.0-0\|cnn\_GOOD | Read | OCL Region | 1 | 1 | 111907 | 111908 | 0.00333333 | 5273.135849 | 5273.135852 |
| 5273.135922 | Kernel\_Read\|xilinx:adm-pcie-7v3:1ddr:3.0-0\|cnn\_GOOD | Read | OCL Region | 1 | 1 | 111929 | 111930 | 0.00333333 | 5273.135922 | 5273.135926 |
| 5273.135982 | Kernel\_Read\|xilinx:adm-pcie-7v3:1ddr:3.0-0\|cnn\_GOOD | Read | OCL Region | 1 | 1 | 111947 | 111948 | 0.00333333 | 5273.135982 | 5273.135986 |
| 5273.136049 | Kernel\_Read\|xilinx:adm-pcie-7v3:1ddr:3.0-0\|cnn\_GOOD | Read | OCL Region | 1 | 1 | 111967 | 111968 | 0.00333333 | 5273.136049 | 5273.136052 |
| 5273.136122 | Kernel\_Read\|xilinx:adm-pcie-7v3:1ddr:3.0-0\|cnn\_GOOD | Read | OCL Region | 1 | 1 | 111989 | 111990 | 0.00333333 | 5273.136122 | 5273.136126 |
| 5273.136182 | Kernel\_Read\|xilinx:adm-pcie-7v3:1ddr:3.0-0\|cnn\_GOOD | Read | OCL Region | 1 | 1 | 112007 | 112008 | 0.00333333 | 5273.136182 | 5273.136186 |
| 5273.136249 | Kernel\_Read\|xilinx:adm-pcie-7v3:1ddr:3.0-0\|cnn\_GOOD | Read | OCL Region | 1 | 1 | 112027 | 112028 | 0.00333333 | 5273.136249 | 5273.136252 |
| 5273.136322 | Kernel\_Read\|xilinx:adm-pcie-7v3:1ddr:3.0-0\|cnn\_GOOD | Read | OCL Region | 1 | 1 | 112049 | 112050 | 0.00333333 | 5273.136322 | 5273.136326 |
| 5273.136382 | Kernel\_Read\|xilinx:adm-pcie-7v3:1ddr:3.0-0\|cnn\_GOOD | Read | OCL Region | 1 | 1 | 112067 | 112068 | 0.00333333 | 5273.136382 | 5273.136386 |
| 5273.136449 | Kernel\_Read\|xilinx:adm-pcie-7v3:1ddr:3.0-0\|cnn\_GOOD | Read | OCL Region | 1 | 1 | 112087 | 112088 | 0.00333333 | 5273.136449 | 5273.136452 |
| 5273.136522 | Kernel\_Read\|xilinx:adm-pcie-7v3:1ddr:3.0-0\|cnn\_GOOD | Read | OCL Region | 1 | 1 | 112109 | 112110 | 0.00333333 | 5273.136522 | 5273.136526 |
| 5273.136582 | Kernel\_Read\|xilinx:adm-pcie-7v3:1ddr:3.0-0\|cnn\_GOOD | Read | OCL Region | 1 | 1 | 112127 | 112128 | 0.00333333 | 5273.136582 | 5273.136586 |
| 5273.136649 | Kernel\_Read\|xilinx:adm-pcie-7v3:1ddr:3.0-0\|cnn\_GOOD | Read | OCL Region | 1 | 1 | 112147 | 112148 | 0.00333333 | 5273.136649 | 5273.136652 |
| 5273.136722 | Kernel\_Read\|xilinx:adm-pcie-7v3:1ddr:3.0-0\|cnn\_GOOD | Read | OCL Region | 1 | 1 | 112169 | 112170 | 0.00333333 | 5273.136722 | 5273.136726 |
| 5273.136782 | Kernel\_Read\|xilinx:adm-pcie-7v3:1ddr:3.0-0\|cnn\_GOOD | Read | OCL Region | 1 | 1 | 112187 | 112188 | 0.00333333 | 5273.136782 | 5273.136786 |
| 5273.137059 | Kernel\_Read\|xilinx:adm-pcie-7v3:1ddr:3.0-0\|cnn\_GOOD | Read | OCL Region | 1 | 1 | 112270 | 112271 | 0.00333333 | 5273.137059 | 5273.137062 |
| 5273.137062 | Kernel\_Read\|xilinx:adm-pcie-7v3:1ddr:3.0-0\|cnn\_GOOD | Read | OCL Region | 1 | 1 | 112271 | 112272 | 0.00333333 | 5273.137062 | 5273.137066 |
| 5273.137072 | Kernel\_Read\|xilinx:adm-pcie-7v3:1ddr:3.0-0\|cnn\_GOOD | Read | OCL Region | 1 | 1 | 112274 | 112275 | 0.00333333 | 5273.137072 | 5273.137076 |
| 5273.137076 | Kernel\_Read\|xilinx:adm-pcie-7v3:1ddr:3.0-0\|cnn\_GOOD | Read | OCL Region | 1 | 1 | 112275 | 112276 | 0.00333333 | 5273.137076 | 5273.137079 |
| 5273.137132 | Kernel\_Read\|xilinx:adm-pcie-7v3:1ddr:3.0-0\|cnn\_GOOD | Read | OCL Region | 1 | 1 | 112292 | 112293 | 0.00333333 | 5273.137132 | 5273.137136 |
| 5273.137189 | Kernel\_Read\|xilinx:adm-pcie-7v3:1ddr:3.0-0\|cnn\_GOOD | Read | OCL Region | 1 | 1 | 112309 | 112310 | 0.00333333 | 5273.137189 | 5273.137192 |
| 5273.137249 | Kernel\_Read\|xilinx:adm-pcie-7v3:1ddr:3.0-0\|cnn\_GOOD | Read | OCL Region | 1 | 1 | 112327 | 112328 | 0.00333333 | 5273.137249 | 5273.137252 |
| 5273.137322 | Kernel\_Read\|xilinx:adm-pcie-7v3:1ddr:3.0-0\|cnn\_GOOD | Read | OCL Region | 1 | 1 | 112349 | 112350 | 0.00333333 | 5273.137322 | 5273.137326 |
| 5273.137382 | Kernel\_Read\|xilinx:adm-pcie-7v3:1ddr:3.0-0\|cnn\_GOOD | Read | OCL Region | 1 | 1 | 112367 | 112368 | 0.00333333 | 5273.137382 | 5273.137386 |
| 5273.137449 | Kernel\_Read\|xilinx:adm-pcie-7v3:1ddr:3.0-0\|cnn\_GOOD | Read | OCL Region | 1 | 1 | 112387 | 112388 | 0.00333333 | 5273.137449 | 5273.137452 |
| 5273.137522 | Kernel\_Read\|xilinx:adm-pcie-7v3:1ddr:3.0-0\|cnn\_GOOD | Read | OCL Region | 1 | 1 | 112409 | 112410 | 0.00333333 | 5273.137522 | 5273.137526 |
| 5273.137582 | Kernel\_Read\|xilinx:adm-pcie-7v3:1ddr:3.0-0\|cnn\_GOOD | Read | OCL Region | 1 | 1 | 112427 | 112428 | 0.00333333 | 5273.137582 | 5273.137586 |
| 5273.137649 | Kernel\_Read\|xilinx:adm-pcie-7v3:1ddr:3.0-0\|cnn\_GOOD | Read | OCL Region | 1 | 1 | 112447 | 112448 | 0.00333333 | 5273.137649 | 5273.137652 |
| 5273.137722 | Kernel\_Read\|xilinx:adm-pcie-7v3:1ddr:3.0-0\|cnn\_GOOD | Read | OCL Region | 1 | 1 | 112469 | 112470 | 0.00333333 | 5273.137722 | 5273.137726 |
| 5273.137782 | Kernel\_Read\|xilinx:adm-pcie-7v3:1ddr:3.0-0\|cnn\_GOOD | Read | OCL Region | 1 | 1 | 112487 | 112488 | 0.00333333 | 5273.137782 | 5273.137786 |
| 5273.137849 | Kernel\_Read\|xilinx:adm-pcie-7v3:1ddr:3.0-0\|cnn\_GOOD | Read | OCL Region | 1 | 1 | 112507 | 112508 | 0.00333333 | 5273.137849 | 5273.137852 |
| 5273.137922 | Kernel\_Read\|xilinx:adm-pcie-7v3:1ddr:3.0-0\|cnn\_GOOD | Read | OCL Region | 1 | 1 | 112529 | 112530 | 0.00333333 | 5273.137922 | 5273.137926 |
| 5273.137989 | Kernel\_Read\|xilinx:adm-pcie-7v3:1ddr:3.0-0\|cnn\_GOOD | Read | OCL Region | 1 | 1 | 112549 | 112550 | 0.00333333 | 5273.137989 | 5273.137992 |
| 5273.138049 | Kernel\_Read\|xilinx:adm-pcie-7v3:1ddr:3.0-0\|cnn\_GOOD | Read | OCL Region | 1 | 1 | 112567 | 112568 | 0.00333333 | 5273.138049 | 5273.138052 |
| 5273.138122 | Kernel\_Read\|xilinx:adm-pcie-7v3:1ddr:3.0-0\|cnn\_GOOD | Read | OCL Region | 1 | 1 | 112589 | 112590 | 0.00333333 | 5273.138122 | 5273.138126 |
| 5273.138182 | Kernel\_Read\|xilinx:adm-pcie-7v3:1ddr:3.0-0\|cnn\_GOOD | Read | OCL Region | 1 | 1 | 112607 | 112608 | 0.00333333 | 5273.138182 | 5273.138186 |
| 5273.138249 | Kernel\_Read\|xilinx:adm-pcie-7v3:1ddr:3.0-0\|cnn\_GOOD | Read | OCL Region | 1 | 1 | 112627 | 112628 | 0.00333333 | 5273.138249 | 5273.138252 |
| 5273.138322 | Kernel\_Read\|xilinx:adm-pcie-7v3:1ddr:3.0-0\|cnn\_GOOD | Read | OCL Region | 1 | 1 | 112649 | 112650 | 0.00333333 | 5273.138322 | 5273.138326 |
| 5273.138382 | Kernel\_Read\|xilinx:adm-pcie-7v3:1ddr:3.0-0\|cnn\_GOOD | Read | OCL Region | 1 | 1 | 112667 | 112668 | 0.00333333 | 5273.138382 | 5273.138386 |
| 5273.138449 | Kernel\_Read\|xilinx:adm-pcie-7v3:1ddr:3.0-0\|cnn\_GOOD | Read | OCL Region | 1 | 1 | 112687 | 112688 | 0.00333333 | 5273.138449 | 5273.138452 |
| 5273.138522 | Kernel\_Read\|xilinx:adm-pcie-7v3:1ddr:3.0-0\|cnn\_GOOD | Read | OCL Region | 1 | 1 | 112709 | 112710 | 0.00333333 | 5273.138522 | 5273.138526 |
| 5273.138582 | Kernel\_Read\|xilinx:adm-pcie-7v3:1ddr:3.0-0\|cnn\_GOOD | Read | OCL Region | 1 | 1 | 112727 | 112728 | 0.00333333 | 5273.138582 | 5273.138586 |
| 5273.138649 | Kernel\_Read\|xilinx:adm-pcie-7v3:1ddr:3.0-0\|cnn\_GOOD | Read | OCL Region | 1 | 1 | 112747 | 112748 | 0.00333333 | 5273.138649 | 5273.138652 |
| 5273.138712 | Kernel\_Read\|xilinx:adm-pcie-7v3:1ddr:3.0-0\|cnn\_GOOD | Read | OCL Region | 1 | 1 | 112766 | 112767 | 0.00333333 | 5273.138712 | 5273.138716 |
| 5273.138779 | Kernel\_Read\|xilinx:adm-pcie-7v3:1ddr:3.0-0\|cnn\_GOOD | Read | OCL Region | 1 | 1 | 112786 | 112787 | 0.00333333 | 5273.138779 | 5273.138782 |
| 5273.138849 | Kernel\_Read\|xilinx:adm-pcie-7v3:1ddr:3.0-0\|cnn\_GOOD | Read | OCL Region | 1 | 1 | 112807 | 112808 | 0.00333333 | 5273.138849 | 5273.138852 |
| 5273.138922 | Kernel\_Read\|xilinx:adm-pcie-7v3:1ddr:3.0-0\|cnn\_GOOD | Read | OCL Region | 1 | 1 | 112829 | 112830 | 0.00333333 | 5273.138922 | 5273.138926 |
| 5273.138982 | Kernel\_Read\|xilinx:adm-pcie-7v3:1ddr:3.0-0\|cnn\_GOOD | Read | OCL Region | 1 | 1 | 112847 | 112848 | 0.00333333 | 5273.138982 | 5273.138986 |
| 5273.139049 | Kernel\_Read\|xilinx:adm-pcie-7v3:1ddr:3.0-0\|cnn\_GOOD | Read | OCL Region | 1 | 1 | 112867 | 112868 | 0.00333333 | 5273.139049 | 5273.139052 |
| 5273.139122 | Kernel\_Read\|xilinx:adm-pcie-7v3:1ddr:3.0-0\|cnn\_GOOD | Read | OCL Region | 1 | 1 | 112889 | 112890 | 0.00333333 | 5273.139122 | 5273.139126 |
| 5273.139182 | Kernel\_Read\|xilinx:adm-pcie-7v3:1ddr:3.0-0\|cnn\_GOOD | Read | OCL Region | 1 | 1 | 112907 | 112908 | 0.00333333 | 5273.139182 | 5273.139186 |
| 5273.139249 | Kernel\_Read\|xilinx:adm-pcie-7v3:1ddr:3.0-0\|cnn\_GOOD | Read | OCL Region | 1 | 1 | 112927 | 112928 | 0.00333333 | 5273.139249 | 5273.139252 |
| 5273.139322 | Kernel\_Read\|xilinx:adm-pcie-7v3:1ddr:3.0-0\|cnn\_GOOD | Read | OCL Region | 1 | 1 | 112949 | 112950 | 0.00333333 | 5273.139322 | 5273.139326 |
| 5273.139382 | Kernel\_Read\|xilinx:adm-pcie-7v3:1ddr:3.0-0\|cnn\_GOOD | Read | OCL Region | 1 | 1 | 112967 | 112968 | 0.00333333 | 5273.139382 | 5273.139386 |
| 5273.139449 | Kernel\_Read\|xilinx:adm-pcie-7v3:1ddr:3.0-0\|cnn\_GOOD | Read | OCL Region | 1 | 1 | 112987 | 112988 | 0.00333333 | 5273.139449 | 5273.139452 |
| 5273.139512 | Kernel\_Read\|xilinx:adm-pcie-7v3:1ddr:3.0-0\|cnn\_GOOD | Read | OCL Region | 1 | 1 | 113006 | 113007 | 0.00333333 | 5273.139512 | 5273.139516 |
| 5273.139582 | Kernel\_Read\|xilinx:adm-pcie-7v3:1ddr:3.0-0\|cnn\_GOOD | Read | OCL Region | 1 | 1 | 113027 | 113028 | 0.00333333 | 5273.139582 | 5273.139586 |
| 5273.139649 | Kernel\_Read\|xilinx:adm-pcie-7v3:1ddr:3.0-0\|cnn\_GOOD | Read | OCL Region | 1 | 1 | 113047 | 113048 | 0.00333333 | 5273.139649 | 5273.139652 |
| 5273.139722 | Kernel\_Read\|xilinx:adm-pcie-7v3:1ddr:3.0-0\|cnn\_GOOD | Read | OCL Region | 1 | 1 | 113069 | 113070 | 0.00333333 | 5273.139722 | 5273.139726 |
| 5273.139782 | Kernel\_Read\|xilinx:adm-pcie-7v3:1ddr:3.0-0\|cnn\_GOOD | Read | OCL Region | 1 | 1 | 113087 | 113088 | 0.00333333 | 5273.139782 | 5273.139786 |
| 5273.139849 | Kernel\_Read\|xilinx:adm-pcie-7v3:1ddr:3.0-0\|cnn\_GOOD | Read | OCL Region | 1 | 1 | 113107 | 113108 | 0.00333333 | 5273.139849 | 5273.139852 |
| 5273.139922 | Kernel\_Read\|xilinx:adm-pcie-7v3:1ddr:3.0-0\|cnn\_GOOD | Read | OCL Region | 1 | 1 | 113129 | 113130 | 0.00333333 | 5273.139922 | 5273.139926 |
| 5273.139982 | Kernel\_Read\|xilinx:adm-pcie-7v3:1ddr:3.0-0\|cnn\_GOOD | Read | OCL Region | 1 | 1 | 113147 | 113148 | 0.00333333 | 5273.139982 | 5273.139986 |
| 5273.140049 | Kernel\_Read\|xilinx:adm-pcie-7v3:1ddr:3.0-0\|cnn\_GOOD | Read | OCL Region | 1 | 1 | 113167 | 113168 | 0.00333333 | 5273.140049 | 5273.140052 |
| 5273.140122 | Kernel\_Read\|xilinx:adm-pcie-7v3:1ddr:3.0-0\|cnn\_GOOD | Read | OCL Region | 1 | 1 | 113189 | 113190 | 0.00333333 | 5273.140122 | 5273.140126 |
| 5273.140182 | Kernel\_Read\|xilinx:adm-pcie-7v3:1ddr:3.0-0\|cnn\_GOOD | Read | OCL Region | 1 | 1 | 113207 | 113208 | 0.00333333 | 5273.140182 | 5273.140186 |
| 5273.140249 | Kernel\_Read\|xilinx:adm-pcie-7v3:1ddr:3.0-0\|cnn\_GOOD | Read | OCL Region | 1 | 1 | 113227 | 113228 | 0.00333333 | 5273.140249 | 5273.140252 |
| 5273.140312 | Kernel\_Read\|xilinx:adm-pcie-7v3:1ddr:3.0-0\|cnn\_GOOD | Read | OCL Region | 1 | 1 | 113246 | 113247 | 0.00333333 | 5273.140312 | 5273.140316 |
| 5273.140382 | Kernel\_Read\|xilinx:adm-pcie-7v3:1ddr:3.0-0\|cnn\_GOOD | Read | OCL Region | 1 | 1 | 113267 | 113268 | 0.00333333 | 5273.140382 | 5273.140386 |
| 5273.140462 | Kernel\_Read\|xilinx:adm-pcie-7v3:1ddr:3.0-0\|cnn\_GOOD | Read | OCL Region | 1 | 1 | 113291 | 113292 | 0.00333333 | 5273.140462 | 5273.140466 |
| 5273.140522 | Kernel\_Read\|xilinx:adm-pcie-7v3:1ddr:3.0-0\|cnn\_GOOD | Read | OCL Region | 1 | 1 | 113309 | 113310 | 0.00333333 | 5273.140522 | 5273.140526 |
| 5273.140582 | Kernel\_Read\|xilinx:adm-pcie-7v3:1ddr:3.0-0\|cnn\_GOOD | Read | OCL Region | 1 | 1 | 113327 | 113328 | 0.00333333 | 5273.140582 | 5273.140586 |
| 5273.140649 | Kernel\_Read\|xilinx:adm-pcie-7v3:1ddr:3.0-0\|cnn\_GOOD | Read | OCL Region | 1 | 1 | 113347 | 113348 | 0.00333333 | 5273.140649 | 5273.140652 |
| 5273.140722 | Kernel\_Read\|xilinx:adm-pcie-7v3:1ddr:3.0-0\|cnn\_GOOD | Read | OCL Region | 1 | 1 | 113369 | 113370 | 0.00333333 | 5273.140722 | 5273.140726 |
| 5273.140782 | Kernel\_Read\|xilinx:adm-pcie-7v3:1ddr:3.0-0\|cnn\_GOOD | Read | OCL Region | 1 | 1 | 113387 | 113388 | 0.00333333 | 5273.140782 | 5273.140786 |
| 5273.140849 | Kernel\_Read\|xilinx:adm-pcie-7v3:1ddr:3.0-0\|cnn\_GOOD | Read | OCL Region | 1 | 1 | 113407 | 113408 | 0.00333333 | 5273.140849 | 5273.140852 |
| 5273.140922 | Kernel\_Read\|xilinx:adm-pcie-7v3:1ddr:3.0-0\|cnn\_GOOD | Read | OCL Region | 1 | 1 | 113429 | 113430 | 0.00333333 | 5273.140922 | 5273.140926 |
| 5273.140982 | Kernel\_Read\|xilinx:adm-pcie-7v3:1ddr:3.0-0\|cnn\_GOOD | Read | OCL Region | 1 | 1 | 113447 | 113448 | 0.00333333 | 5273.140982 | 5273.140986 |
| 5273.141049 | Kernel\_Read\|xilinx:adm-pcie-7v3:1ddr:3.0-0\|cnn\_GOOD | Read | OCL Region | 1 | 1 | 113467 | 113468 | 0.00333333 | 5273.141049 | 5273.141052 |
| 5273.141112 | Kernel\_Read\|xilinx:adm-pcie-7v3:1ddr:3.0-0\|cnn\_GOOD | Read | OCL Region | 1 | 1 | 113486 | 113487 | 0.00333333 | 5273.141112 | 5273.141116 |
| 5273.141182 | Kernel\_Read\|xilinx:adm-pcie-7v3:1ddr:3.0-0\|cnn\_GOOD | Read | OCL Region | 1 | 1 | 113507 | 113508 | 0.00333333 | 5273.141182 | 5273.141186 |
| 5273.141256 | Kernel\_Read\|xilinx:adm-pcie-7v3:1ddr:3.0-0\|cnn\_GOOD | Read | OCL Region | 1 | 1 | 113529 | 113530 | 0.00333333 | 5273.141256 | 5273.141259 |
| 5273.141312 | Kernel\_Read\|xilinx:adm-pcie-7v3:1ddr:3.0-0\|cnn\_GOOD | Read | OCL Region | 1 | 1 | 113546 | 113547 | 0.00333333 | 5273.141312 | 5273.141316 |
| 5273.141382 | Kernel\_Read\|xilinx:adm-pcie-7v3:1ddr:3.0-0\|cnn\_GOOD | Read | OCL Region | 1 | 1 | 113567 | 113568 | 0.00333333 | 5273.141382 | 5273.141386 |
| 5273.141449 | Kernel\_Read\|xilinx:adm-pcie-7v3:1ddr:3.0-0\|cnn\_GOOD | Read | OCL Region | 1 | 1 | 113587 | 113588 | 0.00333333 | 5273.141449 | 5273.141452 |
| 5273.141522 | Kernel\_Read\|xilinx:adm-pcie-7v3:1ddr:3.0-0\|cnn\_GOOD | Read | OCL Region | 1 | 1 | 113609 | 113610 | 0.00333333 | 5273.141522 | 5273.141526 |
| 5273.141582 | Kernel\_Read\|xilinx:adm-pcie-7v3:1ddr:3.0-0\|cnn\_GOOD | Read | OCL Region | 1 | 1 | 113627 | 113628 | 0.00333333 | 5273.141582 | 5273.141586 |
| 5273.141649 | Kernel\_Read\|xilinx:adm-pcie-7v3:1ddr:3.0-0\|cnn\_GOOD | Read | OCL Region | 1 | 1 | 113647 | 113648 | 0.00333333 | 5273.141649 | 5273.141652 |
| 5273.141712 | Kernel\_Read\|xilinx:adm-pcie-7v3:1ddr:3.0-0\|cnn\_GOOD | Read | OCL Region | 1 | 1 | 113666 | 113667 | 0.00333333 | 5273.141712 | 5273.141716 |
| 5273.141782 | Kernel\_Read\|xilinx:adm-pcie-7v3:1ddr:3.0-0\|cnn\_GOOD | Read | OCL Region | 1 | 1 | 113687 | 113688 | 0.00333333 | 5273.141782 | 5273.141786 |
| 5273.141849 | Kernel\_Read\|xilinx:adm-pcie-7v3:1ddr:3.0-0\|cnn\_GOOD | Read | OCL Region | 1 | 1 | 113707 | 113708 | 0.00333333 | 5273.141849 | 5273.141852 |
| 5273.141912 | Kernel\_Read\|xilinx:adm-pcie-7v3:1ddr:3.0-0\|cnn\_GOOD | Read | OCL Region | 1 | 1 | 113726 | 113727 | 0.00333333 | 5273.141912 | 5273.141916 |
| 5273.141982 | Kernel\_Read\|xilinx:adm-pcie-7v3:1ddr:3.0-0\|cnn\_GOOD | Read | OCL Region | 1 | 1 | 113747 | 113748 | 0.00333333 | 5273.141982 | 5273.141986 |
| 5273.142049 | Kernel\_Read\|xilinx:adm-pcie-7v3:1ddr:3.0-0\|cnn\_GOOD | Read | OCL Region | 1 | 1 | 113767 | 113768 | 0.00333333 | 5273.142049 | 5273.142052 |
| 5273.142112 | Kernel\_Read\|xilinx:adm-pcie-7v3:1ddr:3.0-0\|cnn\_GOOD | Read | OCL Region | 1 | 1 | 113786 | 113787 | 0.00333333 | 5273.142112 | 5273.142116 |
| 5273.142182 | Kernel\_Read\|xilinx:adm-pcie-7v3:1ddr:3.0-0\|cnn\_GOOD | Read | OCL Region | 1 | 1 | 113807 | 113808 | 0.00333333 | 5273.142182 | 5273.142186 |
| 5273.142249 | Kernel\_Read\|xilinx:adm-pcie-7v3:1ddr:3.0-0\|cnn\_GOOD | Read | OCL Region | 1 | 1 | 113827 | 113828 | 0.00333333 | 5273.142249 | 5273.142252 |
| 5273.142312 | Kernel\_Read\|xilinx:adm-pcie-7v3:1ddr:3.0-0\|cnn\_GOOD | Read | OCL Region | 1 | 1 | 113846 | 113847 | 0.00333333 | 5273.142312 | 5273.142316 |
| 5273.142382 | Kernel\_Read\|xilinx:adm-pcie-7v3:1ddr:3.0-0\|cnn\_GOOD | Read | OCL Region | 1 | 1 | 113867 | 113868 | 0.00333333 | 5273.142382 | 5273.142386 |
| 5273.142449 | Kernel\_Read\|xilinx:adm-pcie-7v3:1ddr:3.0-0\|cnn\_GOOD | Read | OCL Region | 1 | 1 | 113887 | 113888 | 0.00333333 | 5273.142449 | 5273.142452 |
| 5273.142512 | Kernel\_Read\|xilinx:adm-pcie-7v3:1ddr:3.0-0\|cnn\_GOOD | Read | OCL Region | 1 | 1 | 113906 | 113907 | 0.00333333 | 5273.142512 | 5273.142516 |
| 5273.142582 | Kernel\_Read\|xilinx:adm-pcie-7v3:1ddr:3.0-0\|cnn\_GOOD | Read | OCL Region | 1 | 1 | 113927 | 113928 | 0.00333333 | 5273.142582 | 5273.142586 |
| 5273.142649 | Kernel\_Read\|xilinx:adm-pcie-7v3:1ddr:3.0-0\|cnn\_GOOD | Read | OCL Region | 1 | 1 | 113947 | 113948 | 0.00333333 | 5273.142649 | 5273.142652 |
| 5273.142712 | Kernel\_Read\|xilinx:adm-pcie-7v3:1ddr:3.0-0\|cnn\_GOOD | Read | OCL Region | 1 | 1 | 113966 | 113967 | 0.00333333 | 5273.142712 | 5273.142716 |
| 5273.142782 | Kernel\_Read\|xilinx:adm-pcie-7v3:1ddr:3.0-0\|cnn\_GOOD | Read | OCL Region | 1 | 1 | 113987 | 113988 | 0.00333333 | 5273.142782 | 5273.142786 |
| 5273.142849 | Kernel\_Read\|xilinx:adm-pcie-7v3:1ddr:3.0-0\|cnn\_GOOD | Read | OCL Region | 1 | 1 | 114007 | 114008 | 0.00333333 | 5273.142849 | 5273.142852 |
| 5273.142912 | Kernel\_Read\|xilinx:adm-pcie-7v3:1ddr:3.0-0\|cnn\_GOOD | Read | OCL Region | 1 | 1 | 114026 | 114027 | 0.00333333 | 5273.142912 | 5273.142916 |
| 5273.142982 | Kernel\_Read\|xilinx:adm-pcie-7v3:1ddr:3.0-0\|cnn\_GOOD | Read | OCL Region | 1 | 1 | 114047 | 114048 | 0.00333333 | 5273.142982 | 5273.142986 |
| 5273.143049 | Kernel\_Read\|xilinx:adm-pcie-7v3:1ddr:3.0-0\|cnn\_GOOD | Read | OCL Region | 1 | 1 | 114067 | 114068 | 0.00333333 | 5273.143049 | 5273.143052 |
| 5273.143326 | Kernel\_Read\|xilinx:adm-pcie-7v3:1ddr:3.0-0\|cnn\_GOOD | Read | OCL Region | 1 | 1 | 114150 | 114151 | 0.00333333 | 5273.143326 | 5273.143329 |
| 5273.143329 | Kernel\_Read\|xilinx:adm-pcie-7v3:1ddr:3.0-0\|cnn\_GOOD | Read | OCL Region | 1 | 1 | 114151 | 114152 | 0.00333333 | 5273.143329 | 5273.143332 |
| 5273.143332 | Kernel\_Read\|xilinx:adm-pcie-7v3:1ddr:3.0-0\|cnn\_GOOD | Read | OCL Region | 1 | 1 | 114152 | 114153 | 0.00333333 | 5273.143332 | 5273.143336 |
| 5273.143339 | Kernel\_Read\|xilinx:adm-pcie-7v3:1ddr:3.0-0\|cnn\_GOOD | Read | OCL Region | 1 | 1 | 114154 | 114155 | 0.00333333 | 5273.143339 | 5273.143342 |
| 5273.143399 | Kernel\_Read\|xilinx:adm-pcie-7v3:1ddr:3.0-0\|cnn\_GOOD | Read | OCL Region | 1 | 1 | 114172 | 114173 | 0.00333333 | 5273.143399 | 5273.143402 |
| 5273.143456 | Kernel\_Read\|xilinx:adm-pcie-7v3:1ddr:3.0-0\|cnn\_GOOD | Read | OCL Region | 1 | 1 | 114189 | 114190 | 0.00333333 | 5273.143456 | 5273.143459 |
| 5273.143512 | Kernel\_Read\|xilinx:adm-pcie-7v3:1ddr:3.0-0\|cnn\_GOOD | Read | OCL Region | 1 | 1 | 114206 | 114207 | 0.00333333 | 5273.143512 | 5273.143516 |
| 5273.143582 | Kernel\_Read\|xilinx:adm-pcie-7v3:1ddr:3.0-0\|cnn\_GOOD | Read | OCL Region | 1 | 1 | 114227 | 114228 | 0.00333333 | 5273.143582 | 5273.143586 |
| 5273.143649 | Kernel\_Read\|xilinx:adm-pcie-7v3:1ddr:3.0-0\|cnn\_GOOD | Read | OCL Region | 1 | 1 | 114247 | 114248 | 0.00333333 | 5273.143649 | 5273.143652 |
| 5273.143712 | Kernel\_Read\|xilinx:adm-pcie-7v3:1ddr:3.0-0\|cnn\_GOOD | Read | OCL Region | 1 | 1 | 114266 | 114267 | 0.00333333 | 5273.143712 | 5273.143716 |
| 5273.143782 | Kernel\_Read\|xilinx:adm-pcie-7v3:1ddr:3.0-0\|cnn\_GOOD | Read | OCL Region | 1 | 1 | 114287 | 114288 | 0.00333333 | 5273.143782 | 5273.143786 |
| 5273.143849 | Kernel\_Read\|xilinx:adm-pcie-7v3:1ddr:3.0-0\|cnn\_GOOD | Read | OCL Region | 1 | 1 | 114307 | 114308 | 0.00333333 | 5273.143849 | 5273.143852 |
| 5273.143912 | Kernel\_Read\|xilinx:adm-pcie-7v3:1ddr:3.0-0\|cnn\_GOOD | Read | OCL Region | 1 | 1 | 114326 | 114327 | 0.00333333 | 5273.143912 | 5273.143916 |
| 5273.143982 | Kernel\_Read\|xilinx:adm-pcie-7v3:1ddr:3.0-0\|cnn\_GOOD | Read | OCL Region | 1 | 1 | 114347 | 114348 | 0.00333333 | 5273.143982 | 5273.143986 |
| 5273.144059 | Kernel\_Read\|xilinx:adm-pcie-7v3:1ddr:3.0-0\|cnn\_GOOD | Read | OCL Region | 1 | 1 | 114370 | 114371 | 0.00333333 | 5273.144059 | 5273.144062 |
| 5273.144112 | Kernel\_Read\|xilinx:adm-pcie-7v3:1ddr:3.0-0\|cnn\_GOOD | Read | OCL Region | 1 | 1 | 114386 | 114387 | 0.00333333 | 5273.144112 | 5273.144116 |
| 5273.144182 | Kernel\_Read\|xilinx:adm-pcie-7v3:1ddr:3.0-0\|cnn\_GOOD | Read | OCL Region | 1 | 1 | 114407 | 114408 | 0.00333333 | 5273.144182 | 5273.144186 |
| 5273.144249 | Kernel\_Read\|xilinx:adm-pcie-7v3:1ddr:3.0-0\|cnn\_GOOD | Read | OCL Region | 1 | 1 | 114427 | 114428 | 0.00333333 | 5273.144249 | 5273.144252 |
| 5273.144312 | Kernel\_Read\|xilinx:adm-pcie-7v3:1ddr:3.0-0\|cnn\_GOOD | Read | OCL Region | 1 | 1 | 114446 | 114447 | 0.00333333 | 5273.144312 | 5273.144316 |
| 5273.144382 | Kernel\_Read\|xilinx:adm-pcie-7v3:1ddr:3.0-0\|cnn\_GOOD | Read | OCL Region | 1 | 1 | 114467 | 114468 | 0.00333333 | 5273.144382 | 5273.144386 |
| 5273.144449 | Kernel\_Read\|xilinx:adm-pcie-7v3:1ddr:3.0-0\|cnn\_GOOD | Read | OCL Region | 1 | 1 | 114487 | 114488 | 0.00333333 | 5273.144449 | 5273.144452 |
| 5273.144512 | Kernel\_Read\|xilinx:adm-pcie-7v3:1ddr:3.0-0\|cnn\_GOOD | Read | OCL Region | 1 | 1 | 114506 | 114507 | 0.00333333 | 5273.144512 | 5273.144516 |
| 5273.144582 | Kernel\_Read\|xilinx:adm-pcie-7v3:1ddr:3.0-0\|cnn\_GOOD | Read | OCL Region | 1 | 1 | 114527 | 114528 | 0.00333333 | 5273.144582 | 5273.144586 |
| 5273.144649 | Kernel\_Read\|xilinx:adm-pcie-7v3:1ddr:3.0-0\|cnn\_GOOD | Read | OCL Region | 1 | 1 | 114547 | 114548 | 0.00333333 | 5273.144649 | 5273.144652 |
| 5273.144712 | Kernel\_Read\|xilinx:adm-pcie-7v3:1ddr:3.0-0\|cnn\_GOOD | Read | OCL Region | 1 | 1 | 114566 | 114567 | 0.00333333 | 5273.144712 | 5273.144716 |
| 5273.144782 | Kernel\_Read\|xilinx:adm-pcie-7v3:1ddr:3.0-0\|cnn\_GOOD | Read | OCL Region | 1 | 1 | 114587 | 114588 | 0.00333333 | 5273.144782 | 5273.144786 |
| 5273.144849 | Kernel\_Read\|xilinx:adm-pcie-7v3:1ddr:3.0-0\|cnn\_GOOD | Read | OCL Region | 1 | 1 | 114607 | 114608 | 0.00333333 | 5273.144849 | 5273.144852 |
| 5273.144922 | Kernel\_Read\|xilinx:adm-pcie-7v3:1ddr:3.0-0\|cnn\_GOOD | Read | OCL Region | 1 | 1 | 114629 | 114630 | 0.00333333 | 5273.144922 | 5273.144926 |
| 5273.144982 | Kernel\_Read\|xilinx:adm-pcie-7v3:1ddr:3.0-0\|cnn\_GOOD | Read | OCL Region | 1 | 1 | 114647 | 114648 | 0.00333333 | 5273.144982 | 5273.144986 |
| 5273.145049 | Kernel\_Read\|xilinx:adm-pcie-7v3:1ddr:3.0-0\|cnn\_GOOD | Read | OCL Region | 1 | 1 | 114667 | 114668 | 0.00333333 | 5273.145049 | 5273.145052 |
| 5273.145112 | Kernel\_Read\|xilinx:adm-pcie-7v3:1ddr:3.0-0\|cnn\_GOOD | Read | OCL Region | 1 | 1 | 114686 | 114687 | 0.00333333 | 5273.145112 | 5273.145116 |
| 5273.145182 | Kernel\_Read\|xilinx:adm-pcie-7v3:1ddr:3.0-0\|cnn\_GOOD | Read | OCL Region | 1 | 1 | 114707 | 114708 | 0.00333333 | 5273.145182 | 5273.145186 |
| 5273.145249 | Kernel\_Read\|xilinx:adm-pcie-7v3:1ddr:3.0-0\|cnn\_GOOD | Read | OCL Region | 1 | 1 | 114727 | 114728 | 0.00333333 | 5273.145249 | 5273.145252 |
| 5273.145316 | Kernel\_Read\|xilinx:adm-pcie-7v3:1ddr:3.0-0\|cnn\_GOOD | Read | OCL Region | 1 | 1 | 114747 | 114748 | 0.00333333 | 5273.145316 | 5273.145319 |
| 5273.145382 | Kernel\_Read\|xilinx:adm-pcie-7v3:1ddr:3.0-0\|cnn\_GOOD | Read | OCL Region | 1 | 1 | 114767 | 114768 | 0.00333333 | 5273.145382 | 5273.145386 |
| 5273.145449 | Kernel\_Read\|xilinx:adm-pcie-7v3:1ddr:3.0-0\|cnn\_GOOD | Read | OCL Region | 1 | 1 | 114787 | 114788 | 0.00333333 | 5273.145449 | 5273.145452 |
| 5273.145512 | Kernel\_Read\|xilinx:adm-pcie-7v3:1ddr:3.0-0\|cnn\_GOOD | Read | OCL Region | 1 | 1 | 114806 | 114807 | 0.00333333 | 5273.145512 | 5273.145516 |
| 5273.145582 | Kernel\_Read\|xilinx:adm-pcie-7v3:1ddr:3.0-0\|cnn\_GOOD | Read | OCL Region | 1 | 1 | 114827 | 114828 | 0.00333333 | 5273.145582 | 5273.145586 |
| 5273.145649 | Kernel\_Read\|xilinx:adm-pcie-7v3:1ddr:3.0-0\|cnn\_GOOD | Read | OCL Region | 1 | 1 | 114847 | 114848 | 0.00333333 | 5273.145649 | 5273.145652 |
| 5273.145712 | Kernel\_Read\|xilinx:adm-pcie-7v3:1ddr:3.0-0\|cnn\_GOOD | Read | OCL Region | 1 | 1 | 114866 | 114867 | 0.00333333 | 5273.145712 | 5273.145716 |
| 5273.145782 | Kernel\_Read\|xilinx:adm-pcie-7v3:1ddr:3.0-0\|cnn\_GOOD | Read | OCL Region | 1 | 1 | 114887 | 114888 | 0.00333333 | 5273.145782 | 5273.145786 |
| 5273.145849 | Kernel\_Read\|xilinx:adm-pcie-7v3:1ddr:3.0-0\|cnn\_GOOD | Read | OCL Region | 1 | 1 | 114907 | 114908 | 0.00333333 | 5273.145849 | 5273.145852 |
| 5273.145912 | Kernel\_Read\|xilinx:adm-pcie-7v3:1ddr:3.0-0\|cnn\_GOOD | Read | OCL Region | 1 | 1 | 114926 | 114927 | 0.00333333 | 5273.145912 | 5273.145916 |
| 5273.145982 | Kernel\_Read\|xilinx:adm-pcie-7v3:1ddr:3.0-0\|cnn\_GOOD | Read | OCL Region | 1 | 1 | 114947 | 114948 | 0.00333333 | 5273.145982 | 5273.145986 |
| 5273.146049 | Kernel\_Read\|xilinx:adm-pcie-7v3:1ddr:3.0-0\|cnn\_GOOD | Read | OCL Region | 1 | 1 | 114967 | 114968 | 0.00333333 | 5273.146049 | 5273.146052 |
| 5273.146112 | Kernel\_Read\|xilinx:adm-pcie-7v3:1ddr:3.0-0\|cnn\_GOOD | Read | OCL Region | 1 | 1 | 114986 | 114987 | 0.00333333 | 5273.146112 | 5273.146116 |
| 5273.146182 | Kernel\_Read\|xilinx:adm-pcie-7v3:1ddr:3.0-0\|cnn\_GOOD | Read | OCL Region | 1 | 1 | 115007 | 115008 | 0.00333333 | 5273.146182 | 5273.146186 |
| 5273.146249 | Kernel\_Read\|xilinx:adm-pcie-7v3:1ddr:3.0-0\|cnn\_GOOD | Read | OCL Region | 1 | 1 | 115027 | 115028 | 0.00333333 | 5273.146249 | 5273.146252 |
| 5273.146312 | Kernel\_Read\|xilinx:adm-pcie-7v3:1ddr:3.0-0\|cnn\_GOOD | Read | OCL Region | 1 | 1 | 115046 | 115047 | 0.00333333 | 5273.146312 | 5273.146316 |
| 5273.146382 | Kernel\_Read\|xilinx:adm-pcie-7v3:1ddr:3.0-0\|cnn\_GOOD | Read | OCL Region | 1 | 1 | 115067 | 115068 | 0.00333333 | 5273.146382 | 5273.146386 |
| 5273.146449 | Kernel\_Read\|xilinx:adm-pcie-7v3:1ddr:3.0-0\|cnn\_GOOD | Read | OCL Region | 1 | 1 | 115087 | 115088 | 0.00333333 | 5273.146449 | 5273.146452 |
| 5273.146512 | Kernel\_Read\|xilinx:adm-pcie-7v3:1ddr:3.0-0\|cnn\_GOOD | Read | OCL Region | 1 | 1 | 115106 | 115107 | 0.00333333 | 5273.146512 | 5273.146516 |
| 5273.146582 | Kernel\_Read\|xilinx:adm-pcie-7v3:1ddr:3.0-0\|cnn\_GOOD | Read | OCL Region | 1 | 1 | 115127 | 115128 | 0.00333333 | 5273.146582 | 5273.146586 |
| 5273.146649 | Kernel\_Read\|xilinx:adm-pcie-7v3:1ddr:3.0-0\|cnn\_GOOD | Read | OCL Region | 1 | 1 | 115147 | 115148 | 0.00333333 | 5273.146649 | 5273.146652 |
| 5273.146712 | Kernel\_Read\|xilinx:adm-pcie-7v3:1ddr:3.0-0\|cnn\_GOOD | Read | OCL Region | 1 | 1 | 115166 | 115167 | 0.00333333 | 5273.146712 | 5273.146716 |
| 5273.146782 | Kernel\_Read\|xilinx:adm-pcie-7v3:1ddr:3.0-0\|cnn\_GOOD | Read | OCL Region | 1 | 1 | 115187 | 115188 | 0.00333333 | 5273.146782 | 5273.146786 |
| 5273.146849 | Kernel\_Read\|xilinx:adm-pcie-7v3:1ddr:3.0-0\|cnn\_GOOD | Read | OCL Region | 1 | 1 | 115207 | 115208 | 0.00333333 | 5273.146849 | 5273.146852 |
| 5273.146916 | Kernel\_Read\|xilinx:adm-pcie-7v3:1ddr:3.0-0\|cnn\_GOOD | Read | OCL Region | 1 | 1 | 115227 | 115228 | 0.00333333 | 5273.146916 | 5273.146919 |
| 5273.146982 | Kernel\_Read\|xilinx:adm-pcie-7v3:1ddr:3.0-0\|cnn\_GOOD | Read | OCL Region | 1 | 1 | 115247 | 115248 | 0.00333333 | 5273.146982 | 5273.146986 |
| 5273.147049 | Kernel\_Read\|xilinx:adm-pcie-7v3:1ddr:3.0-0\|cnn\_GOOD | Read | OCL Region | 1 | 1 | 115267 | 115268 | 0.00333333 | 5273.147049 | 5273.147052 |
| 5273.147112 | Kernel\_Read\|xilinx:adm-pcie-7v3:1ddr:3.0-0\|cnn\_GOOD | Read | OCL Region | 1 | 1 | 115286 | 115287 | 0.00333333 | 5273.147112 | 5273.147116 |
| 5273.147182 | Kernel\_Read\|xilinx:adm-pcie-7v3:1ddr:3.0-0\|cnn\_GOOD | Read | OCL Region | 1 | 1 | 115307 | 115308 | 0.00333333 | 5273.147182 | 5273.147186 |
| 5273.147249 | Kernel\_Read\|xilinx:adm-pcie-7v3:1ddr:3.0-0\|cnn\_GOOD | Read | OCL Region | 1 | 1 | 115327 | 115328 | 0.00333333 | 5273.147249 | 5273.147252 |
| 5273.147312 | Kernel\_Read\|xilinx:adm-pcie-7v3:1ddr:3.0-0\|cnn\_GOOD | Read | OCL Region | 1 | 1 | 115346 | 115347 | 0.00333333 | 5273.147312 | 5273.147316 |
| 5273.147396 | Kernel\_Read\|xilinx:adm-pcie-7v3:1ddr:3.0-0\|cnn\_GOOD | Read | OCL Region | 1 | 1 | 115371 | 115372 | 0.00333333 | 5273.147396 | 5273.147399 |
| 5273.147449 | Kernel\_Read\|xilinx:adm-pcie-7v3:1ddr:3.0-0\|cnn\_GOOD | Read | OCL Region | 1 | 1 | 115387 | 115388 | 0.00333333 | 5273.147449 | 5273.147452 |
| 5273.147512 | Kernel\_Read\|xilinx:adm-pcie-7v3:1ddr:3.0-0\|cnn\_GOOD | Read | OCL Region | 1 | 1 | 115406 | 115407 | 0.00333333 | 5273.147512 | 5273.147516 |
| 5273.147582 | Kernel\_Read\|xilinx:adm-pcie-7v3:1ddr:3.0-0\|cnn\_GOOD | Read | OCL Region | 1 | 1 | 115427 | 115428 | 0.00333333 | 5273.147582 | 5273.147586 |
| 5273.147649 | Kernel\_Read\|xilinx:adm-pcie-7v3:1ddr:3.0-0\|cnn\_GOOD | Read | OCL Region | 1 | 1 | 115447 | 115448 | 0.00333333 | 5273.147649 | 5273.147652 |
| 5273.147716 | Kernel\_Read\|xilinx:adm-pcie-7v3:1ddr:3.0-0\|cnn\_GOOD | Read | OCL Region | 1 | 1 | 115467 | 115468 | 0.00333333 | 5273.147716 | 5273.147719 |
| 5273.147782 | Kernel\_Read\|xilinx:adm-pcie-7v3:1ddr:3.0-0\|cnn\_GOOD | Read | OCL Region | 1 | 1 | 115487 | 115488 | 0.00333333 | 5273.147782 | 5273.147786 |
| 5273.147849 | Kernel\_Read\|xilinx:adm-pcie-7v3:1ddr:3.0-0\|cnn\_GOOD | Read | OCL Region | 1 | 1 | 115507 | 115508 | 0.00333333 | 5273.147849 | 5273.147852 |
| 5273.147912 | Kernel\_Read\|xilinx:adm-pcie-7v3:1ddr:3.0-0\|cnn\_GOOD | Read | OCL Region | 1 | 1 | 115526 | 115527 | 0.00333333 | 5273.147912 | 5273.147916 |
| 5273.147982 | Kernel\_Read\|xilinx:adm-pcie-7v3:1ddr:3.0-0\|cnn\_GOOD | Read | OCL Region | 1 | 1 | 115547 | 115548 | 0.00333333 | 5273.147982 | 5273.147986 |
| 5273.148049 | Kernel\_Read\|xilinx:adm-pcie-7v3:1ddr:3.0-0\|cnn\_GOOD | Read | OCL Region | 1 | 1 | 115567 | 115568 | 0.00333333 | 5273.148049 | 5273.148052 |
| 5273.148112 | Kernel\_Read\|xilinx:adm-pcie-7v3:1ddr:3.0-0\|cnn\_GOOD | Read | OCL Region | 1 | 1 | 115586 | 115587 | 0.00333333 | 5273.148112 | 5273.148116 |
| 5273.148182 | Kernel\_Read\|xilinx:adm-pcie-7v3:1ddr:3.0-0\|cnn\_GOOD | Read | OCL Region | 1 | 1 | 115607 | 115608 | 0.00333333 | 5273.148182 | 5273.148186 |
| 5273.148249 | Kernel\_Read\|xilinx:adm-pcie-7v3:1ddr:3.0-0\|cnn\_GOOD | Read | OCL Region | 1 | 1 | 115627 | 115628 | 0.00333333 | 5273.148249 | 5273.148252 |
| 5273.148312 | Kernel\_Read\|xilinx:adm-pcie-7v3:1ddr:3.0-0\|cnn\_GOOD | Read | OCL Region | 1 | 1 | 115646 | 115647 | 0.00333333 | 5273.148312 | 5273.148316 |
| 5273.148382 | Kernel\_Read\|xilinx:adm-pcie-7v3:1ddr:3.0-0\|cnn\_GOOD | Read | OCL Region | 1 | 1 | 115667 | 115668 | 0.00333333 | 5273.148382 | 5273.148386 |
| 5273.148449 | Kernel\_Read\|xilinx:adm-pcie-7v3:1ddr:3.0-0\|cnn\_GOOD | Read | OCL Region | 1 | 1 | 115687 | 115688 | 0.00333333 | 5273.148449 | 5273.148452 |
| 5273.148516 | Kernel\_Read\|xilinx:adm-pcie-7v3:1ddr:3.0-0\|cnn\_GOOD | Read | OCL Region | 1 | 1 | 115707 | 115708 | 0.00333333 | 5273.148516 | 5273.148519 |
| 5273.148582 | Kernel\_Read\|xilinx:adm-pcie-7v3:1ddr:3.0-0\|cnn\_GOOD | Read | OCL Region | 1 | 1 | 115727 | 115728 | 0.00333333 | 5273.148582 | 5273.148586 |
| 5273.148649 | Kernel\_Read\|xilinx:adm-pcie-7v3:1ddr:3.0-0\|cnn\_GOOD | Read | OCL Region | 1 | 1 | 115747 | 115748 | 0.00333333 | 5273.148649 | 5273.148652 |
| 5273.148712 | Kernel\_Read\|xilinx:adm-pcie-7v3:1ddr:3.0-0\|cnn\_GOOD | Read | OCL Region | 1 | 1 | 115766 | 115767 | 0.00333333 | 5273.148712 | 5273.148716 |
| 5273.148782 | Kernel\_Read\|xilinx:adm-pcie-7v3:1ddr:3.0-0\|cnn\_GOOD | Read | OCL Region | 1 | 1 | 115787 | 115788 | 0.00333333 | 5273.148782 | 5273.148786 |
| 5273.148849 | Kernel\_Read\|xilinx:adm-pcie-7v3:1ddr:3.0-0\|cnn\_GOOD | Read | OCL Region | 1 | 1 | 115807 | 115808 | 0.00333333 | 5273.148849 | 5273.148852 |
| 5273.148916 | Kernel\_Read\|xilinx:adm-pcie-7v3:1ddr:3.0-0\|cnn\_GOOD | Read | OCL Region | 1 | 1 | 115827 | 115828 | 0.00333333 | 5273.148916 | 5273.148919 |
| 5273.148982 | Kernel\_Read\|xilinx:adm-pcie-7v3:1ddr:3.0-0\|cnn\_GOOD | Read | OCL Region | 1 | 1 | 115847 | 115848 | 0.00333333 | 5273.148982 | 5273.148986 |
| 5273.149049 | Kernel\_Read\|xilinx:adm-pcie-7v3:1ddr:3.0-0\|cnn\_GOOD | Read | OCL Region | 1 | 1 | 115867 | 115868 | 0.00333333 | 5273.149049 | 5273.149052 |
| 5273.149112 | Kernel\_Read\|xilinx:adm-pcie-7v3:1ddr:3.0-0\|cnn\_GOOD | Read | OCL Region | 1 | 1 | 115886 | 115887 | 0.00333333 | 5273.149112 | 5273.149116 |
| 5273.149182 | Kernel\_Read\|xilinx:adm-pcie-7v3:1ddr:3.0-0\|cnn\_GOOD | Read | OCL Region | 1 | 1 | 115907 | 115908 | 0.00333333 | 5273.149182 | 5273.149186 |
| 5273.149249 | Kernel\_Read\|xilinx:adm-pcie-7v3:1ddr:3.0-0\|cnn\_GOOD | Read | OCL Region | 1 | 1 | 115927 | 115928 | 0.00333333 | 5273.149249 | 5273.149252 |
| 5273.149316 | Kernel\_Read\|xilinx:adm-pcie-7v3:1ddr:3.0-0\|cnn\_GOOD | Read | OCL Region | 1 | 1 | 115947 | 115948 | 0.00333333 | 5273.149316 | 5273.149319 |
| 5273.149589 | Kernel\_Read\|xilinx:adm-pcie-7v3:1ddr:3.0-0\|cnn\_GOOD | Read | OCL Region | 1 | 1 | 116029 | 116030 | 0.00333333 | 5273.149589 | 5273.149592 |
| 5273.149596 | Kernel\_Read\|xilinx:adm-pcie-7v3:1ddr:3.0-0\|cnn\_GOOD | Read | OCL Region | 1 | 1 | 116031 | 116032 | 0.00333333 | 5273.149596 | 5273.149599 |
| 5273.149599 | Kernel\_Read\|xilinx:adm-pcie-7v3:1ddr:3.0-0\|cnn\_GOOD | Read | OCL Region | 1 | 1 | 116032 | 116033 | 0.00333333 | 5273.149599 | 5273.149602 |
| 5273.149606 | Kernel\_Read\|xilinx:adm-pcie-7v3:1ddr:3.0-0\|cnn\_GOOD | Read | OCL Region | 1 | 1 | 116034 | 116035 | 0.00333333 | 5273.149606 | 5273.149609 |
| 5273.149662 | Kernel\_Read\|xilinx:adm-pcie-7v3:1ddr:3.0-0\|cnn\_GOOD | Read | OCL Region | 1 | 1 | 116051 | 116052 | 0.00333333 | 5273.149662 | 5273.149666 |
| 5273.149722 | Kernel\_Read\|xilinx:adm-pcie-7v3:1ddr:3.0-0\|cnn\_GOOD | Read | OCL Region | 1 | 1 | 116069 | 116070 | 0.00333333 | 5273.149722 | 5273.149726 |
| 5273.149782 | Kernel\_Read\|xilinx:adm-pcie-7v3:1ddr:3.0-0\|cnn\_GOOD | Read | OCL Region | 1 | 1 | 116087 | 116088 | 0.00333333 | 5273.149782 | 5273.149786 |
| 5273.149849 | Kernel\_Read\|xilinx:adm-pcie-7v3:1ddr:3.0-0\|cnn\_GOOD | Read | OCL Region | 1 | 1 | 116107 | 116108 | 0.00333333 | 5273.149849 | 5273.149852 |
| 5273.149912 | Kernel\_Read\|xilinx:adm-pcie-7v3:1ddr:3.0-0\|cnn\_GOOD | Read | OCL Region | 1 | 1 | 116126 | 116127 | 0.00333333 | 5273.149912 | 5273.149916 |
| 5273.149982 | Kernel\_Read\|xilinx:adm-pcie-7v3:1ddr:3.0-0\|cnn\_GOOD | Read | OCL Region | 1 | 1 | 116147 | 116148 | 0.00333333 | 5273.149982 | 5273.149986 |
| 5273.150049 | Kernel\_Read\|xilinx:adm-pcie-7v3:1ddr:3.0-0\|cnn\_GOOD | Read | OCL Region | 1 | 1 | 116167 | 116168 | 0.00333333 | 5273.150049 | 5273.150052 |
| 5273.150116 | Kernel\_Read\|xilinx:adm-pcie-7v3:1ddr:3.0-0\|cnn\_GOOD | Read | OCL Region | 1 | 1 | 116187 | 116188 | 0.00333333 | 5273.150116 | 5273.150119 |
| 5273.150182 | Kernel\_Read\|xilinx:adm-pcie-7v3:1ddr:3.0-0\|cnn\_GOOD | Read | OCL Region | 1 | 1 | 116207 | 116208 | 0.00333333 | 5273.150182 | 5273.150186 |
| 5273.150249 | Kernel\_Read\|xilinx:adm-pcie-7v3:1ddr:3.0-0\|cnn\_GOOD | Read | OCL Region | 1 | 1 | 116227 | 116228 | 0.00333333 | 5273.150249 | 5273.150252 |
| 5273.150312 | Kernel\_Read\|xilinx:adm-pcie-7v3:1ddr:3.0-0\|cnn\_GOOD | Read | OCL Region | 1 | 1 | 116246 | 116247 | 0.00333333 | 5273.150312 | 5273.150316 |
| 5273.150382 | Kernel\_Read\|xilinx:adm-pcie-7v3:1ddr:3.0-0\|cnn\_GOOD | Read | OCL Region | 1 | 1 | 116267 | 116268 | 0.00333333 | 5273.150382 | 5273.150386 |
| 5273.150449 | Kernel\_Read\|xilinx:adm-pcie-7v3:1ddr:3.0-0\|cnn\_GOOD | Read | OCL Region | 1 | 1 | 116287 | 116288 | 0.00333333 | 5273.150449 | 5273.150452 |
| 5273.150516 | Kernel\_Read\|xilinx:adm-pcie-7v3:1ddr:3.0-0\|cnn\_GOOD | Read | OCL Region | 1 | 1 | 116307 | 116308 | 0.00333333 | 5273.150516 | 5273.150519 |
| 5273.150582 | Kernel\_Read\|xilinx:adm-pcie-7v3:1ddr:3.0-0\|cnn\_GOOD | Read | OCL Region | 1 | 1 | 116327 | 116328 | 0.00333333 | 5273.150582 | 5273.150586 |
| 5273.150649 | Kernel\_Read\|xilinx:adm-pcie-7v3:1ddr:3.0-0\|cnn\_GOOD | Read | OCL Region | 1 | 1 | 116347 | 116348 | 0.00333333 | 5273.150649 | 5273.150652 |
| 5273.150712 | Kernel\_Read\|xilinx:adm-pcie-7v3:1ddr:3.0-0\|cnn\_GOOD | Read | OCL Region | 1 | 1 | 116366 | 116367 | 0.00333333 | 5273.150712 | 5273.150716 |
| 5273.150782 | Kernel\_Read\|xilinx:adm-pcie-7v3:1ddr:3.0-0\|cnn\_GOOD | Read | OCL Region | 1 | 1 | 116387 | 116388 | 0.00333333 | 5273.150782 | 5273.150786 |
| 5273.150849 | Kernel\_Read\|xilinx:adm-pcie-7v3:1ddr:3.0-0\|cnn\_GOOD | Read | OCL Region | 1 | 1 | 116407 | 116408 | 0.00333333 | 5273.150849 | 5273.150852 |
| 5273.150916 | Kernel\_Read\|xilinx:adm-pcie-7v3:1ddr:3.0-0\|cnn\_GOOD | Read | OCL Region | 1 | 1 | 116427 | 116428 | 0.00333333 | 5273.150916 | 5273.150919 |
| 5273.150982 | Kernel\_Read\|xilinx:adm-pcie-7v3:1ddr:3.0-0\|cnn\_GOOD | Read | OCL Region | 1 | 1 | 116447 | 116448 | 0.00333333 | 5273.150982 | 5273.150986 |
| 5273.151049 | Kernel\_Read\|xilinx:adm-pcie-7v3:1ddr:3.0-0\|cnn\_GOOD | Read | OCL Region | 1 | 1 | 116467 | 116468 | 0.00333333 | 5273.151049 | 5273.151052 |
| 5273.151112 | Kernel\_Read\|xilinx:adm-pcie-7v3:1ddr:3.0-0\|cnn\_GOOD | Read | OCL Region | 1 | 1 | 116486 | 116487 | 0.00333333 | 5273.151112 | 5273.151116 |
| 5273.151192 | Kernel\_Read\|xilinx:adm-pcie-7v3:1ddr:3.0-0\|cnn\_GOOD | Read | OCL Region | 1 | 1 | 116510 | 116511 | 0.00333333 | 5273.151192 | 5273.151196 |
| 5273.151249 | Kernel\_Read\|xilinx:adm-pcie-7v3:1ddr:3.0-0\|cnn\_GOOD | Read | OCL Region | 1 | 1 | 116527 | 116528 | 0.00333333 | 5273.151249 | 5273.151252 |
| 5273.151312 | Kernel\_Read\|xilinx:adm-pcie-7v3:1ddr:3.0-0\|cnn\_GOOD | Read | OCL Region | 1 | 1 | 116546 | 116547 | 0.00333333 | 5273.151312 | 5273.151316 |
| 5273.151382 | Kernel\_Read\|xilinx:adm-pcie-7v3:1ddr:3.0-0\|cnn\_GOOD | Read | OCL Region | 1 | 1 | 116567 | 116568 | 0.00333333 | 5273.151382 | 5273.151386 |
| 5273.151449 | Kernel\_Read\|xilinx:adm-pcie-7v3:1ddr:3.0-0\|cnn\_GOOD | Read | OCL Region | 1 | 1 | 116587 | 116588 | 0.00333333 | 5273.151449 | 5273.151452 |
| 5273.151516 | Kernel\_Read\|xilinx:adm-pcie-7v3:1ddr:3.0-0\|cnn\_GOOD | Read | OCL Region | 1 | 1 | 116607 | 116608 | 0.00333333 | 5273.151516 | 5273.151519 |
| 5273.151582 | Kernel\_Read\|xilinx:adm-pcie-7v3:1ddr:3.0-0\|cnn\_GOOD | Read | OCL Region | 1 | 1 | 116627 | 116628 | 0.00333333 | 5273.151582 | 5273.151586 |
| 5273.151649 | Kernel\_Read\|xilinx:adm-pcie-7v3:1ddr:3.0-0\|cnn\_GOOD | Read | OCL Region | 1 | 1 | 116647 | 116648 | 0.00333333 | 5273.151649 | 5273.151652 |
| 5273.151716 | Kernel\_Read\|xilinx:adm-pcie-7v3:1ddr:3.0-0\|cnn\_GOOD | Read | OCL Region | 1 | 1 | 116667 | 116668 | 0.00333333 | 5273.151716 | 5273.151719 |
| 5273.151782 | Kernel\_Read\|xilinx:adm-pcie-7v3:1ddr:3.0-0\|cnn\_GOOD | Read | OCL Region | 1 | 1 | 116687 | 116688 | 0.00333333 | 5273.151782 | 5273.151786 |
| 5273.151849 | Kernel\_Read\|xilinx:adm-pcie-7v3:1ddr:3.0-0\|cnn\_GOOD | Read | OCL Region | 1 | 1 | 116707 | 116708 | 0.00333333 | 5273.151849 | 5273.151852 |
| 5273.151912 | Kernel\_Read\|xilinx:adm-pcie-7v3:1ddr:3.0-0\|cnn\_GOOD | Read | OCL Region | 1 | 1 | 116726 | 116727 | 0.00333333 | 5273.151912 | 5273.151916 |
| 5273.151979 | Kernel\_Read\|xilinx:adm-pcie-7v3:1ddr:3.0-0\|cnn\_GOOD | Read | OCL Region | 1 | 1 | 116746 | 116747 | 0.00333333 | 5273.151979 | 5273.151982 |
| 5273.152049 | Kernel\_Read\|xilinx:adm-pcie-7v3:1ddr:3.0-0\|cnn\_GOOD | Read | OCL Region | 1 | 1 | 116767 | 116768 | 0.00333333 | 5273.152049 | 5273.152052 |
| 5273.152116 | Kernel\_Read\|xilinx:adm-pcie-7v3:1ddr:3.0-0\|cnn\_GOOD | Read | OCL Region | 1 | 1 | 116787 | 116788 | 0.00333333 | 5273.152116 | 5273.152119 |
| 5273.152182 | Kernel\_Read\|xilinx:adm-pcie-7v3:1ddr:3.0-0\|cnn\_GOOD | Read | OCL Region | 1 | 1 | 116807 | 116808 | 0.00333333 | 5273.152182 | 5273.152186 |
| 5273.152249 | Kernel\_Read\|xilinx:adm-pcie-7v3:1ddr:3.0-0\|cnn\_GOOD | Read | OCL Region | 1 | 1 | 116827 | 116828 | 0.00333333 | 5273.152249 | 5273.152252 |
| 5273.152316 | Kernel\_Read\|xilinx:adm-pcie-7v3:1ddr:3.0-0\|cnn\_GOOD | Read | OCL Region | 1 | 1 | 116847 | 116848 | 0.00333333 | 5273.152316 | 5273.152319 |
| 5273.152382 | Kernel\_Read\|xilinx:adm-pcie-7v3:1ddr:3.0-0\|cnn\_GOOD | Read | OCL Region | 1 | 1 | 116867 | 116868 | 0.00333333 | 5273.152382 | 5273.152386 |
| 5273.152449 | Kernel\_Read\|xilinx:adm-pcie-7v3:1ddr:3.0-0\|cnn\_GOOD | Read | OCL Region | 1 | 1 | 116887 | 116888 | 0.00333333 | 5273.152449 | 5273.152452 |
| 5273.152516 | Kernel\_Read\|xilinx:adm-pcie-7v3:1ddr:3.0-0\|cnn\_GOOD | Read | OCL Region | 1 | 1 | 116907 | 116908 | 0.00333333 | 5273.152516 | 5273.152519 |
| 5273.152582 | Kernel\_Read\|xilinx:adm-pcie-7v3:1ddr:3.0-0\|cnn\_GOOD | Read | OCL Region | 1 | 1 | 116927 | 116928 | 0.00333333 | 5273.152582 | 5273.152586 |
| 5273.152649 | Kernel\_Read\|xilinx:adm-pcie-7v3:1ddr:3.0-0\|cnn\_GOOD | Read | OCL Region | 1 | 1 | 116947 | 116948 | 0.00333333 | 5273.152649 | 5273.152652 |
| 5273.152716 | Kernel\_Read\|xilinx:adm-pcie-7v3:1ddr:3.0-0\|cnn\_GOOD | Read | OCL Region | 1 | 1 | 116967 | 116968 | 0.00333333 | 5273.152716 | 5273.152719 |
| 5273.152782 | Kernel\_Read\|xilinx:adm-pcie-7v3:1ddr:3.0-0\|cnn\_GOOD | Read | OCL Region | 1 | 1 | 116987 | 116988 | 0.00333333 | 5273.152782 | 5273.152786 |
| 5273.152849 | Kernel\_Read\|xilinx:adm-pcie-7v3:1ddr:3.0-0\|cnn\_GOOD | Read | OCL Region | 1 | 1 | 117007 | 117008 | 0.00333333 | 5273.152849 | 5273.152852 |
| 5273.152912 | Kernel\_Read\|xilinx:adm-pcie-7v3:1ddr:3.0-0\|cnn\_GOOD | Read | OCL Region | 1 | 1 | 117026 | 117027 | 0.00333333 | 5273.152912 | 5273.152916 |
| 5273.152982 | Kernel\_Read\|xilinx:adm-pcie-7v3:1ddr:3.0-0\|cnn\_GOOD | Read | OCL Region | 1 | 1 | 117047 | 117048 | 0.00333333 | 5273.152982 | 5273.152986 |
| 5273.153049 | Kernel\_Read\|xilinx:adm-pcie-7v3:1ddr:3.0-0\|cnn\_GOOD | Read | OCL Region | 1 | 1 | 117067 | 117068 | 0.00333333 | 5273.153049 | 5273.153052 |
| 5273.153116 | Kernel\_Read\|xilinx:adm-pcie-7v3:1ddr:3.0-0\|cnn\_GOOD | Read | OCL Region | 1 | 1 | 117087 | 117088 | 0.00333333 | 5273.153116 | 5273.153119 |
| 5273.153182 | Kernel\_Read\|xilinx:adm-pcie-7v3:1ddr:3.0-0\|cnn\_GOOD | Read | OCL Region | 1 | 1 | 117107 | 117108 | 0.00333333 | 5273.153182 | 5273.153186 |
| 5273.153249 | Kernel\_Read\|xilinx:adm-pcie-7v3:1ddr:3.0-0\|cnn\_GOOD | Read | OCL Region | 1 | 1 | 117127 | 117128 | 0.00333333 | 5273.153249 | 5273.153252 |
| 5273.153316 | Kernel\_Read\|xilinx:adm-pcie-7v3:1ddr:3.0-0\|cnn\_GOOD | Read | OCL Region | 1 | 1 | 117147 | 117148 | 0.00333333 | 5273.153316 | 5273.153319 |
| 5273.153382 | Kernel\_Read\|xilinx:adm-pcie-7v3:1ddr:3.0-0\|cnn\_GOOD | Read | OCL Region | 1 | 1 | 117167 | 117168 | 0.00333333 | 5273.153382 | 5273.153386 |
| 5273.153449 | Kernel\_Read\|xilinx:adm-pcie-7v3:1ddr:3.0-0\|cnn\_GOOD | Read | OCL Region | 1 | 1 | 117187 | 117188 | 0.00333333 | 5273.153449 | 5273.153452 |
| 5273.153512 | Kernel\_Read\|xilinx:adm-pcie-7v3:1ddr:3.0-0\|cnn\_GOOD | Read | OCL Region | 1 | 1 | 117206 | 117207 | 0.00333333 | 5273.153512 | 5273.153516 |
| 5273.153582 | Kernel\_Read\|xilinx:adm-pcie-7v3:1ddr:3.0-0\|cnn\_GOOD | Read | OCL Region | 1 | 1 | 117227 | 117228 | 0.00333333 | 5273.153582 | 5273.153586 |
| 5273.153666 | Kernel\_Read\|xilinx:adm-pcie-7v3:1ddr:3.0-0\|cnn\_GOOD | Read | OCL Region | 1 | 1 | 117252 | 117253 | 0.00333333 | 5273.153666 | 5273.153669 |
| 5273.153722 | Kernel\_Read\|xilinx:adm-pcie-7v3:1ddr:3.0-0\|cnn\_GOOD | Read | OCL Region | 1 | 1 | 117269 | 117270 | 0.00333333 | 5273.153722 | 5273.153726 |
| 5273.153782 | Kernel\_Read\|xilinx:adm-pcie-7v3:1ddr:3.0-0\|cnn\_GOOD | Read | OCL Region | 1 | 1 | 117287 | 117288 | 0.00333333 | 5273.153782 | 5273.153786 |
| 5273.153849 | Kernel\_Read\|xilinx:adm-pcie-7v3:1ddr:3.0-0\|cnn\_GOOD | Read | OCL Region | 1 | 1 | 117307 | 117308 | 0.00333333 | 5273.153849 | 5273.153852 |
| 5273.153916 | Kernel\_Read\|xilinx:adm-pcie-7v3:1ddr:3.0-0\|cnn\_GOOD | Read | OCL Region | 1 | 1 | 117327 | 117328 | 0.00333333 | 5273.153916 | 5273.153919 |
| 5273.153982 | Kernel\_Read\|xilinx:adm-pcie-7v3:1ddr:3.0-0\|cnn\_GOOD | Read | OCL Region | 1 | 1 | 117347 | 117348 | 0.00333333 | 5273.153982 | 5273.153986 |
| 5273.154049 | Kernel\_Read\|xilinx:adm-pcie-7v3:1ddr:3.0-0\|cnn\_GOOD | Read | OCL Region | 1 | 1 | 117367 | 117368 | 0.00333333 | 5273.154049 | 5273.154052 |
| 5273.154116 | Kernel\_Read\|xilinx:adm-pcie-7v3:1ddr:3.0-0\|cnn\_GOOD | Read | OCL Region | 1 | 1 | 117387 | 117388 | 0.00333333 | 5273.154116 | 5273.154119 |
| 5273.154182 | Kernel\_Read\|xilinx:adm-pcie-7v3:1ddr:3.0-0\|cnn\_GOOD | Read | OCL Region | 1 | 1 | 117407 | 117408 | 0.00333333 | 5273.154182 | 5273.154186 |
| 5273.154249 | Kernel\_Read\|xilinx:adm-pcie-7v3:1ddr:3.0-0\|cnn\_GOOD | Read | OCL Region | 1 | 1 | 117427 | 117428 | 0.00333333 | 5273.154249 | 5273.154252 |
| 5273.154316 | Kernel\_Read\|xilinx:adm-pcie-7v3:1ddr:3.0-0\|cnn\_GOOD | Read | OCL Region | 1 | 1 | 117447 | 117448 | 0.00333333 | 5273.154316 | 5273.154319 |
| 5273.154382 | Kernel\_Read\|xilinx:adm-pcie-7v3:1ddr:3.0-0\|cnn\_GOOD | Read | OCL Region | 1 | 1 | 117467 | 117468 | 0.00333333 | 5273.154382 | 5273.154386 |
| 5273.154456 | Kernel\_Read\|xilinx:adm-pcie-7v3:1ddr:3.0-0\|cnn\_GOOD | Read | OCL Region | 1 | 1 | 117489 | 117490 | 0.00333333 | 5273.154456 | 5273.154459 |
| 5273.154512 | Kernel\_Read\|xilinx:adm-pcie-7v3:1ddr:3.0-0\|cnn\_GOOD | Read | OCL Region | 1 | 1 | 117506 | 117507 | 0.00333333 | 5273.154512 | 5273.154516 |
| 5273.154582 | Kernel\_Read\|xilinx:adm-pcie-7v3:1ddr:3.0-0\|cnn\_GOOD | Read | OCL Region | 1 | 1 | 117527 | 117528 | 0.00333333 | 5273.154582 | 5273.154586 |
| 5273.154649 | Kernel\_Read\|xilinx:adm-pcie-7v3:1ddr:3.0-0\|cnn\_GOOD | Read | OCL Region | 1 | 1 | 117547 | 117548 | 0.00333333 | 5273.154649 | 5273.154652 |
| 5273.154716 | Kernel\_Read\|xilinx:adm-pcie-7v3:1ddr:3.0-0\|cnn\_GOOD | Read | OCL Region | 1 | 1 | 117567 | 117568 | 0.00333333 | 5273.154716 | 5273.154719 |
| 5273.154782 | Kernel\_Read\|xilinx:adm-pcie-7v3:1ddr:3.0-0\|cnn\_GOOD | Read | OCL Region | 1 | 1 | 117587 | 117588 | 0.00333333 | 5273.154782 | 5273.154786 |
| 5273.154849 | Kernel\_Read\|xilinx:adm-pcie-7v3:1ddr:3.0-0\|cnn\_GOOD | Read | OCL Region | 1 | 1 | 117607 | 117608 | 0.00333333 | 5273.154849 | 5273.154852 |
| 5273.154916 | Kernel\_Read\|xilinx:adm-pcie-7v3:1ddr:3.0-0\|cnn\_GOOD | Read | OCL Region | 1 | 1 | 117627 | 117628 | 0.00333333 | 5273.154916 | 5273.154919 |
| 5273.154982 | Kernel\_Read\|xilinx:adm-pcie-7v3:1ddr:3.0-0\|cnn\_GOOD | Read | OCL Region | 1 | 1 | 117647 | 117648 | 0.00333333 | 5273.154982 | 5273.154986 |
| 5273.155049 | Kernel\_Read\|xilinx:adm-pcie-7v3:1ddr:3.0-0\|cnn\_GOOD | Read | OCL Region | 1 | 1 | 117667 | 117668 | 0.00333333 | 5273.155049 | 5273.155052 |
| 5273.155116 | Kernel\_Read\|xilinx:adm-pcie-7v3:1ddr:3.0-0\|cnn\_GOOD | Read | OCL Region | 1 | 1 | 117687 | 117688 | 0.00333333 | 5273.155116 | 5273.155119 |
| 5273.155182 | Kernel\_Read\|xilinx:adm-pcie-7v3:1ddr:3.0-0\|cnn\_GOOD | Read | OCL Region | 1 | 1 | 117707 | 117708 | 0.00333333 | 5273.155182 | 5273.155186 |
| 5273.155249 | Kernel\_Read\|xilinx:adm-pcie-7v3:1ddr:3.0-0\|cnn\_GOOD | Read | OCL Region | 1 | 1 | 117727 | 117728 | 0.00333333 | 5273.155249 | 5273.155252 |
| 5273.155316 | Kernel\_Read\|xilinx:adm-pcie-7v3:1ddr:3.0-0\|cnn\_GOOD | Read | OCL Region | 1 | 1 | 117747 | 117748 | 0.00333333 | 5273.155316 | 5273.155319 |
| 5273.155382 | Kernel\_Read\|xilinx:adm-pcie-7v3:1ddr:3.0-0\|cnn\_GOOD | Read | OCL Region | 1 | 1 | 117767 | 117768 | 0.00333333 | 5273.155382 | 5273.155386 |
| 5273.155449 | Kernel\_Read\|xilinx:adm-pcie-7v3:1ddr:3.0-0\|cnn\_GOOD | Read | OCL Region | 1 | 1 | 117787 | 117788 | 0.00333333 | 5273.155449 | 5273.155452 |
| 5273.155516 | Kernel\_Read\|xilinx:adm-pcie-7v3:1ddr:3.0-0\|cnn\_GOOD | Read | OCL Region | 1 | 1 | 117807 | 117808 | 0.00333333 | 5273.155516 | 5273.155519 |
| 5273.155582 | Kernel\_Read\|xilinx:adm-pcie-7v3:1ddr:3.0-0\|cnn\_GOOD | Read | OCL Region | 1 | 1 | 117827 | 117828 | 0.00333333 | 5273.155582 | 5273.155586 |
| 5273.155859 | Kernel\_Read\|xilinx:adm-pcie-7v3:1ddr:3.0-0\|cnn\_GOOD | Read | OCL Region | 1 | 1 | 117910 | 117911 | 0.00333333 | 5273.155859 | 5273.155862 |
| 5273.155866 | Kernel\_Read\|xilinx:adm-pcie-7v3:1ddr:3.0-0\|cnn\_GOOD | Read | OCL Region | 1 | 1 | 117912 | 117913 | 0.00333333 | 5273.155866 | 5273.155869 |
| 5273.155872 | Kernel\_Read\|xilinx:adm-pcie-7v3:1ddr:3.0-0\|cnn\_GOOD | Read | OCL Region | 1 | 1 | 117914 | 117915 | 0.00333333 | 5273.155872 | 5273.155876 |
| 5273.155876 | Kernel\_Read\|xilinx:adm-pcie-7v3:1ddr:3.0-0\|cnn\_GOOD | Read | OCL Region | 1 | 1 | 117915 | 117916 | 0.00333333 | 5273.155876 | 5273.155879 |
| 5273.155932 | Kernel\_Read\|xilinx:adm-pcie-7v3:1ddr:3.0-0\|cnn\_GOOD | Read | OCL Region | 1 | 1 | 117932 | 117933 | 0.00333333 | 5273.155932 | 5273.155936 |
| 5273.155992 | Kernel\_Read\|xilinx:adm-pcie-7v3:1ddr:3.0-0\|cnn\_GOOD | Read | OCL Region | 1 | 1 | 117950 | 117951 | 0.00333333 | 5273.155992 | 5273.155996 |
| 5273.156049 | Kernel\_Read\|xilinx:adm-pcie-7v3:1ddr:3.0-0\|cnn\_GOOD | Read | OCL Region | 1 | 1 | 117967 | 117968 | 0.00333333 | 5273.156049 | 5273.156052 |
| 5273.156112 | Kernel\_Read\|xilinx:adm-pcie-7v3:1ddr:3.0-0\|cnn\_GOOD | Read | OCL Region | 1 | 1 | 117986 | 117987 | 0.00333333 | 5273.156112 | 5273.156116 |
| 5273.156182 | Kernel\_Read\|xilinx:adm-pcie-7v3:1ddr:3.0-0\|cnn\_GOOD | Read | OCL Region | 1 | 1 | 118007 | 118008 | 0.00333333 | 5273.156182 | 5273.156186 |
| 5273.156249 | Kernel\_Read\|xilinx:adm-pcie-7v3:1ddr:3.0-0\|cnn\_GOOD | Read | OCL Region | 1 | 1 | 118027 | 118028 | 0.00333333 | 5273.156249 | 5273.156252 |
| 5273.156312 | Kernel\_Read\|xilinx:adm-pcie-7v3:1ddr:3.0-0\|cnn\_GOOD | Read | OCL Region | 1 | 1 | 118046 | 118047 | 0.00333333 | 5273.156312 | 5273.156316 |
| 5273.156382 | Kernel\_Read\|xilinx:adm-pcie-7v3:1ddr:3.0-0\|cnn\_GOOD | Read | OCL Region | 1 | 1 | 118067 | 118068 | 0.00333333 | 5273.156382 | 5273.156386 |
| 5273.156449 | Kernel\_Read\|xilinx:adm-pcie-7v3:1ddr:3.0-0\|cnn\_GOOD | Read | OCL Region | 1 | 1 | 118087 | 118088 | 0.00333333 | 5273.156449 | 5273.156452 |
| 5273.156516 | Kernel\_Read\|xilinx:adm-pcie-7v3:1ddr:3.0-0\|cnn\_GOOD | Read | OCL Region | 1 | 1 | 118107 | 118108 | 0.00333333 | 5273.156516 | 5273.156519 |
| 5273.156582 | Kernel\_Read\|xilinx:adm-pcie-7v3:1ddr:3.0-0\|cnn\_GOOD | Read | OCL Region | 1 | 1 | 118127 | 118128 | 0.00333333 | 5273.156582 | 5273.156586 |
| 5273.156649 | Kernel\_Read\|xilinx:adm-pcie-7v3:1ddr:3.0-0\|cnn\_GOOD | Read | OCL Region | 1 | 1 | 118147 | 118148 | 0.00333333 | 5273.156649 | 5273.156652 |
| 5273.156712 | Kernel\_Read\|xilinx:adm-pcie-7v3:1ddr:3.0-0\|cnn\_GOOD | Read | OCL Region | 1 | 1 | 118166 | 118167 | 0.00333333 | 5273.156712 | 5273.156716 |
| 5273.156782 | Kernel\_Read\|xilinx:adm-pcie-7v3:1ddr:3.0-0\|cnn\_GOOD | Read | OCL Region | 1 | 1 | 118187 | 118188 | 0.00333333 | 5273.156782 | 5273.156786 |
| 5273.156849 | Kernel\_Read\|xilinx:adm-pcie-7v3:1ddr:3.0-0\|cnn\_GOOD | Read | OCL Region | 1 | 1 | 118207 | 118208 | 0.00333333 | 5273.156849 | 5273.156852 |
| 5273.156916 | Kernel\_Read\|xilinx:adm-pcie-7v3:1ddr:3.0-0\|cnn\_GOOD | Read | OCL Region | 1 | 1 | 118227 | 118228 | 0.00333333 | 5273.156916 | 5273.156919 |
| 5273.156982 | Kernel\_Read\|xilinx:adm-pcie-7v3:1ddr:3.0-0\|cnn\_GOOD | Read | OCL Region | 1 | 1 | 118247 | 118248 | 0.00333333 | 5273.156982 | 5273.156986 |
| 5273.157049 | Kernel\_Read\|xilinx:adm-pcie-7v3:1ddr:3.0-0\|cnn\_GOOD | Read | OCL Region | 1 | 1 | 118267 | 118268 | 0.00333333 | 5273.157049 | 5273.157052 |
| 5273.157112 | Kernel\_Read\|xilinx:adm-pcie-7v3:1ddr:3.0-0\|cnn\_GOOD | Read | OCL Region | 1 | 1 | 118286 | 118287 | 0.00333333 | 5273.157112 | 5273.157116 |
| 5273.157182 | Kernel\_Read\|xilinx:adm-pcie-7v3:1ddr:3.0-0\|cnn\_GOOD | Read | OCL Region | 1 | 1 | 118307 | 118308 | 0.00333333 | 5273.157182 | 5273.157186 |
| 5273.157249 | Kernel\_Read\|xilinx:adm-pcie-7v3:1ddr:3.0-0\|cnn\_GOOD | Read | OCL Region | 1 | 1 | 118327 | 118328 | 0.00333333 | 5273.157249 | 5273.157252 |
| 5273.157316 | Kernel\_Read\|xilinx:adm-pcie-7v3:1ddr:3.0-0\|cnn\_GOOD | Read | OCL Region | 1 | 1 | 118347 | 118348 | 0.00333333 | 5273.157316 | 5273.157319 |
| 5273.157382 | Kernel\_Read\|xilinx:adm-pcie-7v3:1ddr:3.0-0\|cnn\_GOOD | Read | OCL Region | 1 | 1 | 118367 | 118368 | 0.00333333 | 5273.157382 | 5273.157386 |
| 5273.157459 | Kernel\_Read\|xilinx:adm-pcie-7v3:1ddr:3.0-0\|cnn\_GOOD | Read | OCL Region | 1 | 1 | 118390 | 118391 | 0.00333333 | 5273.157459 | 5273.157462 |
| 5273.157512 | Kernel\_Read\|xilinx:adm-pcie-7v3:1ddr:3.0-0\|cnn\_GOOD | Read | OCL Region | 1 | 1 | 118406 | 118407 | 0.00333333 | 5273.157512 | 5273.157516 |
| 5273.157582 | Kernel\_Read\|xilinx:adm-pcie-7v3:1ddr:3.0-0\|cnn\_GOOD | Read | OCL Region | 1 | 1 | 118427 | 118428 | 0.00333333 | 5273.157582 | 5273.157586 |
| 5273.157649 | Kernel\_Read\|xilinx:adm-pcie-7v3:1ddr:3.0-0\|cnn\_GOOD | Read | OCL Region | 1 | 1 | 118447 | 118448 | 0.00333333 | 5273.157649 | 5273.157652 |
| 5273.157712 | Kernel\_Read\|xilinx:adm-pcie-7v3:1ddr:3.0-0\|cnn\_GOOD | Read | OCL Region | 1 | 1 | 118466 | 118467 | 0.00333333 | 5273.157712 | 5273.157716 |
| 5273.157782 | Kernel\_Read\|xilinx:adm-pcie-7v3:1ddr:3.0-0\|cnn\_GOOD | Read | OCL Region | 1 | 1 | 118487 | 118488 | 0.00333333 | 5273.157782 | 5273.157786 |
| 5273.157849 | Kernel\_Read\|xilinx:adm-pcie-7v3:1ddr:3.0-0\|cnn\_GOOD | Read | OCL Region | 1 | 1 | 118507 | 118508 | 0.00333333 | 5273.157849 | 5273.157852 |
| 5273.157912 | Kernel\_Read\|xilinx:adm-pcie-7v3:1ddr:3.0-0\|cnn\_GOOD | Read | OCL Region | 1 | 1 | 118526 | 118527 | 0.00333333 | 5273.157912 | 5273.157916 |
| 5273.157982 | Kernel\_Read\|xilinx:adm-pcie-7v3:1ddr:3.0-0\|cnn\_GOOD | Read | OCL Region | 1 | 1 | 118547 | 118548 | 0.00333333 | 5273.157982 | 5273.157986 |
| 5273.158049 | Kernel\_Read\|xilinx:adm-pcie-7v3:1ddr:3.0-0\|cnn\_GOOD | Read | OCL Region | 1 | 1 | 118567 | 118568 | 0.00333333 | 5273.158049 | 5273.158052 |
| 5273.158116 | Kernel\_Read\|xilinx:adm-pcie-7v3:1ddr:3.0-0\|cnn\_GOOD | Read | OCL Region | 1 | 1 | 118587 | 118588 | 0.00333333 | 5273.158116 | 5273.158119 |
| 5273.158182 | Kernel\_Read\|xilinx:adm-pcie-7v3:1ddr:3.0-0\|cnn\_GOOD | Read | OCL Region | 1 | 1 | 118607 | 118608 | 0.00333333 | 5273.158182 | 5273.158186 |
| 5273.158249 | Kernel\_Read\|xilinx:adm-pcie-7v3:1ddr:3.0-0\|cnn\_GOOD | Read | OCL Region | 1 | 1 | 118627 | 118628 | 0.00333333 | 5273.158249 | 5273.158252 |
| 5273.158312 | Kernel\_Read\|xilinx:adm-pcie-7v3:1ddr:3.0-0\|cnn\_GOOD | Read | OCL Region | 1 | 1 | 118646 | 118647 | 0.00333333 | 5273.158312 | 5273.158316 |
| 5273.158382 | Kernel\_Read\|xilinx:adm-pcie-7v3:1ddr:3.0-0\|cnn\_GOOD | Read | OCL Region | 1 | 1 | 118667 | 118668 | 0.00333333 | 5273.158382 | 5273.158386 |
| 5273.158449 | Kernel\_Read\|xilinx:adm-pcie-7v3:1ddr:3.0-0\|cnn\_GOOD | Read | OCL Region | 1 | 1 | 118687 | 118688 | 0.00333333 | 5273.158449 | 5273.158452 |
| 5273.158516 | Kernel\_Read\|xilinx:adm-pcie-7v3:1ddr:3.0-0\|cnn\_GOOD | Read | OCL Region | 1 | 1 | 118707 | 118708 | 0.00333333 | 5273.158516 | 5273.158519 |
| 5273.158582 | Kernel\_Read\|xilinx:adm-pcie-7v3:1ddr:3.0-0\|cnn\_GOOD | Read | OCL Region | 1 | 1 | 118727 | 118728 | 0.00333333 | 5273.158582 | 5273.158586 |
| 5273.158649 | Kernel\_Read\|xilinx:adm-pcie-7v3:1ddr:3.0-0\|cnn\_GOOD | Read | OCL Region | 1 | 1 | 118747 | 118748 | 0.00333333 | 5273.158649 | 5273.158652 |
| 5273.158712 | Kernel\_Read\|xilinx:adm-pcie-7v3:1ddr:3.0-0\|cnn\_GOOD | Read | OCL Region | 1 | 1 | 118766 | 118767 | 0.00333333 | 5273.158712 | 5273.158716 |
| 5273.158782 | Kernel\_Read\|xilinx:adm-pcie-7v3:1ddr:3.0-0\|cnn\_GOOD | Read | OCL Region | 1 | 1 | 118787 | 118788 | 0.00333333 | 5273.158782 | 5273.158786 |
| 5273.158849 | Kernel\_Read\|xilinx:adm-pcie-7v3:1ddr:3.0-0\|cnn\_GOOD | Read | OCL Region | 1 | 1 | 118807 | 118808 | 0.00333333 | 5273.158849 | 5273.158852 |
| 5273.158916 | Kernel\_Read\|xilinx:adm-pcie-7v3:1ddr:3.0-0\|cnn\_GOOD | Read | OCL Region | 1 | 1 | 118827 | 118828 | 0.00333333 | 5273.158916 | 5273.158919 |
| 5273.158982 | Kernel\_Read\|xilinx:adm-pcie-7v3:1ddr:3.0-0\|cnn\_GOOD | Read | OCL Region | 1 | 1 | 118847 | 118848 | 0.00333333 | 5273.158982 | 5273.158986 |
| 5273.159049 | Kernel\_Read\|xilinx:adm-pcie-7v3:1ddr:3.0-0\|cnn\_GOOD | Read | OCL Region | 1 | 1 | 118867 | 118868 | 0.00333333 | 5273.159049 | 5273.159052 |
| 5273.159112 | Kernel\_Read\|xilinx:adm-pcie-7v3:1ddr:3.0-0\|cnn\_GOOD | Read | OCL Region | 1 | 1 | 118886 | 118887 | 0.00333333 | 5273.159112 | 5273.159116 |
| 5273.159182 | Kernel\_Read\|xilinx:adm-pcie-7v3:1ddr:3.0-0\|cnn\_GOOD | Read | OCL Region | 1 | 1 | 118907 | 118908 | 0.00333333 | 5273.159182 | 5273.159186 |
| 5273.159249 | Kernel\_Read\|xilinx:adm-pcie-7v3:1ddr:3.0-0\|cnn\_GOOD | Read | OCL Region | 1 | 1 | 118927 | 118928 | 0.00333333 | 5273.159249 | 5273.159252 |
| 5273.159312 | Kernel\_Read\|xilinx:adm-pcie-7v3:1ddr:3.0-0\|cnn\_GOOD | Read | OCL Region | 1 | 1 | 118946 | 118947 | 0.00333333 | 5273.159312 | 5273.159316 |
| 5273.159382 | Kernel\_Read\|xilinx:adm-pcie-7v3:1ddr:3.0-0\|cnn\_GOOD | Read | OCL Region | 1 | 1 | 118967 | 118968 | 0.00333333 | 5273.159382 | 5273.159386 |
| 5273.159449 | Kernel\_Read\|xilinx:adm-pcie-7v3:1ddr:3.0-0\|cnn\_GOOD | Read | OCL Region | 1 | 1 | 118987 | 118988 | 0.00333333 | 5273.159449 | 5273.159452 |
| 5273.159512 | Kernel\_Read\|xilinx:adm-pcie-7v3:1ddr:3.0-0\|cnn\_GOOD | Read | OCL Region | 1 | 1 | 119006 | 119007 | 0.00333333 | 5273.159512 | 5273.159516 |
| 5273.159582 | Kernel\_Read\|xilinx:adm-pcie-7v3:1ddr:3.0-0\|cnn\_GOOD | Read | OCL Region | 1 | 1 | 119027 | 119028 | 0.00333333 | 5273.159582 | 5273.159586 |
| 5273.159649 | Kernel\_Read\|xilinx:adm-pcie-7v3:1ddr:3.0-0\|cnn\_GOOD | Read | OCL Region | 1 | 1 | 119047 | 119048 | 0.00333333 | 5273.159649 | 5273.159652 |
| 5273.159716 | Kernel\_Read\|xilinx:adm-pcie-7v3:1ddr:3.0-0\|cnn\_GOOD | Read | OCL Region | 1 | 1 | 119067 | 119068 | 0.00333333 | 5273.159716 | 5273.159719 |
| 5273.159782 | Kernel\_Read\|xilinx:adm-pcie-7v3:1ddr:3.0-0\|cnn\_GOOD | Read | OCL Region | 1 | 1 | 119087 | 119088 | 0.00333333 | 5273.159782 | 5273.159786 |
| 5273.159849 | Kernel\_Read\|xilinx:adm-pcie-7v3:1ddr:3.0-0\|cnn\_GOOD | Read | OCL Region | 1 | 1 | 119107 | 119108 | 0.00333333 | 5273.159849 | 5273.159852 |
| 5273.159912 | Kernel\_Read\|xilinx:adm-pcie-7v3:1ddr:3.0-0\|cnn\_GOOD | Read | OCL Region | 1 | 1 | 119126 | 119127 | 0.00333333 | 5273.159912 | 5273.159916 |
| 5273.159982 | Kernel\_Read\|xilinx:adm-pcie-7v3:1ddr:3.0-0\|cnn\_GOOD | Read | OCL Region | 1 | 1 | 119147 | 119148 | 0.00333333 | 5273.159982 | 5273.159986 |
| 5273.160049 | Kernel\_Read\|xilinx:adm-pcie-7v3:1ddr:3.0-0\|cnn\_GOOD | Read | OCL Region | 1 | 1 | 119167 | 119168 | 0.00333333 | 5273.160049 | 5273.160052 |
| 5273.160112 | Kernel\_Read\|xilinx:adm-pcie-7v3:1ddr:3.0-0\|cnn\_GOOD | Read | OCL Region | 1 | 1 | 119186 | 119187 | 0.00333333 | 5273.160112 | 5273.160116 |
| 5273.160182 | Kernel\_Read\|xilinx:adm-pcie-7v3:1ddr:3.0-0\|cnn\_GOOD | Read | OCL Region | 1 | 1 | 119207 | 119208 | 0.00333333 | 5273.160182 | 5273.160186 |
| 5273.160249 | Kernel\_Read\|xilinx:adm-pcie-7v3:1ddr:3.0-0\|cnn\_GOOD | Read | OCL Region | 1 | 1 | 119227 | 119228 | 0.00333333 | 5273.160249 | 5273.160252 |
| 5273.160312 | Kernel\_Read\|xilinx:adm-pcie-7v3:1ddr:3.0-0\|cnn\_GOOD | Read | OCL Region | 1 | 1 | 119246 | 119247 | 0.00333333 | 5273.160312 | 5273.160316 |
| 5273.160382 | Kernel\_Read\|xilinx:adm-pcie-7v3:1ddr:3.0-0\|cnn\_GOOD | Read | OCL Region | 1 | 1 | 119267 | 119268 | 0.00333333 | 5273.160382 | 5273.160386 |
| 5273.160449 | Kernel\_Read\|xilinx:adm-pcie-7v3:1ddr:3.0-0\|cnn\_GOOD | Read | OCL Region | 1 | 1 | 119287 | 119288 | 0.00333333 | 5273.160449 | 5273.160452 |
| 5273.160516 | Kernel\_Read\|xilinx:adm-pcie-7v3:1ddr:3.0-0\|cnn\_GOOD | Read | OCL Region | 1 | 1 | 119307 | 119308 | 0.00333333 | 5273.160516 | 5273.160519 |
| 5273.160582 | Kernel\_Read\|xilinx:adm-pcie-7v3:1ddr:3.0-0\|cnn\_GOOD | Read | OCL Region | 1 | 1 | 119327 | 119328 | 0.00333333 | 5273.160582 | 5273.160586 |
| 5273.160649 | Kernel\_Read\|xilinx:adm-pcie-7v3:1ddr:3.0-0\|cnn\_GOOD | Read | OCL Region | 1 | 1 | 119347 | 119348 | 0.00333333 | 5273.160649 | 5273.160652 |
| 5273.160726 | Kernel\_Read\|xilinx:adm-pcie-7v3:1ddr:3.0-0\|cnn\_GOOD | Read | OCL Region | 1 | 1 | 119370 | 119371 | 0.00333333 | 5273.160726 | 5273.160729 |
| 5273.160782 | Kernel\_Read\|xilinx:adm-pcie-7v3:1ddr:3.0-0\|cnn\_GOOD | Read | OCL Region | 1 | 1 | 119387 | 119388 | 0.00333333 | 5273.160782 | 5273.160786 |
| 5273.160849 | Kernel\_Read\|xilinx:adm-pcie-7v3:1ddr:3.0-0\|cnn\_GOOD | Read | OCL Region | 1 | 1 | 119407 | 119408 | 0.00333333 | 5273.160849 | 5273.160852 |
| 5273.160912 | Kernel\_Read\|xilinx:adm-pcie-7v3:1ddr:3.0-0\|cnn\_GOOD | Read | OCL Region | 1 | 1 | 119426 | 119427 | 0.00333333 | 5273.160912 | 5273.160916 |
| 5273.160982 | Kernel\_Read\|xilinx:adm-pcie-7v3:1ddr:3.0-0\|cnn\_GOOD | Read | OCL Region | 1 | 1 | 119447 | 119448 | 0.00333333 | 5273.160982 | 5273.160986 |
| 5273.161049 | Kernel\_Read\|xilinx:adm-pcie-7v3:1ddr:3.0-0\|cnn\_GOOD | Read | OCL Region | 1 | 1 | 119467 | 119468 | 0.00333333 | 5273.161049 | 5273.161052 |
| 5273.161112 | Kernel\_Read\|xilinx:adm-pcie-7v3:1ddr:3.0-0\|cnn\_GOOD | Read | OCL Region | 1 | 1 | 119486 | 119487 | 0.00333333 | 5273.161112 | 5273.161116 |
| 5273.161182 | Kernel\_Read\|xilinx:adm-pcie-7v3:1ddr:3.0-0\|cnn\_GOOD | Read | OCL Region | 1 | 1 | 119507 | 119508 | 0.00333333 | 5273.161182 | 5273.161186 |
| 5273.161249 | Kernel\_Read\|xilinx:adm-pcie-7v3:1ddr:3.0-0\|cnn\_GOOD | Read | OCL Region | 1 | 1 | 119527 | 119528 | 0.00333333 | 5273.161249 | 5273.161252 |
| 5273.161316 | Kernel\_Read\|xilinx:adm-pcie-7v3:1ddr:3.0-0\|cnn\_GOOD | Read | OCL Region | 1 | 1 | 119547 | 119548 | 0.00333333 | 5273.161316 | 5273.161319 |
| 5273.161382 | Kernel\_Read\|xilinx:adm-pcie-7v3:1ddr:3.0-0\|cnn\_GOOD | Read | OCL Region | 1 | 1 | 119567 | 119568 | 0.00333333 | 5273.161382 | 5273.161386 |
| 5273.161449 | Kernel\_Read\|xilinx:adm-pcie-7v3:1ddr:3.0-0\|cnn\_GOOD | Read | OCL Region | 1 | 1 | 119587 | 119588 | 0.00333333 | 5273.161449 | 5273.161452 |
| 5273.161512 | Kernel\_Read\|xilinx:adm-pcie-7v3:1ddr:3.0-0\|cnn\_GOOD | Read | OCL Region | 1 | 1 | 119606 | 119607 | 0.00333333 | 5273.161512 | 5273.161516 |
| 5273.161582 | Kernel\_Read\|xilinx:adm-pcie-7v3:1ddr:3.0-0\|cnn\_GOOD | Read | OCL Region | 1 | 1 | 119627 | 119628 | 0.00333333 | 5273.161582 | 5273.161586 |
| 5273.161649 | Kernel\_Read\|xilinx:adm-pcie-7v3:1ddr:3.0-0\|cnn\_GOOD | Read | OCL Region | 1 | 1 | 119647 | 119648 | 0.00333333 | 5273.161649 | 5273.161652 |
| 5273.161716 | Kernel\_Read\|xilinx:adm-pcie-7v3:1ddr:3.0-0\|cnn\_GOOD | Read | OCL Region | 1 | 1 | 119667 | 119668 | 0.00333333 | 5273.161716 | 5273.161719 |
| 5273.161782 | Kernel\_Read\|xilinx:adm-pcie-7v3:1ddr:3.0-0\|cnn\_GOOD | Read | OCL Region | 1 | 1 | 119687 | 119688 | 0.00333333 | 5273.161782 | 5273.161786 |
| 5273.161849 | Kernel\_Read\|xilinx:adm-pcie-7v3:1ddr:3.0-0\|cnn\_GOOD | Read | OCL Region | 1 | 1 | 119707 | 119708 | 0.00333333 | 5273.161849 | 5273.161852 |
| 5273.162126 | Kernel\_Read\|xilinx:adm-pcie-7v3:1ddr:3.0-0\|cnn\_GOOD | Read | OCL Region | 1 | 1 | 119790 | 119791 | 0.00333333 | 5273.162126 | 5273.162129 |
| 5273.162129 | Kernel\_Read\|xilinx:adm-pcie-7v3:1ddr:3.0-0\|cnn\_GOOD | Read | OCL Region | 1 | 1 | 119791 | 119792 | 0.00333333 | 5273.162129 | 5273.162132 |
| 5273.162132 | Kernel\_Read\|xilinx:adm-pcie-7v3:1ddr:3.0-0\|cnn\_GOOD | Read | OCL Region | 1 | 1 | 119792 | 119793 | 0.00333333 | 5273.162132 | 5273.162136 |
| 5273.162142 | Kernel\_Read\|xilinx:adm-pcie-7v3:1ddr:3.0-0\|cnn\_GOOD | Read | OCL Region | 1 | 1 | 119795 | 119796 | 0.00333333 | 5273.162142 | 5273.162146 |
| 5273.162199 | Kernel\_Read\|xilinx:adm-pcie-7v3:1ddr:3.0-0\|cnn\_GOOD | Read | OCL Region | 1 | 1 | 119812 | 119813 | 0.00333333 | 5273.162199 | 5273.162202 |
| 5273.162256 | Kernel\_Read\|xilinx:adm-pcie-7v3:1ddr:3.0-0\|cnn\_GOOD | Read | OCL Region | 1 | 1 | 119829 | 119830 | 0.00333333 | 5273.162256 | 5273.162259 |
| 5273.162312 | Kernel\_Read\|xilinx:adm-pcie-7v3:1ddr:3.0-0\|cnn\_GOOD | Read | OCL Region | 1 | 1 | 119846 | 119847 | 0.00333333 | 5273.162312 | 5273.162316 |
| 5273.162382 | Kernel\_Read\|xilinx:adm-pcie-7v3:1ddr:3.0-0\|cnn\_GOOD | Read | OCL Region | 1 | 1 | 119867 | 119868 | 0.00333333 | 5273.162382 | 5273.162386 |
| 5273.162449 | Kernel\_Read\|xilinx:adm-pcie-7v3:1ddr:3.0-0\|cnn\_GOOD | Read | OCL Region | 1 | 1 | 119887 | 119888 | 0.00333333 | 5273.162449 | 5273.162452 |
| 5273.162512 | Kernel\_Read\|xilinx:adm-pcie-7v3:1ddr:3.0-0\|cnn\_GOOD | Read | OCL Region | 1 | 1 | 119906 | 119907 | 0.00333333 | 5273.162512 | 5273.162516 |
| 5273.162582 | Kernel\_Read\|xilinx:adm-pcie-7v3:1ddr:3.0-0\|cnn\_GOOD | Read | OCL Region | 1 | 1 | 119927 | 119928 | 0.00333333 | 5273.162582 | 5273.162586 |
| 5273.162649 | Kernel\_Read\|xilinx:adm-pcie-7v3:1ddr:3.0-0\|cnn\_GOOD | Read | OCL Region | 1 | 1 | 119947 | 119948 | 0.00333333 | 5273.162649 | 5273.162652 |
| 5273.162712 | Kernel\_Read\|xilinx:adm-pcie-7v3:1ddr:3.0-0\|cnn\_GOOD | Read | OCL Region | 1 | 1 | 119966 | 119967 | 0.00333333 | 5273.162712 | 5273.162716 |
| 5273.162782 | Kernel\_Read\|xilinx:adm-pcie-7v3:1ddr:3.0-0\|cnn\_GOOD | Read | OCL Region | 1 | 1 | 119987 | 119988 | 0.00333333 | 5273.162782 | 5273.162786 |
| 5273.162849 | Kernel\_Read\|xilinx:adm-pcie-7v3:1ddr:3.0-0\|cnn\_GOOD | Read | OCL Region | 1 | 1 | 120007 | 120008 | 0.00333333 | 5273.162849 | 5273.162852 |
| 5273.162916 | Kernel\_Read\|xilinx:adm-pcie-7v3:1ddr:3.0-0\|cnn\_GOOD | Read | OCL Region | 1 | 1 | 120027 | 120028 | 0.00333333 | 5273.162916 | 5273.162919 |
| 5273.162982 | Kernel\_Read\|xilinx:adm-pcie-7v3:1ddr:3.0-0\|cnn\_GOOD | Read | OCL Region | 1 | 1 | 120047 | 120048 | 0.00333333 | 5273.162982 | 5273.162986 |
| 5273.163049 | Kernel\_Read\|xilinx:adm-pcie-7v3:1ddr:3.0-0\|cnn\_GOOD | Read | OCL Region | 1 | 1 | 120067 | 120068 | 0.00333333 | 5273.163049 | 5273.163052 |
| 5273.163112 | Kernel\_Read\|xilinx:adm-pcie-7v3:1ddr:3.0-0\|cnn\_GOOD | Read | OCL Region | 1 | 1 | 120086 | 120087 | 0.00333333 | 5273.163112 | 5273.163116 |
| 5273.163182 | Kernel\_Read\|xilinx:adm-pcie-7v3:1ddr:3.0-0\|cnn\_GOOD | Read | OCL Region | 1 | 1 | 120107 | 120108 | 0.00333333 | 5273.163182 | 5273.163186 |
| 5273.163249 | Kernel\_Read\|xilinx:adm-pcie-7v3:1ddr:3.0-0\|cnn\_GOOD | Read | OCL Region | 1 | 1 | 120127 | 120128 | 0.00333333 | 5273.163249 | 5273.163252 |
| 5273.163312 | Kernel\_Read\|xilinx:adm-pcie-7v3:1ddr:3.0-0\|cnn\_GOOD | Read | OCL Region | 1 | 1 | 120146 | 120147 | 0.00333333 | 5273.163312 | 5273.163316 |
| 5273.163382 | Kernel\_Read\|xilinx:adm-pcie-7v3:1ddr:3.0-0\|cnn\_GOOD | Read | OCL Region | 1 | 1 | 120167 | 120168 | 0.00333333 | 5273.163382 | 5273.163386 |
| 5273.163449 | Kernel\_Read\|xilinx:adm-pcie-7v3:1ddr:3.0-0\|cnn\_GOOD | Read | OCL Region | 1 | 1 | 120187 | 120188 | 0.00333333 | 5273.163449 | 5273.163452 |
| 5273.163512 | Kernel\_Read\|xilinx:adm-pcie-7v3:1ddr:3.0-0\|cnn\_GOOD | Read | OCL Region | 1 | 1 | 120206 | 120207 | 0.00333333 | 5273.163512 | 5273.163516 |
| 5273.163582 | Kernel\_Read\|xilinx:adm-pcie-7v3:1ddr:3.0-0\|cnn\_GOOD | Read | OCL Region | 1 | 1 | 120227 | 120228 | 0.00333333 | 5273.163582 | 5273.163586 |
| 5273.163649 | Kernel\_Read\|xilinx:adm-pcie-7v3:1ddr:3.0-0\|cnn\_GOOD | Read | OCL Region | 1 | 1 | 120247 | 120248 | 0.00333333 | 5273.163649 | 5273.163652 |
| 5273.163729 | Kernel\_Read\|xilinx:adm-pcie-7v3:1ddr:3.0-0\|cnn\_GOOD | Read | OCL Region | 1 | 1 | 120271 | 120272 | 0.00333333 | 5273.163729 | 5273.163732 |
| 5273.163782 | Kernel\_Read\|xilinx:adm-pcie-7v3:1ddr:3.0-0\|cnn\_GOOD | Read | OCL Region | 1 | 1 | 120287 | 120288 | 0.00333333 | 5273.163782 | 5273.163786 |
| 5273.163849 | Kernel\_Read\|xilinx:adm-pcie-7v3:1ddr:3.0-0\|cnn\_GOOD | Read | OCL Region | 1 | 1 | 120307 | 120308 | 0.00333333 | 5273.163849 | 5273.163852 |
| 5273.163912 | Kernel\_Read\|xilinx:adm-pcie-7v3:1ddr:3.0-0\|cnn\_GOOD | Read | OCL Region | 1 | 1 | 120326 | 120327 | 0.00333333 | 5273.163912 | 5273.163916 |
| 5273.163982 | Kernel\_Read\|xilinx:adm-pcie-7v3:1ddr:3.0-0\|cnn\_GOOD | Read | OCL Region | 1 | 1 | 120347 | 120348 | 0.00333333 | 5273.163982 | 5273.163986 |
| 5273.164049 | Kernel\_Read\|xilinx:adm-pcie-7v3:1ddr:3.0-0\|cnn\_GOOD | Read | OCL Region | 1 | 1 | 120367 | 120368 | 0.00333333 | 5273.164049 | 5273.164052 |
| 5273.164112 | Kernel\_Read\|xilinx:adm-pcie-7v3:1ddr:3.0-0\|cnn\_GOOD | Read | OCL Region | 1 | 1 | 120386 | 120387 | 0.00333333 | 5273.164112 | 5273.164116 |
| 5273.164182 | Kernel\_Read\|xilinx:adm-pcie-7v3:1ddr:3.0-0\|cnn\_GOOD | Read | OCL Region | 1 | 1 | 120407 | 120408 | 0.00333333 | 5273.164182 | 5273.164186 |
| 5273.164249 | Kernel\_Read\|xilinx:adm-pcie-7v3:1ddr:3.0-0\|cnn\_GOOD | Read | OCL Region | 1 | 1 | 120427 | 120428 | 0.00333333 | 5273.164249 | 5273.164252 |
| 5273.164312 | Kernel\_Read\|xilinx:adm-pcie-7v3:1ddr:3.0-0\|cnn\_GOOD | Read | OCL Region | 1 | 1 | 120446 | 120447 | 0.00333333 | 5273.164312 | 5273.164316 |
| 5273.164382 | Kernel\_Read\|xilinx:adm-pcie-7v3:1ddr:3.0-0\|cnn\_GOOD | Read | OCL Region | 1 | 1 | 120467 | 120468 | 0.00333333 | 5273.164382 | 5273.164386 |
| 5273.164449 | Kernel\_Read\|xilinx:adm-pcie-7v3:1ddr:3.0-0\|cnn\_GOOD | Read | OCL Region | 1 | 1 | 120487 | 120488 | 0.00333333 | 5273.164449 | 5273.164452 |
| 5273.164522 | Kernel\_Read\|xilinx:adm-pcie-7v3:1ddr:3.0-0\|cnn\_GOOD | Read | OCL Region | 1 | 1 | 120509 | 120510 | 0.00333333 | 5273.164522 | 5273.164526 |
| 5273.164582 | Kernel\_Read\|xilinx:adm-pcie-7v3:1ddr:3.0-0\|cnn\_GOOD | Read | OCL Region | 1 | 1 | 120527 | 120528 | 0.00333333 | 5273.164582 | 5273.164586 |
| 5273.164649 | Kernel\_Read\|xilinx:adm-pcie-7v3:1ddr:3.0-0\|cnn\_GOOD | Read | OCL Region | 1 | 1 | 120547 | 120548 | 0.00333333 | 5273.164649 | 5273.164652 |
| 5273.164712 | Kernel\_Read\|xilinx:adm-pcie-7v3:1ddr:3.0-0\|cnn\_GOOD | Read | OCL Region | 1 | 1 | 120566 | 120567 | 0.00333333 | 5273.164712 | 5273.164716 |
| 5273.164782 | Kernel\_Read\|xilinx:adm-pcie-7v3:1ddr:3.0-0\|cnn\_GOOD | Read | OCL Region | 1 | 1 | 120587 | 120588 | 0.00333333 | 5273.164782 | 5273.164786 |
| 5273.164849 | Kernel\_Read\|xilinx:adm-pcie-7v3:1ddr:3.0-0\|cnn\_GOOD | Read | OCL Region | 1 | 1 | 120607 | 120608 | 0.00333333 | 5273.164849 | 5273.164852 |
| 5273.164912 | Kernel\_Read\|xilinx:adm-pcie-7v3:1ddr:3.0-0\|cnn\_GOOD | Read | OCL Region | 1 | 1 | 120626 | 120627 | 0.00333333 | 5273.164912 | 5273.164916 |
| 5273.164982 | Kernel\_Read\|xilinx:adm-pcie-7v3:1ddr:3.0-0\|cnn\_GOOD | Read | OCL Region | 1 | 1 | 120647 | 120648 | 0.00333333 | 5273.164982 | 5273.164986 |
| 5273.165049 | Kernel\_Read\|xilinx:adm-pcie-7v3:1ddr:3.0-0\|cnn\_GOOD | Read | OCL Region | 1 | 1 | 120667 | 120668 | 0.00333333 | 5273.165049 | 5273.165052 |
| 5273.165112 | Kernel\_Read\|xilinx:adm-pcie-7v3:1ddr:3.0-0\|cnn\_GOOD | Read | OCL Region | 1 | 1 | 120686 | 120687 | 0.00333333 | 5273.165112 | 5273.165116 |
| 5273.165182 | Kernel\_Read\|xilinx:adm-pcie-7v3:1ddr:3.0-0\|cnn\_GOOD | Read | OCL Region | 1 | 1 | 120707 | 120708 | 0.00333333 | 5273.165182 | 5273.165186 |
| 5273.165249 | Kernel\_Read\|xilinx:adm-pcie-7v3:1ddr:3.0-0\|cnn\_GOOD | Read | OCL Region | 1 | 1 | 120727 | 120728 | 0.00333333 | 5273.165249 | 5273.165252 |
| 5273.165312 | Kernel\_Read\|xilinx:adm-pcie-7v3:1ddr:3.0-0\|cnn\_GOOD | Read | OCL Region | 1 | 1 | 120746 | 120747 | 0.00333333 | 5273.165312 | 5273.165316 |
| 5273.165382 | Kernel\_Read\|xilinx:adm-pcie-7v3:1ddr:3.0-0\|cnn\_GOOD | Read | OCL Region | 1 | 1 | 120767 | 120768 | 0.00333333 | 5273.165382 | 5273.165386 |
| 5273.165449 | Kernel\_Read\|xilinx:adm-pcie-7v3:1ddr:3.0-0\|cnn\_GOOD | Read | OCL Region | 1 | 1 | 120787 | 120788 | 0.00333333 | 5273.165449 | 5273.165452 |
| 5273.165512 | Kernel\_Read\|xilinx:adm-pcie-7v3:1ddr:3.0-0\|cnn\_GOOD | Read | OCL Region | 1 | 1 | 120806 | 120807 | 0.00333333 | 5273.165512 | 5273.165516 |
| 5273.165582 | Kernel\_Read\|xilinx:adm-pcie-7v3:1ddr:3.0-0\|cnn\_GOOD | Read | OCL Region | 1 | 1 | 120827 | 120828 | 0.00333333 | 5273.165582 | 5273.165586 |
| 5273.165649 | Kernel\_Read\|xilinx:adm-pcie-7v3:1ddr:3.0-0\|cnn\_GOOD | Read | OCL Region | 1 | 1 | 120847 | 120848 | 0.00333333 | 5273.165649 | 5273.165652 |
| 5273.165712 | Kernel\_Read\|xilinx:adm-pcie-7v3:1ddr:3.0-0\|cnn\_GOOD | Read | OCL Region | 1 | 1 | 120866 | 120867 | 0.00333333 | 5273.165712 | 5273.165716 |
| 5273.165782 | Kernel\_Read\|xilinx:adm-pcie-7v3:1ddr:3.0-0\|cnn\_GOOD | Read | OCL Region | 1 | 1 | 120887 | 120888 | 0.00333333 | 5273.165782 | 5273.165786 |
| 5273.165849 | Kernel\_Read\|xilinx:adm-pcie-7v3:1ddr:3.0-0\|cnn\_GOOD | Read | OCL Region | 1 | 1 | 120907 | 120908 | 0.00333333 | 5273.165849 | 5273.165852 |
| 5273.165912 | Kernel\_Read\|xilinx:adm-pcie-7v3:1ddr:3.0-0\|cnn\_GOOD | Read | OCL Region | 1 | 1 | 120926 | 120927 | 0.00333333 | 5273.165912 | 5273.165916 |
| 5273.165982 | Kernel\_Read\|xilinx:adm-pcie-7v3:1ddr:3.0-0\|cnn\_GOOD | Read | OCL Region | 1 | 1 | 120947 | 120948 | 0.00333333 | 5273.165982 | 5273.165986 |
| 5273.166049 | Kernel\_Read\|xilinx:adm-pcie-7v3:1ddr:3.0-0\|cnn\_GOOD | Read | OCL Region | 1 | 1 | 120967 | 120968 | 0.00333333 | 5273.166049 | 5273.166052 |
| 5273.166112 | Kernel\_Read\|xilinx:adm-pcie-7v3:1ddr:3.0-0\|cnn\_GOOD | Read | OCL Region | 1 | 1 | 120986 | 120987 | 0.00333333 | 5273.166112 | 5273.166116 |
| 5273.166179 | Kernel\_Read\|xilinx:adm-pcie-7v3:1ddr:3.0-0\|cnn\_GOOD | Read | OCL Region | 1 | 1 | 121006 | 121007 | 0.00333333 | 5273.166179 | 5273.166182 |
| 5273.166249 | Kernel\_Read\|xilinx:adm-pcie-7v3:1ddr:3.0-0\|cnn\_GOOD | Read | OCL Region | 1 | 1 | 121027 | 121028 | 0.00333333 | 5273.166249 | 5273.166252 |
| 5273.166312 | Kernel\_Read\|xilinx:adm-pcie-7v3:1ddr:3.0-0\|cnn\_GOOD | Read | OCL Region | 1 | 1 | 121046 | 121047 | 0.00333333 | 5273.166312 | 5273.166316 |
| 5273.166382 | Kernel\_Read\|xilinx:adm-pcie-7v3:1ddr:3.0-0\|cnn\_GOOD | Read | OCL Region | 1 | 1 | 121067 | 121068 | 0.00333333 | 5273.166382 | 5273.166386 |
| 5273.166449 | Kernel\_Read\|xilinx:adm-pcie-7v3:1ddr:3.0-0\|cnn\_GOOD | Read | OCL Region | 1 | 1 | 121087 | 121088 | 0.00333333 | 5273.166449 | 5273.166452 |
| 5273.166512 | Kernel\_Read\|xilinx:adm-pcie-7v3:1ddr:3.0-0\|cnn\_GOOD | Read | OCL Region | 1 | 1 | 121106 | 121107 | 0.00333333 | 5273.166512 | 5273.166516 |
| 5273.166582 | Kernel\_Read\|xilinx:adm-pcie-7v3:1ddr:3.0-0\|cnn\_GOOD | Read | OCL Region | 1 | 1 | 121127 | 121128 | 0.00333333 | 5273.166582 | 5273.166586 |
| 5273.166649 | Kernel\_Read\|xilinx:adm-pcie-7v3:1ddr:3.0-0\|cnn\_GOOD | Read | OCL Region | 1 | 1 | 121147 | 121148 | 0.00333333 | 5273.166649 | 5273.166652 |
| 5273.166712 | Kernel\_Read\|xilinx:adm-pcie-7v3:1ddr:3.0-0\|cnn\_GOOD | Read | OCL Region | 1 | 1 | 121166 | 121167 | 0.00333333 | 5273.166712 | 5273.166716 |
| 5273.166782 | Kernel\_Read\|xilinx:adm-pcie-7v3:1ddr:3.0-0\|cnn\_GOOD | Read | OCL Region | 1 | 1 | 121187 | 121188 | 0.00333333 | 5273.166782 | 5273.166786 |
| 5273.166849 | Kernel\_Read\|xilinx:adm-pcie-7v3:1ddr:3.0-0\|cnn\_GOOD | Read | OCL Region | 1 | 1 | 121207 | 121208 | 0.00333333 | 5273.166849 | 5273.166852 |
| 5273.166912 | Kernel\_Read\|xilinx:adm-pcie-7v3:1ddr:3.0-0\|cnn\_GOOD | Read | OCL Region | 1 | 1 | 121226 | 121227 | 0.00333333 | 5273.166912 | 5273.166916 |
| 5273.166996 | Kernel\_Read\|xilinx:adm-pcie-7v3:1ddr:3.0-0\|cnn\_GOOD | Read | OCL Region | 1 | 1 | 121251 | 121252 | 0.00333333 | 5273.166996 | 5273.166999 |
| 5273.167049 | Kernel\_Read\|xilinx:adm-pcie-7v3:1ddr:3.0-0\|cnn\_GOOD | Read | OCL Region | 1 | 1 | 121267 | 121268 | 0.00333333 | 5273.167049 | 5273.167052 |
| 5273.167112 | Kernel\_Read\|xilinx:adm-pcie-7v3:1ddr:3.0-0\|cnn\_GOOD | Read | OCL Region | 1 | 1 | 121286 | 121287 | 0.00333333 | 5273.167112 | 5273.167116 |
| 5273.167182 | Kernel\_Read\|xilinx:adm-pcie-7v3:1ddr:3.0-0\|cnn\_GOOD | Read | OCL Region | 1 | 1 | 121307 | 121308 | 0.00333333 | 5273.167182 | 5273.167186 |
| 5273.167249 | Kernel\_Read\|xilinx:adm-pcie-7v3:1ddr:3.0-0\|cnn\_GOOD | Read | OCL Region | 1 | 1 | 121327 | 121328 | 0.00333333 | 5273.167249 | 5273.167252 |
| 5273.167312 | Kernel\_Read\|xilinx:adm-pcie-7v3:1ddr:3.0-0\|cnn\_GOOD | Read | OCL Region | 1 | 1 | 121346 | 121347 | 0.00333333 | 5273.167312 | 5273.167316 |
| 5273.167382 | Kernel\_Read\|xilinx:adm-pcie-7v3:1ddr:3.0-0\|cnn\_GOOD | Read | OCL Region | 1 | 1 | 121367 | 121368 | 0.00333333 | 5273.167382 | 5273.167386 |
| 5273.167449 | Kernel\_Read\|xilinx:adm-pcie-7v3:1ddr:3.0-0\|cnn\_GOOD | Read | OCL Region | 1 | 1 | 121387 | 121388 | 0.00333333 | 5273.167449 | 5273.167452 |
| 5273.167512 | Kernel\_Read\|xilinx:adm-pcie-7v3:1ddr:3.0-0\|cnn\_GOOD | Read | OCL Region | 1 | 1 | 121406 | 121407 | 0.00333333 | 5273.167512 | 5273.167516 |
| 5273.167582 | Kernel\_Read\|xilinx:adm-pcie-7v3:1ddr:3.0-0\|cnn\_GOOD | Read | OCL Region | 1 | 1 | 121427 | 121428 | 0.00333333 | 5273.167582 | 5273.167586 |
| 5273.167649 | Kernel\_Read\|xilinx:adm-pcie-7v3:1ddr:3.0-0\|cnn\_GOOD | Read | OCL Region | 1 | 1 | 121447 | 121448 | 0.00333333 | 5273.167649 | 5273.167652 |
| 5273.167716 | Kernel\_Read\|xilinx:adm-pcie-7v3:1ddr:3.0-0\|cnn\_GOOD | Read | OCL Region | 1 | 1 | 121467 | 121468 | 0.00333333 | 5273.167716 | 5273.167719 |
| 5273.167782 | Kernel\_Read\|xilinx:adm-pcie-7v3:1ddr:3.0-0\|cnn\_GOOD | Read | OCL Region | 1 | 1 | 121487 | 121488 | 0.00333333 | 5273.167782 | 5273.167786 |
| 5273.167856 | Kernel\_Read\|xilinx:adm-pcie-7v3:1ddr:3.0-0\|cnn\_GOOD | Read | OCL Region | 1 | 1 | 121509 | 121510 | 0.00333333 | 5273.167856 | 5273.167859 |
| 5273.167912 | Kernel\_Read\|xilinx:adm-pcie-7v3:1ddr:3.0-0\|cnn\_GOOD | Read | OCL Region | 1 | 1 | 121526 | 121527 | 0.00333333 | 5273.167912 | 5273.167916 |
| 5273.167982 | Kernel\_Read\|xilinx:adm-pcie-7v3:1ddr:3.0-0\|cnn\_GOOD | Read | OCL Region | 1 | 1 | 121547 | 121548 | 0.00333333 | 5273.167982 | 5273.167986 |
| 5273.168049 | Kernel\_Read\|xilinx:adm-pcie-7v3:1ddr:3.0-0\|cnn\_GOOD | Read | OCL Region | 1 | 1 | 121567 | 121568 | 0.00333333 | 5273.168049 | 5273.168052 |
| 5273.168112 | Kernel\_Read\|xilinx:adm-pcie-7v3:1ddr:3.0-0\|cnn\_GOOD | Read | OCL Region | 1 | 1 | 121586 | 121587 | 0.00333333 | 5273.168112 | 5273.168116 |
| 5273.168389 | Kernel\_Read\|xilinx:adm-pcie-7v3:1ddr:3.0-0\|cnn\_GOOD | Read | OCL Region | 1 | 1 | 121669 | 121670 | 0.00333333 | 5273.168389 | 5273.168392 |
| 5273.168396 | Kernel\_Read\|xilinx:adm-pcie-7v3:1ddr:3.0-0\|cnn\_GOOD | Read | OCL Region | 1 | 1 | 121671 | 121672 | 0.00333333 | 5273.168396 | 5273.168399 |
| 5273.168399 | Kernel\_Read\|xilinx:adm-pcie-7v3:1ddr:3.0-0\|cnn\_GOOD | Read | OCL Region | 1 | 1 | 121672 | 121673 | 0.00333333 | 5273.168399 | 5273.168402 |
| 5273.168406 | Kernel\_Read\|xilinx:adm-pcie-7v3:1ddr:3.0-0\|cnn\_GOOD | Read | OCL Region | 1 | 1 | 121674 | 121675 | 0.00333333 | 5273.168406 | 5273.168409 |
| 5273.168462 | Kernel\_Read\|xilinx:adm-pcie-7v3:1ddr:3.0-0\|cnn\_GOOD | Read | OCL Region | 1 | 1 | 121691 | 121692 | 0.00333333 | 5273.168462 | 5273.168466 |
| 5273.168522 | Kernel\_Read\|xilinx:adm-pcie-7v3:1ddr:3.0-0\|cnn\_GOOD | Read | OCL Region | 1 | 1 | 121709 | 121710 | 0.00333333 | 5273.168522 | 5273.168526 |
| 5273.168582 | Kernel\_Read\|xilinx:adm-pcie-7v3:1ddr:3.0-0\|cnn\_GOOD | Read | OCL Region | 1 | 1 | 121727 | 121728 | 0.00333333 | 5273.168582 | 5273.168586 |
| 5273.168649 | Kernel\_Read\|xilinx:adm-pcie-7v3:1ddr:3.0-0\|cnn\_GOOD | Read | OCL Region | 1 | 1 | 121747 | 121748 | 0.00333333 | 5273.168649 | 5273.168652 |
| 5273.168712 | Kernel\_Read\|xilinx:adm-pcie-7v3:1ddr:3.0-0\|cnn\_GOOD | Read | OCL Region | 1 | 1 | 121766 | 121767 | 0.00333333 | 5273.168712 | 5273.168716 |
| 5273.168782 | Kernel\_Read\|xilinx:adm-pcie-7v3:1ddr:3.0-0\|cnn\_GOOD | Read | OCL Region | 1 | 1 | 121787 | 121788 | 0.00333333 | 5273.168782 | 5273.168786 |
| 5273.168849 | Kernel\_Read\|xilinx:adm-pcie-7v3:1ddr:3.0-0\|cnn\_GOOD | Read | OCL Region | 1 | 1 | 121807 | 121808 | 0.00333333 | 5273.168849 | 5273.168852 |
| 5273.168912 | Kernel\_Read\|xilinx:adm-pcie-7v3:1ddr:3.0-0\|cnn\_GOOD | Read | OCL Region | 1 | 1 | 121826 | 121827 | 0.00333333 | 5273.168912 | 5273.168916 |
| 5273.168982 | Kernel\_Read\|xilinx:adm-pcie-7v3:1ddr:3.0-0\|cnn\_GOOD | Read | OCL Region | 1 | 1 | 121847 | 121848 | 0.00333333 | 5273.168982 | 5273.168986 |
| 5273.169049 | Kernel\_Read\|xilinx:adm-pcie-7v3:1ddr:3.0-0\|cnn\_GOOD | Read | OCL Region | 1 | 1 | 121867 | 121868 | 0.00333333 | 5273.169049 | 5273.169052 |
| 5273.169112 | Kernel\_Read\|xilinx:adm-pcie-7v3:1ddr:3.0-0\|cnn\_GOOD | Read | OCL Region | 1 | 1 | 121886 | 121887 | 0.00333333 | 5273.169112 | 5273.169116 |
| 5273.169182 | Kernel\_Read\|xilinx:adm-pcie-7v3:1ddr:3.0-0\|cnn\_GOOD | Read | OCL Region | 1 | 1 | 121907 | 121908 | 0.00333333 | 5273.169182 | 5273.169186 |
| 5273.169249 | Kernel\_Read\|xilinx:adm-pcie-7v3:1ddr:3.0-0\|cnn\_GOOD | Read | OCL Region | 1 | 1 | 121927 | 121928 | 0.00333333 | 5273.169249 | 5273.169252 |
| 5273.169316 | Kernel\_Read\|xilinx:adm-pcie-7v3:1ddr:3.0-0\|cnn\_GOOD | Read | OCL Region | 1 | 1 | 121947 | 121948 | 0.00333333 | 5273.169316 | 5273.169319 |
| 5273.169382 | Kernel\_Read\|xilinx:adm-pcie-7v3:1ddr:3.0-0\|cnn\_GOOD | Read | OCL Region | 1 | 1 | 121967 | 121968 | 0.00333333 | 5273.169382 | 5273.169386 |
| 5273.169449 | Kernel\_Read\|xilinx:adm-pcie-7v3:1ddr:3.0-0\|cnn\_GOOD | Read | OCL Region | 1 | 1 | 121987 | 121988 | 0.00333333 | 5273.169449 | 5273.169452 |
| 5273.169512 | Kernel\_Read\|xilinx:adm-pcie-7v3:1ddr:3.0-0\|cnn\_GOOD | Read | OCL Region | 1 | 1 | 122006 | 122007 | 0.00333333 | 5273.169512 | 5273.169516 |
| 5273.169582 | Kernel\_Read\|xilinx:adm-pcie-7v3:1ddr:3.0-0\|cnn\_GOOD | Read | OCL Region | 1 | 1 | 122027 | 122028 | 0.00333333 | 5273.169582 | 5273.169586 |
| 5273.169649 | Kernel\_Read\|xilinx:adm-pcie-7v3:1ddr:3.0-0\|cnn\_GOOD | Read | OCL Region | 1 | 1 | 122047 | 122048 | 0.00333333 | 5273.169649 | 5273.169652 |
| 5273.169712 | Kernel\_Read\|xilinx:adm-pcie-7v3:1ddr:3.0-0\|cnn\_GOOD | Read | OCL Region | 1 | 1 | 122066 | 122067 | 0.00333333 | 5273.169712 | 5273.169716 |
| 5273.169782 | Kernel\_Read\|xilinx:adm-pcie-7v3:1ddr:3.0-0\|cnn\_GOOD | Read | OCL Region | 1 | 1 | 122087 | 122088 | 0.00333333 | 5273.169782 | 5273.169786 |
| 5273.169849 | Kernel\_Read\|xilinx:adm-pcie-7v3:1ddr:3.0-0\|cnn\_GOOD | Read | OCL Region | 1 | 1 | 122107 | 122108 | 0.00333333 | 5273.169849 | 5273.169852 |
| 5273.169912 | Kernel\_Read\|xilinx:adm-pcie-7v3:1ddr:3.0-0\|cnn\_GOOD | Read | OCL Region | 1 | 1 | 122126 | 122127 | 0.00333333 | 5273.169912 | 5273.169916 |
| 5273.169979 | Kernel\_Read\|xilinx:adm-pcie-7v3:1ddr:3.0-0\|cnn\_GOOD | Read | OCL Region | 1 | 1 | 122146 | 122147 | 0.00333333 | 5273.169979 | 5273.169982 |
| 5273.170049 | Kernel\_Read\|xilinx:adm-pcie-7v3:1ddr:3.0-0\|cnn\_GOOD | Read | OCL Region | 1 | 1 | 122167 | 122168 | 0.00333333 | 5273.170049 | 5273.170052 |
| 5273.170112 | Kernel\_Read\|xilinx:adm-pcie-7v3:1ddr:3.0-0\|cnn\_GOOD | Read | OCL Region | 1 | 1 | 122186 | 122187 | 0.00333333 | 5273.170112 | 5273.170116 |
| 5273.170182 | Kernel\_Read\|xilinx:adm-pcie-7v3:1ddr:3.0-0\|cnn\_GOOD | Read | OCL Region | 1 | 1 | 122207 | 122208 | 0.00333333 | 5273.170182 | 5273.170186 |
| 5273.170249 | Kernel\_Read\|xilinx:adm-pcie-7v3:1ddr:3.0-0\|cnn\_GOOD | Read | OCL Region | 1 | 1 | 122227 | 122228 | 0.00333333 | 5273.170249 | 5273.170252 |
| 5273.170312 | Kernel\_Read\|xilinx:adm-pcie-7v3:1ddr:3.0-0\|cnn\_GOOD | Read | OCL Region | 1 | 1 | 122246 | 122247 | 0.00333333 | 5273.170312 | 5273.170316 |
| 5273.170382 | Kernel\_Read\|xilinx:adm-pcie-7v3:1ddr:3.0-0\|cnn\_GOOD | Read | OCL Region | 1 | 1 | 122267 | 122268 | 0.00333333 | 5273.170382 | 5273.170386 |
| 5273.170449 | Kernel\_Read\|xilinx:adm-pcie-7v3:1ddr:3.0-0\|cnn\_GOOD | Read | OCL Region | 1 | 1 | 122287 | 122288 | 0.00333333 | 5273.170449 | 5273.170452 |
| 5273.170512 | Kernel\_Read\|xilinx:adm-pcie-7v3:1ddr:3.0-0\|cnn\_GOOD | Read | OCL Region | 1 | 1 | 122306 | 122307 | 0.00333333 | 5273.170512 | 5273.170516 |
| 5273.170582 | Kernel\_Read\|xilinx:adm-pcie-7v3:1ddr:3.0-0\|cnn\_GOOD | Read | OCL Region | 1 | 1 | 122327 | 122328 | 0.00333333 | 5273.170582 | 5273.170586 |
| 5273.170649 | Kernel\_Read\|xilinx:adm-pcie-7v3:1ddr:3.0-0\|cnn\_GOOD | Read | OCL Region | 1 | 1 | 122347 | 122348 | 0.00333333 | 5273.170649 | 5273.170652 |
| 5273.170712 | Kernel\_Read\|xilinx:adm-pcie-7v3:1ddr:3.0-0\|cnn\_GOOD | Read | OCL Region | 1 | 1 | 122366 | 122367 | 0.00333333 | 5273.170712 | 5273.170716 |
| 5273.170789 | Kernel\_Read\|xilinx:adm-pcie-7v3:1ddr:3.0-0\|cnn\_GOOD | Read | OCL Region | 1 | 1 | 122389 | 122390 | 0.00333333 | 5273.170789 | 5273.170792 |
| 5273.170849 | Kernel\_Read\|xilinx:adm-pcie-7v3:1ddr:3.0-0\|cnn\_GOOD | Read | OCL Region | 1 | 1 | 122407 | 122408 | 0.00333333 | 5273.170849 | 5273.170852 |
| 5273.170916 | Kernel\_Read\|xilinx:adm-pcie-7v3:1ddr:3.0-0\|cnn\_GOOD | Read | OCL Region | 1 | 1 | 122427 | 122428 | 0.00333333 | 5273.170916 | 5273.170919 |
| 5273.170982 | Kernel\_Read\|xilinx:adm-pcie-7v3:1ddr:3.0-0\|cnn\_GOOD | Read | OCL Region | 1 | 1 | 122447 | 122448 | 0.00333333 | 5273.170982 | 5273.170986 |
| 5273.171049 | Kernel\_Read\|xilinx:adm-pcie-7v3:1ddr:3.0-0\|cnn\_GOOD | Read | OCL Region | 1 | 1 | 122467 | 122468 | 0.00333333 | 5273.171049 | 5273.171052 |
| 5273.171112 | Kernel\_Read\|xilinx:adm-pcie-7v3:1ddr:3.0-0\|cnn\_GOOD | Read | OCL Region | 1 | 1 | 122486 | 122487 | 0.00333333 | 5273.171112 | 5273.171116 |
| 5273.171182 | Kernel\_Read\|xilinx:adm-pcie-7v3:1ddr:3.0-0\|cnn\_GOOD | Read | OCL Region | 1 | 1 | 122507 | 122508 | 0.00333333 | 5273.171182 | 5273.171186 |
| 5273.171249 | Kernel\_Read\|xilinx:adm-pcie-7v3:1ddr:3.0-0\|cnn\_GOOD | Read | OCL Region | 1 | 1 | 122527 | 122528 | 0.00333333 | 5273.171249 | 5273.171252 |
| 5273.171316 | Kernel\_Read\|xilinx:adm-pcie-7v3:1ddr:3.0-0\|cnn\_GOOD | Read | OCL Region | 1 | 1 | 122547 | 122548 | 0.00333333 | 5273.171316 | 5273.171319 |
| 5273.171382 | Kernel\_Read\|xilinx:adm-pcie-7v3:1ddr:3.0-0\|cnn\_GOOD | Read | OCL Region | 1 | 1 | 122567 | 122568 | 0.00333333 | 5273.171382 | 5273.171386 |
| 5273.171449 | Kernel\_Read\|xilinx:adm-pcie-7v3:1ddr:3.0-0\|cnn\_GOOD | Read | OCL Region | 1 | 1 | 122587 | 122588 | 0.00333333 | 5273.171449 | 5273.171452 |
| 5273.171512 | Kernel\_Read\|xilinx:adm-pcie-7v3:1ddr:3.0-0\|cnn\_GOOD | Read | OCL Region | 1 | 1 | 122606 | 122607 | 0.00333333 | 5273.171512 | 5273.171516 |
| 5273.171582 | Kernel\_Read\|xilinx:adm-pcie-7v3:1ddr:3.0-0\|cnn\_GOOD | Read | OCL Region | 1 | 1 | 122627 | 122628 | 0.00333333 | 5273.171582 | 5273.171586 |
| 5273.171649 | Kernel\_Read\|xilinx:adm-pcie-7v3:1ddr:3.0-0\|cnn\_GOOD | Read | OCL Region | 1 | 1 | 122647 | 122648 | 0.00333333 | 5273.171649 | 5273.171652 |
| 5273.171716 | Kernel\_Read\|xilinx:adm-pcie-7v3:1ddr:3.0-0\|cnn\_GOOD | Read | OCL Region | 1 | 1 | 122667 | 122668 | 0.00333333 | 5273.171716 | 5273.171719 |
| 5273.171782 | Kernel\_Read\|xilinx:adm-pcie-7v3:1ddr:3.0-0\|cnn\_GOOD | Read | OCL Region | 1 | 1 | 122687 | 122688 | 0.00333333 | 5273.171782 | 5273.171786 |
| 5273.171849 | Kernel\_Read\|xilinx:adm-pcie-7v3:1ddr:3.0-0\|cnn\_GOOD | Read | OCL Region | 1 | 1 | 122707 | 122708 | 0.00333333 | 5273.171849 | 5273.171852 |
| 5273.171912 | Kernel\_Read\|xilinx:adm-pcie-7v3:1ddr:3.0-0\|cnn\_GOOD | Read | OCL Region | 1 | 1 | 122726 | 122727 | 0.00333333 | 5273.171912 | 5273.171916 |
| 5273.171982 | Kernel\_Read\|xilinx:adm-pcie-7v3:1ddr:3.0-0\|cnn\_GOOD | Read | OCL Region | 1 | 1 | 122747 | 122748 | 0.00333333 | 5273.171982 | 5273.171986 |
| 5273.172049 | Kernel\_Read\|xilinx:adm-pcie-7v3:1ddr:3.0-0\|cnn\_GOOD | Read | OCL Region | 1 | 1 | 122767 | 122768 | 0.00333333 | 5273.172049 | 5273.172052 |
| 5273.172112 | Kernel\_Read\|xilinx:adm-pcie-7v3:1ddr:3.0-0\|cnn\_GOOD | Read | OCL Region | 1 | 1 | 122786 | 122787 | 0.00333333 | 5273.172112 | 5273.172116 |
| 5273.172182 | Kernel\_Read\|xilinx:adm-pcie-7v3:1ddr:3.0-0\|cnn\_GOOD | Read | OCL Region | 1 | 1 | 122807 | 122808 | 0.00333333 | 5273.172182 | 5273.172186 |
| 5273.172249 | Kernel\_Read\|xilinx:adm-pcie-7v3:1ddr:3.0-0\|cnn\_GOOD | Read | OCL Region | 1 | 1 | 122827 | 122828 | 0.00333333 | 5273.172249 | 5273.172252 |
| 5273.172312 | Kernel\_Read\|xilinx:adm-pcie-7v3:1ddr:3.0-0\|cnn\_GOOD | Read | OCL Region | 1 | 1 | 122846 | 122847 | 0.00333333 | 5273.172312 | 5273.172316 |
| 5273.172382 | Kernel\_Read\|xilinx:adm-pcie-7v3:1ddr:3.0-0\|cnn\_GOOD | Read | OCL Region | 1 | 1 | 122867 | 122868 | 0.00333333 | 5273.172382 | 5273.172386 |
| 5273.172449 | Kernel\_Read\|xilinx:adm-pcie-7v3:1ddr:3.0-0\|cnn\_GOOD | Read | OCL Region | 1 | 1 | 122887 | 122888 | 0.00333333 | 5273.172449 | 5273.172452 |
| 5273.172516 | Kernel\_Read\|xilinx:adm-pcie-7v3:1ddr:3.0-0\|cnn\_GOOD | Read | OCL Region | 1 | 1 | 122907 | 122908 | 0.00333333 | 5273.172516 | 5273.172519 |
| 5273.172582 | Kernel\_Read\|xilinx:adm-pcie-7v3:1ddr:3.0-0\|cnn\_GOOD | Read | OCL Region | 1 | 1 | 122927 | 122928 | 0.00333333 | 5273.172582 | 5273.172586 |
| 5273.172649 | Kernel\_Read\|xilinx:adm-pcie-7v3:1ddr:3.0-0\|cnn\_GOOD | Read | OCL Region | 1 | 1 | 122947 | 122948 | 0.00333333 | 5273.172649 | 5273.172652 |
| 5273.172712 | Kernel\_Read\|xilinx:adm-pcie-7v3:1ddr:3.0-0\|cnn\_GOOD | Read | OCL Region | 1 | 1 | 122966 | 122967 | 0.00333333 | 5273.172712 | 5273.172716 |
| 5273.172782 | Kernel\_Read\|xilinx:adm-pcie-7v3:1ddr:3.0-0\|cnn\_GOOD | Read | OCL Region | 1 | 1 | 122987 | 122988 | 0.00333333 | 5273.172782 | 5273.172786 |
| 5273.172849 | Kernel\_Read\|xilinx:adm-pcie-7v3:1ddr:3.0-0\|cnn\_GOOD | Read | OCL Region | 1 | 1 | 123007 | 123008 | 0.00333333 | 5273.172849 | 5273.172852 |
| 5273.172912 | Kernel\_Read\|xilinx:adm-pcie-7v3:1ddr:3.0-0\|cnn\_GOOD | Read | OCL Region | 1 | 1 | 123026 | 123027 | 0.00333333 | 5273.172912 | 5273.172916 |
| 5273.172982 | Kernel\_Read\|xilinx:adm-pcie-7v3:1ddr:3.0-0\|cnn\_GOOD | Read | OCL Region | 1 | 1 | 123047 | 123048 | 0.00333333 | 5273.172982 | 5273.172986 |
| 5273.173049 | Kernel\_Read\|xilinx:adm-pcie-7v3:1ddr:3.0-0\|cnn\_GOOD | Read | OCL Region | 1 | 1 | 123067 | 123068 | 0.00333333 | 5273.173049 | 5273.173052 |
| 5273.173112 | Kernel\_Read\|xilinx:adm-pcie-7v3:1ddr:3.0-0\|cnn\_GOOD | Read | OCL Region | 1 | 1 | 123086 | 123087 | 0.00333333 | 5273.173112 | 5273.173116 |
| 5273.173182 | Kernel\_Read\|xilinx:adm-pcie-7v3:1ddr:3.0-0\|cnn\_GOOD | Read | OCL Region | 1 | 1 | 123107 | 123108 | 0.00333333 | 5273.173182 | 5273.173186 |
| 5273.173249 | Kernel\_Read\|xilinx:adm-pcie-7v3:1ddr:3.0-0\|cnn\_GOOD | Read | OCL Region | 1 | 1 | 123127 | 123128 | 0.00333333 | 5273.173249 | 5273.173252 |
| 5273.173316 | Kernel\_Read\|xilinx:adm-pcie-7v3:1ddr:3.0-0\|cnn\_GOOD | Read | OCL Region | 1 | 1 | 123147 | 123148 | 0.00333333 | 5273.173316 | 5273.173319 |
| 5273.173382 | Kernel\_Read\|xilinx:adm-pcie-7v3:1ddr:3.0-0\|cnn\_GOOD | Read | OCL Region | 1 | 1 | 123167 | 123168 | 0.00333333 | 5273.173382 | 5273.173386 |
| 5273.173449 | Kernel\_Read\|xilinx:adm-pcie-7v3:1ddr:3.0-0\|cnn\_GOOD | Read | OCL Region | 1 | 1 | 123187 | 123188 | 0.00333333 | 5273.173449 | 5273.173452 |
| 5273.173512 | Kernel\_Read\|xilinx:adm-pcie-7v3:1ddr:3.0-0\|cnn\_GOOD | Read | OCL Region | 1 | 1 | 123206 | 123207 | 0.00333333 | 5273.173512 | 5273.173516 |
| 5273.173582 | Kernel\_Read\|xilinx:adm-pcie-7v3:1ddr:3.0-0\|cnn\_GOOD | Read | OCL Region | 1 | 1 | 123227 | 123228 | 0.00333333 | 5273.173582 | 5273.173586 |
| 5273.173649 | Kernel\_Read\|xilinx:adm-pcie-7v3:1ddr:3.0-0\|cnn\_GOOD | Read | OCL Region | 1 | 1 | 123247 | 123248 | 0.00333333 | 5273.173649 | 5273.173652 |
| 5273.173712 | Kernel\_Read\|xilinx:adm-pcie-7v3:1ddr:3.0-0\|cnn\_GOOD | Read | OCL Region | 1 | 1 | 123266 | 123267 | 0.00333333 | 5273.173712 | 5273.173716 |
| 5273.173782 | Kernel\_Read\|xilinx:adm-pcie-7v3:1ddr:3.0-0\|cnn\_GOOD | Read | OCL Region | 1 | 1 | 123287 | 123288 | 0.00333333 | 5273.173782 | 5273.173786 |
| 5273.173849 | Kernel\_Read\|xilinx:adm-pcie-7v3:1ddr:3.0-0\|cnn\_GOOD | Read | OCL Region | 1 | 1 | 123307 | 123308 | 0.00333333 | 5273.173849 | 5273.173852 |
| 5273.173912 | Kernel\_Read\|xilinx:adm-pcie-7v3:1ddr:3.0-0\|cnn\_GOOD | Read | OCL Region | 1 | 1 | 123326 | 123327 | 0.00333333 | 5273.173912 | 5273.173916 |
| 5273.173982 | Kernel\_Read\|xilinx:adm-pcie-7v3:1ddr:3.0-0\|cnn\_GOOD | Read | OCL Region | 1 | 1 | 123347 | 123348 | 0.00333333 | 5273.173982 | 5273.173986 |
| 5273.174049 | Kernel\_Read\|xilinx:adm-pcie-7v3:1ddr:3.0-0\|cnn\_GOOD | Read | OCL Region | 1 | 1 | 123367 | 123368 | 0.00333333 | 5273.174049 | 5273.174052 |
| 5273.174126 | Kernel\_Read\|xilinx:adm-pcie-7v3:1ddr:3.0-0\|cnn\_GOOD | Read | OCL Region | 1 | 1 | 123390 | 123391 | 0.00333333 | 5273.174126 | 5273.174129 |
| 5273.174182 | Kernel\_Read\|xilinx:adm-pcie-7v3:1ddr:3.0-0\|cnn\_GOOD | Read | OCL Region | 1 | 1 | 123407 | 123408 | 0.00333333 | 5273.174182 | 5273.174186 |
| 5273.174249 | Kernel\_Read\|xilinx:adm-pcie-7v3:1ddr:3.0-0\|cnn\_GOOD | Read | OCL Region | 1 | 1 | 123427 | 123428 | 0.00333333 | 5273.174249 | 5273.174252 |
| 5273.174312 | Kernel\_Read\|xilinx:adm-pcie-7v3:1ddr:3.0-0\|cnn\_GOOD | Read | OCL Region | 1 | 1 | 123446 | 123447 | 0.00333333 | 5273.174312 | 5273.174316 |
| 5273.174382 | Kernel\_Read\|xilinx:adm-pcie-7v3:1ddr:3.0-0\|cnn\_GOOD | Read | OCL Region | 1 | 1 | 123467 | 123468 | 0.00333333 | 5273.174382 | 5273.174386 |
| 5273.174659 | Kernel\_Read\|xilinx:adm-pcie-7v3:1ddr:3.0-0\|cnn\_GOOD | Read | OCL Region | 1 | 1 | 123550 | 123551 | 0.00333333 | 5273.174659 | 5273.174662 |
| 5273.174662 | Kernel\_Read\|xilinx:adm-pcie-7v3:1ddr:3.0-0\|cnn\_GOOD | Read | OCL Region | 1 | 1 | 123551 | 123552 | 0.00333333 | 5273.174662 | 5273.174666 |
| 5273.174712 | Kernel\_Read\|xilinx:adm-pcie-7v3:1ddr:3.0-0\|cnn\_GOOD | Read | OCL Region | 1 | 1 | 123566 | 123567 | 0.00333333 | 5273.174712 | 5273.174716 |
| 5273.174722 | Kernel\_Read\|xilinx:adm-pcie-7v3:1ddr:3.0-0\|cnn\_GOOD | Read | OCL Region | 1 | 1 | 123569 | 123570 | 0.00333333 | 5273.174722 | 5273.174726 |
| 5273.174726 | Kernel\_Read\|xilinx:adm-pcie-7v3:1ddr:3.0-0\|cnn\_GOOD | Read | OCL Region | 1 | 1 | 123570 | 123571 | 0.00333333 | 5273.174726 | 5273.174729 |
| 5273.174782 | Kernel\_Read\|xilinx:adm-pcie-7v3:1ddr:3.0-0\|cnn\_GOOD | Read | OCL Region | 1 | 1 | 123587 | 123588 | 0.00333333 | 5273.174782 | 5273.174786 |
| 5273.174849 | Kernel\_Read\|xilinx:adm-pcie-7v3:1ddr:3.0-0\|cnn\_GOOD | Read | OCL Region | 1 | 1 | 123607 | 123608 | 0.00333333 | 5273.174849 | 5273.174852 |
| 5273.174916 | Kernel\_Read\|xilinx:adm-pcie-7v3:1ddr:3.0-0\|cnn\_GOOD | Read | OCL Region | 1 | 1 | 123627 | 123628 | 0.00333333 | 5273.174916 | 5273.174919 |
| 5273.174982 | Kernel\_Read\|xilinx:adm-pcie-7v3:1ddr:3.0-0\|cnn\_GOOD | Read | OCL Region | 1 | 1 | 123647 | 123648 | 0.00333333 | 5273.174982 | 5273.174986 |
| 5273.175049 | Kernel\_Read\|xilinx:adm-pcie-7v3:1ddr:3.0-0\|cnn\_GOOD | Read | OCL Region | 1 | 1 | 123667 | 123668 | 0.00333333 | 5273.175049 | 5273.175052 |
| 5273.175112 | Kernel\_Read\|xilinx:adm-pcie-7v3:1ddr:3.0-0\|cnn\_GOOD | Read | OCL Region | 1 | 1 | 123686 | 123687 | 0.00333333 | 5273.175112 | 5273.175116 |
| 5273.175182 | Kernel\_Read\|xilinx:adm-pcie-7v3:1ddr:3.0-0\|cnn\_GOOD | Read | OCL Region | 1 | 1 | 123707 | 123708 | 0.00333333 | 5273.175182 | 5273.175186 |
| 5273.175249 | Kernel\_Read\|xilinx:adm-pcie-7v3:1ddr:3.0-0\|cnn\_GOOD | Read | OCL Region | 1 | 1 | 123727 | 123728 | 0.00333333 | 5273.175249 | 5273.175252 |
| 5273.175312 | Kernel\_Read\|xilinx:adm-pcie-7v3:1ddr:3.0-0\|cnn\_GOOD | Read | OCL Region | 1 | 1 | 123746 | 123747 | 0.00333333 | 5273.175312 | 5273.175316 |
| 5273.175382 | Kernel\_Read\|xilinx:adm-pcie-7v3:1ddr:3.0-0\|cnn\_GOOD | Read | OCL Region | 1 | 1 | 123767 | 123768 | 0.00333333 | 5273.175382 | 5273.175386 |
| 5273.175449 | Kernel\_Read\|xilinx:adm-pcie-7v3:1ddr:3.0-0\|cnn\_GOOD | Read | OCL Region | 1 | 1 | 123787 | 123788 | 0.00333333 | 5273.175449 | 5273.175452 |
| 5273.175512 | Kernel\_Read\|xilinx:adm-pcie-7v3:1ddr:3.0-0\|cnn\_GOOD | Read | OCL Region | 1 | 1 | 123806 | 123807 | 0.00333333 | 5273.175512 | 5273.175516 |
| 5273.175579 | Kernel\_Read\|xilinx:adm-pcie-7v3:1ddr:3.0-0\|cnn\_GOOD | Read | OCL Region | 1 | 1 | 123826 | 123827 | 0.00333333 | 5273.175579 | 5273.175582 |
| 5273.175649 | Kernel\_Read\|xilinx:adm-pcie-7v3:1ddr:3.0-0\|cnn\_GOOD | Read | OCL Region | 1 | 1 | 123847 | 123848 | 0.00333333 | 5273.175649 | 5273.175652 |
| 5273.175716 | Kernel\_Read\|xilinx:adm-pcie-7v3:1ddr:3.0-0\|cnn\_GOOD | Read | OCL Region | 1 | 1 | 123867 | 123868 | 0.00333333 | 5273.175716 | 5273.175719 |
| 5273.175782 | Kernel\_Read\|xilinx:adm-pcie-7v3:1ddr:3.0-0\|cnn\_GOOD | Read | OCL Region | 1 | 1 | 123887 | 123888 | 0.00333333 | 5273.175782 | 5273.175786 |
| 5273.175849 | Kernel\_Read\|xilinx:adm-pcie-7v3:1ddr:3.0-0\|cnn\_GOOD | Read | OCL Region | 1 | 1 | 123907 | 123908 | 0.00333333 | 5273.175849 | 5273.175852 |
| 5273.175912 | Kernel\_Read\|xilinx:adm-pcie-7v3:1ddr:3.0-0\|cnn\_GOOD | Read | OCL Region | 1 | 1 | 123926 | 123927 | 0.00333333 | 5273.175912 | 5273.175916 |
| 5273.175982 | Kernel\_Read\|xilinx:adm-pcie-7v3:1ddr:3.0-0\|cnn\_GOOD | Read | OCL Region | 1 | 1 | 123947 | 123948 | 0.00333333 | 5273.175982 | 5273.175986 |
| 5273.176049 | Kernel\_Read\|xilinx:adm-pcie-7v3:1ddr:3.0-0\|cnn\_GOOD | Read | OCL Region | 1 | 1 | 123967 | 123968 | 0.00333333 | 5273.176049 | 5273.176052 |
| 5273.176112 | Kernel\_Read\|xilinx:adm-pcie-7v3:1ddr:3.0-0\|cnn\_GOOD | Read | OCL Region | 1 | 1 | 123986 | 123987 | 0.00333333 | 5273.176112 | 5273.176116 |
| 5273.176182 | Kernel\_Read\|xilinx:adm-pcie-7v3:1ddr:3.0-0\|cnn\_GOOD | Read | OCL Region | 1 | 1 | 124007 | 124008 | 0.00333333 | 5273.176182 | 5273.176186 |
| 5273.176249 | Kernel\_Read\|xilinx:adm-pcie-7v3:1ddr:3.0-0\|cnn\_GOOD | Read | OCL Region | 1 | 1 | 124027 | 124028 | 0.00333333 | 5273.176249 | 5273.176252 |
| 5273.176312 | Kernel\_Read\|xilinx:adm-pcie-7v3:1ddr:3.0-0\|cnn\_GOOD | Read | OCL Region | 1 | 1 | 124046 | 124047 | 0.00333333 | 5273.176312 | 5273.176316 |
| 5273.176382 | Kernel\_Read\|xilinx:adm-pcie-7v3:1ddr:3.0-0\|cnn\_GOOD | Read | OCL Region | 1 | 1 | 124067 | 124068 | 0.00333333 | 5273.176382 | 5273.176386 |
| 5273.176449 | Kernel\_Read\|xilinx:adm-pcie-7v3:1ddr:3.0-0\|cnn\_GOOD | Read | OCL Region | 1 | 1 | 124087 | 124088 | 0.00333333 | 5273.176449 | 5273.176452 |
| 5273.176512 | Kernel\_Read\|xilinx:adm-pcie-7v3:1ddr:3.0-0\|cnn\_GOOD | Read | OCL Region | 1 | 1 | 124106 | 124107 | 0.00333333 | 5273.176512 | 5273.176516 |
| 5273.176582 | Kernel\_Read\|xilinx:adm-pcie-7v3:1ddr:3.0-0\|cnn\_GOOD | Read | OCL Region | 1 | 1 | 124127 | 124128 | 0.00333333 | 5273.176582 | 5273.176586 |
| 5273.176649 | Kernel\_Read\|xilinx:adm-pcie-7v3:1ddr:3.0-0\|cnn\_GOOD | Read | OCL Region | 1 | 1 | 124147 | 124148 | 0.00333333 | 5273.176649 | 5273.176652 |
| 5273.176712 | Kernel\_Read\|xilinx:adm-pcie-7v3:1ddr:3.0-0\|cnn\_GOOD | Read | OCL Region | 1 | 1 | 124166 | 124167 | 0.00333333 | 5273.176712 | 5273.176716 |
| 5273.176782 | Kernel\_Read\|xilinx:adm-pcie-7v3:1ddr:3.0-0\|cnn\_GOOD | Read | OCL Region | 1 | 1 | 124187 | 124188 | 0.00333333 | 5273.176782 | 5273.176786 |
| 5273.176849 | Kernel\_Read\|xilinx:adm-pcie-7v3:1ddr:3.0-0\|cnn\_GOOD | Read | OCL Region | 1 | 1 | 124207 | 124208 | 0.00333333 | 5273.176849 | 5273.176852 |
| 5273.176912 | Kernel\_Read\|xilinx:adm-pcie-7v3:1ddr:3.0-0\|cnn\_GOOD | Read | OCL Region | 1 | 1 | 124226 | 124227 | 0.00333333 | 5273.176912 | 5273.176916 |
| 5273.176982 | Kernel\_Read\|xilinx:adm-pcie-7v3:1ddr:3.0-0\|cnn\_GOOD | Read | OCL Region | 1 | 1 | 124247 | 124248 | 0.00333333 | 5273.176982 | 5273.176986 |
| 5273.177049 | Kernel\_Read\|xilinx:adm-pcie-7v3:1ddr:3.0-0\|cnn\_GOOD | Read | OCL Region | 1 | 1 | 124267 | 124268 | 0.00333333 | 5273.177049 | 5273.177052 |
| 5273.177112 | Kernel\_Read\|xilinx:adm-pcie-7v3:1ddr:3.0-0\|cnn\_GOOD | Read | OCL Region | 1 | 1 | 124286 | 124287 | 0.00333333 | 5273.177112 | 5273.177116 |
| 5273.177182 | Kernel\_Read\|xilinx:adm-pcie-7v3:1ddr:3.0-0\|cnn\_GOOD | Read | OCL Region | 1 | 1 | 124307 | 124308 | 0.00333333 | 5273.177182 | 5273.177186 |
| 5273.177259 | Kernel\_Read\|xilinx:adm-pcie-7v3:1ddr:3.0-0\|cnn\_GOOD | Read | OCL Region | 1 | 1 | 124330 | 124331 | 0.00333333 | 5273.177259 | 5273.177262 |
| 5273.177316 | Kernel\_Read\|xilinx:adm-pcie-7v3:1ddr:3.0-0\|cnn\_GOOD | Read | OCL Region | 1 | 1 | 124347 | 124348 | 0.00333333 | 5273.177316 | 5273.177319 |
| 5273.177382 | Kernel\_Read\|xilinx:adm-pcie-7v3:1ddr:3.0-0\|cnn\_GOOD | Read | OCL Region | 1 | 1 | 124367 | 124368 | 0.00333333 | 5273.177382 | 5273.177386 |
| 5273.177449 | Kernel\_Read\|xilinx:adm-pcie-7v3:1ddr:3.0-0\|cnn\_GOOD | Read | OCL Region | 1 | 1 | 124387 | 124388 | 0.00333333 | 5273.177449 | 5273.177452 |
| 5273.177512 | Kernel\_Read\|xilinx:adm-pcie-7v3:1ddr:3.0-0\|cnn\_GOOD | Read | OCL Region | 1 | 1 | 124406 | 124407 | 0.00333333 | 5273.177512 | 5273.177516 |
| 5273.177582 | Kernel\_Read\|xilinx:adm-pcie-7v3:1ddr:3.0-0\|cnn\_GOOD | Read | OCL Region | 1 | 1 | 124427 | 124428 | 0.00333333 | 5273.177582 | 5273.177586 |
| 5273.177649 | Kernel\_Read\|xilinx:adm-pcie-7v3:1ddr:3.0-0\|cnn\_GOOD | Read | OCL Region | 1 | 1 | 124447 | 124448 | 0.00333333 | 5273.177649 | 5273.177652 |
| 5273.177712 | Kernel\_Read\|xilinx:adm-pcie-7v3:1ddr:3.0-0\|cnn\_GOOD | Read | OCL Region | 1 | 1 | 124466 | 124467 | 0.00333333 | 5273.177712 | 5273.177716 |
| 5273.177782 | Kernel\_Read\|xilinx:adm-pcie-7v3:1ddr:3.0-0\|cnn\_GOOD | Read | OCL Region | 1 | 1 | 124487 | 124488 | 0.00333333 | 5273.177782 | 5273.177786 |
| 5273.177849 | Kernel\_Read\|xilinx:adm-pcie-7v3:1ddr:3.0-0\|cnn\_GOOD | Read | OCL Region | 1 | 1 | 124507 | 124508 | 0.00333333 | 5273.177849 | 5273.177852 |
| 5273.177912 | Kernel\_Read\|xilinx:adm-pcie-7v3:1ddr:3.0-0\|cnn\_GOOD | Read | OCL Region | 1 | 1 | 124526 | 124527 | 0.00333333 | 5273.177912 | 5273.177916 |
| 5273.177982 | Kernel\_Read\|xilinx:adm-pcie-7v3:1ddr:3.0-0\|cnn\_GOOD | Read | OCL Region | 1 | 1 | 124547 | 124548 | 0.00333333 | 5273.177982 | 5273.177986 |
| 5273.178049 | Kernel\_Read\|xilinx:adm-pcie-7v3:1ddr:3.0-0\|cnn\_GOOD | Read | OCL Region | 1 | 1 | 124567 | 124568 | 0.00333333 | 5273.178049 | 5273.178052 |
| 5273.178116 | Kernel\_Read\|xilinx:adm-pcie-7v3:1ddr:3.0-0\|cnn\_GOOD | Read | OCL Region | 1 | 1 | 124587 | 124588 | 0.00333333 | 5273.178116 | 5273.178119 |
| 5273.178182 | Kernel\_Read\|xilinx:adm-pcie-7v3:1ddr:3.0-0\|cnn\_GOOD | Read | OCL Region | 1 | 1 | 124607 | 124608 | 0.00333333 | 5273.178182 | 5273.178186 |
| 5273.178249 | Kernel\_Read\|xilinx:adm-pcie-7v3:1ddr:3.0-0\|cnn\_GOOD | Read | OCL Region | 1 | 1 | 124627 | 124628 | 0.00333333 | 5273.178249 | 5273.178252 |
| 5273.178312 | Kernel\_Read\|xilinx:adm-pcie-7v3:1ddr:3.0-0\|cnn\_GOOD | Read | OCL Region | 1 | 1 | 124646 | 124647 | 0.00333333 | 5273.178312 | 5273.178316 |
| 5273.178382 | Kernel\_Read\|xilinx:adm-pcie-7v3:1ddr:3.0-0\|cnn\_GOOD | Read | OCL Region | 1 | 1 | 124667 | 124668 | 0.00333333 | 5273.178382 | 5273.178386 |
| 5273.178449 | Kernel\_Read\|xilinx:adm-pcie-7v3:1ddr:3.0-0\|cnn\_GOOD | Read | OCL Region | 1 | 1 | 124687 | 124688 | 0.00333333 | 5273.178449 | 5273.178452 |
| 5273.178512 | Kernel\_Read\|xilinx:adm-pcie-7v3:1ddr:3.0-0\|cnn\_GOOD | Read | OCL Region | 1 | 1 | 124706 | 124707 | 0.00333333 | 5273.178512 | 5273.178516 |
| 5273.178582 | Kernel\_Read\|xilinx:adm-pcie-7v3:1ddr:3.0-0\|cnn\_GOOD | Read | OCL Region | 1 | 1 | 124727 | 124728 | 0.00333333 | 5273.178582 | 5273.178586 |
| 5273.178649 | Kernel\_Read\|xilinx:adm-pcie-7v3:1ddr:3.0-0\|cnn\_GOOD | Read | OCL Region | 1 | 1 | 124747 | 124748 | 0.00333333 | 5273.178649 | 5273.178652 |
| 5273.178712 | Kernel\_Read\|xilinx:adm-pcie-7v3:1ddr:3.0-0\|cnn\_GOOD | Read | OCL Region | 1 | 1 | 124766 | 124767 | 0.00333333 | 5273.178712 | 5273.178716 |
| 5273.178782 | Kernel\_Read\|xilinx:adm-pcie-7v3:1ddr:3.0-0\|cnn\_GOOD | Read | OCL Region | 1 | 1 | 124787 | 124788 | 0.00333333 | 5273.178782 | 5273.178786 |
| 5273.178849 | Kernel\_Read\|xilinx:adm-pcie-7v3:1ddr:3.0-0\|cnn\_GOOD | Read | OCL Region | 1 | 1 | 124807 | 124808 | 0.00333333 | 5273.178849 | 5273.178852 |
| 5273.178916 | Kernel\_Read\|xilinx:adm-pcie-7v3:1ddr:3.0-0\|cnn\_GOOD | Read | OCL Region | 1 | 1 | 124827 | 124828 | 0.00333333 | 5273.178916 | 5273.178919 |
| 5273.178982 | Kernel\_Read\|xilinx:adm-pcie-7v3:1ddr:3.0-0\|cnn\_GOOD | Read | OCL Region | 1 | 1 | 124847 | 124848 | 0.00333333 | 5273.178982 | 5273.178986 |
| 5273.179049 | Kernel\_Read\|xilinx:adm-pcie-7v3:1ddr:3.0-0\|cnn\_GOOD | Read | OCL Region | 1 | 1 | 124867 | 124868 | 0.00333333 | 5273.179049 | 5273.179052 |
| 5273.179112 | Kernel\_Read\|xilinx:adm-pcie-7v3:1ddr:3.0-0\|cnn\_GOOD | Read | OCL Region | 1 | 1 | 124886 | 124887 | 0.00333333 | 5273.179112 | 5273.179116 |
| 5273.179182 | Kernel\_Read\|xilinx:adm-pcie-7v3:1ddr:3.0-0\|cnn\_GOOD | Read | OCL Region | 1 | 1 | 124907 | 124908 | 0.00333333 | 5273.179182 | 5273.179186 |
| 5273.179249 | Kernel\_Read\|xilinx:adm-pcie-7v3:1ddr:3.0-0\|cnn\_GOOD | Read | OCL Region | 1 | 1 | 124927 | 124928 | 0.00333333 | 5273.179249 | 5273.179252 |
| 5273.179312 | Kernel\_Read\|xilinx:adm-pcie-7v3:1ddr:3.0-0\|cnn\_GOOD | Read | OCL Region | 1 | 1 | 124946 | 124947 | 0.00333333 | 5273.179312 | 5273.179316 |
| 5273.179382 | Kernel\_Read\|xilinx:adm-pcie-7v3:1ddr:3.0-0\|cnn\_GOOD | Read | OCL Region | 1 | 1 | 124967 | 124968 | 0.00333333 | 5273.179382 | 5273.179386 |
| 5273.179449 | Kernel\_Read\|xilinx:adm-pcie-7v3:1ddr:3.0-0\|cnn\_GOOD | Read | OCL Region | 1 | 1 | 124987 | 124988 | 0.00333333 | 5273.179449 | 5273.179452 |
| 5273.179512 | Kernel\_Read\|xilinx:adm-pcie-7v3:1ddr:3.0-0\|cnn\_GOOD | Read | OCL Region | 1 | 1 | 125006 | 125007 | 0.00333333 | 5273.179512 | 5273.179516 |
| 5273.179582 | Kernel\_Read\|xilinx:adm-pcie-7v3:1ddr:3.0-0\|cnn\_GOOD | Read | OCL Region | 1 | 1 | 125027 | 125028 | 0.00333333 | 5273.179582 | 5273.179586 |
| 5273.179649 | Kernel\_Read\|xilinx:adm-pcie-7v3:1ddr:3.0-0\|cnn\_GOOD | Read | OCL Region | 1 | 1 | 125047 | 125048 | 0.00333333 | 5273.179649 | 5273.179652 |
| 5273.179716 | Kernel\_Read\|xilinx:adm-pcie-7v3:1ddr:3.0-0\|cnn\_GOOD | Read | OCL Region | 1 | 1 | 125067 | 125068 | 0.00333333 | 5273.179716 | 5273.179719 |
| 5273.179782 | Kernel\_Read\|xilinx:adm-pcie-7v3:1ddr:3.0-0\|cnn\_GOOD | Read | OCL Region | 1 | 1 | 125087 | 125088 | 0.00333333 | 5273.179782 | 5273.179786 |
| 5273.179849 | Kernel\_Read\|xilinx:adm-pcie-7v3:1ddr:3.0-0\|cnn\_GOOD | Read | OCL Region | 1 | 1 | 125107 | 125108 | 0.00333333 | 5273.179849 | 5273.179852 |
| 5273.179912 | Kernel\_Read\|xilinx:adm-pcie-7v3:1ddr:3.0-0\|cnn\_GOOD | Read | OCL Region | 1 | 1 | 125126 | 125127 | 0.00333333 | 5273.179912 | 5273.179916 |
| 5273.179982 | Kernel\_Read\|xilinx:adm-pcie-7v3:1ddr:3.0-0\|cnn\_GOOD | Read | OCL Region | 1 | 1 | 125147 | 125148 | 0.00333333 | 5273.179982 | 5273.179986 |
| 5273.180049 | Kernel\_Read\|xilinx:adm-pcie-7v3:1ddr:3.0-0\|cnn\_GOOD | Read | OCL Region | 1 | 1 | 125167 | 125168 | 0.00333333 | 5273.180049 | 5273.180052 |
| 5273.180112 | Kernel\_Read\|xilinx:adm-pcie-7v3:1ddr:3.0-0\|cnn\_GOOD | Read | OCL Region | 1 | 1 | 125186 | 125187 | 0.00333333 | 5273.180112 | 5273.180116 |
| 5273.180182 | Kernel\_Read\|xilinx:adm-pcie-7v3:1ddr:3.0-0\|cnn\_GOOD | Read | OCL Region | 1 | 1 | 125207 | 125208 | 0.00333333 | 5273.180182 | 5273.180186 |
| 5273.180249 | Kernel\_Read\|xilinx:adm-pcie-7v3:1ddr:3.0-0\|cnn\_GOOD | Read | OCL Region | 1 | 1 | 125227 | 125228 | 0.00333333 | 5273.180249 | 5273.180252 |
| 5273.180312 | Kernel\_Read\|xilinx:adm-pcie-7v3:1ddr:3.0-0\|cnn\_GOOD | Read | OCL Region | 1 | 1 | 125246 | 125247 | 0.00333333 | 5273.180312 | 5273.180316 |
| 5273.180382 | Kernel\_Read\|xilinx:adm-pcie-7v3:1ddr:3.0-0\|cnn\_GOOD | Read | OCL Region | 1 | 1 | 125267 | 125268 | 0.00333333 | 5273.180382 | 5273.180386 |
| 5273.180449 | Kernel\_Read\|xilinx:adm-pcie-7v3:1ddr:3.0-0\|cnn\_GOOD | Read | OCL Region | 1 | 1 | 125287 | 125288 | 0.00333333 | 5273.180449 | 5273.180452 |
| 5273.180526 | Kernel\_Read\|xilinx:adm-pcie-7v3:1ddr:3.0-0\|cnn\_GOOD | Read | OCL Region | 1 | 1 | 125310 | 125311 | 0.00333333 | 5273.180526 | 5273.180529 |
| 5273.180582 | Kernel\_Read\|xilinx:adm-pcie-7v3:1ddr:3.0-0\|cnn\_GOOD | Read | OCL Region | 1 | 1 | 125327 | 125328 | 0.00333333 | 5273.180582 | 5273.180586 |
| 5273.180649 | Kernel\_Read\|xilinx:adm-pcie-7v3:1ddr:3.0-0\|cnn\_GOOD | Read | OCL Region | 1 | 1 | 125347 | 125348 | 0.00333333 | 5273.180649 | 5273.180652 |
| 5273.180926 | Kernel\_Read\|xilinx:adm-pcie-7v3:1ddr:3.0-0\|cnn\_GOOD | Read | OCL Region | 1 | 1 | 125430 | 125431 | 0.00333333 | 5273.180926 | 5273.180929 |
| 5273.180929 | Kernel\_Read\|xilinx:adm-pcie-7v3:1ddr:3.0-0\|cnn\_GOOD | Read | OCL Region | 1 | 1 | 125431 | 125432 | 0.00333333 | 5273.180929 | 5273.180932 |
| 5273.180932 | Kernel\_Read\|xilinx:adm-pcie-7v3:1ddr:3.0-0\|cnn\_GOOD | Read | OCL Region | 1 | 1 | 125432 | 125433 | 0.00333333 | 5273.180932 | 5273.180936 |
| 5273.180942 | Kernel\_Read\|xilinx:adm-pcie-7v3:1ddr:3.0-0\|cnn\_GOOD | Read | OCL Region | 1 | 1 | 125435 | 125436 | 0.00333333 | 5273.180942 | 5273.180946 |
| 5273.180999 | Kernel\_Read\|xilinx:adm-pcie-7v3:1ddr:3.0-0\|cnn\_GOOD | Read | OCL Region | 1 | 1 | 125452 | 125453 | 0.00333333 | 5273.180999 | 5273.181002 |
| 5273.181056 | Kernel\_Read\|xilinx:adm-pcie-7v3:1ddr:3.0-0\|cnn\_GOOD | Read | OCL Region | 1 | 1 | 125469 | 125470 | 0.00333333 | 5273.181056 | 5273.181059 |
| 5273.181112 | Kernel\_Read\|xilinx:adm-pcie-7v3:1ddr:3.0-0\|cnn\_GOOD | Read | OCL Region | 1 | 1 | 125486 | 125487 | 0.00333333 | 5273.181112 | 5273.181116 |
| 5273.181182 | Kernel\_Read\|xilinx:adm-pcie-7v3:1ddr:3.0-0\|cnn\_GOOD | Read | OCL Region | 1 | 1 | 125507 | 125508 | 0.00333333 | 5273.181182 | 5273.181186 |
| 5273.181249 | Kernel\_Read\|xilinx:adm-pcie-7v3:1ddr:3.0-0\|cnn\_GOOD | Read | OCL Region | 1 | 1 | 125527 | 125528 | 0.00333333 | 5273.181249 | 5273.181252 |
| 5273.181316 | Kernel\_Read\|xilinx:adm-pcie-7v3:1ddr:3.0-0\|cnn\_GOOD | Read | OCL Region | 1 | 1 | 125547 | 125548 | 0.00333333 | 5273.181316 | 5273.181319 |
| 5273.181382 | Kernel\_Read\|xilinx:adm-pcie-7v3:1ddr:3.0-0\|cnn\_GOOD | Read | OCL Region | 1 | 1 | 125567 | 125568 | 0.00333333 | 5273.181382 | 5273.181386 |
| 5273.181449 | Kernel\_Read\|xilinx:adm-pcie-7v3:1ddr:3.0-0\|cnn\_GOOD | Read | OCL Region | 1 | 1 | 125587 | 125588 | 0.00333333 | 5273.181449 | 5273.181452 |
| 5273.181512 | Kernel\_Read\|xilinx:adm-pcie-7v3:1ddr:3.0-0\|cnn\_GOOD | Read | OCL Region | 1 | 1 | 125606 | 125607 | 0.00333333 | 5273.181512 | 5273.181516 |
| 5273.181582 | Kernel\_Read\|xilinx:adm-pcie-7v3:1ddr:3.0-0\|cnn\_GOOD | Read | OCL Region | 1 | 1 | 125627 | 125628 | 0.00333333 | 5273.181582 | 5273.181586 |
| 5273.181649 | Kernel\_Read\|xilinx:adm-pcie-7v3:1ddr:3.0-0\|cnn\_GOOD | Read | OCL Region | 1 | 1 | 125647 | 125648 | 0.00333333 | 5273.181649 | 5273.181652 |
| 5273.181712 | Kernel\_Read\|xilinx:adm-pcie-7v3:1ddr:3.0-0\|cnn\_GOOD | Read | OCL Region | 1 | 1 | 125666 | 125667 | 0.00333333 | 5273.181712 | 5273.181716 |
| 5273.181782 | Kernel\_Read\|xilinx:adm-pcie-7v3:1ddr:3.0-0\|cnn\_GOOD | Read | OCL Region | 1 | 1 | 125687 | 125688 | 0.00333333 | 5273.181782 | 5273.181786 |
| 5273.181849 | Kernel\_Read\|xilinx:adm-pcie-7v3:1ddr:3.0-0\|cnn\_GOOD | Read | OCL Region | 1 | 1 | 125707 | 125708 | 0.00333333 | 5273.181849 | 5273.181852 |
| 5273.181912 | Kernel\_Read\|xilinx:adm-pcie-7v3:1ddr:3.0-0\|cnn\_GOOD | Read | OCL Region | 1 | 1 | 125726 | 125727 | 0.00333333 | 5273.181912 | 5273.181916 |
| 5273.181982 | Kernel\_Read\|xilinx:adm-pcie-7v3:1ddr:3.0-0\|cnn\_GOOD | Read | OCL Region | 1 | 1 | 125747 | 125748 | 0.00333333 | 5273.181982 | 5273.181986 |
| 5273.182049 | Kernel\_Read\|xilinx:adm-pcie-7v3:1ddr:3.0-0\|cnn\_GOOD | Read | OCL Region | 1 | 1 | 125767 | 125768 | 0.00333333 | 5273.182049 | 5273.182052 |
| 5273.182116 | Kernel\_Read\|xilinx:adm-pcie-7v3:1ddr:3.0-0\|cnn\_GOOD | Read | OCL Region | 1 | 1 | 125787 | 125788 | 0.00333333 | 5273.182116 | 5273.182119 |
| 5273.182182 | Kernel\_Read\|xilinx:adm-pcie-7v3:1ddr:3.0-0\|cnn\_GOOD | Read | OCL Region | 1 | 1 | 125807 | 125808 | 0.00333333 | 5273.182182 | 5273.182186 |
| 5273.182249 | Kernel\_Read\|xilinx:adm-pcie-7v3:1ddr:3.0-0\|cnn\_GOOD | Read | OCL Region | 1 | 1 | 125827 | 125828 | 0.00333333 | 5273.182249 | 5273.182252 |
| 5273.182312 | Kernel\_Read\|xilinx:adm-pcie-7v3:1ddr:3.0-0\|cnn\_GOOD | Read | OCL Region | 1 | 1 | 125846 | 125847 | 0.00333333 | 5273.182312 | 5273.182316 |
| 5273.182382 | Kernel\_Read\|xilinx:adm-pcie-7v3:1ddr:3.0-0\|cnn\_GOOD | Read | OCL Region | 1 | 1 | 125867 | 125868 | 0.00333333 | 5273.182382 | 5273.182386 |
| 5273.182449 | Kernel\_Read\|xilinx:adm-pcie-7v3:1ddr:3.0-0\|cnn\_GOOD | Read | OCL Region | 1 | 1 | 125887 | 125888 | 0.00333333 | 5273.182449 | 5273.182452 |
| 5273.182512 | Kernel\_Read\|xilinx:adm-pcie-7v3:1ddr:3.0-0\|cnn\_GOOD | Read | OCL Region | 1 | 1 | 125906 | 125907 | 0.00333333 | 5273.182512 | 5273.182516 |
| 5273.182582 | Kernel\_Read\|xilinx:adm-pcie-7v3:1ddr:3.0-0\|cnn\_GOOD | Read | OCL Region | 1 | 1 | 125927 | 125928 | 0.00333333 | 5273.182582 | 5273.182586 |
| 5273.182649 | Kernel\_Read\|xilinx:adm-pcie-7v3:1ddr:3.0-0\|cnn\_GOOD | Read | OCL Region | 1 | 1 | 125947 | 125948 | 0.00333333 | 5273.182649 | 5273.182652 |
| 5273.182712 | Kernel\_Read\|xilinx:adm-pcie-7v3:1ddr:3.0-0\|cnn\_GOOD | Read | OCL Region | 1 | 1 | 125966 | 125967 | 0.00333333 | 5273.182712 | 5273.182716 |
| 5273.182782 | Kernel\_Read\|xilinx:adm-pcie-7v3:1ddr:3.0-0\|cnn\_GOOD | Read | OCL Region | 1 | 1 | 125987 | 125988 | 0.00333333 | 5273.182782 | 5273.182786 |
| 5273.182849 | Kernel\_Read\|xilinx:adm-pcie-7v3:1ddr:3.0-0\|cnn\_GOOD | Read | OCL Region | 1 | 1 | 126007 | 126008 | 0.00333333 | 5273.182849 | 5273.182852 |
| 5273.182916 | Kernel\_Read\|xilinx:adm-pcie-7v3:1ddr:3.0-0\|cnn\_GOOD | Read | OCL Region | 1 | 1 | 126027 | 126028 | 0.00333333 | 5273.182916 | 5273.182919 |
| 5273.182982 | Kernel\_Read\|xilinx:adm-pcie-7v3:1ddr:3.0-0\|cnn\_GOOD | Read | OCL Region | 1 | 1 | 126047 | 126048 | 0.00333333 | 5273.182982 | 5273.182986 |
| 5273.183049 | Kernel\_Read\|xilinx:adm-pcie-7v3:1ddr:3.0-0\|cnn\_GOOD | Read | OCL Region | 1 | 1 | 126067 | 126068 | 0.00333333 | 5273.183049 | 5273.183052 |
| 5273.183112 | Kernel\_Read\|xilinx:adm-pcie-7v3:1ddr:3.0-0\|cnn\_GOOD | Read | OCL Region | 1 | 1 | 126086 | 126087 | 0.00333333 | 5273.183112 | 5273.183116 |
| 5273.183182 | Kernel\_Read\|xilinx:adm-pcie-7v3:1ddr:3.0-0\|cnn\_GOOD | Read | OCL Region | 1 | 1 | 126107 | 126108 | 0.00333333 | 5273.183182 | 5273.183186 |
| 5273.183249 | Kernel\_Read\|xilinx:adm-pcie-7v3:1ddr:3.0-0\|cnn\_GOOD | Read | OCL Region | 1 | 1 | 126127 | 126128 | 0.00333333 | 5273.183249 | 5273.183252 |
| 5273.183312 | Kernel\_Read\|xilinx:adm-pcie-7v3:1ddr:3.0-0\|cnn\_GOOD | Read | OCL Region | 1 | 1 | 126146 | 126147 | 0.00333333 | 5273.183312 | 5273.183316 |
| 5273.183382 | Kernel\_Read\|xilinx:adm-pcie-7v3:1ddr:3.0-0\|cnn\_GOOD | Read | OCL Region | 1 | 1 | 126167 | 126168 | 0.00333333 | 5273.183382 | 5273.183386 |
| 5273.183449 | Kernel\_Read\|xilinx:adm-pcie-7v3:1ddr:3.0-0\|cnn\_GOOD | Read | OCL Region | 1 | 1 | 126187 | 126188 | 0.00333333 | 5273.183449 | 5273.183452 |
| 5273.183516 | Kernel\_Read\|xilinx:adm-pcie-7v3:1ddr:3.0-0\|cnn\_GOOD | Read | OCL Region | 1 | 1 | 126207 | 126208 | 0.00333333 | 5273.183516 | 5273.183519 |
| 5273.183582 | Kernel\_Read\|xilinx:adm-pcie-7v3:1ddr:3.0-0\|cnn\_GOOD | Read | OCL Region | 1 | 1 | 126227 | 126228 | 0.00333333 | 5273.183582 | 5273.183586 |
| 5273.183649 | Kernel\_Read\|xilinx:adm-pcie-7v3:1ddr:3.0-0\|cnn\_GOOD | Read | OCL Region | 1 | 1 | 126247 | 126248 | 0.00333333 | 5273.183649 | 5273.183652 |
| 5273.183716 | Kernel\_Read\|xilinx:adm-pcie-7v3:1ddr:3.0-0\|cnn\_GOOD | Read | OCL Region | 1 | 1 | 126267 | 126268 | 0.00333333 | 5273.183716 | 5273.183719 |
| 5273.183782 | Kernel\_Read\|xilinx:adm-pcie-7v3:1ddr:3.0-0\|cnn\_GOOD | Read | OCL Region | 1 | 1 | 126287 | 126288 | 0.00333333 | 5273.183782 | 5273.183786 |
| 5273.183849 | Kernel\_Read\|xilinx:adm-pcie-7v3:1ddr:3.0-0\|cnn\_GOOD | Read | OCL Region | 1 | 1 | 126307 | 126308 | 0.00333333 | 5273.183849 | 5273.183852 |
| 5273.183912 | Kernel\_Read\|xilinx:adm-pcie-7v3:1ddr:3.0-0\|cnn\_GOOD | Read | OCL Region | 1 | 1 | 126326 | 126327 | 0.00333333 | 5273.183912 | 5273.183916 |
| 5273.183982 | Kernel\_Read\|xilinx:adm-pcie-7v3:1ddr:3.0-0\|cnn\_GOOD | Read | OCL Region | 1 | 1 | 126347 | 126348 | 0.00333333 | 5273.183982 | 5273.183986 |
| 5273.184049 | Kernel\_Read\|xilinx:adm-pcie-7v3:1ddr:3.0-0\|cnn\_GOOD | Read | OCL Region | 1 | 1 | 126367 | 126368 | 0.00333333 | 5273.184049 | 5273.184052 |
| 5273.184116 | Kernel\_Read\|xilinx:adm-pcie-7v3:1ddr:3.0-0\|cnn\_GOOD | Read | OCL Region | 1 | 1 | 126387 | 126388 | 0.00333333 | 5273.184116 | 5273.184119 |
| 5273.184192 | Kernel\_Read\|xilinx:adm-pcie-7v3:1ddr:3.0-0\|cnn\_GOOD | Read | OCL Region | 1 | 1 | 126410 | 126411 | 0.00333333 | 5273.184192 | 5273.184196 |
| 5273.184249 | Kernel\_Read\|xilinx:adm-pcie-7v3:1ddr:3.0-0\|cnn\_GOOD | Read | OCL Region | 1 | 1 | 126427 | 126428 | 0.00333333 | 5273.184249 | 5273.184252 |
| 5273.184316 | Kernel\_Read\|xilinx:adm-pcie-7v3:1ddr:3.0-0\|cnn\_GOOD | Read | OCL Region | 1 | 1 | 126447 | 126448 | 0.00333333 | 5273.184316 | 5273.184319 |
| 5273.184382 | Kernel\_Read\|xilinx:adm-pcie-7v3:1ddr:3.0-0\|cnn\_GOOD | Read | OCL Region | 1 | 1 | 126467 | 126468 | 0.00333333 | 5273.184382 | 5273.184386 |
| 5273.184449 | Kernel\_Read\|xilinx:adm-pcie-7v3:1ddr:3.0-0\|cnn\_GOOD | Read | OCL Region | 1 | 1 | 126487 | 126488 | 0.00333333 | 5273.184449 | 5273.184452 |
| 5273.184516 | Kernel\_Read\|xilinx:adm-pcie-7v3:1ddr:3.0-0\|cnn\_GOOD | Read | OCL Region | 1 | 1 | 126507 | 126508 | 0.00333333 | 5273.184516 | 5273.184519 |
| 5273.184582 | Kernel\_Read\|xilinx:adm-pcie-7v3:1ddr:3.0-0\|cnn\_GOOD | Read | OCL Region | 1 | 1 | 126527 | 126528 | 0.00333333 | 5273.184582 | 5273.184586 |
| 5273.184649 | Kernel\_Read\|xilinx:adm-pcie-7v3:1ddr:3.0-0\|cnn\_GOOD | Read | OCL Region | 1 | 1 | 126547 | 126548 | 0.00333333 | 5273.184649 | 5273.184652 |
| 5273.184712 | Kernel\_Read\|xilinx:adm-pcie-7v3:1ddr:3.0-0\|cnn\_GOOD | Read | OCL Region | 1 | 1 | 126566 | 126567 | 0.00333333 | 5273.184712 | 5273.184716 |
| 5273.184782 | Kernel\_Read\|xilinx:adm-pcie-7v3:1ddr:3.0-0\|cnn\_GOOD | Read | OCL Region | 1 | 1 | 126587 | 126588 | 0.00333333 | 5273.184782 | 5273.184786 |
| 5273.184849 | Kernel\_Read\|xilinx:adm-pcie-7v3:1ddr:3.0-0\|cnn\_GOOD | Read | OCL Region | 1 | 1 | 126607 | 126608 | 0.00333333 | 5273.184849 | 5273.184852 |
| 5273.184912 | Kernel\_Read\|xilinx:adm-pcie-7v3:1ddr:3.0-0\|cnn\_GOOD | Read | OCL Region | 1 | 1 | 126626 | 126627 | 0.00333333 | 5273.184912 | 5273.184916 |
| 5273.184979 | Kernel\_Read\|xilinx:adm-pcie-7v3:1ddr:3.0-0\|cnn\_GOOD | Read | OCL Region | 1 | 1 | 126646 | 126647 | 0.00333333 | 5273.184979 | 5273.184982 |
| 5273.185049 | Kernel\_Read\|xilinx:adm-pcie-7v3:1ddr:3.0-0\|cnn\_GOOD | Read | OCL Region | 1 | 1 | 126667 | 126668 | 0.00333333 | 5273.185049 | 5273.185052 |
| 5273.185116 | Kernel\_Read\|xilinx:adm-pcie-7v3:1ddr:3.0-0\|cnn\_GOOD | Read | OCL Region | 1 | 1 | 126687 | 126688 | 0.00333333 | 5273.185116 | 5273.185119 |
| 5273.185182 | Kernel\_Read\|xilinx:adm-pcie-7v3:1ddr:3.0-0\|cnn\_GOOD | Read | OCL Region | 1 | 1 | 126707 | 126708 | 0.00333333 | 5273.185182 | 5273.185186 |
| 5273.185249 | Kernel\_Read\|xilinx:adm-pcie-7v3:1ddr:3.0-0\|cnn\_GOOD | Read | OCL Region | 1 | 1 | 126727 | 126728 | 0.00333333 | 5273.185249 | 5273.185252 |
| 5273.185316 | Kernel\_Read\|xilinx:adm-pcie-7v3:1ddr:3.0-0\|cnn\_GOOD | Read | OCL Region | 1 | 1 | 126747 | 126748 | 0.00333333 | 5273.185316 | 5273.185319 |
| 5273.185382 | Kernel\_Read\|xilinx:adm-pcie-7v3:1ddr:3.0-0\|cnn\_GOOD | Read | OCL Region | 1 | 1 | 126767 | 126768 | 0.00333333 | 5273.185382 | 5273.185386 |
| 5273.185449 | Kernel\_Read\|xilinx:adm-pcie-7v3:1ddr:3.0-0\|cnn\_GOOD | Read | OCL Region | 1 | 1 | 126787 | 126788 | 0.00333333 | 5273.185449 | 5273.185452 |
| 5273.185516 | Kernel\_Read\|xilinx:adm-pcie-7v3:1ddr:3.0-0\|cnn\_GOOD | Read | OCL Region | 1 | 1 | 126807 | 126808 | 0.00333333 | 5273.185516 | 5273.185519 |
| 5273.185582 | Kernel\_Read\|xilinx:adm-pcie-7v3:1ddr:3.0-0\|cnn\_GOOD | Read | OCL Region | 1 | 1 | 126827 | 126828 | 0.00333333 | 5273.185582 | 5273.185586 |
| 5273.185649 | Kernel\_Read\|xilinx:adm-pcie-7v3:1ddr:3.0-0\|cnn\_GOOD | Read | OCL Region | 1 | 1 | 126847 | 126848 | 0.00333333 | 5273.185649 | 5273.185652 |
| 5273.185716 | Kernel\_Read\|xilinx:adm-pcie-7v3:1ddr:3.0-0\|cnn\_GOOD | Read | OCL Region | 1 | 1 | 126867 | 126868 | 0.00333333 | 5273.185716 | 5273.185719 |
| 5273.185782 | Kernel\_Read\|xilinx:adm-pcie-7v3:1ddr:3.0-0\|cnn\_GOOD | Read | OCL Region | 1 | 1 | 126887 | 126888 | 0.00333333 | 5273.185782 | 5273.185786 |
| 5273.185849 | Kernel\_Read\|xilinx:adm-pcie-7v3:1ddr:3.0-0\|cnn\_GOOD | Read | OCL Region | 1 | 1 | 126907 | 126908 | 0.00333333 | 5273.185849 | 5273.185852 |
| 5273.185916 | Kernel\_Read\|xilinx:adm-pcie-7v3:1ddr:3.0-0\|cnn\_GOOD | Read | OCL Region | 1 | 1 | 126927 | 126928 | 0.00333333 | 5273.185916 | 5273.185919 |
| 5273.185982 | Kernel\_Read\|xilinx:adm-pcie-7v3:1ddr:3.0-0\|cnn\_GOOD | Read | OCL Region | 1 | 1 | 126947 | 126948 | 0.00333333 | 5273.185982 | 5273.185986 |
| 5273.186049 | Kernel\_Read\|xilinx:adm-pcie-7v3:1ddr:3.0-0\|cnn\_GOOD | Read | OCL Region | 1 | 1 | 126967 | 126968 | 0.00333333 | 5273.186049 | 5273.186052 |
| 5273.186116 | Kernel\_Read\|xilinx:adm-pcie-7v3:1ddr:3.0-0\|cnn\_GOOD | Read | OCL Region | 1 | 1 | 126987 | 126988 | 0.00333333 | 5273.186116 | 5273.186119 |
| 5273.186182 | Kernel\_Read\|xilinx:adm-pcie-7v3:1ddr:3.0-0\|cnn\_GOOD | Read | OCL Region | 1 | 1 | 127007 | 127008 | 0.00333333 | 5273.186182 | 5273.186186 |
| 5273.186249 | Kernel\_Read\|xilinx:adm-pcie-7v3:1ddr:3.0-0\|cnn\_GOOD | Read | OCL Region | 1 | 1 | 127027 | 127028 | 0.00333333 | 5273.186249 | 5273.186252 |
| 5273.186316 | Kernel\_Read\|xilinx:adm-pcie-7v3:1ddr:3.0-0\|cnn\_GOOD | Read | OCL Region | 1 | 1 | 127047 | 127048 | 0.00333333 | 5273.186316 | 5273.186319 |
| 5273.186382 | Kernel\_Read\|xilinx:adm-pcie-7v3:1ddr:3.0-0\|cnn\_GOOD | Read | OCL Region | 1 | 1 | 127067 | 127068 | 0.00333333 | 5273.186382 | 5273.186386 |
| 5273.186449 | Kernel\_Read\|xilinx:adm-pcie-7v3:1ddr:3.0-0\|cnn\_GOOD | Read | OCL Region | 1 | 1 | 127087 | 127088 | 0.00333333 | 5273.186449 | 5273.186452 |
| 5273.186512 | Kernel\_Read\|xilinx:adm-pcie-7v3:1ddr:3.0-0\|cnn\_GOOD | Read | OCL Region | 1 | 1 | 127106 | 127107 | 0.00333333 | 5273.186512 | 5273.186516 |
| 5273.186582 | Kernel\_Read\|xilinx:adm-pcie-7v3:1ddr:3.0-0\|cnn\_GOOD | Read | OCL Region | 1 | 1 | 127127 | 127128 | 0.00333333 | 5273.186582 | 5273.186586 |
| 5273.186666 | Kernel\_Read\|xilinx:adm-pcie-7v3:1ddr:3.0-0\|cnn\_GOOD | Read | OCL Region | 1 | 1 | 127152 | 127153 | 0.00333333 | 5273.186666 | 5273.186669 |
| 5273.186722 | Kernel\_Read\|xilinx:adm-pcie-7v3:1ddr:3.0-0\|cnn\_GOOD | Read | OCL Region | 1 | 1 | 127169 | 127170 | 0.00333333 | 5273.186722 | 5273.186726 |
| 5273.186782 | Kernel\_Read\|xilinx:adm-pcie-7v3:1ddr:3.0-0\|cnn\_GOOD | Read | OCL Region | 1 | 1 | 127187 | 127188 | 0.00333333 | 5273.186782 | 5273.186786 |
| 5273.186849 | Kernel\_Read\|xilinx:adm-pcie-7v3:1ddr:3.0-0\|cnn\_GOOD | Read | OCL Region | 1 | 1 | 127207 | 127208 | 0.00333333 | 5273.186849 | 5273.186852 |
| 5273.186916 | Kernel\_Read\|xilinx:adm-pcie-7v3:1ddr:3.0-0\|cnn\_GOOD | Read | OCL Region | 1 | 1 | 127227 | 127228 | 0.00333333 | 5273.186916 | 5273.186919 |
| 5273.187192 | Kernel\_Read\|xilinx:adm-pcie-7v3:1ddr:3.0-0\|cnn\_GOOD | Read | OCL Region | 1 | 1 | 127310 | 127311 | 0.00333333 | 5273.187192 | 5273.187196 |
| 5273.187196 | Kernel\_Read\|xilinx:adm-pcie-7v3:1ddr:3.0-0\|cnn\_GOOD | Read | OCL Region | 1 | 1 | 127311 | 127312 | 0.00333333 | 5273.187196 | 5273.187199 |
| 5273.187199 | Kernel\_Read\|xilinx:adm-pcie-7v3:1ddr:3.0-0\|cnn\_GOOD | Read | OCL Region | 1 | 1 | 127312 | 127313 | 0.00333333 | 5273.187199 | 5273.187202 |
| 5273.187206 | Kernel\_Read\|xilinx:adm-pcie-7v3:1ddr:3.0-0\|cnn\_GOOD | Read | OCL Region | 1 | 1 | 127314 | 127315 | 0.00333333 | 5273.187206 | 5273.187209 |
| 5273.187266 | Kernel\_Read\|xilinx:adm-pcie-7v3:1ddr:3.0-0\|cnn\_GOOD | Read | OCL Region | 1 | 1 | 127332 | 127333 | 0.00333333 | 5273.187266 | 5273.187269 |
| 5273.187322 | Kernel\_Read\|xilinx:adm-pcie-7v3:1ddr:3.0-0\|cnn\_GOOD | Read | OCL Region | 1 | 1 | 127349 | 127350 | 0.00333333 | 5273.187322 | 5273.187326 |
| 5273.187382 | Kernel\_Read\|xilinx:adm-pcie-7v3:1ddr:3.0-0\|cnn\_GOOD | Read | OCL Region | 1 | 1 | 127367 | 127368 | 0.00333333 | 5273.187382 | 5273.187386 |
| 5273.187449 | Kernel\_Read\|xilinx:adm-pcie-7v3:1ddr:3.0-0\|cnn\_GOOD | Read | OCL Region | 1 | 1 | 127387 | 127388 | 0.00333333 | 5273.187449 | 5273.187452 |
| 5273.187516 | Kernel\_Read\|xilinx:adm-pcie-7v3:1ddr:3.0-0\|cnn\_GOOD | Read | OCL Region | 1 | 1 | 127407 | 127408 | 0.00333333 | 5273.187516 | 5273.187519 |
| 5273.187582 | Kernel\_Read\|xilinx:adm-pcie-7v3:1ddr:3.0-0\|cnn\_GOOD | Read | OCL Region | 1 | 1 | 127427 | 127428 | 0.00333333 | 5273.187582 | 5273.187586 |
| 5273.187649 | Kernel\_Read\|xilinx:adm-pcie-7v3:1ddr:3.0-0\|cnn\_GOOD | Read | OCL Region | 1 | 1 | 127447 | 127448 | 0.00333333 | 5273.187649 | 5273.187652 |
| 5273.187716 | Kernel\_Read\|xilinx:adm-pcie-7v3:1ddr:3.0-0\|cnn\_GOOD | Read | OCL Region | 1 | 1 | 127467 | 127468 | 0.00333333 | 5273.187716 | 5273.187719 |
| 5273.187782 | Kernel\_Read\|xilinx:adm-pcie-7v3:1ddr:3.0-0\|cnn\_GOOD | Read | OCL Region | 1 | 1 | 127487 | 127488 | 0.00333333 | 5273.187782 | 5273.187786 |
| 5273.187849 | Kernel\_Read\|xilinx:adm-pcie-7v3:1ddr:3.0-0\|cnn\_GOOD | Read | OCL Region | 1 | 1 | 127507 | 127508 | 0.00333333 | 5273.187849 | 5273.187852 |
| 5273.187916 | Kernel\_Read\|xilinx:adm-pcie-7v3:1ddr:3.0-0\|cnn\_GOOD | Read | OCL Region | 1 | 1 | 127527 | 127528 | 0.00333333 | 5273.187916 | 5273.187919 |
| 5273.187982 | Kernel\_Read\|xilinx:adm-pcie-7v3:1ddr:3.0-0\|cnn\_GOOD | Read | OCL Region | 1 | 1 | 127547 | 127548 | 0.00333333 | 5273.187982 | 5273.187986 |
| 5273.188049 | Kernel\_Read\|xilinx:adm-pcie-7v3:1ddr:3.0-0\|cnn\_GOOD | Read | OCL Region | 1 | 1 | 127567 | 127568 | 0.00333333 | 5273.188049 | 5273.188052 |
| 5273.188116 | Kernel\_Read\|xilinx:adm-pcie-7v3:1ddr:3.0-0\|cnn\_GOOD | Read | OCL Region | 1 | 1 | 127587 | 127588 | 0.00333333 | 5273.188116 | 5273.188119 |
| 5273.188182 | Kernel\_Read\|xilinx:adm-pcie-7v3:1ddr:3.0-0\|cnn\_GOOD | Read | OCL Region | 1 | 1 | 127607 | 127608 | 0.00333333 | 5273.188182 | 5273.188186 |
| 5273.188249 | Kernel\_Read\|xilinx:adm-pcie-7v3:1ddr:3.0-0\|cnn\_GOOD | Read | OCL Region | 1 | 1 | 127627 | 127628 | 0.00333333 | 5273.188249 | 5273.188252 |
| 5273.188316 | Kernel\_Read\|xilinx:adm-pcie-7v3:1ddr:3.0-0\|cnn\_GOOD | Read | OCL Region | 1 | 1 | 127647 | 127648 | 0.00333333 | 5273.188316 | 5273.188319 |
| 5273.188382 | Kernel\_Read\|xilinx:adm-pcie-7v3:1ddr:3.0-0\|cnn\_GOOD | Read | OCL Region | 1 | 1 | 127667 | 127668 | 0.00333333 | 5273.188382 | 5273.188386 |
| 5273.188449 | Kernel\_Read\|xilinx:adm-pcie-7v3:1ddr:3.0-0\|cnn\_GOOD | Read | OCL Region | 1 | 1 | 127687 | 127688 | 0.00333333 | 5273.188449 | 5273.188452 |
| 5273.188516 | Kernel\_Read\|xilinx:adm-pcie-7v3:1ddr:3.0-0\|cnn\_GOOD | Read | OCL Region | 1 | 1 | 127707 | 127708 | 0.00333333 | 5273.188516 | 5273.188519 |
| 5273.188582 | Kernel\_Read\|xilinx:adm-pcie-7v3:1ddr:3.0-0\|cnn\_GOOD | Read | OCL Region | 1 | 1 | 127727 | 127728 | 0.00333333 | 5273.188582 | 5273.188586 |
| 5273.188649 | Kernel\_Read\|xilinx:adm-pcie-7v3:1ddr:3.0-0\|cnn\_GOOD | Read | OCL Region | 1 | 1 | 127747 | 127748 | 0.00333333 | 5273.188649 | 5273.188652 |
| 5273.188716 | Kernel\_Read\|xilinx:adm-pcie-7v3:1ddr:3.0-0\|cnn\_GOOD | Read | OCL Region | 1 | 1 | 127767 | 127768 | 0.00333333 | 5273.188716 | 5273.188719 |
| 5273.188779 | Kernel\_Read\|xilinx:adm-pcie-7v3:1ddr:3.0-0\|cnn\_GOOD | Read | OCL Region | 1 | 1 | 127786 | 127787 | 0.00333333 | 5273.188779 | 5273.188782 |
| 5273.188849 | Kernel\_Read\|xilinx:adm-pcie-7v3:1ddr:3.0-0\|cnn\_GOOD | Read | OCL Region | 1 | 1 | 127807 | 127808 | 0.00333333 | 5273.188849 | 5273.188852 |
| 5273.188916 | Kernel\_Read\|xilinx:adm-pcie-7v3:1ddr:3.0-0\|cnn\_GOOD | Read | OCL Region | 1 | 1 | 127827 | 127828 | 0.00333333 | 5273.188916 | 5273.188919 |
| 5273.188982 | Kernel\_Read\|xilinx:adm-pcie-7v3:1ddr:3.0-0\|cnn\_GOOD | Read | OCL Region | 1 | 1 | 127847 | 127848 | 0.00333333 | 5273.188982 | 5273.188986 |
| 5273.189049 | Kernel\_Read\|xilinx:adm-pcie-7v3:1ddr:3.0-0\|cnn\_GOOD | Read | OCL Region | 1 | 1 | 127867 | 127868 | 0.00333333 | 5273.189049 | 5273.189052 |
| 5273.189116 | Kernel\_Read\|xilinx:adm-pcie-7v3:1ddr:3.0-0\|cnn\_GOOD | Read | OCL Region | 1 | 1 | 127887 | 127888 | 0.00333333 | 5273.189116 | 5273.189119 |
| 5273.189182 | Kernel\_Read\|xilinx:adm-pcie-7v3:1ddr:3.0-0\|cnn\_GOOD | Read | OCL Region | 1 | 1 | 127907 | 127908 | 0.00333333 | 5273.189182 | 5273.189186 |
| 5273.189249 | Kernel\_Read\|xilinx:adm-pcie-7v3:1ddr:3.0-0\|cnn\_GOOD | Read | OCL Region | 1 | 1 | 127927 | 127928 | 0.00333333 | 5273.189249 | 5273.189252 |
| 5273.189316 | Kernel\_Read\|xilinx:adm-pcie-7v3:1ddr:3.0-0\|cnn\_GOOD | Read | OCL Region | 1 | 1 | 127947 | 127948 | 0.00333333 | 5273.189316 | 5273.189319 |
| 5273.189382 | Kernel\_Read\|xilinx:adm-pcie-7v3:1ddr:3.0-0\|cnn\_GOOD | Read | OCL Region | 1 | 1 | 127967 | 127968 | 0.00333333 | 5273.189382 | 5273.189386 |
| 5273.189449 | Kernel\_Read\|xilinx:adm-pcie-7v3:1ddr:3.0-0\|cnn\_GOOD | Read | OCL Region | 1 | 1 | 127987 | 127988 | 0.00333333 | 5273.189449 | 5273.189452 |
| 5273.189516 | Kernel\_Read\|xilinx:adm-pcie-7v3:1ddr:3.0-0\|cnn\_GOOD | Read | OCL Region | 1 | 1 | 128007 | 128008 | 0.00333333 | 5273.189516 | 5273.189519 |
| 5273.189582 | Kernel\_Read\|xilinx:adm-pcie-7v3:1ddr:3.0-0\|cnn\_GOOD | Read | OCL Region | 1 | 1 | 128027 | 128028 | 0.00333333 | 5273.189582 | 5273.189586 |
| 5273.189649 | Kernel\_Read\|xilinx:adm-pcie-7v3:1ddr:3.0-0\|cnn\_GOOD | Read | OCL Region | 1 | 1 | 128047 | 128048 | 0.00333333 | 5273.189649 | 5273.189652 |
| 5273.189716 | Kernel\_Read\|xilinx:adm-pcie-7v3:1ddr:3.0-0\|cnn\_GOOD | Read | OCL Region | 1 | 1 | 128067 | 128068 | 0.00333333 | 5273.189716 | 5273.189719 |
| 5273.189782 | Kernel\_Read\|xilinx:adm-pcie-7v3:1ddr:3.0-0\|cnn\_GOOD | Read | OCL Region | 1 | 1 | 128087 | 128088 | 0.00333333 | 5273.189782 | 5273.189786 |
| 5273.189849 | Kernel\_Read\|xilinx:adm-pcie-7v3:1ddr:3.0-0\|cnn\_GOOD | Read | OCL Region | 1 | 1 | 128107 | 128108 | 0.00333333 | 5273.189849 | 5273.189852 |
| 5273.189916 | Kernel\_Read\|xilinx:adm-pcie-7v3:1ddr:3.0-0\|cnn\_GOOD | Read | OCL Region | 1 | 1 | 128127 | 128128 | 0.00333333 | 5273.189916 | 5273.189919 |
| 5273.189982 | Kernel\_Read\|xilinx:adm-pcie-7v3:1ddr:3.0-0\|cnn\_GOOD | Read | OCL Region | 1 | 1 | 128147 | 128148 | 0.00333333 | 5273.189982 | 5273.189986 |
| 5273.190049 | Kernel\_Read\|xilinx:adm-pcie-7v3:1ddr:3.0-0\|cnn\_GOOD | Read | OCL Region | 1 | 1 | 128167 | 128168 | 0.00333333 | 5273.190049 | 5273.190052 |
| 5273.190116 | Kernel\_Read\|xilinx:adm-pcie-7v3:1ddr:3.0-0\|cnn\_GOOD | Read | OCL Region | 1 | 1 | 128187 | 128188 | 0.00333333 | 5273.190116 | 5273.190119 |
| 5273.190182 | Kernel\_Read\|xilinx:adm-pcie-7v3:1ddr:3.0-0\|cnn\_GOOD | Read | OCL Region | 1 | 1 | 128207 | 128208 | 0.00333333 | 5273.190182 | 5273.190186 |
| 5273.190249 | Kernel\_Read\|xilinx:adm-pcie-7v3:1ddr:3.0-0\|cnn\_GOOD | Read | OCL Region | 1 | 1 | 128227 | 128228 | 0.00333333 | 5273.190249 | 5273.190252 |
| 5273.190316 | Kernel\_Read\|xilinx:adm-pcie-7v3:1ddr:3.0-0\|cnn\_GOOD | Read | OCL Region | 1 | 1 | 128247 | 128248 | 0.00333333 | 5273.190316 | 5273.190319 |
| 5273.190382 | Kernel\_Read\|xilinx:adm-pcie-7v3:1ddr:3.0-0\|cnn\_GOOD | Read | OCL Region | 1 | 1 | 128267 | 128268 | 0.00333333 | 5273.190382 | 5273.190386 |
| 5273.190459 | Kernel\_Read\|xilinx:adm-pcie-7v3:1ddr:3.0-0\|cnn\_GOOD | Read | OCL Region | 1 | 1 | 128290 | 128291 | 0.00333333 | 5273.190459 | 5273.190462 |
| 5273.190516 | Kernel\_Read\|xilinx:adm-pcie-7v3:1ddr:3.0-0\|cnn\_GOOD | Read | OCL Region | 1 | 1 | 128307 | 128308 | 0.00333333 | 5273.190516 | 5273.190519 |
| 5273.190582 | Kernel\_Read\|xilinx:adm-pcie-7v3:1ddr:3.0-0\|cnn\_GOOD | Read | OCL Region | 1 | 1 | 128327 | 128328 | 0.00333333 | 5273.190582 | 5273.190586 |
| 5273.190649 | Kernel\_Read\|xilinx:adm-pcie-7v3:1ddr:3.0-0\|cnn\_GOOD | Read | OCL Region | 1 | 1 | 128347 | 128348 | 0.00333333 | 5273.190649 | 5273.190652 |
| 5273.190716 | Kernel\_Read\|xilinx:adm-pcie-7v3:1ddr:3.0-0\|cnn\_GOOD | Read | OCL Region | 1 | 1 | 128367 | 128368 | 0.00333333 | 5273.190716 | 5273.190719 |
| 5273.190782 | Kernel\_Read\|xilinx:adm-pcie-7v3:1ddr:3.0-0\|cnn\_GOOD | Read | OCL Region | 1 | 1 | 128387 | 128388 | 0.00333333 | 5273.190782 | 5273.190786 |
| 5273.190849 | Kernel\_Read\|xilinx:adm-pcie-7v3:1ddr:3.0-0\|cnn\_GOOD | Read | OCL Region | 1 | 1 | 128407 | 128408 | 0.00333333 | 5273.190849 | 5273.190852 |
| 5273.190916 | Kernel\_Read\|xilinx:adm-pcie-7v3:1ddr:3.0-0\|cnn\_GOOD | Read | OCL Region | 1 | 1 | 128427 | 128428 | 0.00333333 | 5273.190916 | 5273.190919 |
| 5273.190982 | Kernel\_Read\|xilinx:adm-pcie-7v3:1ddr:3.0-0\|cnn\_GOOD | Read | OCL Region | 1 | 1 | 128447 | 128448 | 0.00333333 | 5273.190982 | 5273.190986 |
| 5273.191049 | Kernel\_Read\|xilinx:adm-pcie-7v3:1ddr:3.0-0\|cnn\_GOOD | Read | OCL Region | 1 | 1 | 128467 | 128468 | 0.00333333 | 5273.191049 | 5273.191052 |
| 5273.191116 | Kernel\_Read\|xilinx:adm-pcie-7v3:1ddr:3.0-0\|cnn\_GOOD | Read | OCL Region | 1 | 1 | 128487 | 128488 | 0.00333333 | 5273.191116 | 5273.191119 |
| 5273.191182 | Kernel\_Read\|xilinx:adm-pcie-7v3:1ddr:3.0-0\|cnn\_GOOD | Read | OCL Region | 1 | 1 | 128507 | 128508 | 0.00333333 | 5273.191182 | 5273.191186 |
| 5273.191249 | Kernel\_Read\|xilinx:adm-pcie-7v3:1ddr:3.0-0\|cnn\_GOOD | Read | OCL Region | 1 | 1 | 128527 | 128528 | 0.00333333 | 5273.191249 | 5273.191252 |
