# Supplementary material for: Research on OpenCL optimization for FPGA deep learning application
Source: PLoS One. 2019 Oct 10;14(10):e0222984. doi: 10.1371/journal.pone.0222984 (PMC6786543; doi:10.1371/journal.pone.0222984)
Supplement: S1 File — (Xilinx and this paper). (ZIP) [file pone.0222984.s001.zip › SupportingFiles/ optimized data.zip!/ optimized data/sdaccel_profile_summary.html]

# SDAccel Profile Summary

  

### Generated on: 2018-02-01 14:59:09

### Profiled application: Xilinx\_fpgacnn\_burst.exe

### Target platform: Xilinx

### Target devices: xilinx:adm-pcie-7v3:1ddr:3.0-0

### Flow mode: System Run

### Tool version: 2016.3

  
  

## OpenCL API Calls

| API Name | Number Of Calls | Total Time (ms) | Minimum Time (ms) | Average Time (ms) | Maximum Time (ms) |
| --- | --- | --- | --- | --- | --- |
| clCreateProgramWithBinary | 1 | 3221.95 | 3221.95 | 3221.95 | 3221.95 |
| clCreateContext | 1 | 2026.01 | 2026.01 | 2026.01 | 2026.01 |
| clFinish | 1 | 11.6365 | 11.6365 | 11.6365 | 11.6365 |
| clEnqueueWriteBuffer | 2 | 2.47228 | 0.659632 | 1.23614 | 1.81265 |
| clEnqueueReadBuffer | 1 | 1.13796 | 1.13796 | 1.13796 | 1.13796 |
| clSetKernelArg | 6 | 0.13416 | 0.019733 | 0.02236 | 0.032815 |
| clCreateBuffer | 3 | 0.129311 | 0.027455 | 0.0431037 | 0.066446 |
| clGetPlatformIDs | 3 | 0.122201 | 0.01093 | 0.0407337 | 0.057066 |
| clEnqueueNDRangeKernel | 1 | 0.108759 | 0.108759 | 0.108759 | 0.108759 |
| clReleaseMemObject | 3 | 0.068496 | 0.01946 | 0.022832 | 0.028683 |
| clCreateKernel | 1 | 0.055963 | 0.055963 | 0.055963 | 0.055963 |
| clGetEventProfilingInfo | 2 | 0.045214 | 0.019867 | 0.022607 | 0.025347 |
| clBuildProgram | 1 | 0.03297 | 0.03297 | 0.03297 | 0.03297 |
| clCreateCommandQueue | 1 | 0.027714 | 0.027714 | 0.027714 | 0.027714 |
| clGetDeviceInfo | 2 | 0.0261 | 0.010978 | 0.01305 | 0.015122 |
| clGetPlatformInfo | 2 | 0.025295 | 0.011065 | 0.0126475 | 0.01423 |
| clReleaseKernel | 1 | 0.02142 | 0.02142 | 0.02142 | 0.02142 |
| clReleaseCommandQueue | 1 | 0.021387 | 0.021387 | 0.021387 | 0.021387 |
| clReleaseProgram | 1 | 0.021324 | 0.021324 | 0.021324 | 0.021324 |
| clReleaseContext | 1 | 0.020967 | 0.020967 | 0.020967 | 0.020967 |
| clGetDeviceIDs | 1 | 0.013678 | 0.013678 | 0.013678 | 0.013678 |

  

## Kernel Execution

| Kernel | Number Of Enqueues | Total Time (ms) | Minimum Time (ms) | Average Time (ms) | Maximum Time (ms) |
| --- | --- | --- | --- | --- | --- |
| good\_cnn | 1 | 10.886 | 10.886 | 10.886 | 10.886 |

  

## Compute Unit Utilization

| Device | Compute Unit | Kernel | Global Work Size | Local Work Size | Number Of Calls | Total Time (ms) | Minimum Time (ms) | Average Time (ms) | Maximum Time (ms) |
| --- | --- | --- | --- | --- | --- | --- | --- | --- | --- |
| xilinx:adm-pcie-7v3:1ddr:3.0-0 | good\_cnn\_1 | good\_cnn | 6:1:1 | 1:1:1 | 1 | 9.59365 | 9.59365 | 9.59365 | 9.59365 |
| xilinx:adm-pcie-7v3:1ddr:3.0-0 | good\_cnn\_2 | good\_cnn | 6:1:1 | 1:1:1 | 1 | 9.99453 | 9.99453 | 9.99453 | 9.99453 |
| xilinx:adm-pcie-7v3:1ddr:3.0-0 | good\_cnn\_3 | good\_cnn | 6:1:1 | 1:1:1 | 1 | 10.2538 | 10.2538 | 10.2538 | 10.2538 |
| xilinx:adm-pcie-7v3:1ddr:3.0-0 | good\_cnn\_4 | good\_cnn | 6:1:1 | 1:1:1 | 1 | 9.9835 | 9.9835 | 9.9835 | 9.9835 |
| xilinx:adm-pcie-7v3:1ddr:3.0-0 | good\_cnn\_5 | good\_cnn | 6:1:1 | 1:1:1 | 1 | 10.1155 | 10.1155 | 10.1155 | 10.1155 |
| xilinx:adm-pcie-7v3:1ddr:3.0-0 | good\_cnn\_6 | good\_cnn | 6:1:1 | 1:1:1 | 1 | 10.1653 | 10.1653 | 10.1653 | 10.1653 |

  

## Data Transfer: Host and Global Memory

| Context:Number of Devices | Transfer Type | Number Of Transfers | Transfer Rate (MB/s) | Average Bandwidth Utilization (%) | Average Size (KB) | Total Time (ms) | Average Time (ms) |
| --- | --- | --- | --- | --- | --- | --- | --- |
| context0:1 | READ | 1 | 854.922387 | 8.905442 | 746.496 | 0.873174 | 0.873174 |
| context0:1 | WRITE | 2 | 763.220052 | 7.950209 | 684.384 | 1.793412 | 0.896706 |

  

## Data Transfer: Kernels and Global Memory

| Device | Transfer Type | Number Of Transfers | Transfer Rate (MB/s) | Average Bandwidth Utilization (%) | Average Size (KB) | Average Time (ns) |
| --- | --- | --- | --- | --- | --- | --- |
| xilinx:adm-pcie-7v3:1ddr:3.0-0 | READ | 32322 | 190.024 | 1.64951 | 0.064 | 1760.34 |
| xilinx:adm-pcie-7v3:1ddr:3.0-0 | WRITE | 11863 | 68.5737 | 0.595258 | 0.0629264 | 39.0072 |

  

## Top Data Transfer: Kernels and Global Memory

| Device | Kernel Name | Number of Transfers | Average Bytes per Transfer | Transfer Efficiency (%) | Total Data Transfer (MB) | Total Write (MB) | Total Read (MB) | Transfer Rate (MB/s) | Average Bandwidth Utilization (%) |
| --- | --- | --- | --- | --- | --- | --- | --- | --- | --- |
| xilinx:adm-pcie-7v3:1ddr:3.0-0 | ALL | 44185 | 63.7118 | 1.55546 | 2.8151 | 0.746496 | 2.06861 | 129.299 | 1.12239 |
